# Supplementary material for: Catalytic diazene synthesis from sterically hindered amines for deaminative functionalization
Source: Nat Commun. 2025 Jul 7;16:6266. doi: 10.1038/s41467-025-61662-9 (PMC12234842; doi:10.1038/s41467-025-61662-9)
Supplement: Supplementary file 1 — Supplementary Information [file 41467_2025_61662_MOESM1_ESM.pdf]

Supplementary Information

Catalytic Diazene Synthesis from Sterically Hindered Amines for Deaminative Functionalization

Taro Tsuji,[1] Isora Fukumoto,[1] Takara Hario,[1] Mikihiro Hayashi,[2] Ayumi Osawa,[1] Takashi Ohshima,[1]* and Ryo Yazaki[1,3]*

[1] *Graduate School of Pharmaceutical Sciences, Kyushu University, Fukuoka 812-8582, Japan*
[2] *Department of Life Science and Applied Chemistry Graduate School of Engineering, Nagoya Institute of Technology, Aichi 466–8555, Japan.*
[3] *Institute for Advanced Study, Kyushu University, Fukuoka 819-0395, Japan*
ohshima@phar.kyushu-u.ac.jp, yazaki@phar.kyushu-u.ac.jp

1. General
2. Instrumentation
3. Materials
4. Preparation of Starting Materials
5. General Procedure and Characterization of the Products
6. Transformation of the Products
7. Mechanistic Study
8. Optimization Study
9. DFT Calculation
10. Functional Group Evaluation (FGE) Kit Evaluation
11. Aminyl Radical Detection
12. Thermogravimetric Analysis
13. Reference
14. NMR Spectra of New Compounds

1. General

All reactions were run under argon atmosphere unless otherwise noted. Air- and moisture- sensitive liquids were transferred via a syringe and a stainless-steel needle. Reactions were magnetically stirred and monitored by thin layer chromatography using Merck Silica Gel 60 F254 plates. All work-up and purification procedures were carried out with reagent-grade solvents under ambient atmosphere. Flash chromatography was performed using silica gel 60N (spherical neutral, particle size 40–50 nm) purchased from Kanto Chemical Co.

Ltd. Alternatively, automated flash chromatography (EPCLC Smart Flash, EPCLC-AI-580S, Yamazen Corporation) was performed using Sfär Silica (HC D High Capacity Duo 20 µm) purchased from Biotage.

2. Instrumentation

Nuclear magnetic resonance (NMR) spectra were acquired on 500 MHz Bruker Avance III spectrometers. ^{1}H and ^{13}C{^{1}H} NMR chemical shifts are reported in ppm and referenced to tetramethylsilane or residual solvent peaks as internal standards (for $CDCl_3$, tetramethylsilane 0 ppm for ^{1}H and ^{13}C{^{1}H}; for DMSO-d_6, 2.50 ppm for ^{1}H and 39.52 ppm for ^{13}C{^{1}H}; for Acetone-d_6, 2.05 ppm for ^{1}H and 29.84 ppm for ^{13}C{^{1}H}). NMR data are reported as follows: chemical shifts, multiplicity (s: singlet, d: doublet, dd: doublet of doublets, t: triplet, q: quartet, quin: quintet, sep: septet, m: multiplet, br: broad signal), coupling constant (Hz), and integration. Infrared (IR) spectra were recorded on with Shimadzu IR Affinity-1S. High-resolution mass spectroscopy (HRMS) was obtained with IonSense DART-OS (DART) of Shimazu LCMS-9030 or with ESI of Shimazu LCMS-9030. Fmoc solid-phase peptide synthesis was performed by Biotage® Initiator+ Alstra™. High performance liquid chromatography (HPLC) was performed with Shimadzu LC-20AR pump and SPD-M40 photo diode array detector. HPLC analysis was performed with YMC-Triart C18 column (20 ϕ x 250 mm). Optical rotation was measured with JASCO P2200 polarimeter.

3. Materials

DBU ((1,8-diazabicyclo[5.4.0]undec-7-ene)) was distilled after dried with CaH_2 and stored in schlenk flask. Almost all solvents were dried with MS4A. Methanol was dried with MS3A. CuOAc was purchased from Wako (StremChemicals, Inc.) and used as received (sublimed grade, ≥99.9% trace metals basis). CuOAc was stored in a dry box. DBDMH (1,3-Dibromo-5,5-dimethylhydantoin) was purchased from TCI and used as received. All other commercially available reagents were used as received. All other commercially available reagents were used as received.

4. Preparation of Starting Materials

General Procedure (1a, 1c, 1d and 1o) [1]

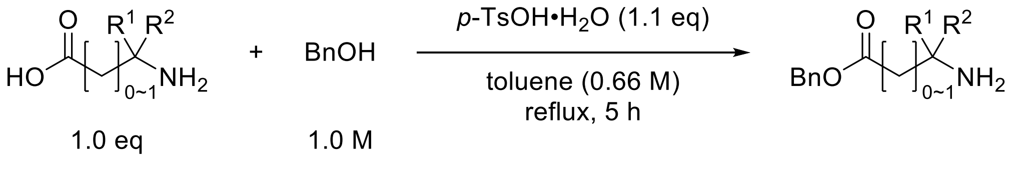

The appropriate amino acid (1.0 equiv.), p-toluene sulfonic acid monohydrate (1.1 equiv.) and benzyl alcohol (1.0 M) were heated at reflux in toluene (0.66 M) with Dean-Stark trap for 5 h. On cooling to room temperature, Hexane/EtOAc = 1/1 were added and the mixture was left for 30 min then filtrated and washed with Hexane/EtOAc = 1/1. The solid was dissolved in DCM and washed with sat. Na_2CO_3 aq. The organic layer was dried over Na_2SO_4, filtered and the solvent removed under reduced pressure to give an oil.

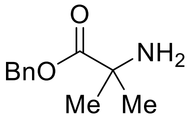

Benzyl 2-amino-2-methylpropanoate (1a): CAS Registry Number 55456-40-1 (Colorless oil, 50 mmol scale: 7.8607 g, 81% yield); ^{1}H NMR (500 MHz, $CDCl_3$) δ 7.39–7.33 (m, 5H, Ar*H*), 5.14 (s, 2H, ArC*H₂*), 1.66 (br, 2H, N*H₂*), 1.37 (s, 6H, C*H₃*); ^{13}C NMR (125 MHz, $CDCl_3$) δ 177.9, 136.0, 128.6, 128.2, 127.9, 66.7, 54.8, 27.7.

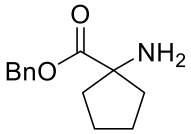

Benzyl 1-aminocyclopentane-1-carboxylate (1c): CAS Registry Number 5471-59-0 (colorless oil, 10 mmol scale: 1.9744 g, 90% yield); ^{1}H NMR (500 MHz, $CDCl_3$, 27 °C) δ 7.39–7.31 (m, 5H, Ar*H*), 5.15 (s, 2H, ArC*H₂*), 2.14–2.09 (m, 2H, CCH_2CH_2), 1.92–1.82 (m, 2H, CCH_2CH_2), 1.81–1.71 (m, 2H, CCH_2CH_2), 1.63–1.59 (m, 4H, CCH_2CH_2, NH_2); ^{13}C NMR (125 MHz, $CDCl_3$, 27 °C) δ 178.1, 136.0, 128.6, 128.2, 128.0, 66.7, 65.0, 39.6, 25.0.

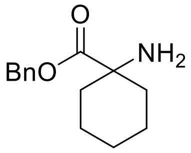

Benzyl 1-aminocyclohexane-1-carboxylate (1d): CAS Registry Number 102373-23-9 (colorless oil, 10 mmol scale: 1.8894 g, 81% yield); ^{1}H NMR (500 MHz, $CDCl_3$, 27 °C) δ 7.39–7.31 (m, 5H, Ar*H*), 5.15 (s, 2H, ArC*H₂*), 1.98–1.92 (m, 2H, $CCH_2CH_2CH_2$), 1.69–1.62 (m, 2H, $CCH_2CH_2CH_2$), 1.58 (br, 2H, NH_2), 1.51–1.43 (m, 5H, $CCH_2CH_2CH_2$), 1.41–1.35 (m, 1H, $CCH_2CH_2CH_2$); ^{13}C NMR (125 MHz, $CDCl_3$, 27 °C) δ 177.2, 136.1, 128.6, 128.2, 128.0, 66.5, 57.4, 35.3, 25.4, 21.9.

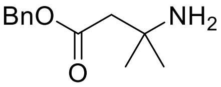

Benzyl 3-amino-3-methylbutanoate (1o): CAS Registry Number 91563-87-0 (colorless oil, 20 mmol scale: 2.68 g, 65% yield); ^{1}H NMR (500 MHz, $CDCl_3$, 27 °C) δ 7.37–7.31 (m, 5H, Ar*H*), 5.13 (s, 2H, OCH_2), 2.45 (s, 2H, CCH_2), 1.60 (br, 2H, NH_2), 1.20 (s, 6H, CH_3); ^{13}C NMR (125 MHz, $CDCl_3$, 27 °C) δ 171.8, 135.9, 128.6, 128.3, 128.3, 66.1, 49.1, 48.6, 30.7.

Procedure (1g, 1h) [2]

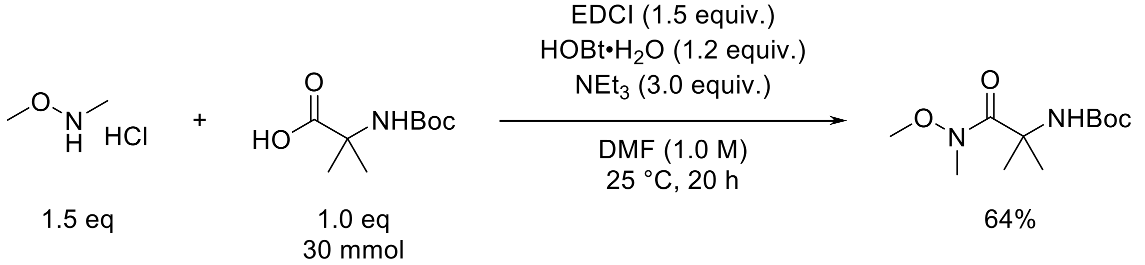

To a solution of 2-((*tert*-butoxycarbonyl)amino)-2-methylpropanoic acid (6.10 g, 30 mmol), *N,O*-dimethylhydroxylamine hydrochloride (4.39 g, 45 mmol), NEt_3 (12.5 mL, 90 mmol), and HOBt monohydrate (5.51 g, 36 mmol) in DMF (30 mL) was added EDCI (8.63 g, 45 mmol). The mixture was stirred at 25 °C for 20 h. The mixture was treated with water (300ml) and extracted with Hexane/EtOAc = 1/1 (300ml). The organic layer was separated, dried over Na_2SO_4, and concentrated under vacuum. The resultant mixture was purified by silica gel flash chromatography (Hexane/EtOAc = 30/1 to 10/1 to 4/1) to obtain product.

***tert*-butyl (1-(methoxy(methyl)amino)-2-methyl-1-oxopropan-2-yl)carbamate:** CAS Registry Number 160816-27-3 (White solid, 64% yield, 4.7346 g); ^1H NMR (500 MHz, $CDCl_3$) δ 5.24 (br, 1H, N*H*), 3.68 (s, 3H, OC*H*3), 3.21 (s, 3H, NC*H*3), 1.55 (s, 6H, CC*H*3), 1.44 (s, 9H, CC*H*3).

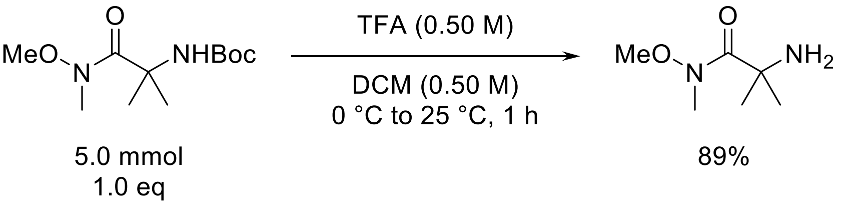

The Boc protected Weinreb amide (1.23 g, 5.0 mmol) was taken up in DCM (0.50 M, 10 mL) and cooled by ice bath. Then TFA (0.50 M, 10 ml) was added and stirred at 25 °C for 1 h. The solvent was removed under reduced pressure. The mixture was dissolved in DCM (300 ml) and washed with sat. Na_2CO_3 aq. (100 ml). The organic layer was dried over Na_2SO_4, filtered and the solvent removed under reduced pressure to give an oil.

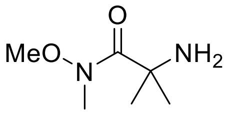

2-Amino-*N*-methoxy-*N*,2-dimethylpropanamide (1g): CAS Registry Number 500168-13-8 (Pale yellow oil, 89% yield, 652.2 mg); ^1H NMR (500 MHz, $CDCl_3$) δ 3.72 (s, 3H, OC*H*3), 3.22 (s, 3H, NC*H*3), 1.85 (br, 2H, N*H*2), 1.38 (s, 6H, CC*H*3); ^{13}C NMR (125 MHz, $CDCl_3$, 27 °C) δ 178.4, 60.7, 55.5, 33.8, 27.2.

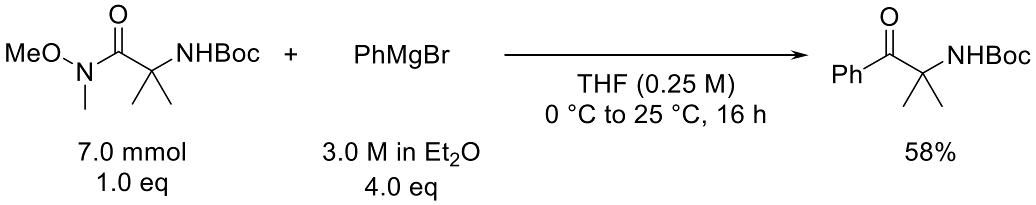

To a solution of Boc protected Weinreb amide (1.72 g, 7.0 mmol) in THF (28 mL) was added dropwise phenylmagnesium bromide (9.3 mL, 3.0 M in Et_2O, 28 mmol) at 0 °C. The mixture was stirred at 25 °C for 16 h. The mixture was quenched with sat. NH_4Cl aq. at −40 °C and extracted with DCM. The organic layer was separated, dried over Na_2SO_4, and concentrated under vacuum. The residue was purified by column chromatography (*n*-Hexane/Ethyl acetate = 20/1 to 10/1) to yield the white solid.

***tert*-Butyl (2-methyl-1-oxo-1-phenylpropan-2-yl)carbamate:** CAS Registry Number 870095-47-9 (White solid, 58% yield, 1.08 g); ^{1}H NMR (500 MHz, $CDCl_3$) atropisomer mixture, major: δ 7.96 (br, 2H, Ar*H*), 7.39 (br, 3H, Ar*H*), 5.21 (br, 1H, N*H*), 1.61 (s, 6H, CCH_3), 1.24 (s, 9H, CCH_3); minor: δ 8.12 (br, 2H, Ar*H*), 7.47 (br, 3H, Ar*H*), 4.95 (br, 1H, N*H*), 1.61 (s, 6H, CCH_3), 1.11 (s, 9H, CCH_3).

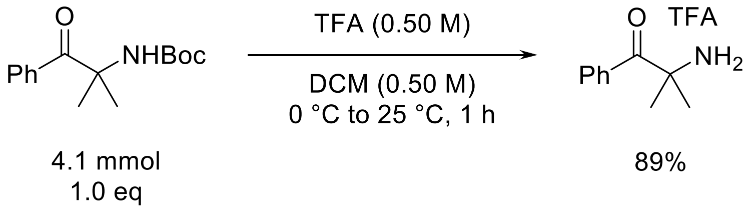

The Boc protected ketone (1.1 g, 4.1 mmol) was taken up in DCM (0.50 M, 8.2 mL) and cooled by ice bath. Then TFA (0.50 M, 8.2 ml) was added and stirred at 25 °C for 1 h. The solvent was removed under reduced pressure to give a solid. This was filtrated, washed with Et_2O and dried to give the pure TFA salt.

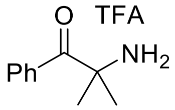

2-Amino-2-methyl-1-phenylpropan-1-one 2,2,2-trifluoroacetate (1:1) (1h): CAS Registry Number 870095-49-1 (White solid, 89% yield, 1.02 g); ^{1}H NMR (500 MHz, DMSO) δ 8.46 (br, 3H, NH_3), 7.99 (dd, 2H, *J* = 1.0, 8.0 Hz, Ar*H*), 7.71 (t, 1H, *J* = 7.5 Hz, Ar*H*), 7.58 (dd, 2H, *J* = 7.5, 7.5 Hz, Ar*H*), 1.72 (s, 6H, CH_3); ^{19}F NMR (470 MHz, DMSO) δ −73.6; HRMS (DART) *m/z* calc'd. for $C_{10}H_{13}NO$ ($PhC(O)C(Me)_2NH_2$ + H)$^+$ 164.1070, found 164.1071.

Procedure (1s)

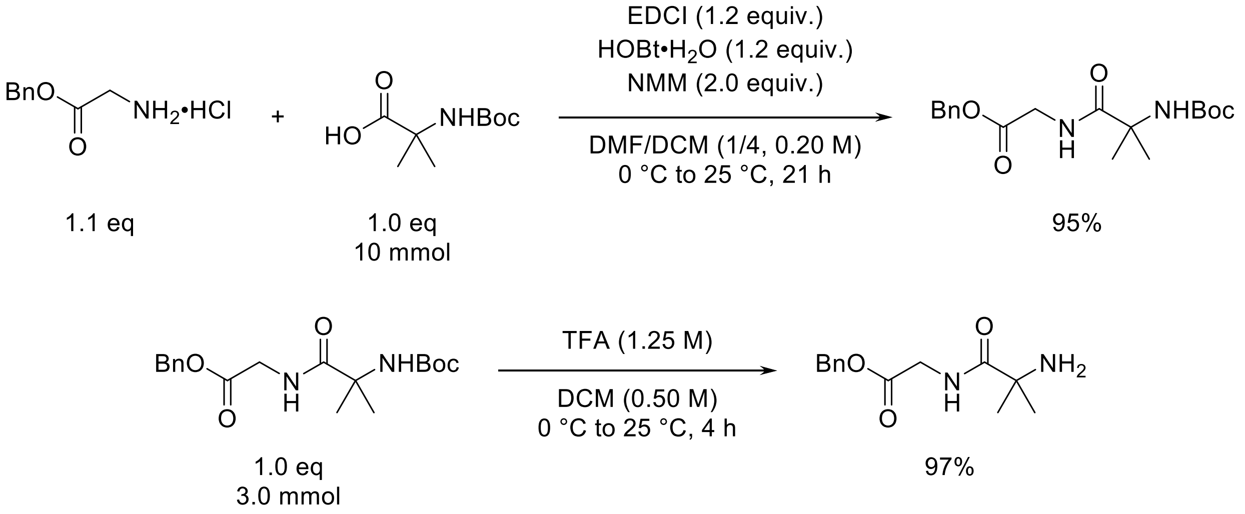

STEP 1: To a solution of *N*-Boc amino acid (1.0 eq.), amine hydrochloride (1.1 eq.), *N*-methylmorphiline (2.0 eq.), and HOBt monohydrate (1.2 eq.) in DMF/DCM (1/4, 0.20 M) was added EDCI (1.2 eq.) in an ice bath. The mixture was stirred overnight at 25 °C. The mixture was diluted with EtOAc and washed by water, sat. Na_2CO_3 aq., 1 M HCl aq. and water. The organic layer was separated, dried over Na_2SO_4, and concentrated under vacuum. The desired product was obtained, which was used in the next step without further purification.

STEP 2: The appropriate amino acid (1.0 eq.) was taken up in DCM (0.50 M) and cooled by ice bath. Then TFA (1.25 M) was added and stirred at 25 °C for 4 h. The mixture was diluted with DCM and washed by sat. Na_2CO_3 aq.. The organic layer was separated, dried over Na_2SO_4, and concentrated under vacuum to give the desired product. If impurities were present, it was purified by flash column chromatography to afford the desired compound.

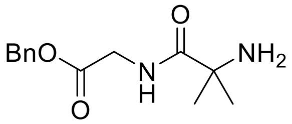

Benzyl (2-amino-2-methylpropanoyl)glycinate (1s): CAS Registry Number 1588635-01-1 (STEP 1: 10 mmol scale, White solid, 95%, 3.3153 g. STEP 2: 3.0 mmol scale, Pale yellow solid, 97%, 728.8 mg); ^{1}H NMR (500 MHz, $CDCl_3$) δ 8.08 (br, 1H, N*H*), 7.39–7.32 (m, 5H, Ar*H*), 5.18 (s, 2H, OC*H₂*), 4.06 (d, *J* = 5.6 Hz, 2H, NC*H₂*), 1.60 (br, 2H, N*H₂*), 1.37 (s, 6H, C*H₃*); ^{13}C NMR (125 MHz, $CDCl_3$, 27 °C) δ 177.9, 170.0, 135.3, 128.6, 128.5, 128.4, 67.1, 54.9, 41.3, 29.1.

General Procedure (1t–1v)

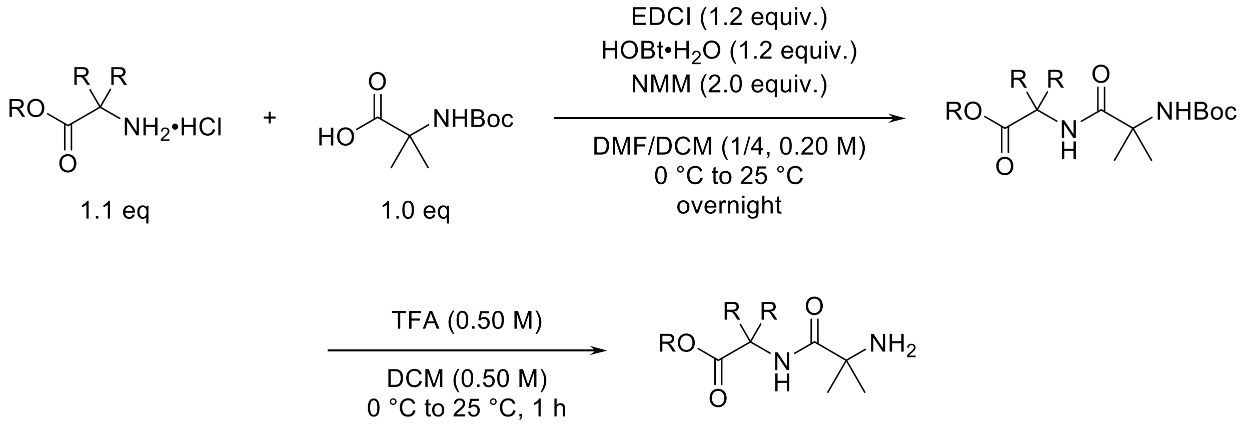

STEP 1: To a solution of *N*-Boc amino acid (1.0 eq.), amine hydrochloride (1.1 eq.), *N*-methylmorphiline (2.0 eq.), and HOBt monohydrate (1.2 eq.) in DMF/DCM (1/4, 0.20 M) was added EDCI (1.2 eq.) in an ice bath. The mixture was stirred overnight at 25 °C. The mixture was diluted with EtOAc and washed by water, sat. Na_2CO_3 aq., 1 M HCl aq. and water. The organic layer was separated, dried over Na_2SO_4, and concentrated under vacuum. The desired product was obtained, which was used in the next step without further purification.

STEP 2: The appropriate amino acid (1.0 eq.) was taken up in DCM (0.50 M) and cooled by ice bath. Then TFA (0.50 M) was added and stirred at 25 °C for 1 h. The mixture was diluted with DCM and washed by sat. Na_2CO_3 aq.. The organic layer was separated, dried over Na_2SO_4, and concentrated under vacuum. The residue was purified by flash column chromatography to afford the desired compound.

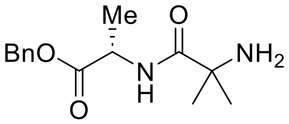

Benzyl (2-amino-2-methylpropanoyl)-*L*-alaninate (1t): CAS Registry Number 16947-00-5 (STEP 1: 10 mmol scale, White solid, >99%, 3.7075 g. STEP 2: 10 mmol scale, White gel, 85%, 2.2569 g); ^{1}H NMR (500 MHz, $CDCl_3$) δ 8.05 (br, *J* = 6.3 Hz, 1H, N*H*), 7.38–7.31 (m, 5H, Ar*H*), 5.17 (dd, *J* = 30.6 Hz, 12.3 Hz, 2H, OC*H₂*), 4.57 (quintet, *J* = 7.4 Hz, 1H, NHC*H*), 1.49 (br, 2H, N*H₂*), 1.42 (d, *J* = 7.3 Hz, 3H, CHC*H₃*), 1.35 (s, 3H, CC*H₃*), 1.34 (s, 3H, CC*H₃*); ^{13}C NMR (125 MHz, $CDCl_3$, 27 °C) δ 177.3, 173.0, 135.5, 128.6, 128.3, 128.1, 67.0, 54.7, 47.9, 29.1, 29.1, 18.3; HRMS (DART) *m/z* calc'd. for $C_{14}H_{20}N_2O_3$ $(M + H)^+$ 265.1547, found 265.1541.

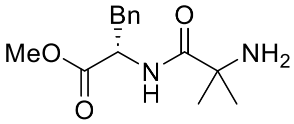

Methyl (2-amino-2-methylpropanoyl)-*L*-phenylalaninate (1u): CAS Registry Number 344607-92-7 (STEP 1: 10 mmol scale, White solid, 99%, 3.5985 g. STEP 2: 10 mmol scale, White gel, 88%, 2.3301 g); ^{1}H NMR (500 MHz, $CDCl_3$) δ 7.99 (br, *J* = 7.5 Hz, 1H, N*H*), 7.28 (t, *J* = 7.2 Hz, 2H, Ar*H*), 7.23 (tt, *J* = 7.3 Hz, 1.7 Hz, 1H, Ar*H*), 7.13 (d, *J* = 7.0 Hz, 2H, Ar*H*), 4.82–4.78 (m, 1H, NHC*H*), 3.72 (s, 3H, OC*H₃*), 3.19–3.04 (m, 2H, CHC*H₂*), 1.39 (br, 2H, N*H₂*), 1.32 (s, 3H, CC*H₃*), 1.25 (s, 3H, CC*H₃*); ^{13}C NMR (125 MHz, $CDCl_3$, 27 °C) δ 177.2, 172.2, 136.2, 129.3, 128.5, 127.0, 54.7, 52.9, 52.2, 38.0, 29.1, 29.0; HRMS (DART) *m/z* calc'd. for $C_{14}H_{20}N_2O_3$ $(M + H)^+$ 265.1547, found 265.1536.

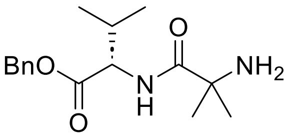

Benzyl (2-amino-2-methylpropanoyl)-*L*-valinate (1v): (STEP 1: 10 mmol scale, White solid, >99%, 3.7075 g. STEP 2: 10 mmol scale, Pale yellow oil, 99%, 2.8814 g); ^{1}H NMR (500 MHz, $CDCl_3$) δ 8.12 (br, 1H, N*H*), 7.38–7.31 (m, 5H, Ar*H*), 5.16 (dd, *J* = 43.3 Hz, 12.2 Hz, 2H, OC*H₂*), 4.51 (q, *J* = 4.7 Hz, 1H, NHC*H*), 2.25–2.18 (m, 1H, CHC*H*), 1.59 (br, 2H, N*H₂*), 1.37 (s, 3H, CC*H₃*), 1.37 (s, 3H, CC*H₃*), 0.92 (d, *J* = 6.9 Hz, 3H, CHC*H₃*), 0.87 (d, *J* = 6.8 Hz, 3H, CHC*H₃*); ^{13}C NMR (125 MHz, $CDCl_3$, 27 °C) δ 177.6, 172.0, 135.5, 128.6, 128.4, 128.3, 66.8, 56.9, 55.0, 31.2, 29.4, 29.0, 19.1, 17.6; IR (neat) 3362, 2965, 2930, 2874, 1736, 1667, 1499, 1456, 1389, 1375, 1358, 1335, 1304, 1261, 1234, 1211, 1180, 1142, 1082, 1001, 974, 910, 831, 795, 750, 696, 673, 621, 600, 586, 567, 523, 501, 482, 453, 440, 428, 420, 409 cm^{-1}; HRMS (DART) *m/z* calc'd. for $C_{16}H_{24}N_2O_3$ $(M + H)^+$ 293.1860, found 293.1859.

Procedure (1w) NH2 free

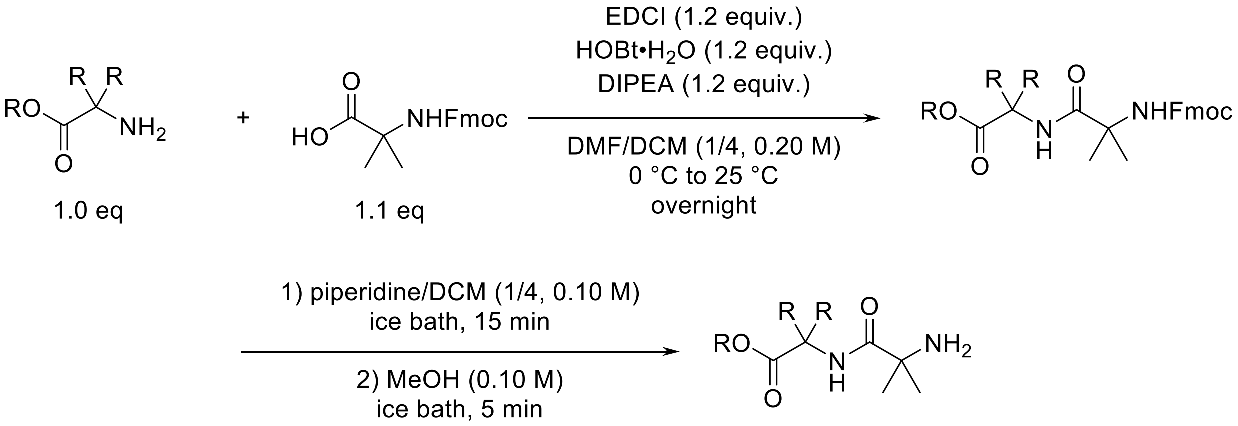

STEP 1: To a solution of *N*-Fmoc amino acid (1.1 eq.), amine (1.0 eq.), *N,N*-Diisopropylethylamine (1.2 eq.), and HOBt monohydrate (1.2 eq.) in DMF/DCM (1/4, 0.20 M) was added EDCI (1.2 eq.) in an ice bath. The mixture was stirred overnight at 25 °C. The mixture was diluted with EtOAc and washed by water, sat. Na_2CO_3 aq., 1 M HCl aq. and water. The organic layer was separated, dried over Na_2SO_4, and concentrated under vacuum. The desired product was obtained, which was used in the next step without further purification.

STEP 2: Fmoc-protected dipeptide derivatives (1.0 eq) were taken up in piperidine/DCM (1/4, 0.10 M) and cooled by ice bath. Stirring was continued for 15 min, then MeOH (0.10 M) was added to the solution. Stirring was continued for 5 min. The solvent was removed using a vacuum evaporator. The residue was purified by flash column chromatography to afford the desired compound.

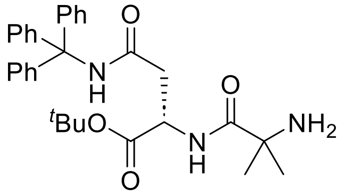

***tert*-Butyl *N*²-(2-amino-2-methylpropanoyl)-*N*⁴-trityl-*L*-asparaginate (1w):** (STEP 1: 2.35 mmol scale, White solid, >99%, 1.7383 g. STEP 2: 2.35 mmol scale, white solid, 87% yield, 99% pure(g/g), 1.0584 g); ^{1}H NMR (500 MHz, $CDCl_3$) δ 8.22 (d, *J* = 7.9 Hz, 1H, CHN*H*), 7.29–7.22 (m, 9H, Ar*H*), 7.19–7.17 (m, 6H, Ar*H*), 6.69 (br, 1H, N*H*), 4.56–4.53 (m, 1H, C*H*), 3.00 (dd, *J* = 16.0 Hz, 4.7 Hz, 1H, C*H₂*), 2.86 (dd, *J* = 16.0 Hz, 4.5 Hz, 1H, C*H₂*), 1.57 (br, 2H, N*H₂*), 1.37 (s, 9H, OCC*H₃*), 1.32 (s, 3H, CC*H₃*), 1.31 (s, 3H, CC*H₃*); ^{13}C NMR (125 MHz, $CDCl_3$, 27 °C) δ 177.6, 169.9, 168.9, 144.5, 128.7, 128.0, 127.0, 82.1, 70.8, 54.7, 49.6, 38.5, 28.9, 28.8, 27.8; IR (neat) 3300, 3186, 1736, 1726, 1659, 1520, 1506, 1449, 1364, 1277, 1242, 1153, 1038, 1003, 972, 849, 770, 743, 702, 638, 625, 573, 478 cm^{-1}; HRMS (DART) *m/z* calc'd. for $C_{31}H_{37}N_3O_4$ $(M + H)^+$ 516.2857, found 516.2854.

General Procedure (1q, 1r) [3,4]

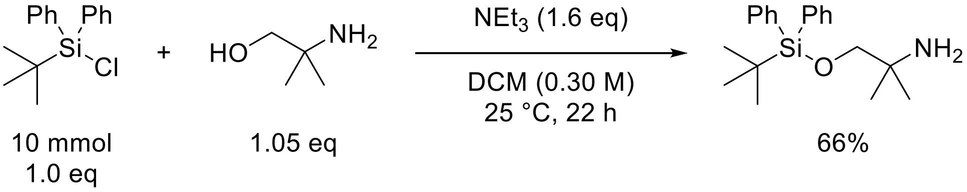

2-Amino-2-methylpropan-1-ol (1.0 ml, 10.5 mmol) was dissolved in DCM (0.30 M, 30 mL). Then, triethylamine (2.2 mL, 16.0 mmol) and *tert*-2 (2.57 mL, 10.0 mmol) were added. The reaction mixture was stirred at 25 °C for 22 hours, quenched with sat. Na_2CO_3 aq. and extracted with DCM. The organic layer was separated, dried over Na_2SO_4, and concentrated in vacuo. The residue was purified by flash column chromatography (gradient elution: DCM/MeOH = 100/0 to 20/1) to afford the desired compound.

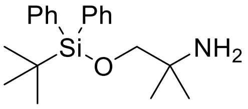

1-((*tert*-Butyldiphenylsilyl)oxy)-2-methylpropan-2-amine (1q): CAS Registry Number 134304-53-3 (White solid, 66%, 2.1665 g); ^{1}H NMR (500 MHz, $CDCl_3$) δ 7.66 (dd, *J* = 8.0 Hz, 1.5 Hz, 4H, Ar*H*), 7.44–7.37 (m, 6H, Ar*H*), 3.37 (s, 2H, OC*H₂*), 1.53 (br, 2H, N*H₂*), 1.09 (s, 6H, NCC*H₃*), 1.08 (s, 9H, SiCC*H₃*); ^{13}C NMR (125 MHz, $CDCl_3$, 27 °C) δ 135.7, 133.6, 129.7, 127.7, 73.8, 51.0, 26.9, 26.9, 19.4.

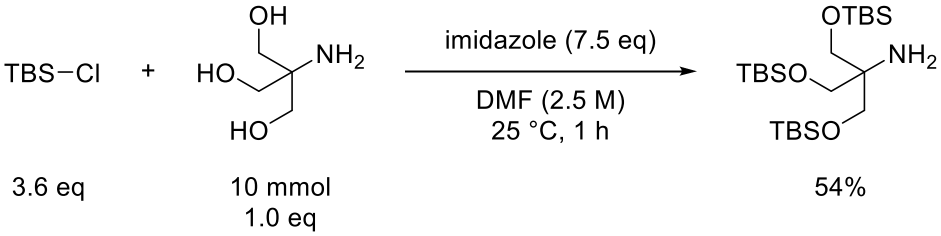

2-Amino-2-(hydroxymethyl)propane-1,3-diol (1.211 g, 10 mmol), *tert*-Butyldimethylchlorosilane (5.426 g, 36 mmol) and imidazole (5.106 g, 75 mmol) were dissolved in DMF (2.5 M, 4 mL). The mixture stirred at 25 °C for 1 h. The product was washed with water, extracted with DCM, dried over anhydrous Na_2SO_4 and filtered. The solvent was removed under reduced pressure and the residue was purified by flash column chromatography (gradient elution: *n*-Hexane/Et_2O = 5/1) to afford the desired compound.

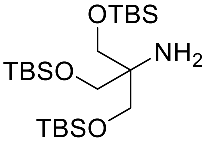

6-(((*tert*-Butyldimethylsilyl)oxy)methyl)-2,2,3,3,9,9,10,10-octamethyl-4,8-dioxa-3,9-disilaundecan-6-amine (1r): CAS Registry Number 102522-47-4 (White solid, 54%, 2.5217 g); ^{1}H NMR (500 MHz, $CDCl_3$) δ 3.45 (s, 6H,

OC*H₂*), 1.55 (br, 2H, N*H₂*), 0.89 (s, 27H, CC*H₃*), 0.04 (s, 18H, SiC*H₃*); ^{13}C NMR (125 MHz, $CDCl_3$, 27 °C) δ 64.1, 57.4, 25.9, 18.2, -5.5.

General Procedure (1i–1m) [5,6,7]

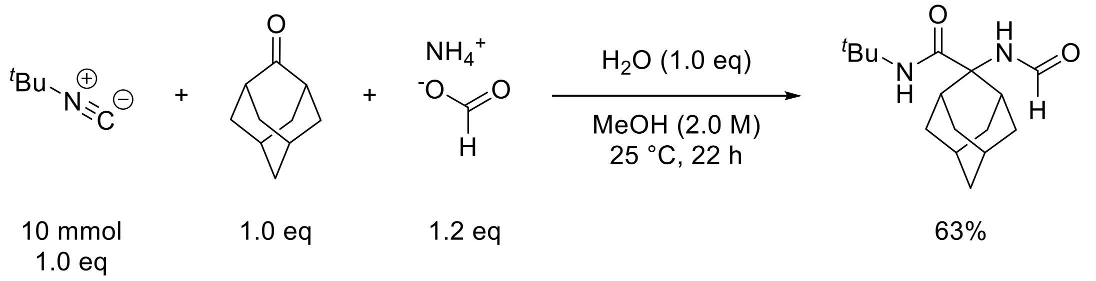

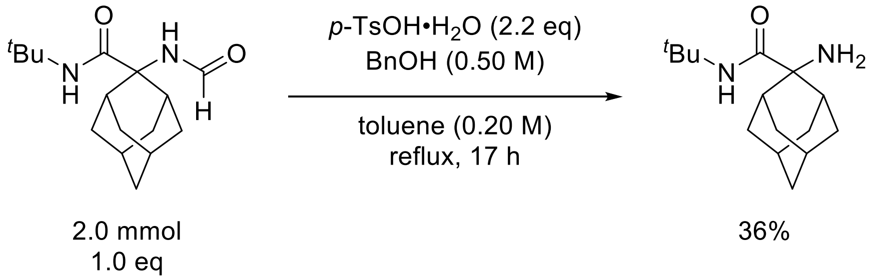

STEP 1: To a solution of adamantan-2-one (1.50 g, 10 mmol, 1.0 eq.) and ammonium formate (757 mg, 12 mmol, 1.2 eq.) in MeOH (5.0 ml, 2.0 M) were added isocyanide (1.1 ml, 10 mmol, 1.0 eq) and water (0.18 ml, 10 mmol, 1.0 eq.). The mixture was stirred at 25 °C for 22 h, then the volatiles were evaporated. The residue was dissolved in DCM, washed with water, dried over Na_2SO_4, and evaporated to give the residue which was purified by recrystallization (EtOAc, 70 °C to 25 °C).

***N*-(tert-butyl)-2-formamidoadamantane-2-carboxamide:** CAS Registry Number 2407915-00-6 (White solid, 63%, 1.7622 g); ^{1}H NMR (500 MHz, $CDCl_3$) δ 8.14 (s, 1H, CO*H*), 6.95 (br, 1H, N*H*), 5.58 (br, 1H, N*H*), 2.64 (br, 2H, Ad*H*), 1.99–1.93 (m, 4H, Ad*H*), 1.87–1.65 (m, 8H, Ad*H*), 1.34 (s, 9H, C*H₃*); ^{13}C NMR (125 MHz, $CDCl_3$, 27 °C) δ 170.7, 161.4, 65.3, 50.9, 37.2, 34.0, 32.5, 32.1, 28.5, 26.6, 26.3.

STEP 2: The *N*-(tert-butyl)-2-formamidoadamantane-2-carboxamide (557 mg, 2.0 mmol, 1.0 eq.), p-toluene sulfonic acid monohydrate (837 mg, 4.4 mmol, 2.2 eq.) and benzyl alcohol (0.50 M) were heated at reflux in toluene (0.20 M) for 17 h. On cooling to room temperature, Hexane/Et_2O = 1/1 were added and the mixture was left for 30 min then filtrated and washed with Hexane/Et_2O = 1/1. The solid was dissolved in DCM and washed with sat. Na_2CO_3 aq. The organic layer was dried over Na_2SO_4, filtered and the solvent removed under reduced pressure. The residue was purified by flash column chromatography (gradient elution: *n*-Hexane/EtOAc = 40/1 to 10/1 to 2/1) to afford the desired compound.

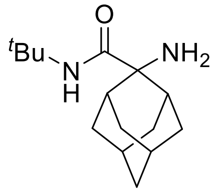

2-Amino-*N*-(tert-butyl)adamantane-2-carboxamide (1i): (White solid, 36% yield, 180.5 mg); ^{1}H NMR (500 MHz, $CDCl_3$) δ 5.84 (br, 1H, N*H*), 2.11 (d, *J* = 12.8 Hz, 2H, Ad*H*), 1.98–1.95 (m, 4H, Ad*H*), 1.81–1.60 (m, 10H, Ad*H*, N*H₂*), 1.36 (s, 9H, C*H₃*); ^{13}C NMR (125 MHz, $CDCl_3$) δ 175.7, 61.2, 50.7, 37.8, 35.3, 35.0, 32.5, 28.7, 26.9, 26.7; IR (neat) 3435, 2970, 2903, 2853, 2025, 1647, 1504, 1445, 1389, 1377, 1350, 1296, 1273, 1227, 1169, 1121, 1101, 1084, 1045, 1028, 1001, 932, 862, 835, 802, 773, 745, 556, 498, 473, 461, 455, 420, 405 cm^{-1}; HRMS (DART) *m/z* calc'd. for $C_{15}H_{26}N_2O$ $(M + H)^+$ 251.2118, found 251.2110.

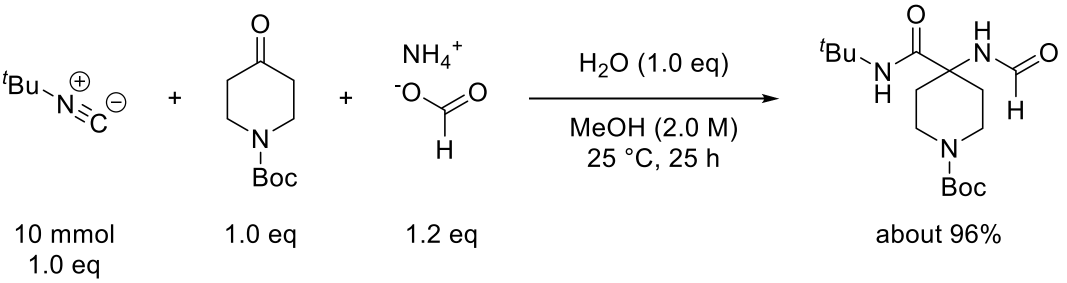

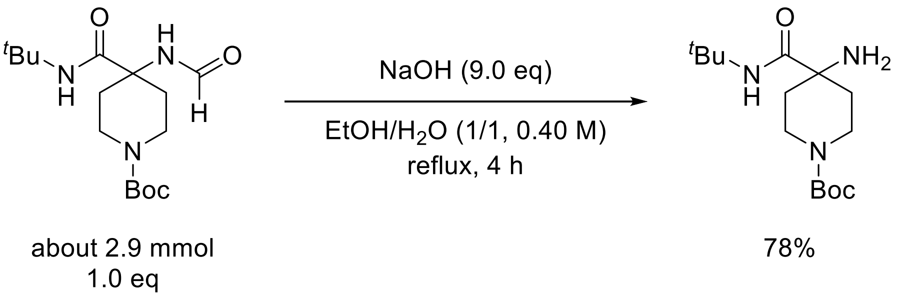

STEP 1: Isocyanide (1.1 ml, 10 mmol, 1.0 eq.) and water (0.18 ml, 10 mmol, 1.0 eq.) were added to a solution of ketone (1.99 g, 10 mmol, 1.0 eq.) and ammonium formate (757 mg, 12 mmol, 1.2 eq.) in MeOH (5.0 ml, 2.0 M). The mixture was stirred at 25 °C for 20 h, then the volatile matter was evaporated. The residue was dissolved in DCM, washed with water, dried over Na_2SO_4 and evaporated to give the crude (Pale yellow solid, about 96% yield, 3.1534 g), which was used in the next step without further purification.

STEP 2: The formyl-protected amine (0.950 g, about 2.9 mmol, 1.0 eq.), NaOH (1.04 g, 26.1 mmol, 9.0 eq) and EtOH/H_2O (7.2 ml, 1/1, 0.40 M) were heated at reflux for 4 h. On cooling to room temperature, the crude was dissolved in DCM and washed with H_2O. The organic layer was dried over Na_2SO_4, filtered and the solvent removed under reduced pressure. The residue was purified by flash column chromatography (gradient elution: *n*-Hexane/EtOAc = 4/1 to 1/1 to 1/2) to afford the desired compound.

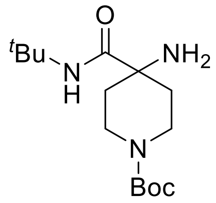

***tert*-Butyl 4-amino-4-(*tert*-butylcarbamoyl)piperidine-1-carboxylate (1j):** (White solid, 78% yield, 0.6758 g); ^{1}H NMR (500 MHz, $CDCl_3$) δ 7.52 (br, 1H, N*H*), 3.94 (br, 2H, NC*H₂*), 3.03 (br, 2H, NC*H₂*), 2.16–2.10 (m, 2H, CC*H₂*), 1.45 (m, 11H, CC*H₃*, N*H₂*), 1.34–1.29 (m, 11H, CC*H₃*, CC*H₂*); ^{13}C NMR (125 MHz, $CDCl_3$) δ 175.6, 154.7, 79.6, 55.6, 50.3, 34.8, 28.7, 28.4; IR (neat) 3387, 3264, 3250, 2961, 2922, 1975, 1676, 1657, 1514, 1481, 1449, 1427, 1391, 1375, 1362, 1346, 1281, 1261, 1248, 1229, 1206, 1159, 1078, 1045, 1007, 974, 962, 941, 926, 878, 860, 833, 804, 768, 714, 694, 638, 559, 534, 478, 465, 426, 411 cm^{-1}; HRMS (DART) *m/z* calc'd. for $C_{30}H_{58}N_6O_6$ (2M + H)$^+$ 599.4491, found 599.4488.

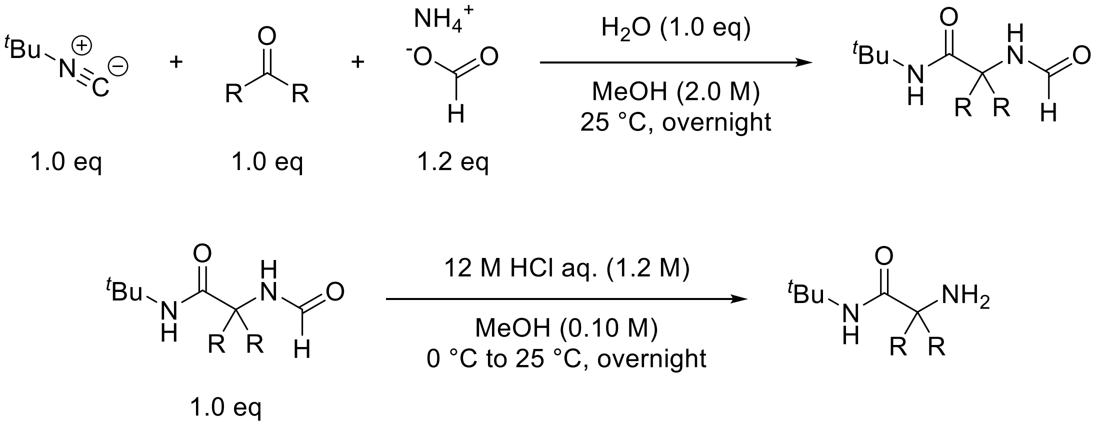

STEP 1: Isocyanide (1.0 eq.) and water (1.0 eq.) were added to a solution of ketone (1.0 eq.) and ammonium formate (1.2 eq.) in MeOH (2.0 M). The mixture was stirred overnight at 25 °C, then the volatile matter was evaporated. The residue was dissolved in DCM, washed with water, dried over Na_2SO_4 and evaporated to give the crude, which was used in the next step without further purification.

STEP 2: To a solution of formyl-protected amine (about 1.0 eq.) in MeOH (0.10 M) was added conc. HCl aq. (1.2 M) with ice bath and the mixture was stirred overnight at 25 °C, then the crude was evaporated. The residue was dissolved in water and washed with DCM. The aqueous layer was made basic by the addition of sat. Na_2CO_3 aq. and extracted with DCM. The organic layer was dried over Na_2SO_4, filtered and the solvent removed under reduced pressure. The residue was purified by flash column chromatography (gradient elution: *n*-Hexane/EtOAc = 4/1 to 1/1 to 1/2) to afford the desired compound.

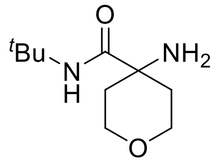

4-Amino-*N*-(*tert*-butyl)tetrahydro-2*H*-pyran-4-carboxamide (1k): CAS Registry Number 285996-64-7 (STEP 1: 10 mmol scale, 15 h, White solid, about 58%, 0.9371 g. STEP 2: about 3.0 mmol scale, 18 h, White solid, 71%, 426.3 mg); ^{1}H NMR (500 MHz, $CDCl_3$) δ 7.54 (br, 1H, N*H*), 3.91 (dt, *J* = 11.8 Hz, 4.0 Hz, 2H, OC*H₂*), 3.63 (td, *J* = 11.6 Hz, 2.5 Hz, 2H, OCH_2), 2.32–2.27 (m, 2H, CCH_2), 1.47 (br, 2H, NH_2), 1.35 (s, 9H, CCH_3), 1.27–1.24 (m, 2H, CCH_2); ^{13}C NMR (125 MHz, $CDCl_3$, 27 °C) δ 175.6, 63.5, 54.7, 50.2, 35.4, 28.7; HRMS (DART) *m/z* calc'd. for $C_{10}H_{20}N_2O_2$ (M + H)$^+$ 201.1598, found 201.1600.

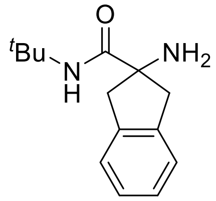

2-Amino-*N*-(*tert*-butyl)-2,3-dihydro-1*H*-indene-2-carboxamide (1m): (White solid, 42%, 290.6 mg); ^{1}H NMR (500 MHz, $CDCl_3$) δ 7.67 (br, 1H, N*H*), 7.22–7.17 (m, 4H, Ar*H*), 3.72 (d, *J* = 16.1 Hz, 2H, CCH_2), 2.68 (d, *J* = 16.0 Hz, 2H, CCH_2), 1.60 (br, 2H, NH_2), 1.39 (s, 9H, CCH_3); ^{13}C NMR (125 MHz, $CDCl_3$, 27 °C) δ 174.6, 140.8, 126.8, 125.1, 66.3, 50.5, 47.2, 28.8; IR (neat) 3364, 3291, 2963, 2909, 1971, 1651, 1514, 1479, 1450, 1389, 1360, 1302, 1273, 1263, 1246, 1101, 1069, 1024, 947, 930, 918, 876, 851, 818, 797, 735, 696, 575, 507, 486, 469, 447, 438, 419, 405 cm^{-1}; HRMS (DART) *m/z* calc'd. for $C_{14}H_{20}N_2O$ (M + H)$^+$ 233.1648, found 233.1649.

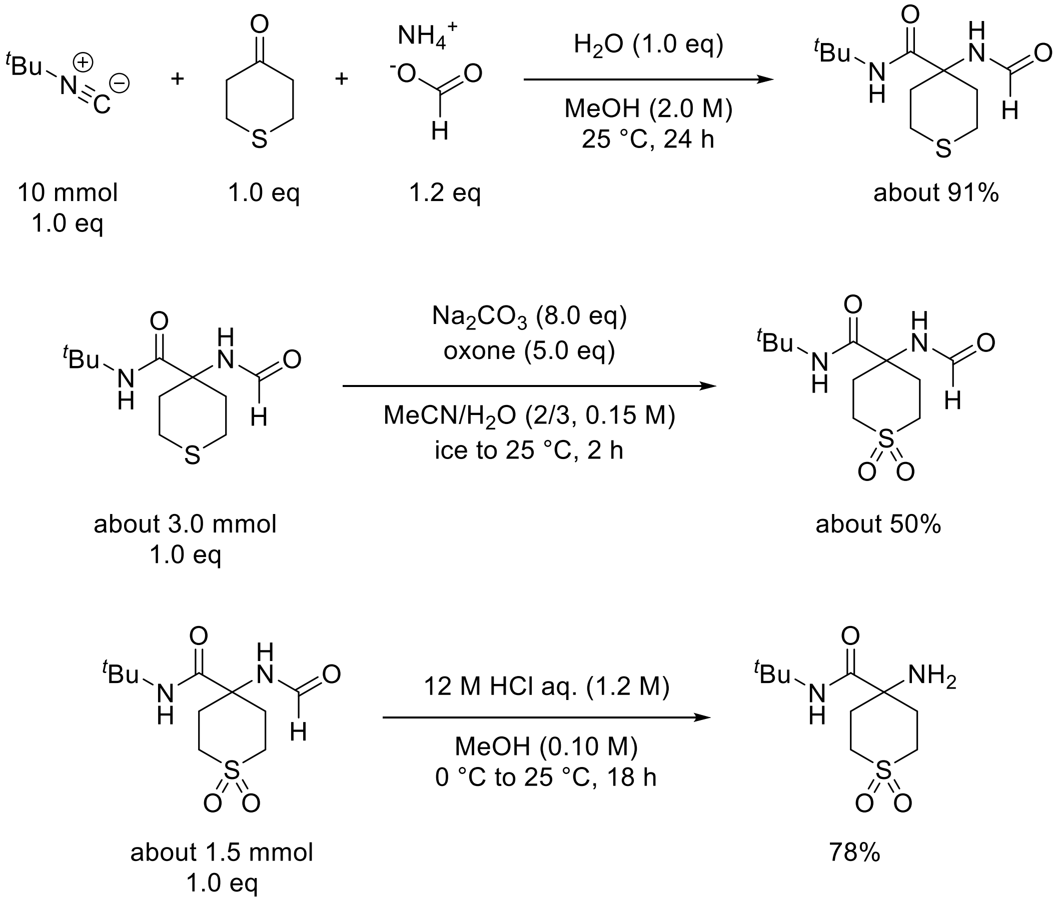

STEP 1: Isocyanide (1.0 eq.) and water (1.0 eq.) were added to a solution of ketone (1.0 eq.) and ammonium formate (1.2 eq.) in MeOH (2.0 M). The mixture was stirred overnight at 25 °C, then the volatile matter was evaporated. The residue was dissolved in DCM, washed with water, dried over Na_2SO_4 and evaporated to give the crude, which was used in the next step without further purification.

STEP 2: A mixture of *N*-(*tert*-butyl)-4-formamidotetrahydro-2*H*-thiopyran-4-carboxamide (about 3.0 mmol, 733 mg, 1.0 eq.) in acetonitrile (8.0 mL) and water (12 mL) was treated with sodium bicarbonate (2.02 g, 8.0 eq.) at ice bath. Then, Oxone (9.22 g, 5.0 eq.) was added portionwise and the resulting mixture was then warmed to 25 °C. After 2 h, the mixture was diluted with ethyl acetate and the solid was filtered off. The filter cake was washed with ethyl acetate. The layers of the filtrate were separated, and the organic layer was washed with sat. Na_2CO_3 aq.. The washed organic layer was dried over Na_2SO_4 and evaporated to give the crude (White solid, about 50%, 0.4147 g), which was used in the next step without further purification.

STEP 3: To a solution of formyl-protected amine (about 1.5 mmol, 0.4147 g, 1.0 eq.) in MeOH (0.10 M) was added conc. HCl aq. (1.2 M) with ice bath and the mixture was stirred at 25 °C for 18 h, then the crude was evaporated. The residue was dissolved in water and washed with DCM. The aqueous layer was made basic by the addition of sat. Na_2CO_3 aq. and extracted with DCM. The organic layer was dried over Na_2SO_4, filtered and

the solvent removed under reduced pressure. The residue was purified by flash column chromatography (gradient elution: *n*-Hexane/EtOAc = 4/1 to 1/1 to 1/2) to afford the desired compound.

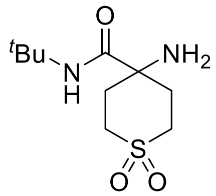

4-Amino-*N*-(*tert*-butyl)tetrahydro-2*H*-thiopyran-4-carboxamide 1,1-dioxide (1l): (White solid, 78%, 289.6 mg); ^{1}H NMR (500 MHz, $CDCl_3$) δ 7.10 (br, 1H, N*H*), 3.56–3.50 (m, 2H, SC*H*$_2$), 3.07–3.02 (m, 2H, SC*H*$_2$), 2.43–2.38 (m, 2H, CC*H*$_2$), 2.12–2.06 (m, 2H, CC*H*$_2$), 1.45 (br, 2H, N*H*$_2$), 1.35 (s, 9H, CC*H*$_3$); ^{13}C NMR (125 MHz, $CDCl_3$, 27 °C) δ 174.2, 54.1, 50.8, 47.8, 35.5, 28.6; IR (neat) 3391, 3339, 2963, 1657, 1499, 1447, 1423, 1410, 1389, 1364, 1358, 1339, 1290, 1279, 1267, 1227, 1211, 1126, 1098, 1016, 982, 935, 856, 845, 789, 745, 708, 665, 656, 554, 478, 440, 417 cm^{-1}; HRMS (DART) *m/z* calc'd. for $C_{10}H_{20}N_2O_3S$ $(M + H)^+$ 249.1267, found 249.1270.

General Procedure (1n) [8]

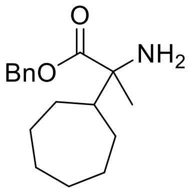

Benzyl 2-amino-2-cycloheptylpropanoate (1n): ^{1}H NMR (500 MHz, $CDCl_3$) δ 7.39–7.31 (m, 5H, Ar*H*), 5.17–5.12 (m, 2H, PhC*H*$_2$), 1.81 (tt, *J* = 10.1 Hz, 3.2 Hz, 1H, CC*H*), 1.69–1.63 (m, 3H, CHC*H*$_2$C*H*$_2$C*H*$_2$), 1.59–1.39 (m, 8H, N*H*$_2$, CHC*H*$_2$C*H*$_2$C*H*$_2$), 1.35–1.22 (m, 3H, CHC*H*$_2$C*H*$_2$C*H*$_2$), 1.26 (s, 3H, C*H*$_3$); ^{13}C NMR (125 MHz, $CDCl_3$, 27 °C) δ 178.0, 136.0, 128.6, 128.2, 128.2, 66.6, 61.9, 46.6, 29.0, 28.3, 28.2, 27.7, 27.7, 27.2, 23.2; IR (neat) 2924, 2853, 1724, 1668, 1454, 1368, 1250, 1223, 1152, 1098, 1028, 976, 910, 856, 754, 737, 696, 602, 586, 486, 469, 413, 405 cm^{-1}; HRMS (DART) *m/z* calc'd. for $C_{17}H_{25}NO_2$ $(M + H)^+$ 276.1958, found 276.1956.

Procedure (12') [9,10]

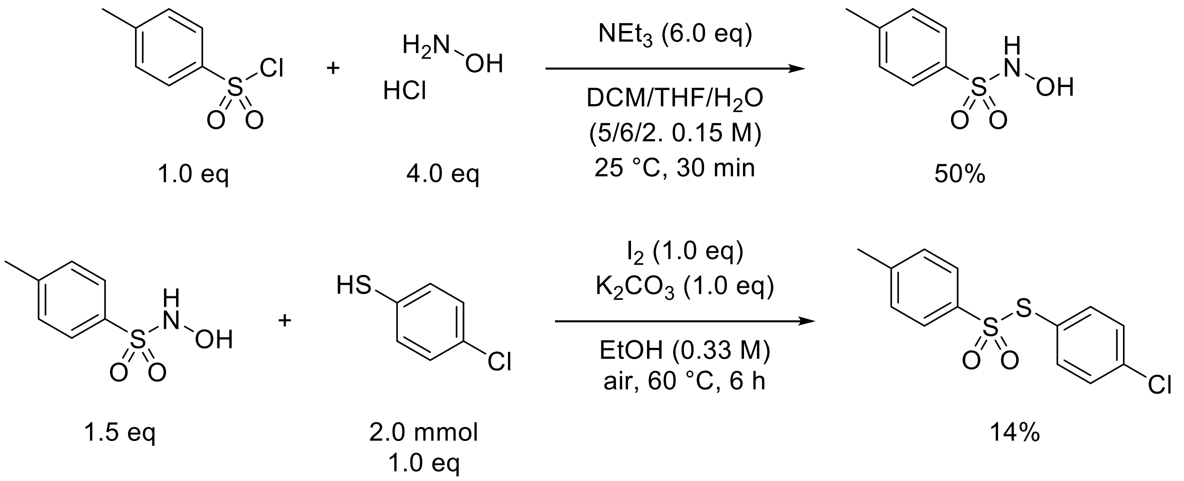

STEP 1: 4-Methylbenzenesulfonyl chloride (1.0 eq, 3.81 g, 20.0 mmol) in DCM (51 mL) was cooled by ice bath and added to a solution of hydroxylamine hydrochloride (4.0 eq, 5.56 g, 80.0 mmol) and triethylamine (6.0 eq, 16.7 ml, 120 mmol) in THF (62 mL)/H_2O (20 mL). After being stirred at 25°C for 30 min, the mixture was poured into 1 N HC1 aq. and DCM. The organic layer was separated, dried over Na_2SO_4, and concentrated under vacuum. The residue was purified by column chromatography (gradient elution: *n*-hexane/Et_2O = 2/1 to 1/1) to afford the desired compound (white solid, 50%, 1.86 g).

***N*-Hydroxy-4-methylbenzenesulfonamide**: CAS Registry Number 1593-60-8; ^1H NMR (500 MHz, CD_3OD) δ 7.79 (d, *J* = 8.3 Hz, 2H, Ar*H*), 7.39 (d, *J* = 8.0 Hz, 2H, Ar*H*), 2.44 (s, 3H, C*H₃*); ^{13}C NMR (125 MHz, CD_3OD, 27 °C) δ 145.5, 135.7, 130.4, 129.6, 21.5.

STEP 2: A mixture of 4-chlorobenzenethiol (1.0 eq, 2.0 mmol, 289 mg), N-Hydroxy-4-methylbenzenesulfonamide (1.5 eq, 3.0 mmol, 562 mg), iodine (1.0 eq, 2.0 mmol, 508 mg) and K_2CO_3 (1.0 eq, 2.0 mmol, 276 mg) in EtOH (0.33 M, 6 mL), placed in 20 ml vial under aerobic condition, was stirred at 60°C for 6 h. The reaction mixture was cooled to room temperature and quenched by the addition of a saturated sodium thiosulfate solution. The resulting mixture was extracted with DCM. The organic layer was dried over Na_2SO_4, filtered and concentrated under vacuum. The residue was purified by column chromatography (gradient elution: *n*-hexane/EtOAc = 100/1 to 50/1 to 30/1) to afford the desired compound (white solid, 14%, 84.3 mg).

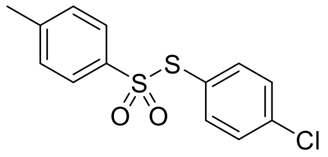

***S*-(4-Chlorophenyl) 4-methylbenzenesulfonothioate (12')**: CAS Registry Number 28823-18-9; ^1H NMR (500 MHz, $CDCl_3$) δ 7.47 (d, *J* = 8.4 Hz, 2H, Ar*H*), 7.33–7.29 (m, 4H, Ar*H*), 7.24 (d, *J* = 8.2 Hz, 2H, Ar*H*), 2.43 (s, 3H, C*H₃*); ^{13}C NMR (125 MHz, $CDCl_3$, 27 °C) δ 145.0, 140.2, 138.2, 137.7, 129.7, 129.5, 127.6, 126.6, 21.7; HRMS (DART) *m/z* calc'd. for $C_{13}H_{11}O_2S_2Cl$ $(M + NH_4)^+$ 316.0227, found 316.0226.

Procedure (16') [11]

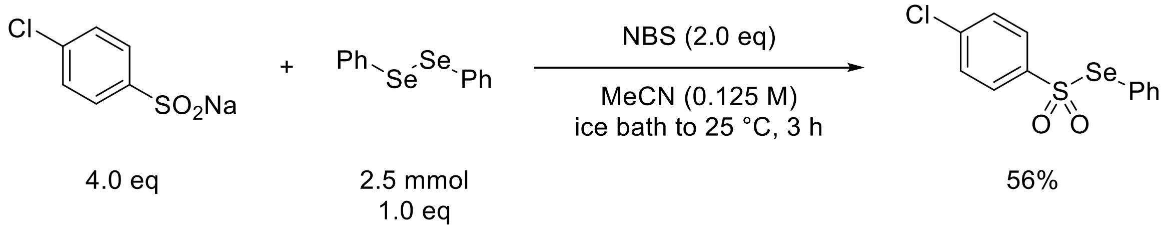

A solution of sodium 4-chlorobenzenesulfinate (1.99 g, 10 mmol), 1,2-diphenyldiselane (780 mg, 2.5 mmol), and NBS (890 mg, 5.0 mmol) in MeCN (20 mL) was stirred at 25°C for 30 min. The reaction was quenched with water, and the resulting mixture was extracted with DCM. The organic layer was dried over Na_2SO_4, filtered and concentrated under vacuum. The residue was purified by column chromatography (gradient elution: *n*-hexane/EtOAc = 100/1 to 50/1 to 30/1) to afford the desired compound (yellow solid, 56%, 932 mg).

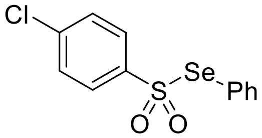

***Se*-phenyl 4-chlorobenzenesulfonoselenoate (16')**: CAS Registry Number 76200-59-4; ^{1}H NMR (500 MHz, $CDCl_3$) δ 7.52–7.48 (m, 3H, Ar*H*), 7.44–7.42 (m, 2H, Ar*H*), 7.38–7.35 (m, 4H, Ar*H*); ^{13}C NMR (125 MHz, $CDCl_3$, 27 °C) δ 143.6, 140.1, 137.2, 131.2, 129.8, 129.0, 128.4, 127.7; HRMS (DART) *m/z* calc'd. for $C_{12}H_9O_2SClSe$ (M + NH_4)$^+$ 349.9515, found 349.9513.

Procedure (15') [12]

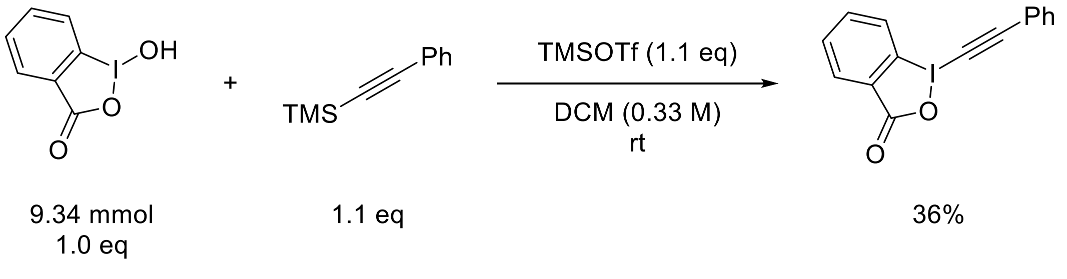

Trimethylsilyl triflate (1.87 ml, 10.3 mmol, 1.1 equiv.) was added to a suspension of hydroxybenziodoxole (BI-OH, 2.47 g, 9.34 mmol, 1.0 equiv.) in DCM (28 ml, 0.33 M) at room temperature. The resulting yellow mixture was stirred for 1 h, followed by the addition of trimethyl(phenylethynyl)silane (2.0 ml, 10.3 mmol, 1.1 equiv.). After stirring for 6 h at room temperature, saturated $NaHCO_3$ (30 mL) was added and the mixture was stirred vigorously for 30 min. After filtration, the filtrate was washed with saturated $NaHCO_3$, dried over Na_2SO_4, filtered and concentrated in vacuo. The resulting mixture was combined with the previously obtained solid and recrystallized in MeCN to afford the desired compound (white solid, 36%, 1.16 g).

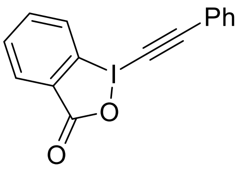

1-(2-Phenylethynyl)-1,2-benziodoxol-3(1*H*)-one (15’): CAS Registry Number 181934-31-6; ^{1}H NMR (500 MHz, $CDCl_3$) δ 8.44–8.42 (m, 1H, Ar*H*), 8.27–8.25 (m, 1H, Ar*H*), 7.80–7.75 (m, 2H, Ar*H*), 7.61–7.60 (m, 2H, Ar*H*), 7.51–7.48 (m, 1H, Ar*H*), 7.46–7.42 (m, 2H, Ar*H*); ^{13}C NMR (125 MHz, $CDCl_3$, 27 °C) δ 166.5, 134.9, 132.9, 132.5, 131.7, 131.4, 130.8, 128.8, 126.2, 120.6, 116.2, 106.7, 50.3.

Procedure (NHC catalyst) [13]

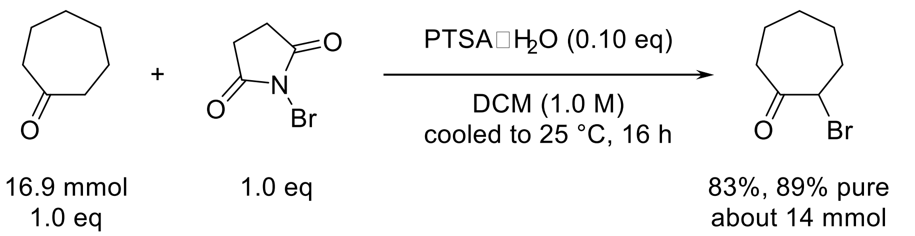

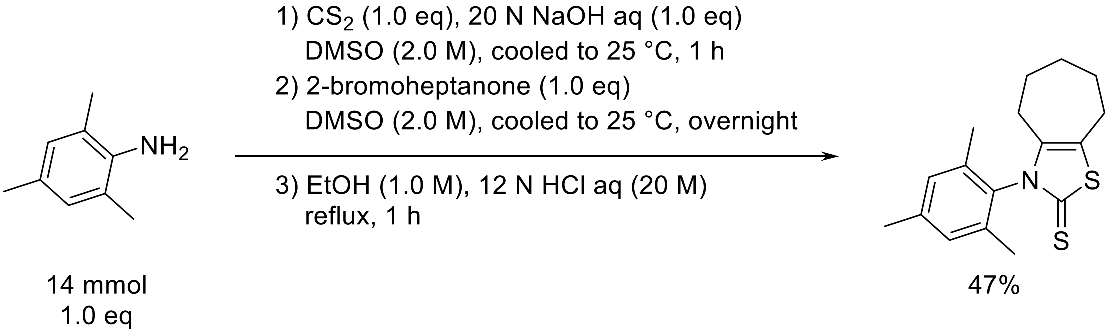

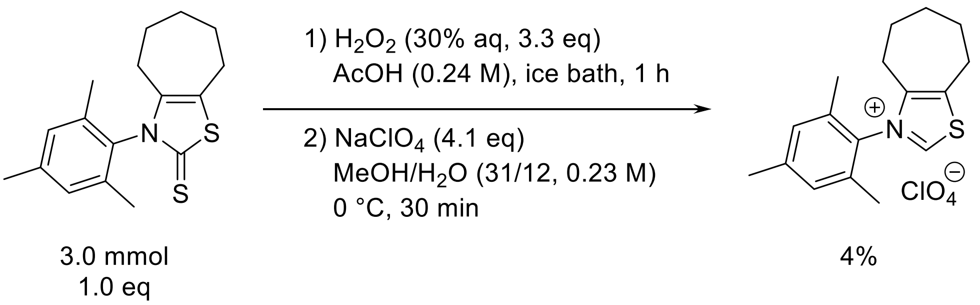

STEP 1: To a solution of N-bromosuccinimide (3.01 g, 16.9 mmol, 1.0 eq) in DCM (17 mL) was added p-toluenesulfonic acid monohydrate (322 mg, 1.69 mmol, 0.1 eq). The reaction was cooled down with an ice bath and cycloheptanone (2.0 ml, 16.9 mmol, 1.0 eq) was added. The reaction was stirred at 25 °C for 16 h and then sat. $Na_2S_2O_3$ aq. was added. The organic phase was extracted with DCM and washed with sat Na_2CO_3 aq.. The organic layer was dried over Na_2SO_4 and evaporated to give the crude, which was used in the next step without further purification (3.00 g, 83% yield, 89% pure (Mixing of cycloheptanone and DCM.)).

STEP2: To a solution of 2,4,6-trimethylaniline (2.0 mL, 14.0 mmol, 1.0 eq) in DMSO (7.0 mL) was added 20 M NaOH aq. (1.0 eq). The reaction was cooled down with an ice bath and CS_2 (0.85 mL, 14.0 mmol, 1.0 eq) was added dropwise and stirred at 25 °C for 1 h. Then, the reaction was cooled down with an ice bath. The 2-bromoheptanone (2.68 g, 14.0 mmol, 1.0 eq) was added dropwise and stirred at 25 °C for 18 h. After that, water (14 mL) was added and the mixture was further stirred for 10 min in an ice bath. The resulting slurry was decanted to remove the water and the suspension was dissolved in EtOH (14 mL). 0.70 mL of concd. HCl was added and the mixture was refluxed for 1 h. After cooling to room temperature, the mixture was placed in the fridge, where crystals precipitated. The solid was collected by suction filtration and was washed with *n*-hexane.

It was purified by flash column chromatography (gradient elution: *n*-hexane/EtOAc = 100/0 to 10/1) to afford the desired compound, which was used in the next step without further purification (yellow solid, 1.99 g, 47%).

STEP 3: The thione (910 mg, 3.0 mmol, 1.0 eq) was dissolved in glacial acetic acid (12 mL) and cooled with an ice bath. 30% H_2O_2 aq. (1.01 mL, 9.9 mmol, 3.3 eq) was added dropwise and stirred for 1 h in an ice bath. The volatiles were removed under reduced pressure. The residue was dissolved in MeOH (2.1 mL). Sodium perchlorate (1.51 g, 12.3 mmol, 4.1 eq) in a mixture (2:1) of MeOH/H_2O (10.4 mL) was added at 0 °C and the mixture was stirred at 0 °C for 30 min. The mixture was diluted with H_2O (10 ml) and extracted with DCM (3×50 mL). The organic layer was separated, dried over Na_2SO_4, and concentrated under vacuum. The residue was purified by column chromatography (DCM/MeOH = 100/1 to 30/1) to afford the desired compound (yellow solid, 4%, 46.9 mg).

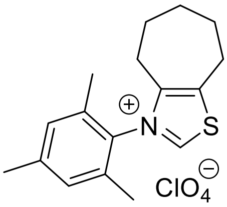

3-Mesityl-5,6,7,8-tetrahydro-4*H*-cyclohepta[*d*]thiazol-3-ium perchlorate: CAS Registry Number 1062158-63-7; ^{1}H NMR (500 MHz, $CDCl_3$) δ 9.69 (s, 1H, NC*H*), 7.07 (s, 2H, Ar*H*), 3.14–3.12 (m, 2H, CC*H*$_2$CH$_2$CH$_2$), 2.57–2.54 (m, 2H, CC*H*$_2$CH$_2$CH$_2$), 2.38 (s, 3H, C*H*$_3$), 2.01–1.94 (m, 2H, CCH$_2$C*H*$_2$C*H*$_2$), 1.97 (s, 6H, C*H*$_3$), 1.94–1.88 (m, 2H, CCH$_2$C*H*$_2$C*H*$_2$), 1.69–1.65 (m, 2H, CCH$_2$C*H*$_2$C*H*$_2$); ^{13}C NMR (125 MHz, $CDCl_3$, 27 °C) δ 155.9, 147.7, 142.3, 140.7, 134.0, 132.8, 130.2, 30.9, 28.2, 26.9, 26.7, 25.6, 21.2, 17.3.

5. General Procedure and Characterization of the Products

5-1. General procedure for catalytic oxidative homo-coupling reaction

Normal condition: To a 4 ml vial equipped with a magnetic stirrer bar, CuOAc (1.2 mg, 10 µmol, 5.0 mol%) was added in a glove box followed by the addition of cold DMF (0.20 ml, 1.0 M), 1,3-dibromo-5,5-dimethylhydantoin (68.6 mg, 0.24 mmol, 1.2 eq), amine (0.20 mmol, 1.0 eq) and 1,8-diazabicyclo[5.4.0]undec-7-ene (30 µl, 0.20 mmol, 1.0 eq) under Ar atmosphere. The reaction mixture was stirred at 25 °C for 10 min and diluted with DCM. The diluted solution was filtered through silica short column and washed with EtOAc. After evaporation of the organic solvent under reduced pressure, the resultant mixture was purified by silica gel flash chromatography to obtain the desired compound.

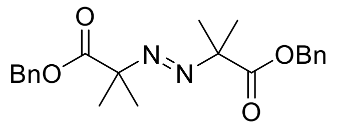

Dibenzyl 2,2'-(diazene-1,2-diyl)(*E*)-bis(2-methylpropanoate) (2a): (White solid, SM = amine, *n*-Hexane/Ethyl acetate = 10/1, 93% yield, 35.7 mg), (10 mmol scale: *n*-Hexane/Ethyl acetate = 30/1 to 10/1, 88%, 1.6796 g); ^{1}H NMR (500 MHz, $CDCl_3$) δ 7.33–7.27 (m, 10H, Ar*H*), 5.11 (s, 4H, OC*H₂*), 1.42 (s, 12H, C*H₃*); ^{13}C NMR (125 MHz, $CDCl_3$) δ 172.8, 135.9, 128.4, 128.1, 127.9, 75.2, 66.5, 22.7; IR (neat) 1722, 1497, 1458, 1383, 1364, 1281, 1254, 1213, 1146, 1018, 974, 935, 912, 854, 827, 795, 748, 694, 637, 598, 561, 496, 457, 401 cm^{-1}; HRMS (DART) *m/z* calc'd. for $C_{22}H_{26}N_2O_4$ $(M + H)^+$ 383.1965, found 383.1968.

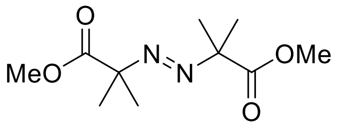

Dimethyl 2,2'-(diazene-1,2-diyl)(*E*)-bis(2-methylpropanoate) (2b): CAS Registry Number 2589-57-3 (Colorless oil, SM = amine HCl salt, *n*-Hexane/Ethyl acetate = 10/1, 84% yield, 19.3 mg); ^{1}H NMR (500 MHz, $CDCl_3$) δ 3.70 (s, 6H, OCH_3), 1.44 (s, 12H, CCH_3); ^{13}C NMR (125 MHz, $CDCl_3$) δ 173.6, 75.1, 52.1, 22.6.

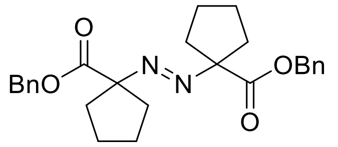

Dibenzyl 1,1'-(diazene-1,2-diyl)(*E*)-bis(cyclopentane-1-carboxylate) (2c): (Pale brown solid, SM = amine, *n*-Hexane/Ethyl acetate = 10/1, 77% yield, 33.6 mg); ^{1}H NMR (500 MHz, $CDCl_3$) δ 7.33–7.26 (m, 10H, Ar*H*), 5.09 (s, 4H, OCH_2), 2.20–2.10 (m, 8H, CCH_2CH$_2$), 1.70–1.64 (m, 4H, CCH$_2$CH_2), 1.63–1.53 (m, 4H, CCH$_2$CH_2); ^{13}C NMR (125 MHz, $CDCl_3$) δ 172.7, 135.9, 128.4, 128.1, 128.0, 86.0, 66.5, 34.1, 24.8; IR (neat) 2965, 2866, 1722, 1495,

1456, 1437, 1379, 1317, 1263, 1240, 1215, 1186, 1159, 1126, 1074, 1043, 1030, 1015, 1001, 978, 959, 910, 827, 789, 754, 743, 696, 602, 530, 492, 436 cm^{-1}; HRMS (DART) *m/z* calc'd. for $C_{26}H_{30}N_2O_4$ $(M + H)^+$ 435.2278, found 435.2276.

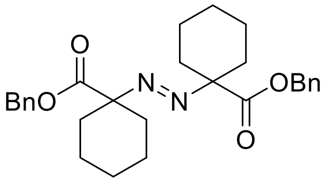

Dibenzyl 1,1'-(diazene-1,2-diyl)(*E*)-bis(cyclohexane-1-carboxylate) (2d): (Pale brown solid, SM = amine, *n*-Hexane/Ethyl acetate = 10/1, 92% yield, 42.7 mg); ^1H NMR (500 MHz, $CDCl_3$) δ 7.33–7.26 (m, 10H, Ar*H*), 5.07 (s, 4H, OCH_2), 2.03–1.99 (m, 4H, C$CH_2$$CH_2$$CH_2$), 1.91–1.86 (m, 4H, C$CH_2$$CH_2$$CH_2$), 1.61–1.57 (m, 4H, $CCH_2$$CH_2$$CH_2$), 1.52–1.32 (m, 8H, $CCH_2$$CH_2$$CH_2$); ^{13}C NMR (125 MHz, $CDCl_3$) δ 171.8, 136.0, 128.4, 128.0, 128.0, 79.4, 66.3, 31.9, 25.1, 22.2; IR (neat) 2940, 2853, 1726, 1495, 1454, 1377, 1312, 1256, 1225, 1211, 1198, 1152, 1134, 1069, 1030, 966, 951, 910, 897, 826, 787, 752, 741, 694, 615, 596, 527, 490, 478, 411 cm^{-1}; HRMS (DART) *m/z* calc'd. for $C_{28}H_{34}N_2O_4$ $(M + H)^+$ 463.2591, found 463.2593.

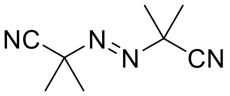

(*E*)-2,2'-(Diazene-1,2-diyl)bis(2-methylpropanenitrile) (2e): CAS Registry Number 78-67-1 (White solid, SM = amine HCl salt, *n*-Hexane/Ethyl acetate = 4/1, 96% yield, 15.7 mg); ^1H NMR (500 MHz, $CDCl_3$) δ 1.73 (s, 12H, CCH_3); ^{13}C NMR (125 MHz, $CDCl_3$) δ 119.1, 68.2, 25.2.

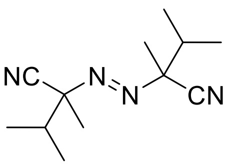

(*E*)-2,2'-(Diazene-1,2-diyl)bis(2,3-dimethylbutanenitrile) (2f): (White solid, SM = amine, *n*-Hexane /EtOAc = 4/1, 93% yield, dr = 1:1, 20.4 mg); ^1H NMR (500 MHz, $CDCl_3$) diastereomer mixture δ 2.52–2.43 (m, 2H+2H, CC*H*), 1.66 (s, 6H, CCH_3), 1.58 (s, 6H, CCH_3), 1.20 (d, *J* = 6.7 Hz, 6H, CHCH_3), 1.18 (d, *J* = 6.8 Hz, 6H, CHCH_3), 1.05 (d, *J* = 6.8 Hz, 6H, CHCH_3), 0.99 (d, *J* = 6.7 Hz, 6H, CHCH_3); ^{13}C NMR (125 MHz, C_6D_6) diastereomer mixture δ 117.1, 117.0, 77.0, 77.0, 35.4, 35.1, 21.5, 21.3, 17.4, 17.0, 16.9, 16.8; IR (neat) 2986, 2918, 2911, 1464, 1391, 1375, 1319, 1211, 1130, 1105, 1049, 926, 864, 667, 635, 613, 600, 501, 480 cm^{-1}; HRMS (DART) *m/z* calc'd. for $C_{12}H_{20}N_4$ $(M + NH_4)^+$ 238.2026, found 238.2022.

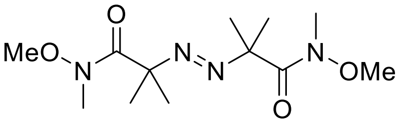

(*E*)-2,2'-(Diazene-1,2-diyl)bis(*N*-methoxy-*N*,2-dimethylpropanamide) (2g): (White solid, SM = amine, *n*-Hexane/Ethyl acetate = 30/1 to 10/1 to 4/1, 82% yield, 23.7 mg); ^{1}H NMR (500 MHz, $CDCl_3$) δ 3.55 (s, 6H, OC*H$_3$*), 3.20 (s, 6H, NC*H$_3$*), 1.42 (s, 12H, CC*H$_3$*); ^{13}C NMR (125 MHz, $CDCl_3$) δ 174.4, 75.3, 60.8, 33.7, 23.5; IR (neat) 2941, 2170, 1655, 1647, 1458, 1400, 1369, 1358, 1196, 1175, 1153, 1121, 1084, 1011, 991, 959, 827, 731, 685, 613, 579, 500, 436, 419, 405 cm^{-1}; HRMS (DART) *m/z* calc'd. for $C_{12}H_{24}N_4O_4$ (M + H)$^+$ 289.1870, found 289.1873.

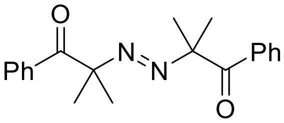

(*E*)-2,2'-(Diazene-1,2-diyl)bis(2-methyl-1-phenylpropan-1-one) (2h): (White solid, SM = amine TFA salt, *n*-Hexane/Et_2O = 40/1 to 20/1, 45% yield, 14.4 mg); ^{1}H NMR (500 MHz, $CDCl_3$) δ 7.63 (dd, 4H, *J* = 1.0, 8.5 Hz, Ar*H*), 7.40 (t, 2H, *J* = 7.2 Hz, Ar*H*), 7.28 (dd, 4H, *J* = 8.0, 8.0 Hz, Ar*H*), 1.54 (s, 12H, C*H$_3$*); ^{13}C NMR (125 MHz, $CDCl_3$) δ 200.6, 134.7, 132.2, 130.2, 128.0, 80.6, 24.3; IR (neat) 2992, 2972, 2928, 1676, 1634, 1597, 1580, 1472, 1456, 1447, 1387, 1371, 1358, 1317, 1267, 1165, 1105, 1076, 1013, 1003, 984, 939, 918, 907, 835, 824, 789, 706, 691, 608, 588, 579, 515, 505, 459, 419, 412 cm^{-1}; HRMS (DART) *m/z* calc'd. for $C_{20}H_{22}N_2O_2$ (M + H)$^+$ 323.1754, found 323.1764.

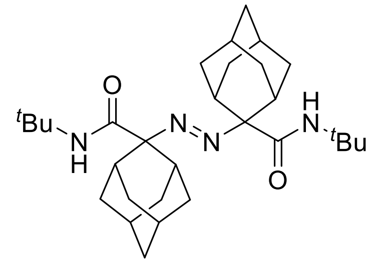

2,2'-((*E*)-Diazene-1,2-diyl)bis(*N*-(tert-butyl)adamantane-2-carboxamide) (2i): (Changed condition : DMF (0.50 M), 3 h., pale yellow solid, SM = amine, *n*-Hexane/DCM/Et_2O = 40/1//1 to 10/1/1, 60% yield, 30.0 mg); ^{1}H NMR (500 MHz, $CDCl_3$) δ 5.54 (br, 2H, N*H*), 2.73 (s, 4H, Ad*H*), 2.03 (d, *J* = 12.8 Hz, 4H, Ad*H*), 1.88 (s, 2H, Ad*H*), 1.79 (d, *J* = 12.2 Hz, 4H, Ad*H*), 1.73–1.69 (m, 10H, Ad*H*), 1.58–1.55 (m, 4H, Ad*H*), 1.28 (s, 18H, C*H$_3$*); ^{13}C NMR (125 MHz, $CDCl_3$) δ 167.7, 82.5, 51.1, 37.4, 34.9, 33.9, 32.3, 28.5, 27.0, 26.8; IR (neat) 3414, 2972, 2932, 2905, 2853, 1668, 1506, 1476, 1468, 1449, 1391, 1364, 1356, 1350, 1267, 1242, 1219, 1099, 1069, 1061, 999, 883, 831, 741, 519, 503, 478, 451, 438, 419 cm^{-1}; HRMS (DART) *m/z* calc'd. for $C_{30}H_{48}N_4O_2$ (M + H)$^+$ 497.3850, found 497.3852.

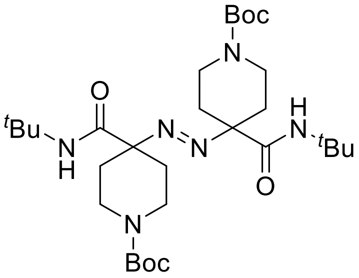

Di-*tert*-butyl 4,4'-(diazene-1,2-diyl)(*E*)-bis(4-(*tert*-butylcarbamoyl)piperidine-1-carboxylate) (2j): (Pale yellow solid, SM = amine, *n*-Hexane/EtOAc = 20/1 to 10/1, 96% yield, 57.2 mg); ^{1}H NMR (500 MHz, $CDCl_3$) δ 5.93 (br, 2H, N*H*), 3.79 (br, 4H, NC*H₂*), 3.03 (br, 4H, NC*H₂*), 2.21–2.07 (m, 8H, CC*H₂*), 1.45 (s, 18H, C*H₃*), 1.33 (s, 18H, C*H₃*); ^{13}C NMR (125 MHz, C_6D_6) δ 169.5, 154.3, 79.0, 76.8, 51.0, 31.1, 28.3, 28.1; IR (neat) 2972, 1732, 1670, 1653, 1522, 1452, 1420, 1391, 1364, 1279, 1244, 1225, 1171, 1138, 1080, 1080, 1063, 1063, 1045, 957, 957, 862, 762, 530, 473 cm^{-1}; HRMS (DART) *m/z* calc'd. for $C_{30}H_{54}N_6O_6$ $(M + H)^+$ 595.4178, found 595.4190.

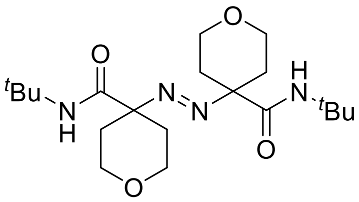

2,2'-((*E*)-Diazene-1,2-diyl)bis(*N*-(tert-butyl)adamantane-2-carboxamide) (2k): (Pale yellow solid, SM = amine, *n*-Hexane/EtOAc = 20/1 to 10/1 to 4/1 to 2/1, 87% yield, 34.3 mg); ^{1}H NMR (500 MHz, $CDCl_3$) δ 5.92 (br, 2H, N*H*), 3.88 (dt, *J* = 11.8 Hz, 4.8 Hz, 4H, OC*H₂*), 3.54–3.50 (m, 4H, OC*H₂*), 2.33–2.27 (m, 4H, CC*H₂*), 2.12–2.07 (m, 4H, CC*H₂*), 1.34 (s, 18H, C*H₃*); ^{13}C NMR (125 MHz, $CDCl_3$) δ 169.8, 75.8, 64.0, 51.6, 31.7, 28.7; IR (neat) 3366, 3294, 2972, 2955, 2868, 1717, 1697, 1659, 1543, 1526, 1474, 1454, 1418, 1385, 1362, 1287, 1242, 1163, 1142, 1107, 1098, 1063, 1034, 1009, 959, 932, 910, 843, 833, 642, 588, 557, 484, 467, 407 cm^{-1}; HRMS (DART) *m/z* calc'd. for $C_{20}H_{36}N_4O_4$ $(M + H)^+$ 397.2809, found 397.2802.

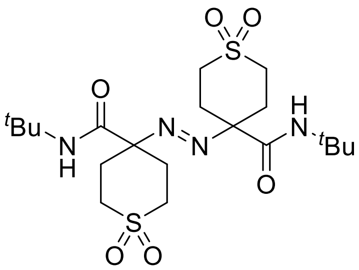

(*E*)-4,4'-(Diazene-1,2-diyl)bis(*N*-(*tert*-butyl)tetrahydro-2*H*-thiopyran-4-carboxamide 1,1-dioxide) (2l): (White solid, SM = amine, DCM/MeOH = 100/1 to 50/1 to 30/1 (As DMF was mixed in the column chromatography, it was filtered by suction and washed with *n*-Hexane /Et_2O = 1/1 (200 ml) to give the white solid), 89% yield, 43.9 mg); ^1H NMR (500 MHz, DMSO) δ 7.02 (br, 2H, N*H*), 3.17–3.14 (m, 4H, SC*H₂*), 3.03–2.97 (m, 4H, SC*H₂*), 2.67–2.64 (m, 4H, CC*H₂*), 2.60–2.54 (m, 4H, CC*H₂*), 1.25 (s, 18H, C*H₃*); ^{13}C NMR (125 MHz, DMSO) δ 168.4, 75.8, 51.7, 47.3, 30.0, 28.7; IR (neat) 3374, 2976, 2160, 1665, 1516, 1460, 1395, 1368, 1325, 1300, 1288, 1250, 1234, 1213, 1188, 1126, 1059, 920, 901, 854, 835, 704, 696, 664, 592, 550, 473, 455, 440, 430, 407 cm^{-1}; HRMS (DART) *m/z* calc'd. for $C_{20}H_{36}N_4O_6S_2$ $(M + H)^+$ 493.2149, found 493.2141.

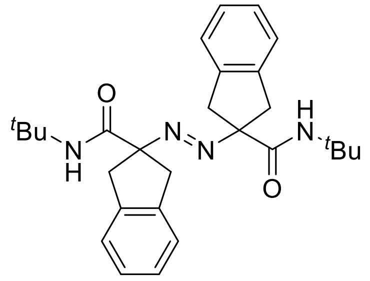

(*E*)-2,2'-(Diazene-1,2-diyl)bis(*N*-(*tert*-butyl)-2,3-dihydro-1*H*-indene-2-carboxamide) (2m): (White solid, SM = amine, DCM/EtOAc = 10/1 (As byproduct was mixed in the column chromatography, it was filtered by suction and washed with *n*-Hexane /EtOAc = 1/1 (20 ml) to give the white solid), 91% yield, 41.7 mg); ^1H NMR (500 MHz, $CDCl_3$) δ 7.15–7.11 (m, 8H, Ar*H*), 6.15 (br, 2H, N*H*), 3.60 (d, *J* = 16.5 Hz, 4H, CC*H₂*), 3.04 (d, *J* = 16.5 Hz, 4H, CC*H₂*), 1.11 (s, 18H, C*H₃*); ^{13}C NMR (125 MHz, $CDCl_3$) δ 170.8, 139.7, 127.1, 124.1, 84.2, 51.0, 40.6, 28.6; IR (neat) 3377, 2363, 1672, 1508, 1487, 1476, 1454, 1364, 1269, 1219, 870, 799, 799, 748, 729, 596, 583, 540, 474, 474 cm^{-1}; HRMS (DART) *m/z* calc'd. for $C_{28}H_{36}N_4O_2$ $(M + H)^+$ 461.2911, found 461.2903.

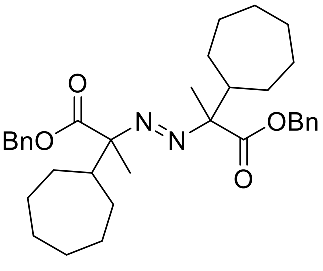

Dibenzyl 2,2'-(diazene-1,2-diyl)(*E*)-bis(2-cycloheptylpropanoate) (2n): (White solid, SM = amine, *n*-Hexane /EtOAc = 20/1, 54% yield, dr = 4:5, 29.3 mg); ^1H NMR (500 MHz, $CDCl_3$) diastereomer mixture δ 7.34–7.27 (m, 10H+12.5H, Ar*H*), 5.13–4.95 (m, 4H+5H, OC*H₂*), 2.43–2.36 (m, 4H, CC*H*C*H₂*C*H₂*C*H₂*), 1.88–1.82 (m, 5H, CC*H*C*H₂*C*H₂*C*H₂*), 1.71–0.82 (m, 63H, C*H₃*, CC*H*C*H₂*C*H₂*C*H₂*),; ^{13}C NMR (125 MHz, $CDCl_3$) diastereomer mixture δ 172.7, 172.6, 135.9, 135.9, 128.4, 128.4, 128.1, 128.1, 128.0, 128.0, 83.2, 83.0, 66.4, 66.3, 44.8, 44.5, 29.7, 29.5, 28.9, 28.9, 28.0, 27.9, 27.7, 27.7, 27.6, 27.6, 27.4, 27.3, 16.3, 16.3; IR (neat) 2918, 2851, 2361, 2342, 2332,

2168, 1724, 1497, 1449, 1373, 1279, 1256, 1240, 1211, 1173, 1142, 1111, 1084, 1030, 968, 955, 939, 916, 895, 827, 787, 748, 735, 696, 602, 573, 494, 484, 476, 442, 424 cm^{-1}; HRMS (DART) *m/z* calc'd. for $C_{34}H_{46}N_2O_4$ (M + H)$^+$ 547.3530, found 547.3523.

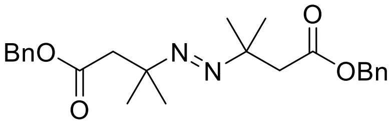

Dibenzyl 3,3'-(diazene-1,2-diyl)(*E*)-bis(3-methylbutanoate) (2o): (Colorless oil, SM = amine, *n*-Hexane/Et_2O = 30/1 to 10/1, 66% yield, 27.1 mg); ^{1}H NMR (500 MHz, $CDCl_3$) δ 7.36–7.28 (m, 10H, Ar*H*), 5.06 (s, 4H, OC*H2*), 2.57 (s, 4H, CC*H2*), 1.23 (s, 12H, CC*H3*); ^{13}C NMR (125 MHz, $CDCl_3$) δ 171.3, 136.0, 128.5, 128.3, 128.2, 67.8, 66.0, 43.8, 24.9; IR (neat) 2972, 2932. 2355, 1732, 1499, 1454, 1375, 1364, 1344, 1314, 1287, 1211, 1194, 1123, 1003, 914, 737, 696, 581, 538, 492, 473 cm^{-1}; HRMS (DART) *m/z* calc'd. for $C_{24}H_{30}N_2O_4$ (M + H)$^+$ 411.2278, found 411.2282.

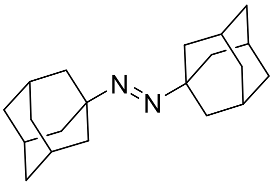

(*E*)-1,2-Di(adamantan-1-yl)diazene (2p): CAS Registry Number 24325-56-2 (White solid, SM = amine, *n*-Hexane/EtOAc = 100/0 to 40/1, 52% yield, 15.6 mg); ^{1}H NMR (500 MHz, $CDCl_3$) δ 2.14 (s, 6H, Ad*H*), 1.75–1.66 (m, 24H, Ad*H*); ^{13}C NMR (125 MHz, $CDCl_3$) δ 66.5, 40.4, 36.7, 29.4; IR (neat) 2899, 2845, 1450, 1364, 1342, 1306, 1184, 1101, 1088, 972, 814, 505 cm^{-1}; HRMS (DART) *m/z* calc'd. for $C_{20}H_{30}N_2$ (M + H)$^+$ 299.2482, found 299.2473.

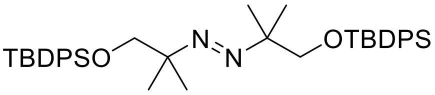

(*E*)-2,2,6,6,9,9,13,13-Octamethyl-3,3,12,12-tetraphenyl-4,11-dioxa-7,8-diaza-3,12-disilatetradec-7-ene (2q): (White solid, SM = amine, 1st: *n*-Hexane/Et_2O = 50/1 to 20/1, 2nd: *n*-Hexane/DCM = 40/1 to 20/1 to 10/1 to 4/1, 78% yield, 51.0 mg); ^{1}H NMR (500 MHz, $CDCl_3$) δ 7.66 (dd, *J* = 7.9 Hz, 1.3 Hz, 8H, Ar*H*), 7.40 (tt, *J* = 7.3 Hz, 1.8 Hz, 4H, Ar*H*), 7.35 (t, *J* = 7.4 Hz, 8H, Ar*H*), 3.84 (s, 4H, OC*H2*), 1.15 (s, 12H, NCC*H3*), 0.99 (s, 18H, SiCC*H3*); ^{13}C NMR (125 MHz, $CDCl_3$) δ 135.7, 133.7, 129.5, 127.6, 70.7, 70.3, 26.8, 21.7, 19.3; IR (neat) 2955, 2924, 2363, 2207, 1470, 1427, 1387, 1360, 1194, 1111, 1094, 1007, 826, 818, 741, 702, 689, 619, 505, 490, 469, 461, 403 cm^{-1}; HRMS (DART) *m/z* calc'd. for $C_{40}H_{54}N_2O_2Si_2$ (M + H)$^+$ 651.3797, found 651.3805.

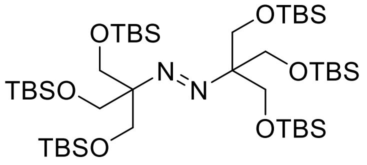

(*E*)-6,6,9,9-Tetrakis(((tert-butyldimethylsilyl)oxy)methyl)-2,2,3,3,12,12,13,13-octamethyl-4,11-dioxa-7,8-diaza-3,12-disilatetradec-7-ene (2r): (Changed condition : DMF (0.50 M), 3 h., pale yellow solid, SM = amine, *n*-Hexane/Et_2O = 4/1, 91% yield, 84.4 mg); ^1H NMR (500 MHz, $CDCl_3$) δ 3.80 (s, 12H, OC*H₂*), 0.87 (s, 54H, CC*H₃*), 0.01 (s, 36H, SiC*H₃*); ^{13}C NMR (125 MHz, $CDCl_3$) δ 77.7, 62.3, 26.0, 18.3, −5.5; IR (neat) 2951, 2926, 2884, 2855, 1472, 1462, 1360, 1248, 1078, 1005, 939, 829, 812, 772, 667, 451 cm^{-1}; HRMS (DART) *m/z* calc'd. for $C_{44}H_{102}N_2O_6Si_6$ $(M + H)^+$ 923.6426, found 923.6421.

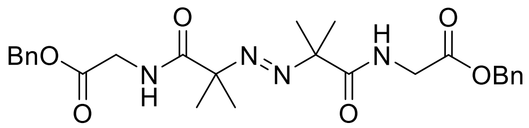

Dibenzyl 2,2'-((2,2'-(diazene-1,2-diyl)bis(2-methylpropanoyl))bis(azanediyl))(*E*)-diacetate (2s): (Colorless oil, SM = amine, *n*-Hexane/EtOAc = 30/1 to 10/1 to 5/1 to 3/1, 83% yield, 41.4 mg); ^1H NMR (500 MHz, $CDCl_3$) δ 7.38–7.33 (m, 12H, N*H*, Ar*H*), 5.18 (s, 4H, OCH_2), 4.17 (d, *J* = 5.0 Hz, 4H, NCH_2), 1.37 (s, 12H, CCH_3); ^{13}C NMR (125 MHz, $CDCl_3$) δ 174.0, 169.7, 135.1, 128.7, 128.6, 128.4, 74.9, 67.3, 41.5, 23.0; IR (neat) 3387, 3360, 2978, 2936, 1744, 1667, 1508, 1456, 1385, 1356, 1258, 1173, 1082, 1022, 955, 914, 870, 826, 737, 696, 579, 557, 523, 494, 484, 465, 457, 444, 424, 417, 405 cm^{-1}; HRMS (DART) *m/z* calc'd. for $C_{26}H_{32}N_4O_6$ $(M + H)^+$ 497.2395, found 497.2395.

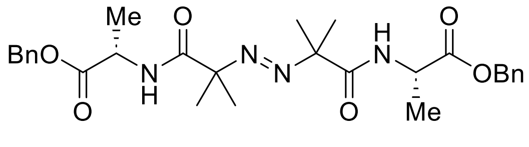

Dibenzyl 2,2'-((2,2'-((*E*)-diazene-1,2-diyl)bis(2-methylpropanoyl))bis(azanediyl))(2*S*,2'*S*)-dipropionate (2t): (Colorless gel, SM = amine, *n*-Hexane/DCM/Et_2O = 5/5/1 to 3/3/1 to 2/2/1, 80% yield, 41.9 mg); ^1H NMR (500 MHz, $CDCl_3$) δ 7.46 (d, *J* = 7.3 Hz, 2H, N*H*), 7.38–7.32 (m, 10H, Ar*H*), 5.18 (dd, *J* = 24.4 Hz, 12.2 Hz, 4H, OCH_2), 4.70 (quintet, *J* = 7.2 Hz, 2H, NHC*H*), 1.47 (d, *J* = 7.1 Hz, 6H, CHCH_3), 1.34 (s, 6H, CCH_3), 1.33 (s, 6H, CCH_3); ^{13}C NMR (125 MHz, $CDCl_3$) δ 173.4, 172.7, 135.3, 128.7, 128.5, 128.2, 74.7, 67.2, 48.2, 23.0, 22.8, 18.4; IR (neat) 3379, 2980, 2940, 2365, 1738, 1672, 1501, 1454, 1385, 1339, 1306, 1258, 1184, 1144, 1051, 1030, 1003, 961, 908, 748, 696, 604, 592, 577, 525, 471, 461, 440, 420, 413, 401 cm^{-1}; HRMS (DART) *m/z* calc'd. for $C_{28}H_{36}N_4O_6$ $(M + H)^+$ 525.2708, found 525.2701.

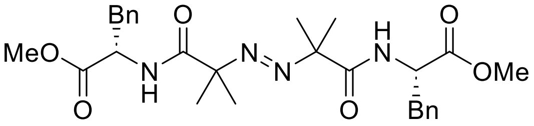

Dimethyl 2,2'-((2,2'-((*E*)-diazene-1,2-diyl)bis(2-methylpropanoyl))bis(azanediyl))(2*S*,2'*S*)-bis(3-phenylpropanoate) (2u): (Colorless gel, SM = amine, *n*-Hexane/DCM/Et_2O = 10/10/1 to 5/5/1 to 3/3/1 to 2/2/1, 64% yield, 33.8 mg); ^{1}H NMR (500 MHz, $CDCl_3$) δ 7.31 (d, *J* = 7.5 Hz, 2H, N*H*), 7.25–7.20 (m, 6H, Ar*H*), 7.07–7.06 (m, 4H, Ar*H*), 4.94 (q, *J* = 6.4 Hz, 2H, C*H*), 3.74 (s, 6H, OC*H3*), 3.24–3.12 (m, 4H, CHC*H2*), 1.20 (s, 6H, CC*H3*), 1.16 (s, 6H, CC*H3*); ^{13}C NMR (125 MHz, $CDCl_3$) δ 173.5, 171.8, 135.7, 129.2, 128.6, 127.2, 74.6, 53.0, 52.4, 37.7, 22.8, 22.7; IR (neat) 3387, 2359, 2340, 1742, 1674, 1497, 1456, 1437, 1373, 1356, 1198, 1177, 1115, 1078, 1018, 895, 866, 814, 745, 700, 669, 598, 556, 492, 471, 455, 447, 438, 430, 422, 405 cm^{-1}; HRMS (DART) *m/z* calc'd. for $C_{28}H_{36}N_4O_6$ $(M + H)^+$ 525.2708, found 525.2714.

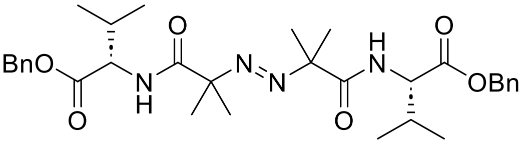

Dibenzyl 2,2'-((2,2'-((*E*)-diazene-1,2-diyl)bis(2-methylpropanoyl))bis(azanediyl))(2*S*,2'*S*)-bis(3-methylbutanoate) (2v): (White solid, SM = amine, *n*-Hexane/Et_2O = 100/1 to 20/1 to 5/1 to 1/1, 92% yield, 53.4 mg); ^{1}H NMR (500 MHz, $CDCl_3$) δ 7.45 (d, *J* = 8.7 Hz, 2H, N*H*), 7.38–7.31 (m, 10H, Ar*H*), 5.17 (dd, *J* = 31.6 Hz, 12.2 Hz, 4H, OC*H2*), 4.69 (q, *J* = 4.4 Hz, 2H, NHC*H*), 2.31–2.22 (m, 2H, CH_3C*H*), 1.35 (s, 6H, CC*H3*), 1.33 (s, 6H, CC*H3*), 0.93 (d, *J* = 6.9 Hz, 6H, CHC*H3*), 0.86 (d, *J* = 6.9 Hz, 6H, CHC*H3*); ^{13}C NMR (125 MHz, $CDCl_3$) δ 173.8, 171.5, 135.3, 128.6, 128.5, 128.4, 74.9, 67.1, 56.9, 31.3, 22.9, 22.8, 19.1, 17.5; IR (neat) 3374, 2980, 2965, 2930, 2874, 1726, 1670, 1506, 1456, 1391, 1373, 1358, 1296, 1250, 1200, 1136, 1103, 1082, 1030, 953, 939, 907, 897, 847, 808, 768, 750, 698, 669, 637, 575, 542, 523, 501, 480, 419, 407 cm^{-1}; HRMS (DART) *m/z* calc'd. for $C_{32}H_{44}N_4O_6$ $(M + H)^+$ 581.3334, found 581.3327.

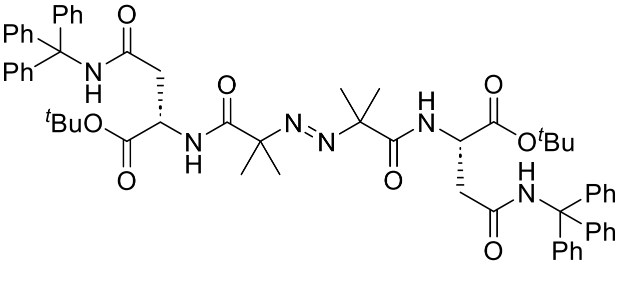

Di-*tert*-butyl 2,2'-((2,2'-((*E*)-diazene-1,2-diyl)bis(2-methylpropanoyl))bis(azanediyl))(2*S*,2'*S*)-bis(4-oxo-4-(tritylamino)butanoate) (2w): (White solid, SM = amine, *n*-Hexane/EtOAc = 10/1 to 5/1 to 3/1, 86% yield, 88.0

mg); ^{1}H NMR (500 MHz, $CDCl_3$) δ 7.96 (d, *J* = 7.8 Hz, 2H, N*H*), 7.25–7.19 (m, 18H, Ar*H*), 7.17–7.13 (m, 12H, Ar*H*), 6.69 (br, 2H, N*H*), 4.71 (quintet, *J* = 4.2 Hz, 2H, C*H*), 3.02 (dd, *J* = 16.0 Hz, 4.7 Hz, 2H, C*H₂*), 2.81 (dd, *J* = 16.0 Hz, 4.3 Hz, 2H, CH_2), 1.38 (s, 18H, CH_3), 1.22 (s, 6H, CH_3), 1.19 (s, 6H, CH_3); ^{13}C NMR (125 MHz, $CDCl_3$) δ 174.1, 169.9, 168.9, 144.5, 128.7, 127.9, 127.0, 82.2, 74.7, 70.7, 49.6, 38.3, 27.9, 22.9, 22.8; IR (neat) 1734, 1663, 1489, 1447, 1368, 1246, 1229, 1153, 1036, 905, 847, 750, 729, 637, 623, 617, 525, 498, 490, 480 cm^{-1}; HRMS (DART) *m/z* calc'd. for $C_{62}H_{70}N_6O_8$ $(M + 2H)^{2+}$ 514.2700, found 514.2696.

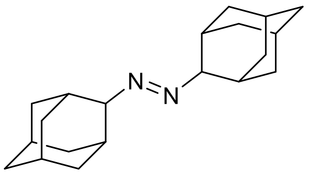

(*E*)-1,2-di((1*R*,3*R*,5*R*,7*R*)-adamantan-2-yl)diazene (2x): (Pale yellow solid, SM = amine HCl salt, *n*-Hexane only to *n*-Hexane/Ethyl acetate = 100/1, 44% yield, 13.2 mg); ^{1}H NMR (500 MHz, $CDCl_3$) δ 3.47 (s, 2H, NC*H*), 2.22 (d, *J* = 12.3 Hz, 4H, Ad*H*), 2.05 (s, 4H,Ad*H*), 1.96–1.81 (m, 16H, Ad*H*), 1.63 (d, *J* = 11.8 Hz, 4H, Ad*H*); ^{13}C NMR (125 MHz, $CDCl_3$) δ 79.7, 37.8, 37.4, 33.0, 32.2, 28.1, 27.5; IR (neat) 2897, 2864, 2847, 1541, 1464, 1447, 1354, 1342, 1260, 1099, 1063, 1034, 1024, 980, 961, 820, 797 cm^{-1}; HRMS (DART) *m/z* calc'd. for $C_{20}H_{30}N_2$ $(M + H)^{+}$ 299.2482, found 299.2492.

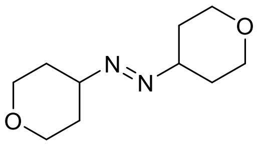

(*E*)-1,2-bis(tetrahydro-2H-pyran-4-yl)diazene (2y): (White solid, SM = amine, *n*-Hexane/Ethyl acetate = 10/1 to 4/1, 42% yield, 8.25 mg); ^{1}H NMR (500 MHz, $CDCl_3$) δ 4.07 (td, *J* = 7.6 Hz, 4.1 Hz, 4H, OCH_2), 3.60 (tt, *J* = 10.5 Hz, 3.0 Hz, 2H, NCH_2), 3.52 (dt, *J* = 16.1 Hz, 5.8 Hz, 4H, OCH_2), 3.54–3.49 (m, 4H, OCH_2CH_2), 1.74–1.71 (m, 4H, OCH_2CH_2) ; ^{13}C NMR (125 MHz, $CDCl_3$) δ72.3, 66.1, 30.5; IR (neat) 3734, 2953, 2928, 2918, 2886, 2853, 2361, 1717, 1460, 1445, 1379, 1350, 1312, 1248, 1236, 1165, 1134, 1080, 1009, 980, 974, 864, 818, 619, 505, 447, 409 cm^{-1}; HRMS (DART) *m/z* calc'd. for $C_{10}H_{18}N_2O_2$ $(M + H)^{+}$ 199.1441, found 199.1447.

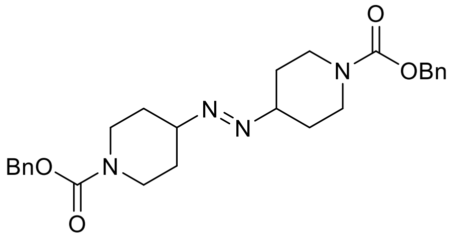

dibenzyl 4,4'-(diazene-1,2-diyl)(*E*)-bis(piperidine-1-carboxylate) (2z): (White solid, SM = amine, *n*-Hexane/Ethyl acetate = 10/1 to 4/1, 31% yield, 73.0 mg); ^{1}H NMR (500 MHz, $CDCl_3$) δ 7.37–7.32 (m, 10H, Ar*H*), 5.15 (s, 4H, ArC*H₂*), 4.17 (s, 4H, NC*H₂*), 3.58–3.52 (m, 2H, NC*H*), 3.05 (s, 4H, NC*H₂*), 1.83–1.78 (m, 8H, NCHC*H₂*); ^{13}C NMR (125 MHz, $CDCl_3$) δ155.3, 136.8, 128.5, 128.0, 127.9, 73.0, 67.2, 42.2, 29.6; IR (neat) 2947, 2928, 2876, 2860, 1703, 1680, 1466, 1456, 1427, 1427, 1383, 1362, 1362, 1290, 1290, 1271, 1223, 1155, 1130, 1094, 1028, 1015, 1001, 961, 943, 910, 770, 750, 729, 694, 598, 588, 554, 459 cm^{-1}; HRMS (DART) *m/z* calc'd. for $C_{26}H_{32}N_4O_4$ (M + H)$^+$ 465.2496, found 465.2486.

Imine was observed as a major byproduct under the reaction conditions. An increased amount of copper salts suppressed the formation of imine.

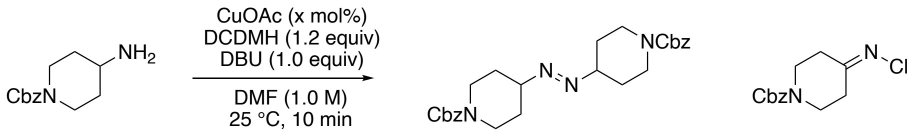

0.2 mmol	x = 5	0.028 mmol	0.114 mmol
	50	0.043 mmol	0.086 mmol
	100	0.057 mmol	0.042 mmol

5-2. General procedure for catalytic oxidative cross-coupling reaction

Condition: To a 4 ml vial equipped with a magnetic stirrer bar, CuOAc (2.5 mg, 20 µmol, 10 mol%) was added in a glove box followed by the addition of cold DMF (0.40 ml, 0.50 M), 1,3-dibromo-5,5-dimethylhydantoin (274 mg, 0.96 mmol, 4.8 eq), amine **1** (0.20 mmol, 1.0 eq), amine **1'** (0.60 mmol, 3.0 eq) and 1,8-diazabicyclo[5.4.0]undec-7-ene (120 µl, 0.80 mmol, 4.0 eq) under Ar atmosphere. The reaction mixture was stirred at 25 °C for 10 min and diluted with DCM. The diluted solution was filtered through silica short column and washed with EtOAc. After evaporation of the organic solvent under reduced pressure, the resultant mixture was purified by silica gel flash chromatography to obtain the desired compound.

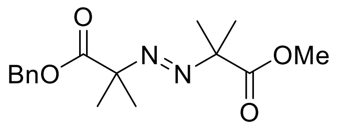

Benzyl (*E*)-2-((1-methoxy-2-methyl-1-oxopropan-2-yl)diazenyl)-2-methylpropanoate (3ab): (Colorless oil, *n*-Hexane/Et_2O = 100/1 to 50/1 to 30/1 to 20/1, 71% yield, 43.5 mg); ^{1}H NMR (500 MHz, $CDCl_3$) δ 7.36–7.29 (m, 5H, Ar*H*), 5.15 (s, 2H, OC*H₂*), 3.64 (s, 3H, OC*H₃*), 1.47 (s, 6H, CC*H₃*), 1.40 (s, 6H, CC*H₃*); ^{13}C NMR (125 MHz, $CDCl_3$) δ 173.5, 172.9, 135.9, 128.4, 128.1, 127.8, 75.2, 75.1, 66.5, 52.0, 22.7, 22.7; IR (neat) 2988, 2938, 2363, 1736, 1464, 1456, 1435, 1381, 1364, 1281, 1142, 1082, 1030, 1011, 989, 949, 912, 854, 820, 793, 750, 737, 696, 631, 619, 600, 586, 559, 494, 478, 457, 449, 438, 424, 409 cm^{-1}; HRMS (DART) *m/z* calc'd. for $C_{16}H_{22}N_2O_4$ $(M + H)^+$ 307.1652, found 307.1665.

The ^{1}H-NMR analysis of the crude reaction mixture confirmed that the remaining substrates were converted into corresponding homo-diazenes.

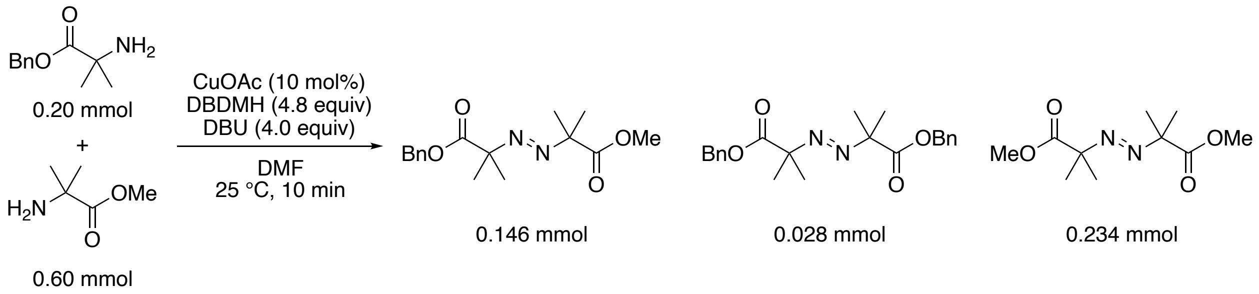

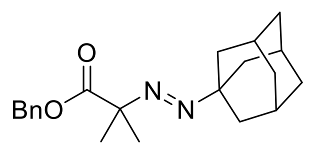

Benzyl 2-((*E*)-((*1s,3s*)-adamantan-1-yl)diazenyl)-2-methylpropanoate (3ap): (Changed condition : 1 h., Colorless oil, *n*-Hexane/Et_2O = 100/1 to 50/1, 64% yield, 43.3 mg); ^{1}H NMR (500 MHz, $CDCl_3$) δ 7.36–7.28 (m, 5H, Ar*H*), 5.14 (s, 2H, OC*H₂*), 2.13 (s, 3H, Ad*H*), 1.74–1.64 (m, 12H, Ad*H*), 1.45 (s, 6H, CC*H₃*); ^{13}C NMR (125 MHz,

$CDCl_3$) δ 173.6, 136.2, 128.4, 127.9, 127.9, 74.6, 67.8, 66.2, 40.0, 36.5, 29.2, 22.9; IR (neat) 2905, 2849, 1738, 1499, 1454, 1381, 1362, 1308, 1279, 1209, 1142, 1103, 1082, 1030, 1009, 974, 812, 733, 696, 584, 546, 501, 484, 442, 401 cm^{-1}; HRMS (DART) *m/z* calc'd. for $C_{21}H_{28}N_2O_2$ (M + H)$^+$ 341.2224, found 341.2237.

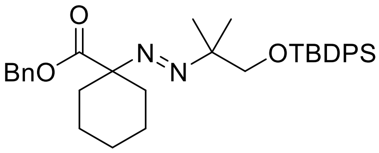

Benzyl (*E*)-1-((1-((*tert*-butyldiphenylsilyl)oxy)-2-methylpropan-2-yl)diazenyl)cyclohexane-1-carboxylate (3dq): (Changed condition : 0.50 mmol scale., Colorless oil, 1st: *n*-Hexane/Et_2O = 100/1 to 50/1, 2nd: *n*-Hexane/EtOAc = 100/1 to 50/1, 74% yield, 204.7 mg); ^{1}H NMR (500 MHz, $CDCl_3$) δ 7.65 (dd, *J* = 7.9 Hz, 1.5 Hz, 4H, Ar*H*), 7.43–7.40 (m, 2H, Ar*H*), 7.39–7.35 (m, 4H, Ar*H*), 7.27–7.25 (m, 5H, Ar*H*), 5.09 (s, 2H, ArOC*H₂*), 3.77 (s, 2H, SiOCH_2), 2.11–2.07 (m, 2H, CH_2CH_2CH_2), 1.97–1.92 (m, 2H, CH_2CH_2CH_2), 1.77–1.68 (m, 2H, CH_2CH_2CH_2), 1.57–1.53 (m, 1H, CH_2CH_2CH_2), 1.49–1.37 (m, 3H, CH_2CH_2CH_2), 1.09 (s, 6H, CH_3), 1.01 (s, 9H, CH_3); ^{13}C NMR (125 MHz, $CDCl_3$) δ 172.3, 135.7, 133.6, 129.6, 128.3, 128.0, 127.9, 127.6, 79.1, 71.6, 69.7, 66.1, 32.1, 26.8, 25.3, 22.5, 21.5, 19.3; IR (neat) 2932, 2857, 2372, 1736, 1472, 1454, 1427, 1389, 1360, 1312, 1198, 1155, 1132, 1109, 997, 937, 822, 739, 700, 613, 517, 503, 492, 473 cm^{-1}; HRMS (DART) *m/z* calc'd. for $C_{34}H_{44}N_2O_3Si$ (M + H)$^+$ 557.3194, found 557.3201.

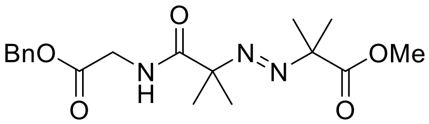

Methyl (*E*)-2-((1-((2-(benzyloxy)-2-oxoethyl)amino)-2-methyl-1-oxopropan-2-yl)diazenyl)-2-methylpropanoate (3sb): (Colorless oil, 1st : *n*-Hexane/EtOAc = 50/1 to 10/1 to 5/1, 2nd : *n*-Hexane/Et_2O = 50/1 to 10/1 to 5/1, 52% yield, 37.4 mg); ^{1}H NMR (500 MHz, $CDCl_3$) δ 7.39–7.32 (m, 6H, Ar*H*+N*H*), 5.19 (s, 2H, OCH_2), 4.17 (d, *J* = 5.2 Hz, 2H, NCH_2), 3.73 (s, 3H, OCH_3), 1.51 (s, 6H, CCH_3), 1.32 (s, 6H, CCH_3); ^{13}C NMR (125 MHz, $CDCl_3$) δ 174.5, 173.4, 169.6, 135.2, 128.6, 128.5, 128.4, 76.0, 74.0, 67.2, 52.3, 41.4, 22.8; IR (neat) 3397, 2984, 2936, 1736, 1676, 1508, 1456, 1383, 1356, 1281, 1186, 1173, 1150, 1013, 962, 866, 831, 802, 752, 739, 698, 679, 669, 577, 546, 532, 511, 494, 457, 449, 436, 430, 405 cm^{-1}; HRMS (DART) *m/z* calc'd. for $C_{18}H_{25}N_3O_5$ (M + H)$^+$ 364.1867, found 364.1879.

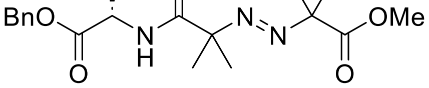

Methyl (*S,E*)-2-((1-((1-(benzyloxy)-1-oxopropan-2-yl)amino)-2-methyl-1-oxopropan-2-yl)diazenyl)-2-methylpropanoate (3tb): (Colorless oil, 1st : *n*-Hexane/EtOAc = 50/1 to 10/1 to 5/1, 2nd : *n*-Hexane/Et_2O = 50/1 to 10/1 to 5/1, 47% yield, 35.8 mg); ^1H NMR (500 MHz, $CDCl_3$) δ 7.38–7.30 (m, 6H, Ar*H*+N*H*), 5.18 (dd, *J* = 25.5 Hz, 12.3 Hz, 2H, OC*H₂*), 4.68 (quintet, *J* = 7.2 Hz, 1H, NC*H*), 3.72 (s, 3H, OC*H₃*), 1.51 (s, 3H, CC*H₃*), 1.50 (s, 3H, CC*H₃*), 1.45 (d, *J* = 7.1 Hz, 3H, CHC*H₃*), 1.31 (s, 3H, CC*H₃*), 1.30 (s, 3H, CC*H₃*); ^{13}C NMR (125 MHz, $CDCl_3$) δ 173.8, 173.4, 172.6, 135.4, 128.6, 128.4, 128.1, 76.0, 74.0, 67.0, 52.3, 48.1, 22.9, 22.8, 22.7, 18.3; IR (neat) 3395, 2984, 2934, 2344, 2332, 1736, 1678, 1503, 1452, 1381, 1339, 1281, 1186, 1146, 1051, 1030, 1011, 989, 908, 835, 770, 748, 698, 685, 667, 648, 581, 542, 532, 517, 457, 447, 436, 430, 407 cm^{-1}; HRMS (DART) *m/z* calc'd. for $C_{19}H_{27}N_3O_5$ $(M + H)^+$ 378.2024, found 378.2038.

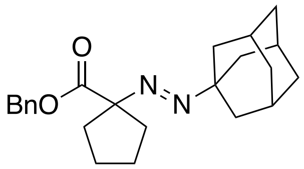

Benzyl 1-((*E*)-((1s,3s)-adamantan-1-yl)diazenyl)cyclopentane-1-carboxylate (3cp): (Changed condition : 1 h., Colorless oil, *n*-Hexane/Et_2O = 100/1 to 50/1, 51% yield, 37.0 mg); ^1H NMR (500 MHz, CD_3OD) δ 7.25–7.17 (m, 5H, ArH), 5.01 (s, 2H, OCH_2), 2.11–2.00 (m, 7H, AdH, CH_2CH_2), 1.68–1.66 (m, 3H, AdH, CH_2CH_2), 1.63–1.53 (m, 13H, AdH, CH_2CH_2); ^{13}C NMR (125 MHz, CD_3OD) δ 173.1, 136.2, 128.0, 127.8, 127.7, 85.7, 67.3, 66.1, 39.8, 36.2, 33.7, 29.2, 24.3; IR (neat) 2936, 2903, 2851, 1736, 1452, 1261, 1165, 1069, 1016, 733, 694 cm^{-1}; HRMS (DART) m/z calc'd. for $C_{23}H_{31}N_2O_2$ $(M + H)^+$ 367.2380, found 367.2368.

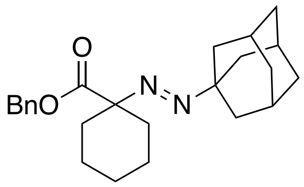

Benzyl 1-((E)-((1s,3s)-adamantan-1-yl)diazenyl)cyclohexane-1-carboxylate (3dp): (Changed condition : 1 h., White solid, *n*-Hexane/Et_2O = 100/1 to 50/1, 56% yield, 42.6 mg); ^1H NMR (500 MHz, $CDCl_3$) δ 7.37–7.27 (m, 5H, Ar*H*), 5.12 (s, 2H, OC*H₂*), 2.11 (s, 3H, Ad*H*), 2.09–2.04 (m, 2H, $CH_2CH_2CH_2$), 1.96–1.90 (m, 2H, $CH_2CH_2CH_2$), 1.74–1.63 (m, 14H, Ad*H*, $CH_2CH_2CH_2$), 1.58–1.52 (m, 1H, $CH_2CH_2CH_2$), 1.50–1.37 (m, 3H, $CH_2CH_2CH_2$); ^{13}C NMR (125 MHz, $CDCl_3$) δ 172.5, 136.3, 128.4, 128.0, 127.9, 78.6, 68.1, 66.0, 40.0, 36.5, 32.1, 29.2, 25.3, 22.5; IR (neat) 2905, 2849, 1726, 1450, 1310, 1213, 1196, 1153, 1130, 941, 748, 737, 694, 608, 496, 476 cm^{-1}; HRMS (DART) *m/z* calc'd. for $C_{24}H_{33}N_2O_2$ $(M + H)^+$ 381.2537, found 381.2535.

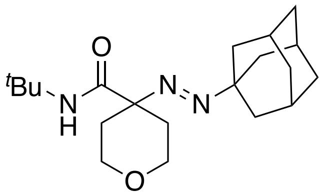

4-((*E*)-((1s,3s)-adamantan-1-yl)diazenyl)-N-(tert-butyl)tetrahydro-2H-pyran-4-carboxamide (3kp): (Changed condition : 1 h., White solid, *n*-Hexane/EtOAc = 50/1 to 10/1 to 2/1, 47% yield, 32.7 mg); ^{1}H NMR (500 MHz, $CDCl_3$) δ 6.02 (br, 1H, N*H*), 3.82 (dt, *J* = 11.5 Hz, 4.6 Hz, 2H, OC*H₂*), 3.50–3.45 (m, 2H, OC*H₂*), 2.29–2.24 (m, 2H, CC*H₂*), 2.20 (s, 3H, Ad*H*), 2.01–1.97 (m, 2H, CC*H₂*), 1.80–1.78 (m, 9H, Ad*H*), 1.72–1.69 (m, 3H, Ad*H*), 1.31 (s, 9H, C*H₃*); ^{13}C NMR (125 MHz, $CDCl_3$) δ 171.2, 73.1, 69.1, 63.9, 50.9, 40.3, 36.5, 31.7, 29.1, 28.7; IR (neat) 2911, 2851, 1674, 1506, 1454, 1360, 1229, 1105, 1030, 854, 557, 480 cm^{-1}; HRMS (DART) *m/z* calc'd. for $C_{20}H_{34}N_3O_2$ $(M + H)^+$ 348.2646, found 348.2661.

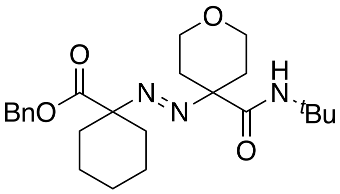

Benzyl (*E*)-1-((4-(tert-butylcarbamoyl)tetrahydro-2H-pyran-4-yl)diazenyl)cyclohexane-1-carboxylate (3dk): (White solid, *n*-Hexane/Et_2O = 50/1 to 20/1 to 10/1 to 2/1, 64% yield, 55.0 mg); ^{1}H NMR (500 MHz, CD_3OD) δ 7.27–7.21 (m, 5H, Ar*H*), 6.09 (br, 1H, N*H*), 5.08 (s, 2H, ArC*H₂*), 3.59 (dt, *J* = 11.8 Hz, 4.4 Hz, 2H, OC*H₂*), 3.27–3.22 (m, 2H, OC*H₂*), 2.10–2.02 (m, 4H, CC*H₂*, C*H₂*C*H₂*C*H₂*), 2.00–1.95 (m, 2H, C*H₂*C*H₂*C*H₂*), 1.83–1.80 (m, 2H, CC*H₂*), 1.65–1.59 (m, 2H, C*H₂*C*H₂*C*H₂*), 1.53–1.48 (m, 1H, C*H₂*C*H₂*C*H₂*), 1.47–1.38 (m, 3H, C*H₂*C*H₂*C*H₂*), 1.16 (s, 9H, C*H₃*); ^{13}C NMR (125 MHz, CD_3OD) δ 171.9, 171.0, 135.9, 128.2, 128.1, 128.1, 80.4, 74.7, 66.4, 63.3, 51.2, 31.7, 30.7, 27.5, 24.7, 22.0; IR (neat) 2943, 2934, 2847, 1726, 1676, 1520, 1452, 1366, 1204, 1153, 1134, 1103, 951, 750, 700, 611, 592, 556, 469 cm^{-1}; HRMS (DART) *m/z* calc'd. for $C_{24}H_{36}N_3O_4$ $(M + H)^+$ 430.2700, found 430.2709.

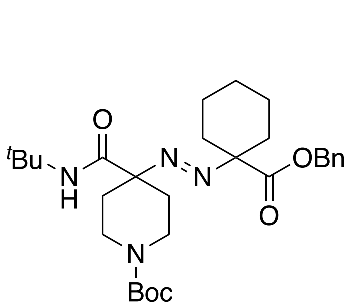

***tert*-butyl (*E*)-4-((1-((benzyloxy)carbonyl)cyclohexyl)diazenyl)-4-(tert-butylcarbamoyl)piperidine-1-carboxylate (3jd):** (White solid, *n*-Hexane/Et_2O = 50/1 to 10/1 to 2/1, 49% yield, 52.5 mg); ^{1}H NMR (500 MHz, CD_3OD) δ 7.26–7.21 (m, 5H, Ar*H*), 6.11 (br, 1H, N*H*), 5.08 (s, 2H, ArC*H₂*), 3.57 (dt, *J* = 13.6 Hz, 4.3 Hz, 2H, NC*H₂*), 2.71 (br, 2H, NC*H₂*), 2.06–1.90 (m, 6H, C*H₂*C*H₂*C*H₂*), 1.85–1.81 (m, 2H, CC*H₂*), 1.62–1.58 (m, 2H, CC*H₂*), 1.53–

1.40 (m, 4H, C*H₂*C*H₂*C*H₂*), 1.35 (s, 9H, C*H₃*), 1.16 (s, 9H, C*H₃*); ^{13}C NMR (125 MHz, CD_3OD) δ 171.8, 171.0, 155.0, 135.9, 128.2, 128.2, 128.1, 80.5, 79.8, 75.4, 66.4, 51.2, 39.9, 38.9, 31.7, 30.0, 27.5, 27.3, 24.6, 22.0; IR (neat) 2972, 2936, 2866, 1730, 1688, 1682, 1514, 1454, 1422, 1364, 1246, 1223, 1173, 1136, 1072, 957, 864, 750, 696, 465 cm^{-1}; HRMS (DART) *m/z* calc'd. for $C_{29}H_{45}N_4O_5$ $(M + H)^+$ 529.3384, found 529.3395.

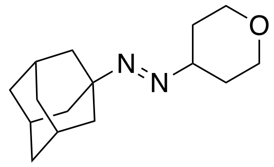

(*E*)-1-((1s,3s)-adamantan-1-yl)-2-(tetrahydro-2H-pyran-4-yl)diazene (3py): (Yellow solid, *n*-Hexane/Ethyl acetate = 100/1 to 50/1 to 10/1, 13% yield, 6.56 mg); ^{1}H NMR (500 MHz, $CDCl_3$) δ 4.07 (td, *J* = 7.7 Hz, 4.0 Hz, 2H, OCH_2), 3.55–3.49 (m, 3H,NC*H*, OCH_2), 2.16 (s, 3H, Ad*H*), 2.01–1.93 (m, 2H, OCH_2CH_2), 1.76–1.67 (m, 14H, OCH_2CH_2, Ad*H*); ^{13}C NMR (125 MHz, $CDCl_3$) δ 72.4, 67.3, 66.2, 40.3, 36.6, 30.6, 29.2; IR (neat) 2903, 2849, 1450, 1352, 1308, 1234, 1134, 1088, 1065, 1009, 982, 827, 814, 598, 503 cm^{-1}; HRMS (DART) *m/z* calc'd. for $C_{15}H_{21}N_2O$ $(M + H)^+$ 249.1961, found 249.1952.

5-3. Peptide synthesis (2x)

(1) Fmoc-solid phase peptide synthesis

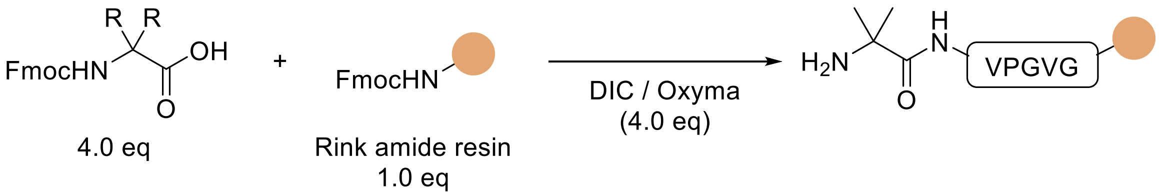

(2) Diazene synthesis

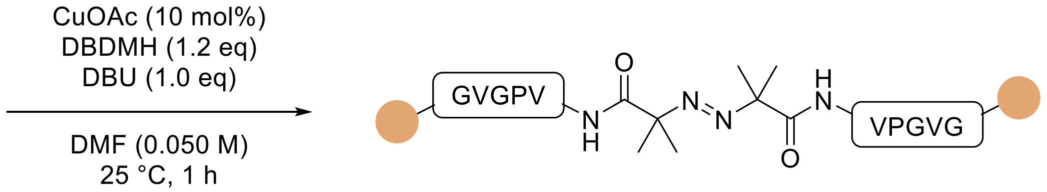

(3) Deprotection

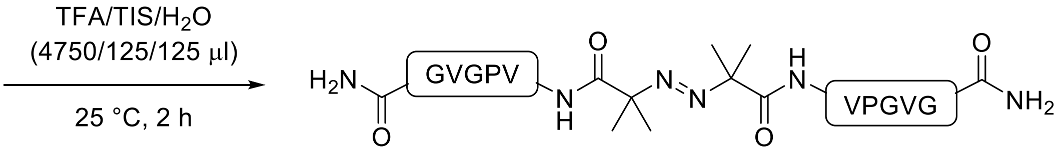

2α: 11%

Fmoc solid-phase peptide synthesis & catalytic diazene synthesis

The peptides were synthesized using Biotage® Initiator+ Alstra™ following the FPPS strategy and using Fmoc Rink Amide ProTide resin (LL) (loading 0.18 mmol/g). The following Fmoc-amino acids were used: Fmoc-Gly-OH, Fmoc-Pro-OH, Fmoc-Val-OH and Fmoc-Aib-OH. The Fmoc deprotections were performed using a solution of 20% piperidine in DMF (4.5 ml). The coupling steps were performed using Fmoc-protected amino acids (4.0 eq, 0.50 M) and a mixture of DIC (4.0 eq, 0.50 M) and OxymaPure (4.0 eq, 0.50 M) at 75 °C for 10 min under microwave irradiation.

Next, to the crude equipped with a magnetic stirrer bar, CuOAc (1.2 mg, 10 µmol, 10 mol%) was added followed by the addition of DMF (2.0 ml, 0.050 M), 1,3-dibromo-5,5-dimethylhydantoin (34.3 mg, 0.12 mmol, 1.2 eq) and 1,8-diazabicyclo[5.4.0]undec-7-ene (15 µl, 0.10 mmol, 1.0 eq). The reaction mixture was stirred at 25 °C for 1 h. The crude peptide was then washed with DMF, H2O, and MeOH and dried.

The cleavage and side-chain deprotections were performed using a cocktail cleavage composed by TFA/TIS/H_2O (4750:125:125 µl) at room temperature for 2 h. Then, the resin was filtered, followed by precipitation with cold Et_2O. The suspensions were centrifuged and lyophilized. The crude peptide was purified by HPLC to give product.

Table S1. HPLC conditions for the purification of 2α

Flow rate 15 mL/min

A: 0.1%TFA/H_2O, **B**: 0.1%TFA/MeCN

**min**	**A**	**B**
**0**	**95%**	**5%**
**10**	**95%**	**5%**
**60**	**0%**	**100%**

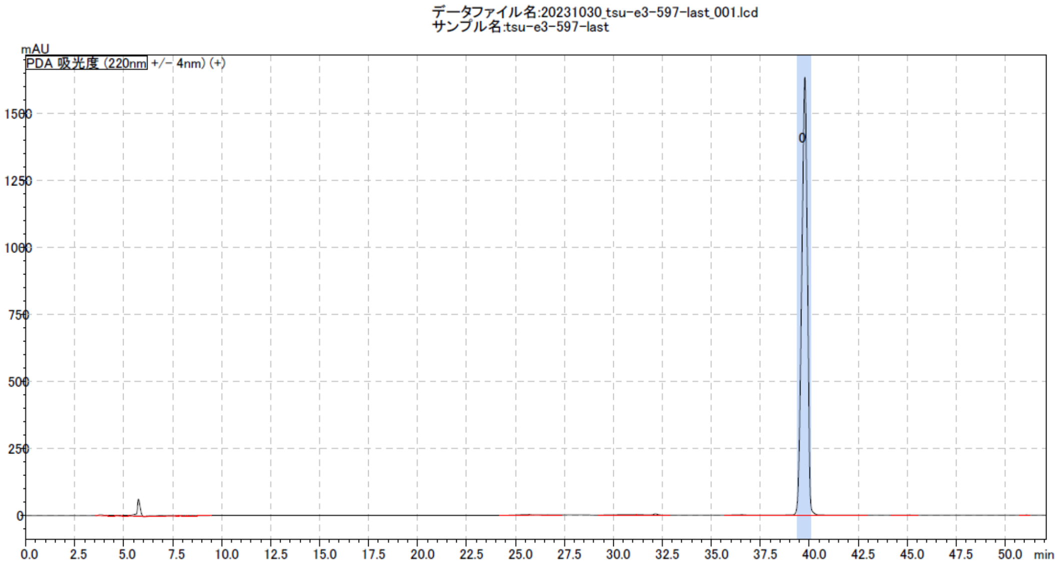

Figure S1. HPLC chromatogram of the purified compound 2α

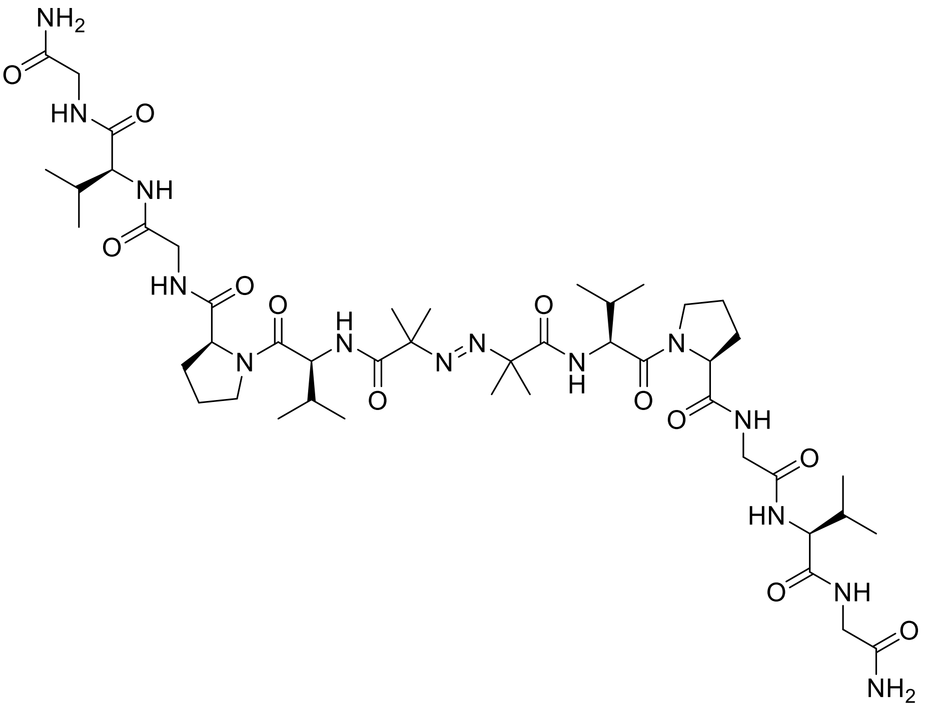

(2*S*,2'*S*)-1,1'-((2*S*,2'*S*)-2,2'-((2,2'-((*E*)-diazene-1,2-diyl)bis(2-methylpropanoyl))bis(azanediyl))bis(3-methylbutanoyl))bis(N-(2-(((*S*)-1-((2-amino-2-oxoethyl)amino)-3-methyl-1-oxobutan-2-yl)amino)-2-oxoethyl)pyrrolidine-2-carboxamide) (2α): (white solid, 0.10 mmol, 5.5 mg, 11% yield.); HRMS (ESI) *m/z* calc'd. for $C_{46}H_{78}N_{14}O_{12}$ (M + Na)$^+$ 1041.5816 found 1041.5799; $[\alpha]^{23}_D$ = −60.0 (c 0.25, H_2O).

6. Transformation of the Products

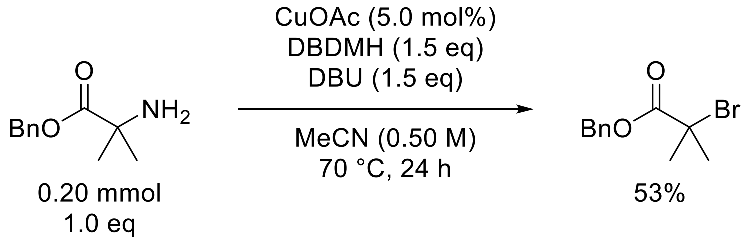

Condition: To a 4 ml vial equipped with a magnetic stirrer bar, CuOAc (1.2 mg, 10 µmol, 5.0 mol%) was added in a glove box followed by the addition of MeCN (0.40 ml, 0.50 M), 1,3-dibromo-5,5-dimethylhydantoin (85.8 mg, 0.30 mmol, 1.5 eq), benzyl 2-amino-2-methylpropanoate (39 µl, 0.20 mmol, 1.0 eq) and 1,8-diazabicyclo[5.4.0]undec-7-ene (45 µl, 0.30 mmol, 1.5 eq) under Ar atmosphere. The reaction mixture was stirred at 70 °C for 24 h and diluted with DCM. The diluted solution was filtered through silica short column and washed with *n*-Hexane/EtOAc = 4/1. After evaporation of the organic solvent under reduced pressure, the resultant mixture was purified by silica gel flash chromatography (*n*-Hexane/Et_2O = 100/1 to 50/1) to obtain the desired compound.

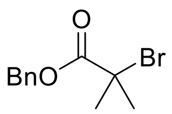

Benzyl 2-bromo-2-methylpropanoate (4): CAS Registry Number 75107-16-3 (Colorless oil, 53% yield, 27.0 mg); ^{1}H NMR (500 MHz, $CDCl_3$) δ 7.39–7.32 (m, 5H, Ar*H*), 5.21 (s, 2H, OC*H*$_2$), 1.95 (s, 6H, C*H*$_3$); ^{13}C NMR (125 MHz, $CDCl_3$) δ 171.5, 135.4, 128.6, 128.3, 127.9, 67.6, 55.7, 30.8; HRMS (DART) *m/z* calc'd. for $C_{11}H_{13}O_2Br$ $(M + NH_4)^+$ 274.0437, found 274.0447.

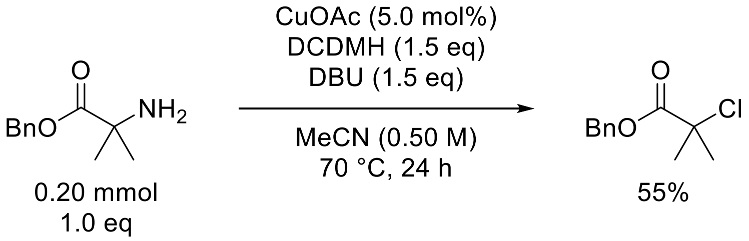

Condition: To a 4 ml vial equipped with a magnetic stirrer bar, CuOAc (1.2 mg, 10 µmol, 5.0 mol%) was added in a glove box followed by the addition of MeCN (0.40 ml, 0.50 M), 1,3-dichloro-5,5-dimethylhydantoin (59.1 mg, 0.30 mmol, 1.5 eq), benzyl 2-amino-2-methylpropanoate (39 µl, 0.20 mmol, 1.0 eq) and 1,8-diazabicyclo[5.4.0]undec-7-ene (45 µl, 0.30 mmol, 1.5 eq) under Ar atmosphere. The reaction mixture was stirred at 70 °C for 24 h and diluted with DCM. The diluted solution was filtered through silica short column and washed with *n*-Hexane/EtOAc = 4/1. After evaporation of the organic solvent under reduced pressure, the

resultant mixture was purified by silica gel flash chromatography (*n*-Hexane/Et_2O = 100/1 to 50/1) to obtain the desired compound.

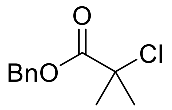

Benzyl 2-chloro-2-methylpropanoate (5): CAS Registry Number 2126701-14-0 (Colorless oil, *n*-Hexane/Et_2O = 100/1 to 50/1, 55% yield, 23.2 mg); ^{1}H NMR (500 MHz, $CDCl_3$) δ 7.38–7.32 (m, 5H, Ar*H*), 5.21 (s, 2H, OC*H₂*), 1.80 (s, 6H, C*H₃*); ^{13}C NMR (125 MHz, $CDCl_3$) δ 171.5, 135.4, 128.6, 128.4, 127.9, 67.6, 64.6, 29.8; HRMS (DART) *m/z* calc'd. for $C_{11}H_{13}O_2Cl$ $(M + NH_4)^+$ 230.0942, found 230.0946.

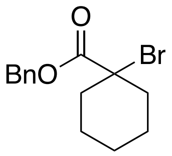

benzyl 1-bromocyclohexane-1-carboxylate (6): (Colorless oil, *n*-Hexane only to *n*-Hexane/toluene = 20/1, 14% yield, 8.4 mg); ^{1}H NMR (500 MHz, $CDCl_3$) δ 7.39–7.31 (m, 5H, Ar*H*), 5.23 (s, 2H, OCH_2), 2.18 (br, 4H, $CH_2CH_2CH_2$), 1.77–1.71 (m, 2H, $CH_2CH_2CH_2$), 1.51–1.50 (m, 3H, $CH_2CH_2CH_2$), 1.42–1.36 (m, 1H, $CH_2CH_2CH_2$); ^{13}C NMR (125 MHz, $CDCl_3$) δ 170.9, 135.5, 128.6, 128.3, 128.0, 67.3, 37.9, 24.7, 23.7; IR (neat) 2938, 2860, 1732, 1449, 1375, 1281, 1250, 1233, 1200, 1146, 1128, 1057, 1005, 735, 696, 679, 474 cm^{-1}; HRMS (DART) *m/z* calc'd. for $C_{14}H_{21}BrNO_2$ $(M + NH_4)^+$ 314.0750, found 314.0751.

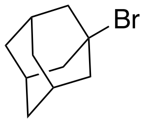

(3s,5s,7s)-1-bromoadamantane (7): CAS Registry Number 768-90-1 (Changed condition : CuOAc 10 mol%, DBDMH 3.0 equiv, DBU 3.0 equiv, DMF 1.0 M, 3 h, White solid, *n*-Hexane only to *n*-Hexane/Et_2O = 50/1, 28% yield, 11.9 mg); ^{1}H NMR (500 MHz, $CDCl_3$) δ 2.37 (d, *J* = 3.1 Hz, 6H, Ad*H*), 2.10 (br, 3H, Ad*H*), 1.73 (t, *J* = 3.1 Hz, 2H, Ad*H*); ^{13}C NMR (125 MHz, $CDCl_3$) δ 66.8, 49.3, 35.5, 32.6; IR (neat) 2905, 2851, 1454, 1342, 1288, 1101, 1026, 947, 806, 764, 675, 463 cm^{-1}.

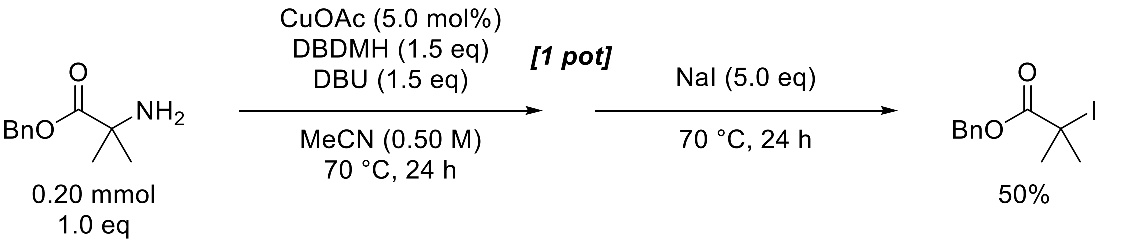

Condition: To a 4 ml vial equipped with a magnetic stirrer bar, CuOAc (1.2 mg, 10 µmol, 5.0 mol%) was added in a glove box followed by the addition of MeCN (0.40 ml, 0.50 M), 1,3-dibromo-5,5-dimethylhydantoin (85.8 mg, 0.30 mmol, 1.5 eq), benzyl 2-amino-2-methylpropanoate (39 µl, 0.20 mmol, 1.0 eq) and 1,8-diazabicyclo[5.4.0]undec-7-ene (45 µl, 0.30 mmol, 1.5 eq) under Ar atmosphere. The reaction mixture was stirred at 70 °C for 24 h. On cooling to room temperature, sodium iodide (150 mg, 1.0 mmol, 5.0 eq) was added to the solution and the mixture was stirred at 70 °C for 24 h. On cooling to room temperature, the mixture was purified by silica gel flash chromatography (*n*-Hexane/DCM = 50/1 to 20/1 to 5/1) to obtain the desired compound.

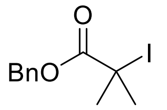

Benzyl 2-iodo-2-methylpropanoate (8): (Colorless oil, *n*-Hexane/DCM = 50/1 to 20/1 to 10/1 to 5/1, 50% yield, 30.2 mg); ^{1}H NMR (500 MHz, $CDCl_3$) δ 7.41–7.33 (m, 5H, Ar*H*), 5.19 (s, 2H, OCH_2), 2.10 (s, 6H, CH_3); ^{13}C NMR (125 MHz, $CDCl_3$) δ 173.2, 135.5, 128.6, 128.3, 128.0, 67.6, 33.7, 33.3; IR (neat) 2972, 2922, 1724, 1497, 1456, 1387, 1369, 1261, 1211, 1148, 1101, 1030, 1011, 966, 903, 876, 812, 733, 694, 637, 600, 583, 527, 507, 488, 457, 424, 419, 407, 401 cm^{-1}; HRMS (DART) *m/z* calc'd. for $C_{11}H_{13}O_2I$ $(M + NH_4)^+$ 322.0299, found 322.0300.

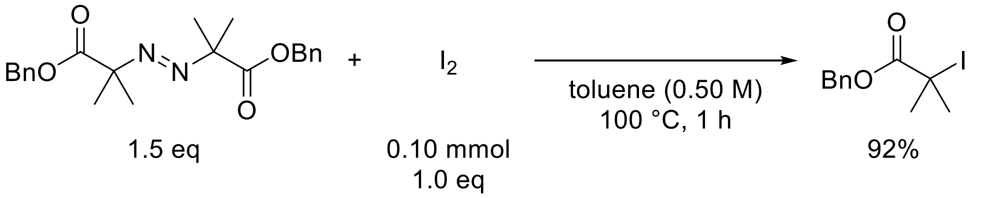

Condition[14]**:** The solution with I_2 (25.4 mg, 0.10 mmol, 1.0 eq), dibenzyl 2,2'-(diazene-1,2-diyl)(*E*)-bis(2-methylpropanoate) (57.4 mg, 0.15 mmol, 1.5 eq), and toluene (0.20 ml, 0.50 M) was heated under Ar atmosphere at 100 °C in the absence of light. After 1 h, the solution was cooled to room temperature, it was purified by flash column chromatography (*n*-Hexane/Et_2O = 100/1 to 50/1 to 30/1) to afford the desired compound (92% yield, 55.7 mg).

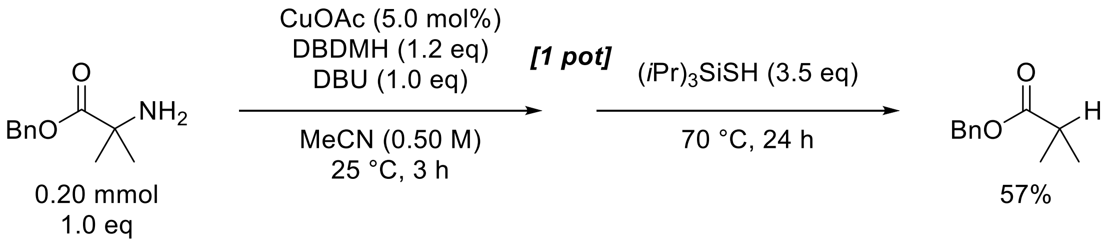

Condition: To a 4 ml vial equipped with a magnetic stirrer bar, CuOAc (1.2 mg, 10 µmol, 5.0 mol%) was added in a glove box followed by the addition of MeCN (0.40 ml, 0.50 M), 1,3-dibromo-5,5-dimethylhydantoin (68.6 mg, 0.24 mmol, 1.2 eq), benzyl 2-amino-2-methylpropanoate (39 µl, 0.20 mmol, 1.0 eq) and 1,8-diazabicyclo[5.4.0]undec-7-ene (30 µl, 0.20 mmol, 1.0 eq) under Ar atmosphere. The reaction mixture was stirred at 25 °C for 3 h. Then, triisopropylsilanethiol (150 µl, 0.70 mmol, 3.5 eq) was added to the solution and the mixture was stirred at 70 °C for 24 h. On cooling to room temperature, the mixture was purified by silica gel flash chromatography to obtain the desired compound.

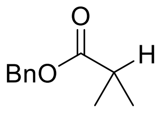

Benzyl isobutyrate (9): CAS Registry Number 103-28-6 (Colorless oil, *n*-Hexane/Et_2O = 70/1 to 40/1 to 20/1, 57% yield, 20.2 mg); ^{1}H NMR (500 MHz, $CDCl_3$) δ 7.38–7.30 (m, 5H, Ar*H*), 5.12 (s, 2H, OCH_2), 2.65–2.56 (m, 1H, C*H*), 1.19 (d, *J* = 7.0 Hz, 6H, CH_3); ^{13}C NMR (125 MHz, $CDCl_3$) δ 177.0, 136.3, 128.5, 128.1, 128.0, 66.0, 34.0, 19.0; HRMS (DART) *m/z* calc'd. for $C_{11}H_{14}O_2$ $(M + NH_4)^+$ 196.1332, found 196.1332.

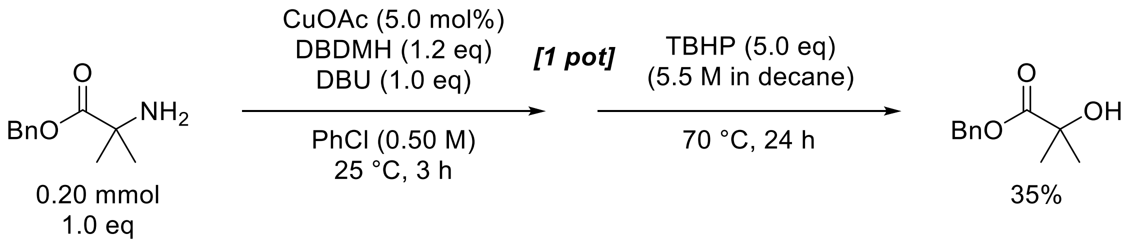

Condition: To a 4 ml vial equipped with a magnetic stirrer bar, CuOAc (1.2 mg, 10 µmol, 5.0 mol%) was added in a glove box followed by the addition of chlorobenzene (0.40 ml, 0.50 M), 1,3-dibromo-5,5-dimethylhydantoin (68.6 mg, 0.24 mmol, 1.2 eq), benzyl 2-amino-2-methylpropanoate (39 µl, 0.20 mmol, 1.0 eq) and 1,8-diazabicyclo[5.4.0]undec-7-ene (30 µl, 0.20 mmol, 1.0 eq) under Ar atmosphere. The reaction mixture was stirred at 25 °C for 3 h. Then, *tert*-butyl hydroperoxide (5.5 M in decane) (182 µl, 1.0 mmol, 10 eq) was added to the solution and the mixture was stirred at 70 °C for 24 h. On cooling to room temperature, the mixture was purified by silica gel flash chromatography to obtain the desired compound.

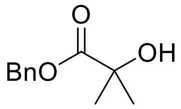

Benzyl 2-hydroxy-2-methylpropanoate (10): CAS Registry Number 19444-23-6 (Pale yellow oil, *n*-Hexane/EtOAc = 100/1 to 50/1 to 10/1 to 8/1, 35% yield, 13.7 mg); ^{1}H NMR (500 MHz, $CDCl_3$) δ 7.40–7.33 (m, 5H, Ar*H*), 5.20 (s, 2H, OC*H$_2$*), 3.08 (br, 1H, O*H*), 1.45 (s, 6H, C*H$_3$*); ^{13}C NMR (125 MHz, $CDCl_3$) δ 177.3, 135.4, 128.7, 128.5, 128.0, 72.1, 67.4, 27.2; HRMS (DART) *m/z* calc'd. for $C_{11}H_{14}O_3$ $(M + H)^+$ 195.1016, found 195.1012.

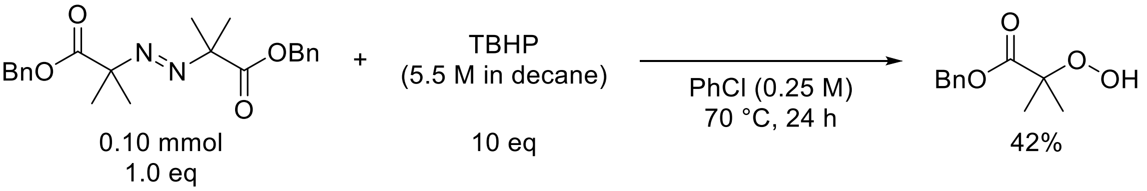

Condition: The solution with dibenzyl 2,2'-(diazene-1,2-diyl)(*E*)-bis(2-methylpropanoate) (38.2 mg, 0.10 mmol, 1.0 eq), chlorobenzene (0.40 ml, 0.25 M) and *tert*-butyl hydroperoxide (5.5 M in decane) (182 µl, 1.0 mmol, 10 eq) was heated under Ar atmosphere at 70 °C. After 24 h, the solution was cooled to room temperature, it was purified by flash column chromatography to afford the desired compound.

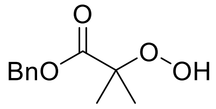

Benzyl 2-hydroperoxy-2-methylpropanoate (11): (Colorless oil, 1st: *n*-Hexane/Et_2O = 100/1 to 50/1 to 20/1, 2nd: *n*-Hexane/EtOAc = 100/1 to 50/1 to 30/1, 42% yield, 17.5 mg); ^{1}H NMR (500 MHz, $CDCl_3$) δ 9.01 (br, 1H, O*H*), 7.40–7.33 (m, 5H, Ar*H*), 5.22 (s, 2H, OC*H$_2$*), 1.50 (s, 6H, C*H$_3$*); ^{13}C NMR (125 MHz, $CDCl_3$) δ 174.3, 135.3, 128.7, 128.5, 128.0, 83.7, 67.1, 22.6; IR (neat) 3445, 1726, 1499, 1456, 1381, 1362, 1281, 1213, 1144, 1082, 968, 943, 914, 847, 795, 750, 737, 696, 600, 583, 554, 521, 471, 457, 438, 428, 420, 413, 407 cm^{-1}; HRMS (DART) *m/z* calc'd. for $C_{11}H_{14}O_4$ $(M + NH_4)^+$ 228.1230, found 228.1229.

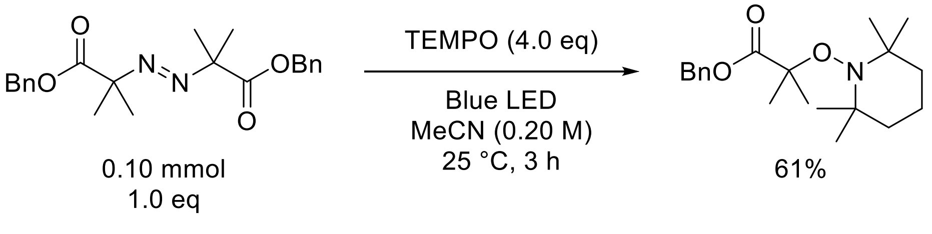

Condition: The solution with dibenzyl 2,2'-(diazene-1,2-diyl)(*E*)-bis(2-methylpropanoate) (38.2 mg, 0.10 mmol, 1.0 eq), TEMPO (62.5 mg, 0.40 mmol, 4.0 eq) and MeCN (0.50 ml, 0.20 M) was stirred and irradiated using two

40 W blue LEDs (Kessil A160WE TUNA Blue, 2 cm away from the light sources) with a fan for 3 hours. After that, the solution was purified by flash column chromatography to afford the desired compound.

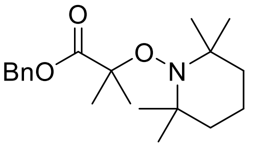

Benzyl 2-methyl-2-((2,2,6,6-tetramethylpiperidin-1-yl)oxy)propanoate (12): (White solid, *n*-Hexane/EtOAc = 100/1 to 50/1, 61% yield, 40.9 mg); ^{1}H NMR (500 MHz, $CDCl_3$) δ 7.40–7.30 (m, 5H, Ar*H*), 5.14 (s, 2H, OC*H₂*), 1.55–1.35 (m, 5H, CCH_2CH_2), 1.48 (s, 6H, CCH_3), 1.30–1.24 (m, 1H, CCH_2CH_2), 1.09 (s, 6H, CCH_3), 0.96 (s, 6H, CCH_3); ^{13}C NMR (125 MHz, $CDCl_3$) δ 175.8, 135.9, 128.6, 128.4, 128.1, 81.2, 66.5, 59.5, 40.6, 33.4, 24.5, 20.5, 17.1; IR (neat) 2974, 2930, 1715, 1470, 1458, 1450, 1373, 1358, 1277, 1260, 1242, 1215, 1204, 1153, 1132, 1082, 1059, 962, 941, 922, 905, 880, 806, 752, 698, 627, 602, 569, 501, 467 cm^{-1}; HRMS (DART) *m/z* calc'd. for $C_{20}H_{31}NO_3$ $(M + H)^+$ 334.2377, found 334.2385.

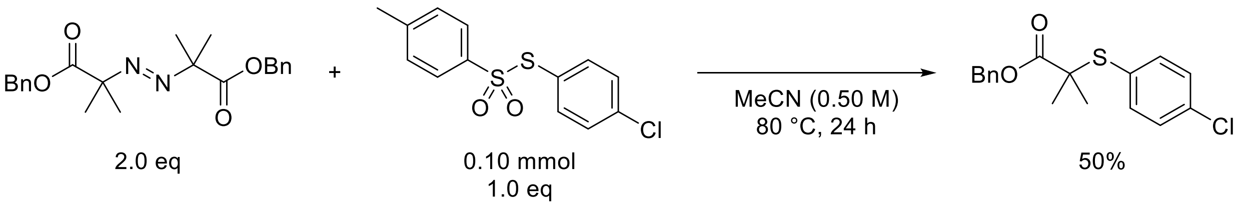

Condition: The solution with dibenzyl 2,2'-(diazene-1,2-diyl)(*E*)-bis(2-methylpropanoate) (76.5 mg, 0.20 mmol, 2.0 eq), *S*-(4-chlorophenyl) 4-methylbenzenesulfonothioate (29.9 mg, 0.10 mmol, 1.0 eq) and MeCN (0.20 ml, 0.50 M) was heated under Ar atmosphere at 80 °C. After 24 h, the solution was cooled to room temperature, it was purified by flash column chromatography to afford the desired compound.

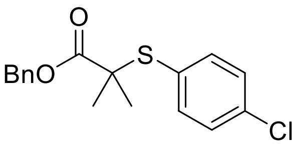

Benzyl 2-((4-chlorophenyl)thio)-2-methylpropanoate (13): (White solid, *n*-Hexane/EtOAc = 100/1 to 50/1, 50% yield, 16.0 mg); ^{1}H NMR (500 MHz, $CDCl_3$) δ 7.39–7.30 (m, 5H, Ar*H*), 7.22–7.16 (m, 4H, Ar*H*), 5.10 (s, 2H, OCH_2), 1.50 (s, 6H, CCH_3); ^{13}C NMR (125 MHz, $CDCl_3$) δ 173.4, 138.0, 135.8, 135.6, 129.8, 128.9, 128.5, 128.3, 128.3, 66.9, 51.1, 25.8; IR (neat) 2932, 1715, 1570, 1495, 1474, 1452, 1383, 1369, 1263, 1256, 1213, 1148, 1125, 1092, 1080, 1013, 959, 897, 829, 818, 752, 696, 608, 557, 503, 486, 403 cm^{-1}; HRMS (DART) *m/z* calc'd. for $C_{17}H_{17}O_2SCl$ $(M + H)^+$ 321.0711, found 321.0709.

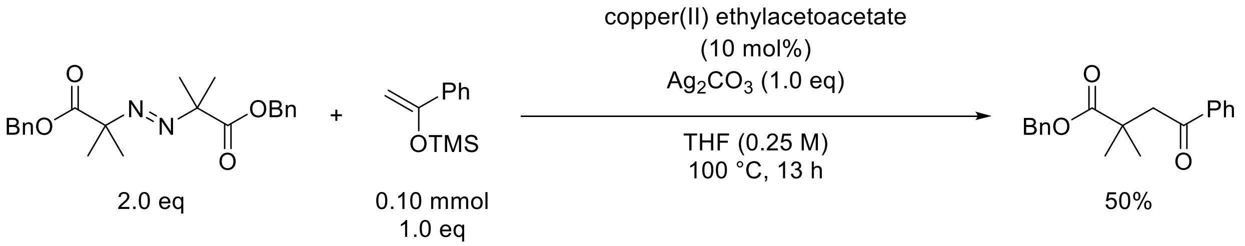

Condition:[15] The solution with dibenzyl 2,2'-(diazene-1,2-diyl)(*E*)-bis(2-methylpropanoate) (76.5 mg, 0.20 mmol, 2.0 eq), copper(II) ethylacetoacetate (3.2 mg, 0.010 mmol, 10 mol%), silver carbonate (27.6 mg, 0.10 mmol, 1.0 eq), THF (0.40 ml, 0.25 M) and trimethyl((1-phenylvinyl)oxy)silane (20 µl, 0.10 mmol, 1.0 eq) was heated under Ar atmosphere at 100 °C. After 13 h, the solution was cooled to room temperature, it was purified by flash column chromatography to afford the desired compound.

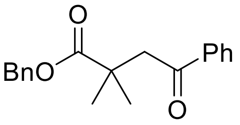

Benzyl 2,2-dimethyl-4-oxo-4-phenylbutanoate (14): (Colorless oil, *n*-Hexane/EtOAc = 100/1 to 50/1 to 20/1, 51% yield, 15.0 mg); ^{1}H NMR (500 MHz, $CDCl_3$) δ 7.93–7.92 (m, 2H, Ar*H*), 7.56 (tt, *J* = 7.4 Hz, 1.4 Hz, 1H, Ar*H*), 7.46–7.43 (m, 2H, Ar*H*), 7.31–7.25 (m, 5H, Ar*H*), 5.13 (s, 2H, OC*H*$_2$), 3.32 (s, 2H, CC*H*$_2$), 1.35 (s, 6H, CC*H*$_3$); ^{13}C NMR (125 MHz, $CDCl_3$) δ 197.6, 177.2, 137.0, 136.3, 133.1, 128.6, 128.4, 127.9, 127.9, 66.4, 48.5, 40.2, 25.8; IR (neat) 2963, 1730, 1686, 1597, 1580, 1497, 1474, 1449, 1404, 1387, 1352, 1300, 1225, 1186, 1148, 1121, 1078, 984, 935, 910, 868, 816, 787, 752, 691, 667, 613, 573, 530, 474, 459, 444, 420, 411, 405 cm^{-1}; HRMS (DART) *m/z* calc'd. for $C_{19}H_{20}O_3$ $(M + H)^+$ 297.1485, found 297.1491.

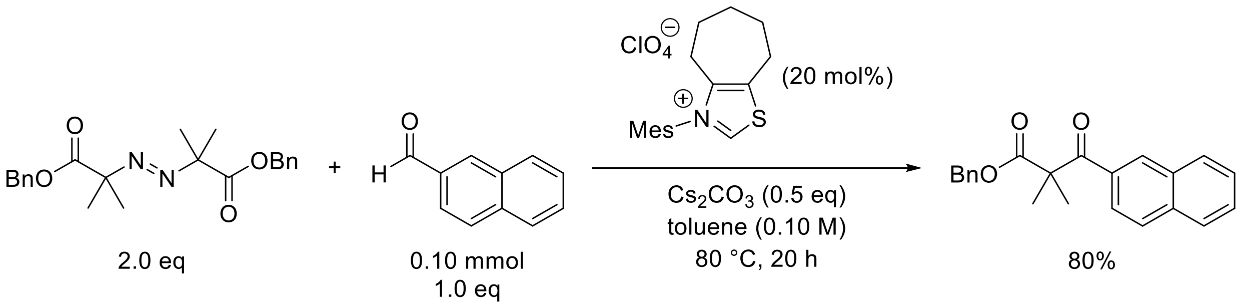

Condition:[16] The solution with dibenzyl 2,2'-(diazene-1,2-diyl)(*E*)-bis(2-methylpropanoate) (76.5 mg, 0.20 mmol, 2.0 eq), 3-mesityl-5,6,7,8-tetrahydro-4*H*-cyclohepta[*d*]thiazol-3-ium perchlorate (7.4 mg, 0.020 mmol, 20 mol%), 2-naphthaldehyde (15.6 mg, 0.10 mmol, 1.0 eq), cesium carbonate (16.3 mg, 0.050 mmol, 0.50 eq) and toluene (1.0 ml, 0.10 M) was heated under Ar atmosphere at 80 °C. After 20 h, the solution was cooled to room temperature, it was purified by flash column chromatography to afford the desired compound.

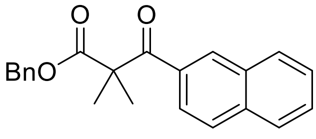

Benzyl 2,2-dimethyl-3-(naphthalen-2-yl)-3-oxopropanoate (15): (White gel, *n*-Hexane/EtOAc = 100/1 to 50/1 to 30/1, 80% yield, 26.6 mg); ^{1}H NMR (500 MHz, $CDCl_3$) δ 8.23 (s, 1H, Ar*H*), 7.85–7.82 (m, 2H, Ar*H*), 7.78–7.75 (m, 2H, Ar*H*), 7.59–7.56 (m, 1H, Ar*H*), 7.52–7.48 (m, 1H, Ar*H*), 7.12–7.09 (m, 1H, Ar*H*), 7.07–7.03 (m, 2H, Ar*H*), 7.01–6.99 (m, 2H, Ar*H*), 5.07 (s, 2H, OC*H₂*), 1.63 (s, 6H, C*CH₃*); ^{13}C NMR (125 MHz, $CDCl_3$) δ 197.6, 174.9, 135.2, 134.9, 132.4, 132.3, 130.1, 129.8, 128.5, 128.3, 128.2, 128.1, 127.6, 126.6, 124.5, 67.2, 53.5, 24.2; IR (neat) 2361, 1732, 1676, 1626, 1595, 1497, 1454, 1387, 1366, 1267, 1252, 1153, 1134, 1117, 1024, 1001, 991, 957, 939, 901, 862, 781, 748, 696, 604, 586, 476, 459, 446, 434, 417, 409, 401 cm^{-1}; HRMS (DART) *m/z* calc'd. for $C_{22}H_{20}O_3$ $(M + H)^+$ 333.1485, found 333.1488.

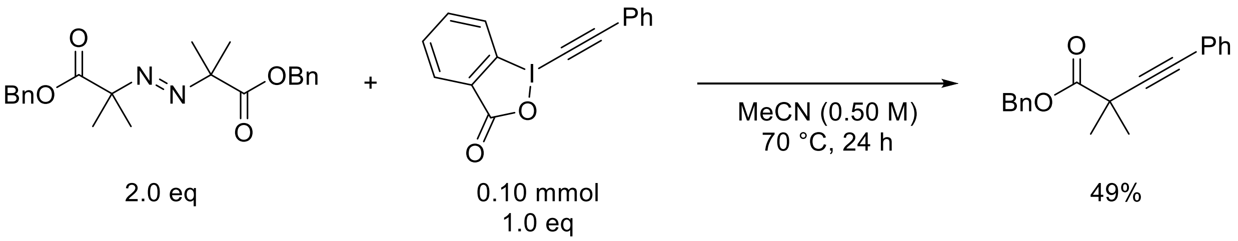

Condition: The solution with dibenzyl 2,2'-(diazene-1,2-diyl)(*E*)-bis(2-methylpropanoate) (76.5 mg, 0.20 mmol, 2.0 eq), 1-(2-Phenylethynyl)-1,2-benziodoxol-3(1*H*)-one (34.8 mg, 0.10 mmol, 1.0 eq) and MeCN (0.20 ml, 0.50 M) was heated under Ar atmosphere at 70 °C. After 24 h, the solution was cooled to room temperature, it was purified by flash column chromatography to afford the desired compound.

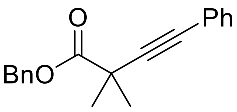

Benzyl 2,2-dimethyl-4-phenylbut-3-ynoate (16): (Colorless oil, *n*-Hexane/EtOAc = 100/1 to 50/1, 49% yield, 13.7 mg); ^{1}H NMR (500 MHz, $CDCl_3$) δ 7.40–7.27 (m, 10H, Ar*H*), 5.22 (s, 2H, OC*H₂*), 1.59 (s, 6H, C*CH₃*); ^{13}C NMR (125 MHz, $CDCl_3$) δ 173.6, 136.0, 131.7, 128.5, 128.2, 128.1, 128.0, 127.6, 123.2, 91.6, 81.9, 66.9, 38.8, 27.1; IR (neat) 2986, 1738, 1732, 1489, 1466, 1454, 1443, 1385, 1254, 1134, 1070, 1020, 912, 799, 756, 735, 692, 588, 556, 527, 486, 455, 442 cm^{-1}; HRMS (DART) *m/z* calc'd. for $C_{19}H_{18}O_2$ $(M + H)^+$ 279.1380, found 279.1378.

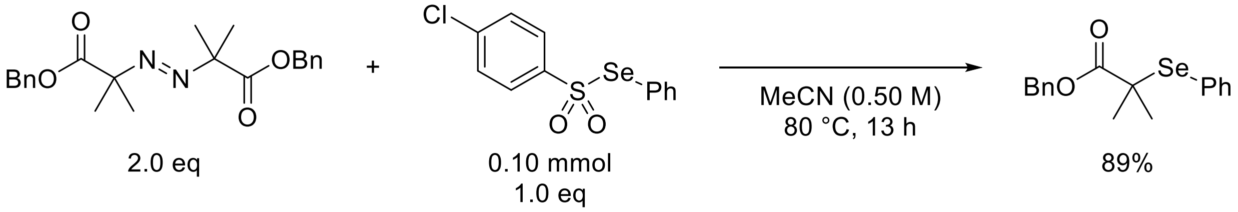

Condition: The solution with dibenzyl 2,2'-(diazene-1,2-diyl)(*E*)-bis(2-methylpropanoate) (76.5 mg, 0.20 mmol, 2.0 eq), *Se*-phenyl 4-chlorobenzenesulfonoselenoate (33.2 mg, 0.10 mmol, 1.0 eq) and MeCN (0.20 ml, 0.50 M) was heated under Ar atmosphere at 80 °C. After 13 h, the solution was cooled to room temperature, it was purified by flash column chromatography to afford the desired compound.

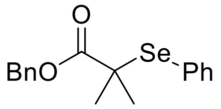

Benzyl 2-methyl-2-(phenylselanyl)propanoate (17): (Colorless oil, 1st: *n*-Hexane/EtOAc = 100/1 to 50/1, 2nd: *n*-Hexane/Et_2O = 100/1 to 50/1, 89% yield, 29.7 mg); ^1H NMR (500 MHz, $CDCl_3$) δ 7.48–7.46 (m, 2H, Ar*H*), 7.37–7.29 (m, 6H, Ar*H*), 7.25–7.22 (m, 2H, Ar*H*), 5.08 (s, 2H, O*CH₂*), 1.60 (s, 6H, C*CH₃*); ^{13}C NMR (125 MHz, $CDCl_3$) δ 174.4, 137.8, 135.9, 129.2, 128.7, 128.4, 128.1, 127.6, 66.7, 45.3, 26.3; IR (neat) 1717, 1497, 1476, 1454, 1437, 1385, 1366, 1258, 1213, 1144, 1111, 1082, 1065, 1022, 1009, 1001, 966, 905, 814, 739, 692, 673, 602, 584, 530, 517, 473, 459, 419, 405 cm^{-1}; HRMS (DART) *m/z* calc'd. for $C_{17}H_{18}O_2Se$ $(M + H)^+$ 335.0545, found 335.0553.

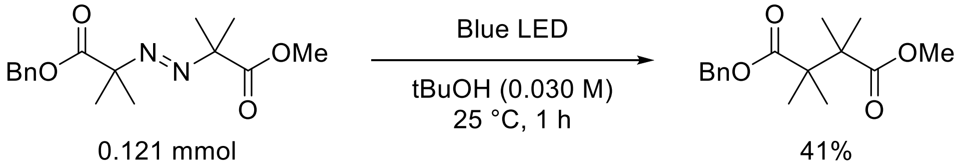

Condition[17]**:** The solution with benzyl (*E*)-2-((1-methoxy-2-methyl-1-oxopropan-2-yl)diazenyl)-2-methylpropanoate (37.0 mg, 0.121 mmol, 1.0 eq) and *t*BuOH (4.0 ml, 0.030 M) was stirred and irradiated using two 40 W blue LEDs (Kessil A160WE TUNA Blue, 2 cm away from the light sources) with a fan for 1 hour. After that, the solution was purified by flash column chromatography to afford the desired compound.

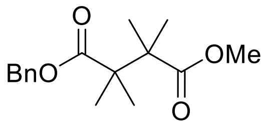

Benzyl (*E*)-2-((1-methoxy-2-methyl-1-oxopropan-2-yl)diazenyl)-2-methylpropanoate (18): (Colorless oil, *n*-Hexane/EtOAc = 100/1 to 50/1, 41% yield, 13.7 mg); ^1H NMR (500 MHz, $CDCl_3$) δ 7.38–7.30 (m, 5H, Ar*H*), 5.09 (s, 2H, OCH_2), 3.55 (s, 3H, OCH_3), 1.26 (s, 6H, CCH_3), 1.23 (s, 6H, CCH_3); ^{13}C NMR (125 MHz, $CDCl_3$) δ 176.5, 175.9, 136.0, 128.5, 128.1, 128.0, 66.5, 51.6, 47.6, 47.5, 22.0, 22.0; IR (neat) 2972, 2945, 1724, 1499, 1464, 1456, 1433, 1400, 1385, 1375, 1258, 1209, 1188, 1163, 1134, 1113, 1022, 1013, 914, 843, 826, 770, 737, 696, 602, 459 cm^{-1}; HRMS (DART) *m/z* calc'd. for $C_{16}H_{22}O_4$ $(M + H)^+$ 279.1591, found 279.1595.

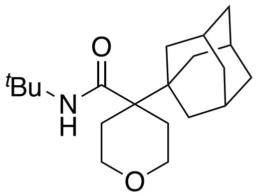

4-((1s,3s)-adamantan-1-yl)-N-(tert-butyl)tetrahydro-2H-pyran-4-carboxamide (19): (Changed condition : 0.060 mmol, White solid, *n*-Hexane/Et_2O = 50/1 to 20/1 to 10/1 to 3/1 to 1/1, 54% yield, 10.4 mg); ^1H NMR (500 MHz, CD_3OD) δ 6.26 (br, 1H, N*H*), 3.76–3.73 (m, 2H, OC*H₂*), 3.31 (t, *J* = 11.5 Hz, 2H, OC*H₂*), 1.89–1.87 (m, 5H, Ad*H,* CC*H₂*), 1.64–1.53 (m, 14H, Ad*H,* CC*H₂*), 1.30 (s, 9H, C*H₃*); ^{13}C NMR (125 MHz, CD_3OD) δ 172.7, 65.9, 51.8, 51.6, 36.8, 36.8, 36.2, 29.0, 27.8, 27.7; IR (neat) 2924, 2853, 1649, 1508, 1449, 1362, 1225, 1101, 1022, 856, 494, 451 cm^{-1}; HRMS (DART) *m/z* calc'd. for $C_{20}H_{34}NO_2$ $(M + H)^+$ 320.2584, found 320.2583.

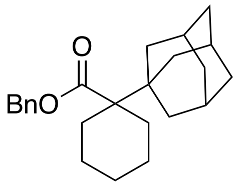

Benzyl 1-((1s,3s)-adamantan-1-yl)cyclohexane-1-carboxylate (20): (Changed condition : 0.060 mmol, White solid, *n*-Hexane/Et_2O = 100/1 to 50/1 to 10/1 to 5/1, 42% yield, 9.0 mg); ^1H NMR (500 MHz, Acetone-d_6) δ 7.46–7.44 (m, 2H, ArH), 7.40–7.37 (m, 2H, ArH), 7.35–7.31 (m, 1H, ArH), 5.16 (s, 2H, $ArCH_2$), 2.13–2.11 (m, 2H, $CH_2CH_2CH_2$), 1.92 (br, 3H, AdH), 1.66–1.52 (m, 15H, AdH, $CH_2CH_2CH_2$), 1.29–1.18 (m, 4H, $CH_2CH_2CH_2$), 1.13–1.05 (m, 1H, $CH_2CH_2CH_2$); ^{13}C NMR (125 MHz, Acetone-d_6) δ 174.3, 137.1, 129.0, 128.8, 128.3, 65.8, 54.6, 37.5, 37.4, 37.2, 29.0, 28.0, 26.0, 24.5; IR (neat) 2941, 2905, 1707, 1447, 1260, 1209, 1175, 1125, 1082, 1024, 802, 750, 694, 604 cm^{-1}; HRMS (DART) m/z calc'd. for $C_{24}H_{33}O_2$ $(M + H)^+$ 353.2475, found 353.2462.

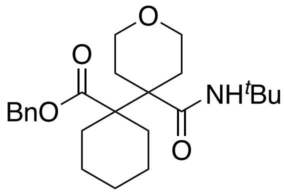

Benzyl 1-(4-(tert-butylcarbamoyl)tetrahydro-2H-pyran-4-yl)cyclohexane-1-carboxylate (21): (Changed condition : 0.060 mmol, Colorless oil, *n*-Hexane/Et_2O = 50/1 to 10/1 to 3/1 to 1/1, 27% yield, 6.5 mg); ^1H NMR (500 MHz, CD_3OD) δ 7.32–7.20 (m, 5H, Ar*H*), 6.36 (br, 1H, N*H*), 5.05 (s, 2H, ArC*H₂*), 3.71–3.68 (m, 2H, OC*H₂*), 3.28–3.24 (m, 2H, OC*H₂*), 2.10–2.07 (m, 2H, C*H₂*C*H₂*C*H₂*), 2.01–1.98 (m, 2H, C*H₂*C*H₂*C*H₂*), 1.60–1.52 (m, 2H,

$CH_2CH_2CH_2$), 1.46–1.44 (m, 1H, $CH_2CH_2CH_2$), 1.25 (s, 9H, CH_3), 1.22–1.19 (m, 2H, $CH_2CH_2CH_2$), 1.16–1.07 (m, 2H, $CH_2CH_2CH_2$), 1.02–0.93 (m, 1H, $CH_2CH_2CH_2$); ^{13}C NMR (125 MHz, CD_3OD) δ 173.6, 171.4, 135.9, 128.4, 128.1, 127.9, 66.2, 65.6, 53.1, 51.8, 51.2, 29.7, 29.2, 27.6, 25.2, 23.8; IR (neat) 2938, 2860, 1717, 1661, 1651, 1510, 1504, 1450, 1364, 1258, 1213, 1196, 1163, 1123, 1103, 1028, 970, 880, 737, 696, 478, 457 cm^{-1}; HRMS (DART) *m/z* calc'd. for $C_{24}H_{36}NO_4$ (M + H)$^+$ 402.2639, found 402.2632.

7. Mechanistic Study

7-1. Identification of 22 and 23

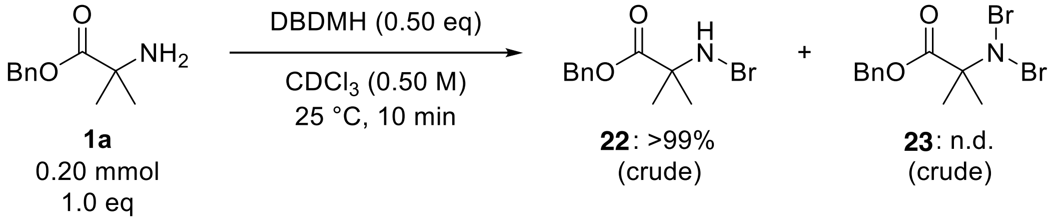

The solution with benzyl 2-amino-2-methylpropanoate (38.7 mg, 0.20 mmol, 1.0 eq.), 1,3-dibromo-5,5-dimethylhydantoin (28.6 mg, 0.10 mmol, 0.50 eq.), and $CDCl_3$ (0.40 ml, 0.50 M) was stirred under an argon atmosphere at 25 °C. After 10 min, the progress of the reaction was confirmed by measuring the crude by ^{1}H-NMR (The yield at this time was >99%.).

*The compound was easily broken during the purification process. Therefore, it was diluted with $CDCl_3$ without purification and measured by NMR. So, the isolation yield could not be determined.

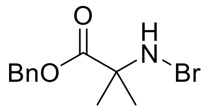

Crude data of **Benzyl 2-(bromoamino)-2-methylpropanoate (22):** ^{1}H NMR (500 MHz, $CDCl_3$) δ 7.40–7.33 (m, 5H, Ar*H*), 5.20 (s, 2H, OC*H*$_2$), 4.71 (br, 1H, N*H*), 1.43 (s, 6H, CC*H*$_3$); ^{13}C NMR (125 MHz, $CDCl_3$) δ 174.1, 135.4, 128.6, 128.4, 128.2, 67.3, 64.7, 24.2; HRMS (DART) *m/z* calc'd. for $C_{11}H_{14}NO_2Br$ $(M + H)^+$ 272.0281 (100%), 274.0261 (97%), found 272.0272, 274.0252.

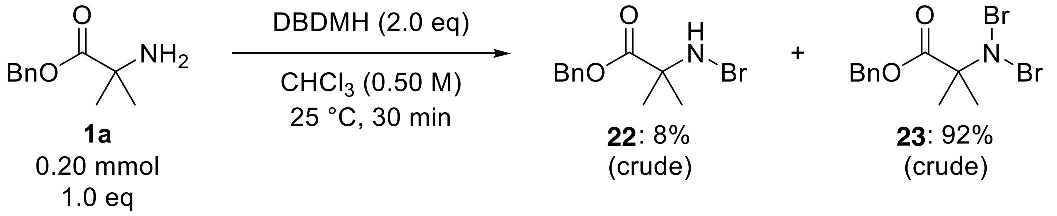

The solution with benzyl 2-amino-2-methylpropanoate (38.7 mg, 0.20 mmol, 1.0 eq.), 1,3-dibromo-5,5-dimethylhydantoin (114 mg, 0.40 mmol, 2.0 eq.), and $CHCl_3$ (0.40 ml, 0.50 M) was stirred under an argon atmosphere at 25 °C. After 30 min, the progress of the reaction was confirmed by measuring the crude by ^{1}H-NMR (The yield at this time was 92%.). It was purified by flash column chromatography to afford the desired compound.

*The compound was easily broken when concentrated. Therefore, it was diluted with $CDCl_3$ without vacuum drying after column and measured by NMR (>90% pure). So, the isolation yield could not be determined.

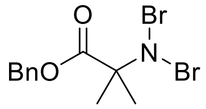

Crude data of **Benzyl 2-(dibromoamino)-2-methylpropanoate (23):** (orange oil, *n*-Hexane/EtOAc = 100/1 to 50/1); ^{1}H NMR (500 MHz, $CDCl_3$) δ 7.41–7.31 (m, 5H, Ar*H*), 5.21 (s, 2H, OC*H₂*), 1.61 (s, 6H, CC*H₃*); ^{13}C NMR (125 MHz, $CDCl_3$) δ 168.9, 135.2, 128.6, 128.4, 128.4, 79.3, 67.7, 24.2; HRMS (DART) *m/z* calc'd. for $C_{11}H_{13}NO_2Br_2$ $(M + H)^+$ 351.9366 (100%), 349.9386 (51%), 353.9345 (49%), found 351.9370, 349.9390, 353.9350.

7-2. Comparison of the timing of catalyst and base addition

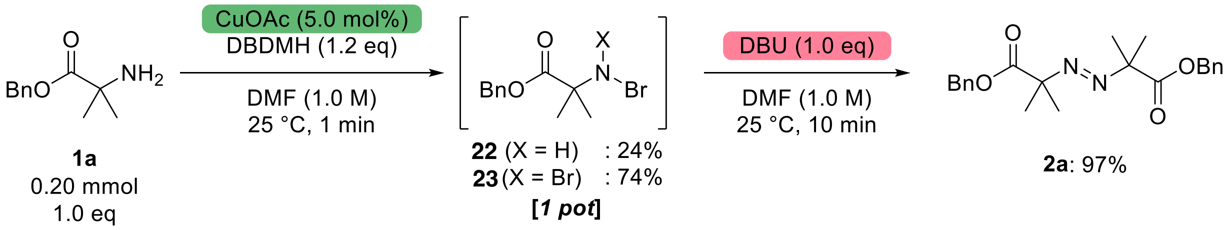

Condition: To a 4 ml vial equipped with a magnetic stirrer bar, CuOAc (1.2 mg, 10 µmol, 5.0 mol%) was added in a glove box followed by the addition of DMF (0.20 ml, 1.0 M), 1,3-dibromo-5,5-dimethylhydantoin (68.6 mg, 0.24 mmol, 1.2 eq) and benzyl 2-amino-2-methylpropanoate (41 µl, 0.20 mmol, 1.0 eq) under Ar atmosphere. The reaction mixture was stirred at 25 °C for 1 min. Then 1,8-diazabicyclo[5.4.0]undec-7-ene (30 µl, 0.20 mmol, 1.0 eq) was added to the crude under Ar atmosphere. The reaction mixture was stirred at 25 °C for 10 min. After that, the progress of the reaction was confirmed by measuring the crude by ^{1}H-NMR.

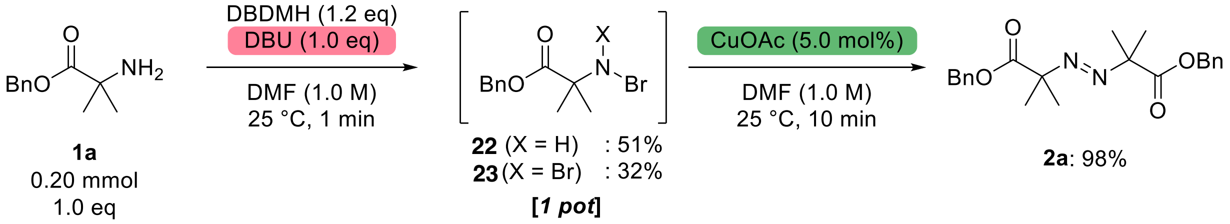

Condition: To a 4 ml vial equipped with a magnetic stirrer bar, 1,3-dibromo-5,5-dimethylhydantoin (68.6 mg, 0.24 mmol, 1.2 eq) was added followed by the addition of DMF (0.20 ml, 1.0 M), benzyl 2-amino-2-methylpropanoate (41 µl, 0.20 mmol, 1.0 eq) and 1,8-diazabicyclo[5.4.0]undec-7-ene (30 µl, 0.20 mmol, 1.0 eq) in a glove box under N_2 atmosphere. The reaction mixture was stirred at 25 °C for 1 min. Then CuOAc (1.2 mg, 10 µmol, 5.0 mol%) was added to the crude in glove box under N_2 atmosphere. The reaction mixture was stirred at 25 °C for 10 min. After that, the progress of the reaction was confirmed by measuring the crude by ^{1}H-NMR.

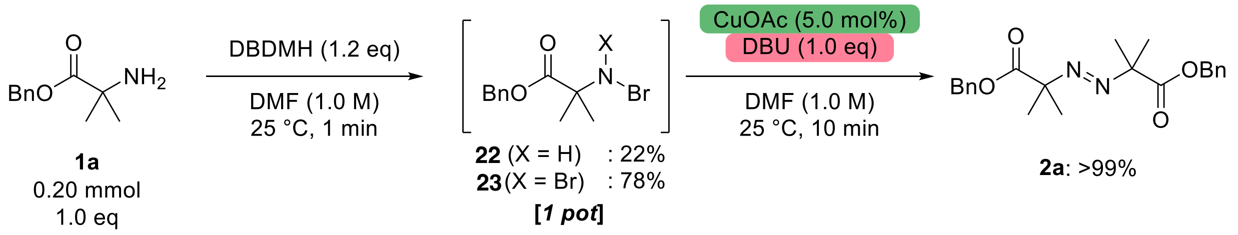

Condition: To a 4 ml vial equipped with a magnetic stirrer bar, 1,3-dibromo-5,5-dimethylhydantoin (68.6 mg, 0.24 mmol, 1.2 eq) was added followed by the addition of DMF (0.20 ml, 1.0 M) and benzyl 2-amino-2-methylpropanoate (41 µl, 0.20 mmol, 1.0 eq) in a glove box under N_2 atmosphere. The reaction mixture was stirred at 25 °C for 1 min. Then CuOAc (1.2 mg, 10 µmol, 5.0 mol%) and 1,8-diazabicyclo[5.4.0]undec-7-ene (30 µl, 0.20 mmol, 1.0 eq) were added to the crude in glove box under N_2 atmosphere. The reaction mixture was stirred at 25 °C for 10 min. After that, the progress of the reaction was confirmed by measuring the crude by ^{1}H-NMR.

7-3. Comparison of ^{1}H-NMR peaks

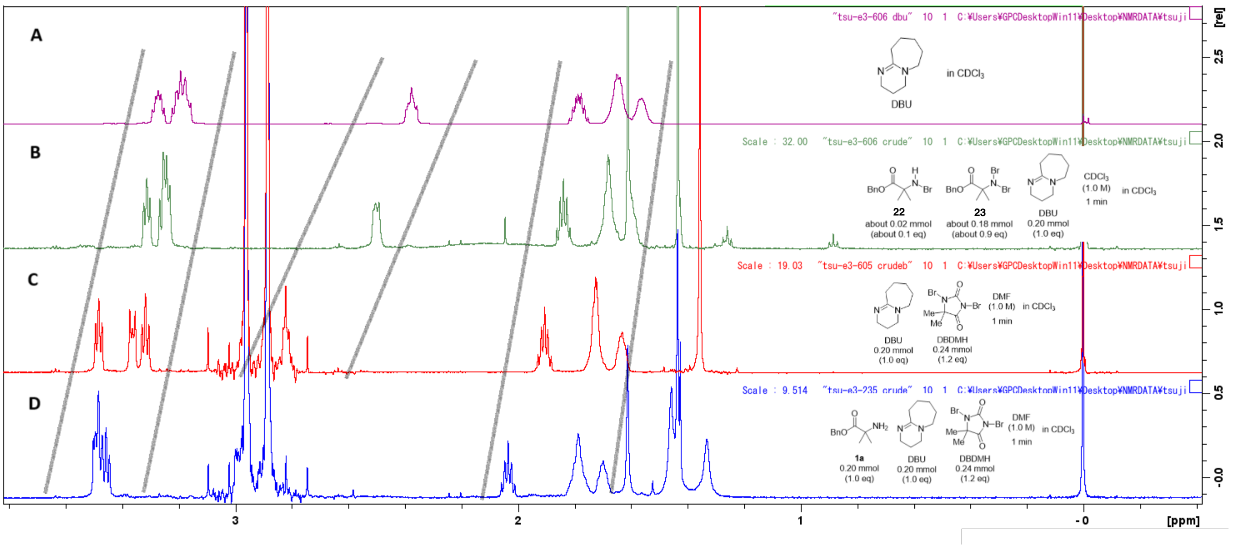

A…DBU

B…(**22** : **23** = 1 : 10) + DBU in $CDCl_3$ 1 min → (**22** : **23** = 1 : 1)

C…DBU + DBDMH in DMF 1 min

D…**1a** + DBU + DBDMH in DMF 1 min → (**22** : **23** = 3 : 2)

B: The solution with benzyl 2-amino-2-methylpropanoate (38.7 mg, 0.20 mmol, 1.0 eq.), 1,3-dibromo-5,5-dimethylhydantoin (114 mg, 0.40 mmol, 2.0 eq.), and $CHCl_3$ (0.40 ml, 0.50 M) was stirred under an argon

atmosphere at 25 °C. After 30 min, it was purified by short pad column to afford the desired compounds (**22** : **23** = 1 : 10) and diluted with $CDCl_3$ (0.20 ml). 1,8-Diazabicyclo[5.4.0]undec-7-ene (30 µl, 0.20 mmol, 1.0 eq) was added to the solution. The reaction mixture was stirred at 25 °C for 1 min and measuring the crude by ^{1}H-NMR.

C: To a 4 ml vial equipped with a magnetic stirrer bar, 1,3-dibromo-5,5-dimethylhydantoin (68.6 mg, 0.24 mmol, 1.2 eq) was added followed by the addition of DMF (0.20 ml, 1.0 M) and 1,8-diazabicyclo[5.4.0]undec-7-ene (30 µl, 0.20 mmol, 1.0 eq). The reaction mixture was stirred at 25 °C for 1 min and measuring the crude by ^{1}H-NMR.

D: To a 4 ml vial equipped with a magnetic stirrer bar, 1,3-dibromo-5,5-dimethylhydantoin (68.6 mg, 0.24 mmol, 1.2 eq) was added followed by the addition of DMF (0.20 ml, 1.0 M), benzyl 2-amino-2-methylpropanoate (41 µl, 0.20 mmol, 1.0 eq) and 1,8-diazabicyclo[5.4.0]undec-7-ene (30 µl, 0.20 mmol, 1.0 eq). The reaction mixture was stirred at 25 °C for 1 min and measuring the crude by ^{1}H-NMR.

From these analyses, peak shifts were observed for DBU, but no peak shift was observed for **22** and **23**. However, the addition of DBU biased the ratio of **22** to **23** toward **22**, indicating that the DBU activates the N–Br bonds via halogen interaction and the DBU becomes some ammonium salts.

8. Optimization Study

8-1. Screening of solvent

Table S2. Solvent screening

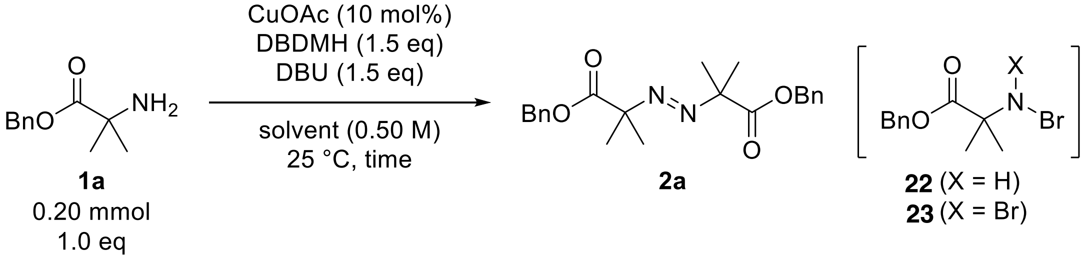

entry	solvent	time	**2a** (%)	**1a** (%)	**22** (%)	**23** (%)
1	PhCl	30 min	41	n.d.	16	41
2	DCE		96	n.d.	n.d.	n.d.
3	toluene		4	n.d.	55	10
4	THF		58	n.d.	n.d.	n.d.
5	MeCN		>99	n.d.	n.d.	n.d.
6	EtOAc		7	n.d.	62	22
7	DMF		>99	n.d.	n.d.	n.d.
8	DMSO		9	n.d.	9	4
9	MeOH		11	n.d.	5	19
10	HFIP		n.d.	n.d.	29	79
11	DCE	10 min	65	n.d.	7	31
12	MeCN		>99	n.d.	n.d.	n.d.
13	DMF		>99	n.d.	n.d.	n.d.
14	MeCN	1 min	>99	n.d.	n.d.	n.d.
15	DMF		>99	n.d.	n.d.	n.d.

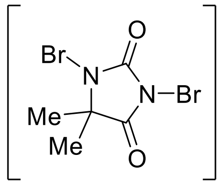

DBDMH

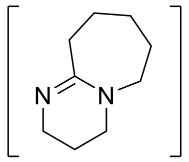

DBU

Yields were determined by ^{1}H NMR spectroscopic analysis.

8-2. Screening of oxidant

Table S3. Oxidant screening

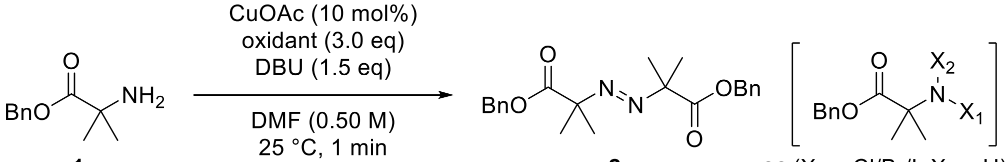

1a
0.20 mmol
1.0 eq

2a

22 (X_1 = Cl/Br/I, X_2 = H)
23 (X_1 = X_2 = Cl/Br/I)

entry	oxidant	**2a** (%)	**1a** (%)	**22** (%)	**23** (%)
1	NCS	81	n.d.	2	21
2	NBS	>99	n.d.	n.d.	n.d.
3	NIS	3	n.d.	n.d.	n.d.
4	NCP	29	n.d.	n.d.	70
5	NBP	99	n.d.	n.d.	n.d.
6	NIP	5	n.d.	n.d.	n.d.
7	DCDMH (1.5 eq)	33	n.d.	74	n.d.
8	DBDMH (1.5 eq)	>99	n.d.	n.d.	n.d.
9	DIDMH (1.5 eq)	4	n.d.	n.d.	n.d.
10	O_2 (1 atm)	n.d.	99 (98[a])	---	---
11	oxone	n.d.	99	---	---
12	$K_2S_2O_8$	n.d.	>99	---	---
13	CAN	n.d.	n.d.	---	---
14	$KMnO_4$	n.d.	99	---	---
15	TEMPO	n.d.	99	---	---
16	DTBP	n.d.	>99	---	---
17	TBHP[b]	n.d.	87	---	---
18	H_2O_2[c]	n.d.	90	---	---

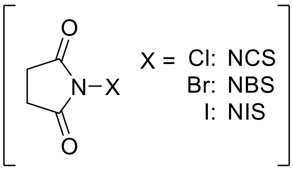

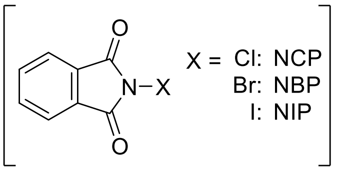

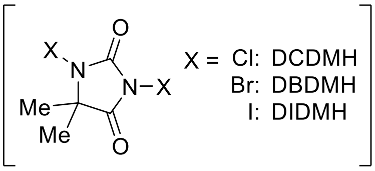

Yields were determined by ^{1}H NMR spectroscopic analysis.
[a] 3 h instead of 1 min. [b] 5.5 M in decane. [c] 35% in H_2O.

8-3. Screening of catalyst

Table S4. Catalyst screening

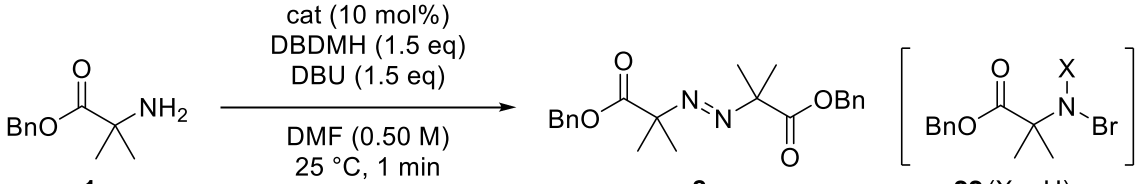

1a 0.20 mmol 1.0 eq

2a

22 (X = H)
23 (X = Br)

entry	cat	**2a** (%)	**22** (%)	**23** (%)
1	none	7	43	51
2	CuOAc	>99	n.d.	n.d.
3	$Cu(OAc)_2$	22	43	40
4	$Cu(OTf)_2$	29	39	33
5	$Cu(acac)_2$	37	20	n.d.
6	CuCl	61	12	29
7	$CuCl_2$	87	4	11
8	CuBr	68	7	19
9	$CuBr_2$	37	28	38
10	CuI	37	33	28
11	$Fe(OAc)_2$	21	13	66
12	AgOAc	35	28	41

Yields were determined by ^{1}H NMR spectroscopic analysis.

8-4. Screening of base

Table S5. Base screening

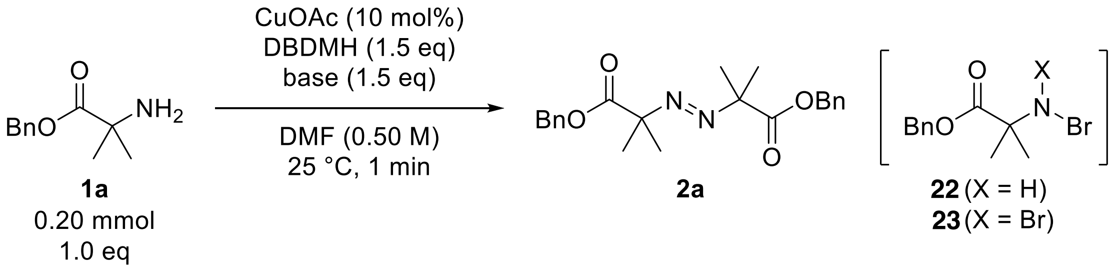

entry	base	**2a** (%)	**1a** (%)	**22** (%)	**23** (%)
1	DBU	>99	n.d.	n.d.	n.d.
2	DABCO	60	n.d.		n.d.
3	NEt_3	5	n.d.	---	---
4	NMM	3	n.d.	n.d.	n.d.
5	K_2CO_3	30	n.d.	12	64
6	KO*t*Bu	25	n.d.	55	21

Yields were determined by ^{1}H NMR spectroscopic analysis.

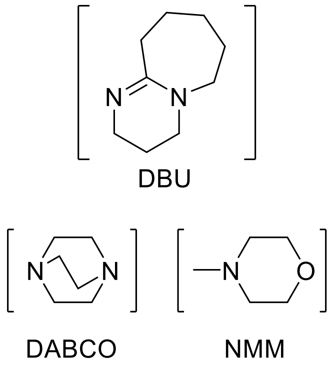

8-5. Screening of equivalent

Table S6. Equivalent screening

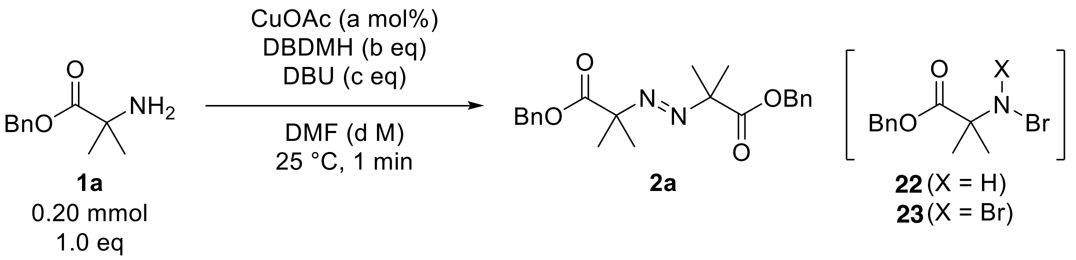

entry	a (mol%)	b (eq)	c (eq)	d (M)	**2a** (%)	**1a** (%)	**22** (%)	**23** (%)
1	10	1.5	1.5	0.50	>99	n.d.	n.d.	n.d.
2	10	1.5	1.0	0.50	>99	n.d.	n.d.	n.d.
3	10	1.5	0.5	0.50	85	n.d.	3	15
4	10	1.5	0.17	0.50	34	n.d.	10	57
5	10	1.2	1.0	0.50	>99	n.d.	n.d.	n.d.
6	10	1.0	1.0	0.50	91	n.d.	n.d.	n.d.
7	10	0.5	1.0	0.50	30	n.d.	n.d.	11
8	5.0	1.2	1.0	1.0	99	n.d.	n.d.	n.d.
9	2.0	1.2	1.0	1.0	21	n.d.	40	39

Yields were determined by ^{1}H NMR spectroscopic analysis.

9. DFT calculation

9-1. General computational details

The DFT calculations were performed using Gaussian 16, revision A.03.[18] Geometry optimizations were performed using the B3LYP functional[19–21] with the 6-31+G(d,p) basis set for C, H, N, O, Cu atoms and the Lanl2dz effective core potential (ECP) for Br.[22,23] After optimization of the structures, frequency calculations were performed at the same level of theory to confirm that the obtained structures were either a stationary point (no imaginary frequencies) or a transition state (one imaginary frequency). IRC calculations were performed for each transition state structure to confirm that the transition state connected the reaction pathway between the starting materials and the products or intermediates. Thermal corrections to the Gibbs energy at 298.15 K were obtained from the frequency calculations. Single-point energy calculations for the optimized geometry were performed using the M06 functional[24] with the 6-311+G(d,p) basis set for C, H, N, O, Cu atoms and the SDD effective core potential (ECP) for Br[22,23] and the SMD solvation model[25] (DMF).

9-2. Calculated reaction pathway

(1-1) Calculated pathway for the generation of N-H aminyl radical

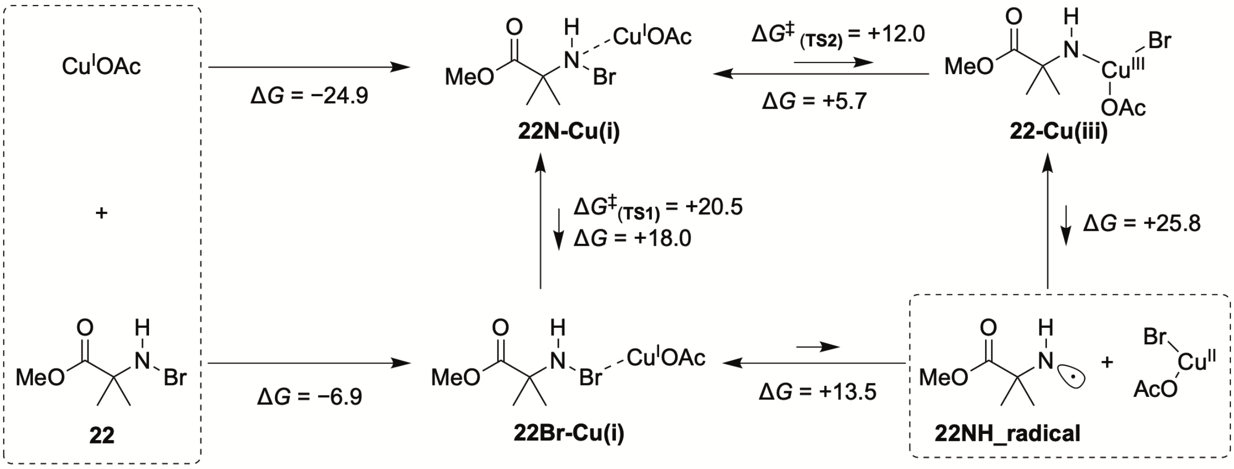

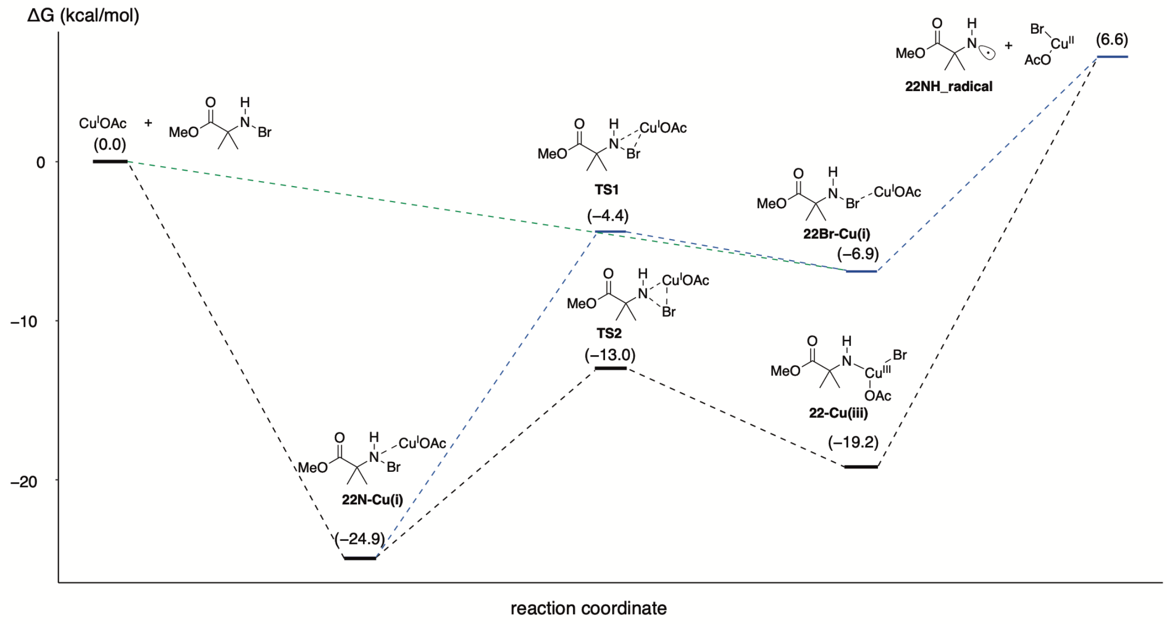

sp M06/SDD(Br)-6-311+G(d,p)/SMD(DMF)//B3LYP/Lanl2dz(Br)-6-31+G(d,p) at 1 atm, 298.15 K.
The values of Gibbs free energy are given in kcal/mol.

Figure S2. Computational pathway for the generation of the N–H aminyl radical

(1-2) Calculated pathway for the generation of N-Br aminyl radical

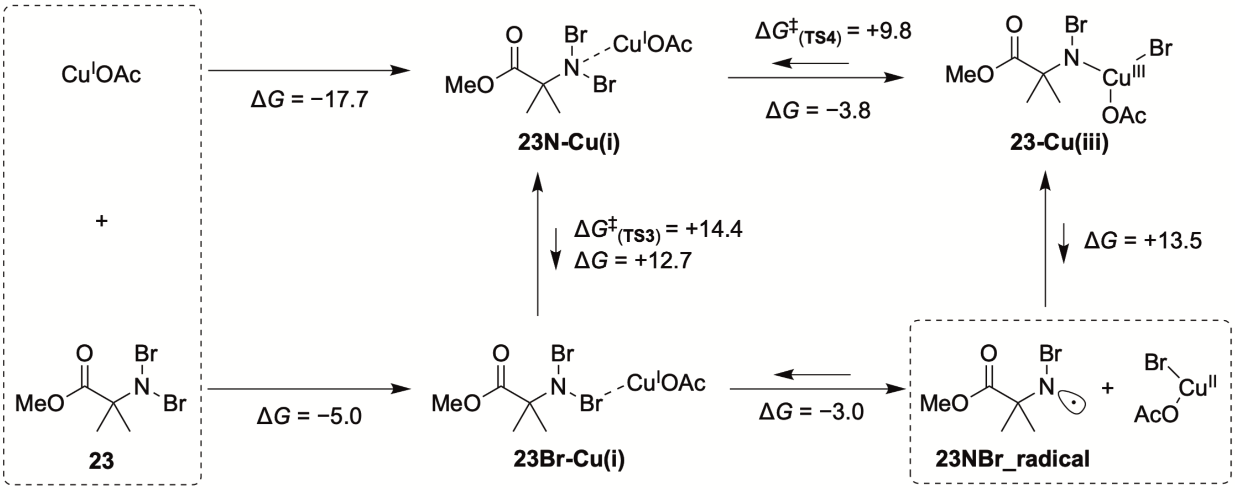

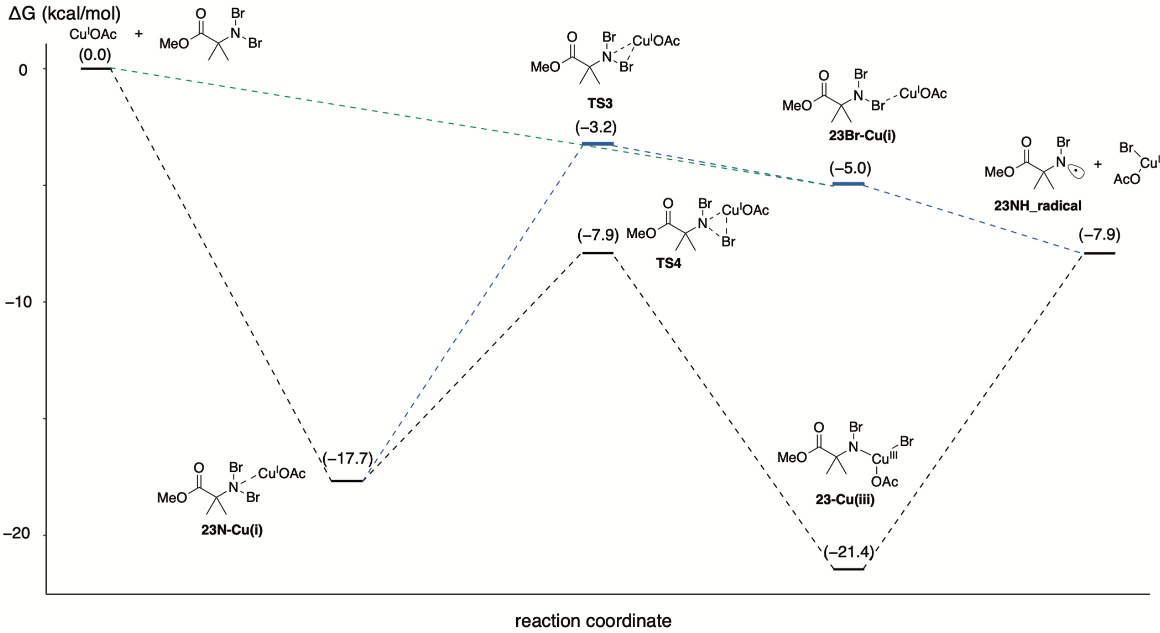

sp M06/SDD(Br)-6-311+G(d,p)/SMD(DMF)//B3LYP/Lanl2dz(Br)-6-31+G(d,p) at 1 atm, 298.15 K.
The values of Gibbs free energy are given in kcal/mol.

Figure S3. Computational pathway for the generation of the N–Br aminyl radical

(2)

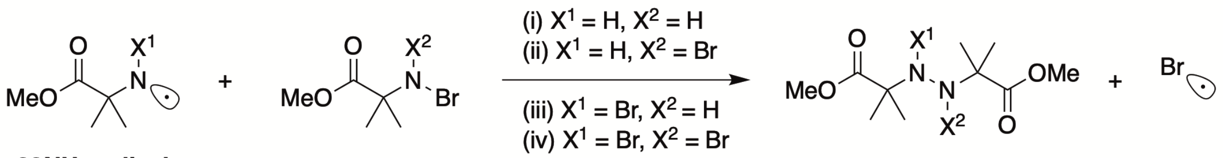

22NH_radical or **23NBr_radical** (i)-1–(iv)-1

22 or **23**

NH-NH or **NHNBr** or **NBr-NBr** (i)-2–(iv)-2

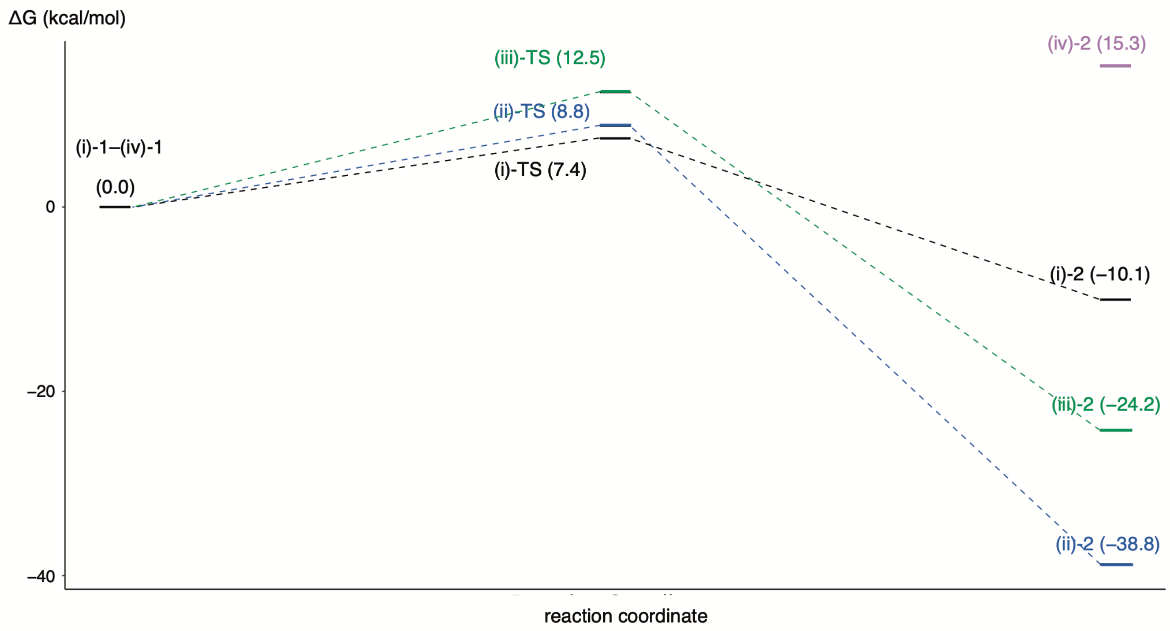

(3-1)

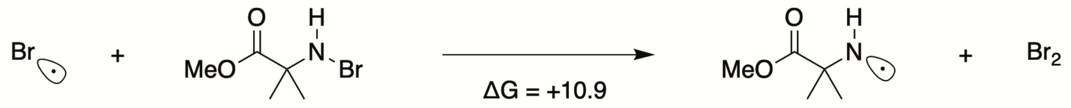

22 **22NH_radical**

(3-2)

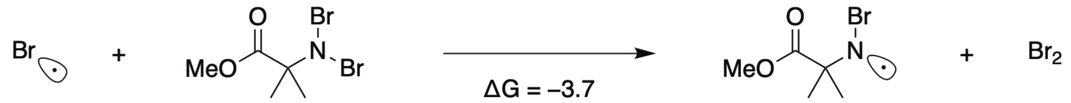

23 **23NBr_radical**

sp UM06/SDD(Br)-6-311+G(d,p)/SMD(DMF)//UB3LYP/Lanl2dz(Br)-6-31+G(d,p) at 1 atm, 298.15 K.
The values of Gibbs free energy are given in kcal/mol.

Figure S4. Computational pathway for N–N bond formation

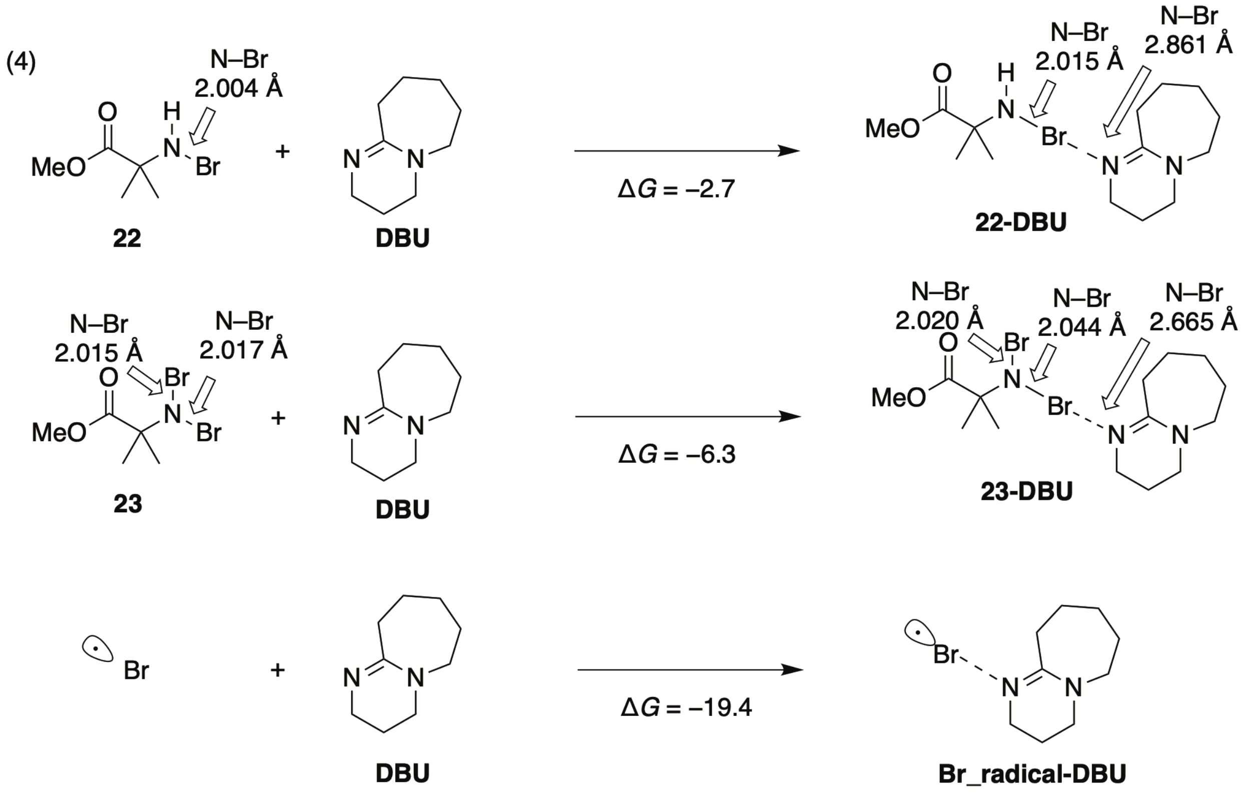

sp M06/SDD(Br)-6-311+G(d,p)/SMD(DMF)//B3LYP/Lanl2dz(Br)-6-31+G(d,p) at 1 atm, 298.15 K.
The values of Gibbs free energy are given in kcal/mol.

Figure S5. Computational analysis of the thermodynamic stability of DBU complexes

9-3. Detailed information for calculated structures

CuOAc

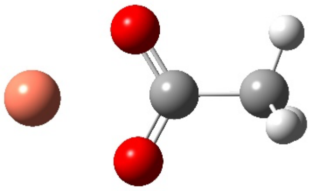

RB3LYP/6-31+G(d,p) free energy: -1868.839995 (a.u.)

RB3LYP/6-31+G(d,p) thermal correction to Gibbs free energy: 0.017661 (a.u.)

Number of imaginary frequencies: 0

RM06/6-311+G(d,p)/SMD(DMF) single point energy: -1868.873278 (a.u.)

C	1.07297100	0.00882800	-0.00159600
O	0.44421800	1.11707400	-0.00071700
O	0.44626700	-1.10303200	-0.00076700
C	2.58545100	-0.00158100	0.00030400
H	2.93945500	-0.50777200	0.90363100
H	2.94473500	-0.57776100	-0.85732800
H	2.98265000	1.01286400	-0.03627300
Cu	-1.30831900	-0.00286700	0.00033100

CuBrOAc

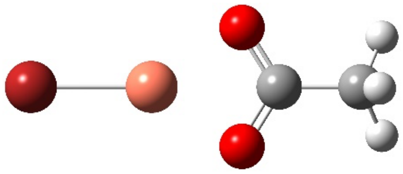

UB3LYP/Lanl2dz(Br)-6-31+G(d,p) free energy: -1882.060098 (a.u.)

UB3LYP/Lanl2dz(Br)-6-31+G(d,p) thermal correction to Gibbs free energy: 0.015292 (a.u.)

Number of imaginary frequencies: 0

UM06/SDD(Br)-6-311+G(d,p)/SMD(DMF) single point energy: -1882.248145 (a.u.)

C	2.36942200	0.00029100	-0.01086600
O	1.69101800	1.08477000	-0.01067500
O	1.69100200	-1.08427900	-0.01070400
C	3.86445600	-0.00017300	0.01455400
H	4.19492500	-0.02363600	1.05927000

H	4.25056300	-0.88968800	-0.48690400
H	4.25143300	0.90899800	-0.44917000
Cu	0.03696800	0.00013200	-0.00456300
Br	-2.23509800	-0.00011800	0.00451500

22

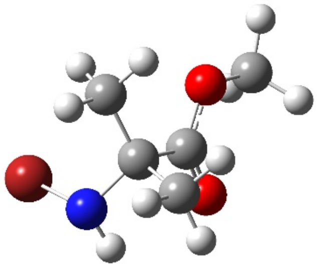

RB3LYP/Lanl2dz(Br)-6-31+G(d,p) free energy: -414.802771 (a.u.)

RB3LYP/Lanl2dz(Br)-6-31+G(d,p) thermal correction to Gibbs free energy: 0.115403 (a.u.)

Number of imaginary frequencies: 0

RM06/SDD(Br)-6-311+G(d,p)/SMD(DMF) single point energy: -414.950439 (a.u.)

C	-1.28564500	-0.15812200	-0.33870000
O	-1.23716300	-0.68710900	-1.43256800
O	-2.14217700	-0.52373800	0.62846200
C	-0.40305500	1.05005900	0.04467600
C	-1.04373300	2.29343900	-0.62798200
H	-2.05494700	2.44604000	-0.23737900
H	-0.43938700	3.17617200	-0.40521400
H	-1.10734300	2.16852500	-1.71298300
C	-0.27160600	1.27579000	1.55307500
H	0.11776000	0.39162000	2.06176000
H	0.41652300	2.10808500	1.72587900
H	-1.24325100	1.51848800	1.98833200
N	0.92105800	0.95455400	-0.59595600
H	0.75825900	0.65403900	-1.56121400
Br	2.00244900	-0.59672100	0.06827000
C	-3.02972900	-1.61700500	0.30747900
H	-3.63081900	-1.77256200	1.20267200
H	-3.66095500	-1.35353800	-0.54447200

H	-2.45162000	-2.51171900	0.06641600

23

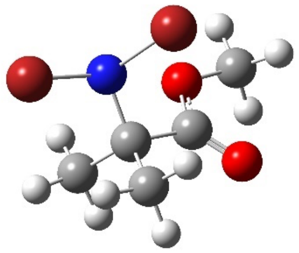

RB3LYP/Lanl2dz(Br)-6-31+G(d,p) free energy: -427.333974 (a.u.)

RB3LYP/Lanl2dz(Br)-6-31+G(d,p) thermal correction to Gibbs free energy: 0.101045 (a.u.)

Number of imaginary frequencies: 0

RM06/SDD(Br)-6-311+G(d,p)/SMD(DMF) single point energy: -427.656278 (a.u.)

C	1.91023300	-0.54578600	0.26401400
O	2.69143800	-0.46529300	1.18738900
O	2.23791500	-0.35069000	-1.02030100
C	0.42644600	-0.98252800	0.43149800
C	0.30181300	-2.38790300	-0.20651300
H	1.07345500	-3.03796900	0.21861800
H	-0.67353000	-2.82449900	0.01642200
H	0.43907300	-2.33618100	-1.28834500
C	0.06470700	-1.01355300	1.91805400
H	0.19605600	-0.03356500	2.38031000
H	-0.97216500	-1.33463700	2.04066000
H	0.71678200	-1.71813200	2.43866300
C	3.60395300	0.03926400	-1.27127200
H	3.66880900	0.17613200	-2.34998800
H	3.83137500	0.97037100	-0.74715700
H	4.28937600	-0.74290900	-0.93594000
N	-0.36199200	-0.05620300	-0.44775100
Br	-2.32906600	-0.49850000	-0.38351700
Br	-0.16559100	1.84552000	0.18950700

22N-Cu(i)

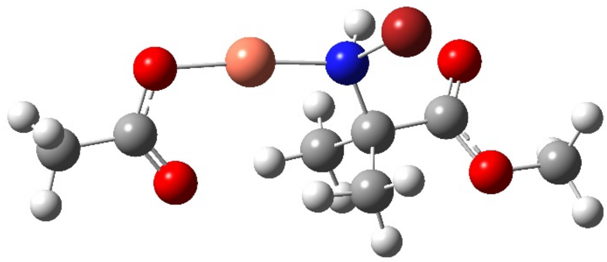

RB3LYP/Lanl2dz(Br)-6-31+G(d,p) free energy: -2283.672085 (a.u.)

RB3LYP/Lanl2dz(Br)-6-31+G(d,p) thermal correction to Gibbs free energy: 0.155753 (a.u.)

Number of imaginary frequencies: 0

RM06/SDD(Br)-6-311+G(d,p)/SMD(DMF) single point energy: -2283.863470 (a.u.)

C	-3.75974400	-0.71261800	0.15171700
O	-2.96691300	-1.59535700	0.52388000
O	-3.38388800	0.43127400	-0.35296200
C	-5.26322600	-0.89829600	0.25687700
H	-5.69043400	-0.10397100	0.87749500
H	-5.71637700	-0.80956900	-0.73582200
H	-5.49896800	-1.87250500	0.68692200
Cu	-1.52498400	0.49335500	-0.40453900
C	2.48685200	-0.86712400	-0.34522300
O	2.86231000	-0.38085700	-1.39590100
O	3.28037300	-1.51480900	0.51221000
C	1.00650800	-0.86198200	0.08245500
C	0.32828500	-2.04955500	-0.65409000
H	0.82569400	-2.97695500	-0.35200100
H	-0.72805400	-2.10809300	-0.37327900
H	0.41928900	-1.94937500	-1.73997200
C	0.76698800	-0.96571100	1.58840200
H	1.25334500	-0.15540300	2.13560600
H	-0.30889400	-0.94373000	1.78546000
H	1.16553600	-1.91300600	1.95596100
N	0.37826900	0.37279600	-0.49511800
H	0.75835800	0.46571400	-1.44350100
Br	1.15236900	2.08199300	0.30596500
C	4.67051900	-1.63407300	0.12840200

H	5.14551300	-2.18101200	0.94146300
H	4.75391000	-2.18223300	-0.81254800
H	5.11267800	-0.64235100	0.01383200

22Br-Cu(i)

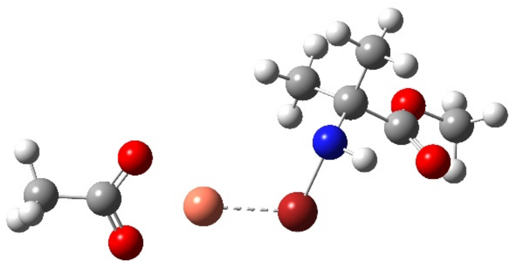

RB3LYP/Lanl2dz(Br)-6-31+G(d,p) free energy: -2283.651826 (a.u.)

RB3LYP/Lanl2dz(Br)-6-31+G(d,p) thermal correction to Gibbs free energy: 0.151262 (a.u.)

Number of imaginary frequencies: 0

RM06/SDD(Br)-6-311+G(d,p)/SMD(DMF) single point energy: -2283.834716 (a.u.)

C	-3.08353600	0.29742200	0.02268000
O	-3.61832000	-0.71998500	-0.37758400
O	-3.57697900	1.06720700	1.00130200
C	-1.79089200	0.86706800	-0.59785200
C	-2.22376400	1.82749400	-1.74473600
H	-2.83082400	2.63525500	-1.32488500
H	-1.33323100	2.25275300	-2.21253100
H	-2.81387700	1.30636400	-2.50475800
C	-0.89779000	1.60937200	0.40140300
H	-0.68344700	0.99804300	1.28182000
H	0.04977100	1.87373500	-0.07616400
H	-1.39549800	2.51874800	0.74372000
N	-1.05355700	-0.17858400	-1.32331200
H	-1.75748000	-0.76959700	-1.78107500
Br	-0.24784900	-1.60861000	-0.08598400
C	-4.82448100	0.63197200	1.58850600
H	-5.05679400	1.37390400	2.35127200
H	-5.60697300	0.59689000	0.82715800
H	-4.70472700	-0.35850300	2.03258000

C	3.77275000	0.90133500	0.19268300
O	2.70694000	1.41080500	-0.25138000
O	3.81088900	-0.33124800	0.55887400
C	5.04325300	1.71578900	0.30690900
H	5.41674700	1.67347600	1.33463300
H	5.81365900	1.27710700	-0.33554400
H	4.86687400	2.75167300	0.01473700
Cu	1.91489400	-0.65846100	0.11911700

23N-Cu(i)

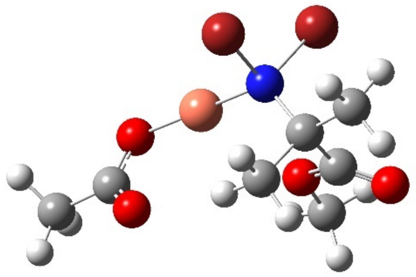

RB3LYP/Lanl2dz(Br)-6-31+G(d,p) free energy: -2296.196028 (a.u.)

RB3LYP/Lanl2dz(Br)-6-31+G(d,p) thermal correction to Gibbs free energy: 0.140206 (a.u.)

Number of imaginary frequencies: 0

RM06/SDD(Br)-6-311+G(d,p)/SMD(DMF) single point energy: -2296.557721 (a.u.)

C	-0.60594600	2.11214800	0.68201400
O	-1.18739300	3.12338000	1.00567500
O	0.51930700	2.07997000	-0.03723500
C	-1.04819600	0.69903200	1.16741800
C	-0.11892000	0.32176800	2.34697400
H	-0.20840400	1.10515600	3.10646800
H	-0.44109600	-0.62116600	2.79411000
H	0.92916800	0.24220900	2.04183300
C	-2.51205700	0.74835400	1.60589400
H	-3.17673000	1.00603900	0.77901000
H	-2.81469800	-0.21104000	2.02879400
H	-2.62017700	1.51902600	2.37030600
C	1.07401900	3.35309300	-0.44485600
H	1.96955500	3.10155800	-1.01024700

H	0.35329800	3.89174700	-1.06346200
H	1.32316100	3.94608300	0.43716700
N	-0.75711100	-0.27944700	0.03598800
Br	-1.38168500	-2.16018700	0.54586300
Br	-1.85013900	0.23064700	-1.61545300
C	3.61440300	-0.45364900	0.08841400
O	3.17509700	-0.08635700	1.18978600
O	2.86070800	-0.71958100	-0.94469700
C	5.10397900	-0.63514400	-0.15283000
H	5.31171900	-1.68789000	-0.37198900
H	5.41581800	-0.05718000	-1.02841500
H	5.67324700	-0.32495000	0.72436700
Cu	1.05908000	-0.49905100	-0.52036500

23Br-Cu(i)

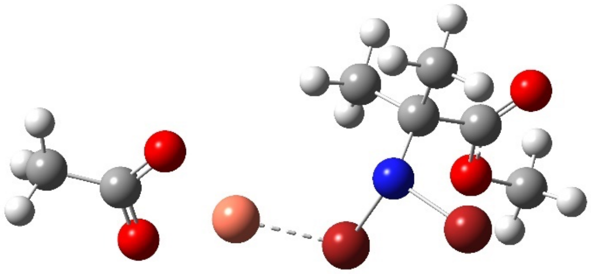

RB3LYP/Lanl2dz(Br)-6-31+G(d,p) free energy: -2296.183299 (a.u.)

RB3LYP/Lanl2dz(Br)-6-31+G(d,p) thermal correction to Gibbs free energy: 0.136702 (a.u.)

Number of imaginary frequencies: 0

RM06/SDD(Br)-6-311+G(d,p)/SMD(DMF) single point energy: -2296.537453 (a.u.)

C	2.64003900	1.06964500	-0.31284500
O	2.81850200	0.39610200	-1.30310500
O	3.40050800	2.12917200	0.02104400
C	1.49096100	0.87779000	0.70027500
C	1.99810100	0.97910800	2.16509900
H	2.35472900	1.99620000	2.33820800
H	1.17171300	0.76588600	2.84733200
H	2.81431900	0.28368900	2.36420300
C	0.43466300	1.98752700	0.46619900

H	0.13093000	2.04681500	-0.58069900
H	-0.44817600	1.79611600	1.08247600
H	0.86913500	2.94800500	0.75316300
N	0.78219200	-0.42177700	0.62166700
Br	-0.19188100	-0.71322000	-1.26686600
C	4.47199400	2.45011900	-0.89388900
H	4.96797100	3.31853600	-0.46219300
H	5.16253500	1.60792500	-0.97522500
H	4.06726100	2.68403400	-1.88119000
C	-4.34502700	0.60914500	0.51351100
O	-3.26611900	0.74597700	1.16965400
O	-4.31918500	0.07384800	-0.64875100
C	-5.66711900	1.05333600	1.09259300
H	-6.23930300	1.60295200	0.34044400
H	-6.25031900	0.16682800	1.36476600
H	-5.51427700	1.66747400	1.98107700
Cu	-2.32505800	-0.17195600	-0.49998000
Br	2.01055200	-1.96866800	0.82912200

22-Cu(iii)

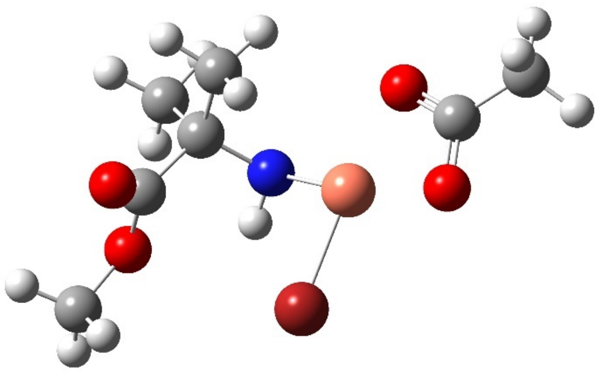

RB3LYP/Lanl2dz(Br)-6-31+G(d,p) free energy: -2283.675224 (a.u.)

RB3LYP/Lanl2dz(Br)-6-31+G(d,p) thermal correction to Gibbs free energy: 0.154273 (a.u.)

Number of imaginary frequencies: 0

RM06/SDD(Br)-6-311+G(d,p)/SMD(DMF) single point energy: -2283.854315 (a.u.)

C	3.35373200	0.46904400	0.06975400
O	2.49535600	1.37656800	-0.16897900
O	2.92226500	-0.73715800	0.12264500

C	4.80537400	0.77432300	0.28962100
H	5.07612500	0.50855300	1.31666700
H	5.41487600	0.16003600	-0.37957900
H	5.00566600	1.83281100	0.11835900
Cu	1.08974500	-0.14981000	-0.26778100
Br	-0.19384300	-2.06480400	0.07401400
N	-0.39012200	0.73171200	-0.94458500
C	-1.25406800	1.51940200	-0.06526200
C	-1.79286600	2.67372200	-0.97001400
H	-0.95417200	3.29423700	-1.29340400
H	-2.30986400	2.28066500	-1.84829700
H	-2.49345300	3.28573900	-0.39147500
C	-0.47729100	2.10245900	1.11931700
H	-0.15242200	1.31133500	1.80228400
H	0.39923300	2.64266000	0.75518400
H	-1.11406200	2.78108900	1.69074200
C	-2.47697100	0.71932300	0.44251200
O	-2.86875900	0.70703600	1.58896800
O	-3.08323700	0.06385800	-0.56728200
C	-4.20384200	-0.76968600	-0.20478300
H	-4.56053700	-1.19850400	-1.14057000
H	-3.87559400	-1.55437300	0.48023900
H	-4.98241400	-0.16901200	0.27090900
H	-0.99003800	0.10144200	-1.48347500

23-Cu(iii)

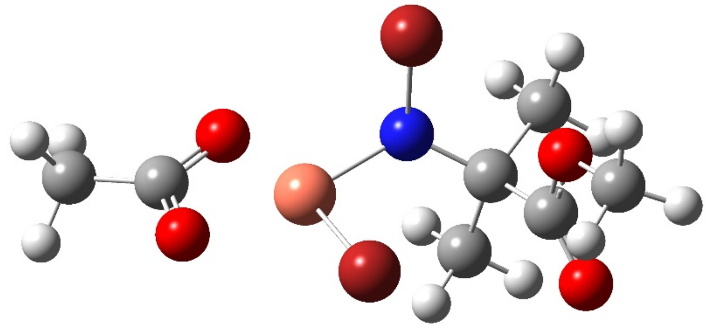

RB3LYP/Lanl2dz(Br)-6-31+G(d,p) free energy: -2296.207521 (a.u.)

RB3LYP/Lanl2dz(Br)-6-31+G(d,p) thermal correction to Gibbs free energy: 0.140338 (a.u.)

Number of imaginary frequencies: 0

RM06/SDD(Br)-6-311+G(d,p)/SMD(DMF) single point energy: -2296.563744 (a.u.)

C	-3.57019400	-0.19114200	0.25795400
O	-2.77268600	-1.14954800	0.48719800
O	-3.04086200	0.94196800	-0.04149900
C	-5.06030500	-0.34135500	0.31532800
H	-5.48690100	0.44532100	0.94421500
H	-5.47158500	-0.21696400	-0.69187200
H	-5.32976500	-1.32529900	0.70178400
Cu	-1.23362200	0.22375100	0.09721200
Br	-0.01411400	2.09389300	-0.58929500
N	0.20106800	-0.91656200	0.45154600
C	1.36245900	-0.62171100	1.30196600
C	2.05270200	-1.96718300	1.66383700
H	1.31277000	-2.65889000	2.07460400
H	2.52360400	-2.42015000	0.79003000
H	2.81855600	-1.77444700	2.42080200
C	0.75036200	0.00303700	2.57342300
H	0.29379500	0.97177800	2.35593000
H	0.00208200	-0.67483000	2.99124100
H	1.54017500	0.17159900	3.30808000
C	2.46727600	0.33007800	0.76104000
O	2.95586300	1.21413300	1.43174800
O	2.87939400	0.00016200	-0.46726300
C	3.88469400	0.85682100	-1.04948400
H	4.09059400	0.43080200	-2.03068400
H	3.49506000	1.87283700	-1.14207600
H	4.78321300	0.86237800	-0.42820500
Br	0.53698100	-1.87166400	-1.22498100

22NH_radical

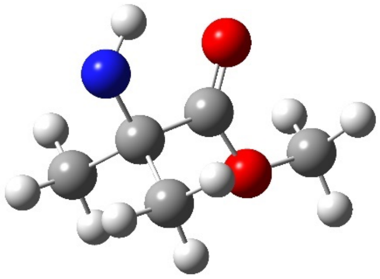

UB3LYP/6-31+G(d,p) free energy: -401.617281 (a.u.)

UB3LYP/6-31+G(d,p) thermal correction to Gibbs free energy: 0.113149 (a.u.)

Number of imaginary frequencies: 0

UM06/6-311+G(d,p)/SMD(DMF) single point energy: -401.565031 (a.u.)

C	0.46411500	0.39584300	0.04685400
O	0.85531100	1.54624600	0.10233900
O	1.29162400	-0.66285700	-0.02554200
C	-1.02362000	-0.01204400	0.02859000
C	-1.36506200	-0.56707200	-1.38442100
H	-0.76860300	-1.46004700	-1.59093200
H	-2.42686400	-0.82353300	-1.41592100
H	-1.16654400	0.18058700	-2.15822100
C	-1.31577100	-1.08668700	1.10089100
H	-1.07095000	-0.71498300	2.10049800
H	-2.38250600	-1.32381900	1.07807800
H	-0.73559400	-1.99275800	0.91070100
C	2.70452700	-0.36693200	-0.03723600
H	3.20148800	-1.33455400	-0.09638600
H	2.95457300	0.25132800	-0.90235300
H	2.98812700	0.15954900	0.87674500
N	-1.89195600	1.12046800	0.26417400
H	-1.31005000	1.96919000	0.20613700

23NBr_radical

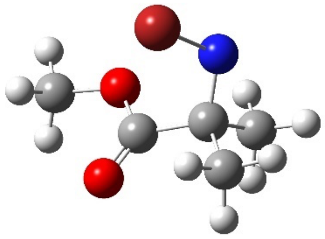

UB3LYP/Lanl2dz(Br)-6-31+G(d,p) free energy: -414.163219 (a.u.)

UB3LYP/Lanl2dz(Br)-6-31+G(d,p) thermal correction to Gibbs free energy: 0.100800 (a.u.)

Number of imaginary frequencies: 0

UM06/SDD(Br)-6-311+G(d,p)/SMD(DMF) single point energy: -414.294029 (a.u.)

C	1.30097600	-0.04654700	0.37427200
O	1.73917000	-0.13668900	1.50094100
O	1.54332200	-0.93757000	-0.60228000
C	0.43923600	1.12921000	-0.14145500
C	-0.01234600	2.00815800	1.03961800
H	0.86468100	2.43760900	1.53011200
H	-0.64904600	2.81664500	0.66980200
H	-0.56459200	1.42405100	1.77768900
C	1.28092700	1.95316300	-1.14879400
H	1.56472200	1.34194000	-2.00770000
H	0.69573900	2.80764000	-1.49929800
H	2.18590700	2.32552800	-0.65776600
N	-0.70593900	0.68146300	-0.94125800
Br	-1.86029500	-0.57187700	0.02066000
C	2.34109500	-2.08179200	-0.23427000
H	2.41026600	-2.68625300	-1.13792500
H	3.33220900	-1.76214600	0.09653300
H	1.85273800	-2.63863800	0.56874700

NH-NH

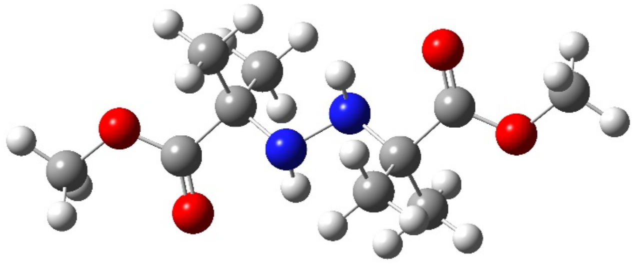

RB3LYP/6-31+G(d,p) free energy: -803.281080 (a.u.)

RB3LYP/6-31+G(d,p) thermal correction to Gibbs free energy: 0.260197 (a.u.)

Number of imaginary frequencies: 0

RM06/6-311+G(d,p)/SMD(DMF) single point energy: -803.224365 (a.u.)

C	2.99922000	-0.29689800	-0.20812000
O	2.97266900	-1.23855900	-0.97759300
O	4.12412200	0.12581500	0.39762600
C	-2.99922000	0.29689800	0.20812000
O	-2.97266900	1.23855900	0.97759300
O	-4.12412200	-0.12581500	-0.39762600
C	5.32679500	-0.60080800	0.07188000
H	6.11800600	-0.12058900	0.64662200
H	5.22345100	-1.65039400	0.35576500
H	5.53094100	-0.53587300	-0.99942400
C	-5.32679500	0.60080800	-0.07188000
H	-5.22345100	1.65039400	-0.35576500
H	-6.11800600	0.12058900	-0.64662200
H	-5.53094100	0.53587300	0.99942400
C	1.77677500	0.58002000	0.10949200
C	-1.77677500	-0.58002000	-0.10949200
C	1.74220800	1.68789600	-0.97803100
H	0.86082100	2.31660400	-0.83832900
H	2.63856100	2.31327000	-0.91620200
H	1.68915100	1.24636900	-1.97814700
C	1.85699800	1.20423700	1.51164700
H	2.74304900	1.83439600	1.60850500

H	0.98073800	1.83439300	1.69515100
H	1.89171200	0.42628500	2.27919100
C	-1.85699800	-1.20423700	-1.51164700
H	-0.98073800	-1.83439300	-1.69515100
H	-2.74304900	-1.83439600	-1.60850500
H	-1.89171200	-0.42628500	-2.27919100
C	-1.74220800	-1.68789600	0.97803100
H	-2.63856100	-2.31327000	0.91620200
H	-0.86082100	-2.31660400	0.83832900
H	-1.68915100	-1.24636900	1.97814700
N	-0.63465900	0.36290200	-0.04890900
H	-0.73785400	0.85708200	0.83687800
N	0.63465900	-0.36290200	0.04890900
H	0.73785400	-0.85708200	-0.83687800

NH-NBr

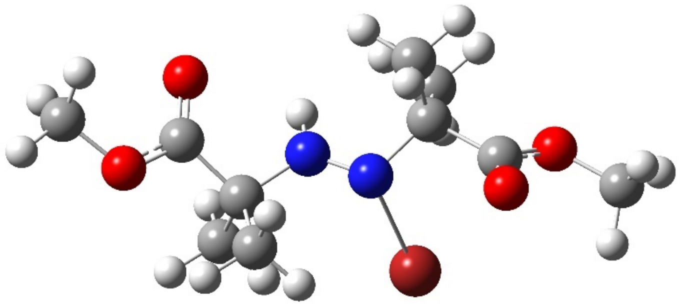

RB3LYP/Lanl2dz(Br)-6-31+G(d,p) free energy: -815.837908 (a.u.)

RB3LYP/Lanl2dz(Br)-6-31+G(d,p) thermal correction to Gibbs free energy: 0.244763 (a.u.)

Number of imaginary frequencies: 0

RM06/SDD(Br)-6-311+G(d,p)/SMD(DMF) single point energy: -815.975960 (a.u.)

C	-2.53041700	-0.97750500	0.49284700
O	-2.58950900	-0.93650400	1.70176500
O	-3.59411000	-0.90245400	-0.32162200
C	3.26309100	-0.39389900	-0.16965500
O	3.23119600	-1.24743700	-1.03813900
O	4.36405000	-0.05803300	0.51075900
C	-4.84784000	-0.59656600	0.32163000

H	-5.58727300	-0.58531100	-0.47857500
H	-5.08978100	-1.35772400	1.06731700
H	-4.78176600	0.38172200	0.80295400
C	5.57512100	-0.76460600	0.15654200
H	5.81856000	-0.59028800	-0.89380300
H	6.34566900	-0.35440100	0.80765000
H	5.44929200	-1.83553300	0.32893400
C	-1.21622800	-1.25482700	-0.27451800
C	2.05191200	0.47152600	0.21957000
C	-1.36618900	-1.23284600	-1.80081900
H	-0.43753600	-1.53492900	-2.29992700
H	-2.13209300	-1.95018300	-2.10182200
H	-1.66139600	-0.23910000	-2.14233000
C	-0.71581500	-2.64249900	0.21399700
H	-1.46184700	-3.40056700	-0.04287300
H	0.22741000	-2.90507100	-0.27299400
H	-0.58148400	-2.63897700	1.29751300
C	2.18842700	1.82783800	-0.51984900
H	1.29641400	2.43003400	-0.33641800
H	3.07327500	2.35227900	-0.14962300
H	2.29454700	1.68005900	-1.59951400
C	1.90626100	0.66685900	1.73196300
H	2.78149200	1.19800700	2.10916300
H	1.00685200	1.24961200	1.93844700
H	1.82295300	-0.29171600	2.25158800
N	0.88916100	-0.27000500	-0.31762500
N	-0.24208400	-0.26188800	0.27528400
Br	-1.45895600	1.92264500	-0.22224100
H	1.00767800	-0.64267800	-1.26324200

NBr-NBr

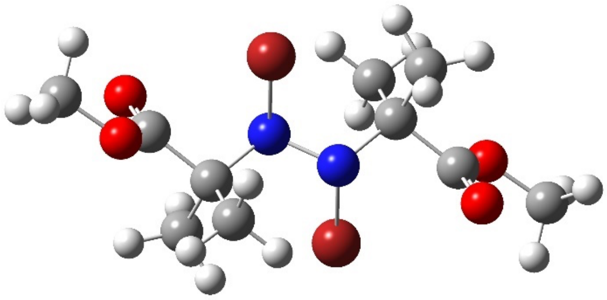

RB3LYP/Lanl2dz(Br)-6-31+G(d,p) free energy: -828.303040 (a.u.)

RB3LYP/Lanl2dz(Br)-6-31+G(d,p) thermal correction to Gibbs free energy: 0.233911 (a.u.)

Number of imaginary frequencies: 0

RM06/SDD(Br)-6-311+G(d,p)/SMD(DMF) single point energy: -828.618785 (a.u.)

C	3.05533300	-0.31492100	0.34480600
O	3.19022300	-1.33011700	0.99284100
O	4.02746100	0.21780600	-0.41936900
C	-3.05533300	0.31492100	-0.34480600
O	-3.19022300	1.33011700	-0.99284100
O	-4.02746100	-0.21780600	0.41936900
C	5.25875600	-0.53064600	-0.46960200
H	5.92142400	0.04788400	-1.11265700
H	5.68167600	-0.63557200	0.53246400
H	5.07858000	-1.52290100	-0.88992000
C	-5.25875600	0.53064600	0.46960200
H	-5.68167600	0.63557200	-0.53246400
H	-5.92142400	-0.04788400	1.11265700
H	-5.07858000	1.52290100	0.88992000
C	1.80601600	0.60239200	0.42150900
C	-1.80601600	-0.60239200	-0.42150900
C	1.83556400	1.79168900	-0.54102500
H	0.92623600	2.38419100	-0.42353900
H	2.69834100	2.42299700	-0.31663600
H	1.90935300	1.46966100	-1.58126700
C	1.90394700	1.08417000	1.90242700
H	2.95723700	1.30149500	2.11082800

H	1.33950500	1.99651300	2.07393000
H	1.57857000	0.30280600	2.59037300
C	-1.90394700	-1.08417000	-1.90242700
H	-1.33950500	-1.99651300	-2.07393000
H	-2.95723700	-1.30149500	-2.11082800
H	-1.57857000	-0.30280600	-2.59037300
C	-1.83556400	-1.79168900	0.54102500
H	-2.69834100	-2.42299700	0.31663600
H	-0.92623600	-2.38419100	0.42353900
H	-1.90935300	-1.46966100	1.58126700
N	-0.60949900	0.35277900	-0.19533400
N	0.60949900	-0.35277900	0.19533400
Br	-1.17055200	1.34667900	1.56380600
Br	1.17055200	-1.34667900	-1.56380600

Br_radical

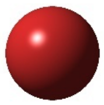

UB3LYP/Lanl2dz(Br)-6-31+G(d,p) free energy: -13.147277 (a.u.)

UB3LYP/Lanl2dz(Br)-6-31+G(d,p) thermal correction to Gibbs free energy: -0.016830 (a.u.)

Number of imaginary frequencies: 0

UM06/SDD(Br)-6-311+G(d,p)/SMD(DMF) single point energy: -13.3071379 (a.u.)

Br	0.00000000	0.00000000	0.00000000

Br_2

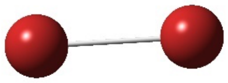

RB3LYP/Lanl2dz(Br)-6-31+G(d,p) free energy: -26.332813 (a.u.)

RB3LYP/Lanl2dz(Br)-6-31+G(d,p) thermal correction to Gibbs free energy: -0.023764 (a.u.)

Number of imaginary frequencies: 0

RM06/SDD(Br)-6-311+G(d,p)/SMD(DMF) single point energy: -26.675238 (a.u.)

Br	0.00000000	0.00000000	1.25530100
Br	0.00000000	0.00000000	-1.25530100

DBU

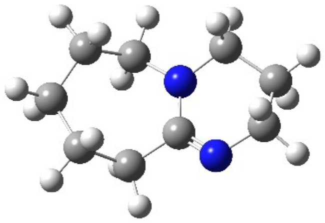

RB3LYP/6-31+G(d,p) free energy: -461.918461 (a.u.)

RB3LYP/6-31+G(d,p) thermal correction to Gibbs free energy: 0.210098 (a.u.)

Number of imaginary frequencies: 0

RM06/6-311+G(d,p)/SMD(DMF) single point energy: -461.877396 (a.u.)

C	0.35340200	-0.72687900	-0.29102800
C	-0.93042900	-1.42972600	-0.69169100
C	-2.07137800	-1.29522600	0.34099000
C	-2.83359600	0.03655300	0.29182900
C	-0.85415800	1.44704100	-0.56816600
C	-1.96911100	1.29055500	0.48374500
H	-1.27602100	-1.07966100	-1.67426100
H	-1.65737900	-1.45829700	1.34498100
H	-3.34413200	0.11439000	-0.67989400
H	-1.25908000	1.21946200	-1.56442600
H	-0.65665800	-2.48086300	-0.79786400
H	-2.78757500	-2.10822800	0.16888500
H	-3.62563000	0.02584100	1.05157700
H	-0.53627000	2.49318600	-0.60214500
H	-2.61839200	2.17455800	0.42715200
H	-1.51683900	1.29650600	1.48474400
C	2.58894800	-0.85492900	0.48992700
H	3.41494000	-1.51165100	0.19182900
H	2.61232100	-0.81875200	1.59035600
C	2.79457500	0.55017300	-0.08237400
H	3.66230900	1.04194600	0.37234100
H	2.97904800	0.48449700	-1.16173300
C	1.53665300	1.38016100	0.16092000
H	1.43756000	1.61029700	1.23482800
H	1.59960900	2.33740600	-0.36942700
N	0.35726200	0.66127300	-0.32661800
N	1.34027600	-1.47941300	0.06635300

22-DBU

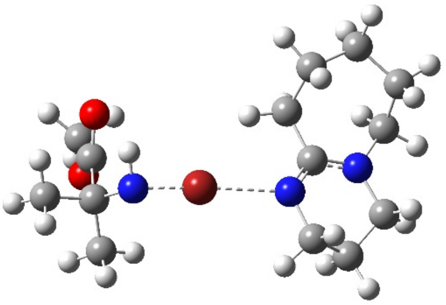

RB3LYP/Lanl2dz(Br)-6-31+G(d,p) free energy: -876.702649 (a.u.)

RB3LYP/Lanl2dz(Br)-6-31+G(d,p) thermal correction to Gibbs free energy: 0.340474 (a.u.)

Number of imaginary frequencies: 0

RM06/SDD(Br)-6-311+G(d,p)/SMD(DMF) single point energy: -876.832167 (a.u.)

C	-2.58262400	-0.07848300	0.40446500
C	-2.13894400	1.37098400	0.35182300
C	-2.58864700	2.11829700	-0.92375500
C	-4.04348700	2.60878700	-0.90323800
C	-4.93214400	0.74024300	0.63615400
C	-5.09751600	1.51335700	-0.68639600
H	-2.48738800	1.91230000	1.24213300
H	-2.41846700	1.46784300	-1.79169000
H	-4.15226400	3.35643200	-0.10306300
H	-4.71774500	1.44767100	1.44944100
H	-1.04918800	1.34586400	0.39211600
H	-1.93215000	2.98625900	-1.05943500
H	-4.25925600	3.13445300	-1.84221000
H	-5.88052000	0.25993400	0.89368500
H	-6.09204600	1.97931000	-0.68631300
H	-5.08595200	0.79800700	-1.51994100
C	-2.04324200	-2.39161600	0.23714900
H	-1.20303000	-2.96026700	0.65171800
H	-2.15983100	-2.73498000	-0.80208100
C	-3.32215600	-2.66949500	1.03069400
H	-3.65386500	-3.70547100	0.89672800
H	-3.13220200	-2.51809600	2.10044400
C	-4.41652800	-1.70772600	0.57409100

H	-4.74875500	-1.96781500	-0.44430300
H	-5.29208500	-1.78388500	1.22897400
N	-3.92765200	-0.32665800	0.62081800
N	-1.67252600	-0.98106400	0.22890300
C	3.77340800	0.87762500	0.35121500
O	3.44239400	1.92969800	-0.16184900
O	4.10230500	0.76234600	1.65168500
C	3.91517200	-0.44039600	-0.44286900
C	5.24366500	-0.32839200	-1.23495500
H	6.08955900	-0.23067300	-0.54608700
H	5.37822300	-1.23058700	-1.83723100
H	5.23420200	0.54259800	-1.89719500
C	3.93700500	-1.69292400	0.43967000
H	3.03778900	-1.76861500	1.05440600
H	3.99032700	-2.57341000	-0.20715300
H	4.80479900	-1.68242800	1.10304900
N	2.87216000	-0.54963800	-1.47604600
H	2.83189700	0.35366500	-1.95321800
Br	1.00612700	-0.66012000	-0.72386900
C	4.02707700	1.97328700	2.43086200
H	4.31905000	1.68624200	3.44081600
H	4.70878600	2.72699200	2.02897900
H	3.00798200	2.36673400	2.41895600

23-DBU

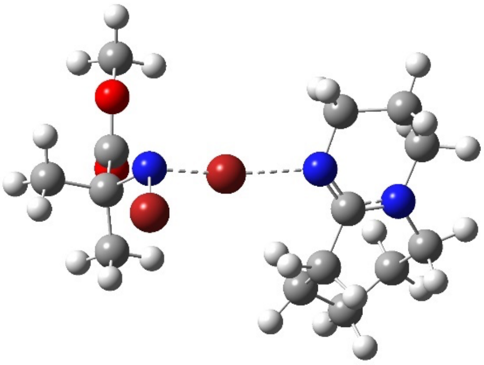

RB3LYP/Lanl2dz(Br)-6-31+G(d,p) free energy: -889.233113 (a.u.)

RB3LYP/Lanl2dz(Br)-6-31+G(d,p) thermal correction to Gibbs free energy: 0.331133 (a.u.)

Number of imaginary frequencies: 0

RM06/SDD(Br)-6-311+G(d,p)/SMD(DMF) single point energy: -889.543797 (a.u.)

C	2.52513800	2.02847500	-0.29364500
O	1.97719500	2.98232300	0.21954300
O	2.79776700	1.94339500	-1.60540400
C	3.07399900	0.81273200	0.50238600
C	4.61786500	0.89240400	0.42046800
H	4.94994900	1.88316000	0.74903700
H	5.07053100	0.14567400	1.07617900
H	4.95622900	0.72396400	-0.60380700
C	2.59882300	0.90608200	1.95671300
H	1.50913200	0.89920700	2.01836200
H	2.99594500	0.06320900	2.52709500
H	2.95469300	1.83653300	2.40553300
C	2.34048800	3.04222600	-2.41499700
H	2.63133200	2.78885300	-3.43417600
H	1.25525600	3.14366900	-2.33621300
H	2.81386600	3.97484200	-2.09709900
N	2.64545900	-0.39087600	-0.27809700
Br	3.30685700	-2.08189300	0.60772000
Br	0.60777500	-0.51514600	-0.37124300
C	-3.00025900	-0.51762100	0.03354600
C	-2.65439400	-0.37139700	1.50222800
C	-2.87993400	1.05185100	2.06041700
C	-4.33763600	1.37718600	2.41551600
C	-5.39517000	-0.18238500	0.65367600
C	-5.33727900	1.23297900	1.25908100
H	-3.21967600	-1.09958200	2.09921300
H	-2.49043100	1.78183600	1.33906500
H	-4.65752600	0.71492100	3.23392900
H	-5.40193800	-0.92839500	1.46038500
H	-1.59973500	-0.63457000	1.58829700
H	-2.27035900	1.16276000	2.96510000
H	-4.39151000	2.39902100	2.81171100
H	-6.33964700	-0.30621500	0.11669100
H	-6.33991900	1.48434600	1.62962100
H	-5.10844000	1.95167900	0.46057700

C	-2.28531300	-0.76405500	-2.23224000
H	-1.50565600	-1.39791600	-2.66825300
H	-2.17783100	0.22490800	-2.70296400
C	-3.67278800	-1.33944500	-2.52183300
H	-3.90379000	-1.29135600	-3.59181600
H	-3.70269000	-2.39431500	-2.22327900
C	-4.71737500	-0.56002100	-1.72739400
H	-4.83151700	0.45465700	-2.14147200
H	-5.69502100	-1.04995800	-1.79638900
N	-4.33654500	-0.49654100	-0.31224100
N	-2.01892300	-0.64035000	-0.80375800

Br_radical-DBU

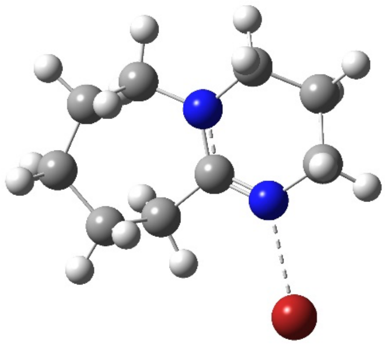

UB3LYP/Lanl2dz(Br)-6-31+G(d,p) free energy: -475.072070 (a.u.)

UB3LYP/Lanl2dz(Br)-6-31+G(d,p) thermal correction to Gibbs free energy: 0.204727 (a.u.)

Number of imaginary frequencies: 0

UM06/SDD(Br)-6-311+G(d,p)/SMD(DMF) single point energy: -475.215405 (a.u.)

C	0.46093000	0.20363800	0.41868600
C	0.49538700	-1.23673600	0.88249200
C	1.04077800	-2.21549500	-0.18118100
C	2.57147200	-2.25101700	-0.29457700
C	2.95177900	0.20080900	0.41356000
C	3.23426900	-0.90449100	-0.62075800
H	1.08689800	-1.31631200	1.80422800
H	0.58828600	-1.97315500	-1.15108800
H	2.98741600	-2.62055700	0.65468600
H	3.05192100	-0.20640800	1.42860300

H	-0.53438200	-1.49790900	1.12638600
H	0.68690400	-3.22074600	0.07549500
H	2.85810700	-2.98482800	-1.05828200
H	3.70789400	0.98533000	0.32283500
H	4.32127300	-1.05127300	-0.66964000
H	2.92084800	-0.55145900	-1.61253100
C	-0.84853900	2.09764100	-0.30577400
H	-1.77363000	2.50230900	0.11607300
H	-0.98107200	2.08164200	-1.39648700
C	0.35699100	2.95829000	0.08575100
H	0.31771000	3.93066800	-0.41725200
H	0.34241000	3.14237700	1.16641300
C	1.64876900	2.23168000	-0.28580500
H	1.76396900	2.19557900	-1.38042900
H	2.51584800	2.76678200	0.11547100
N	1.65388600	0.87313500	0.27197300
N	-0.69197700	0.73787100	0.17249100
Br	-2.91613600	-0.57042900	-0.08314500

TS1 (transition state between **22N-Cu(i)** and **22Br-Cu(i)**)

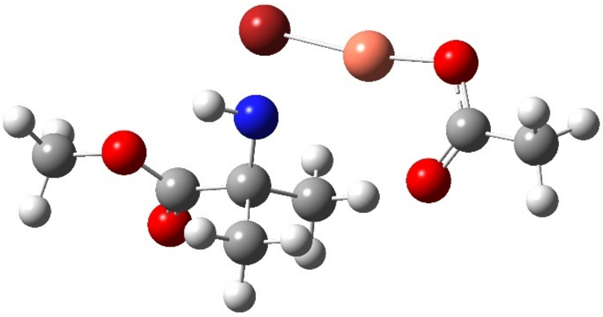

RB3LYP/Lanl2dz(Br)-6-31+G(d,p) free energy: -2283.643818 (a.u.)

RB3LYP/Lanl2dz(Br)-6-31+G(d,p) thermal correction to Gibbs free energy: 0.153303 (a.u.)

Number of imaginary frequencies: 1 (-77.9777)

RM06/SDD(Br)-6-311+G(d,p)/SMD(DMF) single point energy: -2283.830802 (a.u.)

C	3.55063900	0.87042500	-0.09055900
O	2.54497900	1.57301100	0.12995400

O	3.50455100	-0.42008300	-0.23988700
C	4.93603400	1.48493700	-0.19896700
H	5.58485200	1.06404700	0.57607100
H	5.38069700	1.22804500	-1.16564400
H	4.88479300	2.56890100	-0.08840200
Cu	1.68420700	-0.91917900	-0.07963300
Br	-0.62307900	-1.77809200	0.22511800
N	-0.61599900	0.06794100	-0.67938200
C	-1.27610700	1.12721900	0.10122300
C	-1.02788900	2.37983300	-0.79228100
H	0.04813300	2.55753800	-0.85573200
H	-1.44115900	2.25110600	-1.79740600
H	-1.51301700	3.24205900	-0.32452400
C	-0.61764300	1.32368400	1.46743300
H	-0.79667800	0.46640600	2.12203400
H	0.45741300	1.46689800	1.32928200
H	-1.04507500	2.20243900	1.95381300
C	-2.80005100	0.93440600	0.24137400
O	-3.46912400	1.36900300	1.15250000
O	-3.31731400	0.26064500	-0.81112400
C	-4.75013300	0.07544300	-0.80695000
H	-4.97272300	-0.48409900	-1.71467800
H	-5.05314700	-0.48504000	0.07998400
H	-5.25351200	1.04476400	-0.81340300
H	-1.19666700	-0.14553100	-1.49466700

TS2 (transition state between **22N-Cu(i)** and **22-Cu(iii)**)

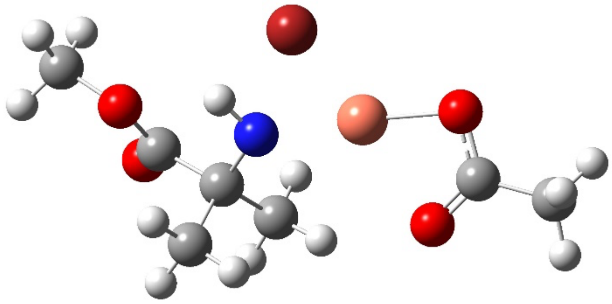

RB3LYP/Lanl2dz(Br)-6-31+G(d,p) free energy: -2283.661356 (a.u.)

RB3LYP/Lanl2dz(Br)-6-31+G(d,p) thermal correction to Gibbs free energy: 0.138757 (a.u.)

Number of imaginary frequencies: 1 (-129.3075)

RM06/SDD(Br)-6-311+G(d,p)/SMD(DMF) single point energy: -2283.844366 (a.u.)

C	3.63605900	0.45083400	0.03894100
O	2.89983400	1.40245300	0.37413800
O	3.14021300	-0.64097600	-0.46427900
C	5.13915200	0.50278200	0.19075200
H	5.47140200	-0.31594900	0.83673600
H	5.61269600	0.35695900	-0.78519700
H	5.44755200	1.45882400	0.61496200
Cu	1.28985700	-0.26150900	-0.47777100
Br	-0.49936100	-1.75941000	0.38447600
N	-0.47015700	0.25328800	-0.72185500
C	-1.23279700	1.22434300	0.07600600
C	-1.28377400	2.50236800	-0.81292000
H	-0.27158800	2.89187200	-0.94128400
H	-1.71983300	2.29468300	-1.79345200
H	-1.89726500	3.25700300	-0.31014700
C	-0.54353000	1.52742500	1.40682100
H	-0.56445000	0.65531800	2.06311500
H	0.49495400	1.82157900	1.23017100
H	-1.06683200	2.34085700	1.91306200
C	-2.67993400	0.73924400	0.30270600
O	-3.30479100	0.88008800	1.32851200
O	-3.18372500	0.17919700	-0.81916400
C	-4.52723200	-0.34303000	-0.72491200
H	-4.73973800	-0.77246600	-1.70296200
H	-4.57667500	-1.10653000	0.05398200
H	-5.22737100	0.46221600	-0.49266600
H	-1.05986200	-0.14415900	-1.45266300

TS3 (transition state between **23N-Cu(i)** and **23Br-Cu(i)**)

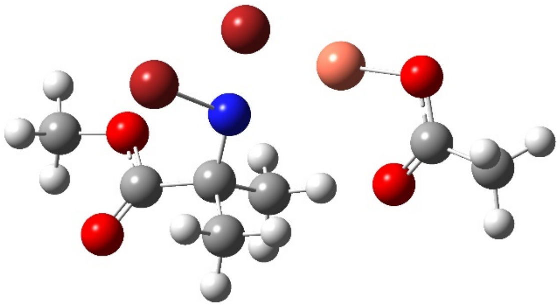

RB3LYP/Lanl2dz(Br)-6-31+G(d,p) free energy: -2296.174650 (a.u.)

RB3LYP/Lanl2dz(Br)-6-31+G(d,p) thermal correction to Gibbs free energy: 0.138757 (a.u.)

Number of imaginary frequencies: 1 (-123.7921)

RM06/SDD(Br)-6-311+G(d,p)/SMD(DMF) single point energy: -2296.534724 (a.u.)

C	-3.83008000	0.75816200	-0.02570500
O	-2.83630900	1.42622700	-0.40736800
O	-3.74873000	-0.50393600	0.22822800
C	-5.18768800	1.40772000	0.15663500
H	-5.52155900	1.27449500	1.19056000
H	-5.14414500	2.47025300	-0.08549500
H	-5.92051500	0.90932100	-0.48579900
Cu	-1.86540700	-0.78074700	-0.14472200
Br	0.36872500	-1.67651900	-0.89909000
N	0.35464100	0.02656900	0.29677800
C	0.91562200	1.26164900	-0.34564400
C	0.40767900	2.40592800	0.56393400
H	-0.68347300	2.38115400	0.59474700
H	0.81935500	2.34134900	1.57147600
H	0.74147100	3.34979800	0.12593300
C	0.30442000	1.43166600	-1.75287800
H	0.65742500	0.66623900	-2.44566000
H	-0.78662500	1.41508200	-1.68782500
H	0.61101400	2.41052000	-2.13391600
C	2.45480800	1.30460000	-0.45803000
O	3.12621400	2.26858100	-0.16080400
O	2.95273800	0.17198700	-0.98025000

C	4.38782900	0.12009000	-1.13566200
H	4.59539200	-0.86054600	-1.56188300
H	4.72254100	0.91554200	-1.80513900
H	4.87427800	0.23280800	-0.16426200
Br	1.34505600	-0.44039800	1.97201600

TS4 (transition state between **23N-Cu(i)** and **23-Cu(iii)**)

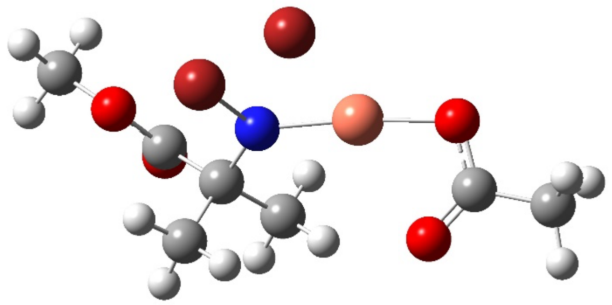

RB3LYP/Lanl2dz(Br)-6-31+G(d,p) free energy: -2296.184607 (a.u.)

RB3LYP/Lanl2dz(Br)-6-31+G(d,p) thermal correction to Gibbs free energy: 0.140660 (a.u.)

Number of imaginary frequencies: 1 (-123.7921)

RM06/SDD(Br)-6-311+G(d,p)/SMD(DMF) single point energy: -2296.542143 (a.u.)

C	3.93886700	0.42312400	-0.31562200
O	3.30741800	1.09898100	-1.14433100
O	3.34246300	-0.37152500	0.53590400
C	5.44910700	0.46008700	-0.21791200
H	5.74660000	0.76317200	0.79093900
H	5.85399900	-0.54290700	-0.38680700
H	5.85988700	1.15448100	-0.95176800
Cu	1.51752600	-0.26457500	0.20443400
Br	-0.49526500	-0.27597100	1.98099700
N	-0.25446700	-0.12812600	-0.36022100
C	-0.95308200	1.12477200	-0.77166000
C	-1.05110400	1.12412000	-2.31974500
H	-0.05630400	0.99281200	-2.75255100
H	-1.71849400	0.34297500	-2.68458500
H	-1.44179500	2.09761600	-2.63126800
C	-0.05561900	2.29001200	-0.31709000

H	0.02916200	2.32850500	0.77002800
H	0.93741200	2.19910100	-0.76573800
H	-0.51596100	3.22552300	-0.64274200
C	-2.37447600	1.31180200	-0.17777200
O	-2.74032000	2.33558600	0.35683700
O	-3.16116900	0.24845000	-0.38538100
C	-4.50063800	0.33409000	0.14964100
H	-4.97039300	-0.61588400	-0.10163900
H	-4.46209000	0.47496200	1.23184300
H	-5.03579100	1.16861200	-0.30887000
Br	-0.96641200	-1.83601100	-1.01120200

(i)-TS (transition state between (**22NH_radical** + **22**) and (**NH-NH** + **Br_radical**))

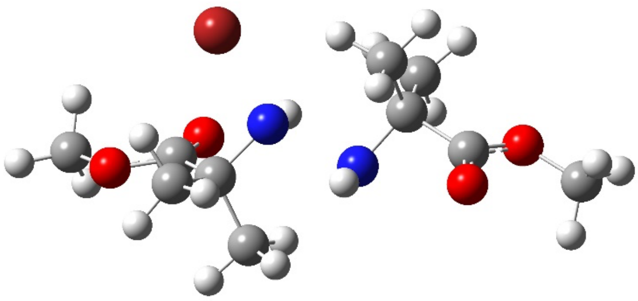

UB3LYP/Lanl2dz(Br)-6-31+G(d,p) free energy: -816.376286 (a.u.)

UB3LYP/Lanl2dz(Br)-6-31+G(d,p) thermal correction to Gibbs free energy: 0.249939 (a.u.)

Number of imaginary frequencies: 1 (-312.2587)

UM06/SDD(Br)-6-311+G(d,p)/SMD(DMF) single point energy: -816.503584 (a.u.)

C	-2.72544600	-0.99634500	-0.44178300
O	-2.57303400	-0.95173900	-1.64687100
O	-3.91506300	-1.13798100	0.15930700
C	3.73375700	-0.27880800	0.23234200
O	4.08286400	-0.57706300	1.35839900
O	4.47368100	-0.52419000	-0.86163100
C	-5.06047600	-1.20482900	-0.71823300
H	-5.92170300	-1.30828200	-0.05917300
H	-5.13081800	-0.29010400	-1.31066600
H	-4.97496400	-2.06511100	-1.38616900

C	5.73203100	-1.19292100	-0.63352900
H	5.56211100	-2.16773100	-0.17047200
H	6.18124100	-1.30481100	-1.61989100
H	6.36911500	-0.59016400	0.01791200
C	-1.54278900	-0.97887400	0.55162500
C	2.37245300	0.40605100	-0.08006100
C	-0.91159200	-2.39442300	0.42804400
H	-0.10134200	-2.52082100	1.14618300
H	-1.68806500	-3.13525300	0.64706500
H	-0.52081000	-2.57137400	-0.57477300
C	-1.93822300	-0.70827300	2.00613800
H	-2.57085100	-1.51420400	2.38418200
H	-1.03301900	-0.65902100	2.61905500
H	-2.47848700	0.23381600	2.10904400
C	2.21406600	0.76946900	-1.56152900
H	1.26704400	1.29798200	-1.70713900
H	3.01955800	1.43044000	-1.89179900
H	2.22349100	-0.12515000	-2.18832600
C	2.26817900	1.65706100	0.81883200
H	3.02134900	2.39999900	0.53300600
H	1.27899200	2.10620100	0.71006700
H	2.43075200	1.39196400	1.86704000
N	1.42729400	-0.66423700	0.27117600
H	1.41766200	-0.71426800	1.29649700
N	-0.56246800	0.05087100	0.08678200
H	-0.48786700	-0.06418100	-0.92878300
Br	-1.58047200	1.98636500	0.00904900

(ii)-TS (transition state between (**22NH_radical** + **23**) and (**NH-NBr** + **Br_radical**))

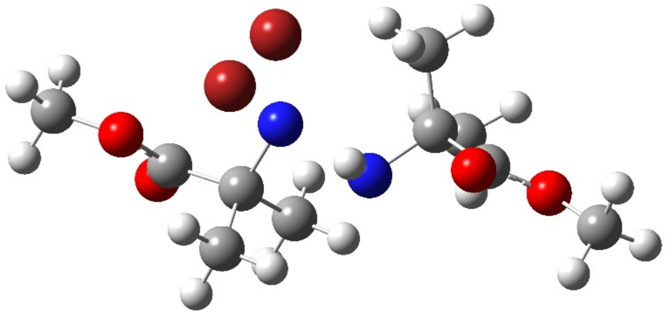

UB3LYP/Lanl2dz(Br)-6-31+G(d,p) free energy: -828.902297 (a.u.)

UB3LYP/Lanl2dz(Br)-6-31+G(d,p) thermal correction to Gibbs free energy: 0.237053 (a.u.)

Number of imaginary frequencies: 1 (-279.0330)

UM06/SDD(Br)-6-311+G(d,p)/SMD(DMF) single point energy: -829.207271 (a.u.)

C	3.83276200	0.31794300	0.16945500
O	4.00028500	1.52040500	0.10767200
O	4.79291300	-0.54912000	0.52528400
C	-2.78064600	-0.30133000	1.20000200
O	-3.37844800	-1.22793300	1.70395600
O	-3.37686800	0.69845700	0.53768600
C	6.07872800	0.02905200	0.83995300
H	6.71642500	-0.81481700	1.10097800
H	6.47497200	0.56351200	-0.02646700
H	5.98471600	0.72107300	1.67981900
C	-4.80116800	0.57234600	0.34813500
H	-5.31070100	0.54570200	1.31424500
H	-5.09325900	1.45364800	-0.22181600
H	-5.02485000	-0.34118600	-0.20726700
C	2.48276200	-0.37192900	-0.18707900
C	-1.24699400	-0.09451800	1.35155300
C	2.45913200	-1.86360900	0.16106800
H	1.48164100	-2.27460300	-0.10232600
H	3.22520800	-2.40695600	-0.39701000
H	2.63310900	-2.02278200	1.22804900
C	2.28020200	-0.15259800	-1.70710200
H	3.06981300	-0.66377000	-2.26858800

H	1.31772300	-0.56675300	-2.01312900
H	2.30876700	0.91126500	-1.95546400
C	-0.65131000	-1.35275200	2.01147700
H	0.40552200	-1.18618100	2.21064400
H	-1.18571100	-1.53204100	2.94693300
H	-0.77881000	-2.23373800	1.38182700
C	-1.02761200	1.13007400	2.26941300
H	-1.51724800	0.92668300	3.22729200
H	0.03898300	1.27214300	2.44665400
H	-1.45851900	2.03788700	1.84642000
N	-0.51592600	0.05786400	0.02611000
N	1.53436000	0.36076300	0.65836800
H	1.53390900	1.32692800	0.31068000
Br	-0.87791200	1.81386100	-0.90283800
Br	-1.35091400	-1.51834500	-1.35518700

(iii)-TS (transition state between (**23NBr_radical** + **22**) and (**NH-NBr** + **Br_radical**))

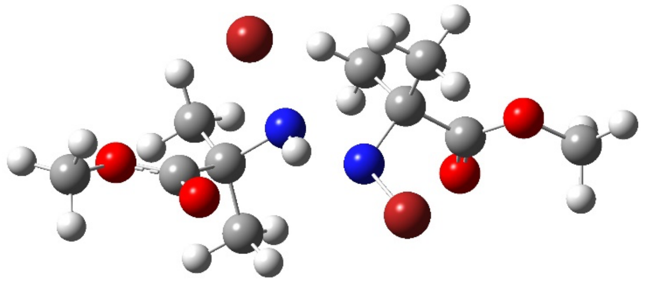

UB3LYP/Lanl2dz(Br)-6-31+G(d,p) free energy: -828.919603 (a.u.)

UB3LYP/Lanl2dz(Br)-6-31+G(d,p) thermal correction to Gibbs free energy: 0.238667 (a.u.)

Number of imaginary frequencies: 1 (-383.1110)

UM06/SDD(Br)-6-311+G(d,p)/SMD(DMF) single point energy: -829.224603 (a.u.)

C	3.01905900	-0.90423900	0.14010700
O	2.96069300	-1.37286600	-0.97971600
O	4.15541100	-0.74924800	0.83159800
C	-3.32386500	0.27107500	0.79880400
O	-3.52616400	-0.28251500	1.85879300
O	-4.28177900	0.51110400	-0.11230500

C	5.36572700	-1.14970800	0.15159700
H	6.17145300	-0.94472300	0.85548000
H	5.32522500	-2.21285400	-0.09631700
H	5.48961300	-0.56686600	-0.76354400
C	-5.60074700	0.03087100	0.22314800
H	-5.94970200	0.49688100	1.14760700
H	-6.23250800	0.31467200	-0.61786700
H	-5.58424700	-1.05413500	0.34896700
C	1.76163200	-0.50702700	0.94708900
C	-1.92763600	0.80559300	0.36164400
C	2.04478000	0.37076200	2.16850300
H	1.10216500	0.60472500	2.66887100
H	2.68605300	-0.16391800	2.87210400
H	2.53840800	1.30317000	1.89023400
C	1.15034800	-1.86253200	1.40281900
H	1.90275400	-2.38994700	1.99848900
H	0.26417100	-1.69175300	2.01276200
H	0.87954700	-2.48719300	0.54991500
C	-1.40484700	1.67148900	1.53115300
H	-0.42736600	2.08397500	1.27995700
H	-2.10150700	2.49939400	1.69902200
H	-1.34517600	1.08322100	2.44853300
C	-1.96833000	1.62067600	-0.93830600
H	-2.60367300	2.50029300	-0.80241900
H	-0.95994600	1.95696300	-1.18673000
H	-2.37053100	1.03953500	-1.76857500
N	-1.06554000	-0.40402900	0.34976300
N	0.83592000	0.19254200	-0.00463000
H	0.88813500	-0.33254400	-0.88619700
Br	-1.54775600	-1.62738600	-1.15948200
Br	1.91268300	1.98475600	-0.82830400

10. Functional Group Evaluation (FGE) Kit Evaluation[26]

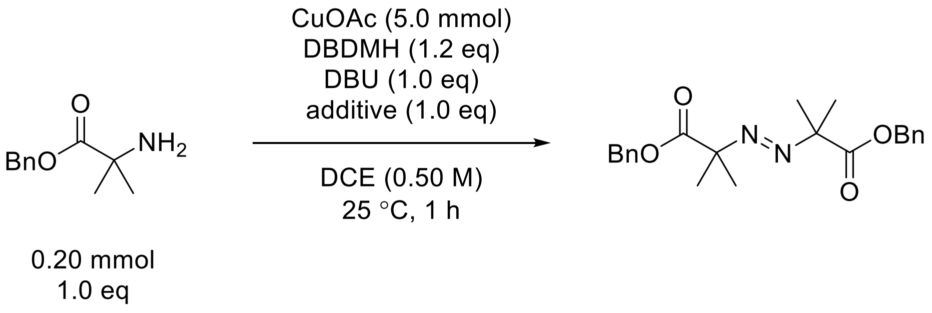

Condition: To a 4 ml vial equipped with a magnetic stirrer bar, CuOAc (1.2 mg, 10 µmol, 5.0 mol%) was added in a glove box followed by the addition of DCE (0.40 ml, 0.50 M), 1,3-dibromo-5,5-dimethylhydantoin (68.6 mg, 0.24 mmol, 1.2 eq), amine (0.20 mmol, 1.0 eq), 1,8-diazabicyclo[5.4.0]undec-7-ene (30 µl, 0.20 mmol, 1.0 eq) and additive (0.10 mmol, 1.0 eq) under Ar atmosphere. The reaction mixture was stirred at 25 °C for 1 hour. The yield of the product and the percentage of remaining additive were determined by ^{1}H NMR analysis of the crude mixture using 1,1,2,2-tetrachloroethane (dibenzyl was used when additive 10 was added) as an internal standard.

Table S6. Results of FGE Kit Evaluation

Additive No.	Structure	Yield (±SE)[a] (%)[b]		Additive remaining (%)[b]	
0	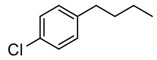	97 (±1.9)	±	>99	±
1	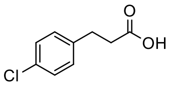	19 (±9.2)	X	0	X
2	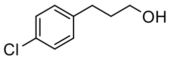	95 (±1.4)	±	98	±
3	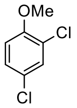	92 (±9.0)	±	>99	±
4	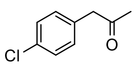	10 (±8.5)	X	0	X
5	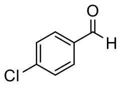	94 (±0.7)	±	77	–
6	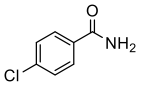	98 (±0.7)	±	74	–
7	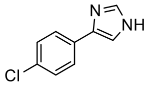	1 (±1.4)	X	0	X
8	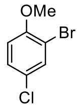	98 (±0.7)	±	>99	±
9	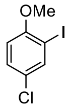	90 (±2.8)	–	>99	±
10	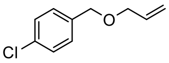	97[c] (±2.1)	±	0[c]	X
11	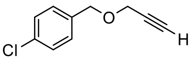	95 (±2.1)	±	26	X
12	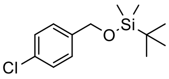	99 (±2.1)	±	78	–
13	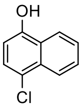	37 (±0.7)	X	0	X
14	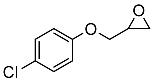	>99 (±0.7)	±	97	±
15	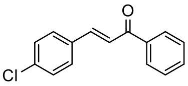	97 (±1.4)	±	83	–
16	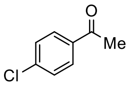	4 (±1.4)	X	19	X
17	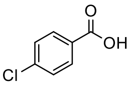	7 (±5.7)	X	0	X
18	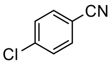	>99 (±2.1)	±	>99	±
19	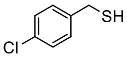	12 (±1.4)	X	0	X
20	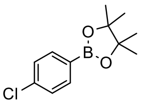	97 (±0.0)	±	>99	±
21	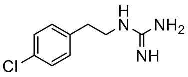	20 (±9.9)	X	0	X
22	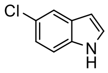	0 (±0.0)	X	0	X
23	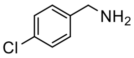	0 (±0.0)	X	0	X
24	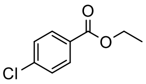	99 (±0.0)	±	>99	±
25	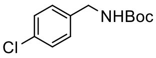	96 (±3.5)	±	82	–
26	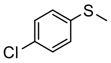	69 (±2.1)	–	0	X

[a] Standard error. [b] Determined by ^{1}H-NMR analysis using 1,1,2,2-tetrachloroethane as an internal standard. [c] Determined by ^{1}H-NMR analysis using dibenzyl as an internal standard.

11. Aminyl Radical detection

To a 4 ml vial equipped with a magnetic stirrer bar, CuOAc (1.2 mg, 10 μmol, 5.0 mol%) and *N*-bromosuccinimide (35.6 mg, 1.0 eq) was added in a glove box followed by the addition of dichloroethane (0.40 ml, 0.5 M), amine **1a** (0.20 mmol, 1.0 eq) under Ar atmosphere. The reaction mixture was stirred at 25 °C for 5 min. To the reaction mixture was added 1,8-diazabicyclo[5.4.0]undec-7-ene (30 μl, 0.20 mmol, 1.0 eq). After stirring for 5 min, 5,5-dimethyl-1-pyrroline *N*-oxide (45.3 mg, 2.0 eq) was added. The resulting solution was analyzed by HR-MS and EPR spectroscopy (X-band ESR spectroscopy JES-FA100, JEOL, Japan).

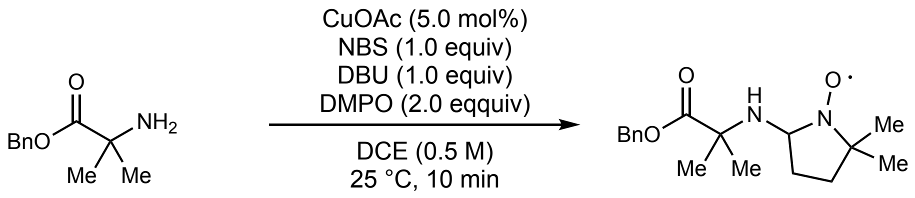

1a **24**

Exact mass: 305.1860
HRMS (DART): 305.1868

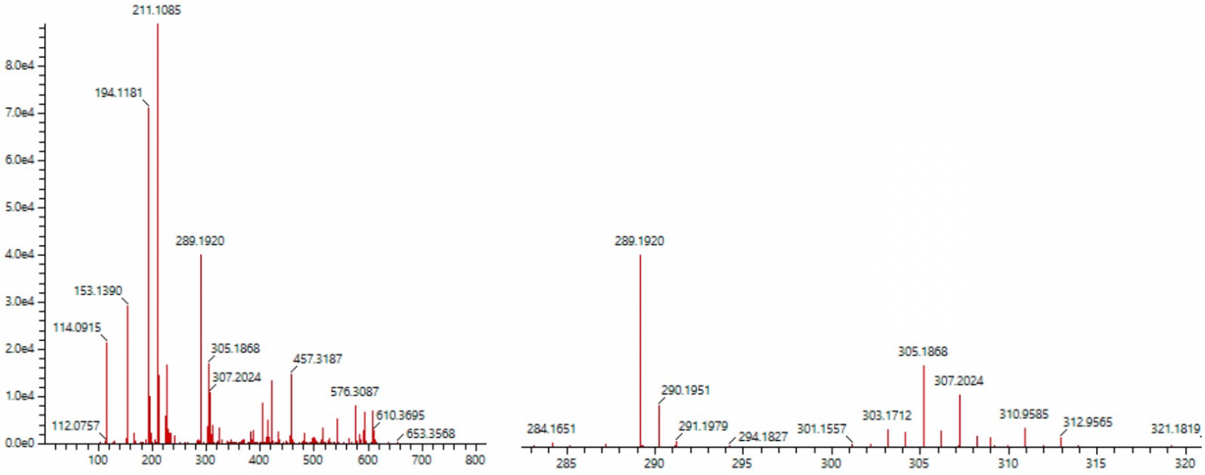

Compound **24** showed an EPR spectrum at g = 2.02962 in 1,2-dichloroethane. The spectrum was reproduced by assuming hyperfine coupling with two nitrogen and one hydrogen atoms (aN = 23.27 G, aH = 34.06 G, aN′ = 3.70 G). The microwave power was 10 mW. The magnetic field was 336.2±5 mT. The amplitude of the 100-kHz field modulation was 0.1 mT. The external magnetic field was swept at a scan rate of 10 mT/min. The time constant was 0.03 s. The spectral simulation was conducted with the EasySpin toolbox[27] on the MATLAB program.

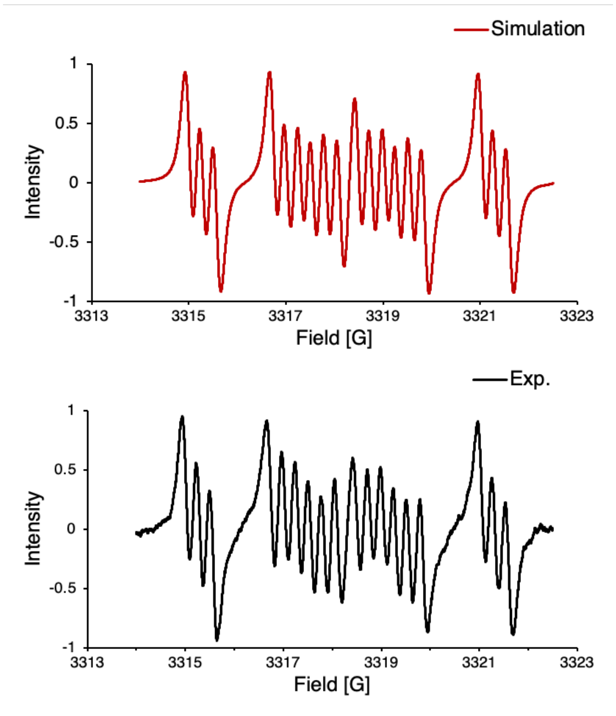

g: 2.02962 a_N: 23.27 G a_H: 34.06 G a_N': 3.70 G

12. Thermogravimetric Analysis

Thermal decomposition behaviors were assessed using thermogravimetric analysis (TGA) with a TG/DTA7300 instrument (HITACHI). The data were obtained from 30 to 550 ºC at a heating rate of 10 ºC /min. The analyses were conducted under a nitrogen (N_2) gas flow.

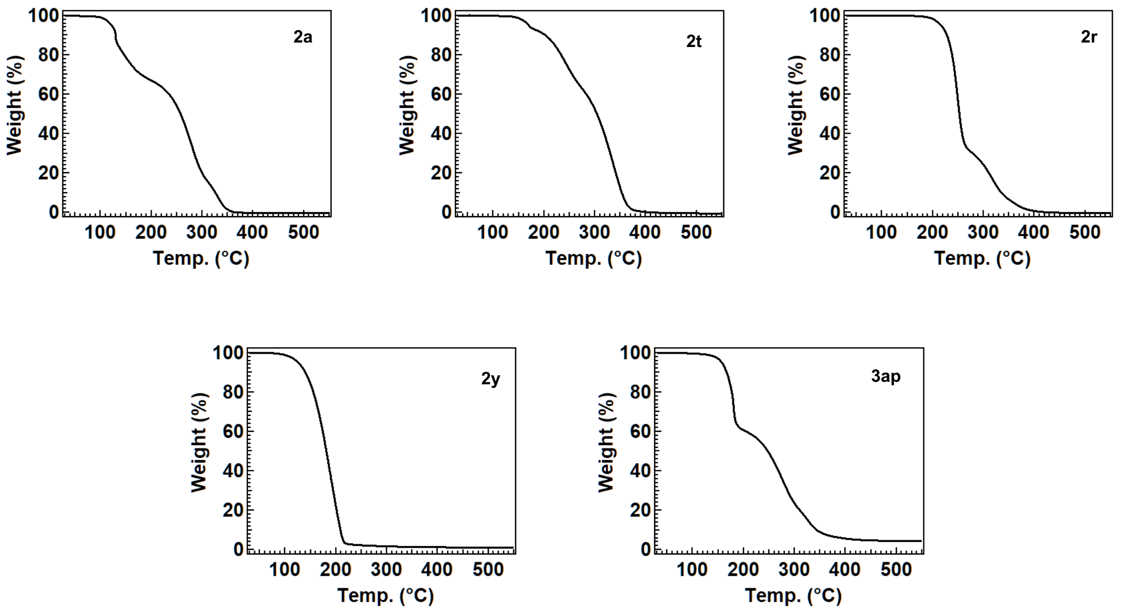

Figure S6. Thermal Stabilities of Representative Compounds (**2a**, **2t**, **2r**, **2y**, **3ap**)

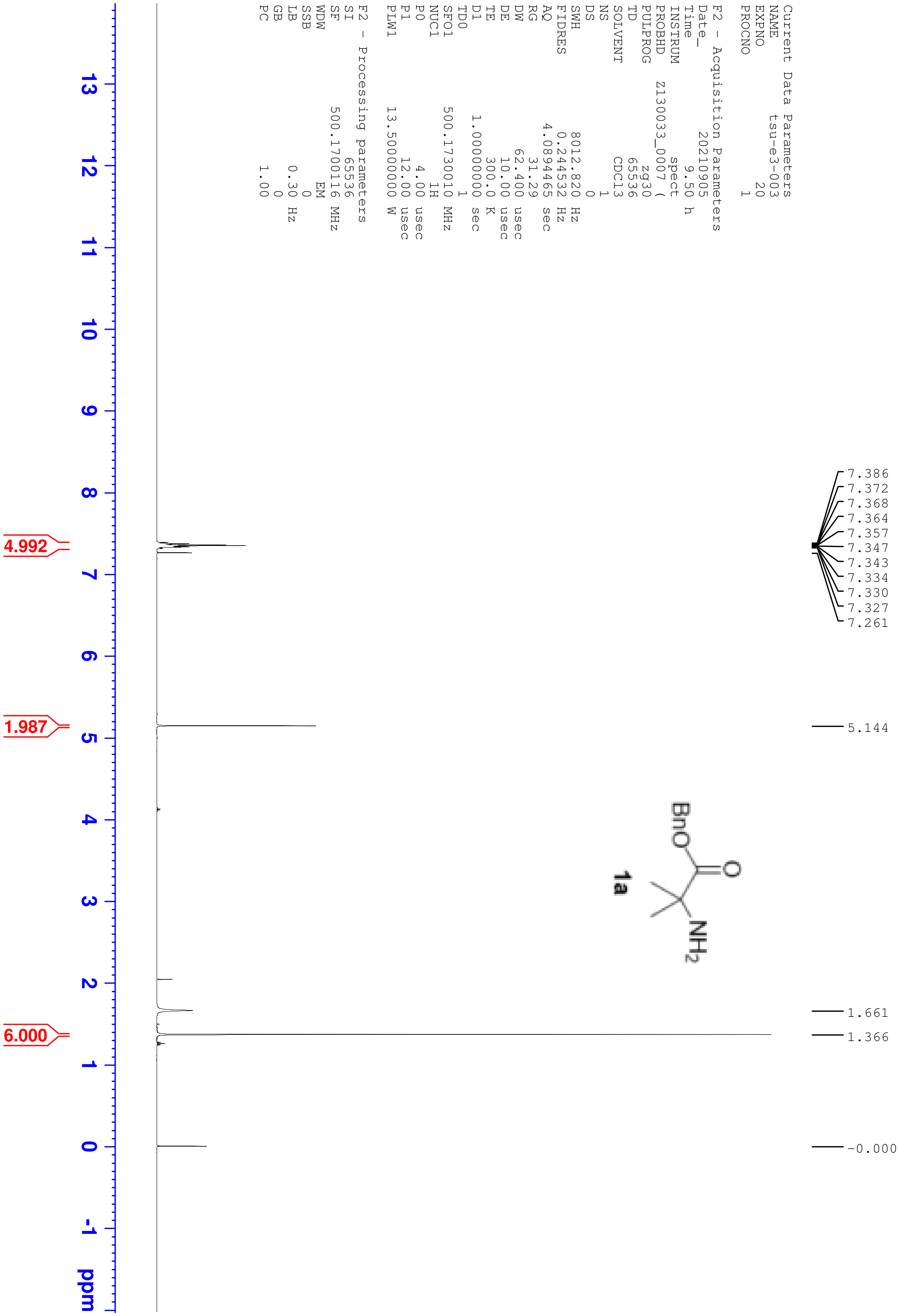

Current Data Parameters
NAME tsu-e3-003
EXPNO 20
PROCNO 1
F2 - Acquisition Parameters
Date_ 20210905
Time 9.50 h
INSTRUM spect
PROBHD Z130033_0007 (
PULPROG zg30
TD 65536
SOLVENT CDCl3
NS 1
DS 0
SWH 8012.820 Hz
FIDRES 0.244532 Hz
AQ 4.0894465 sec
RG 31.29
DW 62.400 usec
DE 10.00 usec
TE 300.0 K
D1 1.00000000 sec
TD0 1
SFO1 500.1730010 MHz
NUC1 1H
P0 4.00 usec
P1 12.00 usec
PLW1 13.50000000 W
F2 - Processing parameters
SI 65536
SF 500.1700116 MHz
WDW EM
SSB 0
LB 0.30 Hz
GB 0
PC 1.00
7.386
7.372
7.368
7.364
7.357
7.347
7.343
7.334
7.330
7.327
7.261
5.144
1.661
1.366
-0.000
O
BnO
NH2
1a
4.992
1.987
6.000
13
12
11
10
9
8
7
6
5
4
3
2
1
0
-1
ppm

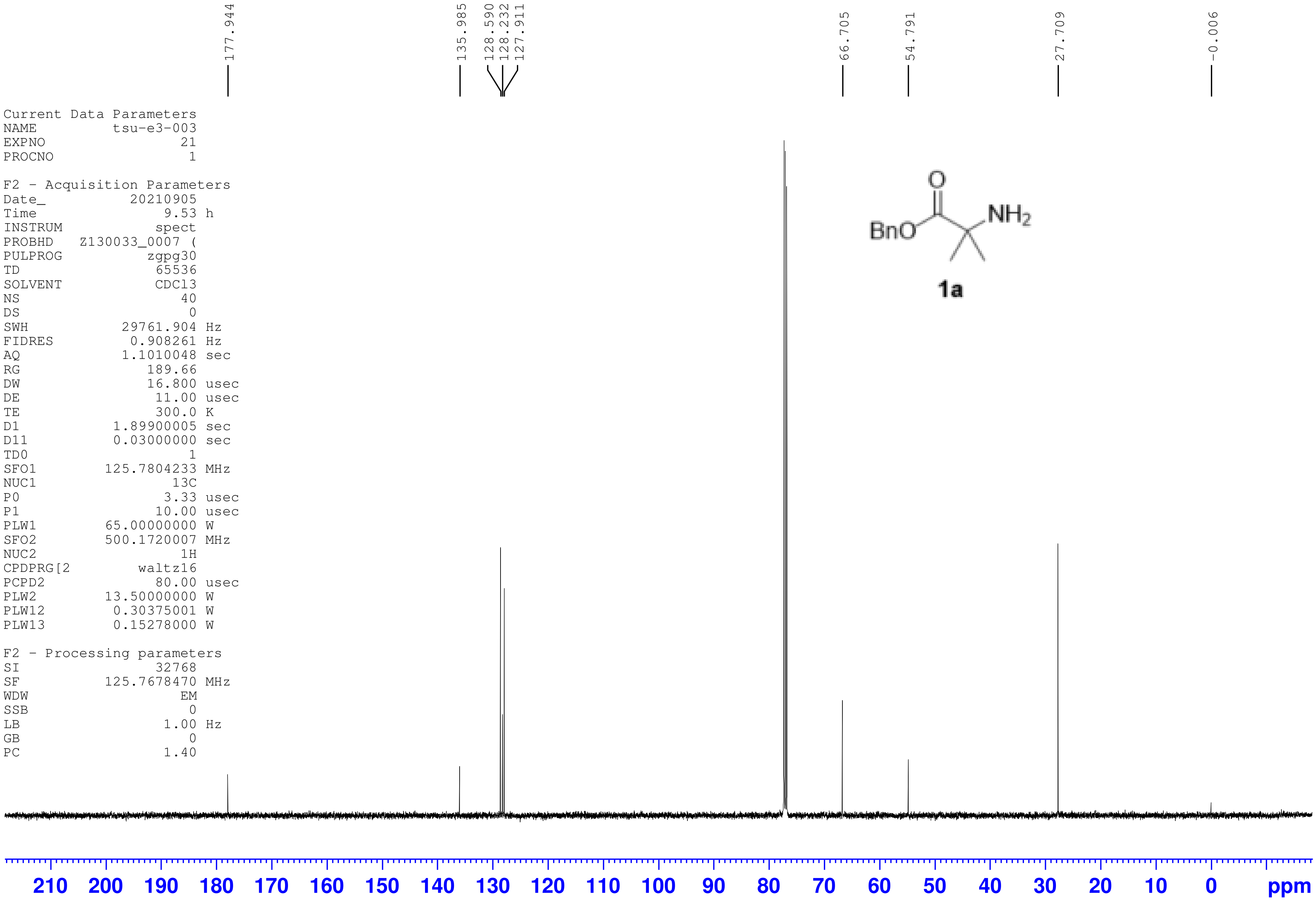
177.944
135.985
128.590
128.232
127.911
66.705
54.791
27.709
-0.006
Current Data Parameters
NAME tsu-e3-003
EXPNO 21
PROCNO 1
F2 - Acquisition Parameters
Date_ 20210905
Time 9.53 h
INSTRUM spect
PROBHD Z130033_0007 (
PULPROG zgpg30
TD 65536
SOLVENT CDCl3
NS 40
DS 0
SWH 29761.904 Hz
FIDRES 0.908261 Hz
AQ 1.1010048 sec
RG 189.66
DW 16.800 usec
DE 11.00 usec
TE 300.0 K
D1 1.89900005 sec
D11 0.03000000 sec
TD0 1
SFO1 125.7804233 MHz
NUC1 13C
P0 3.33 usec
P1 10.00 usec
PLW1 65.00000000 W
SFO2 500.1720007 MHz
NUC2 1H
CPDPRG[2 waltz16
PCPD2 80.00 usec
PLW2 13.50000000 W
PLW12 0.30375001 W
PLW13 0.15278000 W
F2 - Processing parameters
SI 32768
SF 125.7678470 MHz
WDW EM
SSB 0
LB 1.00 Hz
GB 0
PC 1.40
BnO
O
NH2
1a
210 200 190 180 170 160 150 140 130 120 110 100 90 80 70 60 50 40 30 20 10 0 ppm

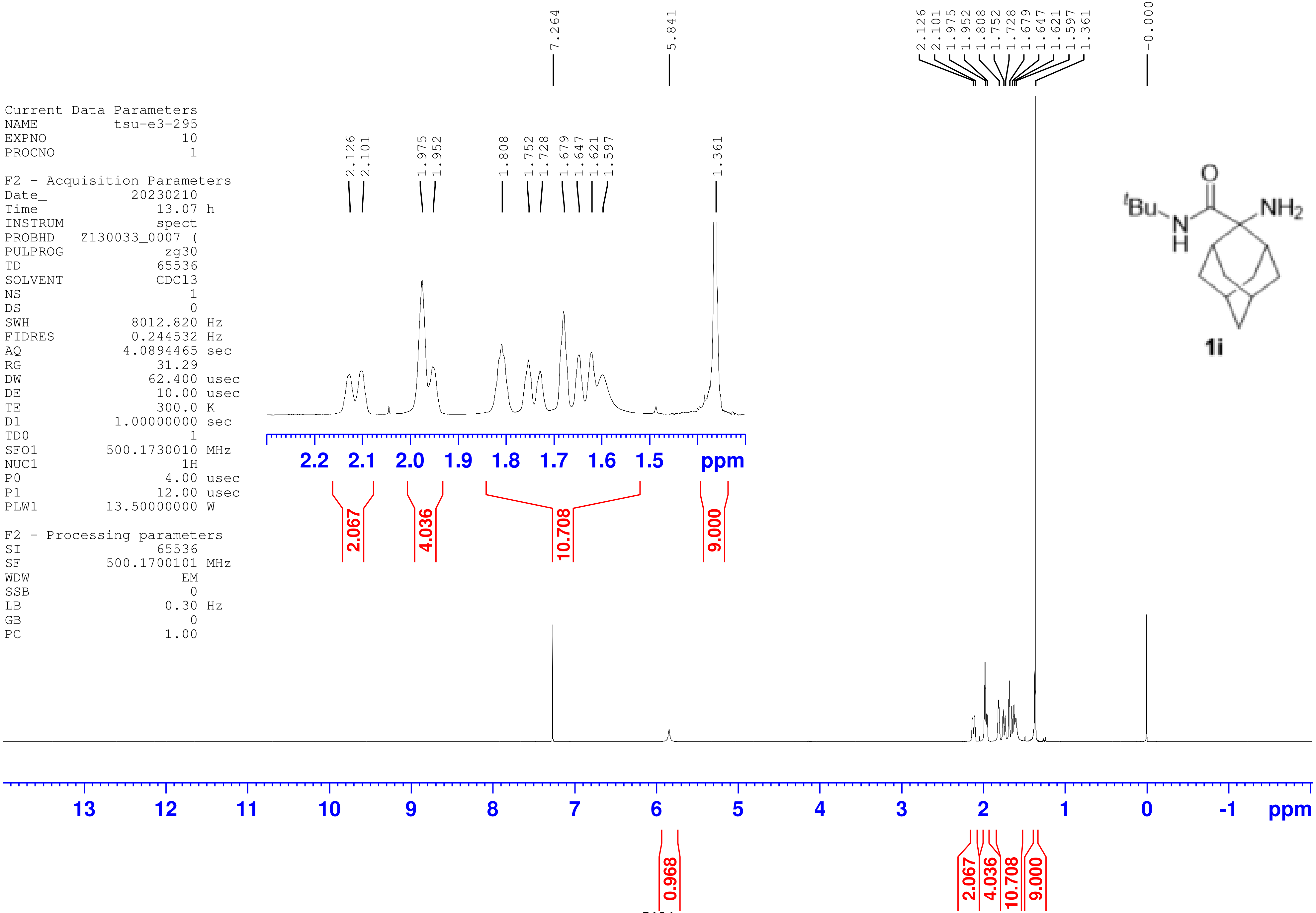

Current Data Parameters
NAME tsu-e3-295
EXPNO 10
PROCNO 1

F2 - Acquisition Parameters
Date_ 20230210
Time 13.07 h
INSTRUM spect
PROBHD Z130033_0007 (
PULPROG zg30
TD 65536
SOLVENT CDCl3
NS 1
DS 0
SWH 8012.820 Hz
FIDRES 0.244532 Hz
AQ 4.0894465 sec
RG 31.29
DW 62.400 usec
DE 10.00 usec
TE 300.0 K
D1 1.00000000 sec
TD0 1
SFO1 500.1730010 MHz
NUC1 1H
P0 4.00 usec
P1 12.00 usec
PLW1 13.50000000 W

F2 - Processing parameters
SI 65536
SF 500.1700101 MHz
WDW EM
SSB 0
LB 0.30 Hz
GB 0
PC 1.00

1i

Current Data Parameters
NAME tsu-e3-295
EXPNO 11
PROCNO 1

F2 - Acquisition Parameters
Date_ 20230210
Time 13.13 h
INSTRUM spect
PROBHD Z130033_0007 (
PULPROG zgpg30
TD 65536
SOLVENT CDCl3
NS 108
DS 0
SWH 29761.904 Hz
FIDRES 0.908261 Hz
AQ 1.1010048 sec
RG 189.66
DW 16.800 usec
DE 11.00 usec
TE 300.0 K
D1 1.89900005 sec
D11 0.03000000 sec
TD0 1
SFO1 125.7804228 MHz
NUC1 13C
P0 3.33 usec
P1 10.00 usec
PLW1 65.00000000 W
SFO2 500.1720007 MHz
NUC2 1H
CPDPRG[2 waltz16
PCPD2 80.00 usec
PLW2 13.50000000 W
PLW12 0.30375001 W
PLW13 0.15278000 W

F2 - Processing parameters
SI 32768
SF 125.7678470 MHz
WDW EM
SSB 0
LB 1.00 Hz
GB 0
PC 1.40

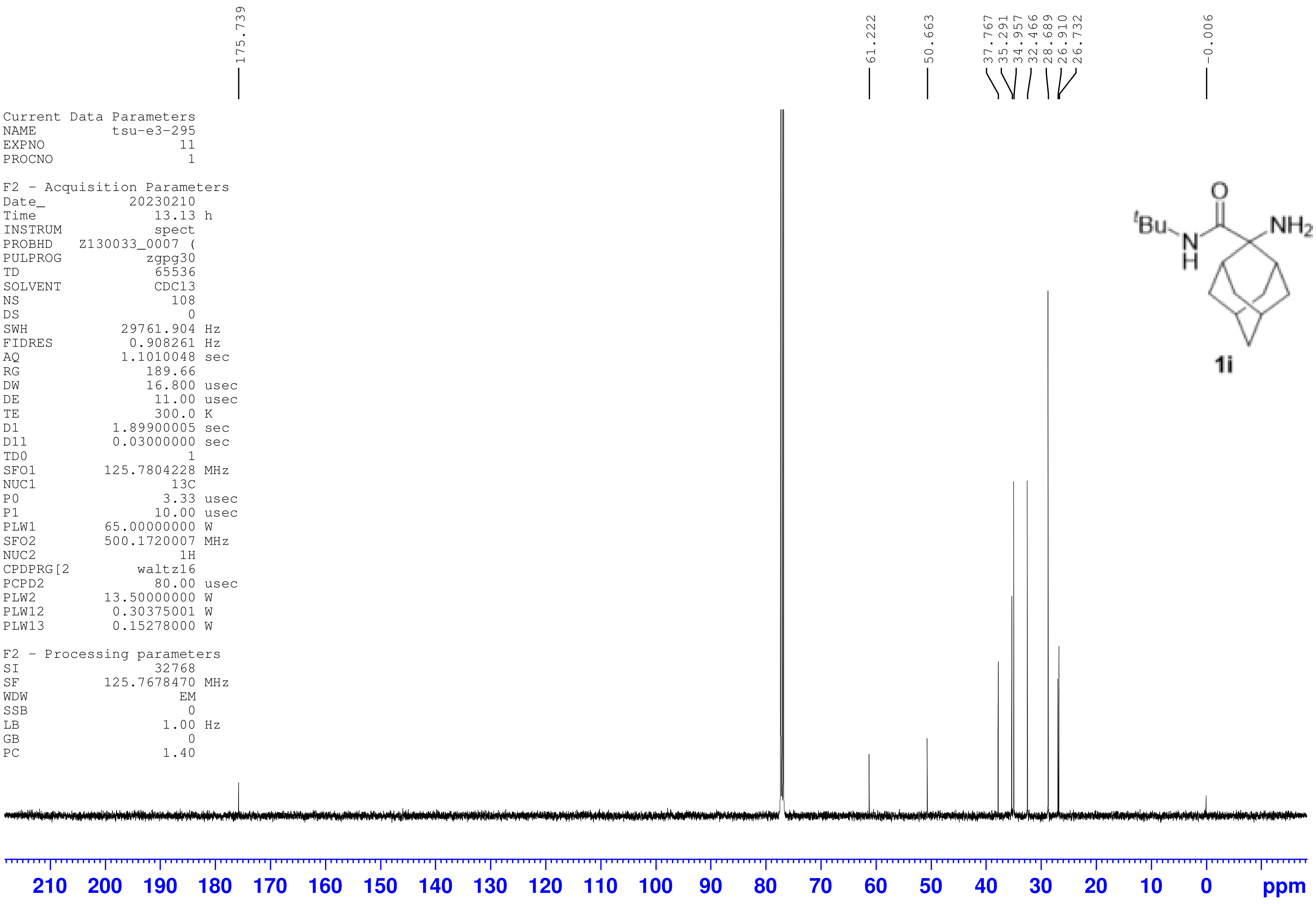

1i

```
Current Data Parameters
NAME              tsu-e3-329
EXPNO                     10
PROCNO                     1

F2 - Acquisition Parameters
Date_               20230304
Time                14.57 h
INSTRUM                spect
PROBHD    Z130033_0007 (
PULPROG                 zg30
TD                     65536
SOLVENT                CDCl3
NS                         1
DS                         0
SWH                 8012.820 Hz
FIDRES              0.244532 Hz
AQ                 4.0894465 sec
RG                     31.29
DW                    62.400 usec
DE                     10.00 usec
TE                     300.0 K
D1                1.00000000 sec
TD0                        1
SFO1             500.1730010 MHz
NUC1                      1H
P0                      4.00 usec
P1                     12.00 usec
PLW1             13.50000000 W

F2 - Processing parameters
SI                     65536
SF               500.1700096 MHz
WDW                       EM
SSB                        0
LB                      0.30 Hz
GB                         0
PC                      1.00
```

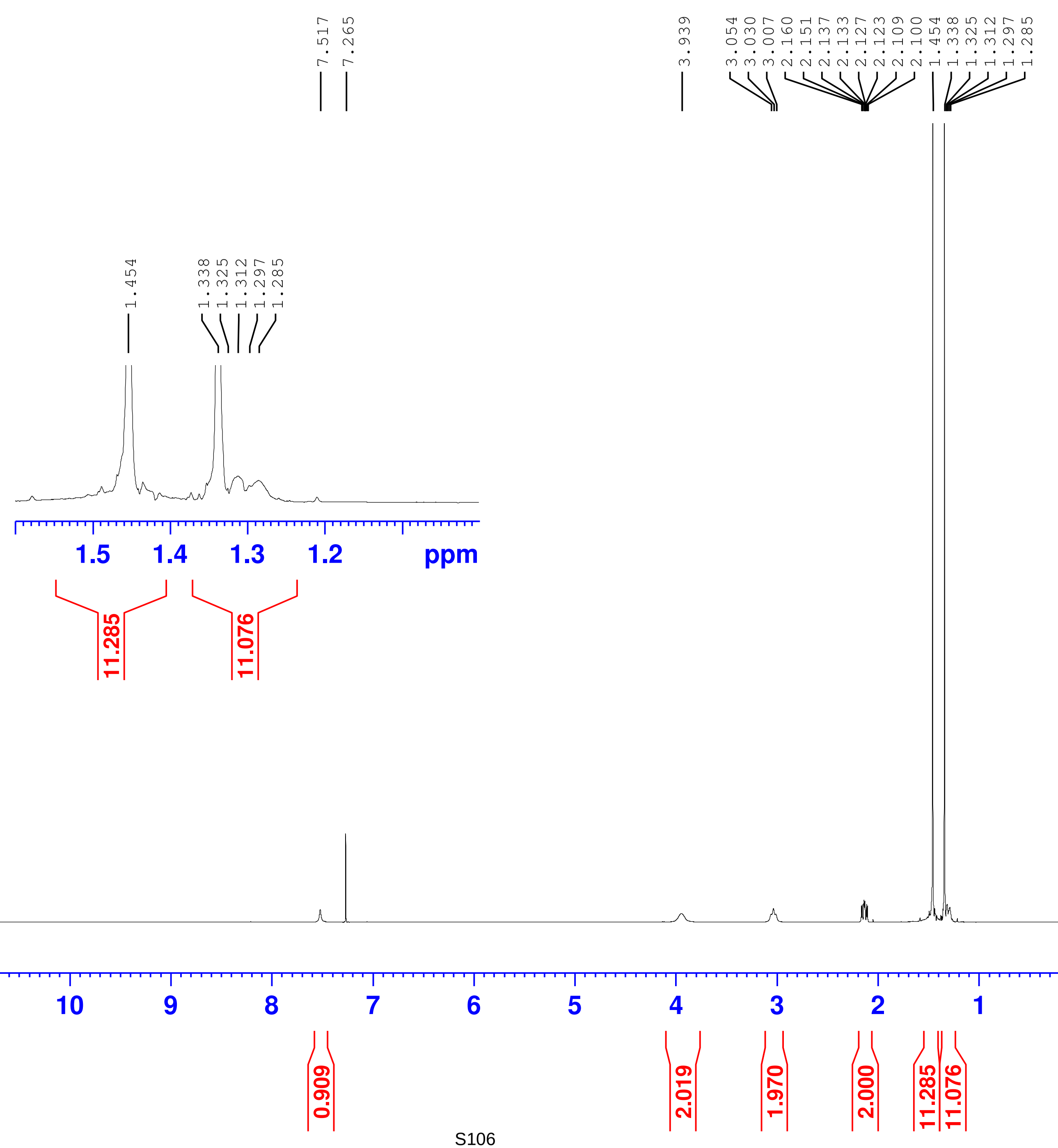

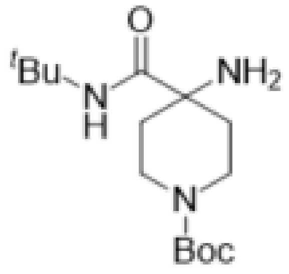

1j

S106

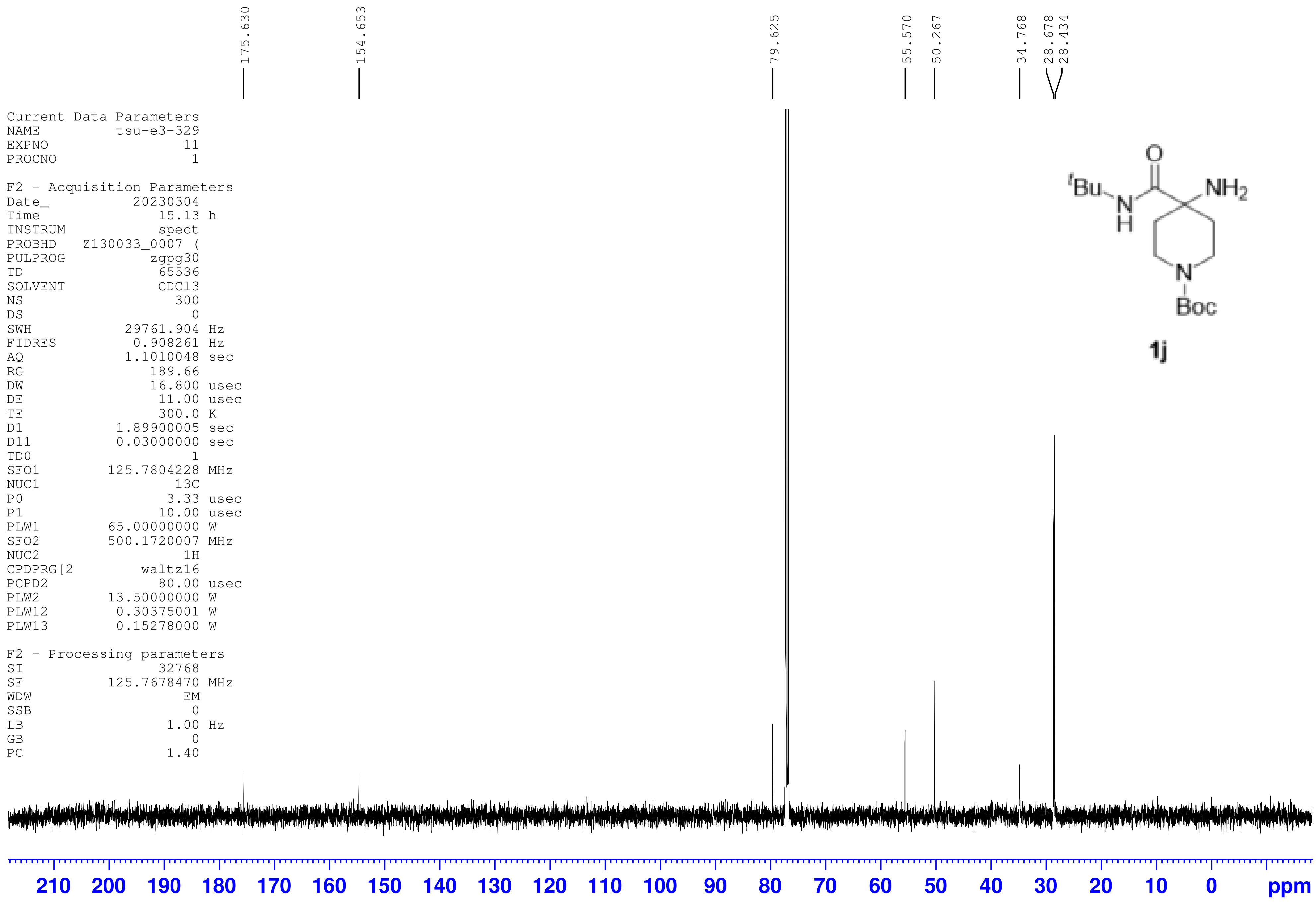
175.630
154.653
79.625
55.570
50.267
34.768
28.678
28.434
Current Data Parameters
NAME tsu-e3-329
EXPNO 11
PROCNO 1
F2 - Acquisition Parameters
Date_ 20230304
Time 15.13 h
INSTRUM spect
PROBHD Z130033_0007 (
PULPROG zgpg30
TD 65536
SOLVENT CDCl3
NS 300
DS 0
SWH 29761.904 Hz
FIDRES 0.908261 Hz
AQ 1.1010048 sec
RG 189.66
DW 16.800 usec
DE 11.00 usec
TE 300.0 K
D1 1.89900005 sec
D11 0.03000000 sec
TD0 1
SFO1 125.7804228 MHz
NUC1 13C
P0 3.33 usec
P1 10.00 usec
PLW1 65.00000000 W
SFO2 500.1720007 MHz
NUC2 1H
CPDPRG[2 waltz16
PCPD2 80.00 usec
PLW2 13.50000000 W
PLW12 0.30375001 W
PLW13 0.15278000 W
F2 - Processing parameters
SI 32768
SF 125.7678470 MHz
WDW EM
SSB 0
LB 1.00 Hz
GB 0
PC 1.40
tBu
N
H
O
NH2
N
Boc
1j
210 200 190 180 170 160 150 140 130 120 110 100 90 80 70 60 50 40 30 20 10 0 ppm

Current Data Parameters
NAME tsu-e3-335
EXPNO 10
PROCNO 1

F2 - Acquisition Parameters
Date_ 20230318
Time 14.37 h
INSTRUM spect
PROBHD Z119470_0344 (
PULPROG zg30
TD 65536
SOLVENT CDCl3
NS 1
DS 0
SWH 8012.820 Hz
FIDRES 0.244532 Hz
AQ 4.0894465 sec
RG 85.91
DW 62.400 usec
DE 6.50 usec
TE 298.0 K
D1 1.00000000 sec
TD0 1
SFO1 500.1730010 MHz
NUC1 1H
P0 4.83 usec
P1 14.50 usec
PLW1 10.80000019 W

F2 - Processing parameters
SI 65536
SF 500.1700085 MHz
WDW EM
SSB 0
LB 0.30 Hz
GB 0
PC 1.00

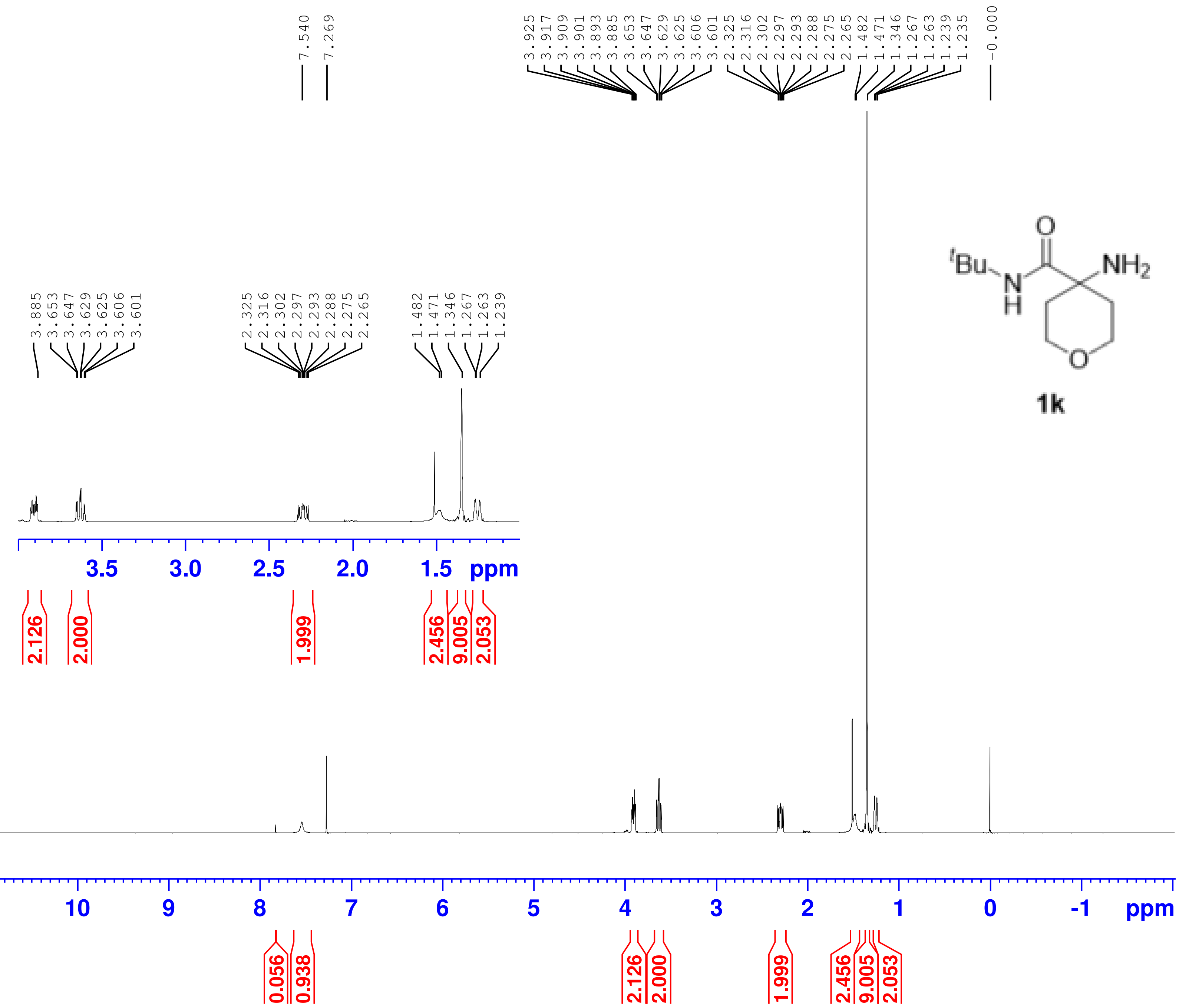

1k

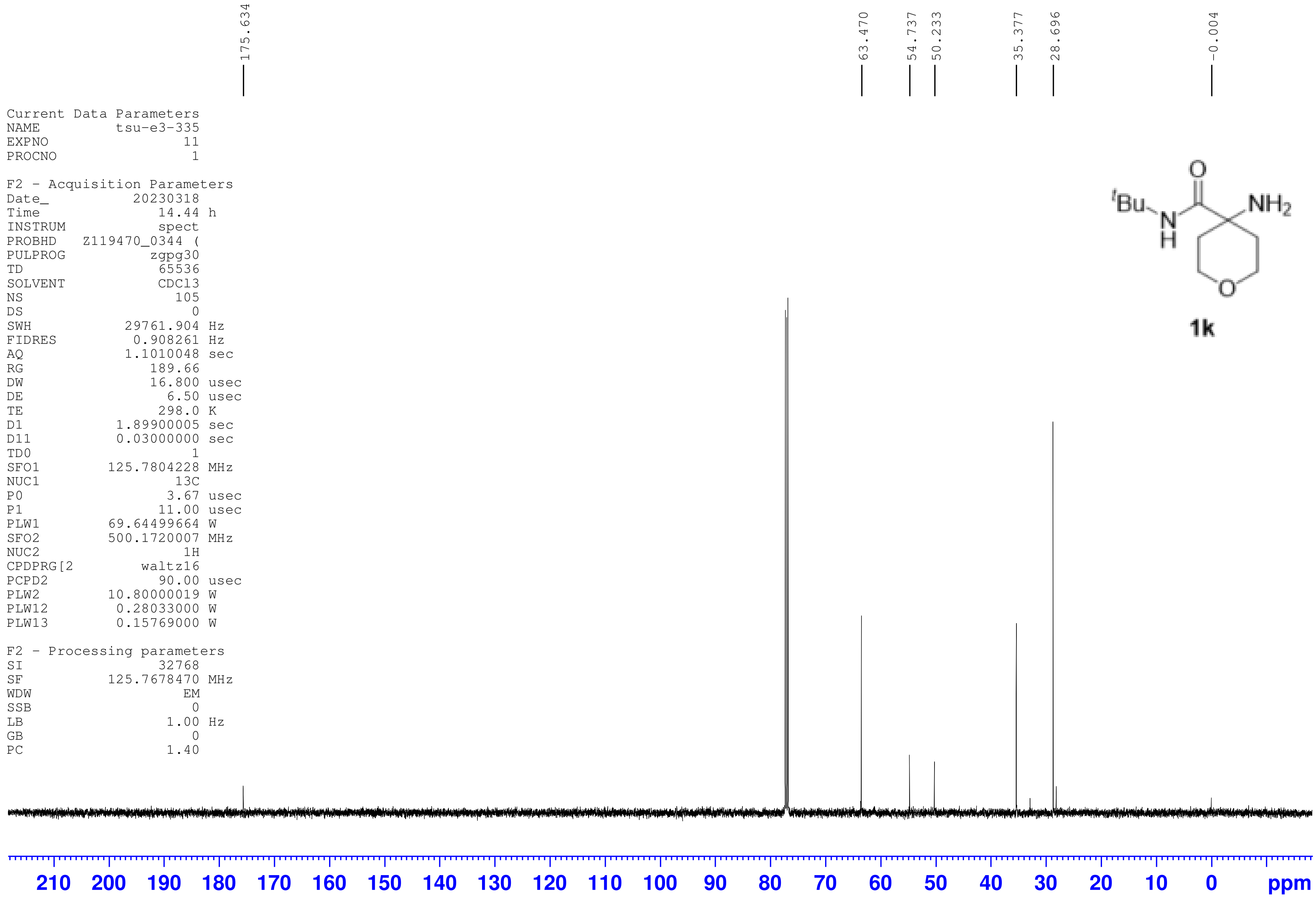
175.634
63.470
54.737
50.233
35.377
28.696
-0.004
Current Data Parameters
NAME tsu-e3-335
EXPNO 11
PROCNO 1
F2 - Acquisition Parameters
Date_ 20230318
Time 14.44 h
INSTRUM spect
PROBHD Z119470_0344 (
PULPROG zgpg30
TD 65536
SOLVENT CDCl3
NS 105
DS 0
SWH 29761.904 Hz
FIDRES 0.908261 Hz
AQ 1.1010048 sec
RG 189.66
DW 16.800 usec
DE 6.50 usec
TE 298.0 K
D1 1.89900005 sec
D11 0.03000000 sec
TD0 1
SFO1 125.7804228 MHz
NUC1 13C
P0 3.67 usec
P1 11.00 usec
PLW1 69.64499664 W
SFO2 500.1720007 MHz
NUC2 1H
CPDPRG[2 waltz16
PCPD2 90.00 usec
PLW2 10.80000019 W
PLW12 0.28033000 W
PLW13 0.15769000 W
F2 - Processing parameters
SI 32768
SF 125.7678470 MHz
WDW EM
SSB 0
LB 1.00 Hz
GB 0
PC 1.40
210 200 190 180 170 160 150 140 130 120 110 100 90 80 70 60 50 40 30 20 10 0 ppm
tBu
N
H
O
NH2
O

1k

```
Current Data Parameters
NAME          tsu-e3-336
EXPNO                 10
PROCNO                 1

F2 - Acquisition Parameters
Date_           20230318
Time               14.47 h
INSTRUM            spect
PROBHD    Z119470_0344 (
PULPROG             zg30
TD                 65536
SOLVENT            CDCl3
NS                     1
DS                     0
SWH             8012.820 Hz
FIDRES          0.244532 Hz
AQ             4.0894465 sec
RG                 94.41
DW                62.400 usec
DE                  6.50 usec
TE                 297.9 K
D1            1.00000000 sec
TD0                    1
SFO1         500.1730010 MHz
NUC1                  1H
P0                  4.83 usec
P1                 14.50 usec
PLW1         10.80000019 W

F2 - Processing parameters
SI                 65536
SF           500.1700085 MHz
WDW                   EM
SSB                    0
LB                  0.30 Hz
GB                     0
PC                  1.00
```

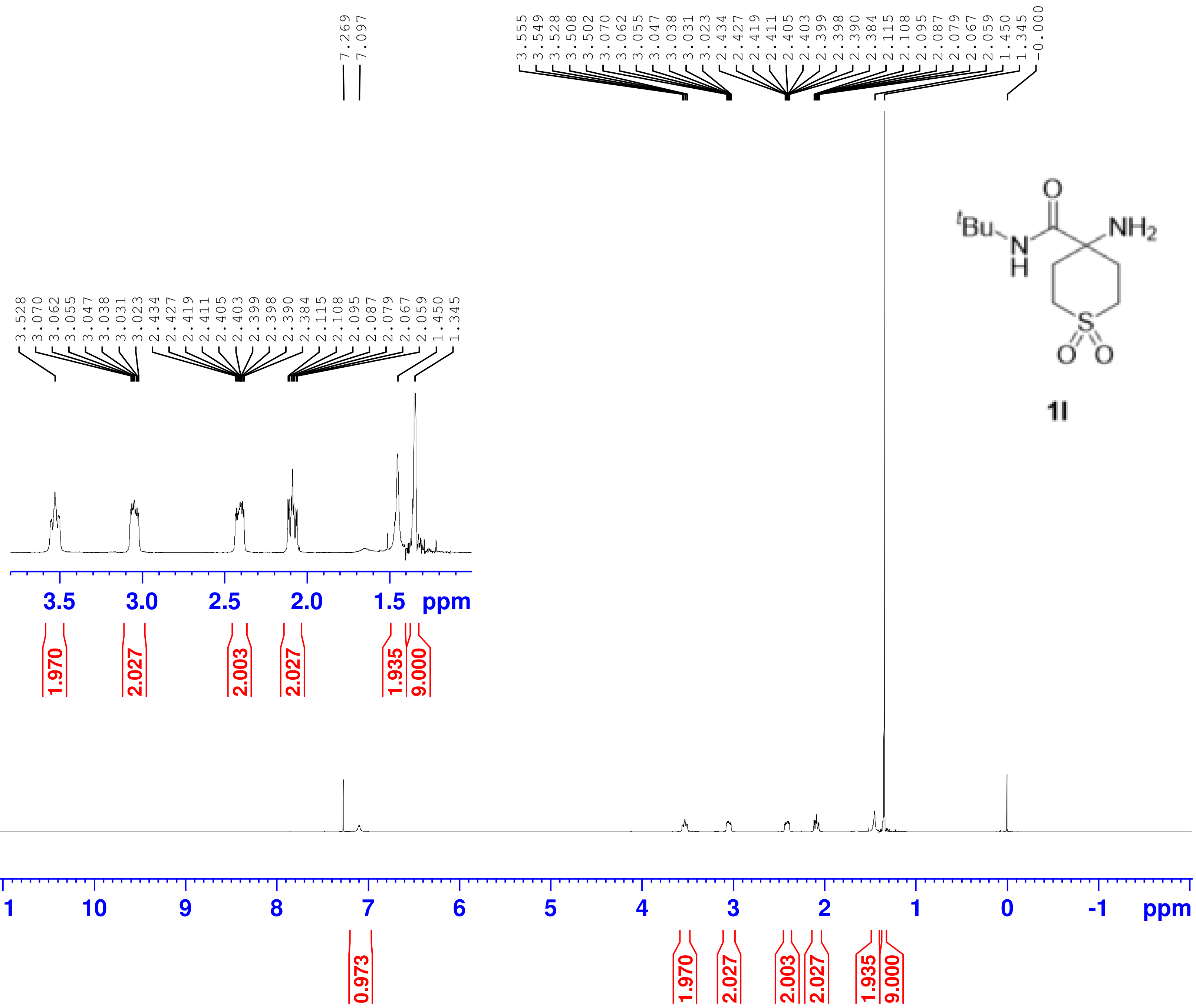

1l

S110

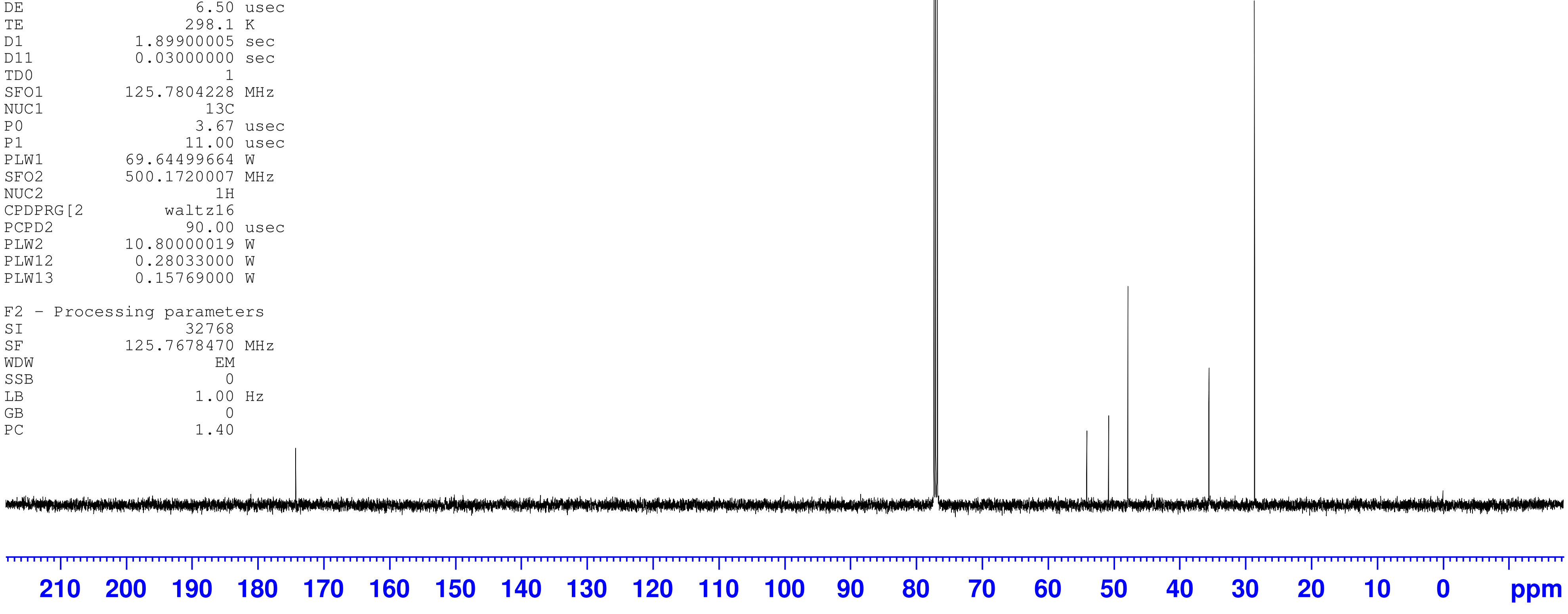

Current Data Parameters

NAME	tsu-e3-336
EXPNO	11
PROCNO	1

F2 - Acquisition Parameters

Date_	20230318
Time	14.54 h
INSTRUM	spect
PROBHD	Z119470_0344 (
PULPROG	zgpg30
TD	65536
SOLVENT	CDCl3
NS	102
DS	0
SWH	29761.904 Hz
FIDRES	0.908261 Hz
AQ	1.1010048 sec
RG	189.66
DW	16.800 usec
DE	6.50 usec
TE	298.1 K
D1	1.89900005 sec
D11	0.03000000 sec
TD0	1
SFO1	125.7804228 MHz
NUC1	13C
P0	3.67 usec
P1	11.00 usec
PLW1	69.64499664 W
SFO2	500.1720007 MHz
NUC2	1H
CPDPRG[2	waltz16
PCPD2	90.00 usec
PLW2	10.80000019 W
PLW12	0.28033000 W
PLW13	0.15769000 W

F2 - Processing parameters

SI	32768
SF	125.7678470 MHz
WDW	EM
SSB	0
LB	1.00 Hz
GB	0
PC	1.40

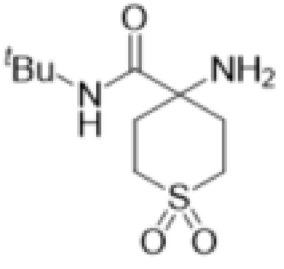

1l

Current Data Parameters
NAME tsu-e3-363
EXPNO 20
PROCNO 1

F2 - Acquisition Parameters
Date_ 20230410
Time 9.16 h
INSTRUM spect
PROBHD Z119470_0344 (
PULPROG zg30
TD 65536
SOLVENT CDCl3
NS 1
DS 0
SWH 8012.820 Hz
FIDRES 0.244532 Hz
AQ 4.0894465 sec
RG 116.65
DW 62.400 usec
DE 6.50 usec
TE 297.9 K
D1 1.00000000 sec
TD0 1
SFO1 500.1730010 MHz
NUC1 1H
P0 4.83 usec
P1 14.50 usec
PLW1 10.80000019 W

F2 - Processing parameters
SI 65536
SF 500.1700128 MHz
WDW EM
SSB 0
LB 0.30 Hz
GB 0
PC 1.00

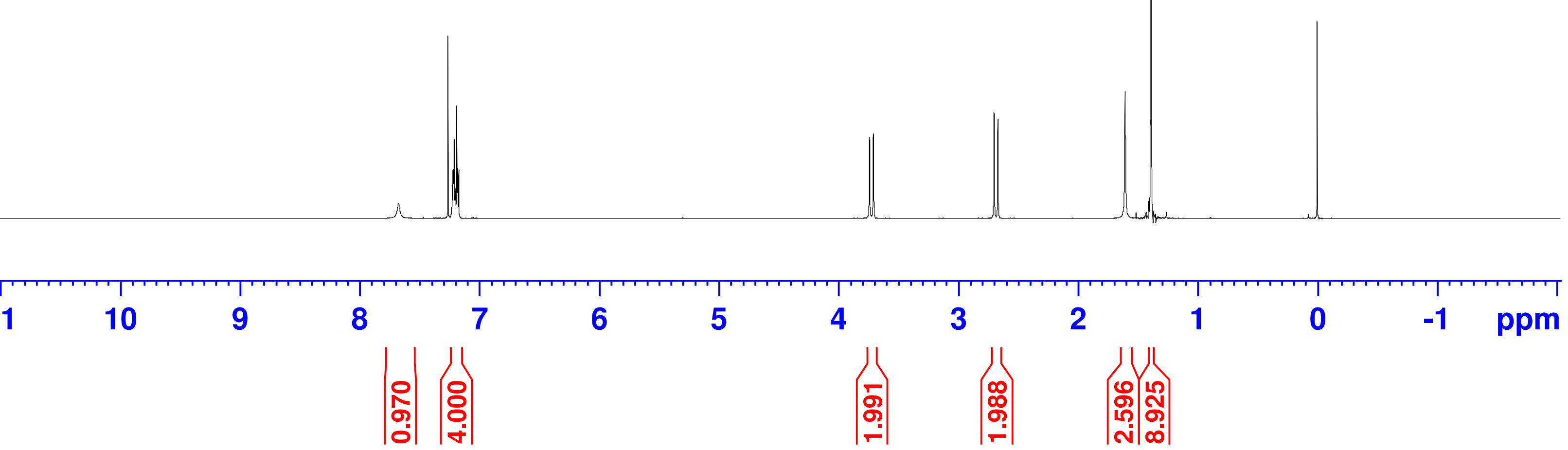

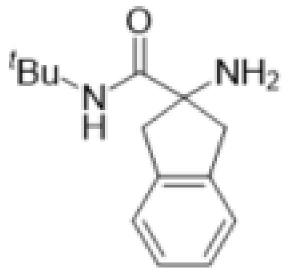

1m

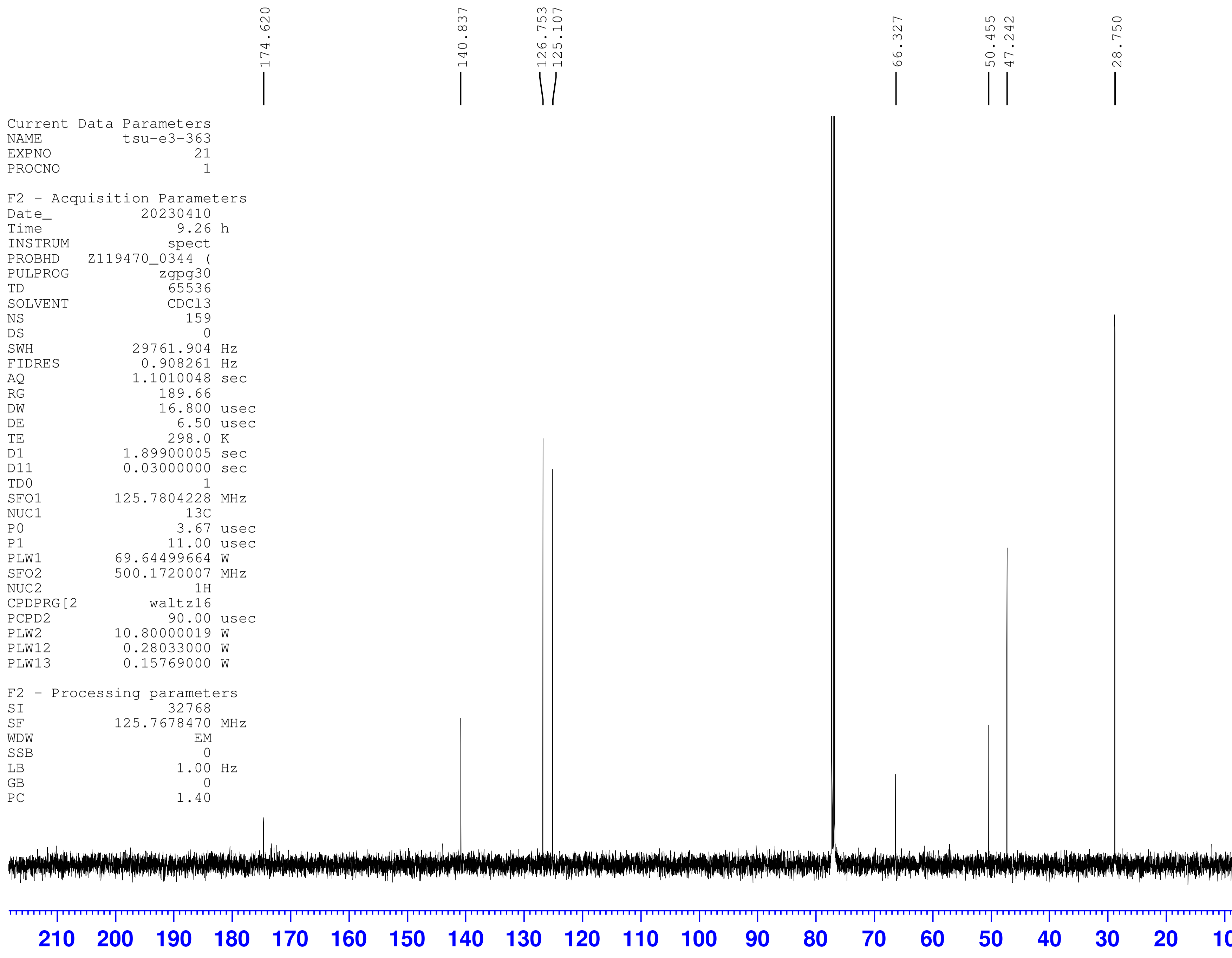
174.620
140.837
126.753
125.107
66.327
50.455
47.242
28.750
0.004
Current Data Parameters
NAME tsu-e3-363
EXPNO 21
PROCNO 1
F2 - Acquisition Parameters
Date_ 20230410
Time 9.26 h
INSTRUM spect
PROBHD Z119470_0344 (
PULPROG zgpg30
TD 65536
SOLVENT CDCl3
NS 159
DS 0
SWH 29761.904 Hz
FIDRES 0.908261 Hz
AQ 1.1010048 sec
RG 189.66
DW 16.800 usec
DE 6.50 usec
TE 298.0 K
D1 1.89900005 sec
D11 0.03000000 sec
TD0 1
SFO1 125.7804228 MHz
NUC1 13C
P0 3.67 usec
P1 11.00 usec
PLW1 69.64499664 W
SFO2 500.1720007 MHz
NUC2 1H
CPDPRG[2 waltz16
PCPD2 90.00 usec
PLW2 10.80000019 W
PLW12 0.28033000 W
PLW13 0.15769000 W
F2 - Processing parameters
SI 32768
SF 125.7678470 MHz
WDW EM
SSB 0
LB 1.00 Hz
GB 0
PC 1.40
210 200 190 180 170 160 150 140 130 120 110 100 90 80 70 60 50 40 30 20 10 0 ppm

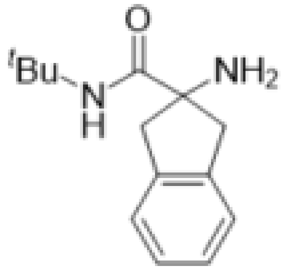
tBu
N
H
O
NH2

1m

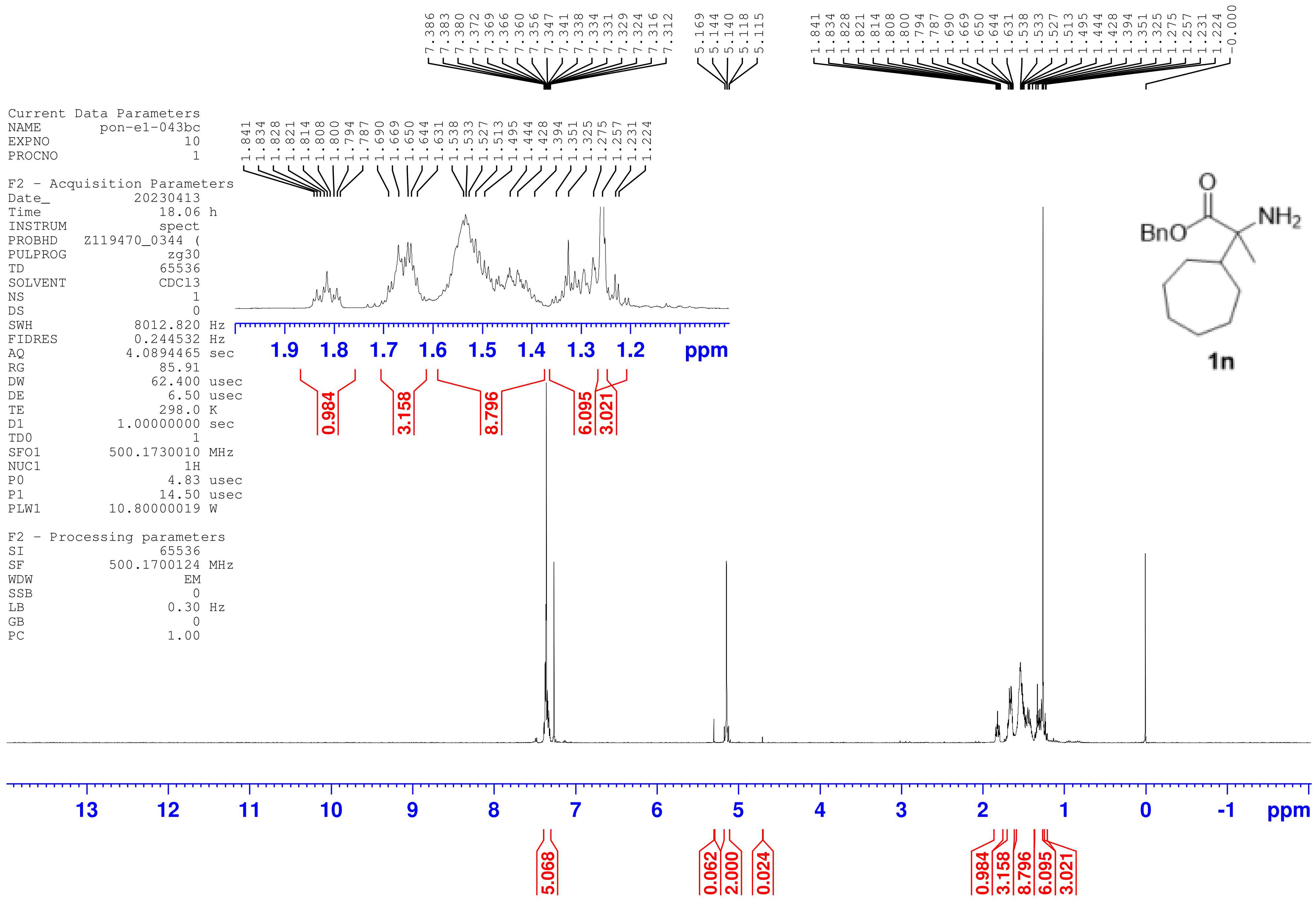
Current Data Parameters
NAME pon-e1-043bc
EXPNO 10
PROCNO 1
F2 - Acquisition Parameters
Date_ 20230413
Time 18.06 h
INSTRUM spect
PROBHD Z119470_0344 (
PULPROG zg30
TD 65536
SOLVENT CDCl3
NS 1
DS 0
SWH 8012.820 Hz
FIDRES 0.244532 Hz
AQ 4.0894465 sec
RG 85.91
DW 62.400 usec
DE 6.50 usec
TE 298.0 K
D1 1.00000000 sec
TD0 1
SFO1 500.1730010 MHz
NUC1 1H
P0 4.83 usec
P1 14.50 usec
PLW1 10.80000019 W
F2 - Processing parameters
SI 65536
SF 500.1700124 MHz
WDW EM
SSB 0
LB 0.30 Hz
GB 0
PC 1.00
BnO
NH2
1n
ppm

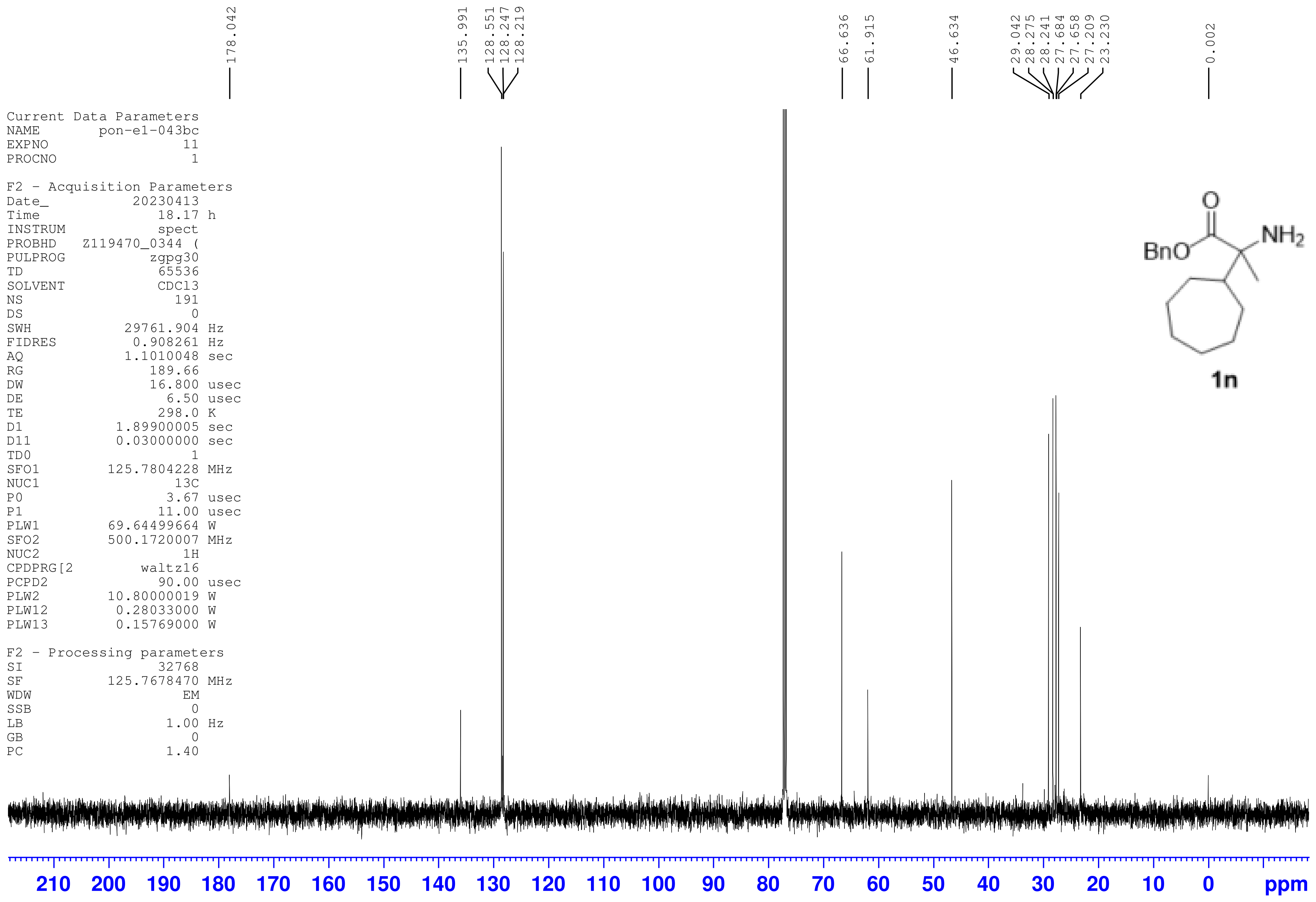
178.042
135.991
128.551
128.247
128.219
66.636
61.915
46.634
29.042
28.275
28.241
27.684
27.658
27.209
23.230
0.002
Current Data Parameters
NAME pon-e1-043bc
EXPNO 11
PROCNO 1
F2 - Acquisition Parameters
Date_ 20230413
Time 18.17 h
INSTRUM spect
PROBHD Z119470_0344 (
PULPROG zgpg30
TD 65536
SOLVENT CDCl3
NS 191
DS 0
SWH 29761.904 Hz
FIDRES 0.908261 Hz
AQ 1.1010048 sec
RG 189.66
DW 16.800 usec
DE 6.50 usec
TE 298.0 K
D1 1.89900005 sec
D11 0.03000000 sec
TD0 1
SFO1 125.7804228 MHz
NUC1 13C
P0 3.67 usec
P1 11.00 usec
PLW1 69.64499664 W
SFO2 500.1720007 MHz
NUC2 1H
CPDPRG[2 waltz16
PCPD2 90.00 usec
PLW2 10.80000019 W
PLW12 0.28033000 W
PLW13 0.15769000 W
F2 - Processing parameters
SI 32768
SF 125.7678470 MHz
WDW EM
SSB 0
LB 1.00 Hz
GB 0
PC 1.40
210 200 190 180 170 160 150 140 130 120 110 100 90 80 70 60 50 40 30 20 10 0 ppm
BnO
O
NH2

1n

Current Data Parameters
NAME tsu-e3-408
EXPNO 30
PROCNO 1

F2 - Acquisition Parameters
Date_ 20230527
Time 12.05 h
INSTRUM spect
PROBHD Z119470_0344 (
PULPROG zg30
TD 65536
SOLVENT CDCl3
NS 1
DS 0
SWH 8012.820 Hz
FIDRES 0.244532 Hz
AQ 4.0894465 sec
RG 116.65
DW 62.400 usec
DE 6.50 usec
TE 298.0 K
D1 1.00000000 sec
TD0 1
SFO1 500.1730010 MHz
NUC1 1H
P0 4.83 usec
P1 14.50 usec
PLW1 10.80000019 W

F2 - Processing parameters
SI 65536
SF 500.1700123 MHz
WDW EM
SSB 0
LB 0.30 Hz
GB 0
PC 1.00

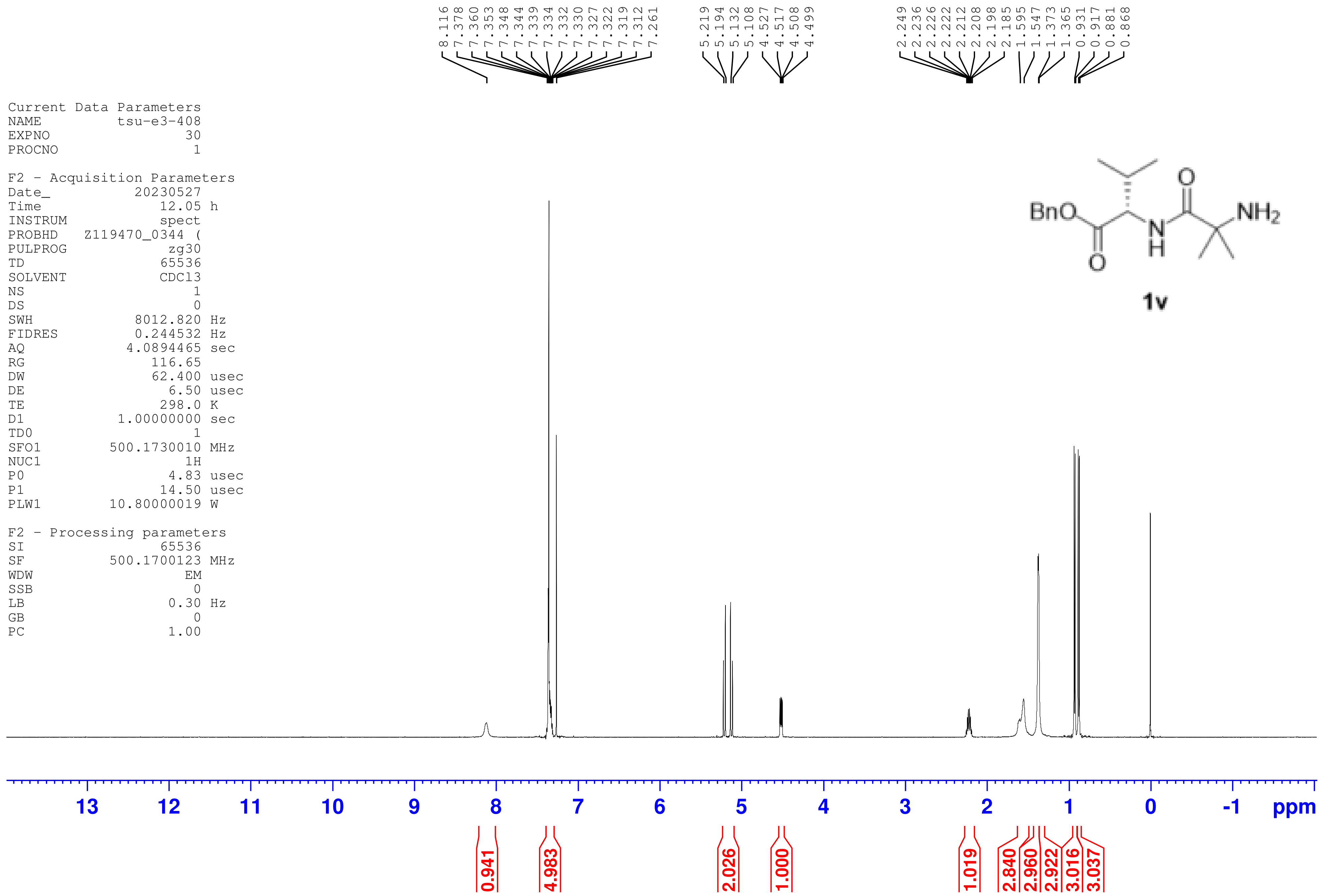

1v

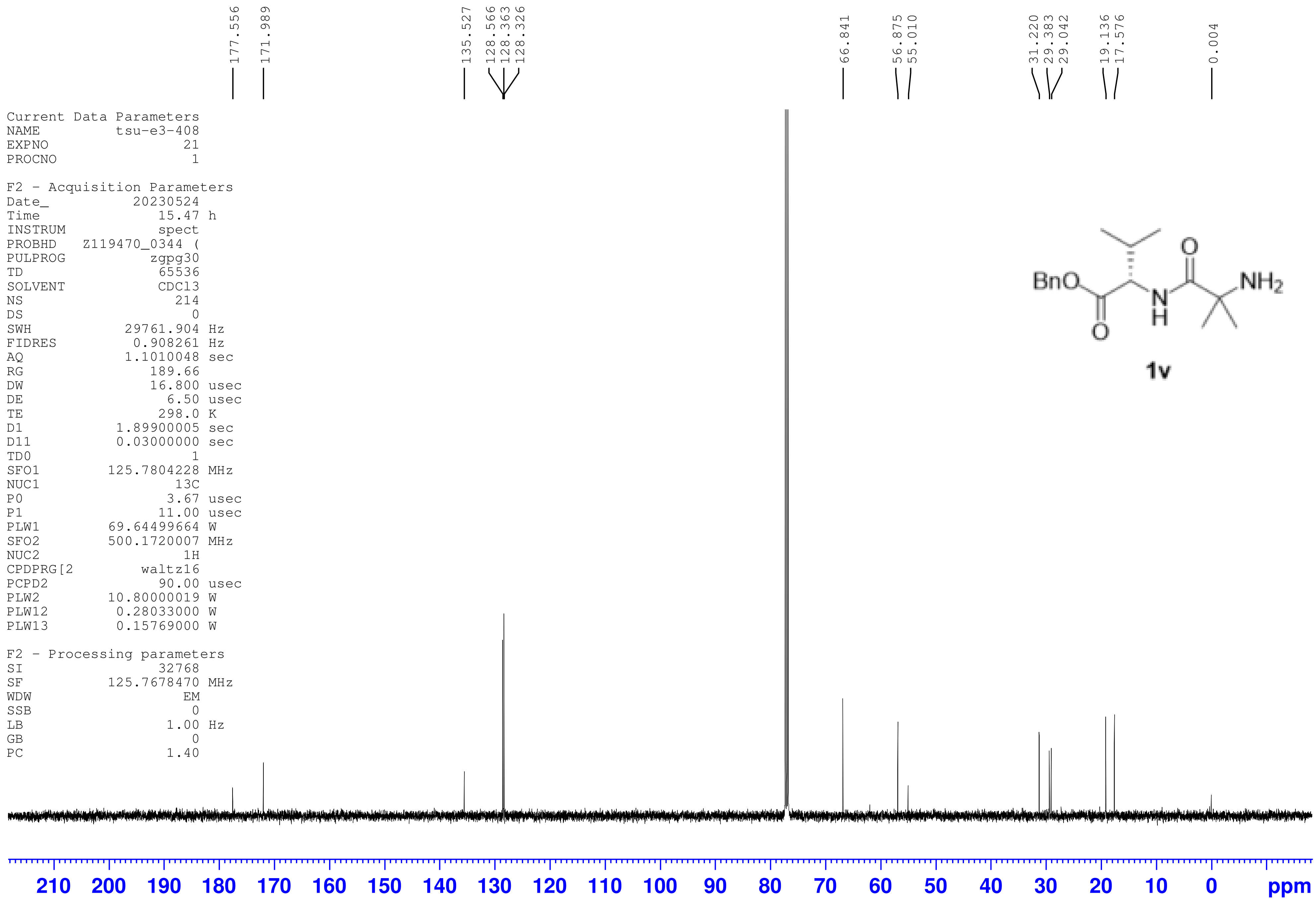
177.556
171.989
135.527
128.566
128.363
128.326
66.841
56.875
55.010
31.220
29.383
29.042
19.136
17.576
0.004
Current Data Parameters
NAME tsu-e3-408
EXPNO 21
PROCNO 1
F2 - Acquisition Parameters
Date_ 20230524
Time 15.47 h
INSTRUM spect
PROBHD Z119470_0344 (
PULPROG zgpg30
TD 65536
SOLVENT CDCl3
NS 214
DS 0
SWH 29761.904 Hz
FIDRES 0.908261 Hz
AQ 1.1010048 sec
RG 189.66
DW 16.800 usec
DE 6.50 usec
TE 298.0 K
D1 1.89900005 sec
D11 0.03000000 sec
TD0 1
SFO1 125.7804228 MHz
NUC1 13C
P0 3.67 usec
P1 11.00 usec
PLW1 69.64499664 W
SFO2 500.1720007 MHz
NUC2 1H
CPDPRG[2 waltz16
PCPD2 90.00 usec
PLW2 10.80000019 W
PLW12 0.28033000 W
PLW13 0.15769000 W
F2 - Processing parameters
SI 32768
SF 125.7678470 MHz
WDW EM
SSB 0
LB 1.00 Hz
GB 0
PC 1.40
BnO
O
N
H
O
NH2
1v
210 200 190 180 170 160 150 140 130 120 110 100 90 80 70 60 50 40 30 20 10 0 ppm

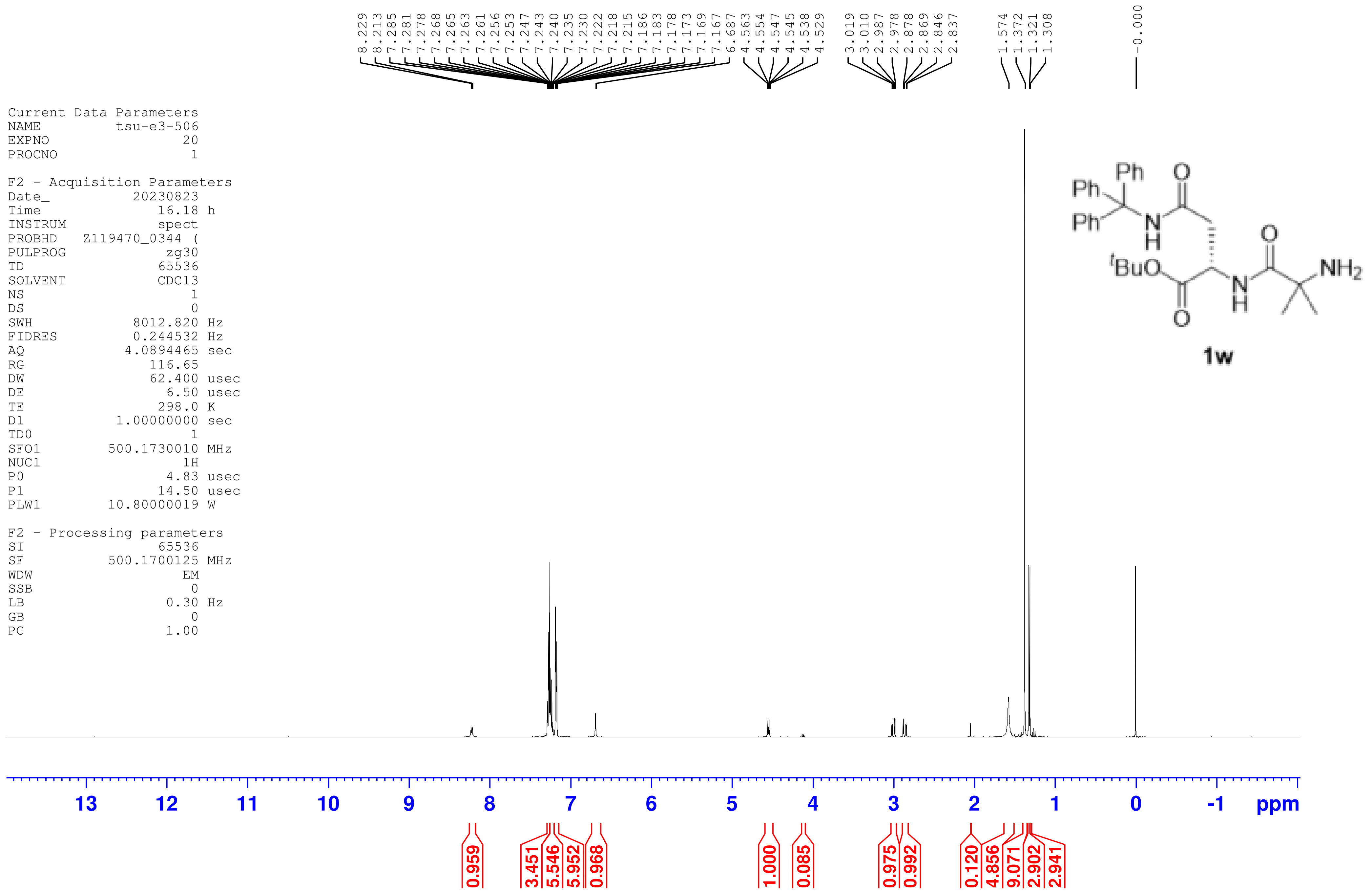
Current Data Parameters
NAME tsu-e3-506
EXPNO 20
PROCNO 1
F2 - Acquisition Parameters
Date_ 20230823
Time 16.18 h
INSTRUM spect
PROBHD Z119470_0344 (
PULPROG zg30
TD 65536
SOLVENT CDCl3
NS 1
DS 0
SWH 8012.820 Hz
FIDRES 0.244532 Hz
AQ 4.0894465 sec
RG 116.65
DW 62.400 usec
DE 6.50 usec
TE 298.0 K
D1 1.00000000 sec
TD0 1
SFO1 500.1730010 MHz
NUC1 1H
P0 4.83 usec
P1 14.50 usec
PLW1 10.80000019 W
F2 - Processing parameters
SI 65536
SF 500.1700125 MHz
WDW EM
SSB 0
LB 0.30 Hz
GB 0
PC 1.00
8.229 8.213 7.285 7.281 7.278 7.268 7.265 7.263 7.261 7.256 7.253 7.247 7.243 7.240 7.235 7.230 7.222 7.218 7.215 7.186 7.183 7.178 7.173 7.169 7.167 6.687 4.563 4.554 4.547 4.545 4.538 4.529 3.019 3.010 2.987 2.978 2.878 2.869 2.846 2.837 1.574 1.372 1.321 1.308 -0.000
0.959 3.451 5.546 5.952 0.968 1.000 0.085 0.975 0.992 0.120 4.856 9.071 2.902 2.941
13 12 11 10 9 8 7 6 5 4 3 2 1 0 -1 ppm
Ph
Ph
Ph
O
N
H
O
NH2
tBuO
N
H
O

1w

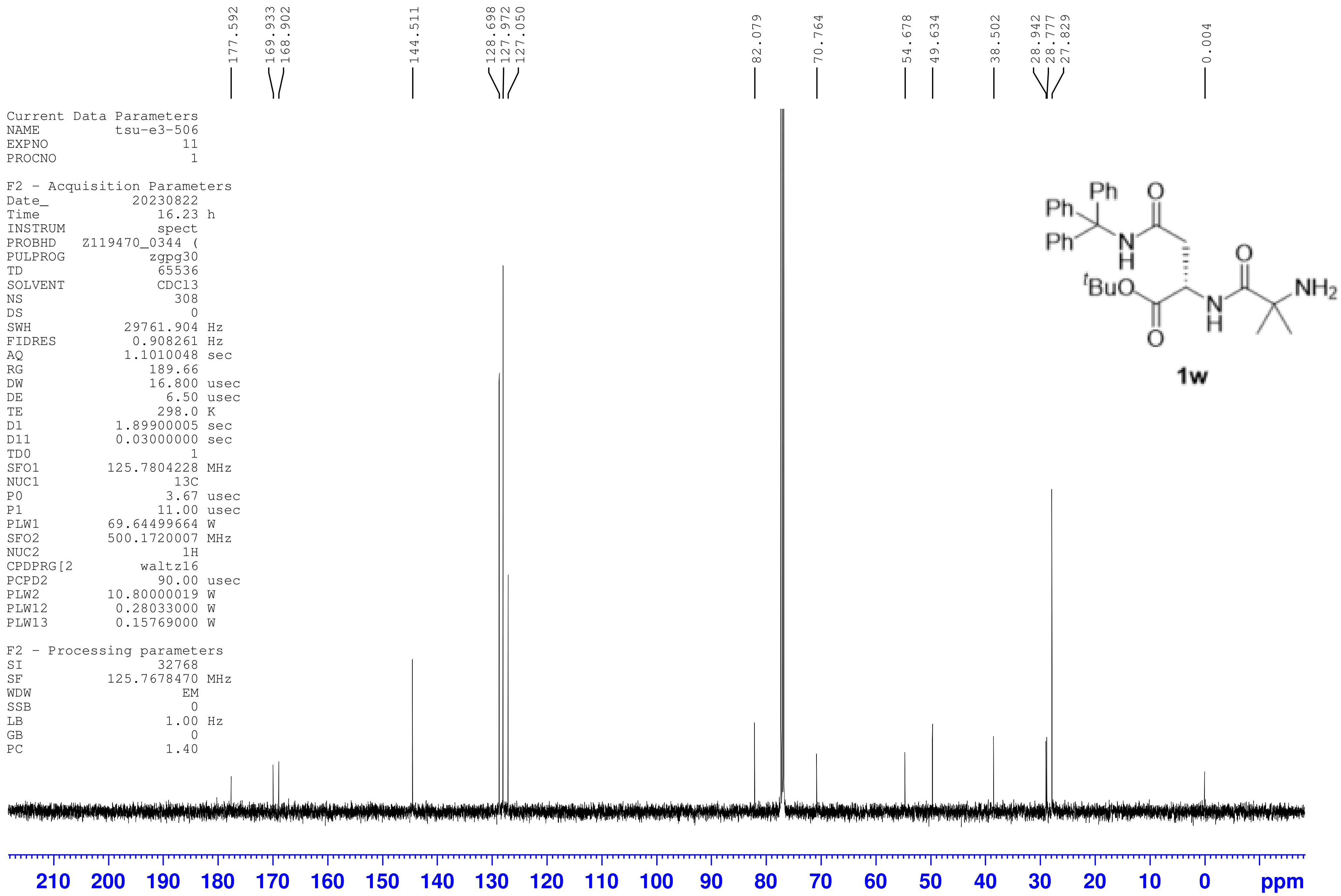
177.592
169.933
168.902
144.511
128.698
127.972
127.050
82.079
70.764
54.678
49.634
38.502
28.942
28.777
27.829
0.004
Current Data Parameters
NAME tsu-e3-506
EXPNO 11
PROCNO 1
F2 - Acquisition Parameters
Date_ 20230822
Time 16.23 h
INSTRUM spect
PROBHD Z119470_0344 (
PULPROG zgpg30
TD 65536
SOLVENT CDCl3
NS 308
DS 0
SWH 29761.904 Hz
FIDRES 0.908261 Hz
AQ 1.1010048 sec
RG 189.66
DW 16.800 usec
DE 6.50 usec
TE 298.0 K
D1 1.89900005 sec
D11 0.03000000 sec
TD0 1
SFO1 125.7804228 MHz
NUC1 13C
P0 3.67 usec
P1 11.00 usec
PLW1 69.64499664 W
SFO2 500.1720007 MHz
NUC2 1H
CPDPRG[2 waltz16
PCPD2 90.00 usec
PLW2 10.80000019 W
PLW12 0.28033000 W
PLW13 0.15769000 W
F2 - Processing parameters
SI 32768
SF 125.7678470 MHz
WDW EM
SSB 0
LB 1.00 Hz
GB 0
PC 1.40
210 200 190 180 170 160 150 140 130 120 110 100 90 80 70 60 50 40 30 20 10 0 ppm
Ph
Ph
Ph
O
N
H
O
^{t}BuO
NH$_2$
N
H
O

1w

```
Current Data Parameters
NAME          tsu-e3-231
EXPNO                 20
PROCNO                 1

F2 - Acquisition Parameters
Date_           20221219
Time               15.05 h
INSTRUM            spect
PROBHD   Z119470_0097 (
PULPROG             zg30
TD                 65536
SOLVENT            CDCl3
NS                     1
DS                     0
SWH             8012.820 Hz
FIDRES          0.244532 Hz
AQ             4.0894465 sec
RG                116.65
DW                62.400 usec
DE                  6.50 usec
TE                 300.0 K
D1            1.00000000 sec
TD0                    1
SFO1         500.1730010 MHz
NUC1                  1H
P0                  4.00 usec
P1                 12.00 usec
PLW1         17.00000000 W

F2 - Processing parameters
SI                 65536
SF           500.1700144 MHz
WDW                   EM
SSB                    0
LB                  0.30 Hz
GB                     0
PC                  1.00
```

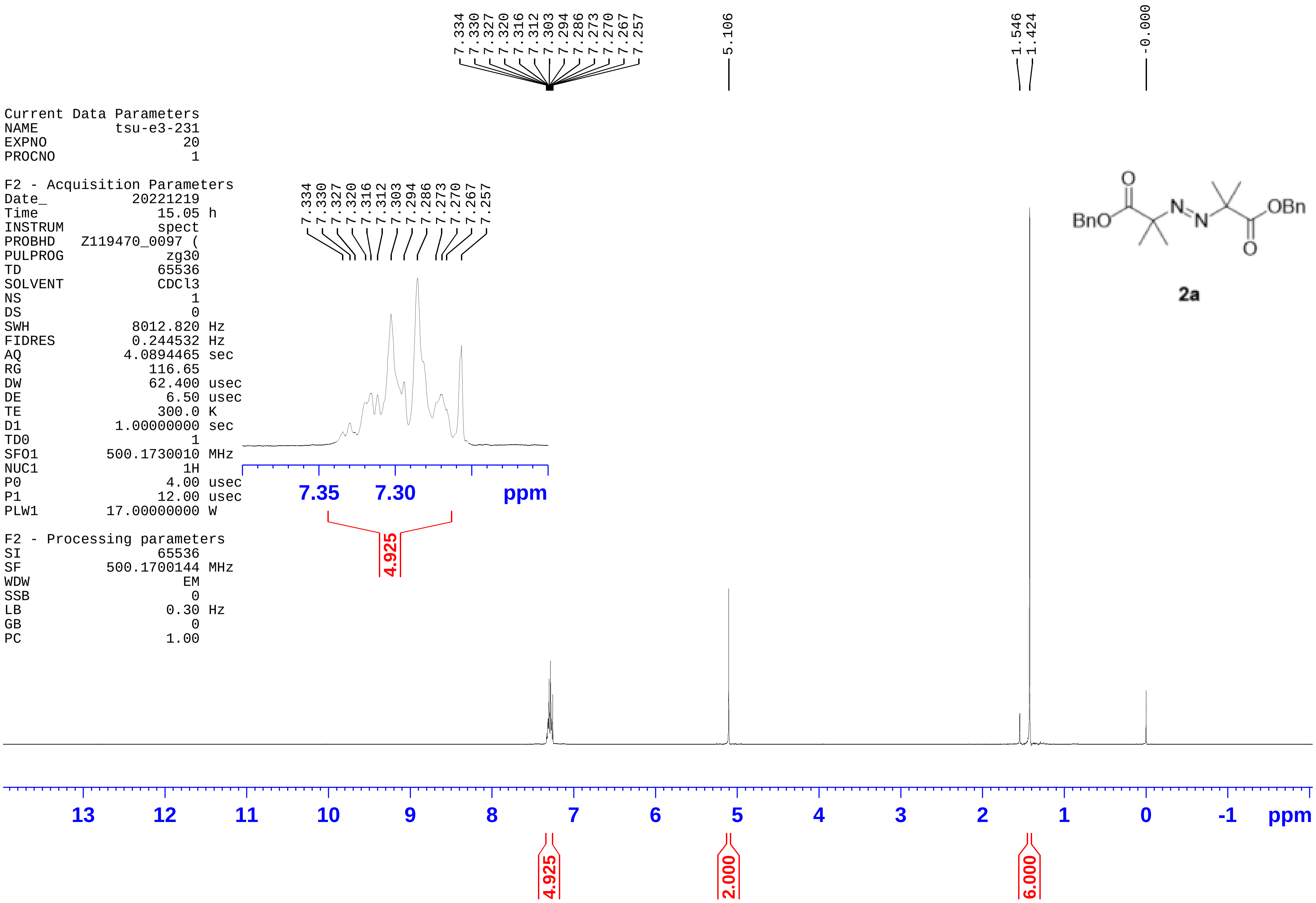

2a

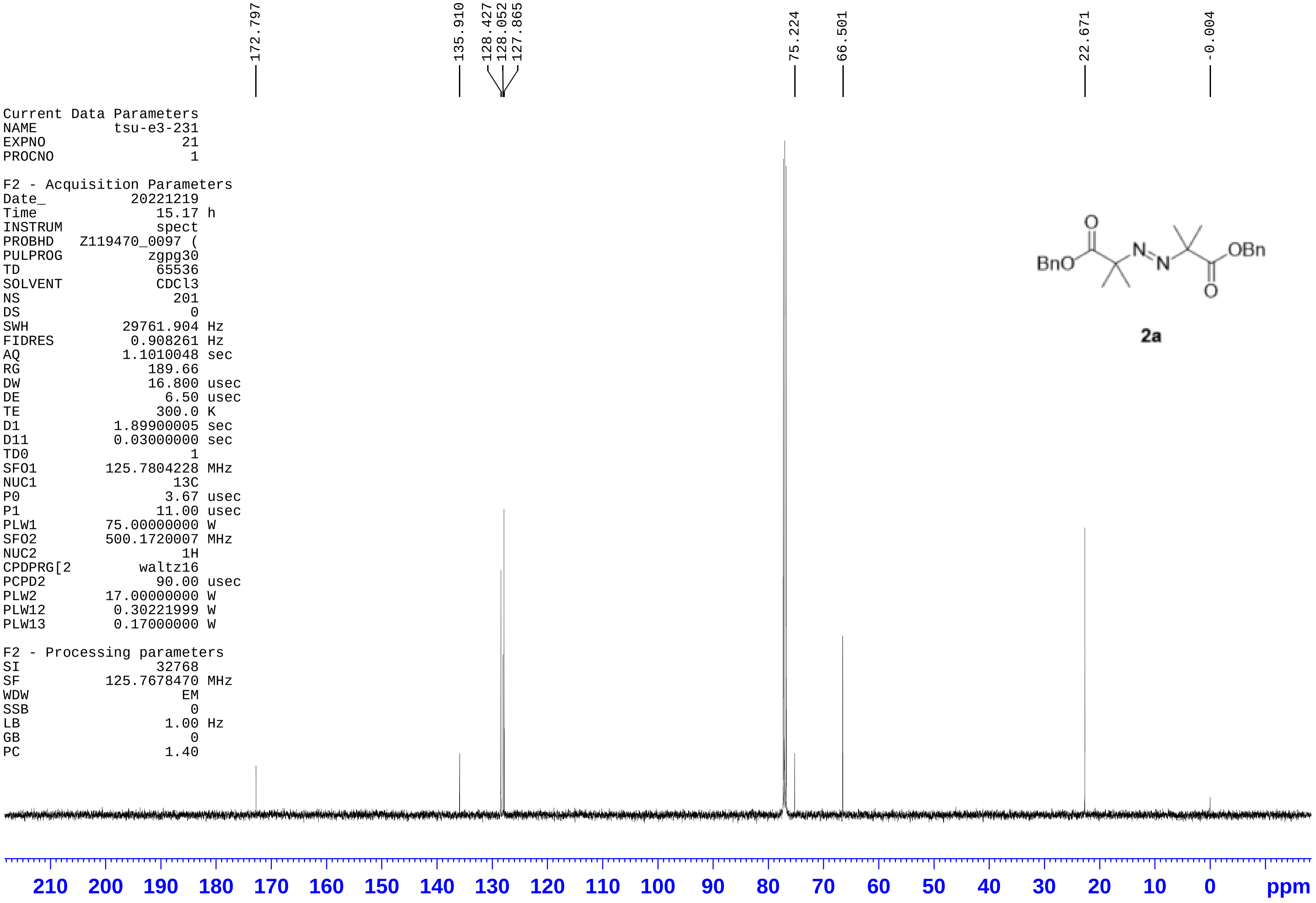


```
Current Data Parameters
NAME          tsu-e3-231
EXPNO                 21
PROCNO                 1

F2 - Acquisition Parameters
Date_           20221219
Time               15.17 h
INSTRUM            spect
PROBHD   Z119470_0097 (
PULPROG          zgpg30
TD                 65536
SOLVENT            CDCl3
NS                   201
DS                     0
SWH            29761.904 Hz
FIDRES          0.908261 Hz
AQ             1.1010048 sec
RG                189.66
DW                16.800 usec
DE                  6.50 usec
TE                 300.0 K
D1            1.89900005 sec
D11           0.03000000 sec
TD0                    1
SFO1         125.7804228 MHz
NUC1                 13C
P0                  3.67 usec
P1                 11.00 usec
PLW1         75.00000000 W
SFO2         500.1720007 MHz
NUC2                  1H
CPDPRG[2         waltz16
PCPD2              90.00 usec
PLW2         17.00000000 W
PLW12         0.30221999 W
PLW13         0.17000000 W

F2 - Processing parameters
SI                 32768
SF           125.7678470 MHz
WDW                   EM
SSB                    0
LB                  1.00 Hz
GB                     0
PC                  1.40
```

Current Data Parameters
NAME tsu-e3-240
EXPNO 10
PROCNO 1

F2 - Acquisition Parameters
Date_ 20221221
Time 14.35 h
INSTRUM spect
PROBHD Z119470_0097 (
PULPROG zg30
TD 65536
SOLVENT CDCl3
NS 1
DS 0
SWH 8012.820 Hz
FIDRES 0.244532 Hz
AQ 4.0894465 sec
RG 116.65
DW 62.400 usec
DE 6.50 usec
TE 300.0 K
D1 1.00000000 sec
TD0 1
SFO1 500.1730010 MHz
NUC1 1H
P0 4.00 usec
P1 12.00 usec
PLW1 17.00000000 W

F2 - Processing parameters
SI 65536
SF 500.1700116 MHz
WDW EM
SSB 0
LB 0.30 Hz
GB 0
PC 1.00

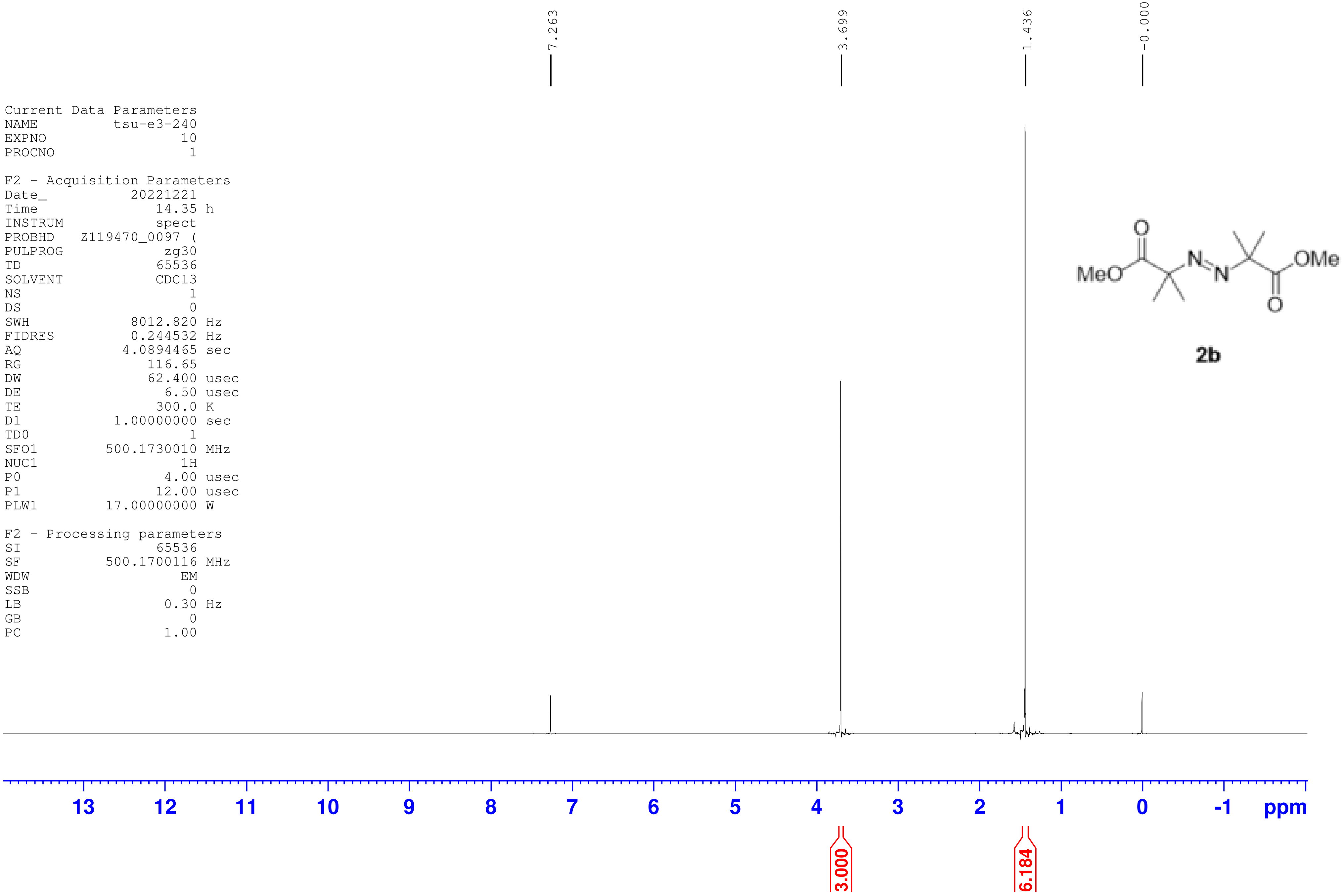

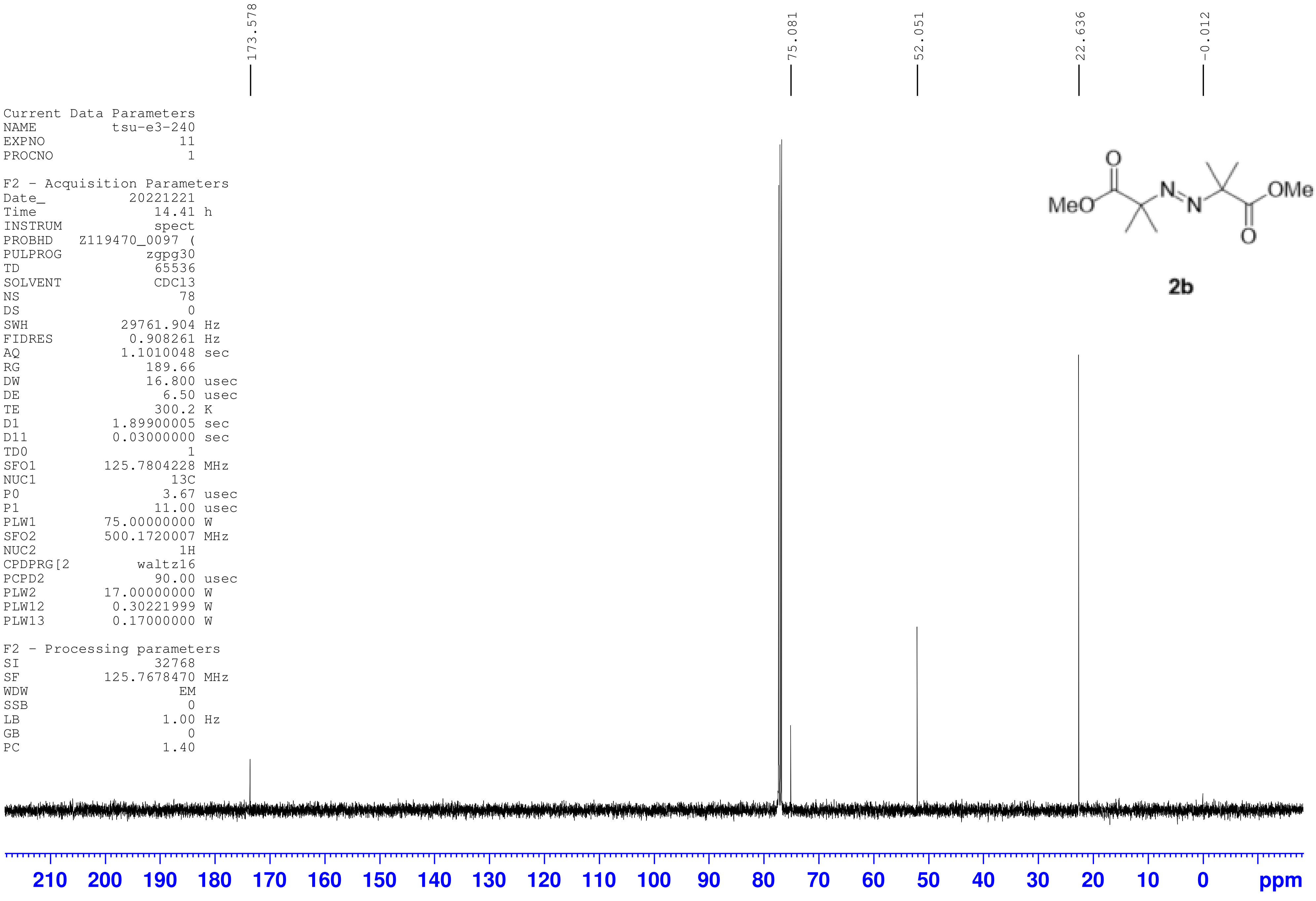
173.578
75.081
52.051
22.636
-0.012
Current Data Parameters
NAME tsu-e3-240
EXPNO 11
PROCNO 1
F2 - Acquisition Parameters
Date_ 20221221
Time 14.41 h
INSTRUM spect
PROBHD Z119470_0097 (
PULPROG zgpg30
TD 65536
SOLVENT CDCl3
NS 78
DS 0
SWH 29761.904 Hz
FIDRES 0.908261 Hz
AQ 1.1010048 sec
RG 189.66
DW 16.800 usec
DE 6.50 usec
TE 300.2 K
D1 1.89900005 sec
D11 0.03000000 sec
TD0 1
SFO1 125.7804228 MHz
NUC1 13C
P0 3.67 usec
P1 11.00 usec
PLW1 75.00000000 W
SFO2 500.1720007 MHz
NUC2 1H
CPDPRG[2 waltz16
PCPD2 90.00 usec
PLW2 17.00000000 W
PLW12 0.30221999 W
PLW13 0.17000000 W
F2 - Processing parameters
SI 32768
SF 125.7678470 MHz
WDW EM
SSB 0
LB 1.00 Hz
GB 0
PC 1.40
210 200 190 180 170 160 150 140 130 120 110 100 90 80 70 60 50 40 30 20 10 0 ppm
MeO
O
N
N
OMe
O

2b

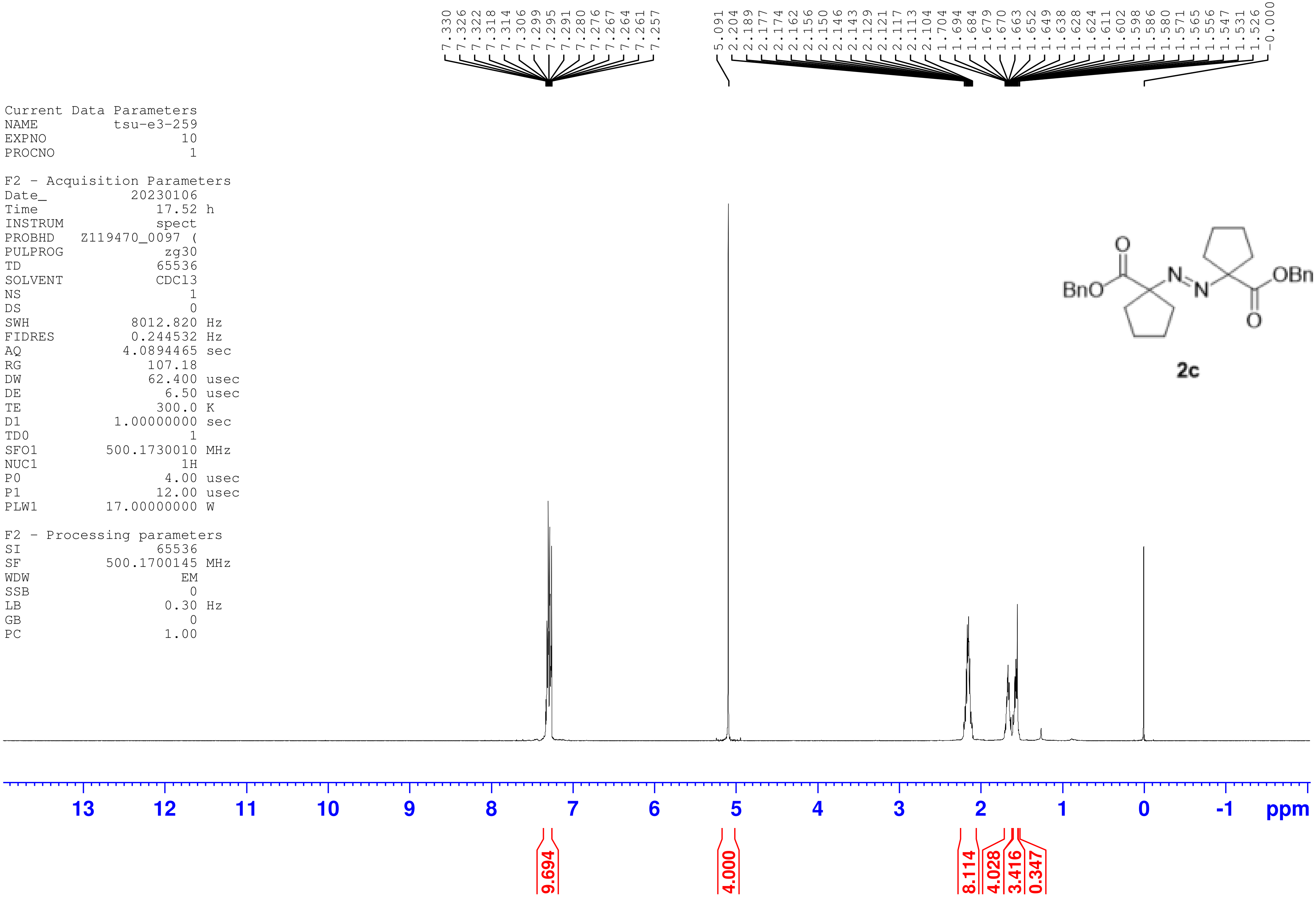


```
Current Data Parameters
NAME          tsu-e3-259
EXPNO                 10
PROCNO                 1

F2 - Acquisition Parameters
Date_           20230106
Time               17.52 h
INSTRUM            spect
PROBHD   Z119470_0097 (
PULPROG             zg30
TD                 65536
SOLVENT            CDCl3
NS                     1
DS                     0
SWH             8012.820 Hz
FIDRES          0.244532 Hz
AQ             4.0894465 sec
RG                107.18
DW                62.400 usec
DE                  6.50 usec
TE                 300.0 K
D1            1.00000000 sec
TD0                    1
SFO1         500.1730010 MHz
NUC1                  1H
P0                  4.00 usec
P1                 12.00 usec
PLW1         17.00000000 W

F2 - Processing parameters
SI                 65536
SF           500.1700145 MHz
WDW                   EM
SSB                    0
LB                  0.30 Hz
GB                     0
PC                  1.00
```

2c

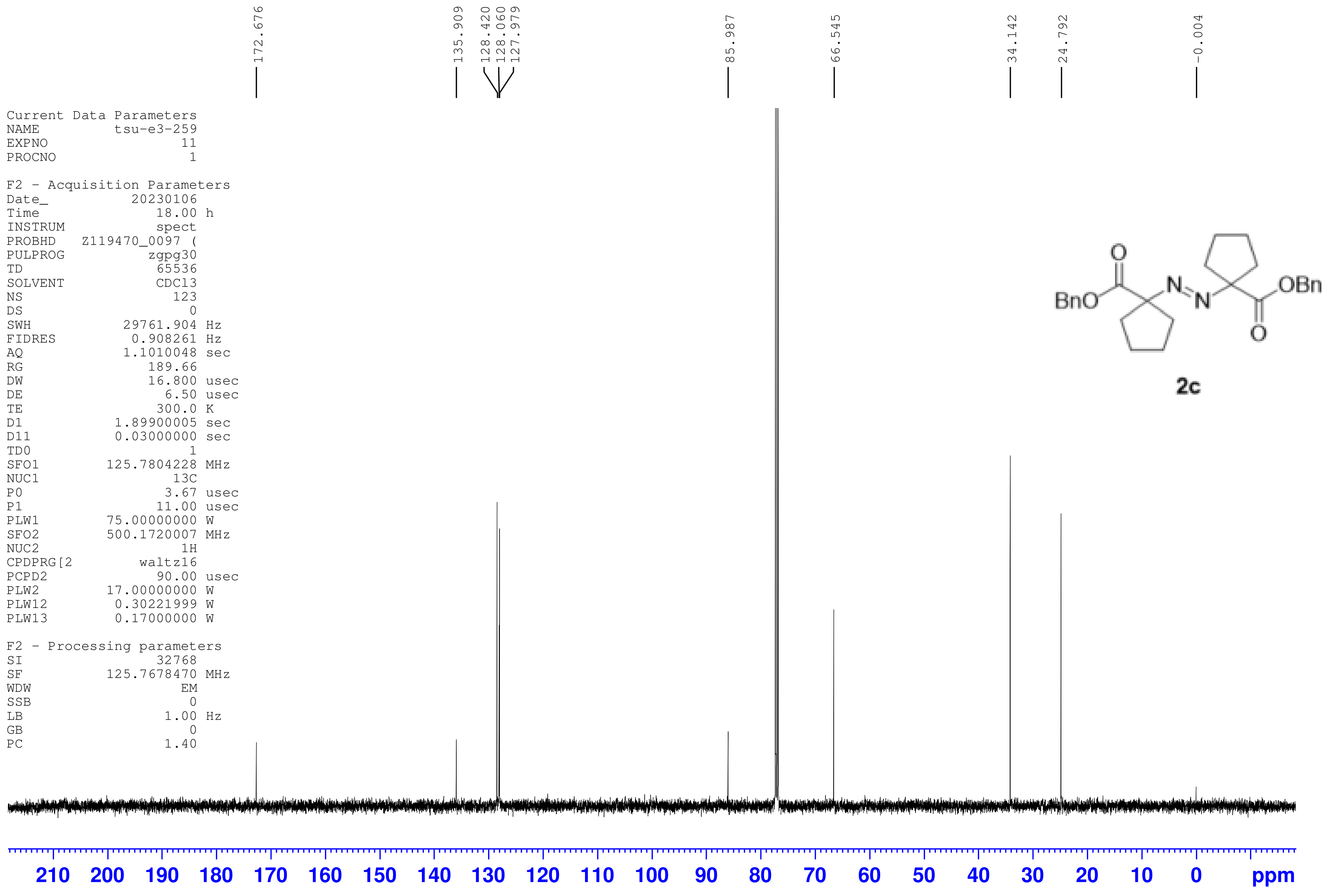


```
Current Data Parameters
NAME          tsu-e3-259
EXPNO                 11
PROCNO                 1

F2 - Acquisition Parameters
Date_           20230106
Time               18.00 h
INSTRUM            spect
PROBHD   Z119470_0097 (
PULPROG          zgpg30
TD                 65536
SOLVENT            CDCl3
NS                   123
DS                     0
SWH            29761.904 Hz
FIDRES          0.908261 Hz
AQ             1.1010048 sec
RG                189.66
DW                16.800 usec
DE                  6.50 usec
TE                 300.0 K
D1            1.89900005 sec
D11           0.03000000 sec
TD0                    1
SFO1         125.7804228 MHz
NUC1                 13C
P0                  3.67 usec
P1                 11.00 usec
PLW1         75.00000000 W
SFO2         500.1720007 MHz
NUC2                  1H
CPDPRG[2         waltz16
PCPD2              90.00 usec
PLW2         17.00000000 W
PLW12         0.30221999 W
PLW13         0.17000000 W

F2 - Processing parameters
SI                 32768
SF           125.7678470 MHz
WDW                   EM
SSB                    0
LB                  1.00 Hz
GB                     0
PC                  1.40
```


2c

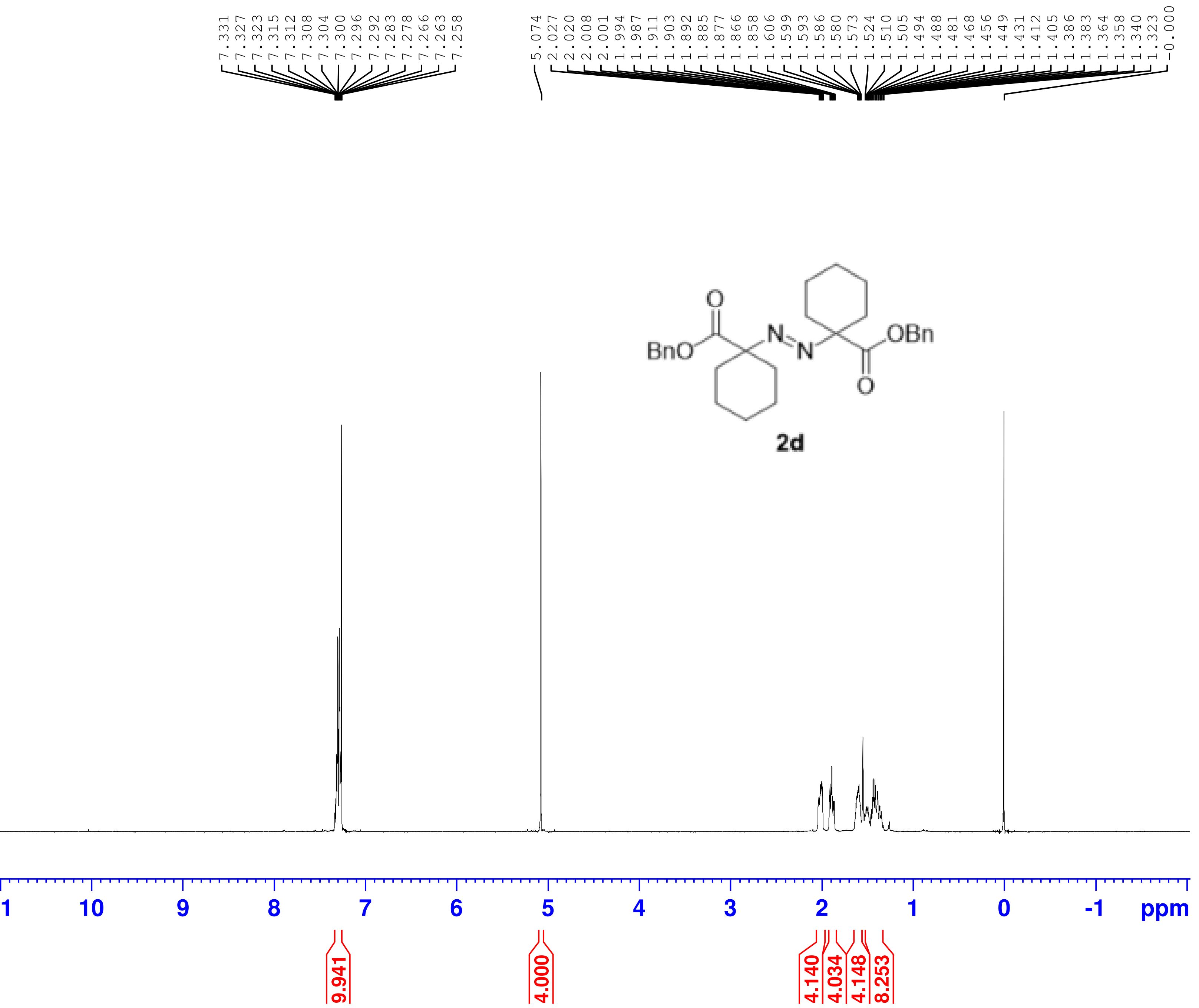


```
Current Data Parameters
NAME           tsu-e3-266
EXPNO                  10
PROCNO                  1

F2 - Acquisition Parameters
Date_            20230110
Time                18.06 h
INSTRUM             spect
PROBHD    Z119470_0097 (
PULPROG              zg30
TD                  65536
SOLVENT             CDCl3
NS                      1
DS                      0
SWH              8012.820 Hz
FIDRES           0.244532 Hz
AQ              4.0894465 sec
RG                 130.52
DW                 62.400 usec
DE                   6.50 usec
TE                  300.0 K
D1             1.00000000 sec
TD0                     1
SFO1          500.1730010 MHz
NUC1                   1H
P0                   4.00 usec
P1                  12.00 usec
PLW1          17.00000000 W

F2 - Processing parameters
SI                  65536
SF            500.1700138 MHz
WDW                    EM
SSB                     0
LB                   0.30 Hz
GB                      0
PC                   1.00
```

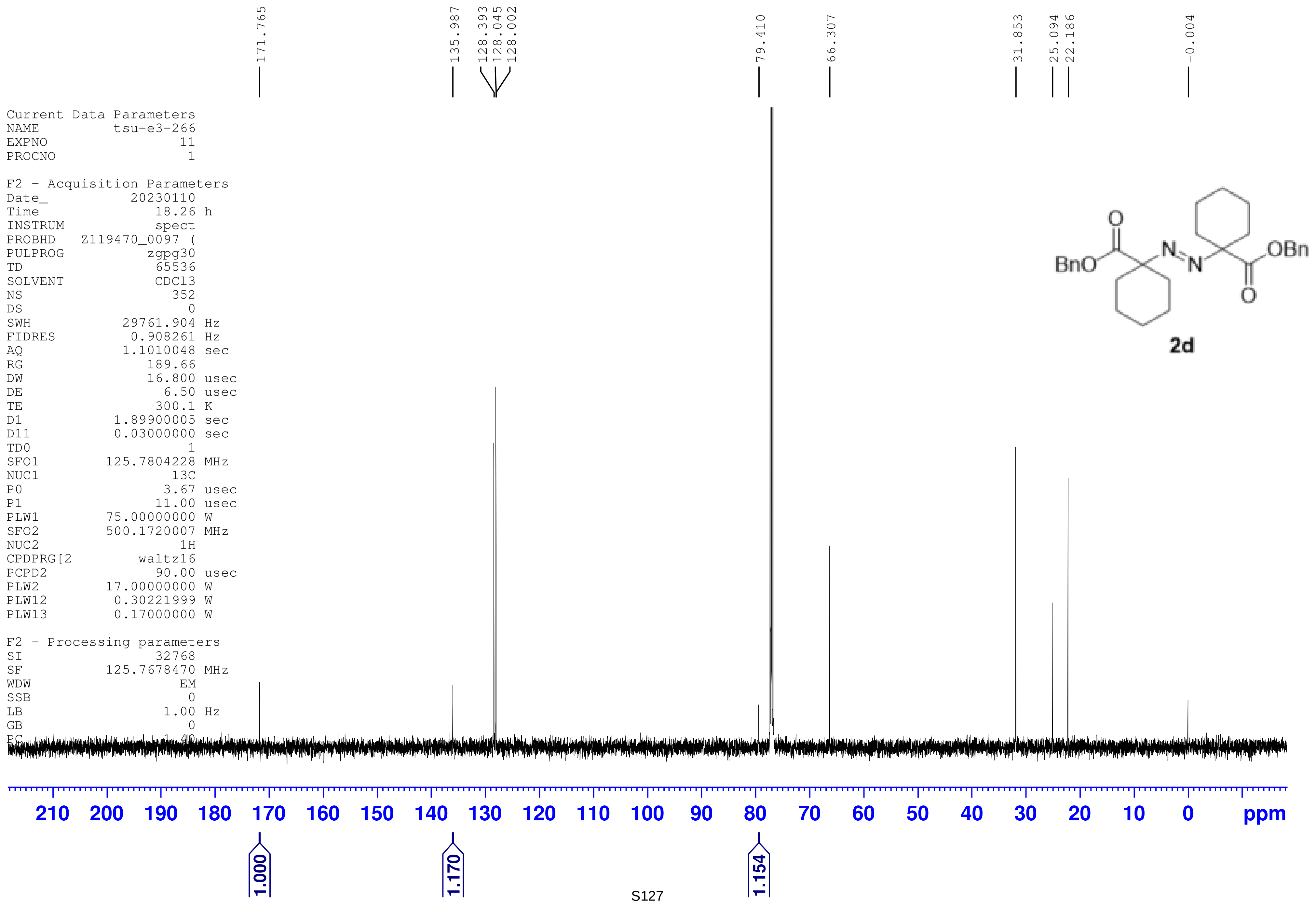
171.765
135.987
128.393
128.045
128.002
79.410
66.307
31.853
25.094
22.186
-0.004
Current Data Parameters
NAME tsu-e3-266
EXPNO 11
PROCNO 1
F2 - Acquisition Parameters
Date_ 20230110
Time 18.26 h
INSTRUM spect
PROBHD Z119470_0097 (
PULPROG zgpg30
TD 65536
SOLVENT CDCl3
NS 352
DS 0
SWH 29761.904 Hz
FIDRES 0.908261 Hz
AQ 1.1010048 sec
RG 189.66
DW 16.800 usec
DE 6.50 usec
TE 300.1 K
D1 1.89900005 sec
D11 0.03000000 sec
TD0 1
SFO1 125.7804228 MHz
NUC1 13C
P0 3.67 usec
P1 11.00 usec
PLW1 75.00000000 W
SFO2 500.1720007 MHz
NUC2 1H
CPDPRG[2 waltz16
PCPD2 90.00 usec
PLW2 17.00000000 W
PLW12 0.30221999 W
PLW13 0.17000000 W
F2 - Processing parameters
SI 32768
SF 125.7678470 MHz
WDW EM
SSB 0
LB 1.00 Hz
GB 0
BnO
O
N
N
OBn
O
2d
210 200 190 180 170 160 150 140 130 120 110 100 90 80 70 60 50 40 30 20 10 0 ppm
1.000
1.170
1.154

```
Current Data Parameters
NAME          tsu-e3-273
EXPNO                 10
PROCNO                 1

F2 - Acquisition Parameters
Date_           20230118
Time               18.19 h
INSTRUM            spect
PROBHD   Z119470_0097 (
PULPROG             zg30
TD                 65536
SOLVENT            CDCl3
NS                     1
DS                     0
SWH             8012.820 Hz
FIDRES          0.244532 Hz
AQ             4.0894465 sec
RG                150.08
DW                62.400 usec
DE                  6.50 usec
TE                 300.0 K
D1            1.00000000 sec
TD0                    1
SFO1         500.1730010 MHz
NUC1                  1H
P0                  4.00 usec
P1                 12.00 usec
PLW1         17.00000000 W

F2 - Processing parameters
SI                 65536
SF           500.1700128 MHz
WDW                   EM
SSB                    0
LB                  0.30 Hz
GB                     0
PC                  1.00
```

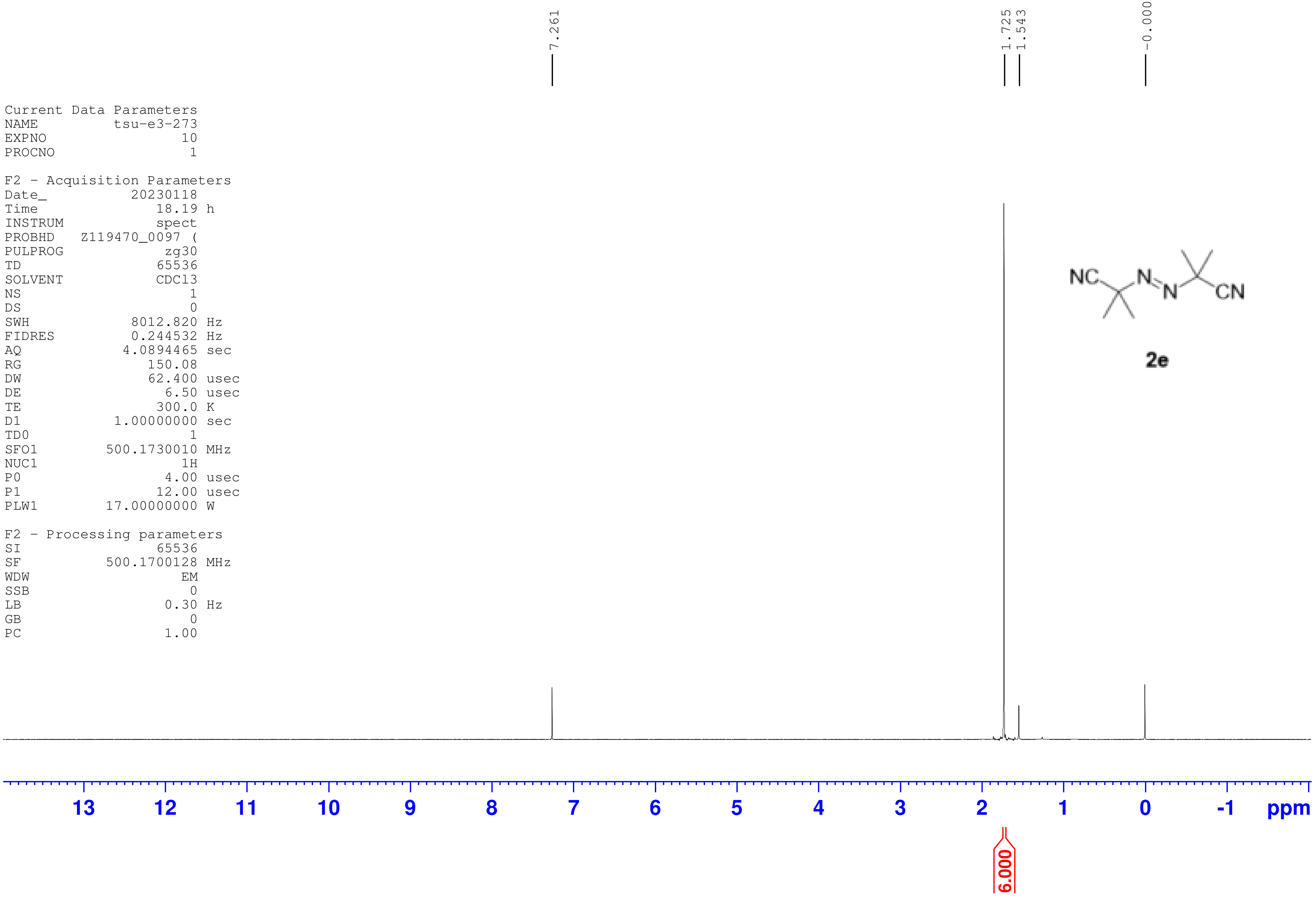

2e

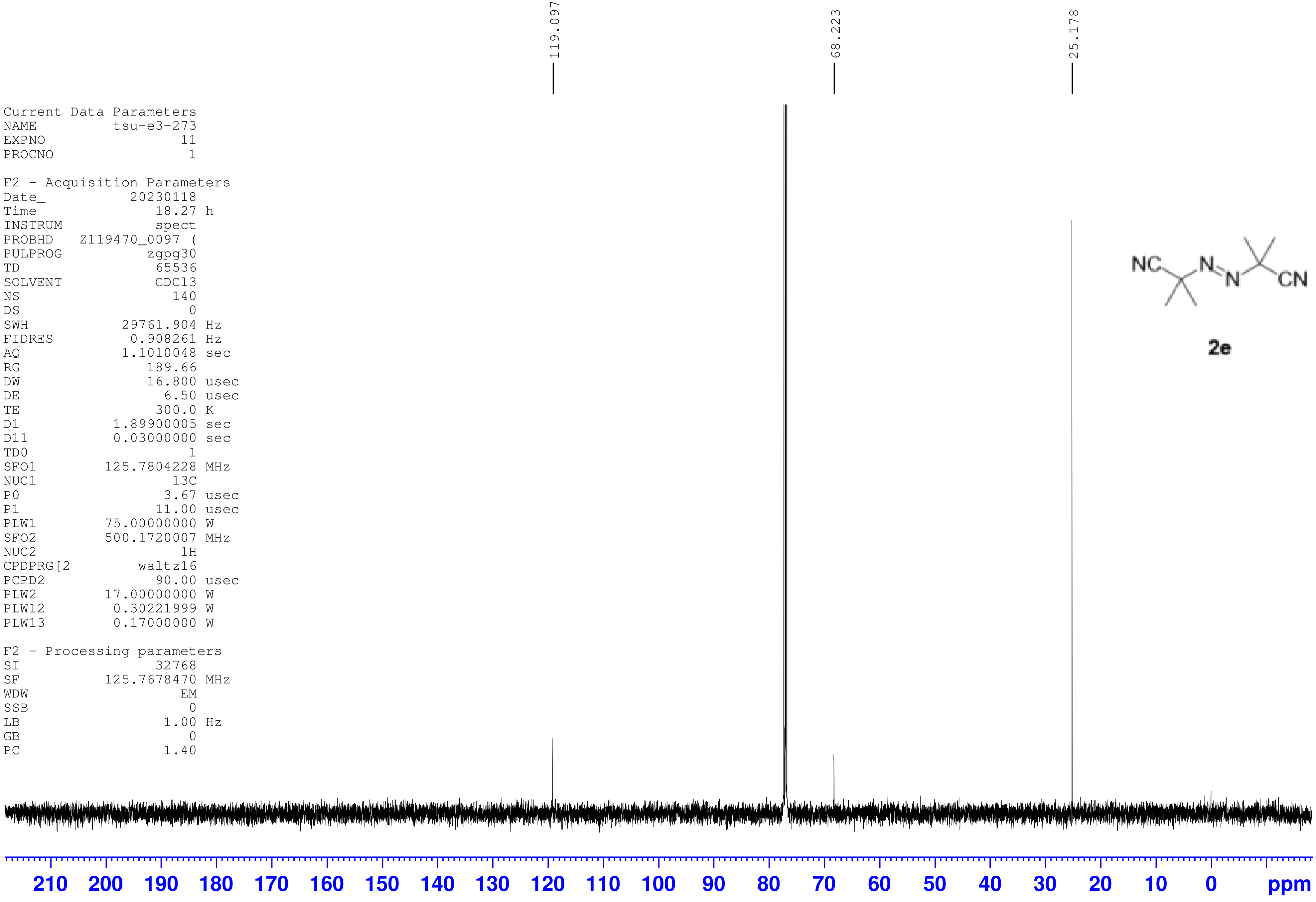


```
Current Data Parameters
NAME          tsu-e3-273
EXPNO                 11
PROCNO                 1

F2 - Acquisition Parameters
Date_           20230118
Time               18.27 h
INSTRUM            spect
PROBHD   Z119470_0097 (
PULPROG          zgpg30
TD                 65536
SOLVENT            CDCl3
NS                   140
DS                     0
SWH            29761.904 Hz
FIDRES          0.908261 Hz
AQ             1.1010048 sec
RG                189.66
DW                16.800 usec
DE                  6.50 usec
TE                 300.0 K
D1            1.89900005 sec
D11           0.03000000 sec
TD0                    1
SFO1         125.7804228 MHz
NUC1                 13C
P0                  3.67 usec
P1                 11.00 usec
PLW1         75.00000000 W
SFO2         500.1720007 MHz
NUC2                  1H
CPDPRG[2         waltz16
PCPD2              90.00 usec
PLW2         17.00000000 W
PLW12         0.30221999 W
PLW13         0.17000000 W

F2 - Processing parameters
SI                 32768
SF           125.7678470 MHz
WDW                   EM
SSB                    0
LB                  1.00 Hz
GB                     0
PC                  1.40
```


2e

Current Data Parameters
NAME tsu-e3-376
EXPNO 10
PROCNO 1

F2 - Acquisition Parameters
Date_ 20230502
Time 14.35 h
INSTRUM spect
PROBHD Z119470_0344 (
PULPROG zg30
TD 65536
SOLVENT CDCl3
NS 1
DS 0
SWH 8012.820 Hz
FIDRES 0.244532 Hz
AQ 4.0894465 sec
RG 107.18
DW 62.400 usec
DE 6.50 usec
TE 298.0 K
D1 1.00000000 sec
TD0 1
SFO1 500.1730010 MHz
NUC1 1H
P0 4.83 usec
P1 14.50 usec
PLW1 10.80000019 W

F2 - Processing parameters
SI 65536
SF 500.1700117 MHz
WDW EM
SSB 0
LB 0.30 Hz
GB 0
PC 1.00

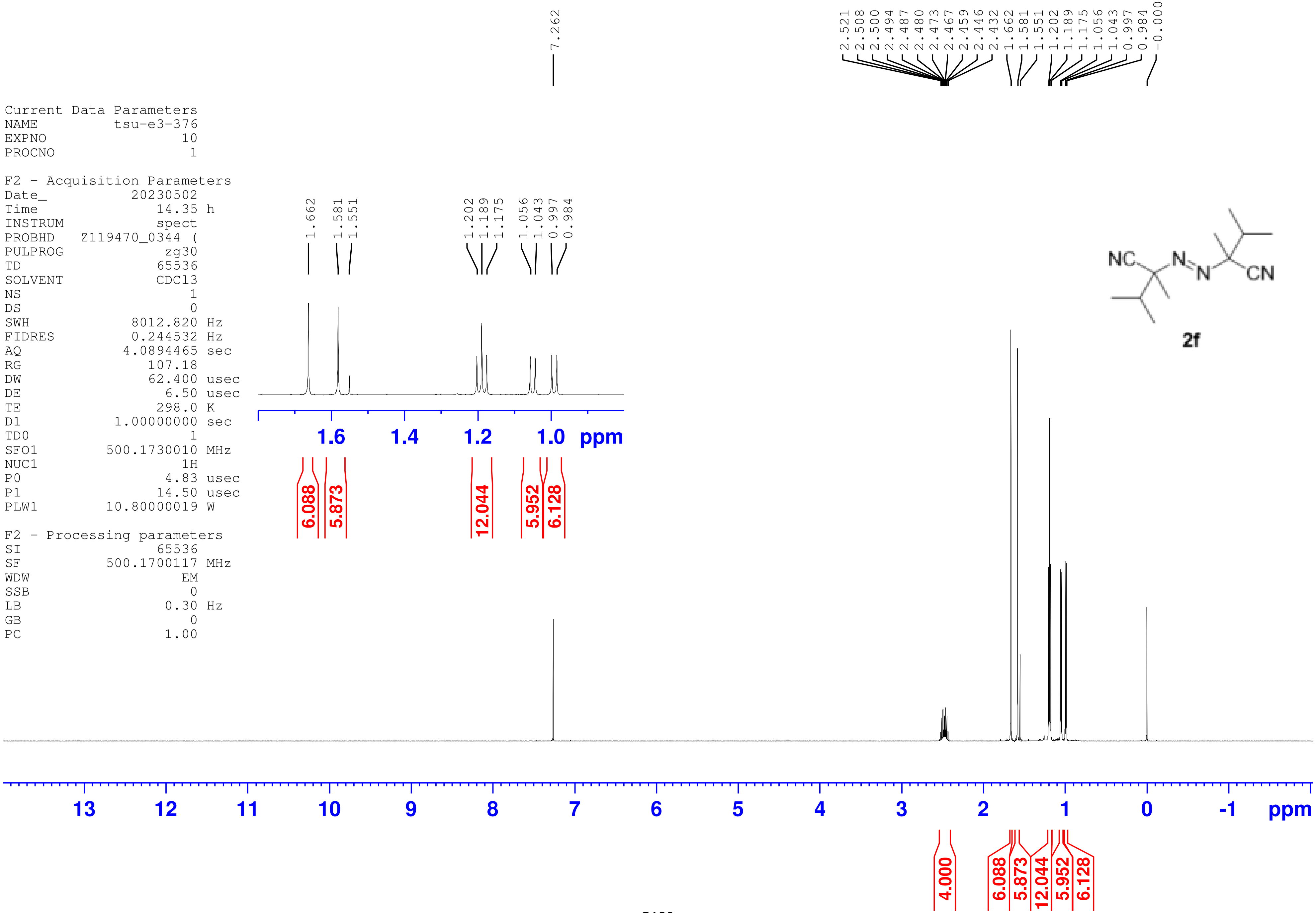

2f

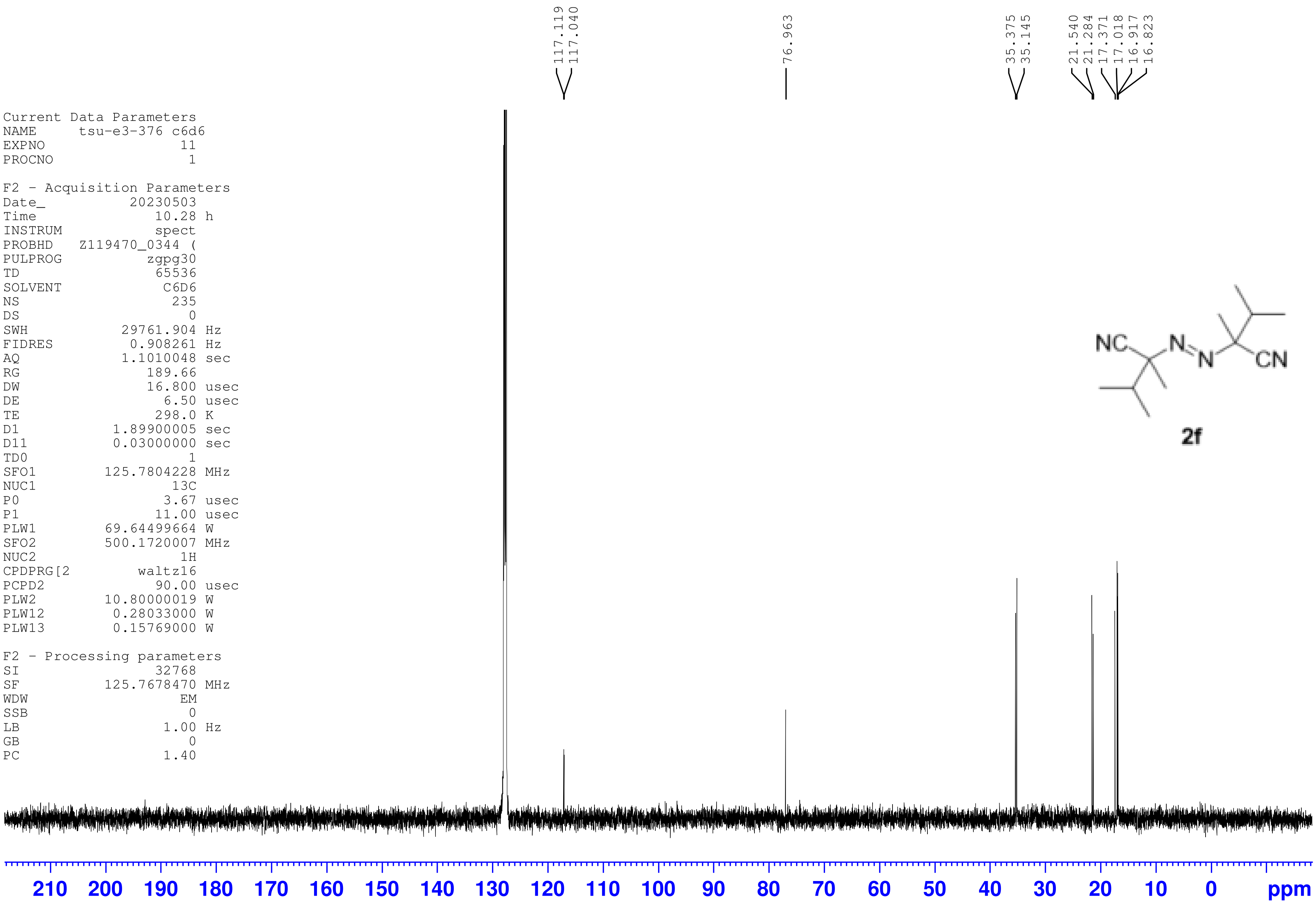


```
Current Data Parameters
NAME      tsu-e3-376 c6d6
EXPNO                  11
PROCNO                  1

F2 - Acquisition Parameters
Date_            20230503
Time                10.28 h
INSTRUM             spect
PROBHD   Z119470_0344 (
PULPROG           zgpg30
TD                 65536
SOLVENT             C6D6
NS                   235
DS                     0
SWH            29761.904 Hz
FIDRES          0.908261 Hz
AQ             1.1010048 sec
RG                189.66
DW                16.800 usec
DE                  6.50 usec
TE                 298.0 K
D1            1.89900005 sec
D11           0.03000000 sec
TD0                    1
SFO1         125.7804228 MHz
NUC1                 13C
P0                  3.67 usec
P1                 11.00 usec
PLW1         69.64499664 W
SFO2         500.1720007 MHz
NUC2                  1H
CPDPRG[2         waltz16
PCPD2              90.00 usec
PLW2         10.80000019 W
PLW12         0.28033000 W
PLW13         0.15769000 W

F2 - Processing parameters
SI                 32768
SF           125.7678470 MHz
WDW                   EM
SSB                    0
LB                  1.00 Hz
GB                     0
PC                  1.40
```


2f

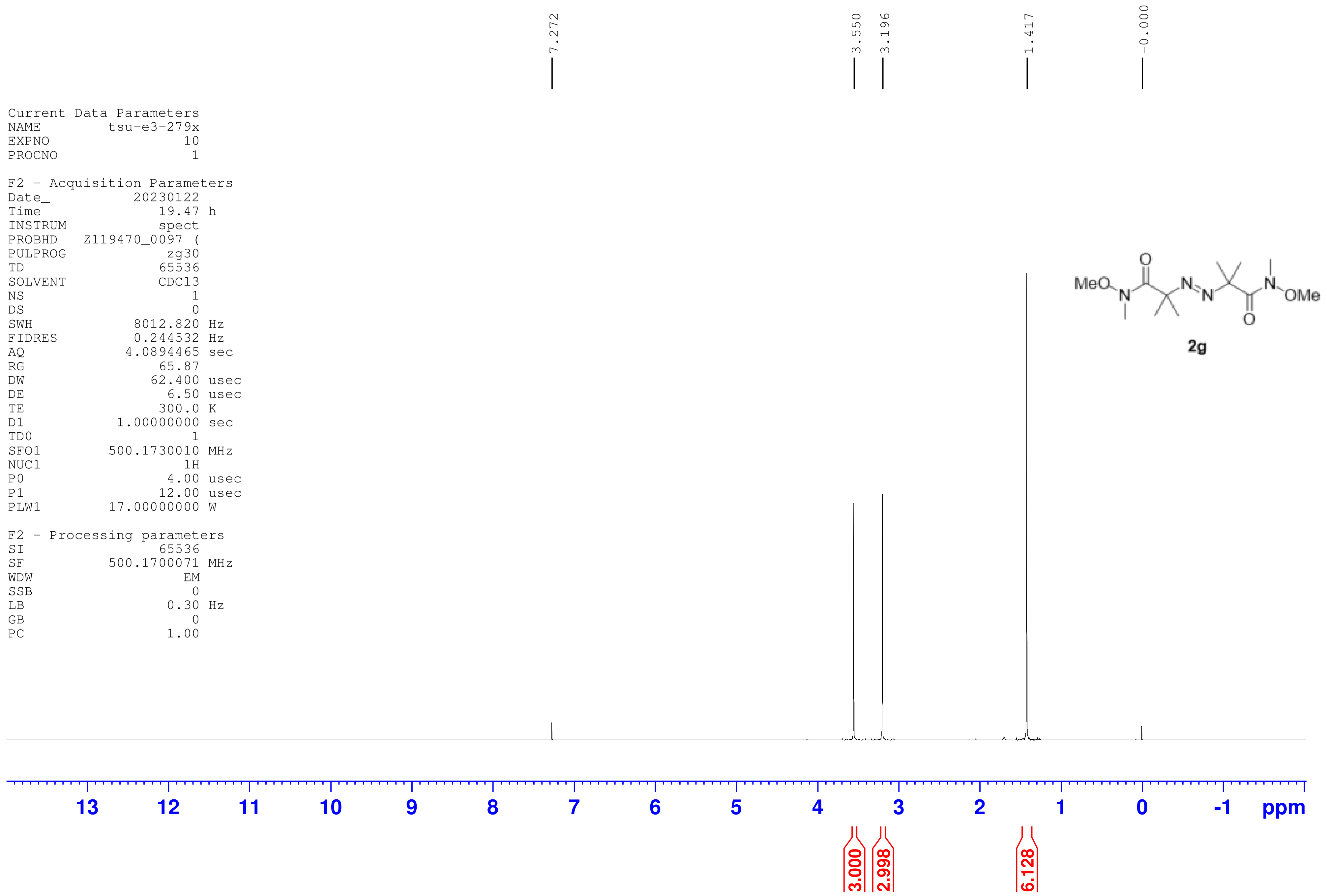
Current Data Parameters
NAME tsu-e3-279x
EXPNO 10
PROCNO 1
F2 - Acquisition Parameters
Date_ 20230122
Time 19.47 h
INSTRUM spect
PROBHD Z119470_0097 (
PULPROG zg30
TD 65536
SOLVENT CDCl3
NS 1
DS 0
SWH 8012.820 Hz
FIDRES 0.244532 Hz
AQ 4.0894465 sec
RG 65.87
DW 62.400 usec
DE 6.50 usec
TE 300.0 K
D1 1.00000000 sec
TD0 1
SFO1 500.1730010 MHz
NUC1 1H
P0 4.00 usec
P1 12.00 usec
PLW1 17.00000000 W
F2 - Processing parameters
SI 65536
SF 500.1700071 MHz
WDW EM
SSB 0
LB 0.30 Hz
GB 0
PC 1.00
7.272
3.550
3.196
1.417
-0.000
MeO
N
O
N
N
N
OMe
O
2g
13
12
11
10
9
8
7
6
5
4
3
2
1
0
-1
ppm
3.000
2.998
6.128

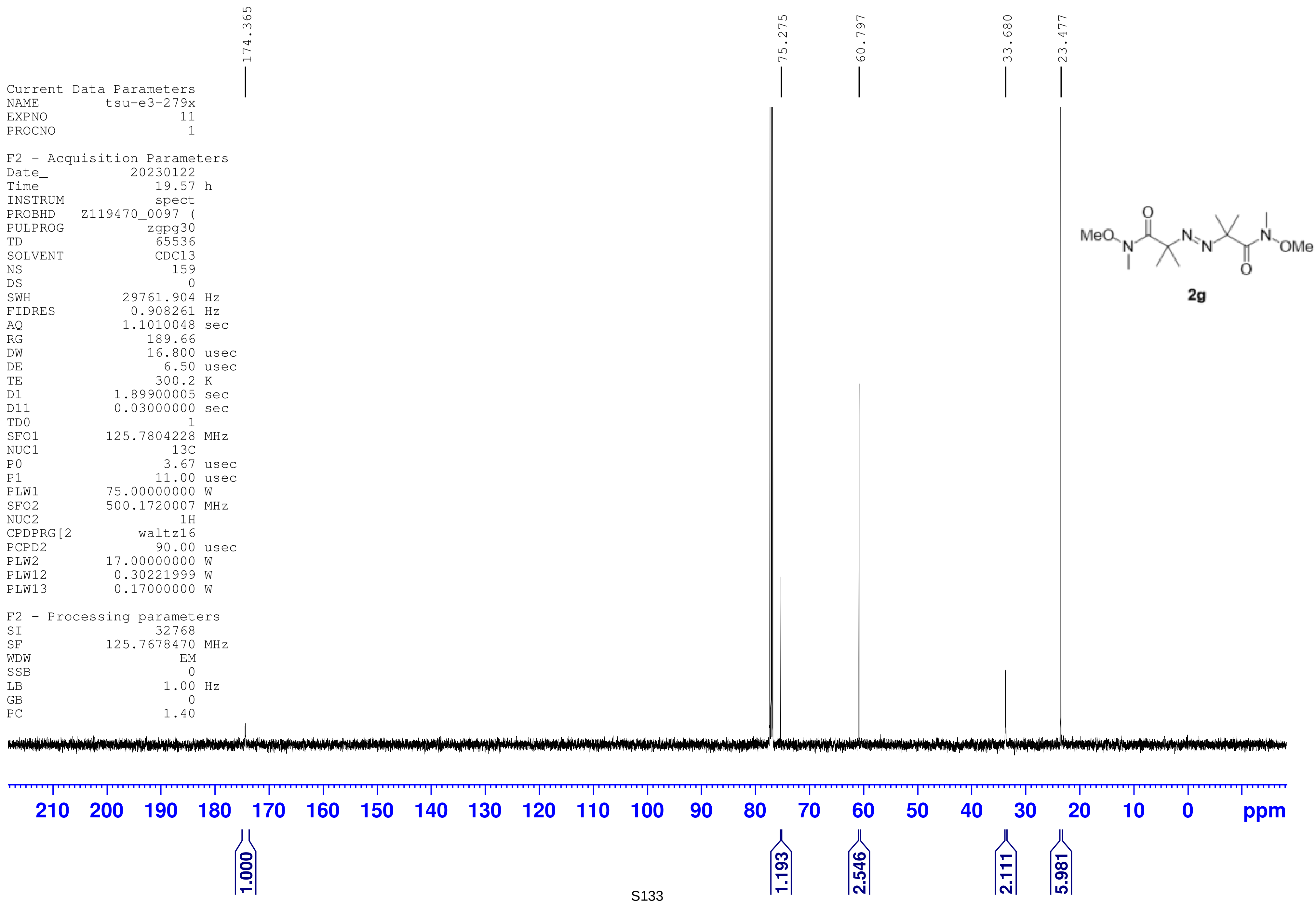
Current Data Parameters
NAME tsu-e3-279x
EXPNO 11
PROCNO 1
F2 - Acquisition Parameters
Date_ 20230122
Time 19.57 h
INSTRUM spect
PROBHD Z119470_0097 (
PULPROG zgpg30
TD 65536
SOLVENT CDCl3
NS 159
DS 0
SWH 29761.904 Hz
FIDRES 0.908261 Hz
AQ 1.1010048 sec
RG 189.66
DW 16.800 usec
DE 6.50 usec
TE 300.2 K
D1 1.89900005 sec
D11 0.03000000 sec
TD0 1
SFO1 125.7804228 MHz
NUC1 13C
P0 3.67 usec
P1 11.00 usec
PLW1 75.00000000 W
SFO2 500.1720007 MHz
NUC2 1H
CPDPRG[2 waltz16
PCPD2 90.00 usec
PLW2 17.00000000 W
PLW12 0.30221999 W
PLW13 0.17000000 W
F2 - Processing parameters
SI 32768
SF 125.7678470 MHz
WDW EM
SSB 0
LB 1.00 Hz
GB 0
PC 1.40
174.365
75.275
60.797
33.680
23.477
210 200 190 180 170 160 150 140 130 120 110 100 90 80 70 60 50 40 30 20 10 0 ppm
1.000
1.193
2.546
2.111
5.981
MeO
N
O
N
N
N
OMe
O

2g

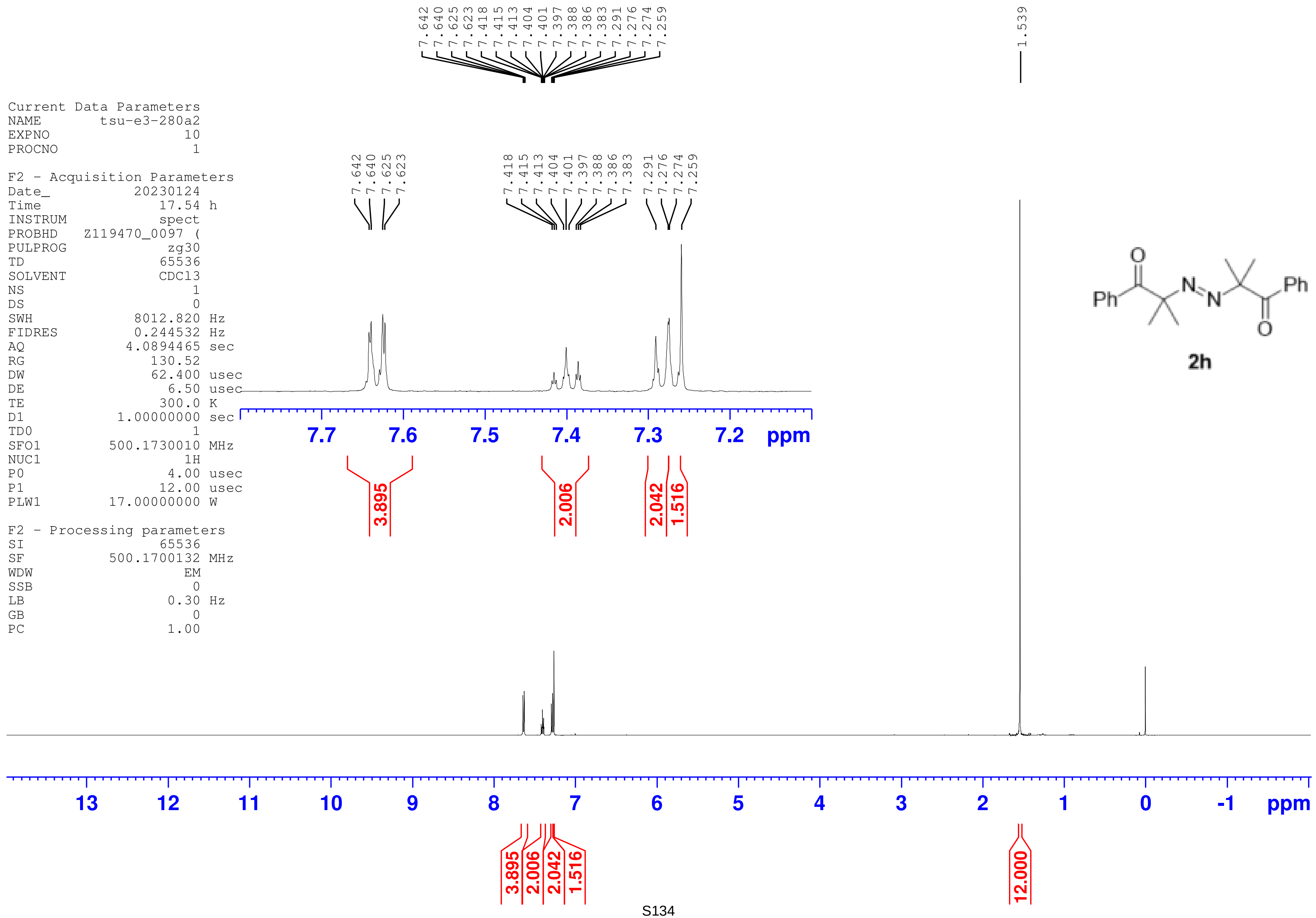
7.642
7.640
7.625
7.623
7.418
7.415
7.413
7.404
7.401
7.397
7.388
7.386
7.383
7.291
7.276
7.274
7.259
1.539
Current Data Parameters
NAME tsu-e3-280a2
EXPNO 10
PROCNO 1
F2 - Acquisition Parameters
Date_ 20230124
Time 17.54 h
INSTRUM spect
PROBHD Z119470_0097 (
PULPROG zg30
TD 65536
SOLVENT CDCl3
NS 1
DS 0
SWH 8012.820 Hz
FIDRES 0.244532 Hz
AQ 4.0894465 sec
RG 130.52
DW 62.400 usec
DE 6.50 usec
TE 300.0 K
D1 1.00000000 sec
TD0 1
SFO1 500.1730010 MHz
NUC1 1H
P0 4.00 usec
P1 12.00 usec
PLW1 17.00000000 W
F2 - Processing parameters
SI 65536
SF 500.1700132 MHz
WDW EM
SSB 0
LB 0.30 Hz
GB 0
PC 1.00
7.7
7.6
7.5
7.4
7.3
7.2
ppm
3.895
2.006
2.042
1.516
13
12
11
10
9
8
7
6
5
4
3
2
1
0
-1
ppm
3.895
2.006
2.042
1.516
12.000
Ph
O
N
N
Ph
O

2h

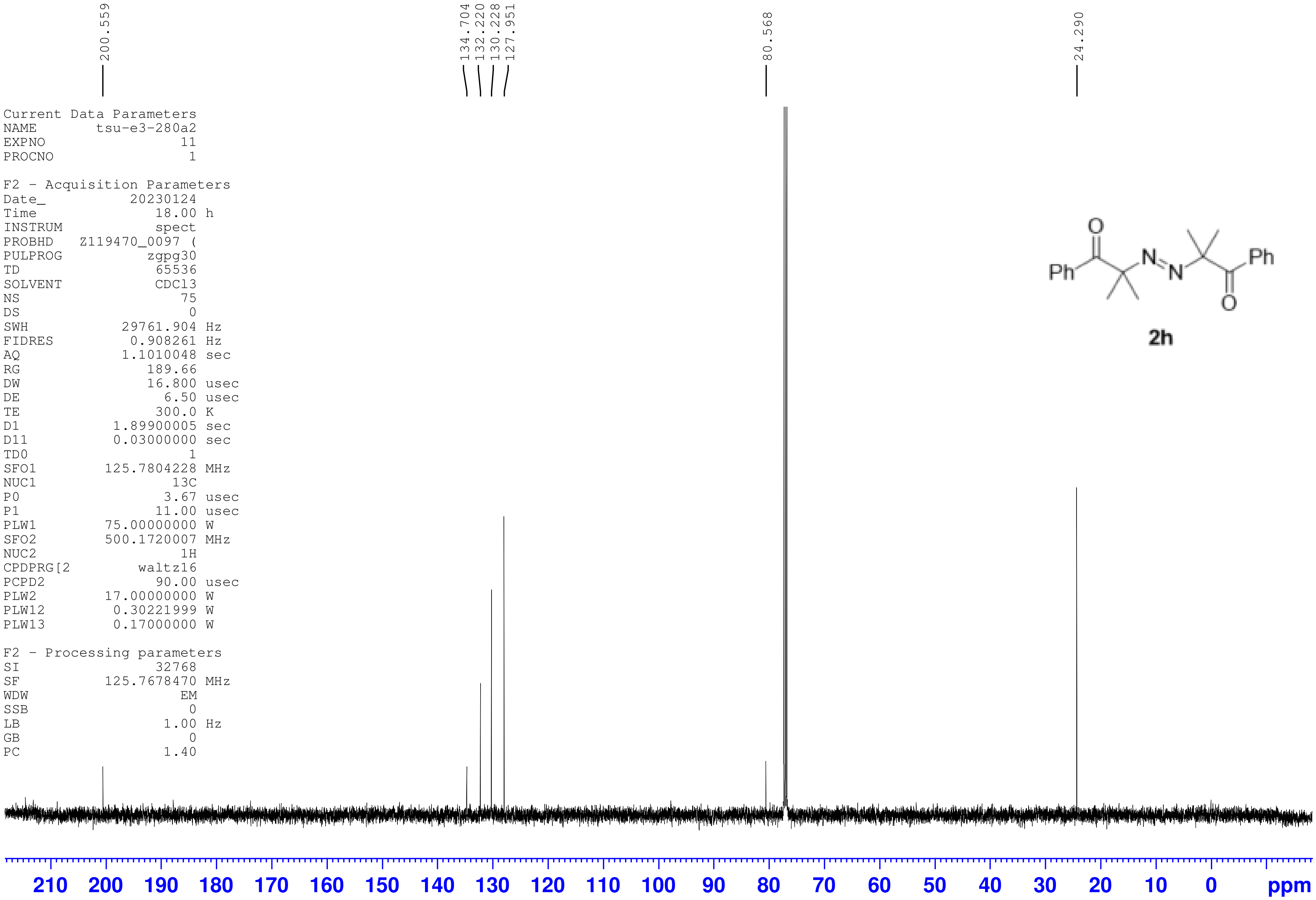
200.559
134.704
132.220
130.228
127.951
80.568
24.290
Current Data Parameters
NAME tsu-e3-280a2
EXPNO 11
PROCNO 1
F2 - Acquisition Parameters
Date_ 20230124
Time 18.00 h
INSTRUM spect
PROBHD Z119470_0097 (
PULPROG zgpg30
TD 65536
SOLVENT CDCl3
NS 75
DS 0
SWH 29761.904 Hz
FIDRES 0.908261 Hz
AQ 1.1010048 sec
RG 189.66
DW 16.800 usec
DE 6.50 usec
TE 300.0 K
D1 1.89900005 sec
D11 0.03000000 sec
TD0 1
SFO1 125.7804228 MHz
NUC1 13C
P0 3.67 usec
P1 11.00 usec
PLW1 75.00000000 W
SFO2 500.1720007 MHz
NUC2 1H
CPDPRG[2 waltz16
PCPD2 90.00 usec
PLW2 17.00000000 W
PLW12 0.30221999 W
PLW13 0.17000000 W
F2 - Processing parameters
SI 32768
SF 125.7678470 MHz
WDW EM
SSB 0
LB 1.00 Hz
GB 0
PC 1.40
Ph
O
N
N
Ph
O
2h
210 200 190 180 170 160 150 140 130 120 110 100 90 80 70 60 50 40 30 20 10 0 ppm

```
Current Data Parameters
NAME          tsu-e3-301x
EXPNO                  10
PROCNO                  1

F2 - Acquisition Parameters
Date_            20230211
Time                17.02 h
INSTRUM             spect
PROBHD    Z130033_0007 (
PULPROG              zg30
TD                  65536
SOLVENT             CDCl3
NS                      1
DS                      0
SWH              8012.820 Hz
FIDRES           0.244532 Hz
AQ              4.0894465 sec
RG                  31.29
DW                 62.400 usec
DE                  10.00 usec
TE                  300.0 K
D1             1.00000000 sec
TD0                     1
SFO1          500.1730010 MHz
NUC1                   1H
P0                   4.00 usec
P1                  12.00 usec
PLW1          13.50000000 W

F2 - Processing parameters
SI                  65536
SF            500.1700115 MHz
WDW                    EM
SSB                     0
LB                   0.30 Hz
GB                      0
PC                   1.00
```

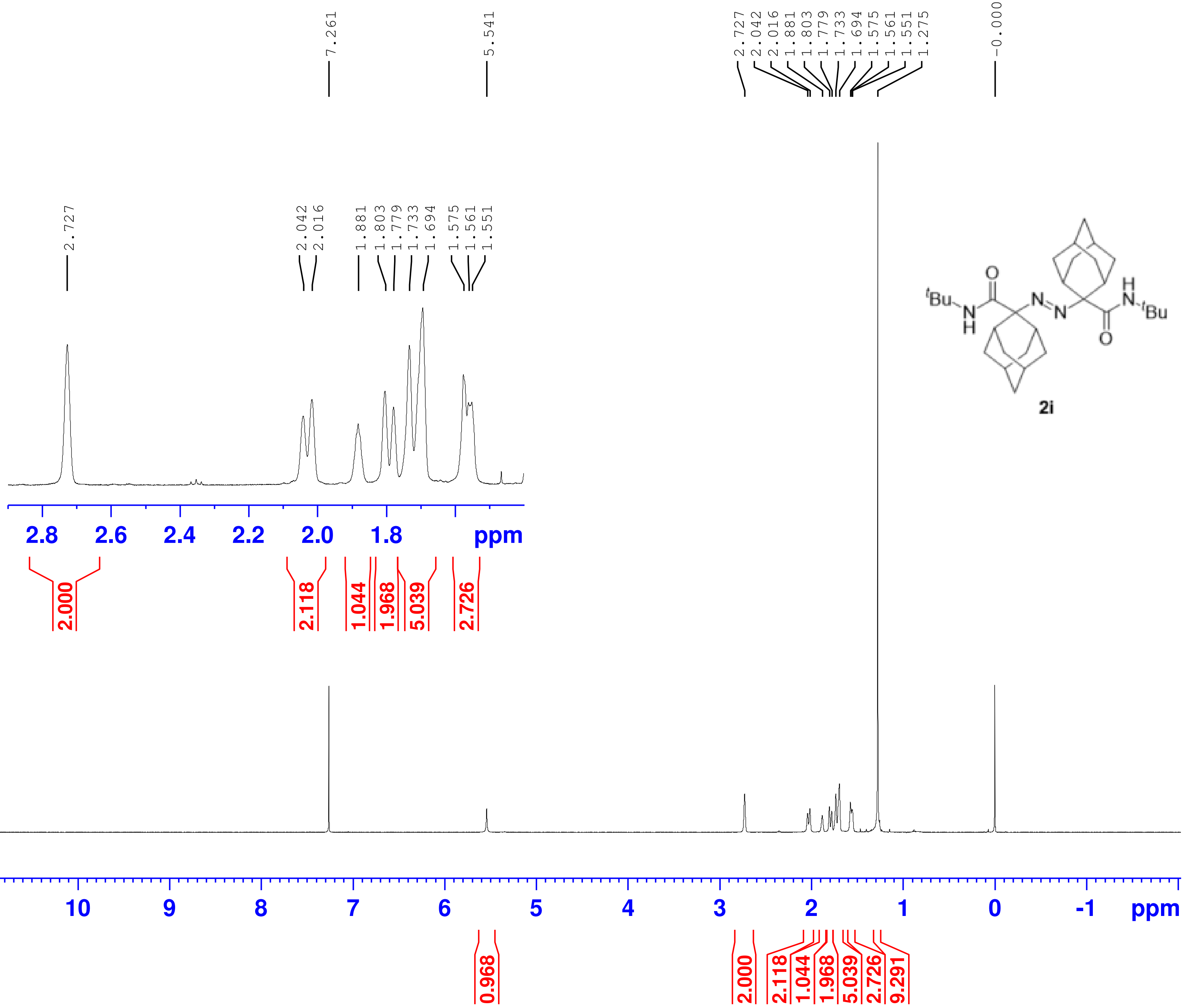

2i

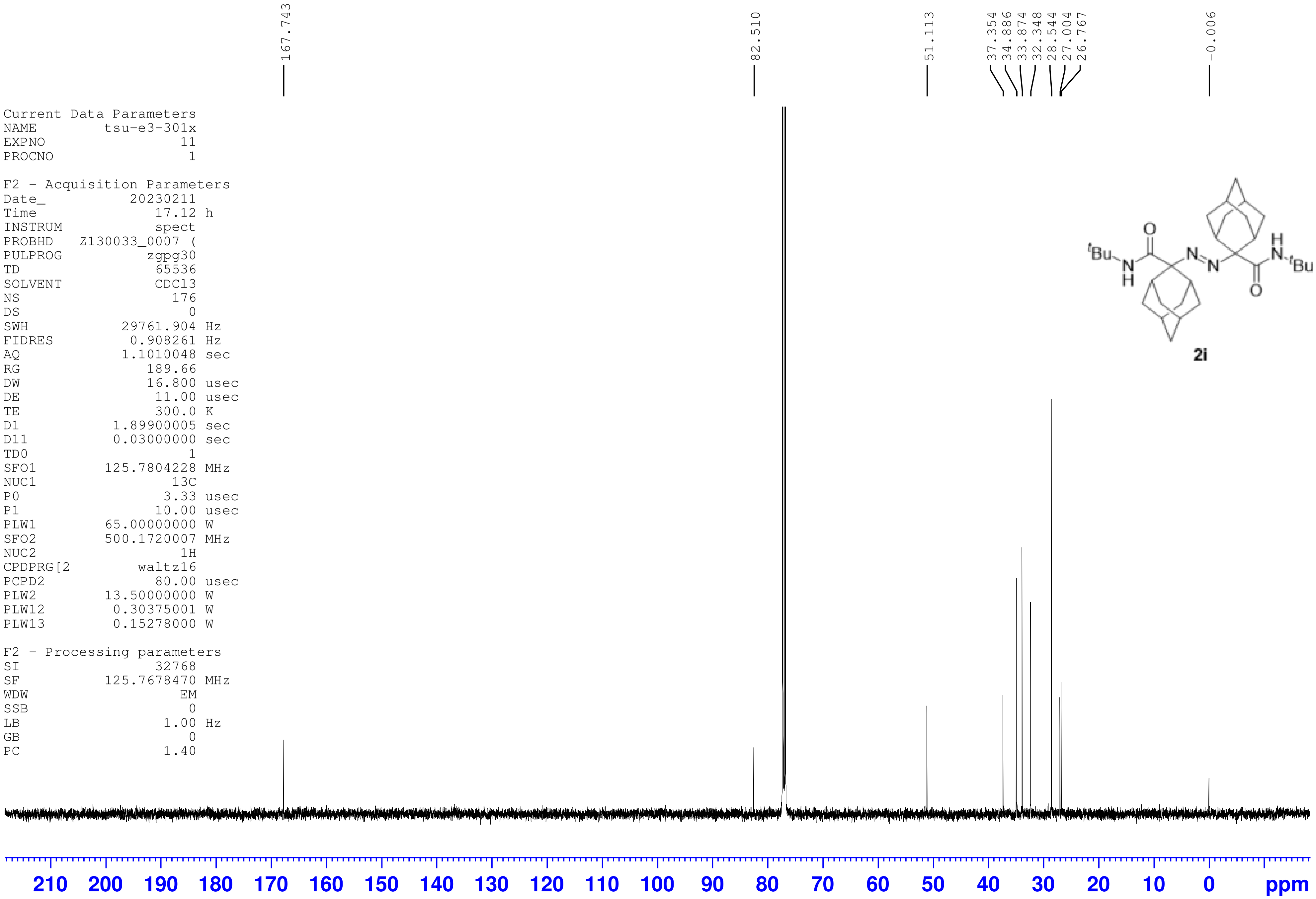


```
Current Data Parameters
NAME          tsu-e3-301x
EXPNO                  11
PROCNO                  1

F2 - Acquisition Parameters
Date_            20230211
Time                17.12 h
INSTRUM             spect
PROBHD   Z130033_0007 (
PULPROG           zgpg30
TD                 65536
SOLVENT            CDCl3
NS                   176
DS                     0
SWH            29761.904 Hz
FIDRES          0.908261 Hz
AQ             1.1010048 sec
RG                189.66
DW                16.800 usec
DE                 11.00 usec
TE                 300.0 K
D1            1.89900005 sec
D11           0.03000000 sec
TD0                    1
SFO1         125.7804228 MHz
NUC1                 13C
P0                  3.33 usec
P1                 10.00 usec
PLW1         65.00000000 W
SFO2         500.1720007 MHz
NUC2                  1H
CPDPRG[2         waltz16
PCPD2              80.00 usec
PLW2         13.50000000 W
PLW12         0.30375001 W
PLW13         0.15278000 W

F2 - Processing parameters
SI                 32768
SF           125.7678470 MHz
WDW                   EM
SSB                    0
LB                  1.00 Hz
GB                     0
PC                  1.40
```

2i

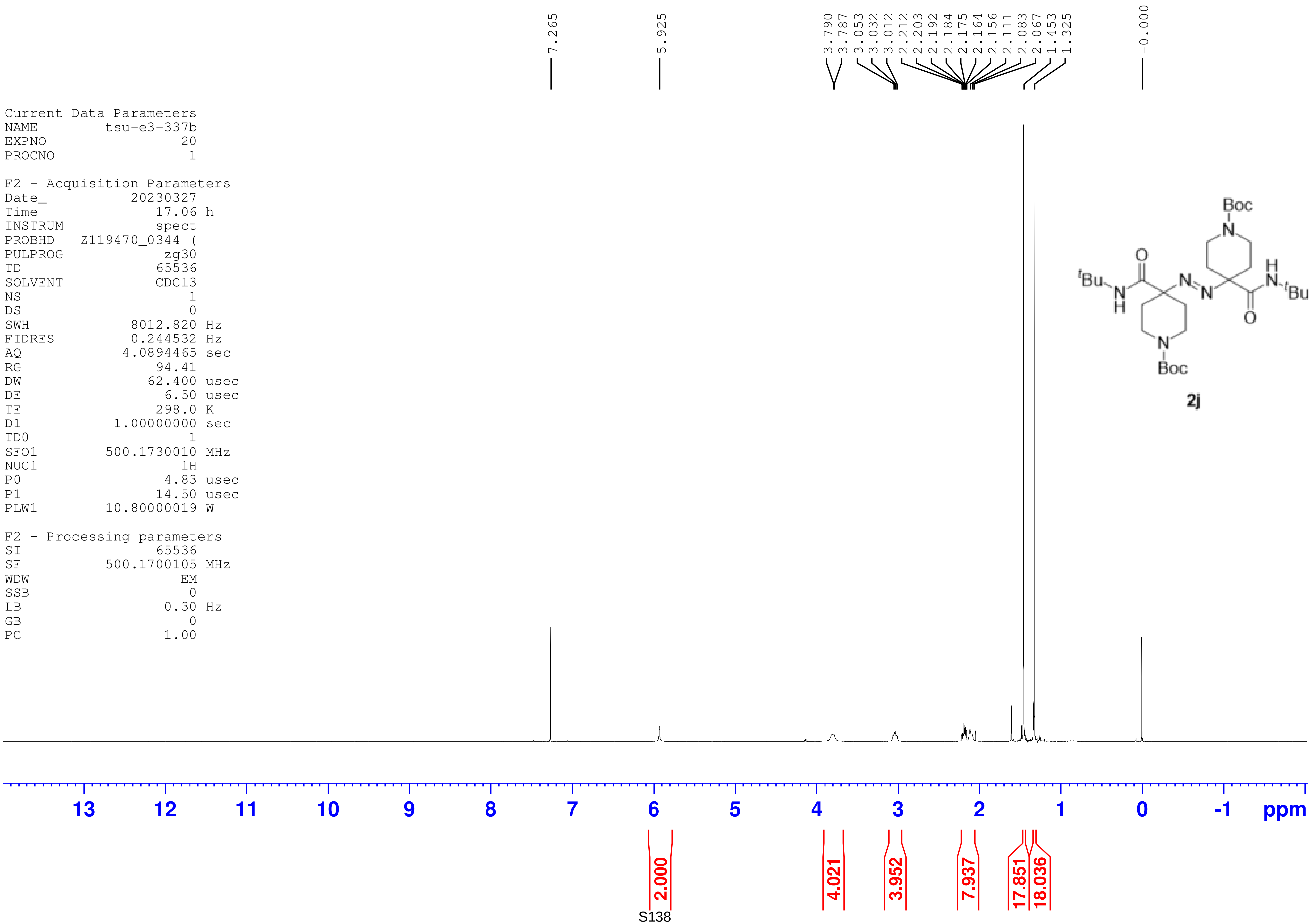
Current Data Parameters
NAME tsu-e3-337b
EXPNO 20
PROCNO 1
F2 - Acquisition Parameters
Date_ 20230327
Time 17.06 h
INSTRUM spect
PROBHD Z119470_0344 (
PULPROG zg30
TD 65536
SOLVENT CDCl3
NS 1
DS 0
SWH 8012.820 Hz
FIDRES 0.244532 Hz
AQ 4.0894465 sec
RG 94.41
DW 62.400 usec
DE 6.50 usec
TE 298.0 K
D1 1.00000000 sec
TD0 1
SFO1 500.1730010 MHz
NUC1 1H
P0 4.83 usec
P1 14.50 usec
PLW1 10.80000019 W
F2 - Processing parameters
SI 65536
SF 500.1700105 MHz
WDW EM
SSB 0
LB 0.30 Hz
GB 0
PC 1.00
7.265
5.925
3.790
3.787
3.053
3.032
3.012
2.212
2.203
2.192
2.184
2.175
2.164
2.156
2.111
2.083
2.067
1.453
1.325
-0.000
13 12 11 10 9 8 7 6 5 4 3 2 1 0 -1 ppm
2.000
4.021
3.952
7.937
17.851
18.036
Boc
N
N
N
H
N
tBu
O
tBu
N
H
O
N
Boc

2j

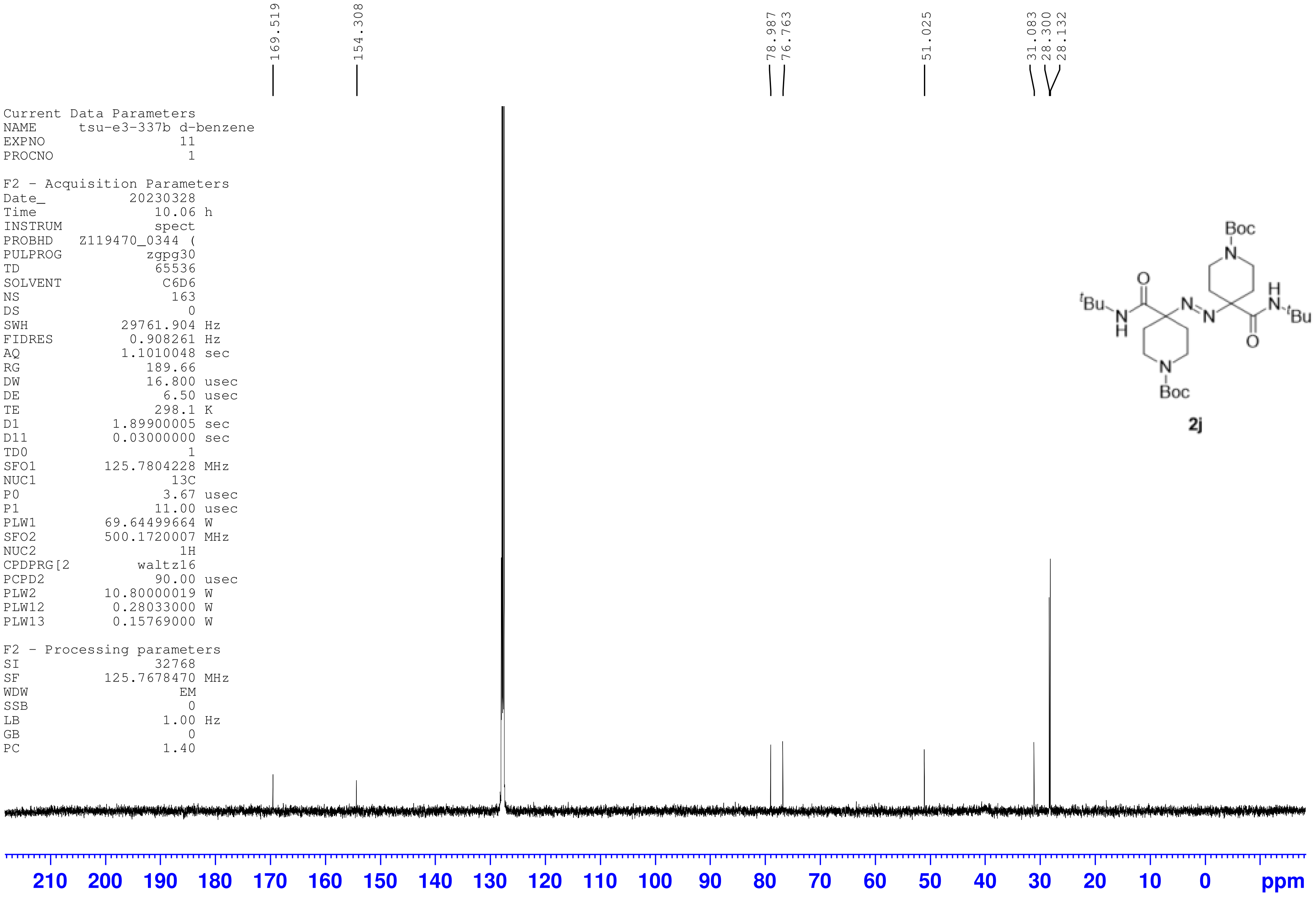
169.519
154.308
78.987
76.763
51.025
31.083
28.300
28.132
Current Data Parameters
NAME tsu-e3-337b d-benzene
EXPNO 11
PROCNO 1
F2 - Acquisition Parameters
Date_ 20230328
Time 10.06 h
INSTRUM spect
PROBHD Z119470_0344 (
PULPROG zgpg30
TD 65536
SOLVENT C6D6
NS 163
DS 0
SWH 29761.904 Hz
FIDRES 0.908261 Hz
AQ 1.1010048 sec
RG 189.66
DW 16.800 usec
DE 6.50 usec
TE 298.1 K
D1 1.89900005 sec
D11 0.03000000 sec
TD0 1
SFO1 125.7804228 MHz
NUC1 13C
P0 3.67 usec
P1 11.00 usec
PLW1 69.64499664 W
SFO2 500.1720007 MHz
NUC2 1H
CPDPRG[2 waltz16
PCPD2 90.00 usec
PLW2 10.80000019 W
PLW12 0.28033000 W
PLW13 0.15769000 W
F2 - Processing parameters
SI 32768
SF 125.7678470 MHz
WDW EM
SSB 0
LB 1.00 Hz
GB 0
PC 1.40
210 200 190 180 170 160 150 140 130 120 110 100 90 80 70 60 50 40 30 20 10 0 ppm
Boc
O
tBu
N
H
N
N
H
N
tBu
O
N
Boc

2j

```
Current Data Parameters
NAME          tsu-e3-339
EXPNO                 10
PROCNO                 1

F2 - Acquisition Parameters
Date_           20230328
Time               17.09 h
INSTRUM            spect
PROBHD   Z119470_0344 (
PULPROG             zg30
TD                 65536
SOLVENT            CDCl3
NS                     1
DS                     0
SWH             8012.820 Hz
FIDRES          0.244532 Hz
AQ             4.0894465 sec
RG                107.18
DW                62.400 usec
DE                  6.50 usec
TE                 298.0 K
D1            1.00000000 sec
TD0                    1
SFO1         500.1730010 MHz
NUC1                  1H
P0                  4.83 usec
P1                 14.50 usec
PLW1         10.80000019 W

F2 - Processing parameters
SI                 65536
SF           500.1700109 MHz
WDW                   EM
SSB                    0
LB                  0.30 Hz
GB                     0
PC                  1.00
```

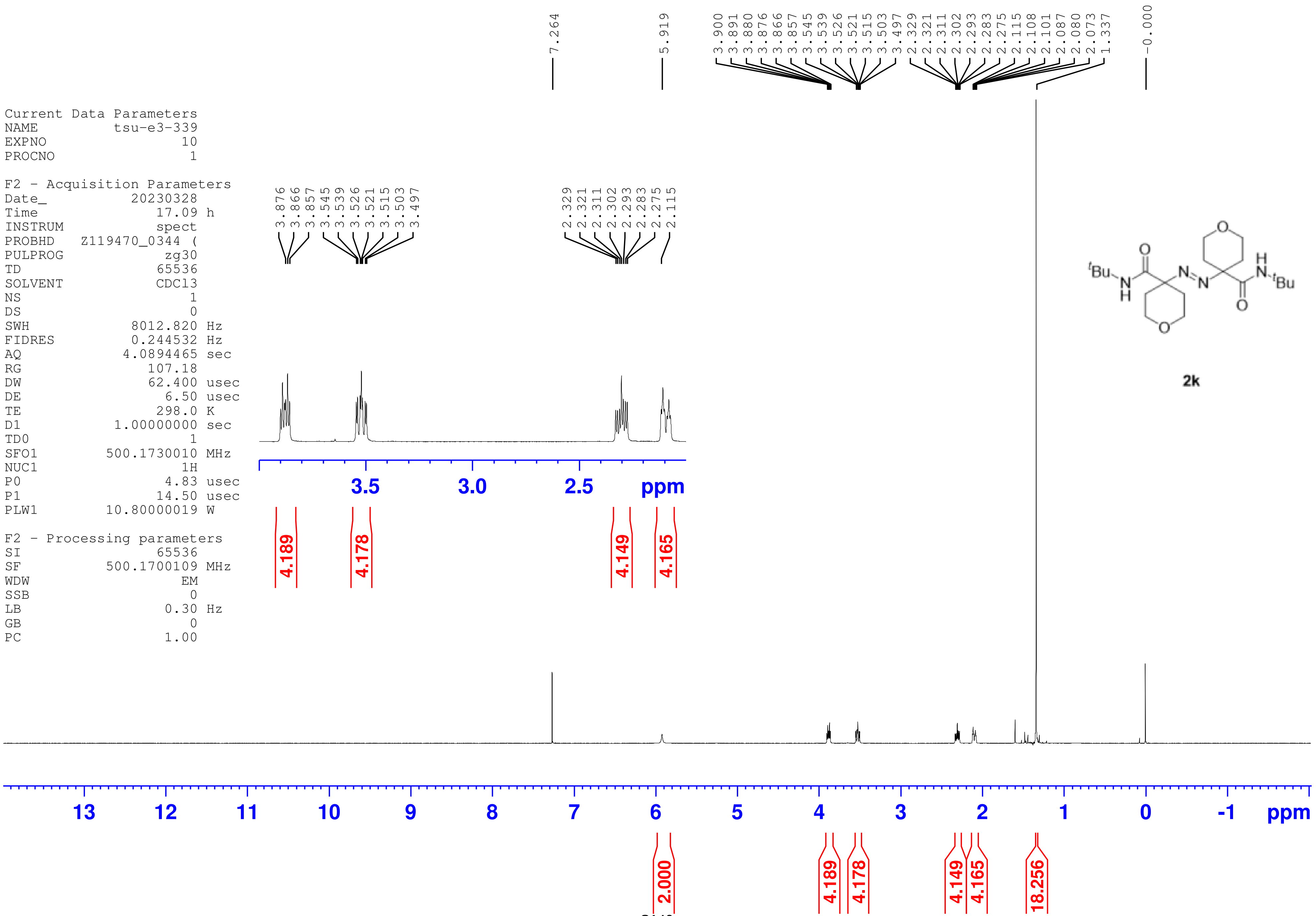

2k

Current Data Parameters
NAME tsu-e3-339
EXPNO 11
PROCNO 1

F2 - Acquisition Parameters
Date_ 20230328
Time 17.17 h
INSTRUM spect
PROBHD Z119470_0344 (
PULPROG zgpg30
TD 65536
SOLVENT CDCl3
NS 129
DS 0
SWH 29761.904 Hz
FIDRES 0.908261 Hz
AQ 1.1010048 sec
RG 189.66
DW 16.800 usec
DE 6.50 usec
TE 298.1 K
D1 1.89900005 sec
D11 0.03000000 sec
TD0 1
SFO1 125.7804228 MHz
NUC1 13C
P0 3.67 usec
P1 11.00 usec
PLW1 69.64499664 W
SFO2 500.1720007 MHz
NUC2 1H
CPDPRG[2 waltz16
PCPD2 90.00 usec
PLW2 10.80000019 W
PLW12 0.28033000 W
PLW13 0.15769000 W

F2 - Processing parameters
SI 32768
SF 125.7678470 MHz
WDW EM
SSB 0
LB 1.00 Hz
GB 0
PC 1.40

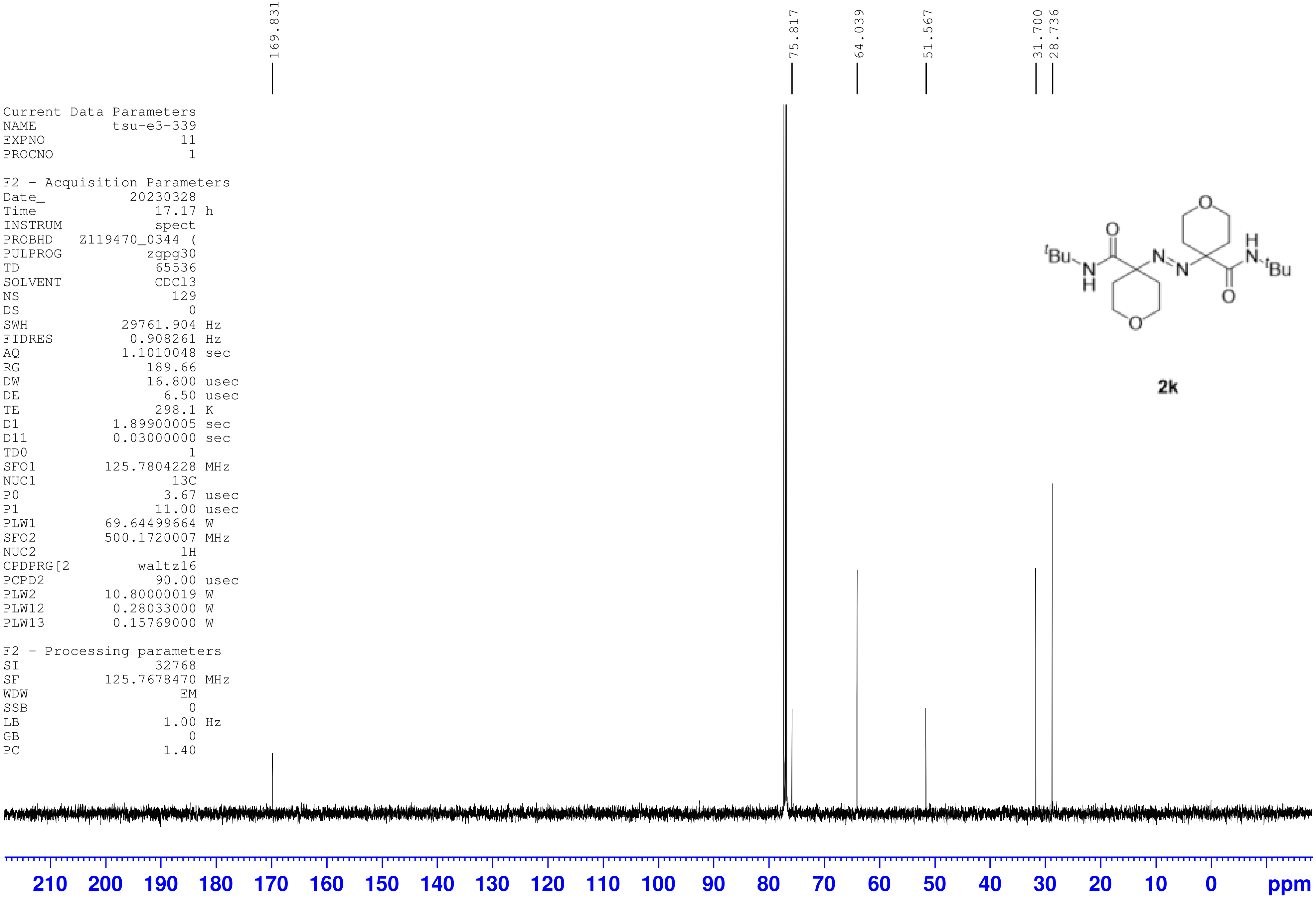

2k

Current Data Parameters
NAME tsu-e3-344 dmso
EXPNO 10
PROCNO 1

F2 - Acquisition Parameters
Date_ 20230401
Time 9.39 h
INSTRUM spect
PROBHD Z119470_0344 (
PULPROG zg30
TD 65536
SOLVENT DMSO
NS 1
DS 0
SWH 8012.820 Hz
FIDRES 0.244532 Hz
AQ 4.0894465 sec
RG 58.77
DW 62.400 usec
DE 6.50 usec
TE 298.0 K
D1 1.00000000 sec
TD0 1
SFO1 500.1730010 MHz
NUC1 1H
P0 4.83 usec
P1 14.50 usec
PLW1 10.80000019 W

F2 - Processing parameters
SI 65536
SF 500.1700000 MHz
WDW EM
SSB 0
LB 0.30 Hz
GB 0
PC 1.00

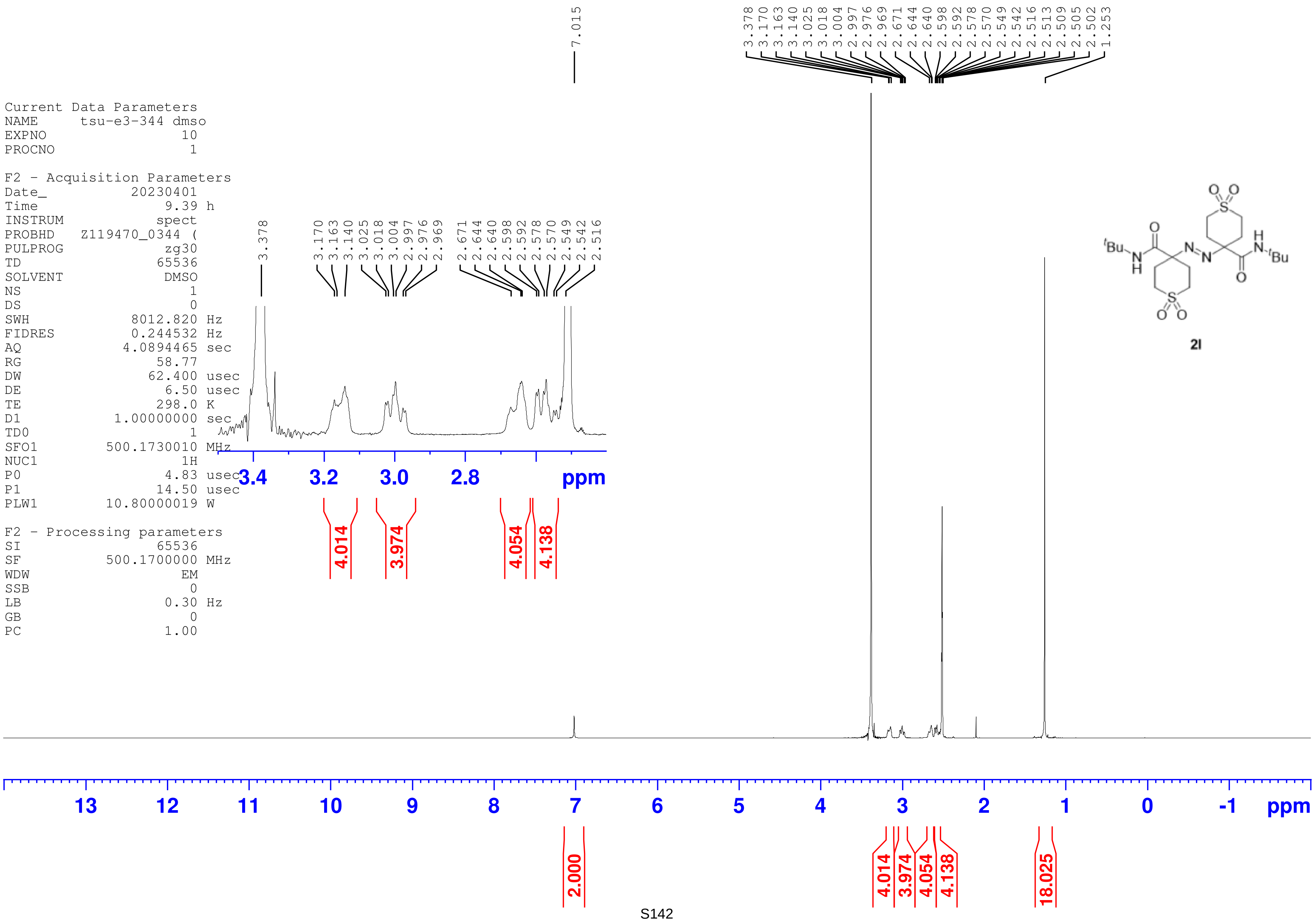

2l

S142

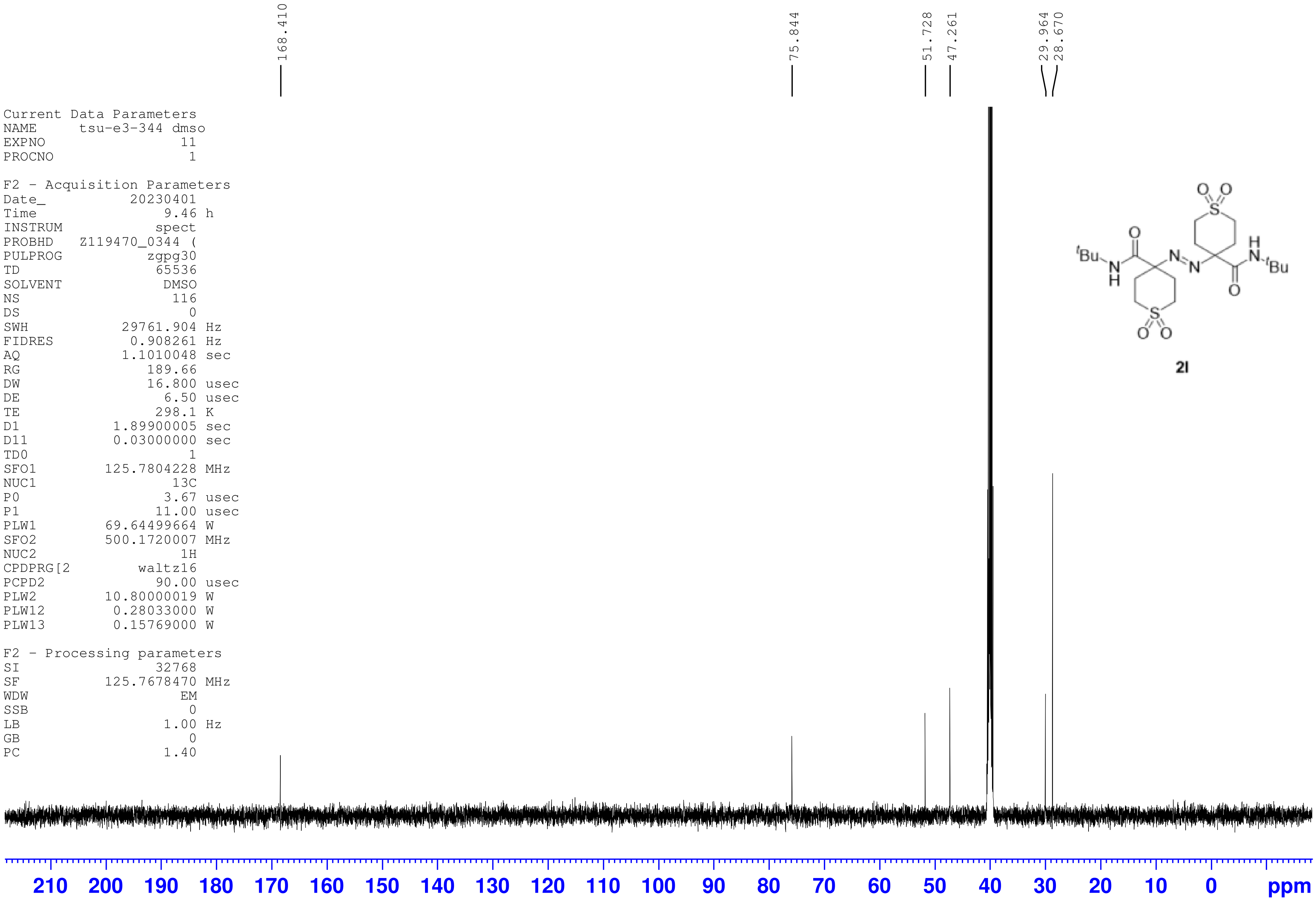
168.410
75.844
51.728
47.261
29.964
28.670
Current Data Parameters
NAME tsu-e3-344 dmso
EXPNO 11
PROCNO 1
F2 - Acquisition Parameters
Date_ 20230401
Time 9.46 h
INSTRUM spect
PROBHD Z119470_0344 (
PULPROG zgpg30
TD 65536
SOLVENT DMSO
NS 116
DS 0
SWH 29761.904 Hz
FIDRES 0.908261 Hz
AQ 1.1010048 sec
RG 189.66
DW 16.800 usec
DE 6.50 usec
TE 298.1 K
D1 1.89900005 sec
D11 0.03000000 sec
TD0 1
SFO1 125.7804228 MHz
NUC1 13C
P0 3.67 usec
P1 11.00 usec
PLW1 69.64499664 W
SFO2 500.1720007 MHz
NUC2 1H
CPDPRG[2 waltz16
PCPD2 90.00 usec
PLW2 10.80000019 W
PLW12 0.28033000 W
PLW13 0.15769000 W
F2 - Processing parameters
SI 32768
SF 125.7678470 MHz
WDW EM
SSB 0
LB 1.00 Hz
GB 0
PC 1.40
210 200 190 180 170 160 150 140 130 120 110 100 90 80 70 60 50 40 30 20 10 0 ppm
tBu
N
H
O
N
N
S
O
O
S
O
O
O
H
N
tBu

2l

```
Current Data Parameters
NAME        tsu-e3-370ab
EXPNO                 10
PROCNO                 1

F2 - Acquisition Parameters
Date_           20230418
Time               15.10 h
INSTRUM            spect
PROBHD   Z119470_0344 (
PULPROG             zg30
TD                 65536
SOLVENT            CDCl3
NS                     1
DS                     0
SWH             8012.820 Hz
FIDRES          0.244532 Hz
AQ             4.0894465 sec
RG                116.65
DW                62.400 usec
DE                  6.50 usec
TE                 298.0 K
D1            1.00000000 sec
TD0                    1
SFO1         500.1730010 MHz
NUC1                  1H
P0                  4.83 usec
P1                 14.50 usec
PLW1         10.80000019 W

F2 - Processing parameters
SI                 65536
SF           500.1700126 MHz
WDW                   EM
SSB                    0
LB                  0.30 Hz
GB                     0
PC                  1.00
```

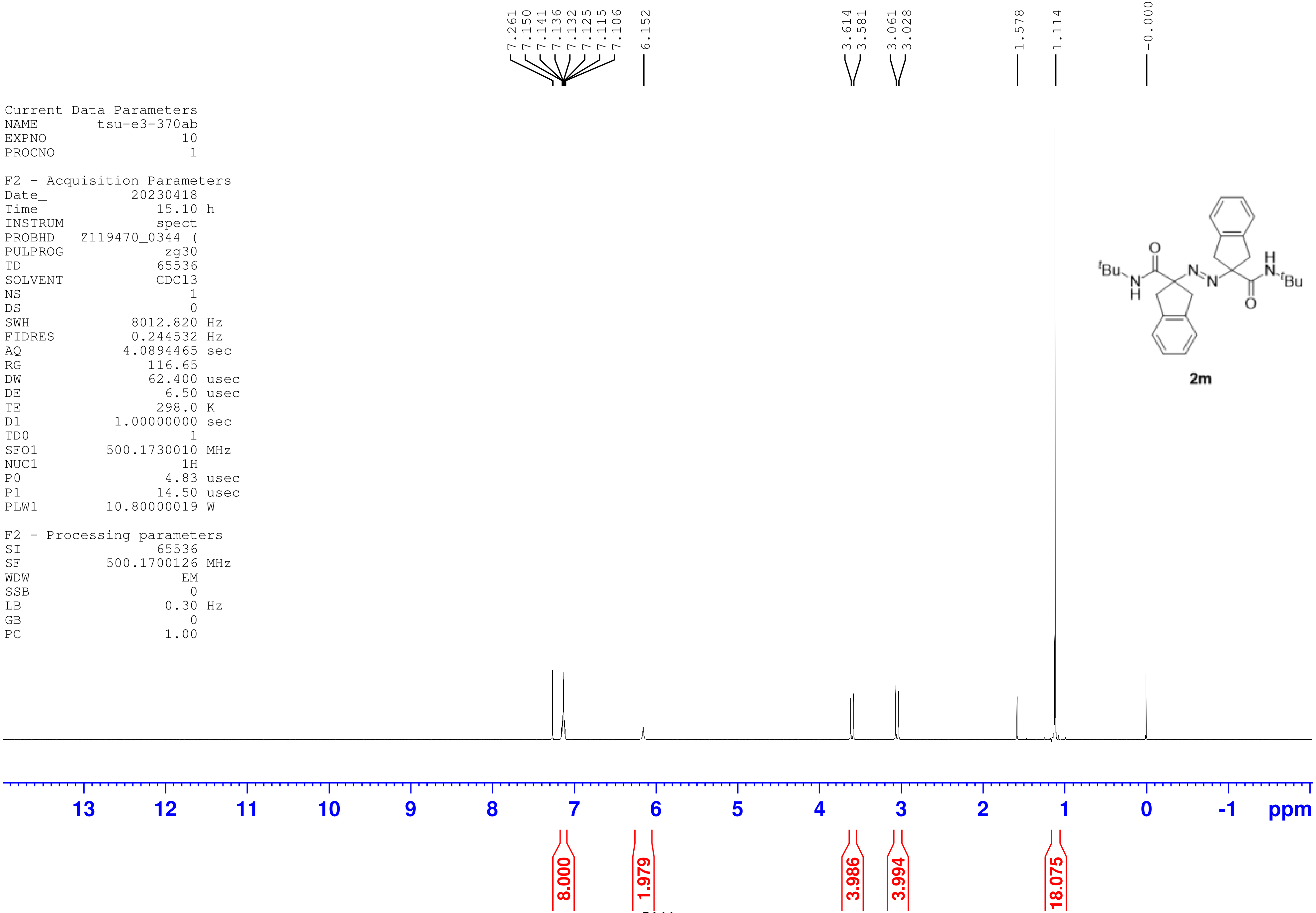

2m

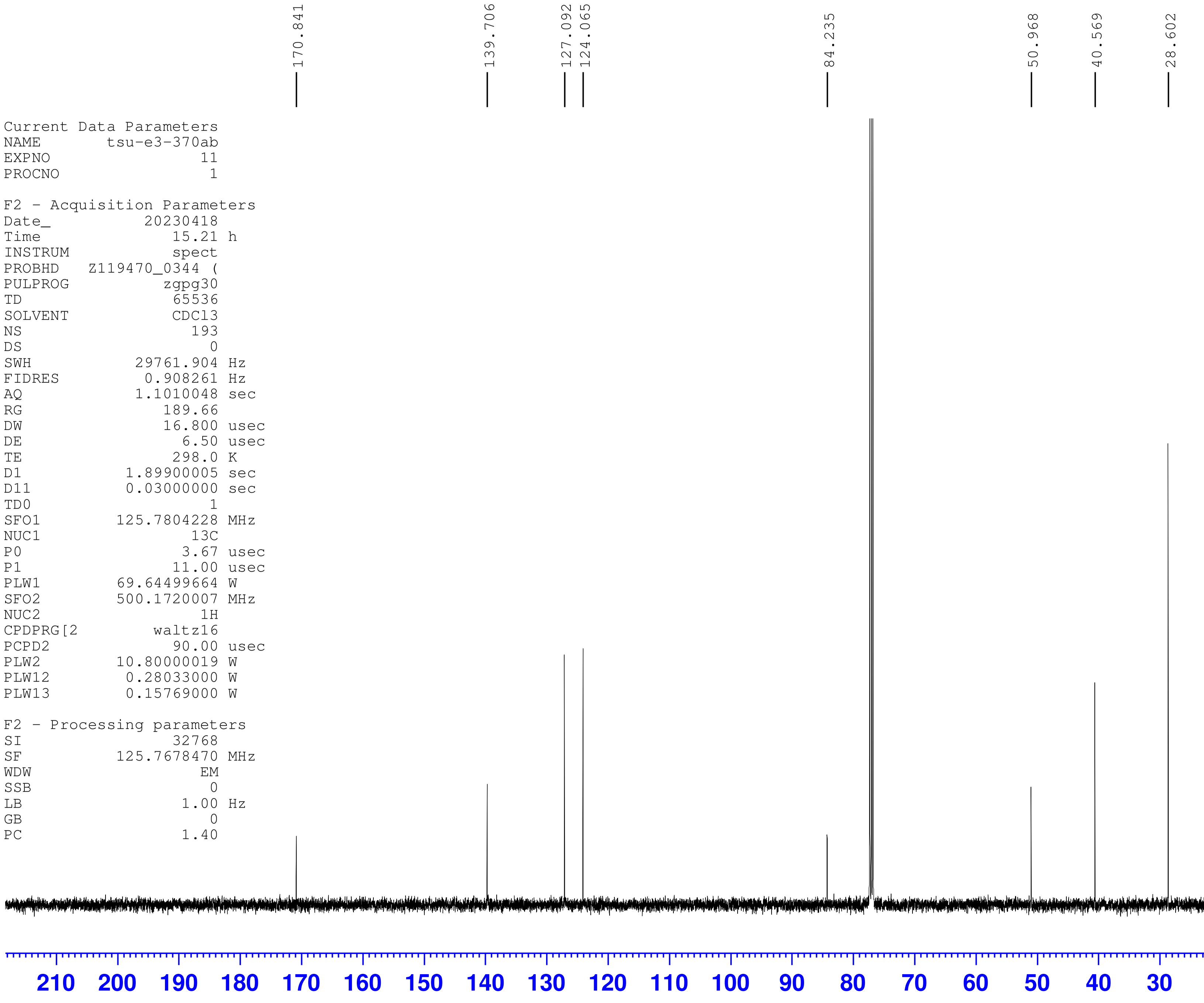

170.841
139.706
127.092
124.065
84.235
50.968
40.569
28.602
0.004
Current Data Parameters
NAME tsu-e3-370ab
EXPNO 11
PROCNO 1
F2 - Acquisition Parameters
Date_ 20230418
Time 15.21 h
INSTRUM spect
PROBHD Z119470_0344 (
PULPROG zgpg30
TD 65536
SOLVENT CDCl3
NS 193
DS 0
SWH 29761.904 Hz
FIDRES 0.908261 Hz
AQ 1.1010048 sec
RG 189.66
DW 16.800 usec
DE 6.50 usec
TE 298.0 K
D1 1.89900005 sec
D11 0.03000000 sec
TD0 1
SFO1 125.7804228 MHz
NUC1 13C
P0 3.67 usec
P1 11.00 usec
PLW1 69.64499664 W
SFO2 500.1720007 MHz
NUC2 1H
CPDPRG[2 waltz16
PCPD2 90.00 usec
PLW2 10.80000019 W
PLW12 0.28033000 W
PLW13 0.15769000 W
F2 - Processing parameters
SI 32768
SF 125.7678470 MHz
WDW EM
SSB 0
LB 1.00 Hz
GB 0
PC 1.40
210 200 190 180 170 160 150 140 130 120 110 100 90 80 70 60 50 40 30 20 10 0 ppm

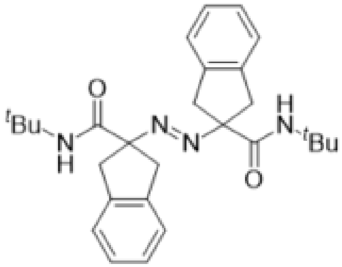

O
tBu
N
H
N
N
H
N
tBu
O

2m

```
Current Data Parameters
NAME          tsu-e3-371c
EXPNO                  20
PROCNO                  1

F2 - Acquisition Parameters
Date_            20230420
Time                16.03 h
INSTRUM             spect
PROBHD   Z119470_0344 (
PULPROG              zg30
TD                  65536
SOLVENT             CDCl3
NS                      1
DS                      0
SWH              8012.820 Hz
FIDRES           0.244532 Hz
AQ              4.0894465 sec
RG                 107.18
DW                 62.400 usec
DE                   6.50 usec
TE                  298.0 K
D1             1.00000000 sec
TD0                     1
SFO1          500.1730010 MHz
NUC1                   1H
P0                   4.83 usec
P1                  14.50 usec
PLW1          10.80000019 W

F2 - Processing parameters
SI                  65536
SF            500.1700131 MHz
WDW                    EM
SSB                     0
LB                   0.30 Hz
GB                      0
PC                   1.00
```

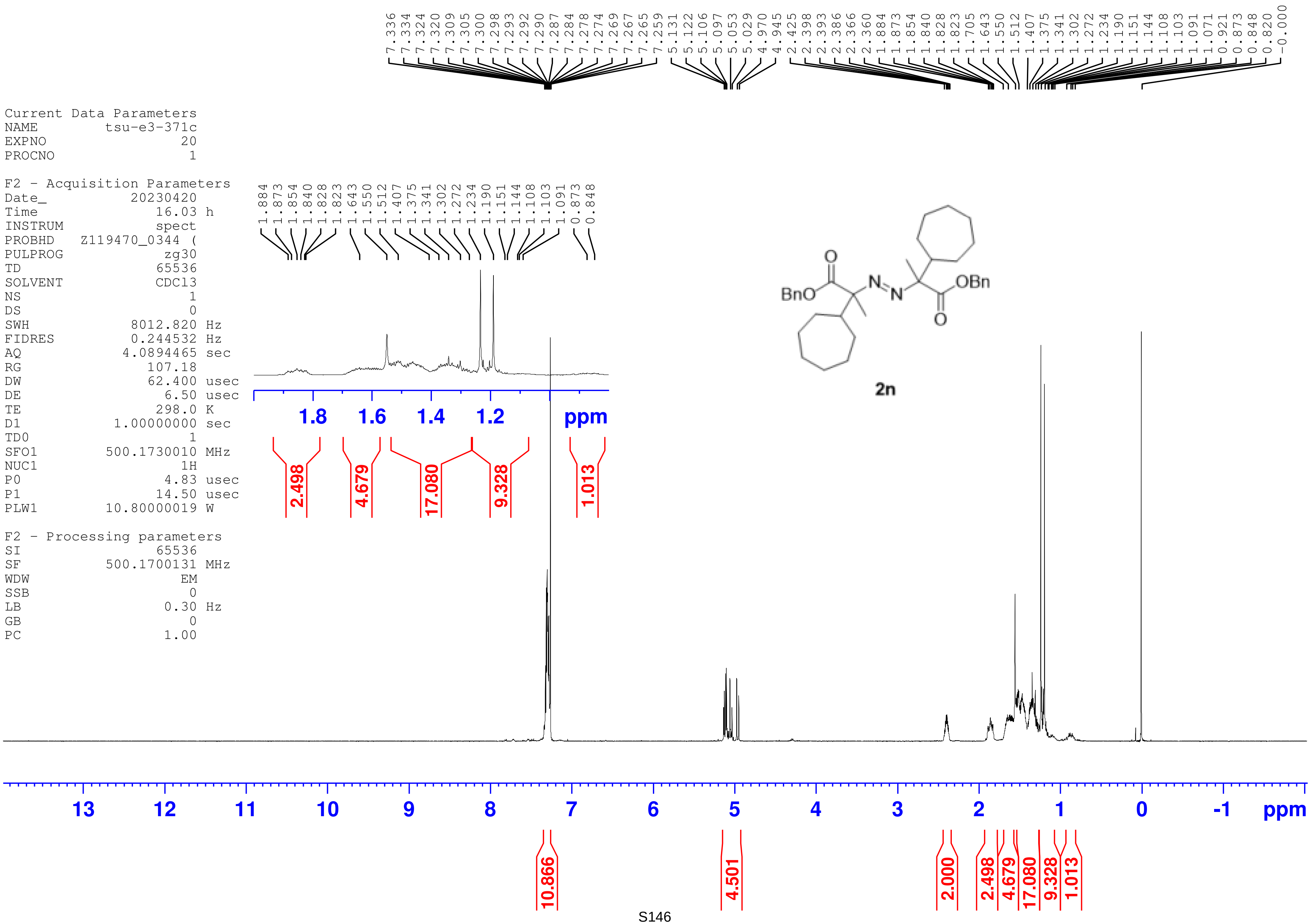

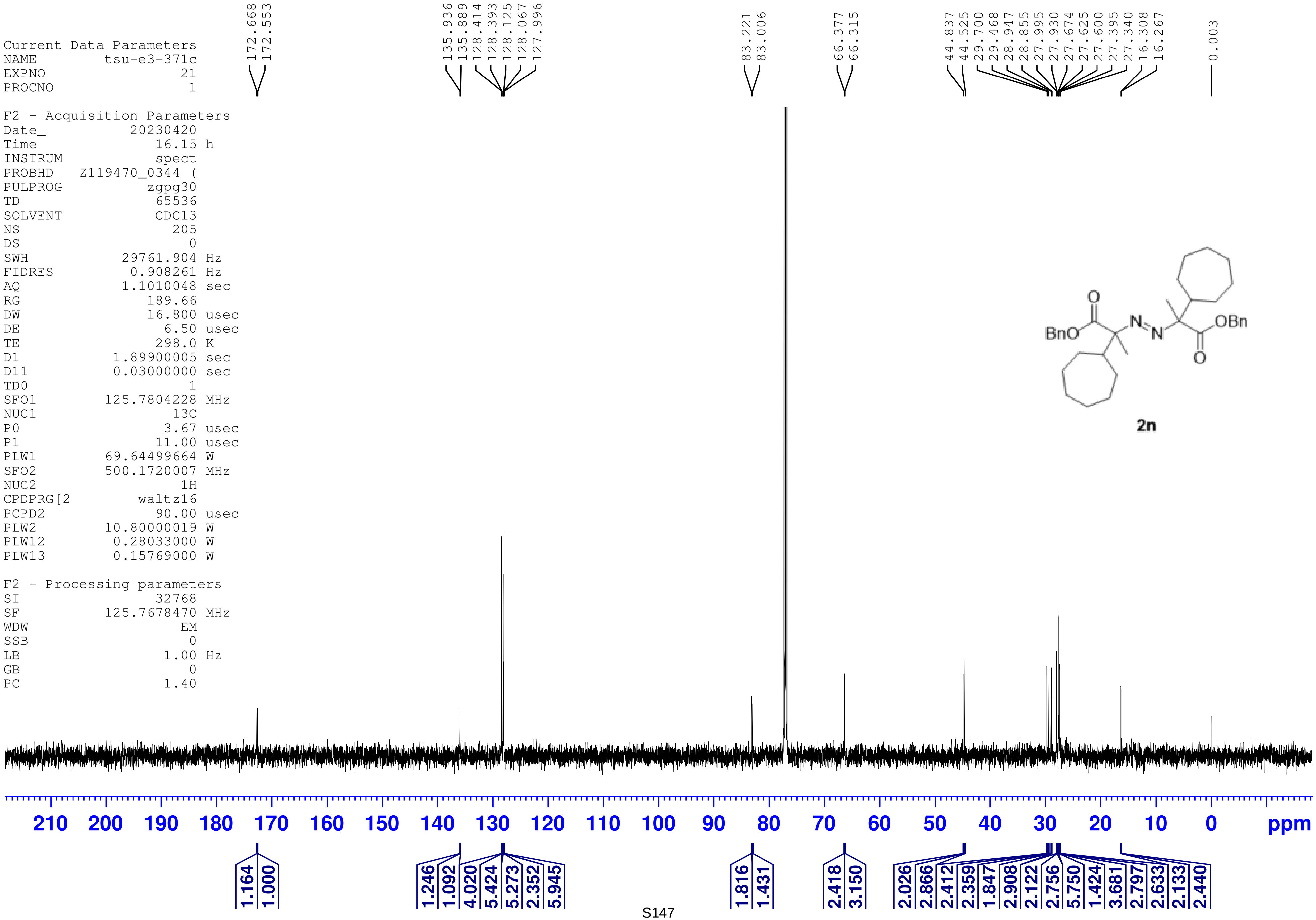
Current Data Parameters
NAME tsu-e3-371c
EXPNO 21
PROCNO 1
F2 - Acquisition Parameters
Date_ 20230420
Time 16.15 h
INSTRUM spect
PROBHD Z119470_0344 (
PULPROG zgpg30
TD 65536
SOLVENT CDCl3
NS 205
DS 0
SWH 29761.904 Hz
FIDRES 0.908261 Hz
AQ 1.1010048 sec
RG 189.66
DW 16.800 usec
DE 6.50 usec
TE 298.0 K
D1 1.89900005 sec
D11 0.03000000 sec
TD0 1
SFO1 125.7804228 MHz
NUC1 13C
P0 3.67 usec
P1 11.00 usec
PLW1 69.64499664 W
SFO2 500.1720007 MHz
NUC2 1H
CPDPRG[2 waltz16
PCPD2 90.00 usec
PLW2 10.80000019 W
PLW12 0.28033000 W
PLW13 0.15769000 W
F2 - Processing parameters
SI 32768
SF 125.7678470 MHz
WDW EM
SSB 0
LB 1.00 Hz
GB 0
PC 1.40
172.668
172.553
135.936
135.889
128.414
128.393
128.125
128.067
127.996
83.221
83.006
66.377
66.315
44.837
44.525
29.700
29.468
28.947
28.855
27.995
27.930
27.674
27.625
27.600
27.395
27.340
16.308
16.267
0.003
210 200 190 180 170 160 150 140 130 120 110 100 90 80 70 60 50 40 30 20 10 0 ppm
1.164
1.000
1.246
1.092
4.020
5.424
5.273
2.352
5.945
1.816
1.431
2.418
3.150
2.026
2.866
2.412
2.359
1.847
2.908
2.122
2.756
5.750
1.424
3.681
2.797
2.633
2.133
2.440
O
BnO
N
N
OBn
O

2n

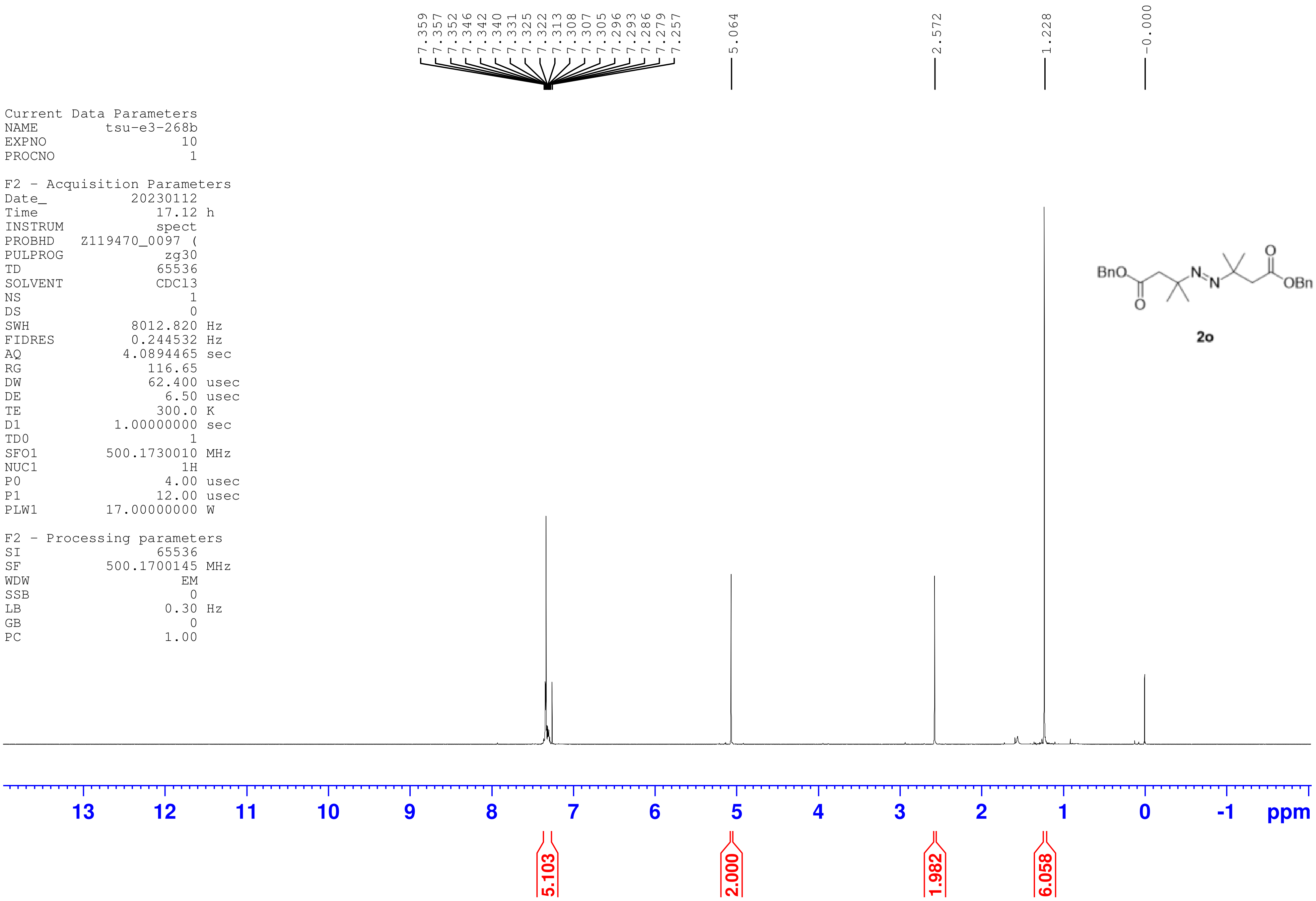

2o

```
Current Data Parameters
NAME           tsu-e3-268b
EXPNO                   10
PROCNO                   1

F2 - Acquisition Parameters
Date_             20230112
Time                 17.12 h
INSTRUM              spect
PROBHD    Z119470_0097 (
PULPROG               zg30
TD                   65536
SOLVENT              CDCl3
NS                       1
DS                       0
SWH               8012.820 Hz
FIDRES            0.244532 Hz
AQ               4.0894465 sec
RG                  116.65
DW                  62.400 usec
DE                    6.50 usec
TE                   300.0 K
D1              1.00000000 sec
TD0                      1
SFO1           500.1730010 MHz
NUC1                    1H
P0                    4.00 usec
P1                   12.00 usec
PLW1           17.00000000 W

F2 - Processing parameters
SI                   65536
SF             500.1700145 MHz
WDW                     EM
SSB                      0
LB                    0.30 Hz
GB                       0
PC                    1.00
```

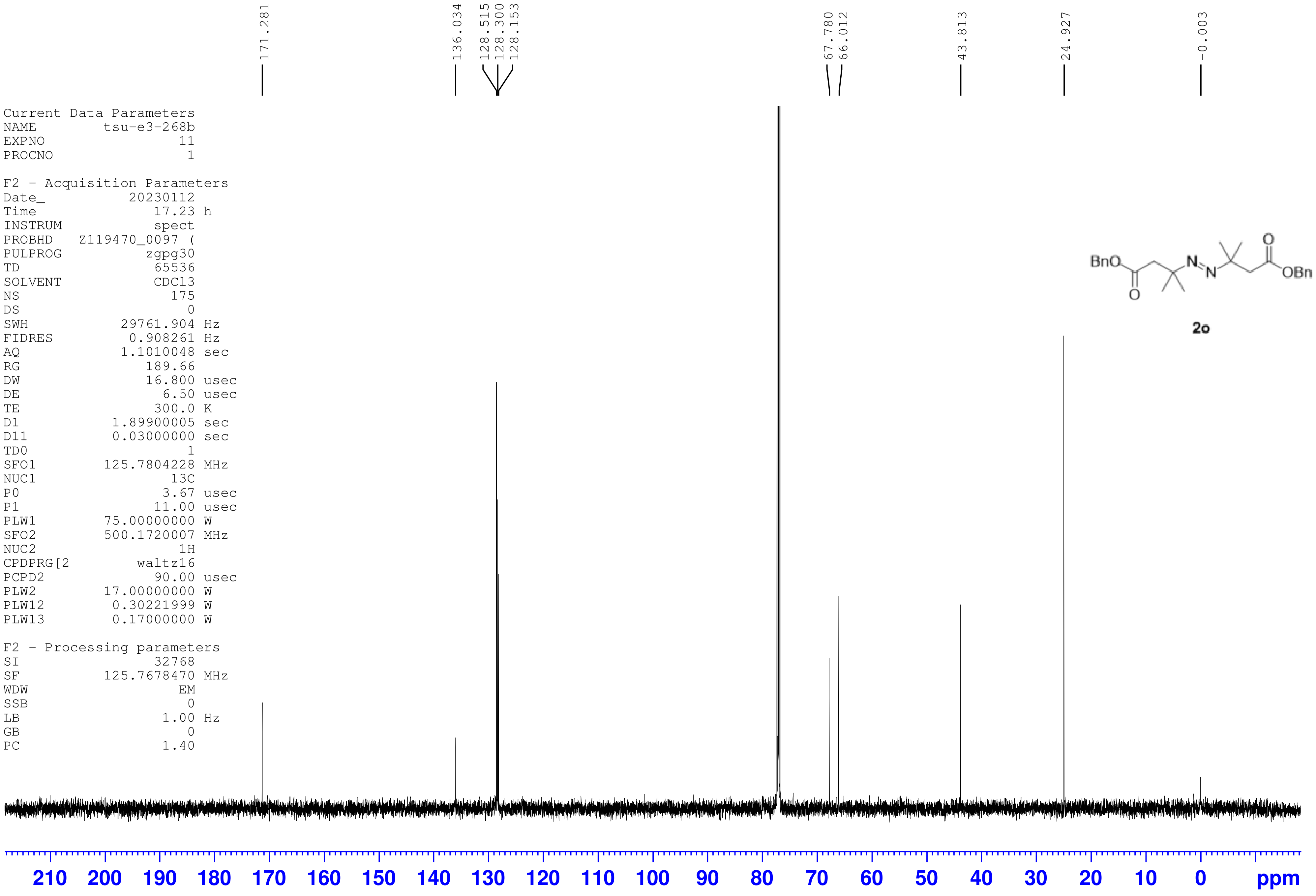
171.281
136.034
128.515
128.300
128.153
67.780
66.012
43.813
24.927
-0.003
Current Data Parameters
NAME tsu-e3-268b
EXPNO 11
PROCNO 1
F2 - Acquisition Parameters
Date_ 20230112
Time 17.23 h
INSTRUM spect
PROBHD Z119470_0097 (
PULPROG zgpg30
TD 65536
SOLVENT CDCl3
NS 175
DS 0
SWH 29761.904 Hz
FIDRES 0.908261 Hz
AQ 1.1010048 sec
RG 189.66
DW 16.800 usec
DE 6.50 usec
TE 300.0 K
D1 1.89900005 sec
D11 0.03000000 sec
TD0 1
SFO1 125.7804228 MHz
NUC1 13C
P0 3.67 usec
P1 11.00 usec
PLW1 75.00000000 W
SFO2 500.1720007 MHz
NUC2 1H
CPDPRG[2 waltz16
PCPD2 90.00 usec
PLW2 17.00000000 W
PLW12 0.30221999 W
PLW13 0.17000000 W
F2 - Processing parameters
SI 32768
SF 125.7678470 MHz
WDW EM
SSB 0
LB 1.00 Hz
GB 0
PC 1.40
210 200 190 180 170 160 150 140 130 120 110 100 90 80 70 60 50 40 30 20 10 0 ppm
BnO
O
N
N
O
OBn

2o

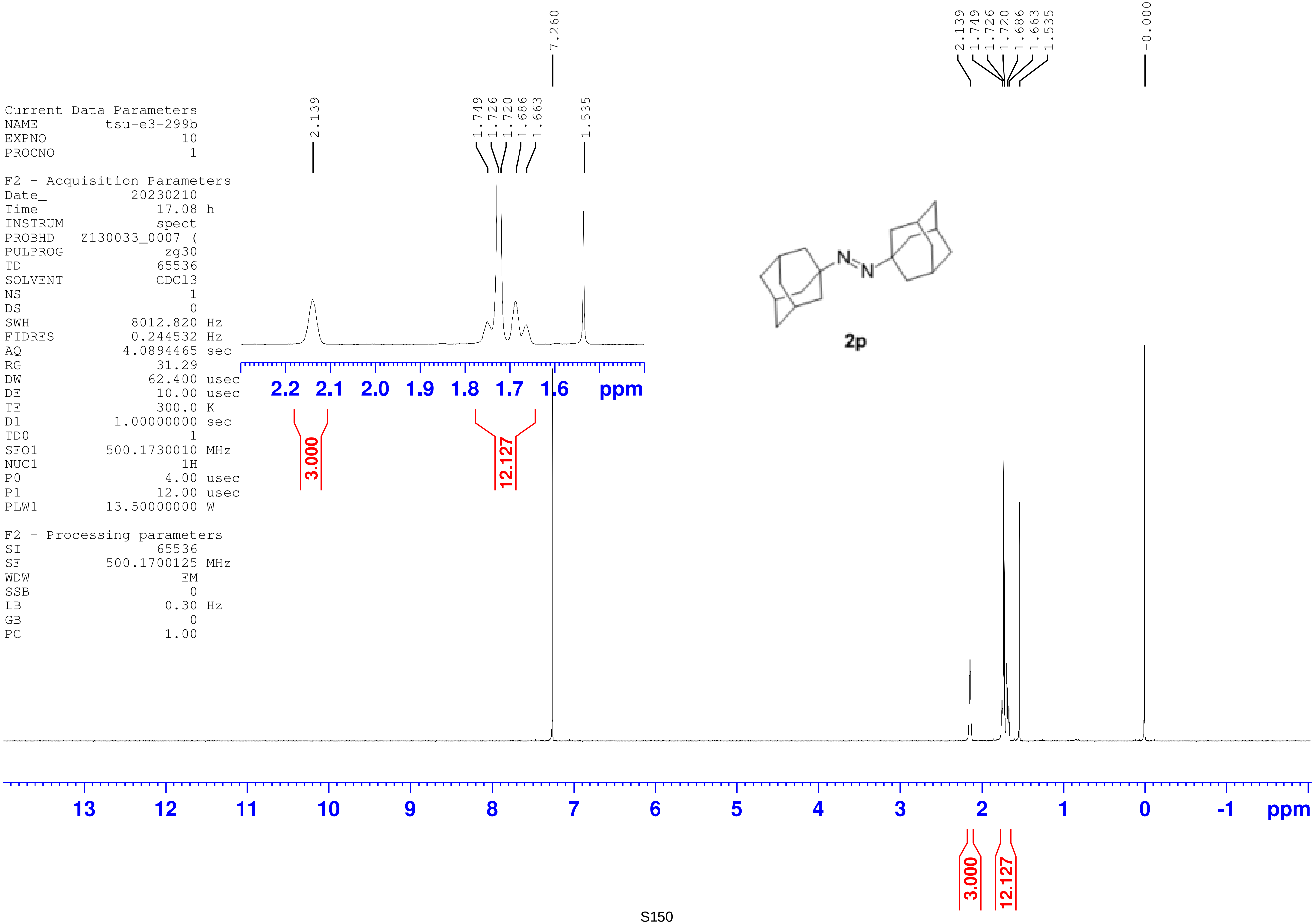
Current Data Parameters
NAME tsu-e3-299b
EXPNO 10
PROCNO 1
F2 - Acquisition Parameters
Date_ 20230210
Time 17.08 h
INSTRUM spect
PROBHD Z130033_0007 (
PULPROG zg30
TD 65536
SOLVENT CDCl3
NS 1
DS 0
SWH 8012.820 Hz
FIDRES 0.244532 Hz
AQ 4.0894465 sec
RG 31.29
DW 62.400 usec
DE 10.00 usec
TE 300.0 K
D1 1.00000000 sec
TD0 1
SFO1 500.1730010 MHz
NUC1 1H
P0 4.00 usec
P1 12.00 usec
PLW1 13.50000000 W
F2 - Processing parameters
SI 65536
SF 500.1700125 MHz
WDW EM
SSB 0
LB 0.30 Hz
GB 0
PC 1.00
2.139
1.749
1.726
1.720
1.686
1.663
1.535
7.260
-0.000
3.000
12.127
2.2 2.1 2.0 1.9 1.8 1.7 1.6 ppm
13 12 11 10 9 8 7 6 5 4 3 2 1 0 -1 ppm

2p

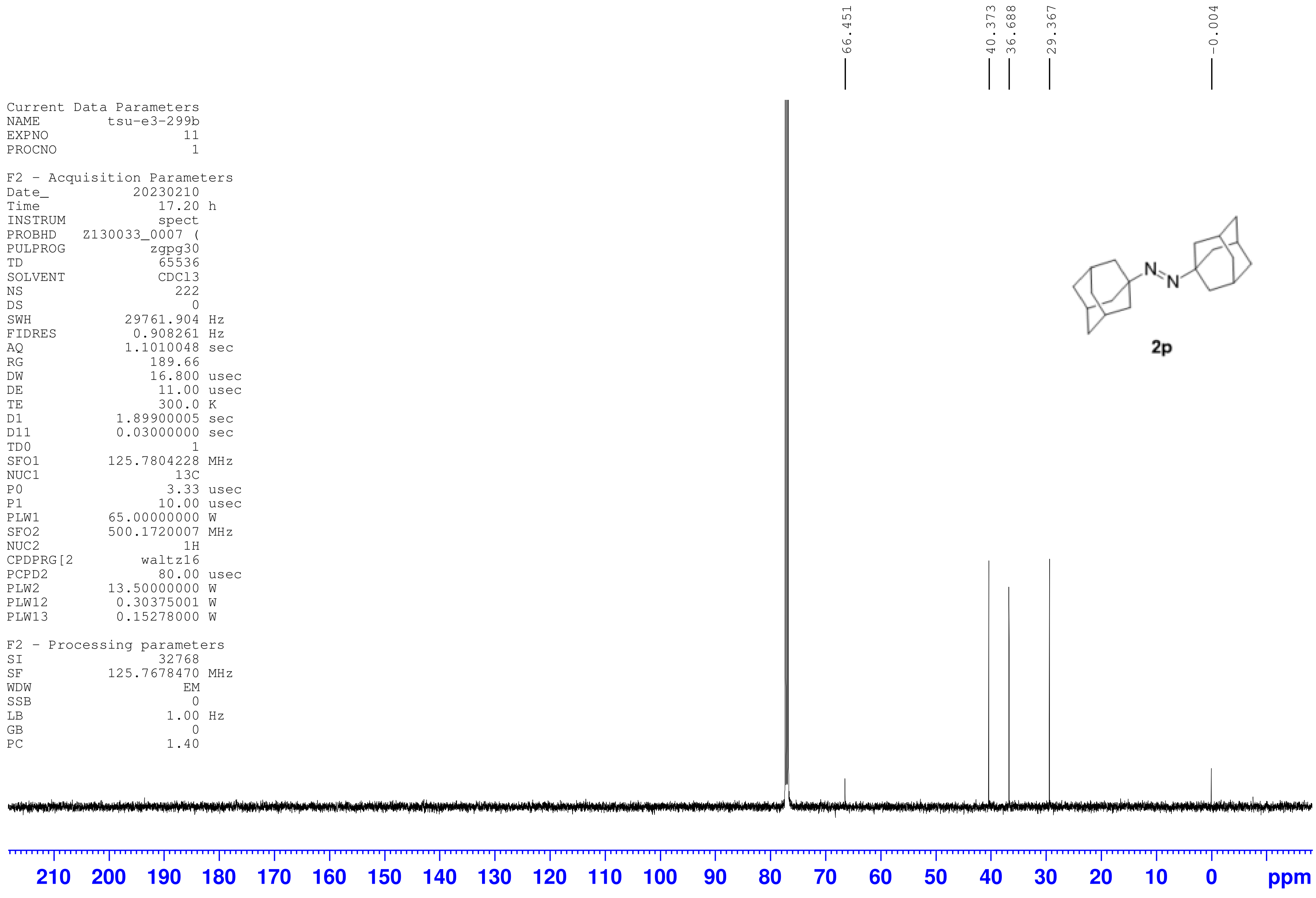
66.451
40.373
36.688
29.367
-0.004
Current Data Parameters
NAME tsu-e3-299b
EXPNO 11
PROCNO 1
F2 - Acquisition Parameters
Date_ 20230210
Time 17.20 h
INSTRUM spect
PROBHD Z130033_0007 (
PULPROG zgpg30
TD 65536
SOLVENT CDCl3
NS 222
DS 0
SWH 29761.904 Hz
FIDRES 0.908261 Hz
AQ 1.1010048 sec
RG 189.66
DW 16.800 usec
DE 11.00 usec
TE 300.0 K
D1 1.89900005 sec
D11 0.03000000 sec
TD0 1
SFO1 125.7804228 MHz
NUC1 13C
P0 3.33 usec
P1 10.00 usec
PLW1 65.00000000 W
SFO2 500.1720007 MHz
NUC2 1H
CPDPRG[2 waltz16
PCPD2 80.00 usec
PLW2 13.50000000 W
PLW12 0.30375001 W
PLW13 0.15278000 W
F2 - Processing parameters
SI 32768
SF 125.7678470 MHz
WDW EM
SSB 0
LB 1.00 Hz
GB 0
PC 1.40
N
N
210 200 190 180 170 160 150 140 130 120 110 100 90 80 70 60 50 40 30 20 10 0 ppm

2p

Current Data Parameters
NAME tsu-e3-283aaa
EXPNO 10
PROCNO 1

F2 - Acquisition Parameters
Date_ 20230127
Time 10.36 h
INSTRUM spect
PROBHD Z119470_0097 (
PULPROG zg30
TD 65536
SOLVENT CDCl3
NS 1
DS 0
SWH 8012.820 Hz
FIDRES 0.244532 Hz
AQ 4.0894465 sec
RG 116.65
DW 62.400 usec
DE 6.50 usec
TE 300.0 K
D1 1.00000000 sec
TD0 1
SFO1 500.1730010 MHz
NUC1 1H
P0 4.00 usec
P1 12.00 usec
PLW1 17.00000000 W

F2 - Processing parameters
SI 65536
SF 500.1700156 MHz
WDW EM
SSB 0
LB 0.30 Hz
GB 0
PC 1.00

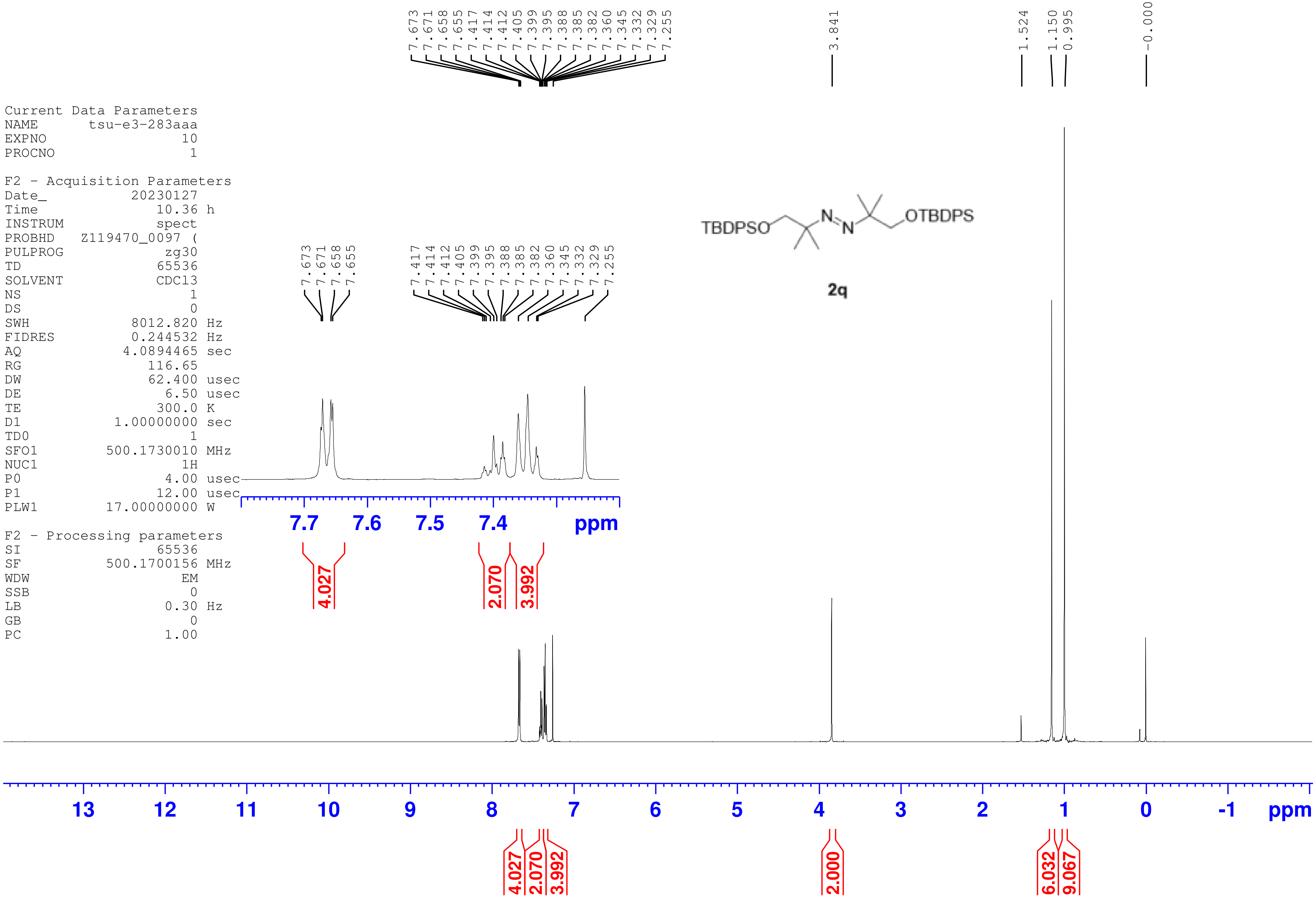

Current Data Parameters
NAME tsu-e3-283aaa
EXPNO 11
PROCNO 1

F2 - Acquisition Parameters
Date_ 20230127
Time 10.47 h
INSTRUM spect
PROBHD Z119470_0097 (
PULPROG zgpg30
TD 65536
SOLVENT CDCl3
NS 167
DS 0
SWH 29761.904 Hz
FIDRES 0.908261 Hz
AQ 1.1010048 sec
RG 189.66
DW 16.800 usec
DE 6.50 usec
TE 300.0 K
D1 1.89900005 sec
D11 0.03000000 sec
TD0 1
SFO1 125.7804228 MHz
NUC1 13C
P0 3.67 usec
P1 11.00 usec
PLW1 75.00000000 W
SFO2 500.1720007 MHz
NUC2 1H
CPDPRG[2 waltz16
PCPD2 90.00 usec
PLW2 17.00000000 W
PLW12 0.30221999 W
PLW13 0.17000000 W

F2 - Processing parameters
SI 32768
SF 125.7678470 MHz
WDW EM
SSB 0
LB 1.00 Hz
GB 0
PC 1.40

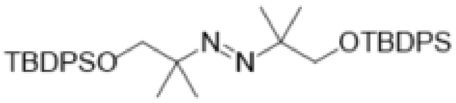

2q

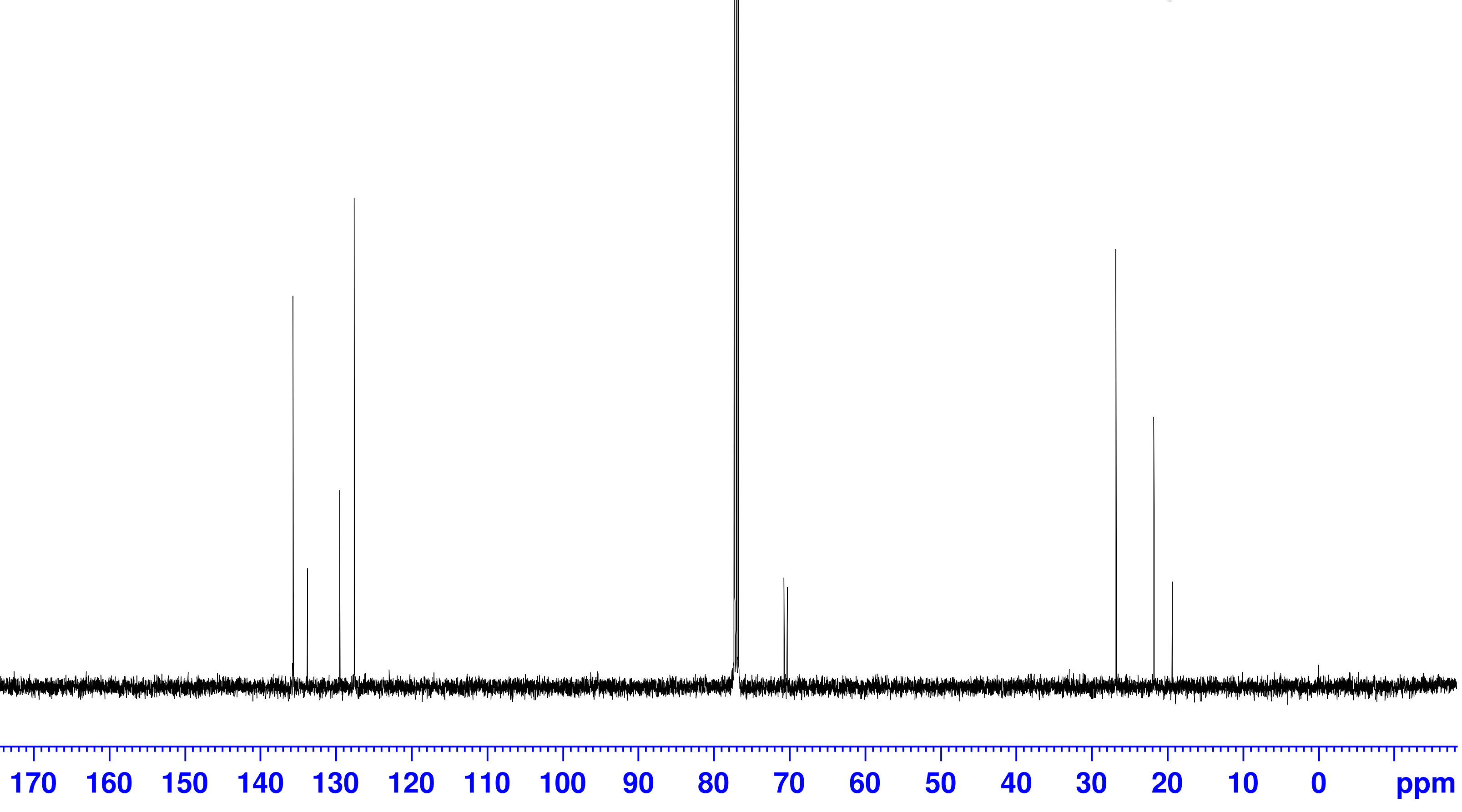

```
Current Data Parameters
NAME           tsu-e3-284a
EXPNO                   10
PROCNO                   1

F2 - Acquisition Parameters
Date_             20230126
Time                 14.53 h
INSTRUM              spect
PROBHD    Z119470_0097 (
PULPROG               zg30
TD                   65536
SOLVENT              CDCl3
NS                       1
DS                       0
SWH               8012.820 Hz
FIDRES            0.244532 Hz
AQ               4.0894465 sec
RG                  130.52
DW                  62.400 usec
DE                    6.50 usec
TE                   300.0 K
D1             1.00000000 sec
TD0                      1
SFO1           500.1730010 MHz
NUC1                    1H
P0                    4.00 usec
P1                   12.00 usec
PLW1           17.00000000 W

F2 - Processing parameters
SI                   65536
SF             500.1700139 MHz
WDW                     EM
SSB                      0
LB                    0.30 Hz
GB                       0
PC                    1.00
```

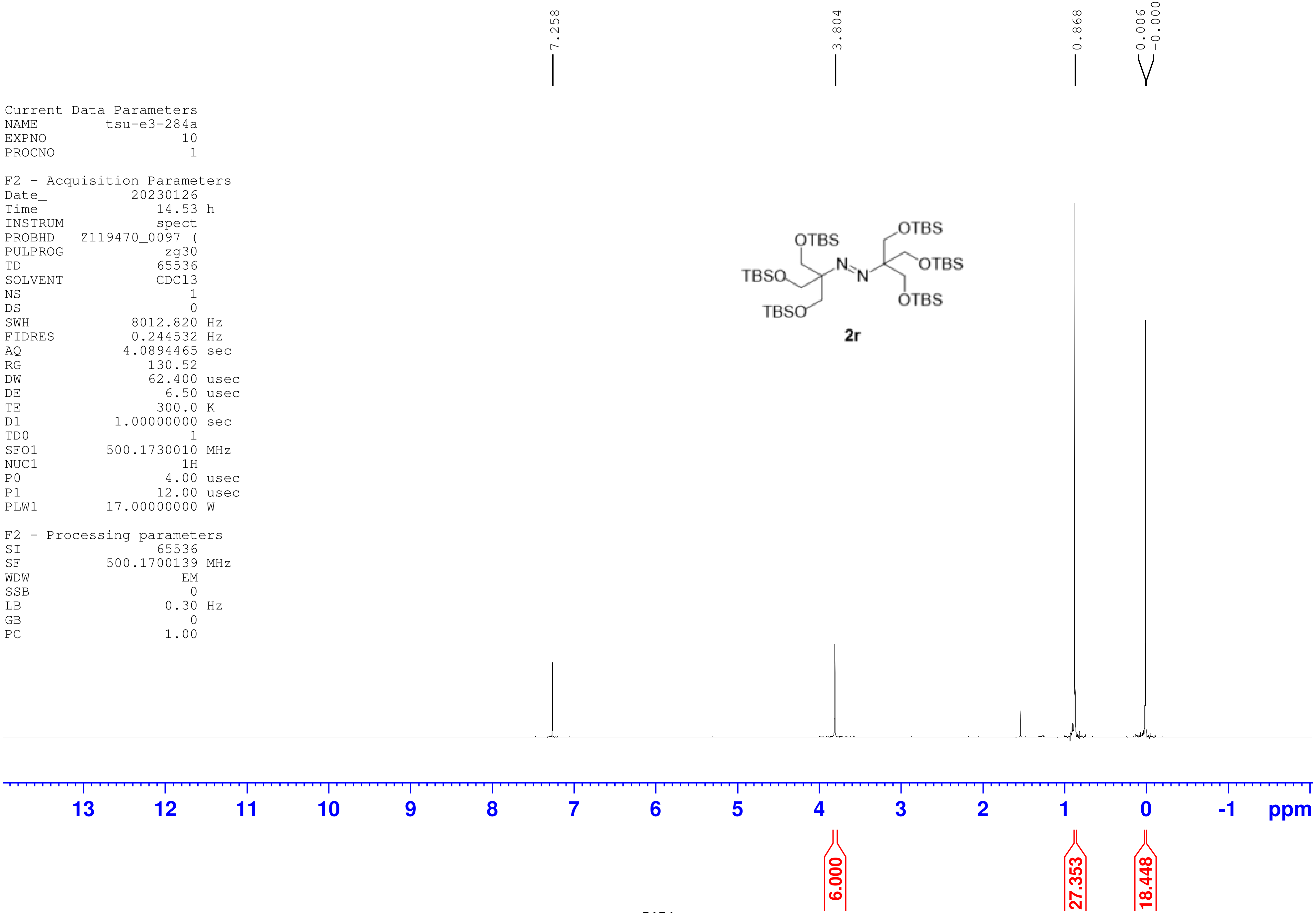

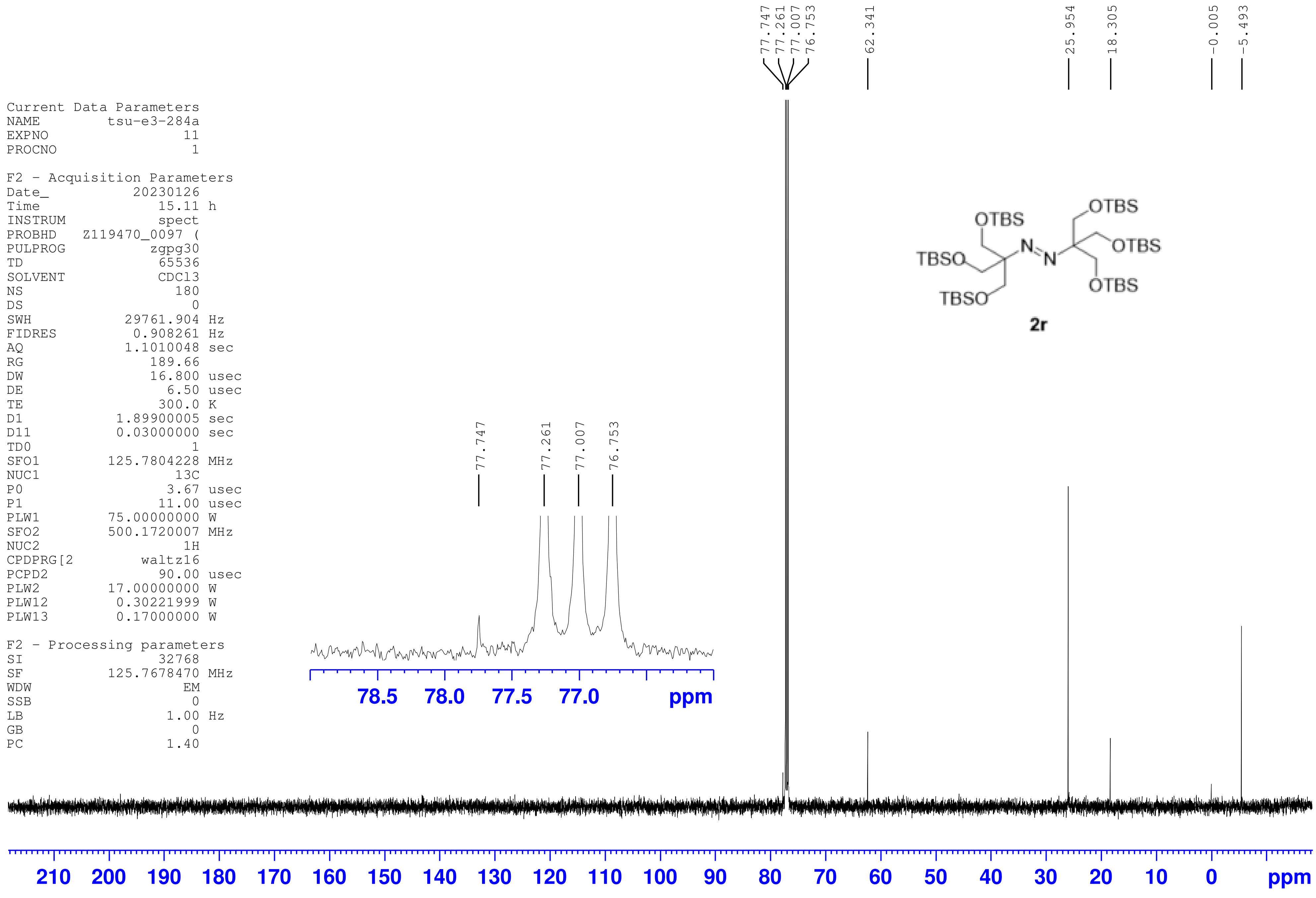
Current Data Parameters
NAME tsu-e3-284a
EXPNO 11
PROCNO 1
F2 - Acquisition Parameters
Date_ 20230126
Time 15.11 h
INSTRUM spect
PROBHD Z119470_0097 (
PULPROG zgpg30
TD 65536
SOLVENT CDCl3
NS 180
DS 0
SWH 29761.904 Hz
FIDRES 0.908261 Hz
AQ 1.1010048 sec
RG 189.66
DW 16.800 usec
DE 6.50 usec
TE 300.0 K
D1 1.89900005 sec
D11 0.03000000 sec
TD0 1
SFO1 125.7804228 MHz
NUC1 13C
P0 3.67 usec
P1 11.00 usec
PLW1 75.00000000 W
SFO2 500.1720007 MHz
NUC2 1H
CPDPRG[2 waltz16
PCPD2 90.00 usec
PLW2 17.00000000 W
PLW12 0.30221999 W
PLW13 0.17000000 W
F2 - Processing parameters
SI 32768
SF 125.7678470 MHz
WDW EM
SSB 0
LB 1.00 Hz
GB 0
PC 1.40
77.747
77.261
77.007
76.753
62.341
25.954
18.305
-0.005
-5.493
TBSO
TBSO
OTBS
N
N
OTBS
OTBS
OTBS
2r
78.5 78.0 77.5 77.0 ppm
210 200 190 180 170 160 150 140 130 120 110 100 90 80 70 60 50 40 30 20 10 0 ppm

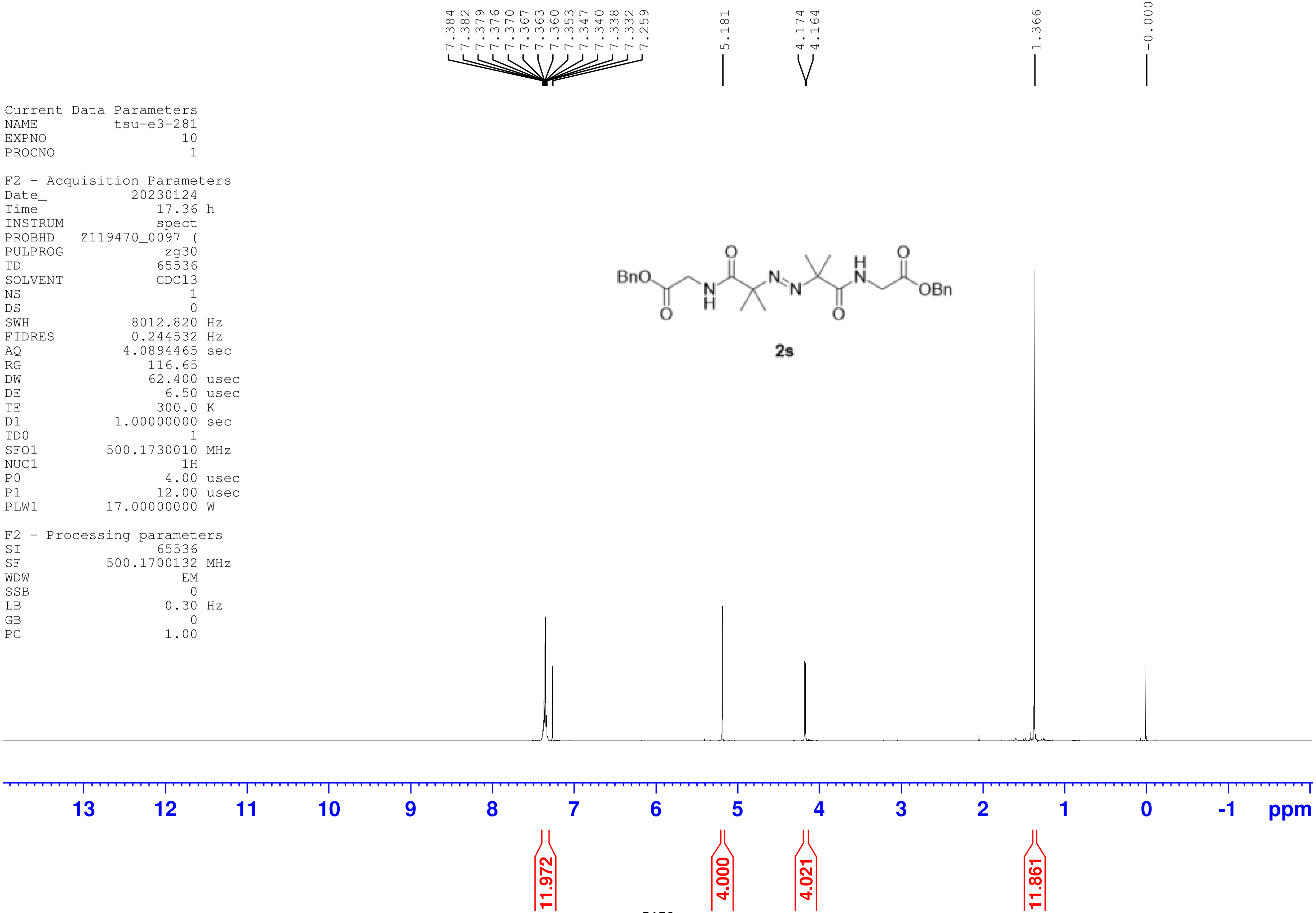
7.384
7.382
7.379
7.376
7.370
7.367
7.363
7.360
7.353
7.347
7.340
7.338
7.332
7.259
5.181
4.174
4.164
1.366
-0.000
Current Data Parameters
NAME tsu-e3-281
EXPNO 10
PROCNO 1
F2 - Acquisition Parameters
Date_ 20230124
Time 17.36 h
INSTRUM spect
PROBHD Z119470_0097 (
PULPROG zg30
TD 65536
SOLVENT CDCl3
NS 1
DS 0
SWH 8012.820 Hz
FIDRES 0.244532 Hz
AQ 4.0894465 sec
RG 116.65
DW 62.400 usec
DE 6.50 usec
TE 300.0 K
D1 1.00000000 sec
TD0 1
SFO1 500.1730010 MHz
NUC1 1H
P0 4.00 usec
P1 12.00 usec
PLW1 17.00000000 W
F2 - Processing parameters
SI 65536
SF 500.1700132 MHz
WDW EM
SSB 0
LB 0.30 Hz
GB 0
PC 1.00
13
12
11
10
9
8
7
6
5
4
3
2
1
0
-1
ppm
11.972
4.000
4.021
11.861
BnO
O
N
H
O
N
N
H
N
O
O
OBn

2s

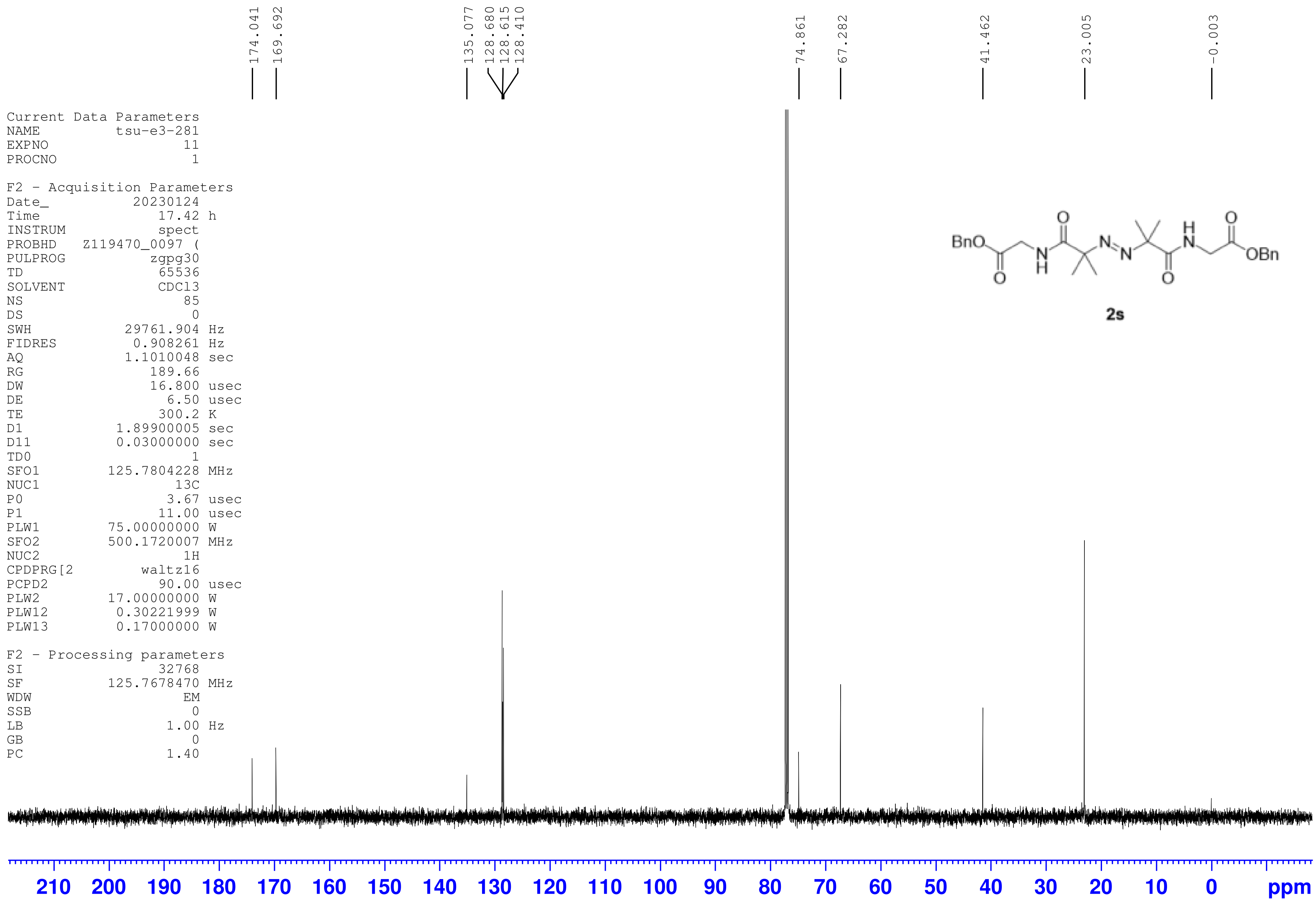
174.041
169.692
135.077
128.680
128.615
128.410
74.861
67.282
41.462
23.005
-0.003
Current Data Parameters
NAME tsu-e3-281
EXPNO 11
PROCNO 1
F2 - Acquisition Parameters
Date_ 20230124
Time 17.42 h
INSTRUM spect
PROBHD Z119470_0097 (
PULPROG zgpg30
TD 65536
SOLVENT CDCl3
NS 85
DS 0
SWH 29761.904 Hz
FIDRES 0.908261 Hz
AQ 1.1010048 sec
RG 189.66
DW 16.800 usec
DE 6.50 usec
TE 300.2 K
D1 1.89900005 sec
D11 0.03000000 sec
TD0 1
SFO1 125.7804228 MHz
NUC1 13C
P0 3.67 usec
P1 11.00 usec
PLW1 75.00000000 W
SFO2 500.1720007 MHz
NUC2 1H
CPDPRG[2 waltz16
PCPD2 90.00 usec
PLW2 17.00000000 W
PLW12 0.30221999 W
PLW13 0.17000000 W
F2 - Processing parameters
SI 32768
SF 125.7678470 MHz
WDW EM
SSB 0
LB 1.00 Hz
GB 0
PC 1.40
210 200 190 180 170 160 150 140 130 120 110 100 90 80 70 60 50 40 30 20 10 0 ppm
BnO
O
N
H
O
N
N
H
N
O
O
OBn

2s

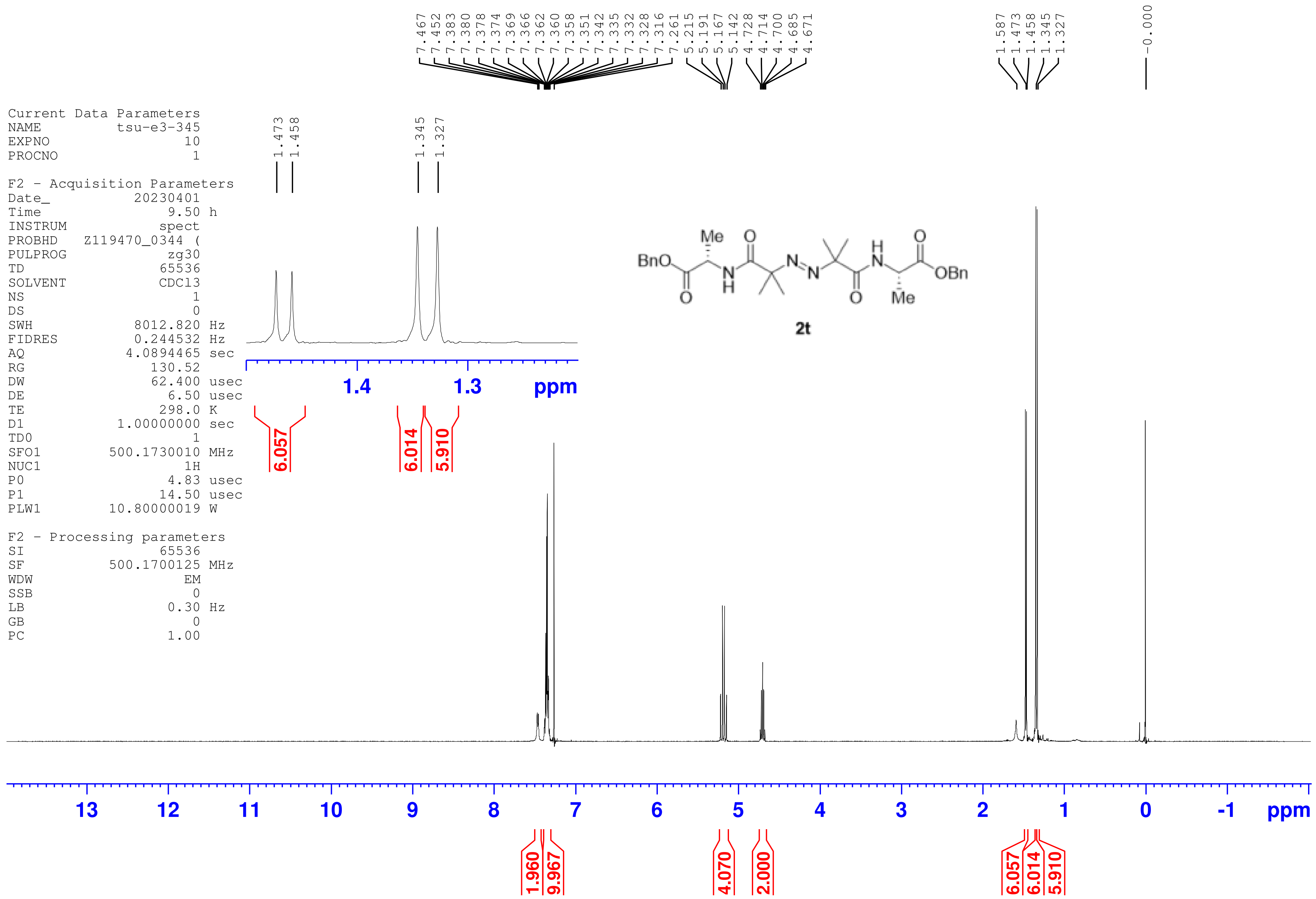
Current Data Parameters
NAME tsu-e3-345
EXPNO 10
PROCNO 1
F2 - Acquisition Parameters
Date_ 20230401
Time 9.50 h
INSTRUM spect
PROBHD Z119470_0344 (
PULPROG zg30
TD 65536
SOLVENT CDCl3
NS 1
DS 0
SWH 8012.820 Hz
FIDRES 0.244532 Hz
AQ 4.0894465 sec
RG 130.52
DW 62.400 usec
DE 6.50 usec
TE 298.0 K
D1 1.00000000 sec
TD0 1
SFO1 500.1730010 MHz
NUC1 1H
P0 4.83 usec
P1 14.50 usec
PLW1 10.80000019 W
F2 - Processing parameters
SI 65536
SF 500.1700125 MHz
WDW EM
SSB 0
LB 0.30 Hz
GB 0
PC 1.00
7.467 7.452 7.383 7.380 7.378 7.374 7.369 7.366 7.362 7.360 7.358 7.351 7.342 7.335 7.332 7.328 7.316 7.261 5.215 5.191 5.167 5.142 4.728 4.714 4.700 4.685 4.671
1.587 1.473 1.458 1.345 1.327
-0.000
1.473 1.458 1.345 1.327
1.4 1.3 ppm
6.057 6.014 5.910
BnO Me O N H N N H N O O Me OBn
2t
13 12 11 10 9 8 7 6 5 4 3 2 1 0 -1 ppm
1.960 9.967 4.070 2.000 6.057 6.014 5.910

Current Data Parameters
NAME tsu-e3-345
EXPNO 11
PROCNO 1

F2 - Acquisition Parameters
Date_ 20230401
Time 9.57 h
INSTRUM spect
PROBHD Z119470_0344 (
PULPROG zgpg30
TD 65536
SOLVENT CDCl3
NS 108
DS 0
SWH 29761.904 Hz
FIDRES 0.908261 Hz
AQ 1.1010048 sec
RG 189.66
DW 16.800 usec
DE 6.50 usec
TE 298.0 K
D1 1.89900005 sec
D11 0.03000000 sec
TD0 1
SFO1 125.7804228 MHz
NUC1 13C
P0 3.67 usec
P1 11.00 usec
PLW1 69.64499664 W
SFO2 500.1720007 MHz
NUC2 1H
CPDPRG[2 waltz16
PCPD2 90.00 usec
PLW2 10.80000019 W
PLW12 0.28033000 W
PLW13 0.15769000 W

F2 - Processing parameters
SI 32768
SF 125.7678470 MHz
WDW EM
SSB 0
LB 1.00 Hz
GB 0
PC 1.40

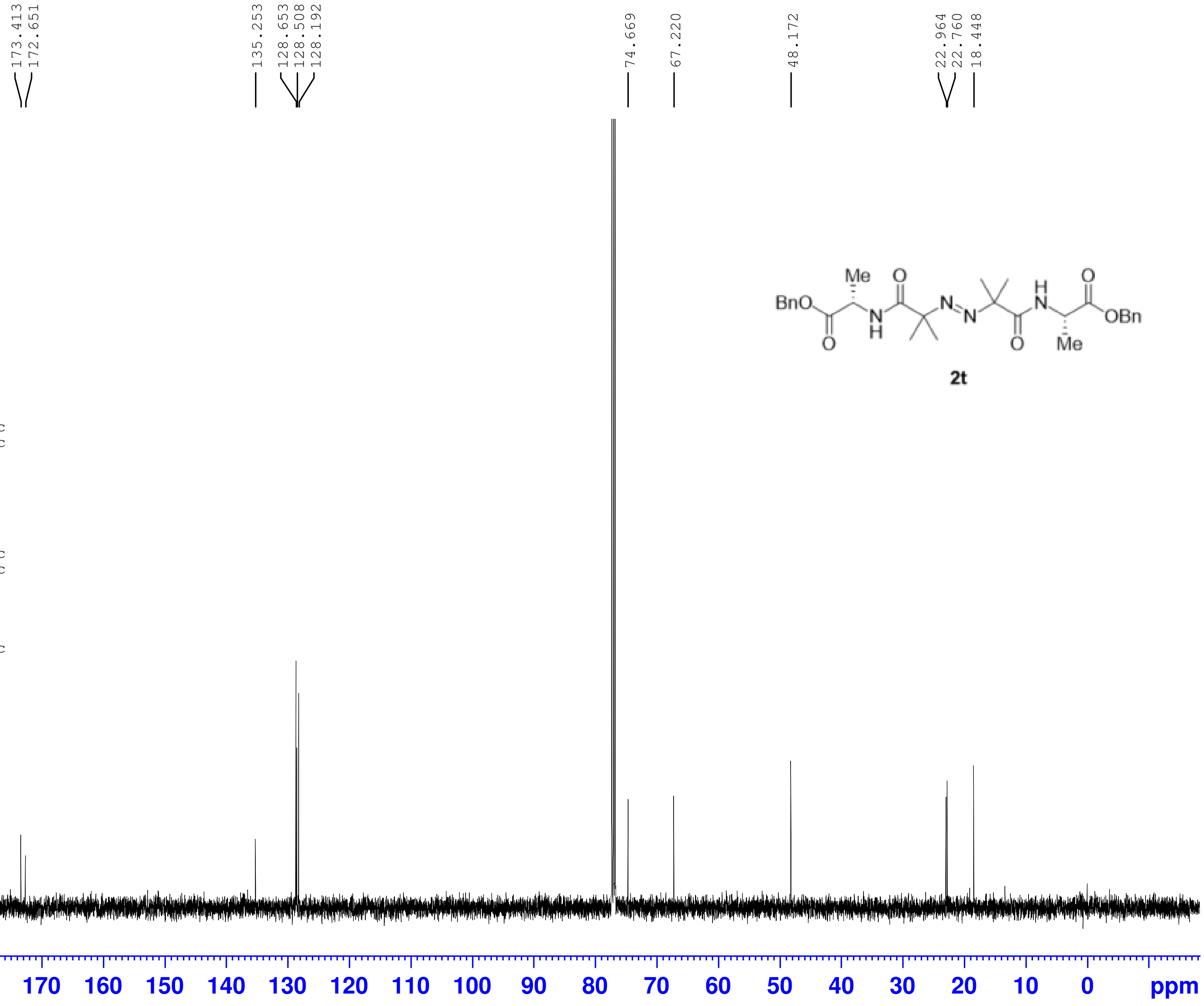

2t

```
Current Data Parameters
NAME          tsu-e3-354
EXPNO                 10
PROCNO                 1

F2 - Acquisition Parameters
Date_           20230405
Time               12.22 h
INSTRUM            spect
PROBHD   Z119470_0344 (
PULPROG             zg30
TD                 65536
SOLVENT            CDCl3
NS                     1
DS                     0
SWH             8012.820 Hz
FIDRES          0.244532 Hz
AQ             4.0894465 sec
RG                116.65
DW                62.400 usec
DE                  6.50 usec
TE                 298.0 K
D1            1.00000000 sec
TD0                    1
SFO1         500.1730010 MHz
NUC1                  1H
P0                  4.83 usec
P1                 14.50 usec
PLW1         10.80000019 W

F2 - Processing parameters
SI                 65536
SF           500.1700132 MHz
WDW                   EM
SSB                    0
LB                  0.30 Hz
GB                     0
PC                  1.00
```

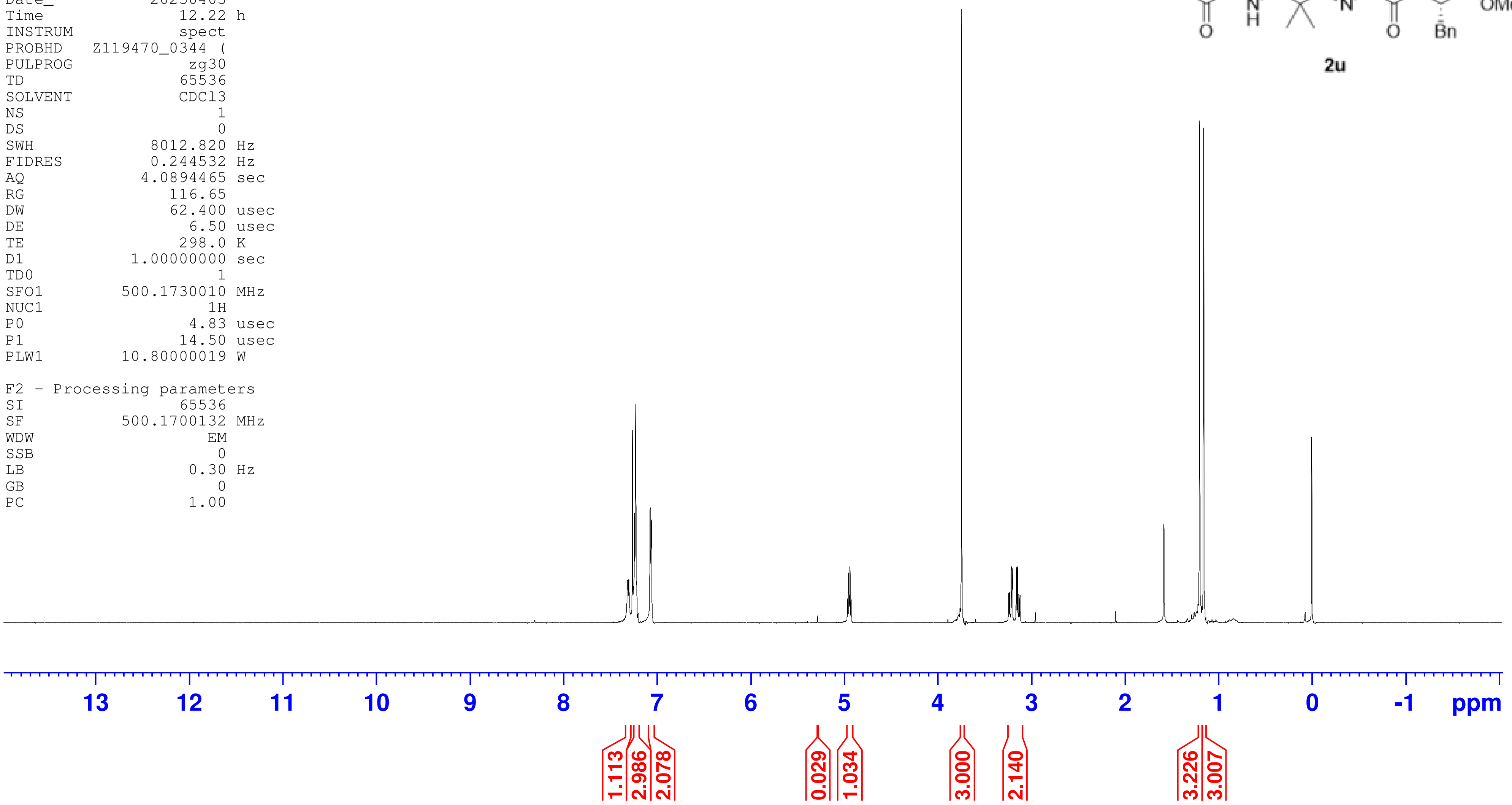

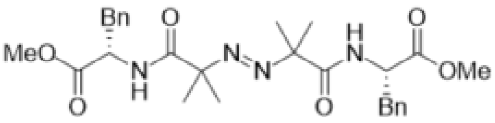

2u

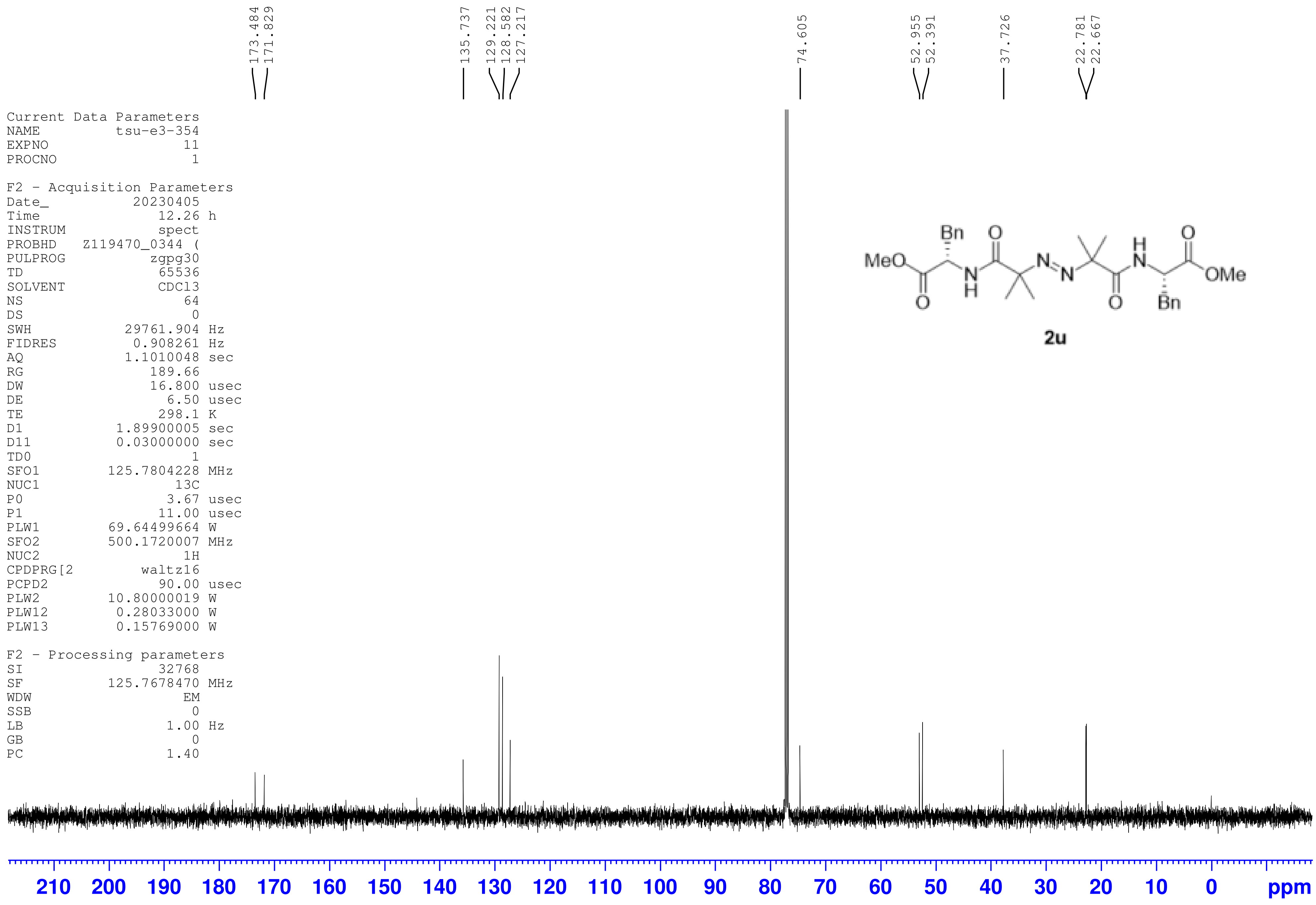
173.484
171.829
135.737
129.221
128.582
127.217
74.605
52.955
52.391
37.726
22.781
22.667
Current Data Parameters
NAME tsu-e3-354
EXPNO 11
PROCNO 1
F2 - Acquisition Parameters
Date_ 20230405
Time 12.26 h
INSTRUM spect
PROBHD Z119470_0344 (
PULPROG zgpg30
TD 65536
SOLVENT CDCl3
NS 64
DS 0
SWH 29761.904 Hz
FIDRES 0.908261 Hz
AQ 1.1010048 sec
RG 189.66
DW 16.800 usec
DE 6.50 usec
TE 298.1 K
D1 1.89900005 sec
D11 0.03000000 sec
TD0 1
SFO1 125.7804228 MHz
NUC1 13C
P0 3.67 usec
P1 11.00 usec
PLW1 69.64499664 W
SFO2 500.1720007 MHz
NUC2 1H
CPDPRG[2 waltz16
PCPD2 90.00 usec
PLW2 10.80000019 W
PLW12 0.28033000 W
PLW13 0.15769000 W
F2 - Processing parameters
SI 32768
SF 125.7678470 MHz
WDW EM
SSB 0
LB 1.00 Hz
GB 0
PC 1.40
210 200 190 180 170 160 150 140 130 120 110 100 90 80 70 60 50 40 30 20 10 0 ppm
MeO
Bn
O
N
H
N
N
O
H
N
O
OMe
O
Bn

2u

Current Data Parameters
NAME tsu-e3-479
EXPNO 10
PROCNO 1

F2 - Acquisition Parameters
Date_ 20230629
Time 17.15 h
INSTRUM spect
PROBHD Z119470_0344 (
PULPROG zg30
TD 65536
SOLVENT CDCl3
NS 1
DS 0
SWH 8012.820 Hz
FIDRES 0.244532 Hz
AQ 4.0894465 sec
RG 107.18
DW 62.400 usec
DE 6.50 usec
TE 298.0 K
D1 1.00000000 sec
TD0 1
SFO1 500.1730010 MHz
NUC1 1H
P0 4.83 usec
P1 14.50 usec
PLW1 10.80000019 W

F2 - Processing parameters
SI 65536
SF 500.1700119 MHz
WDW EM
SSB 0
LB 0.30 Hz
GB 0
PC 1.00

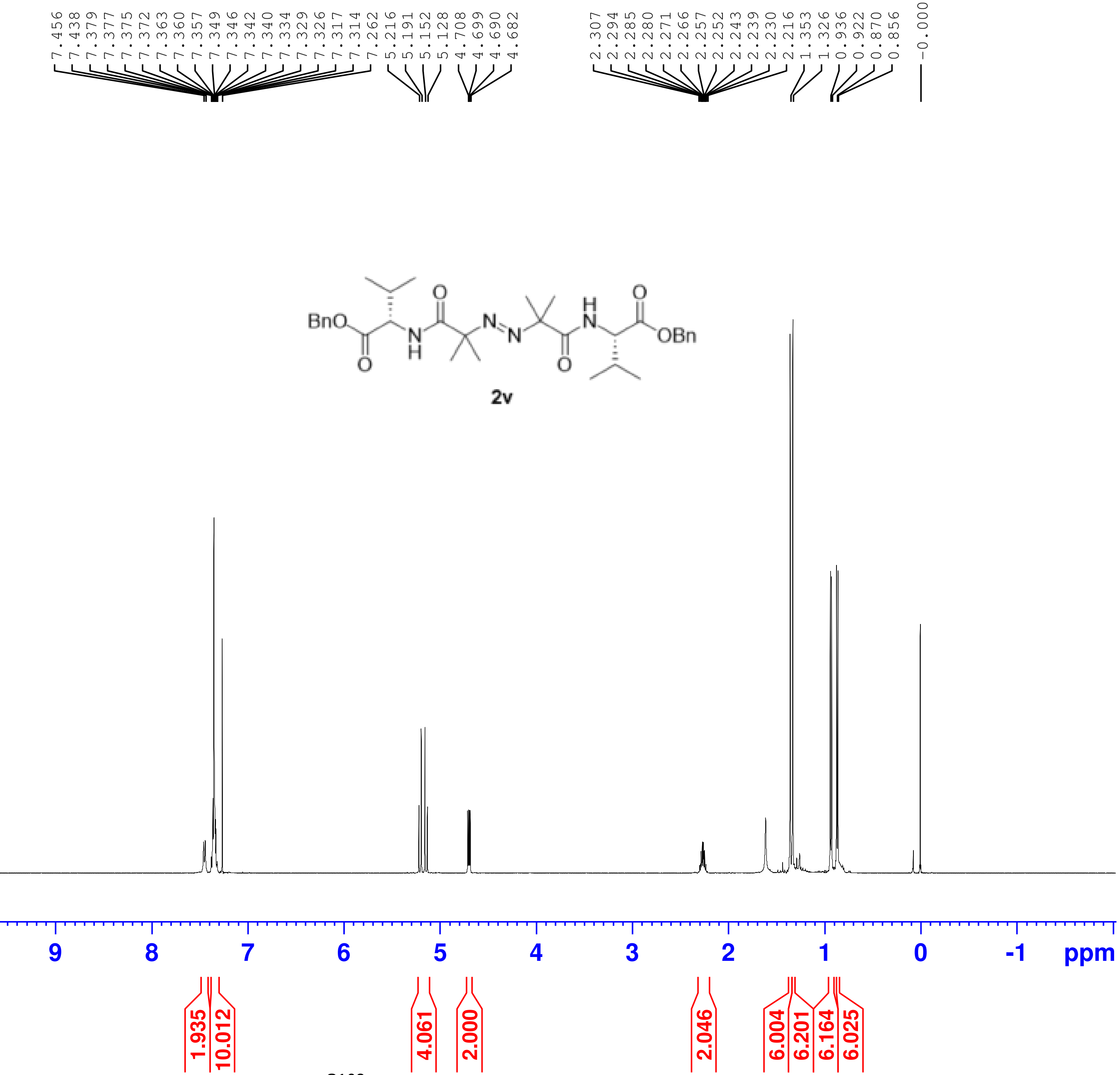

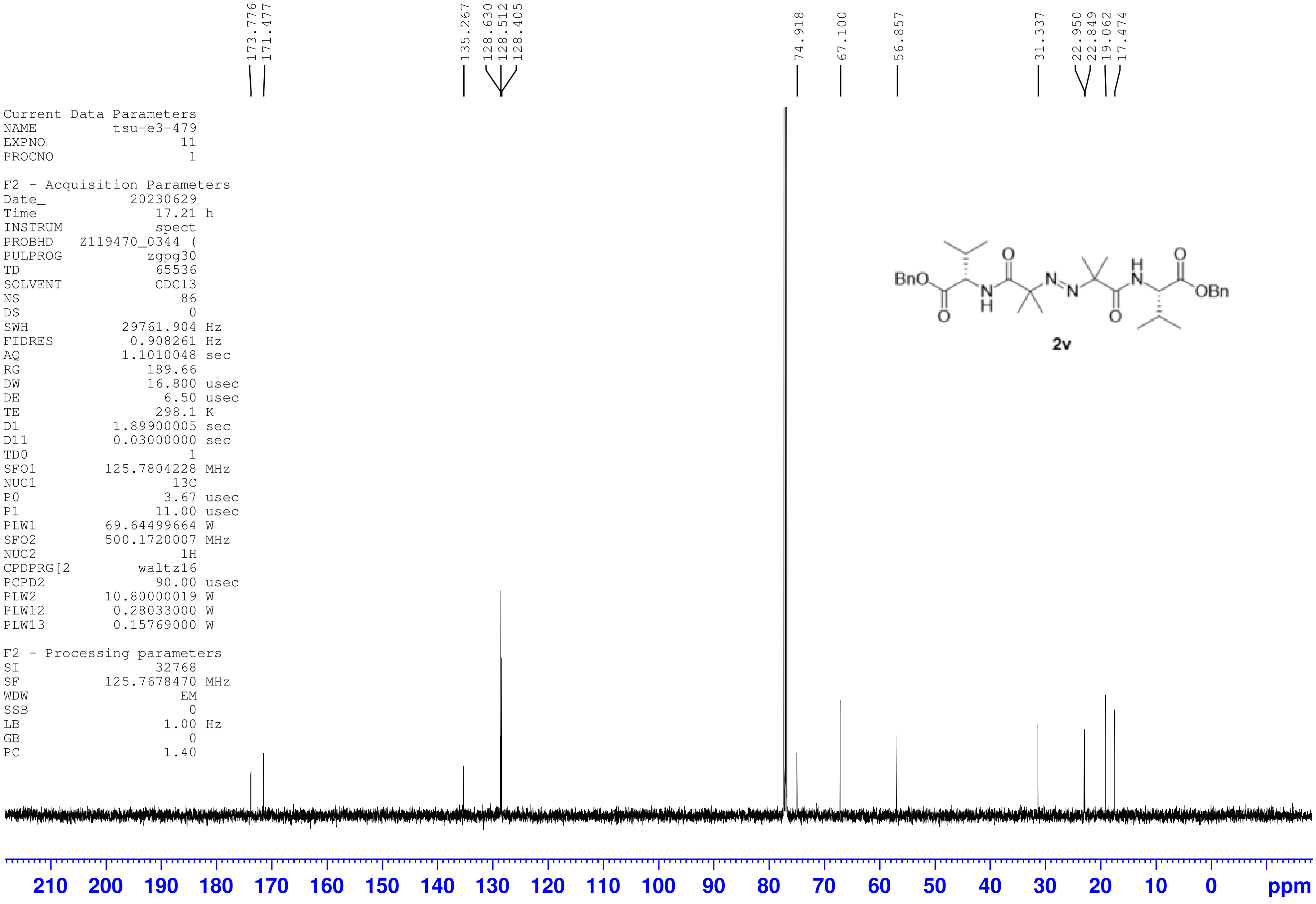
173.776
171.477
135.267
128.630
128.512
128.405
74.918
67.100
56.857
31.337
22.950
22.849
19.062
17.474
Current Data Parameters
NAME tsu-e3-479
EXPNO 11
PROCNO 1
F2 - Acquisition Parameters
Date_ 20230629
Time 17.21 h
INSTRUM spect
PROBHD Z119470_0344 (
PULPROG zgpg30
TD 65536
SOLVENT CDCl3
NS 86
DS 0
SWH 29761.904 Hz
FIDRES 0.908261 Hz
AQ 1.1010048 sec
RG 189.66
DW 16.800 usec
DE 6.50 usec
TE 298.1 K
D1 1.89900005 sec
D11 0.03000000 sec
TD0 1
SFO1 125.7804228 MHz
NUC1 13C
P0 3.67 usec
P1 11.00 usec
PLW1 69.64499664 W
SFO2 500.1720007 MHz
NUC2 1H
CPDPRG[2 waltz16
PCPD2 90.00 usec
PLW2 10.80000019 W
PLW12 0.28033000 W
PLW13 0.15769000 W
F2 - Processing parameters
SI 32768
SF 125.7678470 MHz
WDW EM
SSB 0
LB 1.00 Hz
GB 0
PC 1.40
BnO
OBn
2v
210 200 190 180 170 160 150 140 130 120 110 100 90 80 70 60 50 40 30 20 10 0 ppm

Current Data Parameters
NAME tsu-e3-585b
EXPNO 30
PROCNO 1

F2 - Acquisition Parameters
Date_ 20231016
Time 9.09 h
INSTRUM spect
PROBHD Z130033_0007 (
PULPROG zg30
TD 65536
SOLVENT CDCl3
NS 1
DS 0
SWH 8012.820 Hz
FIDRES 0.244532 Hz
AQ 4.0894465 sec
RG 31.29
DW 62.400 usec
DE 10.00 usec
TE 300.1 K
D1 1.00000000 sec
TD0 1
SFO1 500.1730010 MHz
NUC1 1H
P0 4.00 usec
P1 12.00 usec
PLW1 16.00000000 W

F2 - Processing parameters
SI 65536
SF 500.1700128 MHz
WDW EM
SSB 0
LB 0.30 Hz
GB 0
PC 1.00

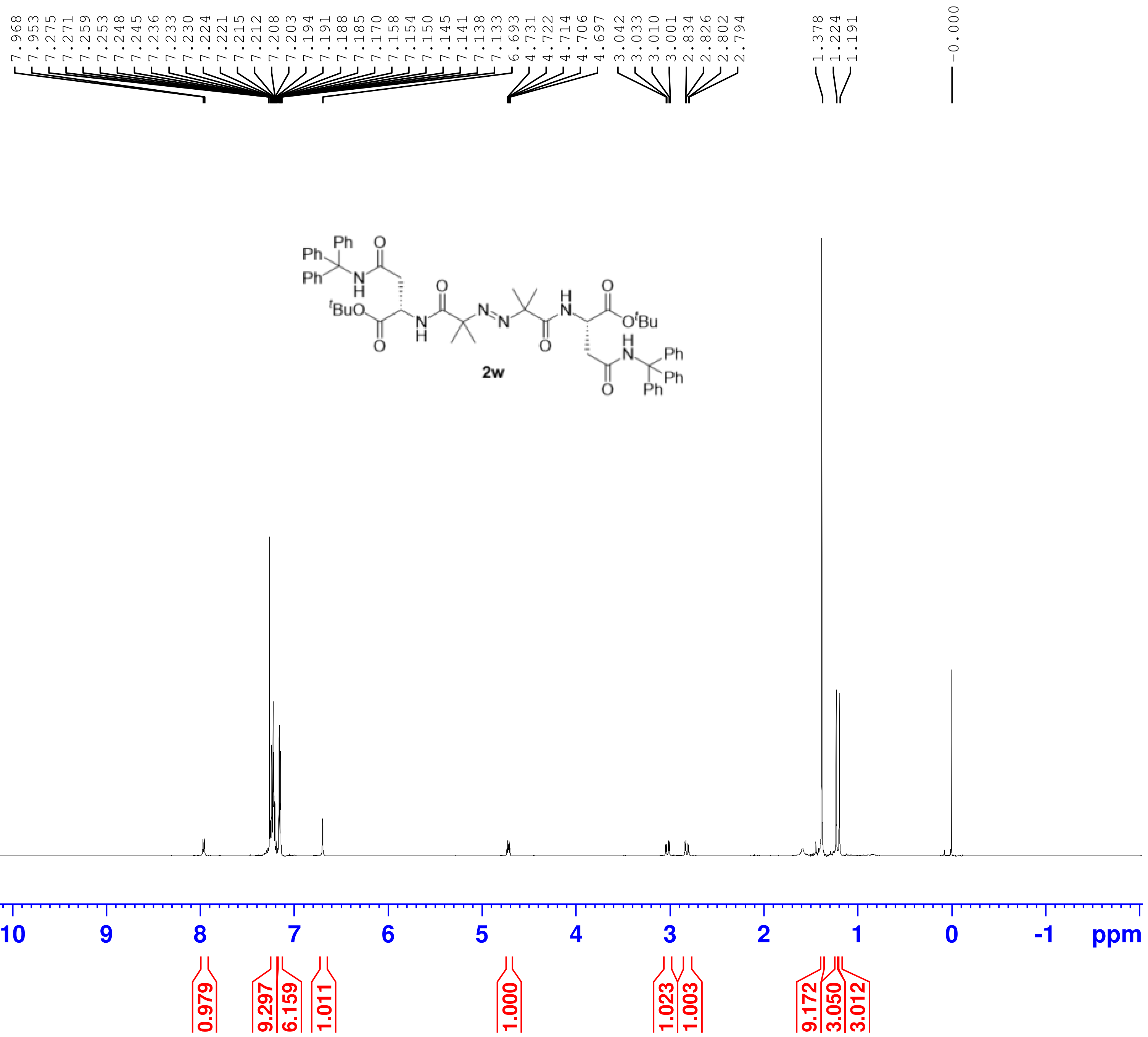

2w

Current Data Parameters
NAME tsu-e3-585b
EXPNO 31
PROCNO 1

F2 - Acquisition Parameters
Date_ 20231016
Time 9.20 h
INSTRUM spect
PROBHD Z130033_0007 (
PULPROG zgpg30
TD 65536
SOLVENT CDCl3
NS 186
DS 0
SWH 29761.904 Hz
FIDRES 0.908261 Hz
AQ 1.1010048 sec
RG 189.66
DW 16.800 usec
DE 11.00 usec
TE 300.0 K
D1 1.89900005 sec
D11 0.03000000 sec
TD0 1
SFO1 125.7804223 MHz
NUC1 13C
P0 3.33 usec
P1 10.00 usec
PLW1 70.00000000 W
SFO2 500.1720007 MHz
NUC2 1H
CPDPRG[2 waltz16
PCPD2 80.00 usec
PLW2 16.00000000 W
PLW12 0.36000001 W
PLW13 0.18108000 W

F2 - Processing parameters
SI 32768
SF 125.7678475 MHz
WDW EM
SSB 0
LB 1.00 Hz
GB 0
PC 1.40

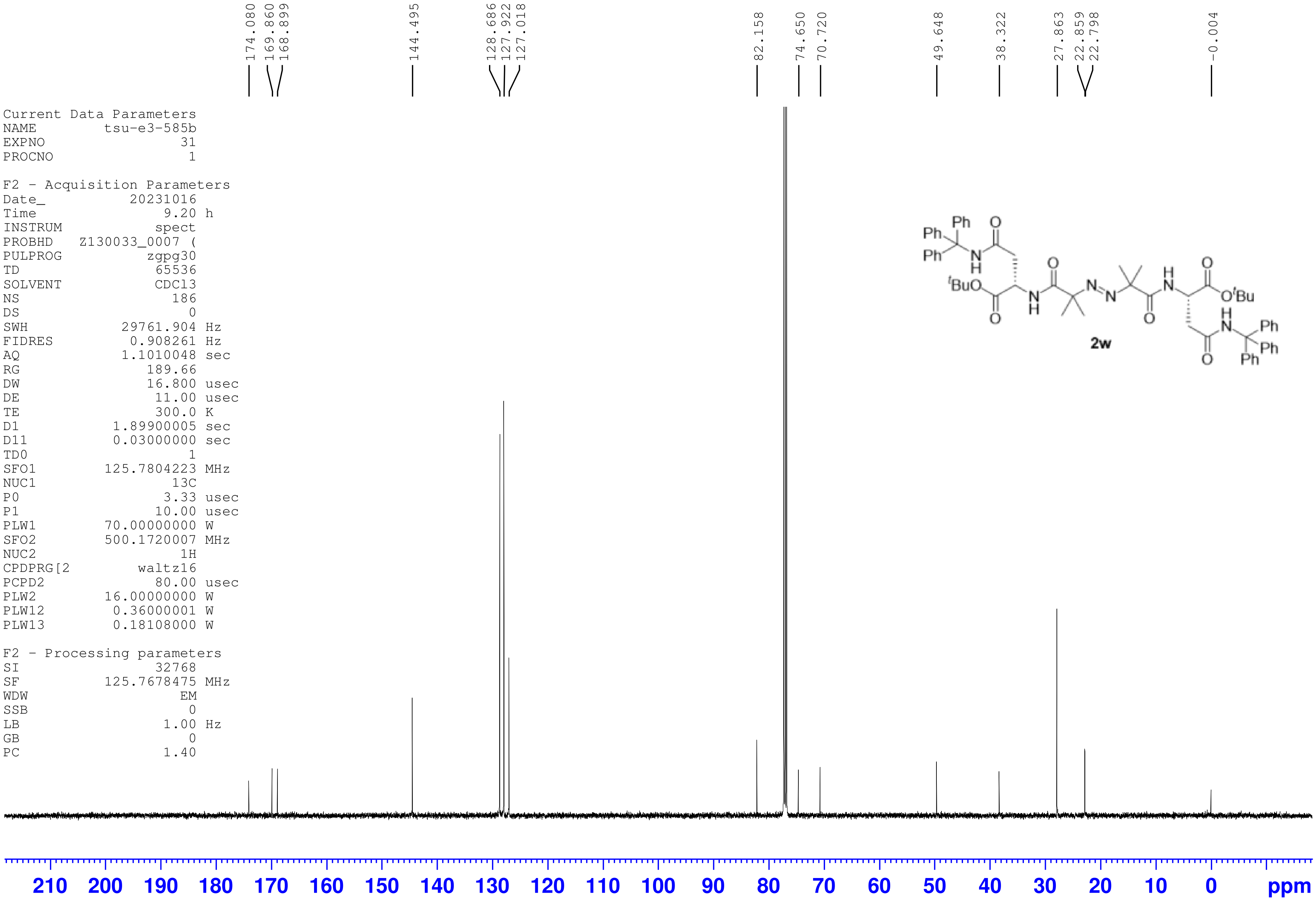

2w

```
Current Data Parameters
NAME      TKR-E-01-019_culumn_AA
EXPNO                 10
PROCNO                 1

F2 - Acquisition Parameters
Date_           20240626
Time               13.32 h
INSTRUM            spect
PROBHD    Z130033_0007 (
PULPROG             zg30
TD                 65536
SOLVENT            CDCl3
NS                     1
DS                     0
SWH             8012.820 Hz
FIDRES          0.244532 Hz
AQ             4.0894465 sec
RG                 31.29
DW                62.400 usec
DE                 10.00 usec
TE                 300.1 K
D1            1.00000000 sec
TD0                    1
SFO1         500.1730010 MHz
NUC1                  1H
P0                  4.00 usec
P1                 12.00 usec
PLW1         16.00000000 W

F2 - Processing parameters
SI                 65536
SF           500.1700115 MHz
WDW                   EM
SSB                    0
LB                  0.30 Hz
GB                     0
PC                  1.00
```

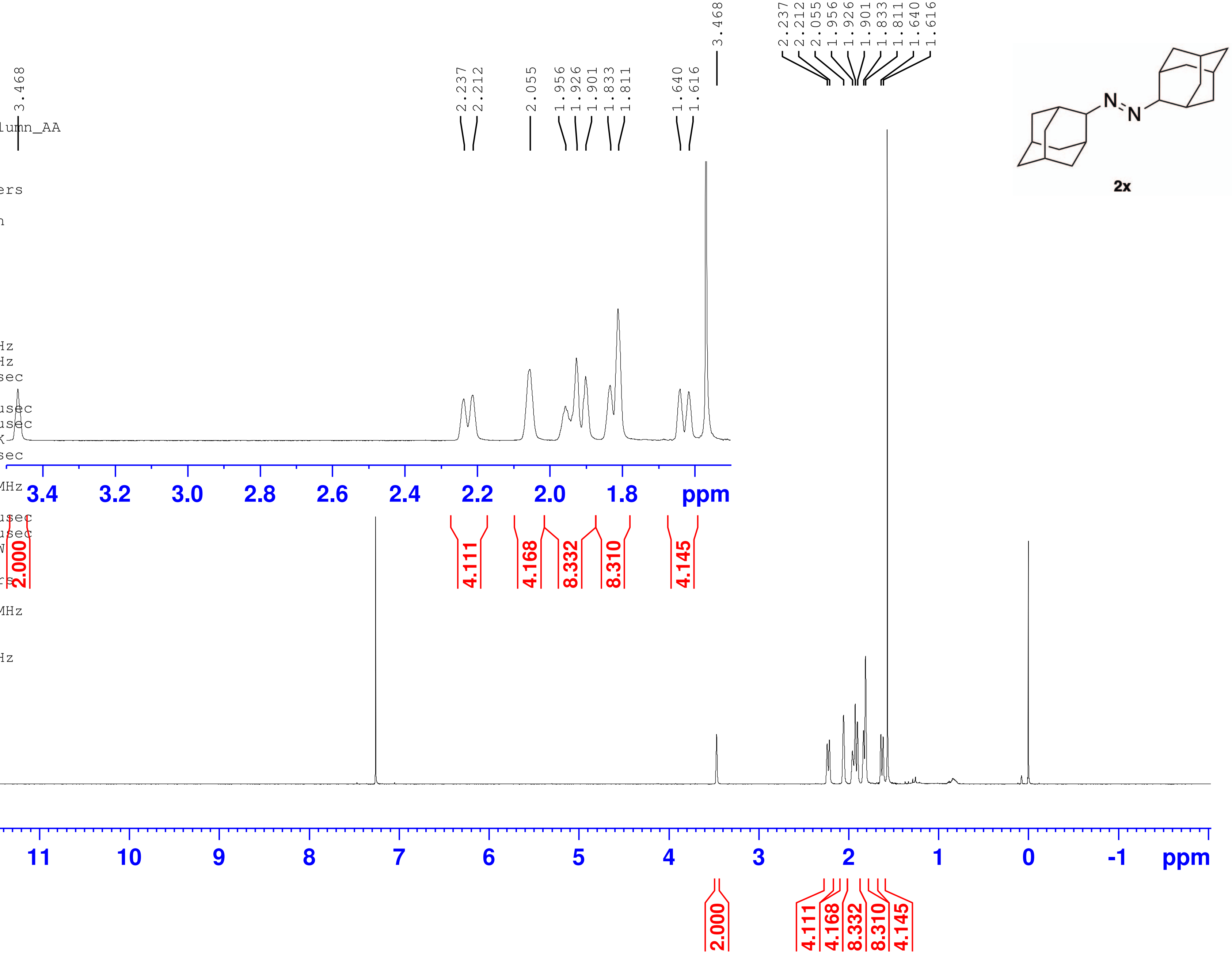

2x

```
Current Data Parameters
NAME      TKR-E-01-019_culumn_AA
EXPNO                   11
PROCNO                   1

F2 - Acquisition Parameters
Date_          20240626
Time              13.40 h
INSTRUM           spect
PROBHD   Z130033_0007 (
PULPROG          zgpg30
TD                65536
SOLVENT           CDCl3
NS                  128
DS                    0
SWH           29761.904 Hz
FIDRES         0.908261 Hz
AQ            1.1010048 sec
RG               189.66
DW               16.800 usec
DE                11.00 usec
TE                300.0 K
D1           1.89900005 sec
D11          0.03000000 sec
TD0                   1
SFO1        125.7804223 MHz
NUC1                13C
P0                 3.33 usec
P1                10.00 usec
PLW1        70.00000000 W
SFO2        500.1720007 MHz
NUC2                 1H
CPDPRG[2        waltz16
PCPD2             80.00 usec
PLW2        16.00000000 W
PLW12        0.36000001 W
PLW13        0.18108000 W

F2 - Processing parameters
SI                32768
SF          125.7678466 MHz
WDW                  EM
SSB                   0
LB                 1.00 Hz
GB                    0
PC                 1.40
```

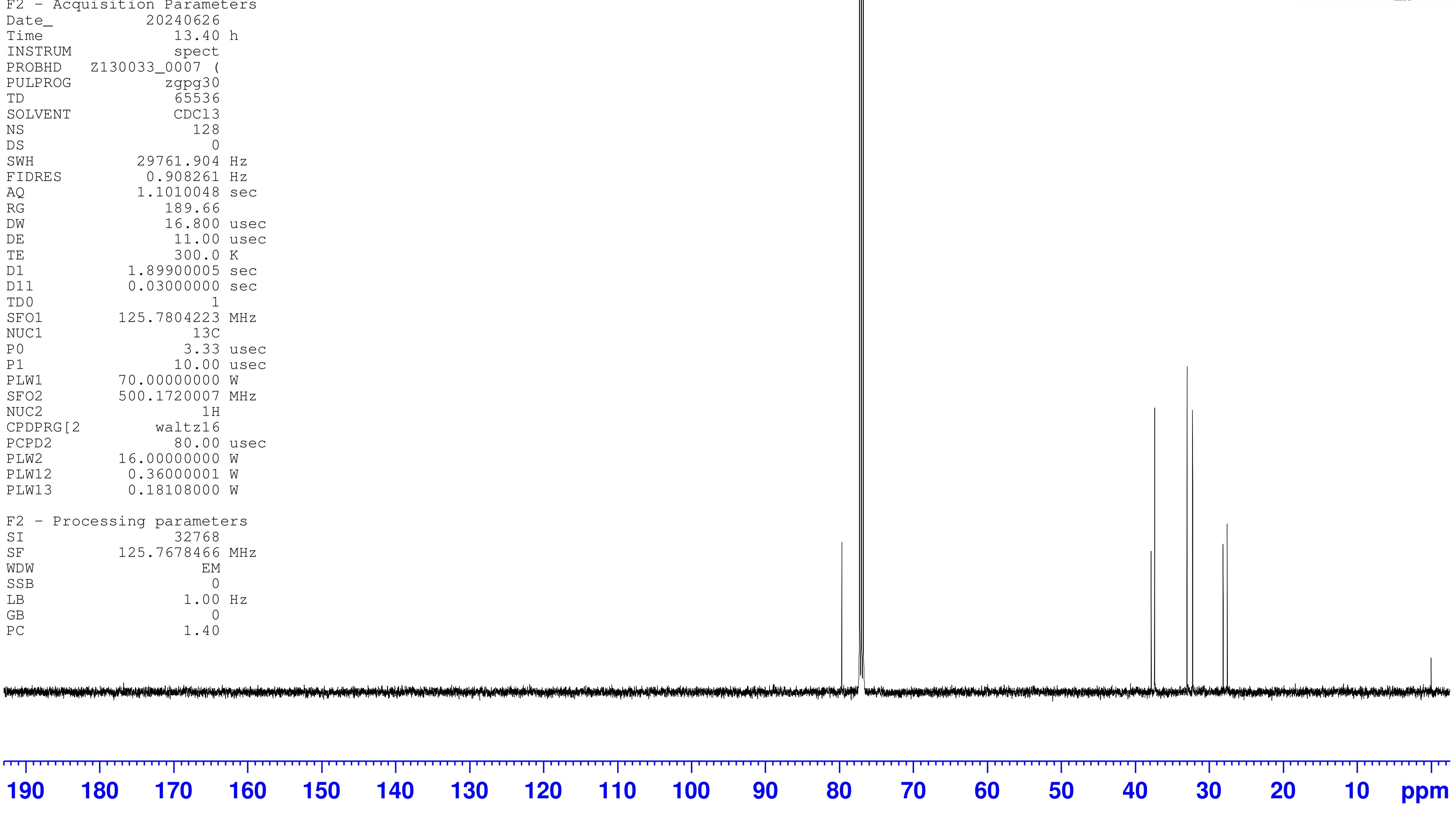

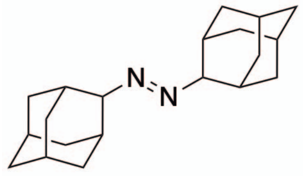

2x

```
Current Data Parameters
NAME       TKR-ER-01-020_culumn_A
EXPNO                        10
PROCNO                        1

F2 - Acquisition Parameters
Date_                  20240629
Time                     9.30 h
INSTRUM                   spect
PROBHD     Z130033_0007 (
PULPROG                    zg30
TD                        65536
SOLVENT                   CDCl3
NS                            1
DS                            0
SWH                    8012.820 Hz
FIDRES                 0.244532 Hz
AQ                    4.0894465 sec
RG                        31.29
DW                       62.400 usec
DE                        10.00 usec
TE                        300.0 K
D1                   1.00000000 sec
TD0                           1
SFO1                500.1730010 MHz
NUC1                         1H
P0                         4.00 usec
P1                        12.00 usec
PLW1                16.00000000 W

F2 - Processing parameters
SI                        65536
SF                  500.1700093 MHz
WDW                          EM
SSB                           0
LB                         0.30 Hz
GB                            0
PC                         1.00
```

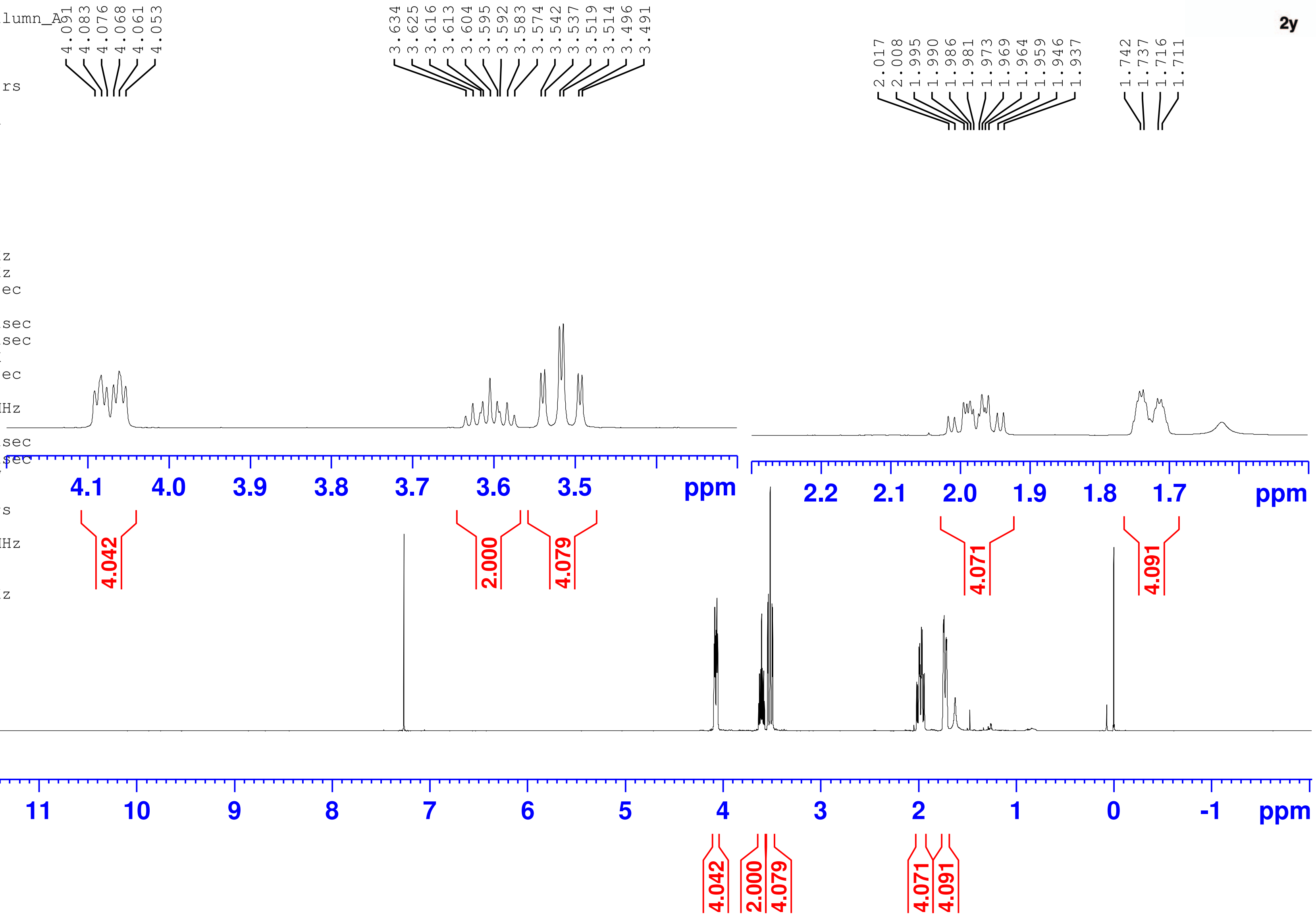

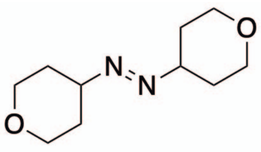

2y

```
Current Data Parameters
NAME     TKR-ER-01-020_culumn_A
EXPNO                 11
PROCNO                 1

F2 - Acquisition Parameters
Date_          20240629
Time               9.38 h
INSTRUM           spect
PROBHD   Z130033_0007 (
PULPROG          zgpg30
TD                65536
SOLVENT           CDCl3
NS                  128
DS                    0
SWH           29761.904 Hz
FIDRES         0.908261 Hz
AQ            1.1010048 sec
RG               189.66
DW               16.800 usec
DE                11.00 usec
TE                300.0 K
D1           1.89900005 sec
D11          0.03000000 sec
TD0                   1
SFO1        125.7804223 MHz
NUC1                13C
P0                 3.33 usec
P1                10.00 usec
PLW1        70.00000000 W
SFO2        500.1720007 MHz
NUC2                 1H
CPDPRG[2        waltz16
PCPD2             80.00 usec
PLW2        16.00000000 W
PLW12        0.36000001 W
PLW13        0.18108000 W

F2 - Processing parameters
SI                32768
SF          125.7678470 MHz
WDW                  EM
SSB                   0
LB                 1.00 Hz
GB                    0
PC                 1.40
```

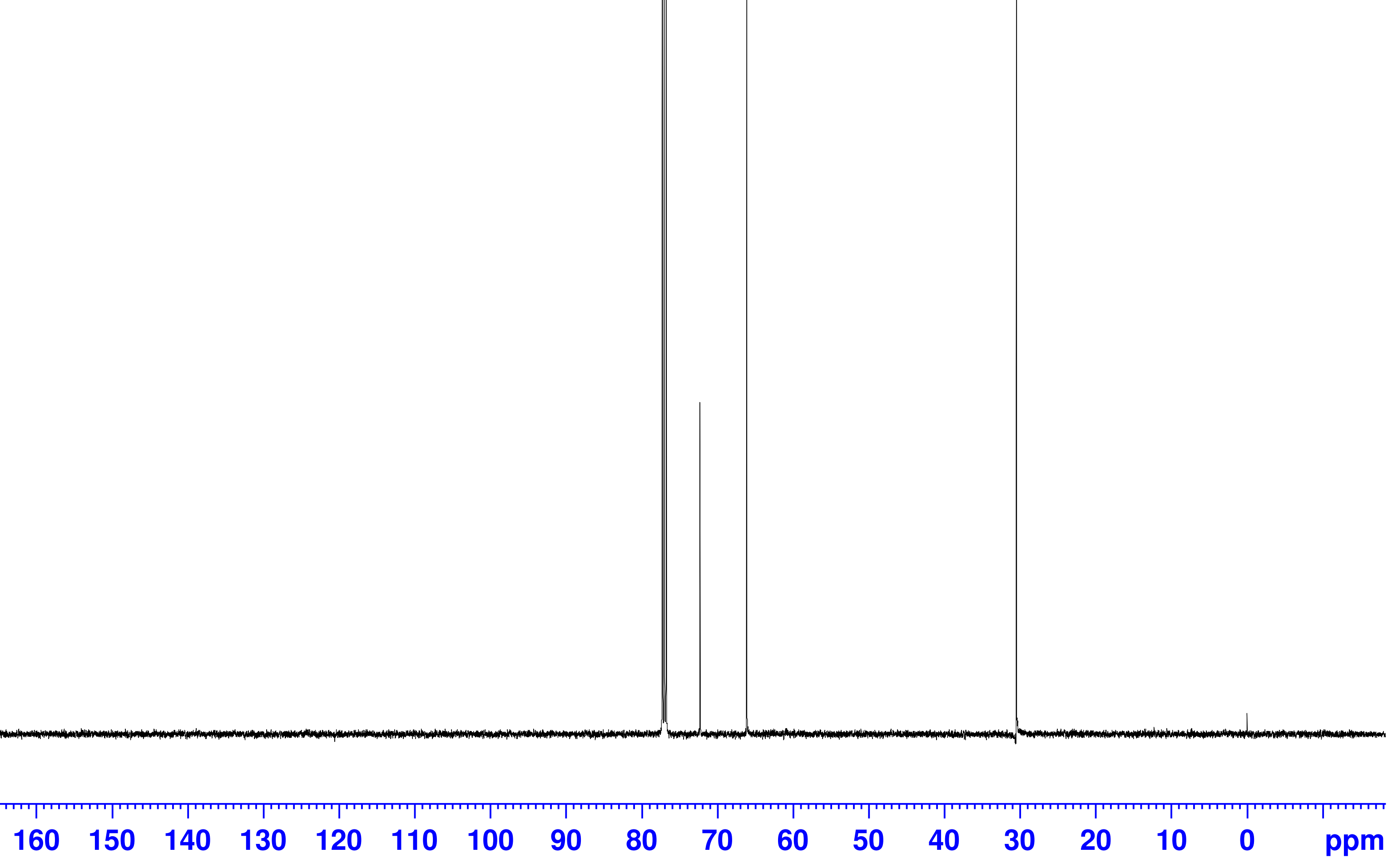

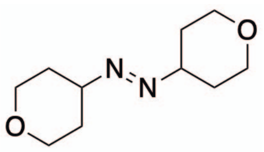

2y

```
Current Data Parameters
NAME             TKR_report
EXPNO                    10
PROCNO                    1

F2 - Acquisition Parameters
Date_            20231121
Time                21.48 h
INSTRUM           spect
PROBHD   Z130033_0007 (
PULPROG            zg30
TD                65536
SOLVENT           CDCl3
NS                    1
DS                    0
SWH            8012.820 Hz
FIDRES         0.244532 Hz
AQ            4.0894465 sec
RG                31.29
DW               62.400 usec
DE                10.00 usec
TE                300.1 K
D1           1.00000000 sec
TD0                   1
SFO1        500.1730010 MHz
NUC1                 1H
P0                 4.00 usec
P1                12.00 usec
PLW1        16.00000000 W

F2 - Processing parameters
SI                65536
SF          500.1700122 MHz
WDW                  EM
SSB                   0
LB                 0.30 Hz
GB                    0
PC                 1.00
```

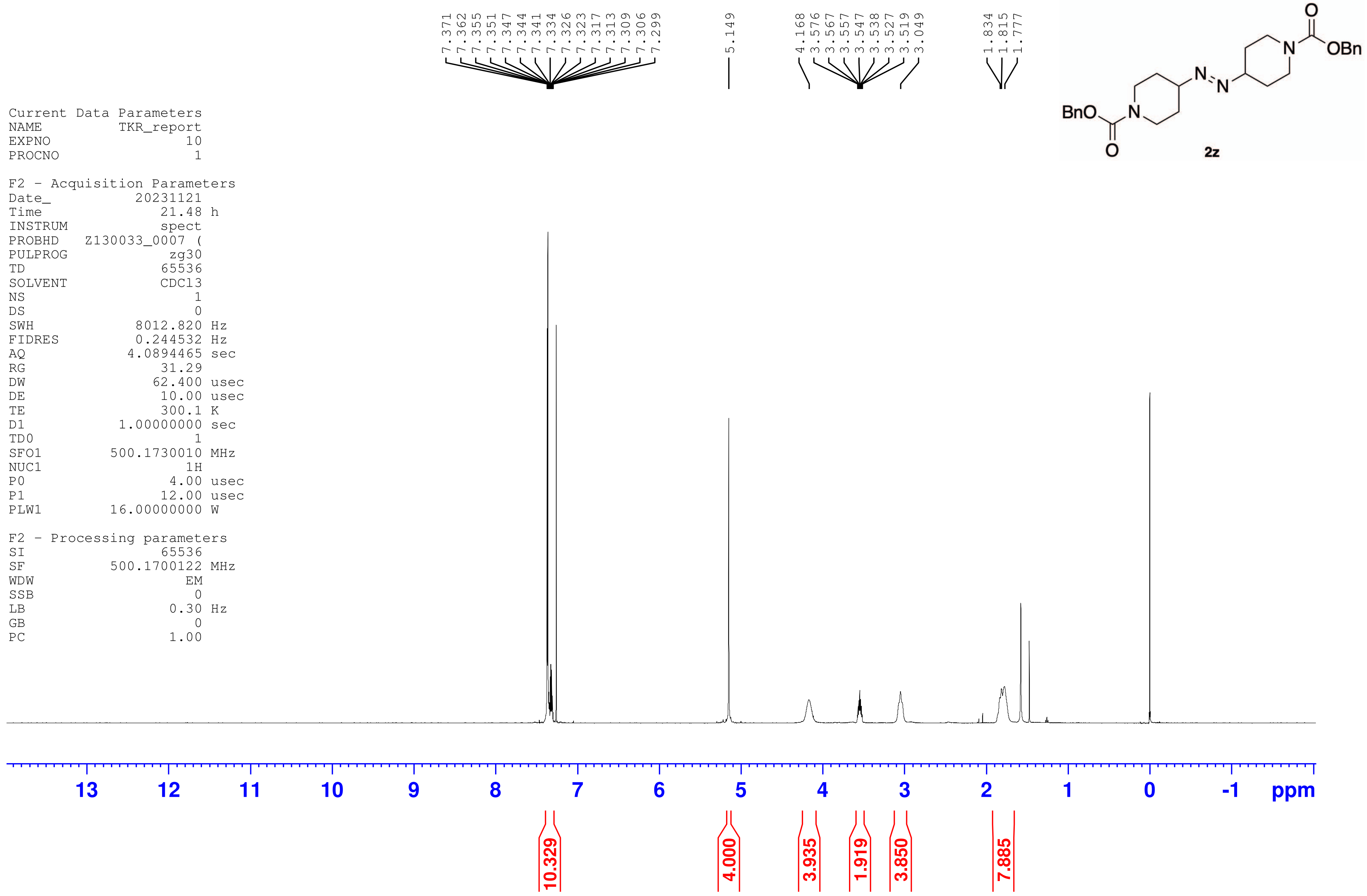

2z

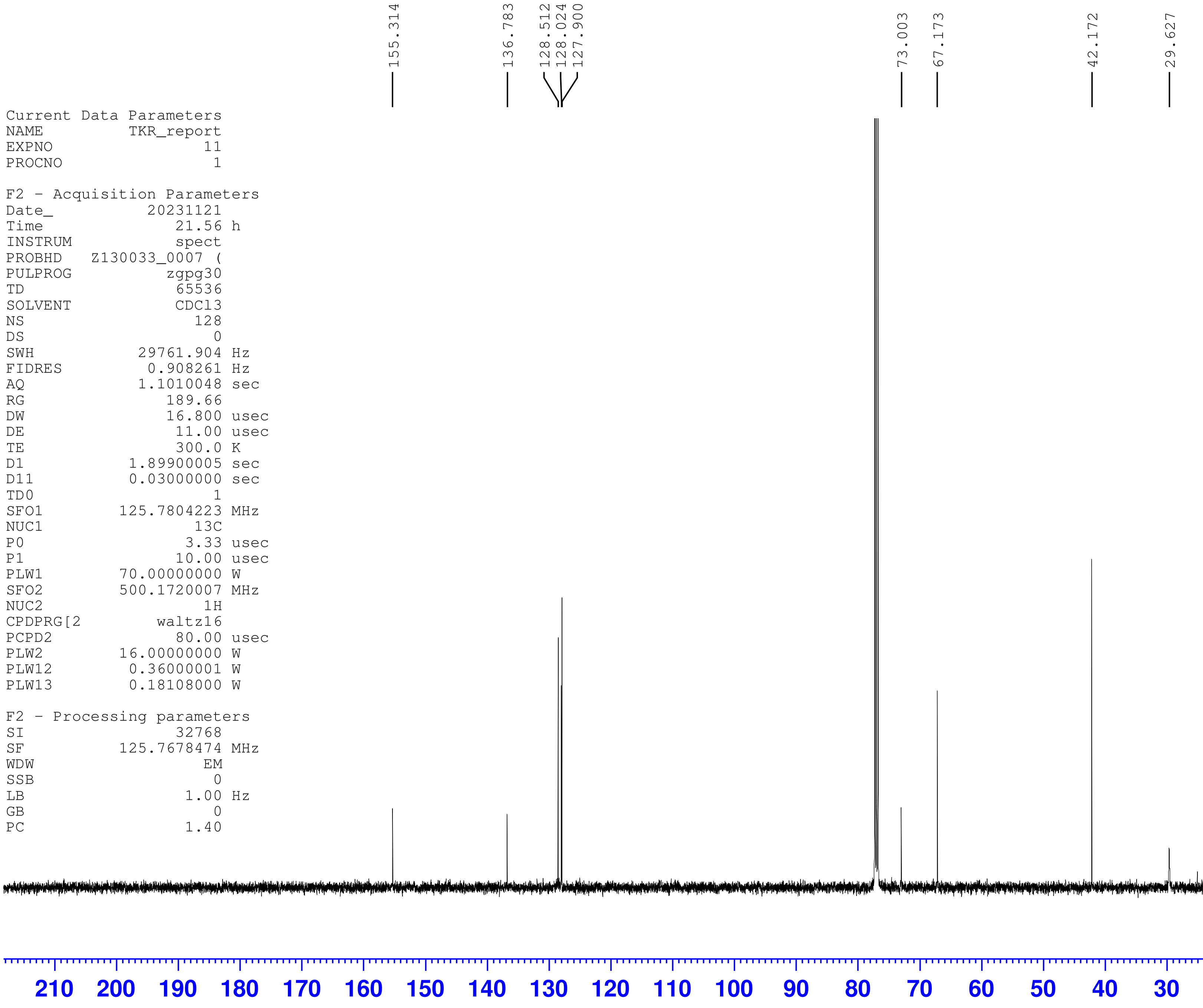
155.314
136.783
128.512
128.024
127.900
73.003
67.173
42.172
29.627
Current Data Parameters
NAME TKR_report
EXPNO 11
PROCNO 1
F2 - Acquisition Parameters
Date_ 20231121
Time 21.56 h
INSTRUM spect
PROBHD Z130033_0007 (
PULPROG zgpg30
TD 65536
SOLVENT CDCl3
NS 128
DS 0
SWH 29761.904 Hz
FIDRES 0.908261 Hz
AQ 1.1010048 sec
RG 189.66
DW 16.800 usec
DE 11.00 usec
TE 300.0 K
D1 1.89900005 sec
D11 0.03000000 sec
TD0 1
SFO1 125.7804223 MHz
NUC1 13C
P0 3.33 usec
P1 10.00 usec
PLW1 70.00000000 W
SFO2 500.1720007 MHz
NUC2 1H
CPDPRG[2 waltz16
PCPD2 80.00 usec
PLW2 16.00000000 W
PLW12 0.36000001 W
PLW13 0.18108000 W
F2 - Processing parameters
SI 32768
SF 125.7678474 MHz
WDW EM
SSB 0
LB 1.00 Hz
GB 0
PC 1.40
210
200
190
180
170
160
150
140
130
120
110
100
90
80
70
60
50
40
30
20
10
0
ppm

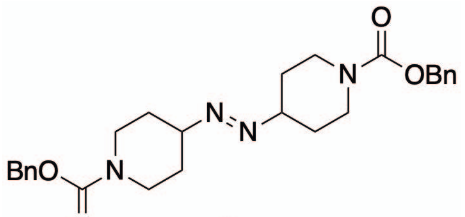
BnO
N
N
N
N
O
O
OBn

2z

Current Data Parameters
NAME tsu-e3-380c
EXPNO 40
PROCNO 1

F2 - Acquisition Parameters
Date_ 20230505
Time 15.38 h
INSTRUM spect
PROBHD Z119470_0344 (
PULPROG zg30
TD 65536
SOLVENT CDCl3
NS 1
DS 0
SWH 8012.820 Hz
FIDRES 0.244532 Hz
AQ 4.0894465 sec
RG 116.65
DW 62.400 usec
DE 6.50 usec
TE 298.0 K
D1 1.00000000 sec
TD0 1
SFO1 500.1730010 MHz
NUC1 1H
P0 4.83 usec
P1 14.50 usec
PLW1 10.80000019 W

F2 - Processing parameters
SI 65536
SF 500.1700127 MHz
WDW EM
SSB 0
LB 0.30 Hz
GB 0
PC 1.00

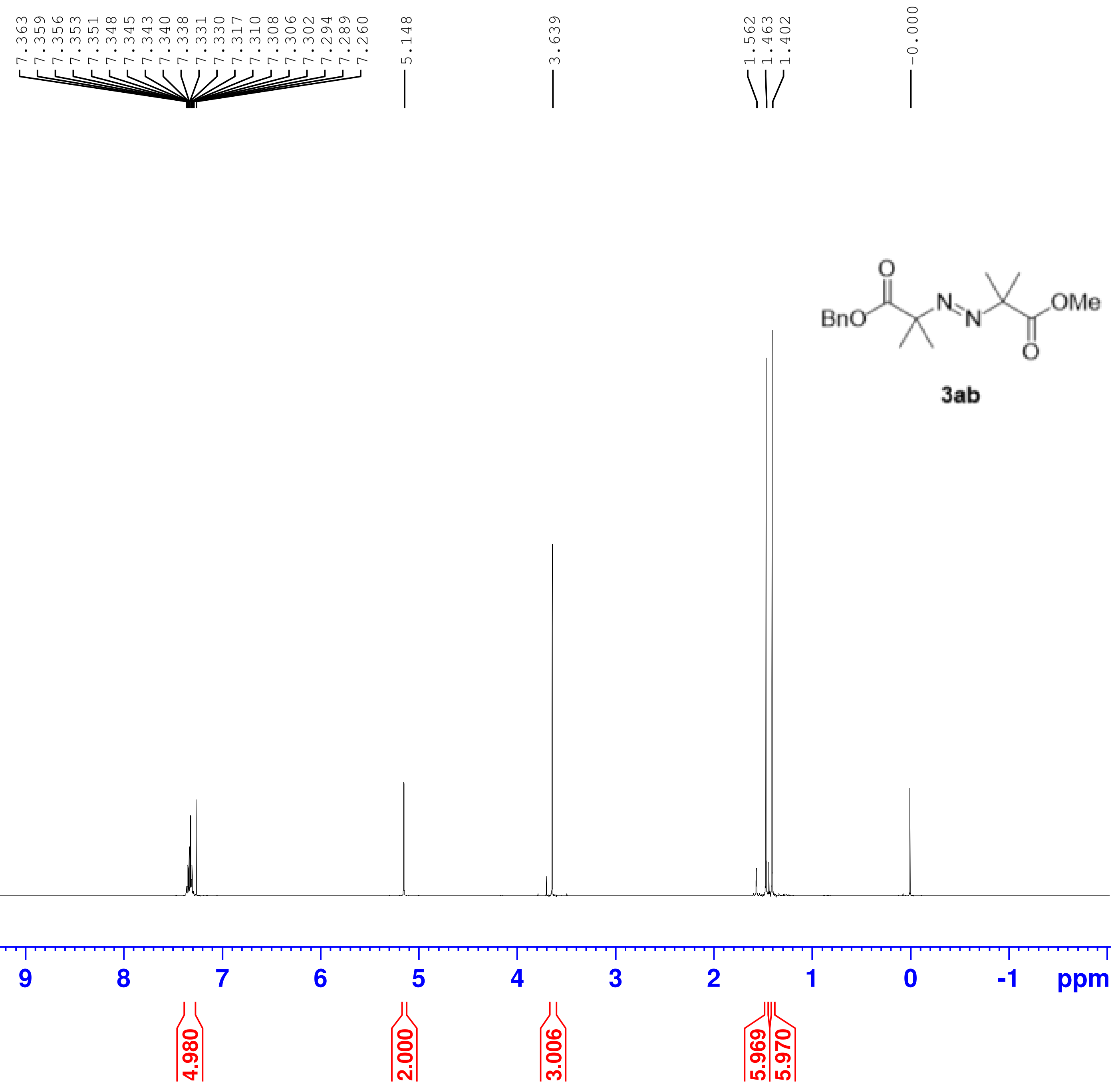

3ab

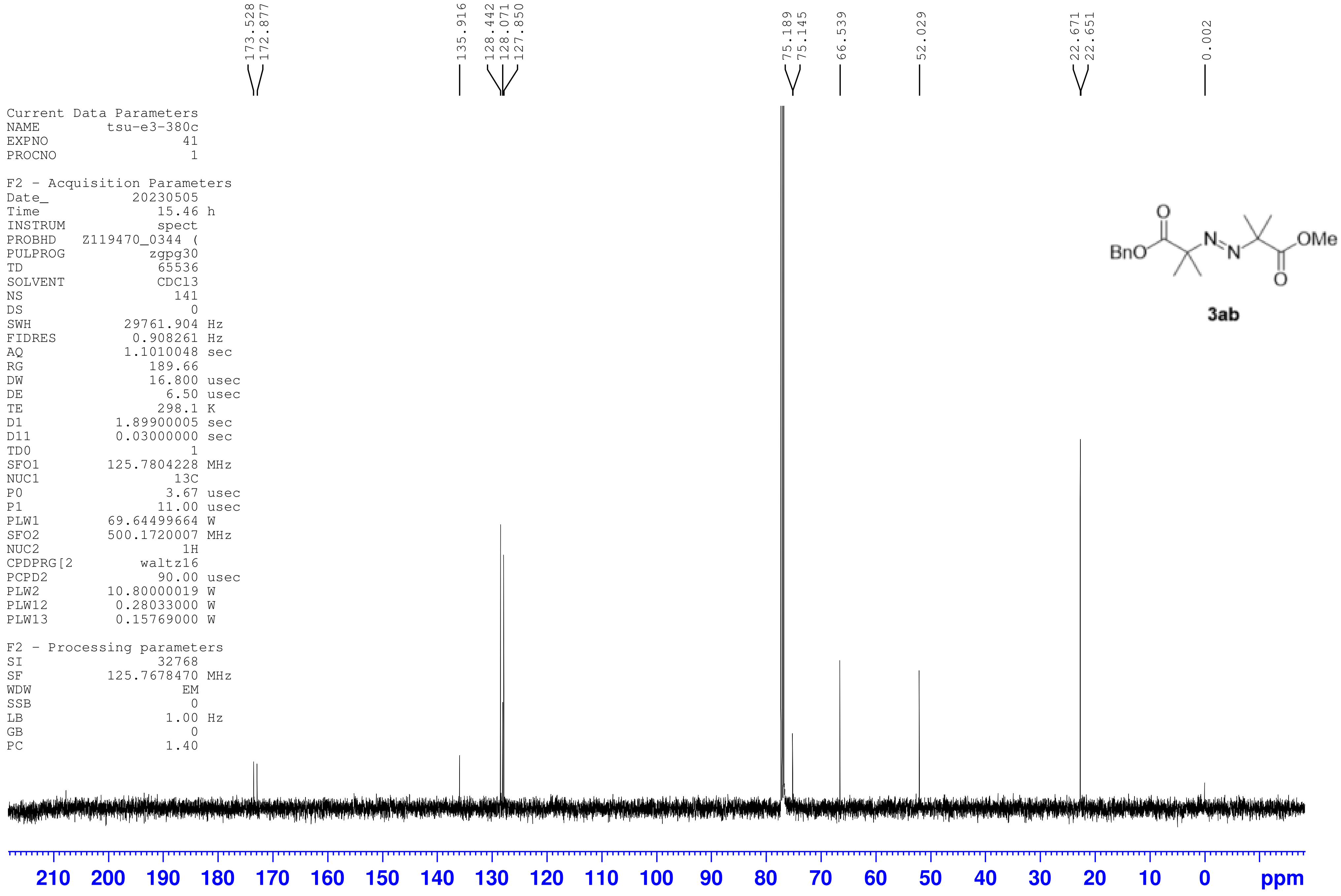
173.528
172.877
135.916
128.442
128.071
127.850
75.189
75.145
66.539
52.029
22.671
22.651
0.002
Current Data Parameters
NAME tsu-e3-380c
EXPNO 41
PROCNO 1
F2 - Acquisition Parameters
Date_ 20230505
Time 15.46 h
INSTRUM spect
PROBHD Z119470_0344 (
PULPROG zgpg30
TD 65536
SOLVENT CDCl3
NS 141
DS 0
SWH 29761.904 Hz
FIDRES 0.908261 Hz
AQ 1.1010048 sec
RG 189.66
DW 16.800 usec
DE 6.50 usec
TE 298.1 K
D1 1.89900005 sec
D11 0.03000000 sec
TD0 1
SFO1 125.7804228 MHz
NUC1 13C
P0 3.67 usec
P1 11.00 usec
PLW1 69.64499664 W
SFO2 500.1720007 MHz
NUC2 1H
CPDPRG[2 waltz16
PCPD2 90.00 usec
PLW2 10.80000019 W
PLW12 0.28033000 W
PLW13 0.15769000 W
F2 - Processing parameters
SI 32768
SF 125.7678470 MHz
WDW EM
SSB 0
LB 1.00 Hz
GB 0
PC 1.40
210 200 190 180 170 160 150 140 130 120 110 100 90 80 70 60 50 40 30 20 10 0 ppm
BnO
O
N
N
OMe
O

3ab

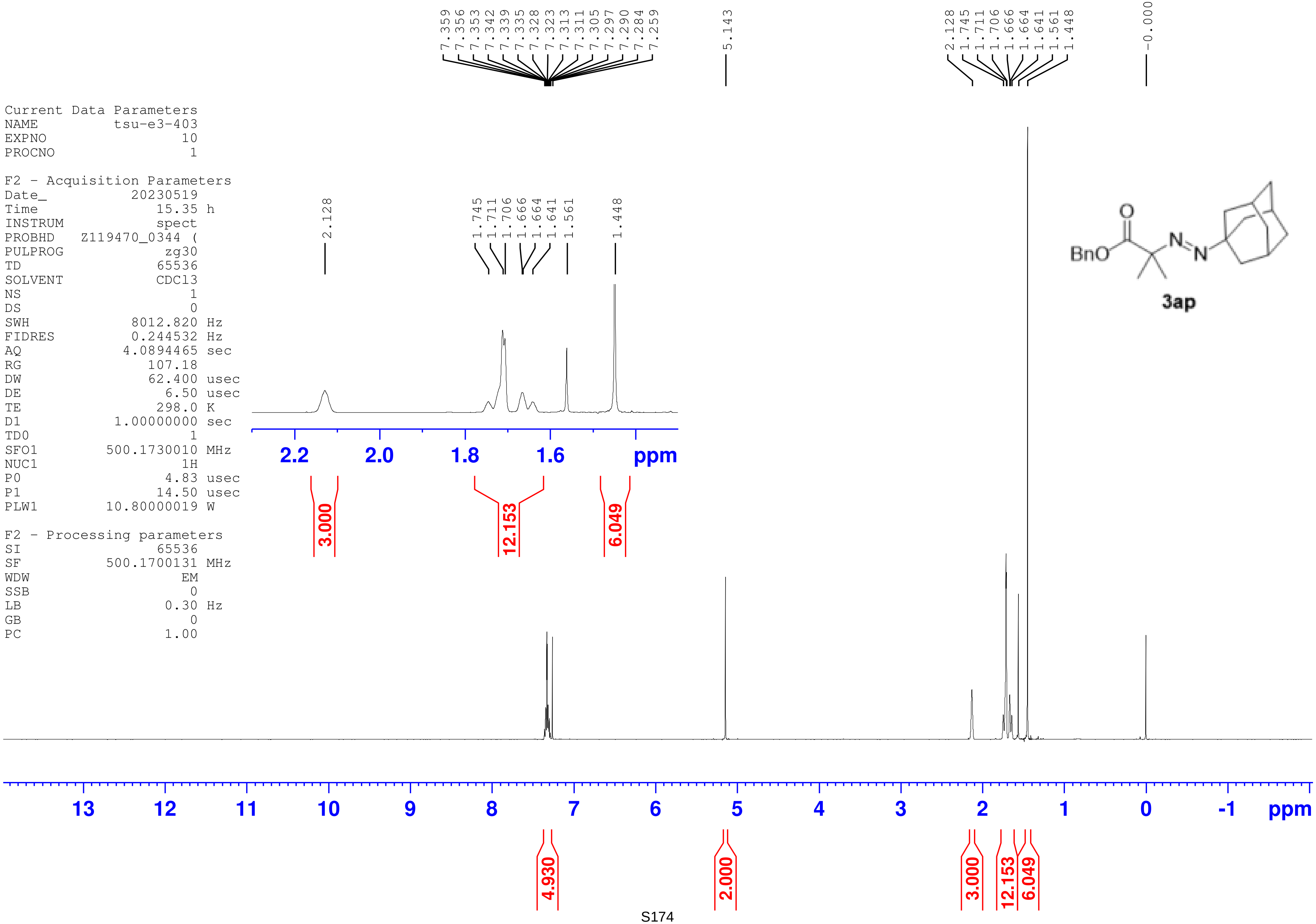


```
Current Data Parameters
NAME          tsu-e3-403
EXPNO                 10
PROCNO                 1

F2 - Acquisition Parameters
Date_           20230519
Time               15.35 h
INSTRUM            spect
PROBHD   Z119470_0344 (
PULPROG             zg30
TD                 65536
SOLVENT            CDCl3
NS                     1
DS                     0
SWH             8012.820 Hz
FIDRES          0.244532 Hz
AQ             4.0894465 sec
RG                107.18
DW                62.400 usec
DE                  6.50 usec
TE                 298.0 K
D1            1.00000000 sec
TD0                    1
SFO1         500.1730010 MHz
NUC1                  1H
P0                  4.83 usec
P1                 14.50 usec
PLW1         10.80000019 W

F2 - Processing parameters
SI                 65536
SF           500.1700131 MHz
WDW                   EM
SSB                    0
LB                  0.30 Hz
GB                     0
PC                  1.00
```

3ap

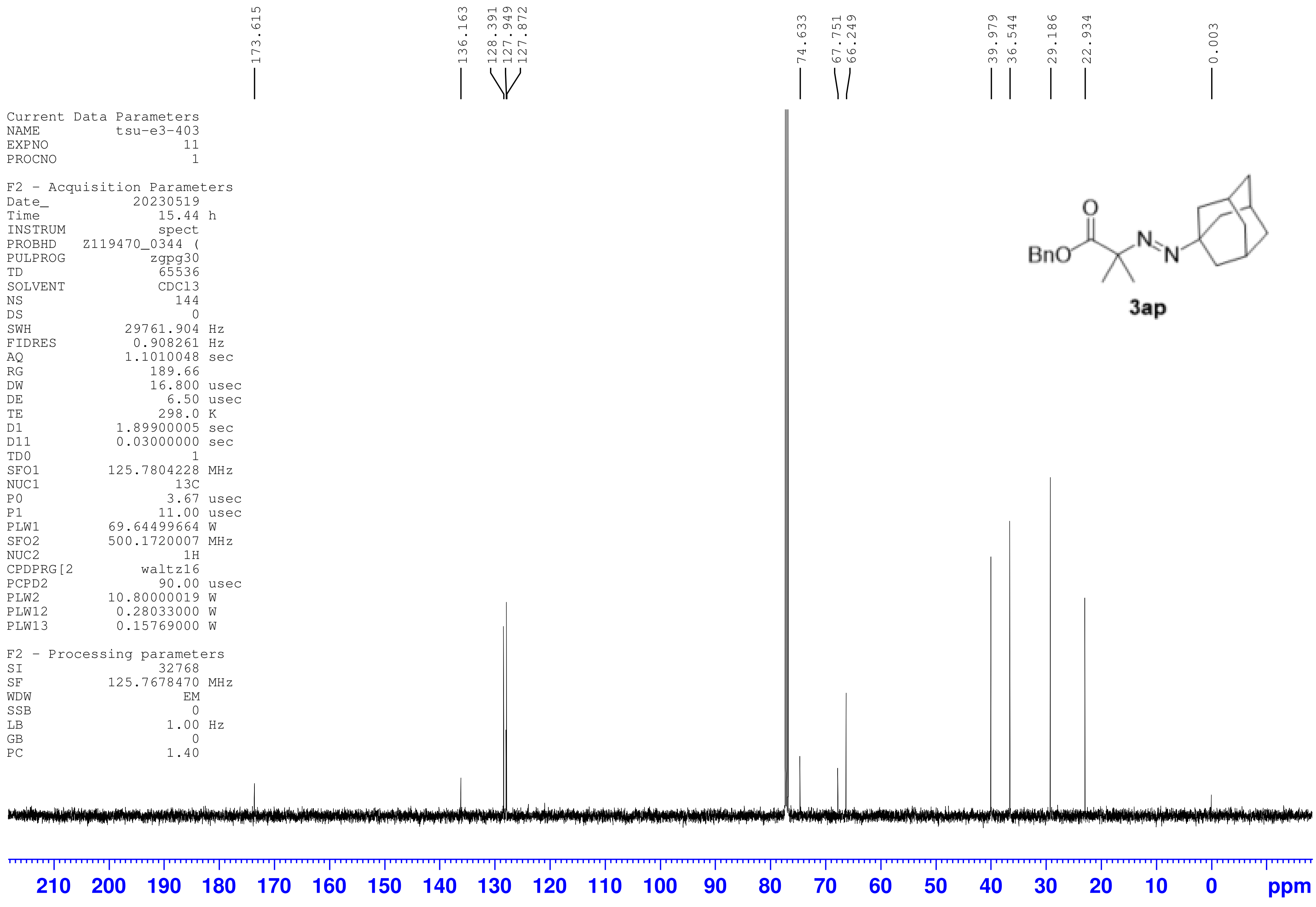
173.615
136.163
128.391
127.949
127.872
74.633
67.751
66.249
39.979
36.544
29.186
22.934
0.003
Current Data Parameters
NAME tsu-e3-403
EXPNO 11
PROCNO 1
F2 - Acquisition Parameters
Date_ 20230519
Time 15.44 h
INSTRUM spect
PROBHD Z119470_0344 (
PULPROG zgpg30
TD 65536
SOLVENT CDCl3
NS 144
DS 0
SWH 29761.904 Hz
FIDRES 0.908261 Hz
AQ 1.1010048 sec
RG 189.66
DW 16.800 usec
DE 6.50 usec
TE 298.0 K
D1 1.89900005 sec
D11 0.03000000 sec
TD0 1
SFO1 125.7804228 MHz
NUC1 13C
P0 3.67 usec
P1 11.00 usec
PLW1 69.64499664 W
SFO2 500.1720007 MHz
NUC2 1H
CPDPRG[2 waltz16
PCPD2 90.00 usec
PLW2 10.80000019 W
PLW12 0.28033000 W
PLW13 0.15769000 W
F2 - Processing parameters
SI 32768
SF 125.7678470 MHz
WDW EM
SSB 0
LB 1.00 Hz
GB 0
PC 1.40
210 200 190 180 170 160 150 140 130 120 110 100 90 80 70 60 50 40 30 20 10 0 ppm
BnO
O
N
N

3ap

```
Current Data Parameters
NAME          tsu-e3-553b
EXPNO                  10
PROCNO                  1

F2 - Acquisition Parameters
Date_            20230922
Time                17.44 h
INSTRUM             spect
PROBHD    Z119470_0344 (
PULPROG              zg30
TD                  65536
SOLVENT             CDCl3
NS                      1
DS                      0
SWH              8012.820 Hz
FIDRES           0.244532 Hz
AQ              4.0894465 sec
RG                 130.52
DW                 62.400 usec
DE                   6.50 usec
TE                  300.0 K
D1             1.00000000 sec
TD0                     1
SFO1          500.1730010 MHz
NUC1                   1H
P0                   4.83 usec
P1                  14.50 usec
PLW1          10.80000019 W

F2 - Processing parameters
SI                  65536
SF            500.1700140 MHz
WDW                    EM
SSB                     0
LB                   0.30 Hz
GB                      0
PC                   1.00
```

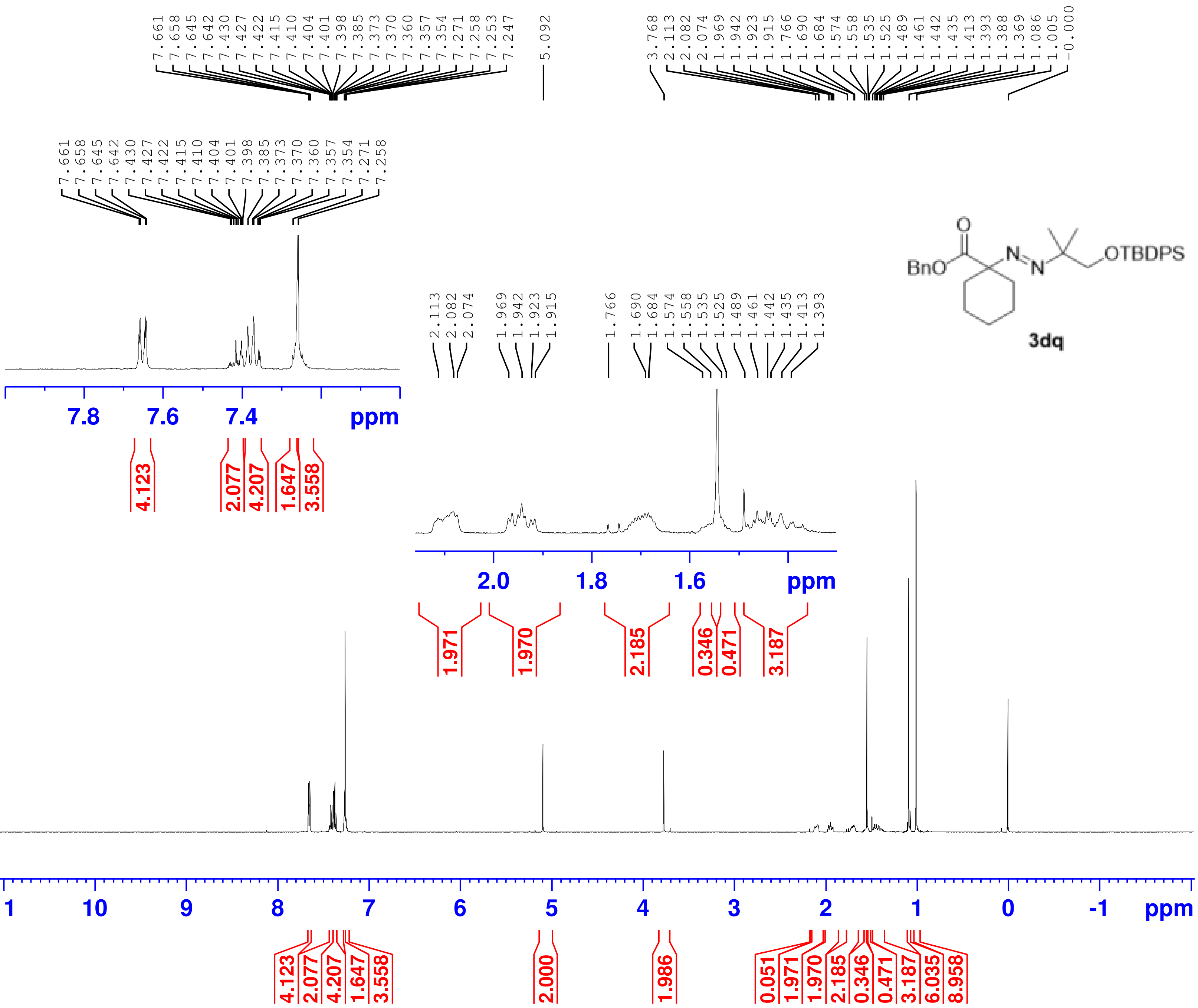

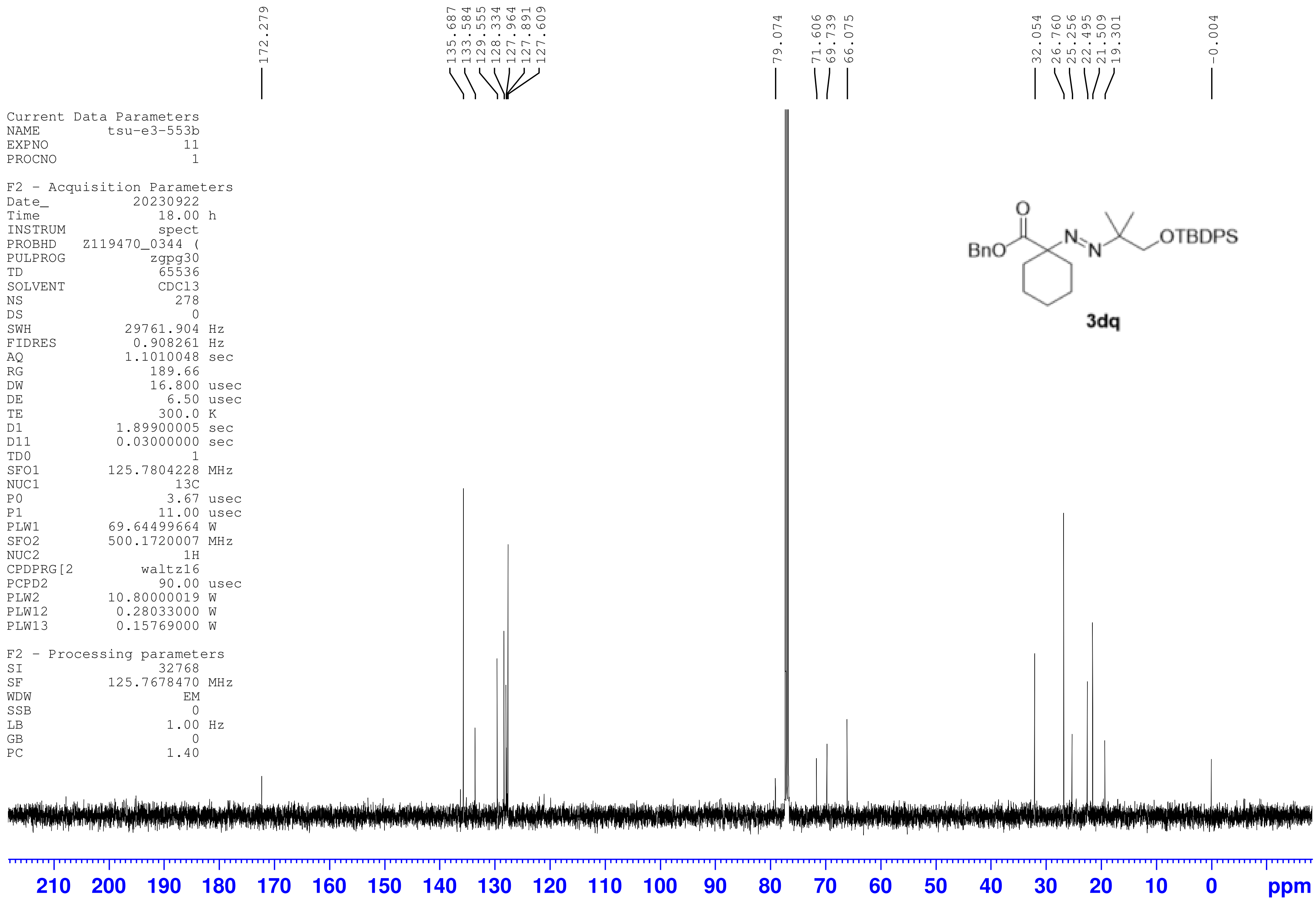


```
Current Data Parameters
NAME          tsu-e3-553b
EXPNO                  11
PROCNO                  1

F2 - Acquisition Parameters
Date_            20230922
Time                18.00 h
INSTRUM             spect
PROBHD   Z119470_0344 (
PULPROG           zgpg30
TD                 65536
SOLVENT            CDCl3
NS                   278
DS                     0
SWH            29761.904 Hz
FIDRES          0.908261 Hz
AQ             1.1010048 sec
RG                189.66
DW                16.800 usec
DE                  6.50 usec
TE                 300.0 K
D1            1.89900005 sec
D11           0.03000000 sec
TD0                    1
SFO1         125.7804228 MHz
NUC1                 13C
P0                  3.67 usec
P1                 11.00 usec
PLW1         69.64499664 W
SFO2         500.1720007 MHz
NUC2                  1H
CPDPRG[2         waltz16
PCPD2              90.00 usec
PLW2         10.80000019 W
PLW12         0.28033000 W
PLW13         0.15769000 W

F2 - Processing parameters
SI                 32768
SF           125.7678470 MHz
WDW                   EM
SSB                    0
LB                  1.00 Hz
GB                     0
PC                  1.40
```

Current Data Parameters
NAME tsu-e3-387aba
EXPNO 10
PROCNO 1

F2 - Acquisition Parameters
Date_ 20230513
Time 13.21 h
INSTRUM spect
PROBHD Z119470_0344 (
PULPROG zg30
TD 65536
SOLVENT CDCl3
NS 1
DS 0
SWH 8012.820 Hz
FIDRES 0.244532 Hz
AQ 4.0894465 sec
RG 116.65
DW 62.400 usec
DE 6.50 usec
TE 298.0 K
D1 1.00000000 sec
TD0 1
SFO1 500.1730010 MHz
NUC1 1H
P0 4.83 usec
P1 14.50 usec
PLW1 10.80000019 W

F2 - Processing parameters
SI 65536
SF 500.1700117 MHz
WDW EM
SSB 0
LB 0.30 Hz
GB 0
PC 1.00

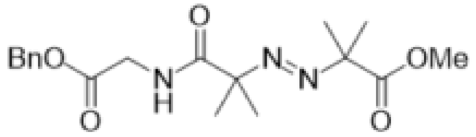

3sb

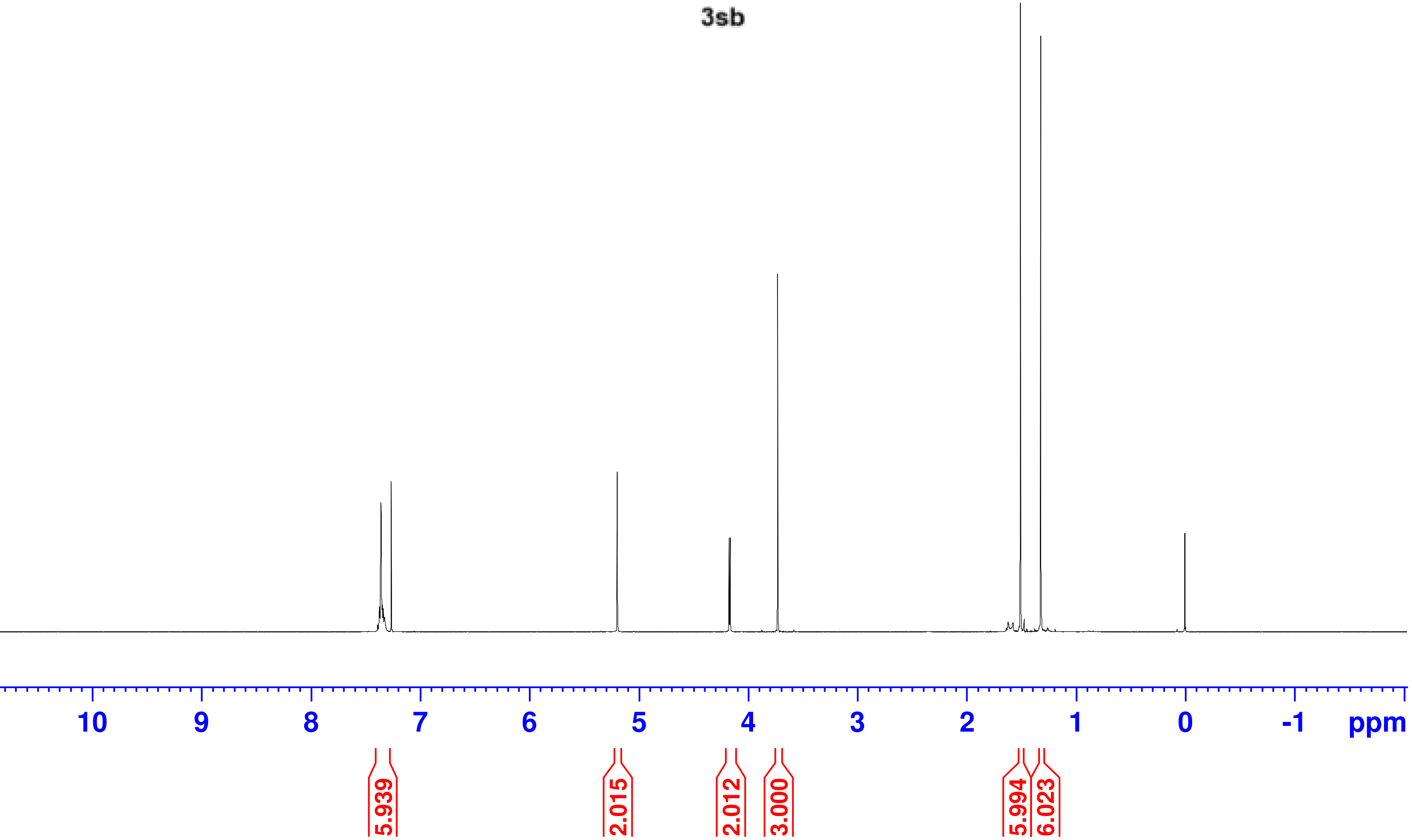

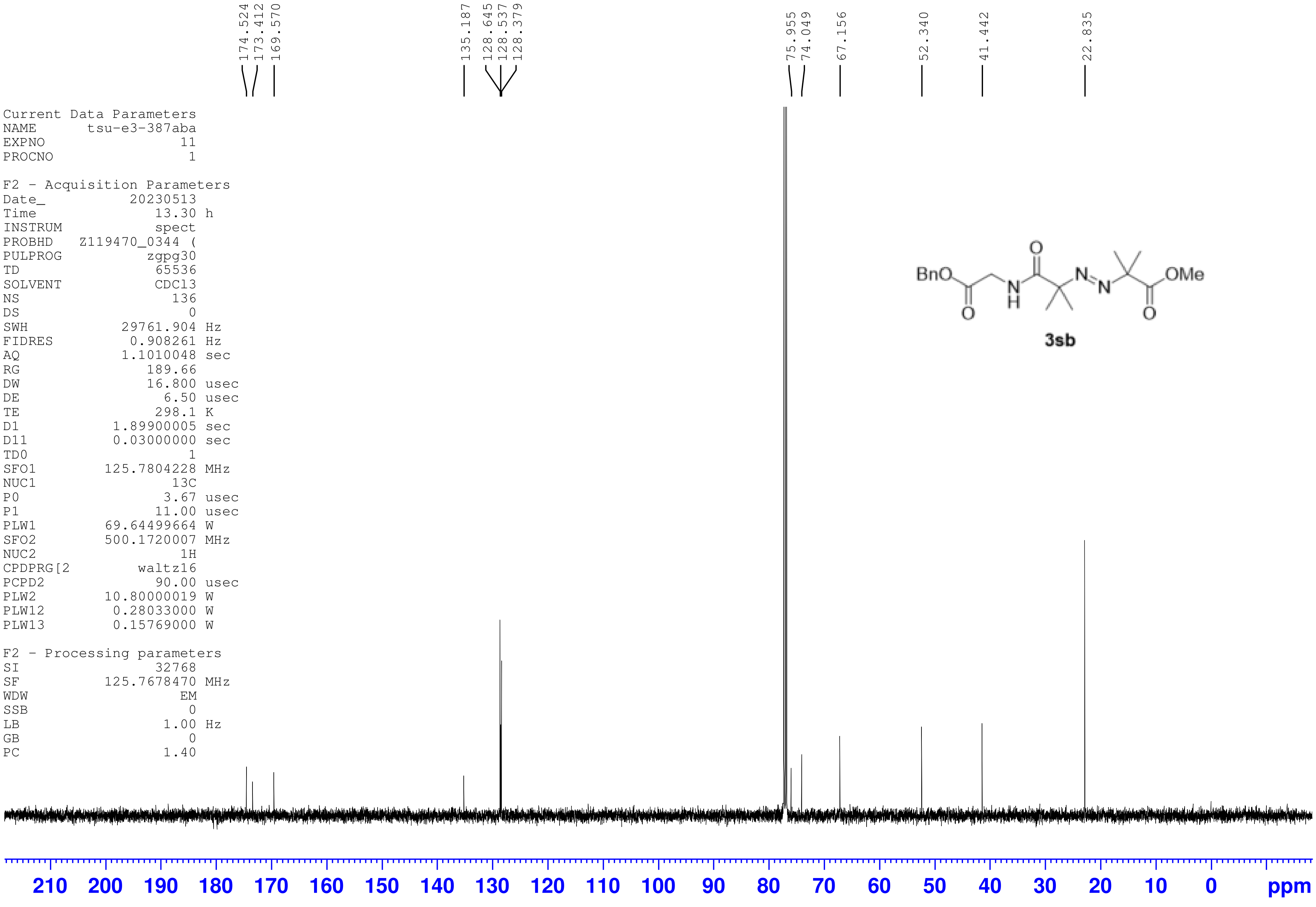


```
Current Data Parameters
NAME       tsu-e3-387aba
EXPNO                 11
PROCNO                 1

F2 - Acquisition Parameters
Date_           20230513
Time               13.30 h
INSTRUM            spect
PROBHD   Z119470_0344 (
PULPROG          zgpg30
TD                65536
SOLVENT           CDCl3
NS                  136
DS                    0
SWH           29761.904 Hz
FIDRES         0.908261 Hz
AQ            1.1010048 sec
RG               189.66
DW               16.800 usec
DE                 6.50 usec
TE                298.1 K
D1           1.89900005 sec
D11          0.03000000 sec
TD0                   1
SFO1        125.7804228 MHz
NUC1                13C
P0                 3.67 usec
P1                11.00 usec
PLW1        69.64499664 W
SFO2        500.1720007 MHz
NUC2                 1H
CPDPRG[2        waltz16
PCPD2             90.00 usec
PLW2        10.80000019 W
PLW12        0.28033000 W
PLW13        0.15769000 W

F2 - Processing parameters
SI                32768
SF          125.7678470 MHz
WDW                  EM
SSB                   0
LB                 1.00 Hz
GB                    0
PC                 1.40
```


3sb

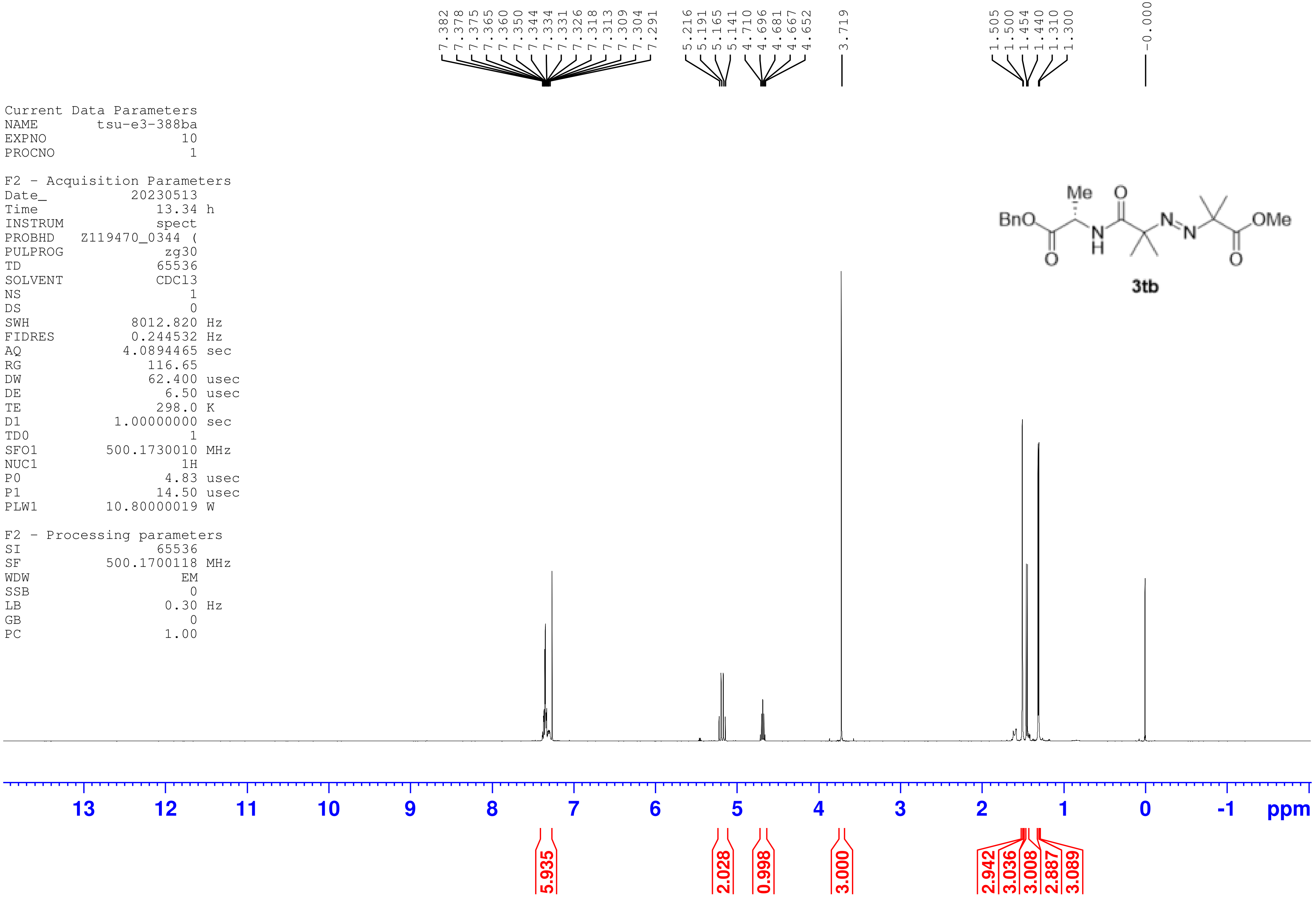

Current Data Parameters
NAME tsu-e3-388ba
EXPNO 10
PROCNO 1

F2 - Acquisition Parameters
Date_ 20230513
Time 13.34 h
INSTRUM spect
PROBHD Z119470_0344 (
PULPROG zg30
TD 65536
SOLVENT CDCl3
NS 1
DS 0
SWH 8012.820 Hz
FIDRES 0.244532 Hz
AQ 4.0894465 sec
RG 116.65
DW 62.400 usec
DE 6.50 usec
TE 298.0 K
D1 1.00000000 sec
TD0 1
SFO1 500.1730010 MHz
NUC1 1H
P0 4.83 usec
P1 14.50 usec
PLW1 10.80000019 W

F2 - Processing parameters
SI 65536
SF 500.1700118 MHz
WDW EM
SSB 0
LB 0.30 Hz
GB 0
PC 1.00

3tb

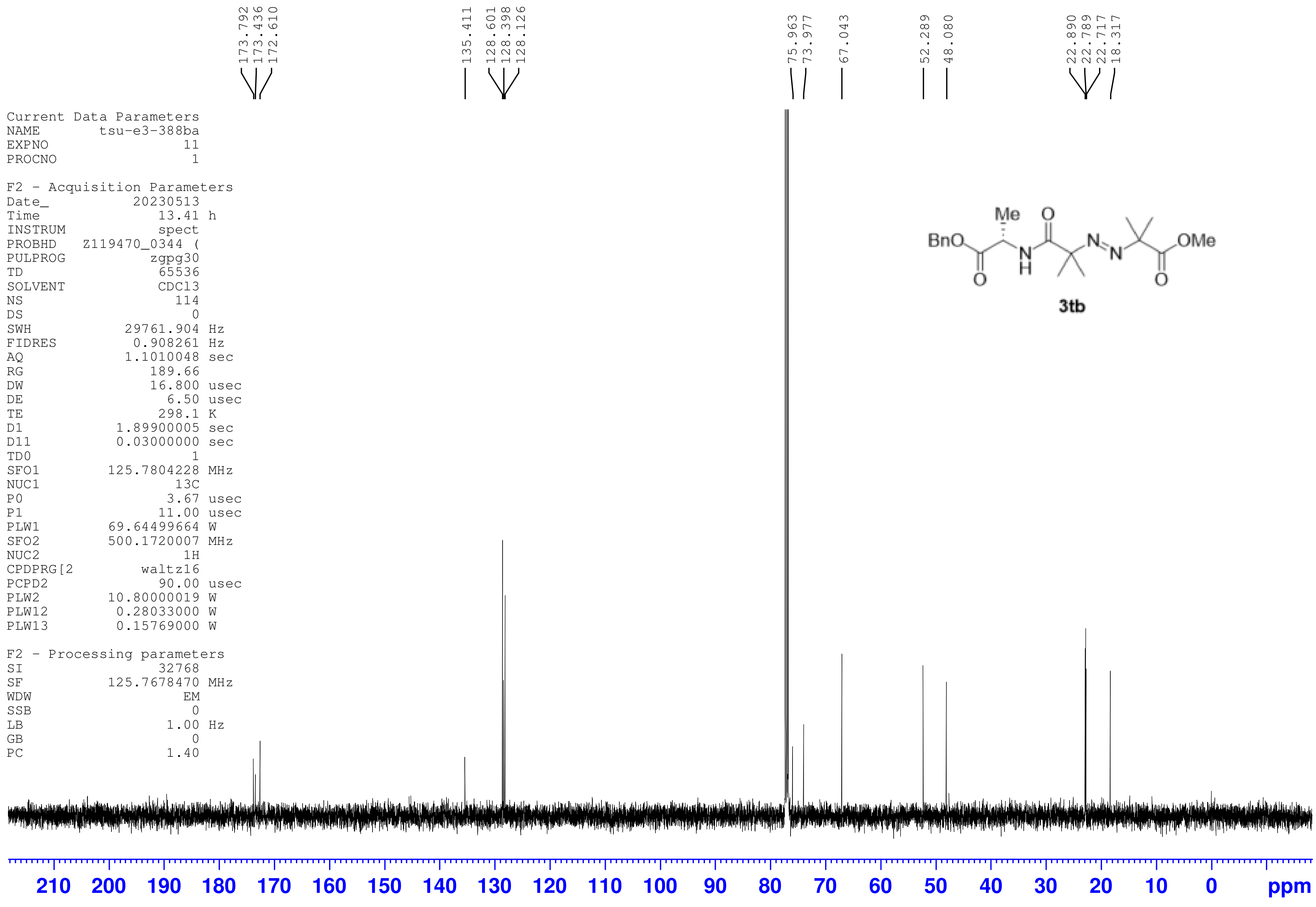
173.792
173.436
172.610
135.411
128.601
128.398
128.126
75.963
73.977
67.043
52.289
48.080
22.890
22.789
22.717
18.317
Current Data Parameters
NAME tsu-e3-388ba
EXPNO 11
PROCNO 1
F2 - Acquisition Parameters
Date_ 20230513
Time 13.41 h
INSTRUM spect
PROBHD Z119470_0344 (
PULPROG zgpg30
TD 65536
SOLVENT CDCl3
NS 114
DS 0
SWH 29761.904 Hz
FIDRES 0.908261 Hz
AQ 1.1010048 sec
RG 189.66
DW 16.800 usec
DE 6.50 usec
TE 298.1 K
D1 1.89900005 sec
D11 0.03000000 sec
TD0 1
SFO1 125.7804228 MHz
NUC1 13C
P0 3.67 usec
P1 11.00 usec
PLW1 69.64499664 W
SFO2 500.1720007 MHz
NUC2 1H
CPDPRG[2 waltz16
PCPD2 90.00 usec
PLW2 10.80000019 W
PLW12 0.28033000 W
PLW13 0.15769000 W
F2 - Processing parameters
SI 32768
SF 125.7678470 MHz
WDW EM
SSB 0
LB 1.00 Hz
GB 0
PC 1.40
210 200 190 180 170 160 150 140 130 120 110 100 90 80 70 60 50 40 30 20 10 0 ppm
BnO
Me
O
N
H
N
N
OMe
O
O

3tb

```
Current Data Parameters
NAME      FKI-E-05-0047 CD3OD
EXPNO                    10
PROCNO                    1

F2 - Acquisition Parameters
Date_              20250503
Time                  12.38 h
INSTRUM               spect
PROBHD    Z130033_0007 (
PULPROG               zg30
TD                    65536
SOLVENT                MeOD
NS                        1
DS                        0
SWH                8012.820 Hz
FIDRES             0.244532 Hz
AQ                4.0894465 sec
RG                    17.03
DW                   62.400 usec
DE                    10.00 usec
TE                    300.0 K
D1               1.00000000 sec
TD0                       1
SFO1            500.1730010 MHz
NUC1                     1H
P0                     4.00 usec
P1                    12.00 usec
PLW1            16.00000000 W

F2 - Processing parameters
SI                    65536
SF              500.1700610 MHz
WDW                      EM
SSB                       0
LB                     0.30 Hz
GB                        0
PC                     1.00
```

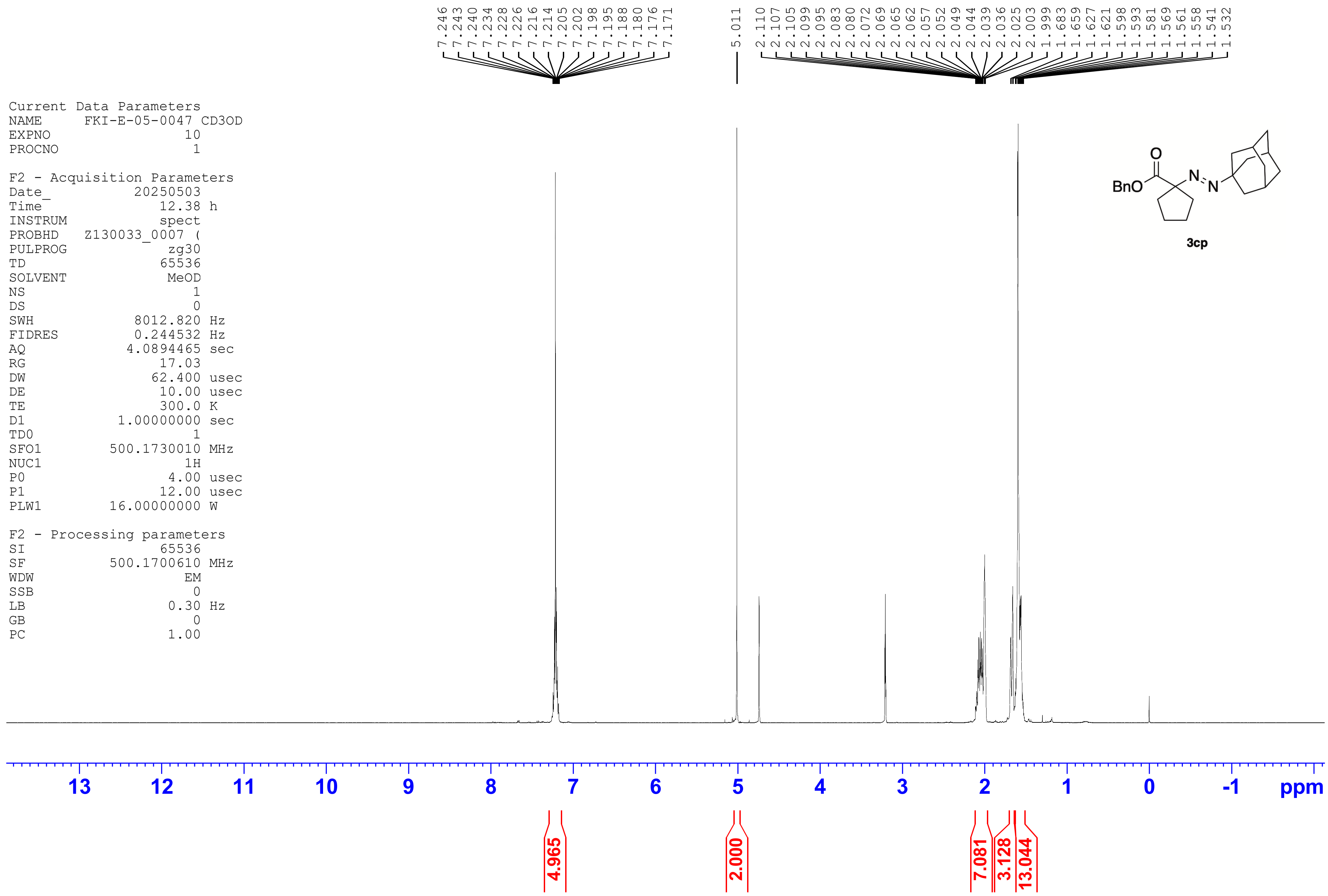

3cp

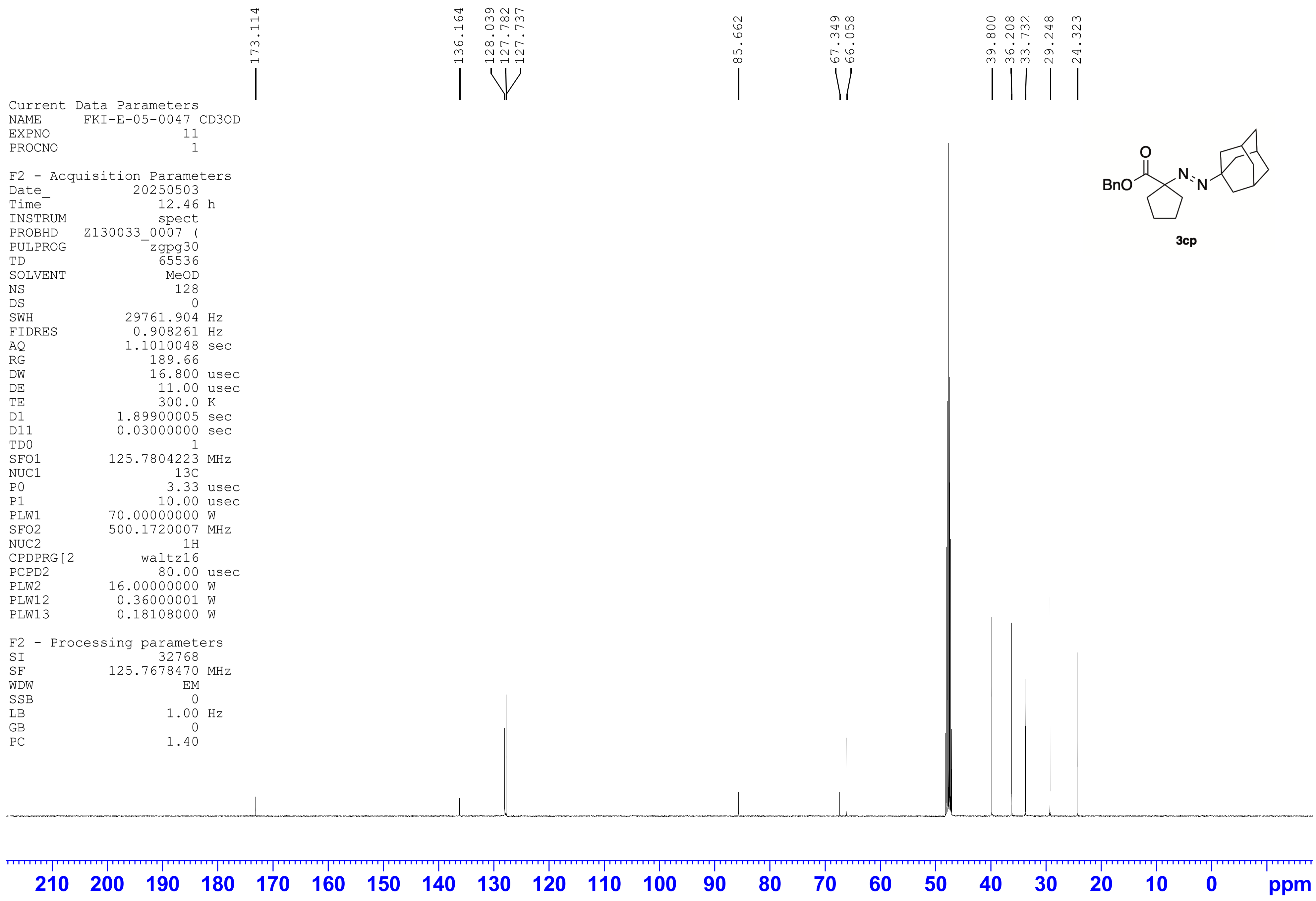
173.114
136.164
128.039
127.782
127.737
85.662
67.349
66.058
39.800
36.208
33.732
29.248
24.323
Current Data Parameters
NAME FKI-E-05-0047 CD3OD
EXPNO 11
PROCNO 1
F2 - Acquisition Parameters
Date_ 20250503
Time_ 12.46 h
INSTRUM spect
PROBHD Z130033_0007 (
PULPROG zgpg30
TD 65536
SOLVENT MeOD
NS 128
DS 0
SWH 29761.904 Hz
FIDRES 0.908261 Hz
AQ 1.1010048 sec
RG 189.66
DW 16.800 usec
DE 11.00 usec
TE 300.0 K
D1 1.89900005 sec
D11 0.03000000 sec
TD0 1
SFO1 125.7804223 MHz
NUC1 13C
P0 3.33 usec
P1 10.00 usec
PLW1 70.00000000 W
SFO2 500.1720007 MHz
NUC2 1H
CPDPRG[2 waltz16
PCPD2 80.00 usec
PLW2 16.00000000 W
PLW12 0.36000001 W
PLW13 0.18108000 W
F2 - Processing parameters
SI 32768
SF 125.7678470 MHz
WDW EM
SSB 0
LB 1.00 Hz
GB 0
PC 1.40
BnO
O
N
N
3cp
210 200 190 180 170 160 150 140 130 120 110 100 90 80 70 60 50 40 30 20 10 0 ppm

```
Current Data Parameters
NAME       FKI-E-05-0024 TM
EXPNO                    10
PROCNO                    1

F2 - Acquisition Parameters
Date_              20240704
Time                  11.21 h
INSTRUM               spect
PROBHD    Z130033_0007 (
PULPROG                zg30
TD                    65536
SOLVENT               CDCl3
NS                        1
DS                        0
SWH                8012.820 Hz
FIDRES             0.244532 Hz
AQ                4.0894465 sec
RG                    17.03
DW                   62.400 usec
DE                    10.00 usec
TE                    300.1 K
D1               1.00000000 sec
TD0                       1
SFO1            500.1730010 MHz
NUC1                     1H
P0                     4.00 usec
P1                    12.00 usec
PLW1            16.00000000 W

F2 - Processing parameters
SI                    65536
SF              500.1700142 MHz
WDW                      EM
SSB                       0
LB                     0.30 Hz
GB                        0
PC                     1.00
```

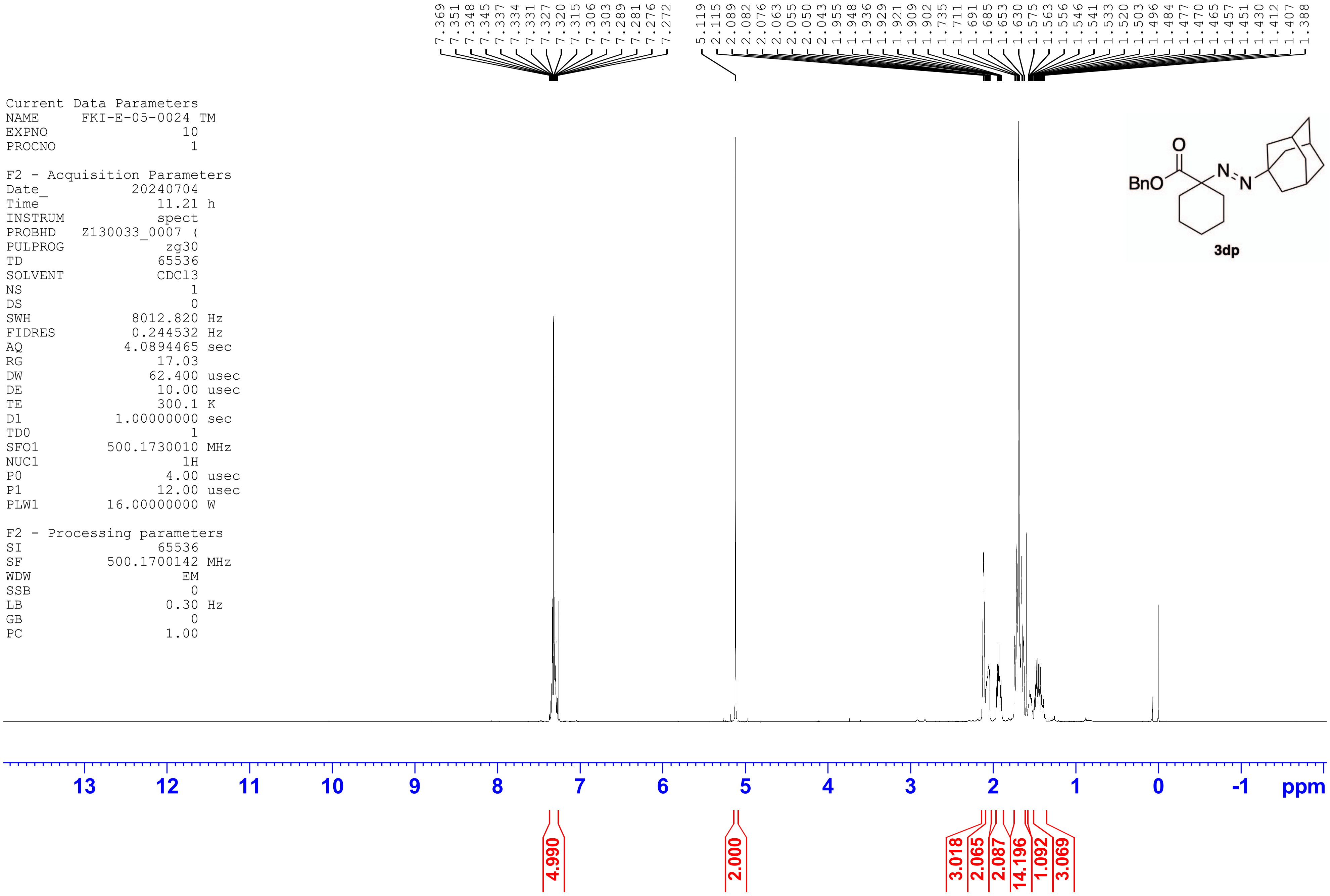

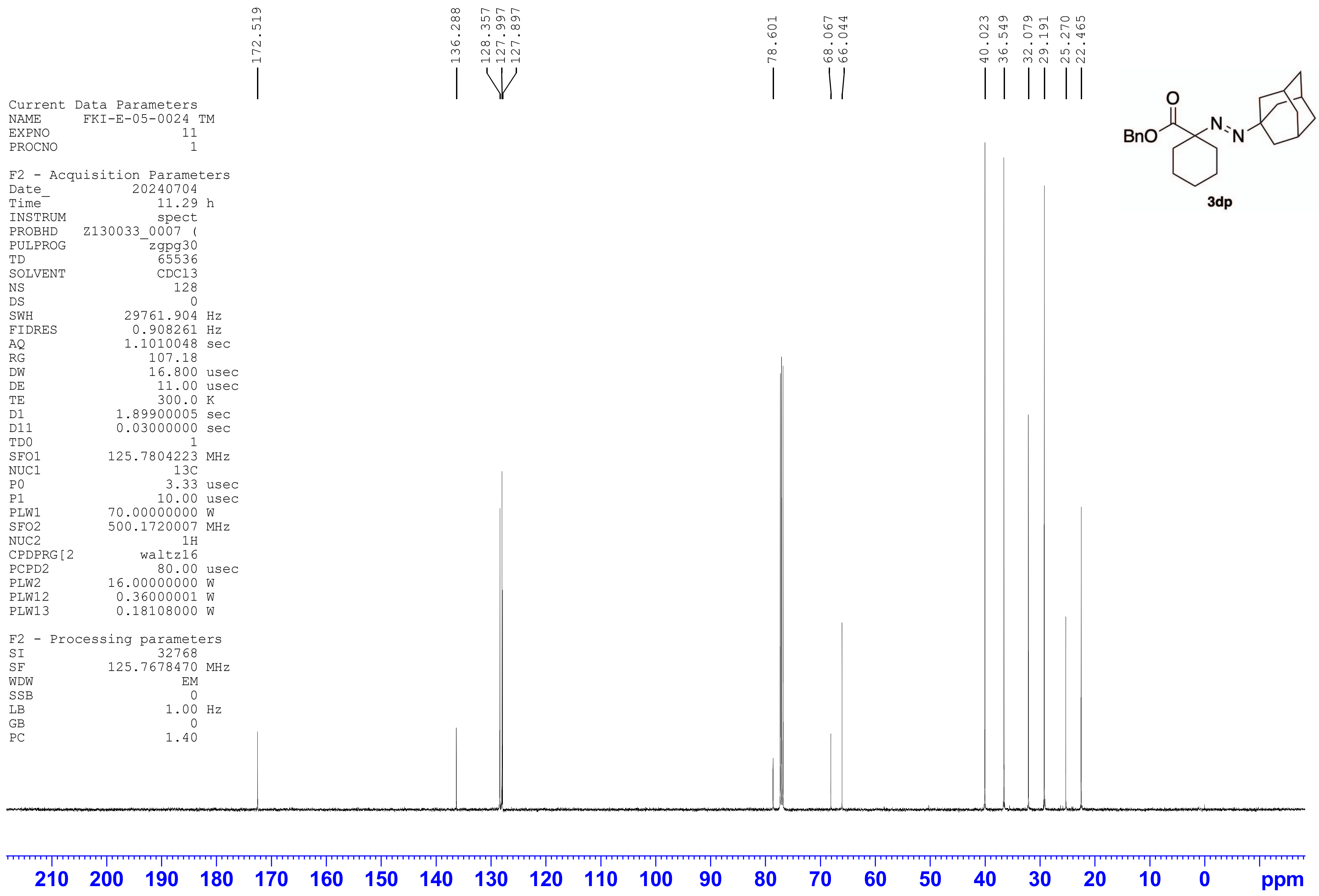

Current Data Parameters
NAME FKI-E-05-0024 TM
EXPNO 11
PROCNO 1
F2 - Acquisition Parameters
Date_ 20240704
Time_ 11.29 h
INSTRUM spect
PROBHD Z130033_0007 (
PULPROG zgpg30
TD 65536
SOLVENT CDCl3
NS 128
DS 0
SWH 29761.904 Hz
FIDRES 0.908261 Hz
AQ 1.1010048 sec
RG 107.18
DW 16.800 usec
DE 11.00 usec
TE 300.0 K
D1 1.89900005 sec
D11 0.03000000 sec
TD0 1
SFO1 125.7804223 MHz
NUC1 13C
P0 3.33 usec
P1 10.00 usec
PLW1 70.00000000 W
SFO2 500.1720007 MHz
NUC2 1H
CPDPRG[2 waltz16
PCPD2 80.00 usec
PLW2 16.00000000 W
PLW12 0.36000001 W
PLW13 0.18108000 W
F2 - Processing parameters
SI 32768
SF 125.7678470 MHz
WDW EM
SSB 0
LB 1.00 Hz
GB 0
PC 1.40
172.519
136.288
128.357
127.997
127.897
78.601
68.067
66.044
40.023
36.549
32.079
29.191
25.270
22.465
210 200 190 180 170 160 150 140 130 120 110 100 90 80 70 60 50 40 30 20 10 0 ppm
BnO
O
N
N

3dp

Current Data Parameters
NAME FKI-E-05-0030 TM
EXPNO 10
PROCNO 1

F2 - Acquisition Parameters
Date_ 20240802
Time 10.35 h
INSTRUM spect
PROBHD Z130033_0007 (
PULPROG zg30
TD 65536
SOLVENT CDCl3
NS 1
DS 0
SWH 8012.820 Hz
FIDRES 0.244532 Hz
AQ 4.0894465 sec
RG 15.99
DW 62.400 usec
DE 10.00 usec
TE 300.1 K
D1 1.00000000 sec
TD0 1
SFO1 500.1730010 MHz
NUC1 1H
P0 4.00 usec
P1 12.00 usec
PLW1 16.00000000 W

F2 - Processing parameters
SI 65536
SF 500.1700044 MHz
WDW EM
SSB 0
LB 0.30 Hz
GB 0
PC 1.00

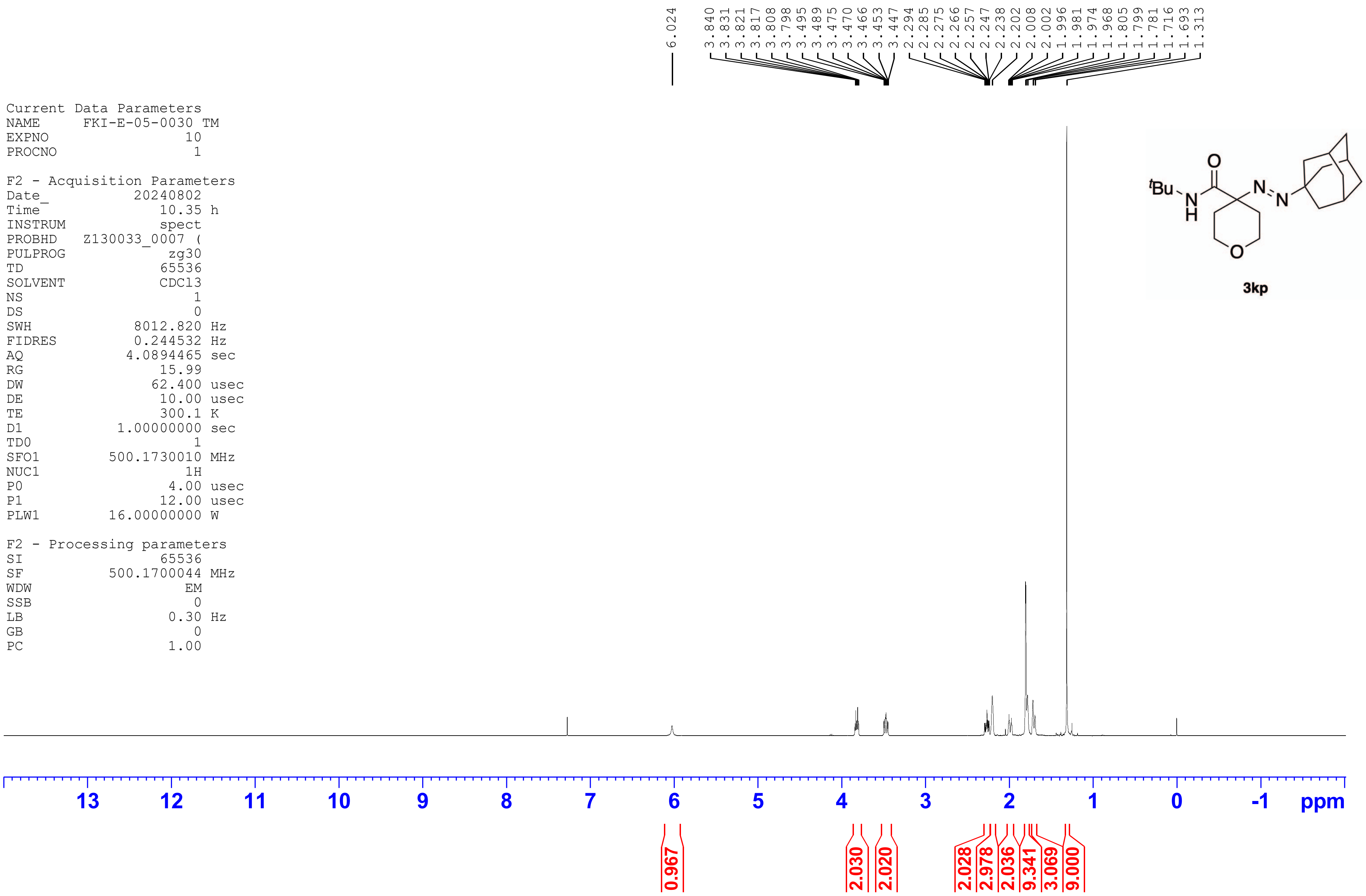

3kp

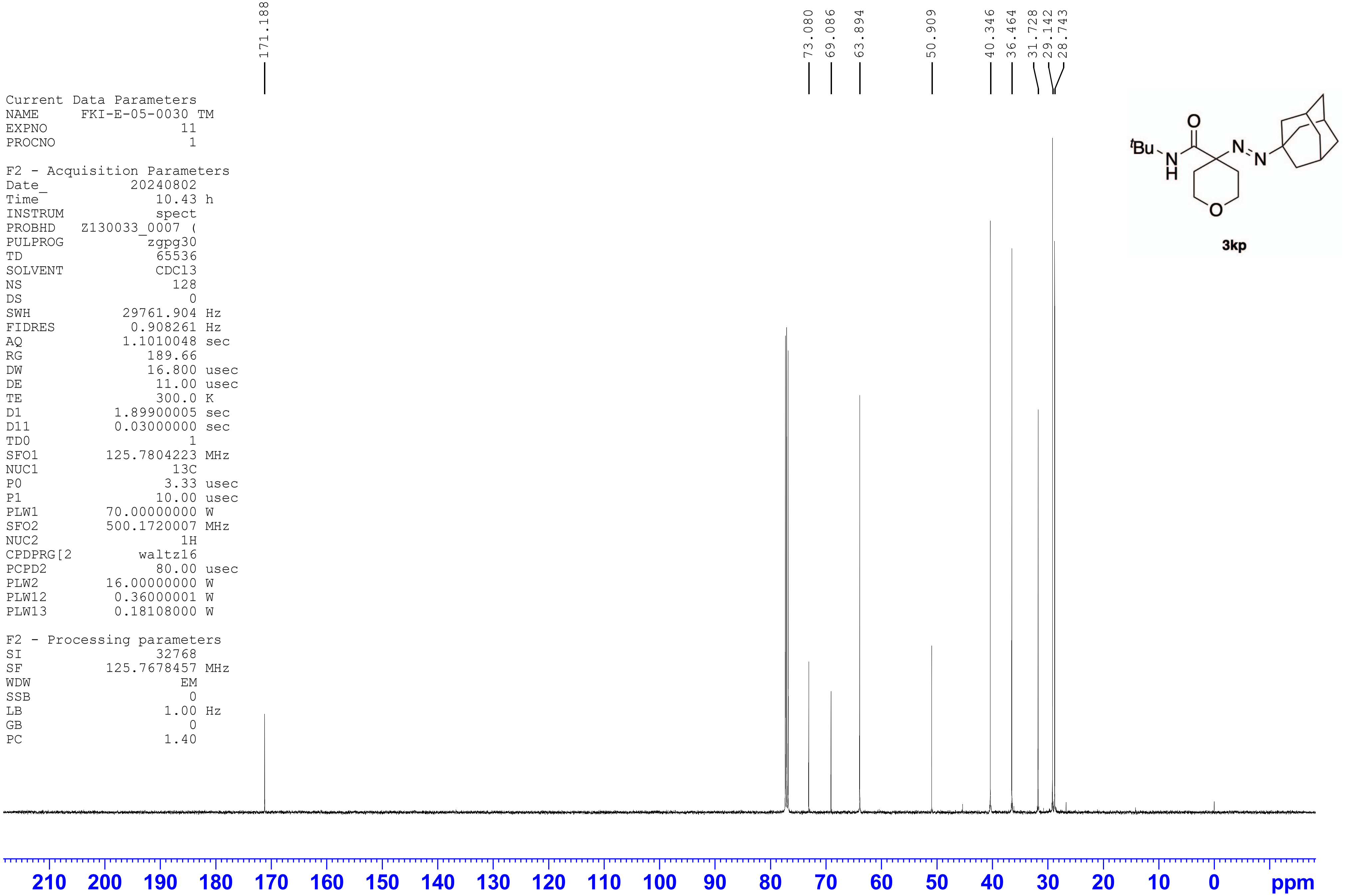
Current Data Parameters
NAME FKI-E-05-0030 TM
EXPNO 11
PROCNO 1
F2 - Acquisition Parameters
Date_ 20240802
Time_ 10.43 h
INSTRUM spect
PROBHD Z130033_0007 (
PULPROG zgpg30
TD 65536
SOLVENT CDCl3
NS 128
DS 0
SWH 29761.904 Hz
FIDRES 0.908261 Hz
AQ 1.1010048 sec
RG 189.66
DW 16.800 usec
DE 11.00 usec
TE 300.0 K
D1 1.89900005 sec
D11 0.03000000 sec
TD0 1
SFO1 125.7804223 MHz
NUC1 13C
P0 3.33 usec
P1 10.00 usec
PLW1 70.00000000 W
SFO2 500.1720007 MHz
NUC2 1H
CPDPRG[2 waltz16
PCPD2 80.00 usec
PLW2 16.00000000 W
PLW12 0.36000001 W
PLW13 0.18108000 W
F2 - Processing parameters
SI 32768
SF 125.7678457 MHz
WDW EM
SSB 0
LB 1.00 Hz
GB 0
PC 1.40
171.188
73.080
69.086
63.894
50.909
40.346
36.464
31.728
29.142
28.743
210 200 190 180 170 160 150 140 130 120 110 100 90 80 70 60 50 40 30 20 10 0 ppm
tBu
N
H
O
N
N
O

3kp

Current Data Parameters
NAME FKI-E-05-0021 TM MeOD
EXPNO 10
PROCNO 1

F2 - Acquisition Parameters
Date_ 20240716
Time 18.20 h
INSTRUM spect
PROBHD Z130033_0007 (
PULPROG zg30
TD 65536
SOLVENT MeOD
NS 1
DS 0
SWH 8012.820 Hz
FIDRES 0.244532 Hz
AQ 4.0894465 sec
RG 17.03
DW 62.400 usec
DE 10.00 usec
TE 300.0 K
D1 1.00000000 sec
TD0 1
SFO1 500.1730010 MHz
NUC1 1H
P0 4.00 usec
P1 12.00 usec
PLW1 16.00000000 W

F2 - Processing parameters
SI 65536
SF 500.1700609 MHz
WDW EM
SSB 0
LB 0.30 Hz
GB 0
PC 1.00

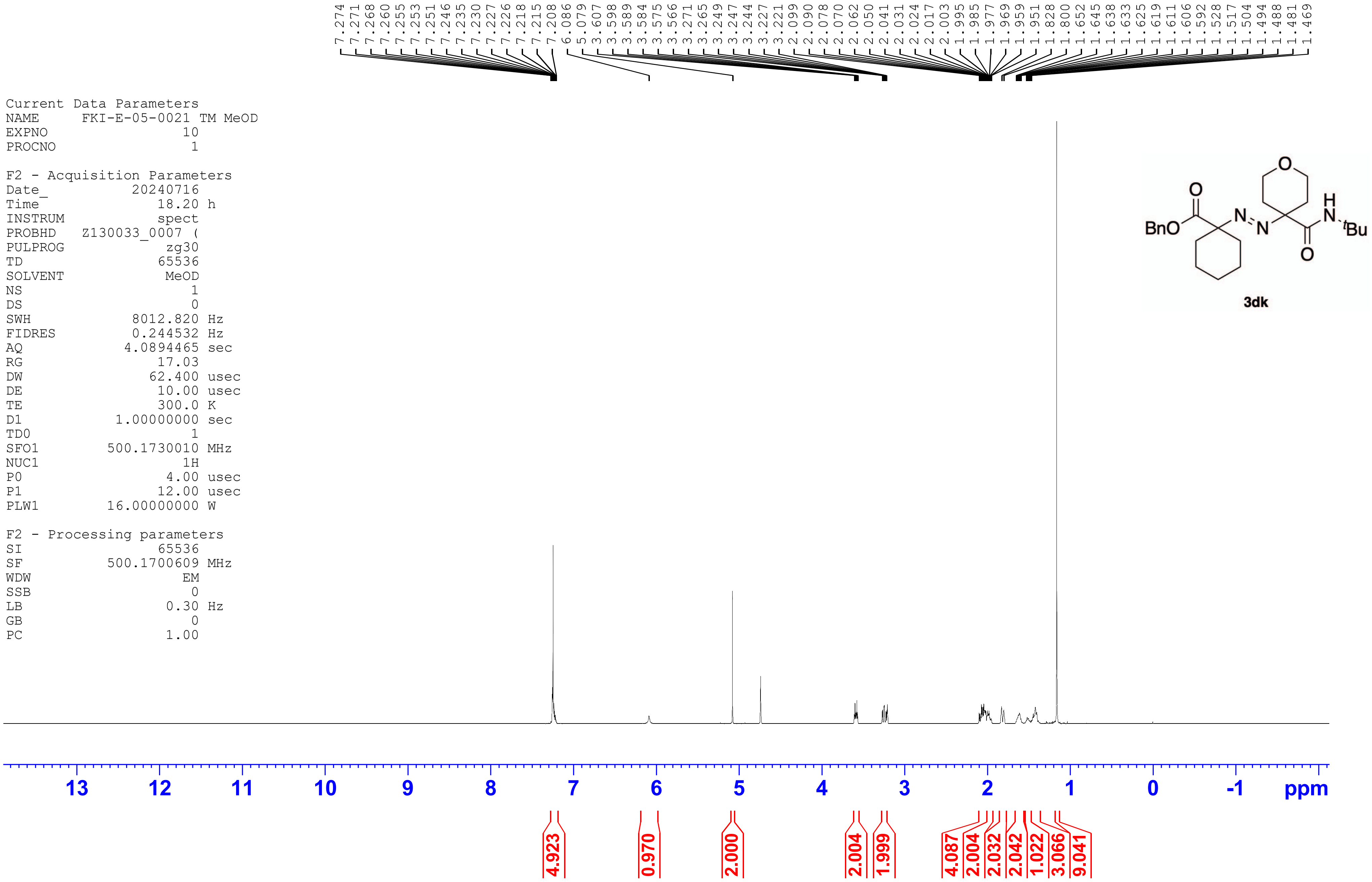

3dk

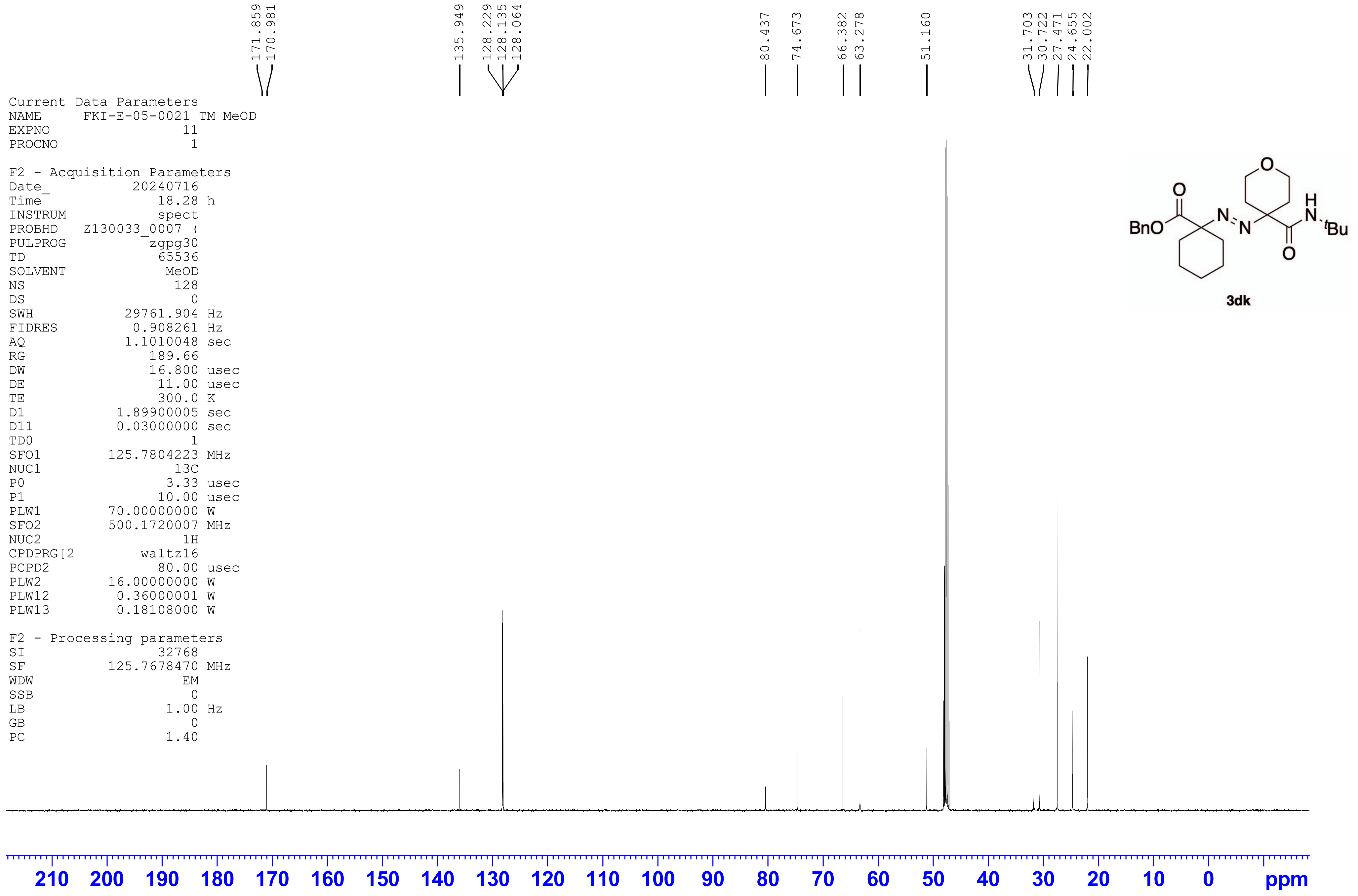
171.859
170.981
135.949
128.229
128.135
128.064
80.437
74.673
66.382
63.278
51.160
31.703
30.722
27.471
24.655
22.002
Current Data Parameters
NAME FKI-E-05-0021 TM MeOD
EXPNO 11
PROCNO 1
F2 - Acquisition Parameters
Date_ 20240716
Time_ 18.28 h
INSTRUM spect
PROBHD Z130033_0007 (
PULPROG zgpg30
TD 65536
SOLVENT MeOD
NS 128
DS 0
SWH 29761.904 Hz
FIDRES 0.908261 Hz
AQ 1.1010048 sec
RG 189.66
DW 16.800 usec
DE 11.00 usec
TE 300.0 K
D1 1.89900005 sec
D11 0.03000000 sec
TD0 1
SFO1 125.7804223 MHz
NUC1 13C
P0 3.33 usec
P1 10.00 usec
PLW1 70.00000000 W
SFO2 500.1720007 MHz
NUC2 1H
CPDPRG[2 waltz16
PCPD2 80.00 usec
PLW2 16.00000000 W
PLW12 0.36000001 W
PLW13 0.18108000 W
F2 - Processing parameters
SI 32768
SF 125.7678470 MHz
WDW EM
SSB 0
LB 1.00 Hz
GB 0
PC 1.40
BnO
O
N
N
O
H
N
tBu
O
3dk
210 200 190 180 170 160 150 140 130 120 110 100 90 80 70 60 50 40 30 20 10 0 ppm

Current Data Parameters
NAME FKI-E-05-0032 CD3OD
EXPNO 10
PROCNO 1

F2 - Acquisition Parameters
Date_ 20240804
Time_ 13.16 h
INSTRUM spect
PROBHD Z130033_0007 (
PULPROG zg30
TD 65536
SOLVENT MeOD
NS 1
DS 0
SWH 8012.820 Hz
FIDRES 0.244532 Hz
AQ 4.0894465 sec
RG 27.56
DW 62.400 usec
DE 10.00 usec
TE 300.0 K
D1 1.00000000 sec
TD0 1
SFO1 500.1730010 MHz
NUC1 1H
P0 4.00 usec
P1 12.00 usec
PLW1 16.00000000 W

F2 - Processing parameters
SI 65536
SF 500.1700605 MHz
WDW EM
SSB 0
LB 0.30 Hz
GB 0
PC 1.00

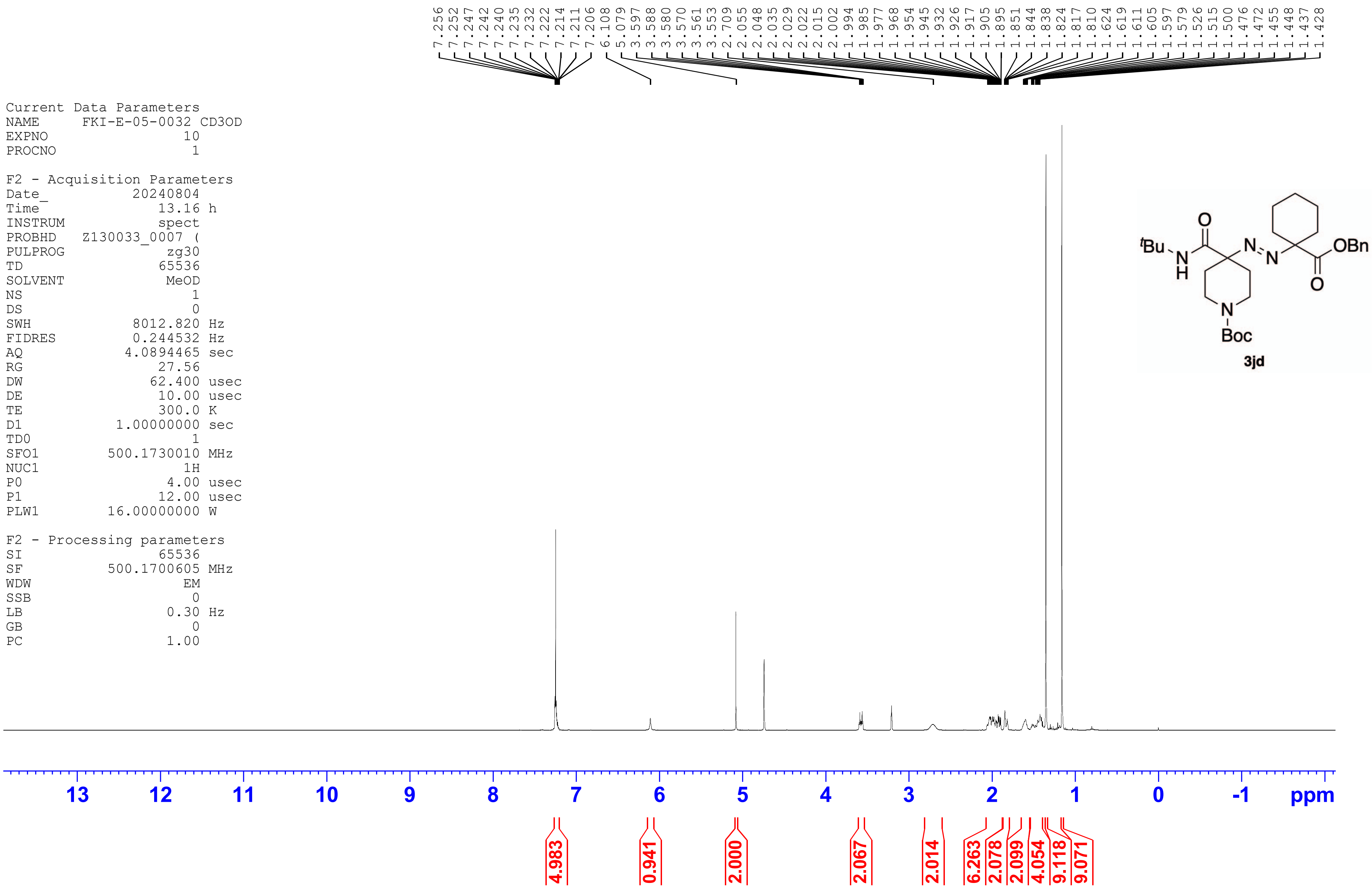

3jd

Current Data Parameters
NAME FKI-E-05-0032 CD3OD
EXPNO 11
PROCNO 1

F2 - Acquisition Parameters
Date_ 20240804
Time_ 13.24 h
INSTRUM spect
PROBHD Z130033_0007 (
PULPROG zgpg30
TD 65536
SOLVENT MeOD
NS 128
DS 0
SWH 29761.904 Hz
FIDRES 0.908261 Hz
AQ 1.1010048 sec
RG 189.66
DW 16.800 usec
DE 11.00 usec
TE 300.0 K
D1 1.89900005 sec
D11 0.03000000 sec
TD0 1
SFO1 125.7804223 MHz
NUC1 13C
P0 3.33 usec
P1 10.00 usec
PLW1 70.00000000 W
SFO2 500.1720007 MHz
NUC2 1H
CPDPRG[2 waltz16
PCPD2 80.00 usec
PLW2 16.00000000 W
PLW12 0.36000001 W
PLW13 0.18108000 W

F2 - Processing parameters
SI 32768
SF 125.7678470 MHz
WDW EM
SSB 0
LB 1.00 Hz
GB 0
PC 1.40

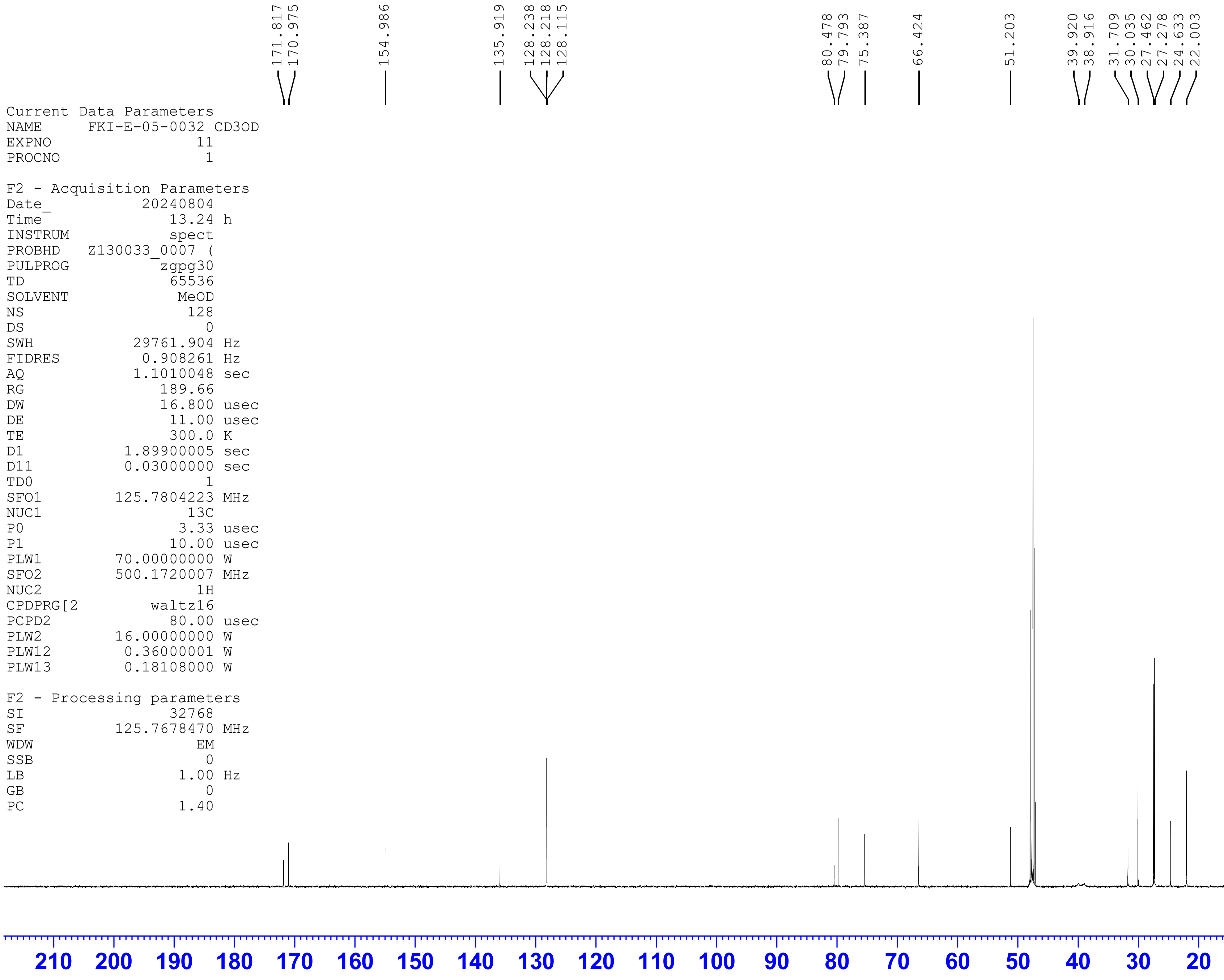

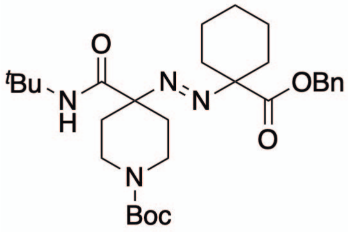

3jd

Current Data Parameters
NAME TKR-E-01-030_culumn_A
EXPNO 20
PROCNO 1

F2 - Acquisition Parameters
Date_ 20241009
Time 9.08 h
INSTRUM spect
PROBHD Z130033_0007 (
PULPROG zg30
TD 65536
SOLVENT CDCl3
NS 1
DS 0
SWH 8012.820 Hz
FIDRES 0.244532 Hz
AQ 4.0894465 sec
RG 31.29
DW 62.400 usec
DE 10.00 usec
TE 300.0 K
D1 1.00000000 sec
TD0 1
SFO1 500.1730010 MHz
NUC1 1H
P0 4.00 usec
P1 12.00 usec
PLW1 16.00000000 W

F2 - Processing parameters
SI 65536
SF 500.1700107 MHz
WDW EM
SSB 0
LB 0.30 Hz
GB 0
PC 1.00

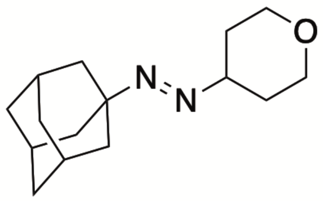

3py

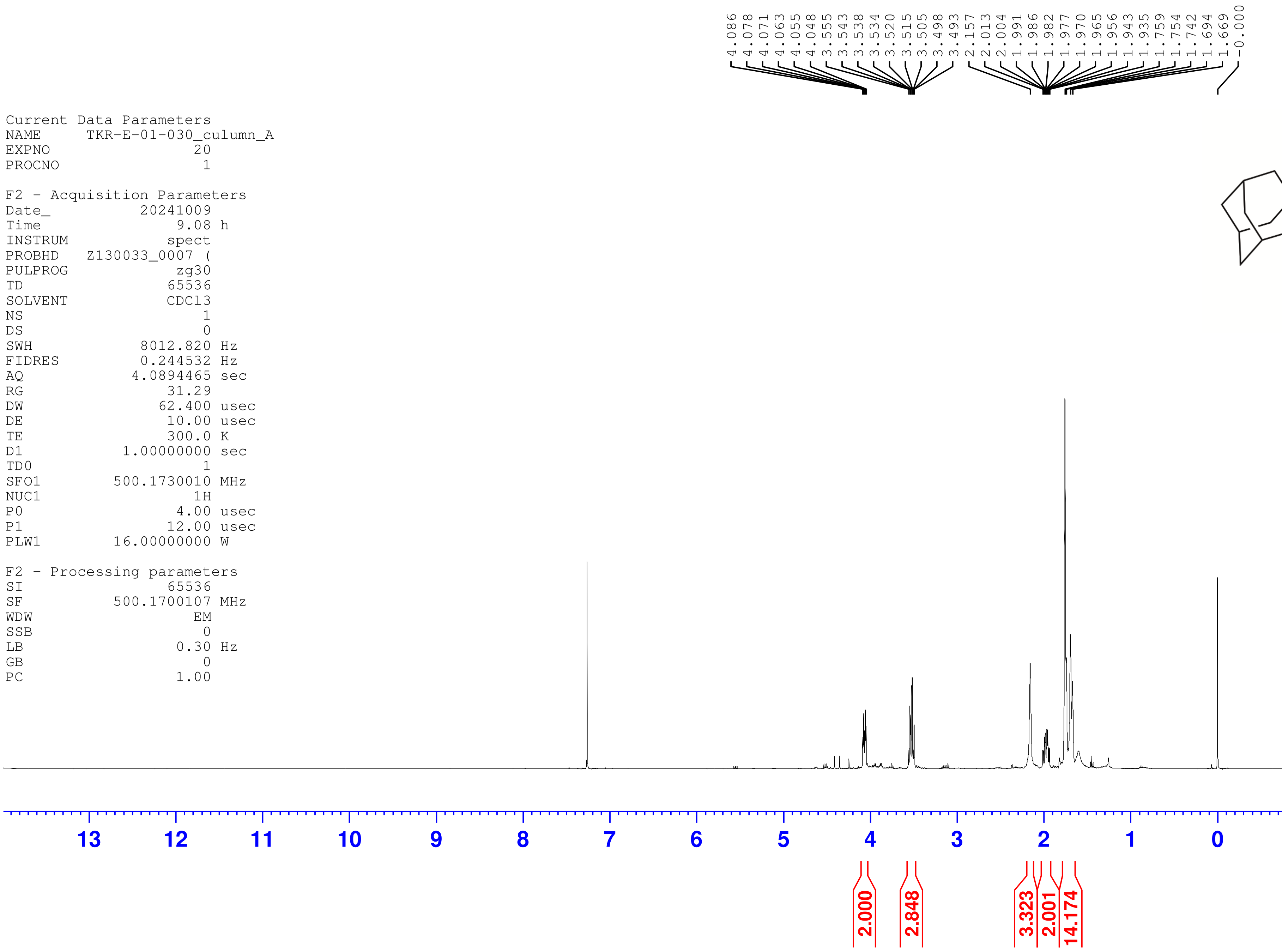

```
Current Data Parameters
NAME        TKR-E-01-030_culumn_A
EXPNO                    21
PROCNO                    1

F2 - Acquisition Parameters
Date_              20241009
Time                   9.16 h
INSTRUM               spect
PROBHD     Z130033_0007 (
PULPROG             zgpg30
TD                   65536
SOLVENT              CDCl3
NS                     128
DS                       0
SWH             29761.904 Hz
FIDRES           0.908261 Hz
AQ              1.1010048 sec
RG                  189.66
DW                  16.800 usec
DE                   11.00 usec
TE                   300.0 K
D1             1.89900005 sec
D11            0.03000000 sec
TD0                      1
SFO1         125.7804223 MHz
NUC1                   13C
P0                    3.33 usec
P1                   10.00 usec
PLW1          70.00000000 W
SFO2         500.1720007 MHz
NUC2                    1H
CPDPRG[2           waltz16
PCPD2                80.00 usec
PLW2          16.00000000 W
PLW12          0.36000001 W
PLW13          0.18108000 W

F2 - Processing parameters
SI                   32768
SF           125.7678465 MHz
WDW                     EM
SSB                      0
LB                    1.00 Hz
GB                       0
PC                    1.40
```

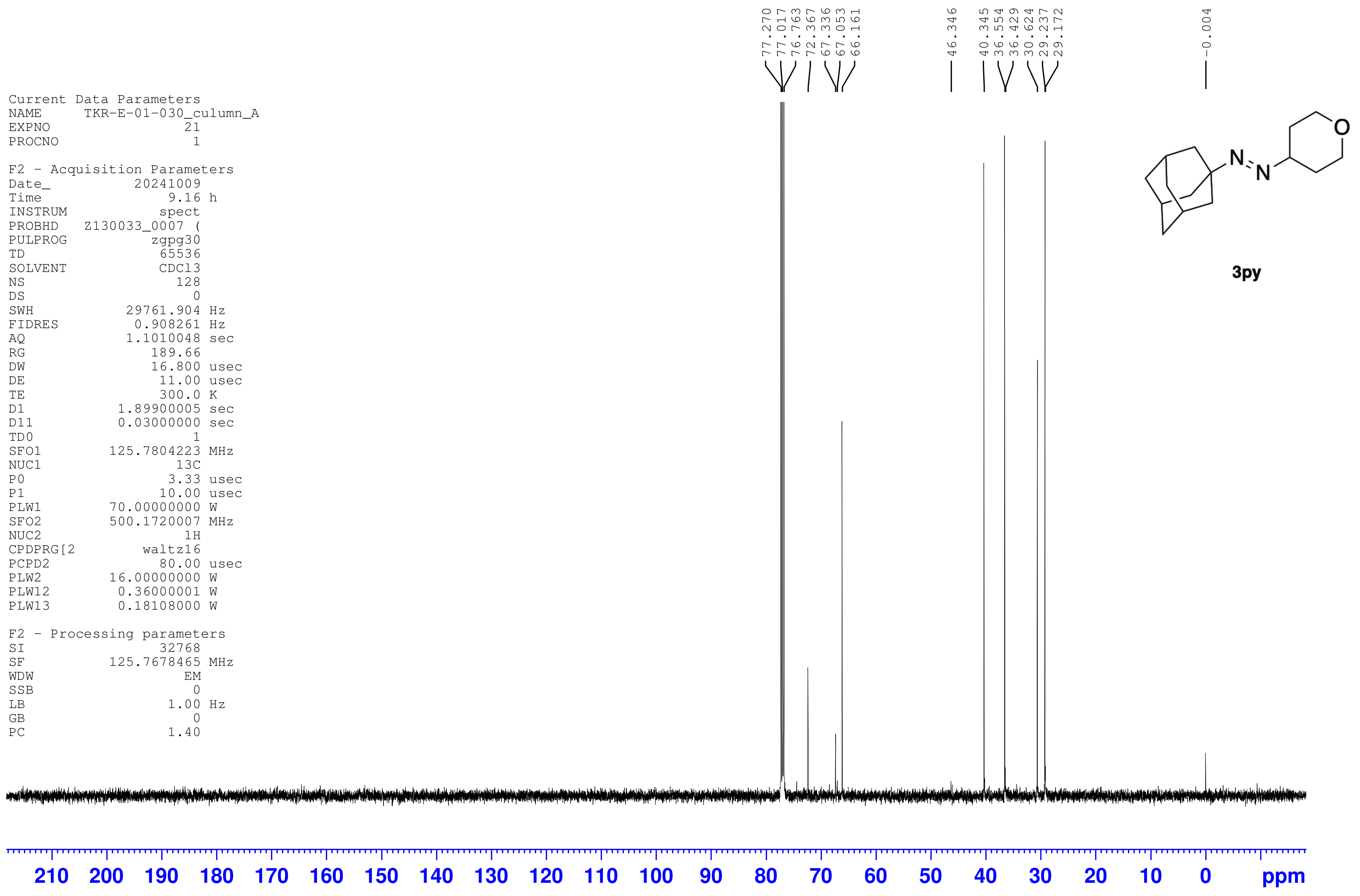

3py

```
Current Data Parameters
NAME          tsu-e3-394b
EXPNO                  10
PROCNO                  1

F2 - Acquisition Parameters
Date_            20230515
Time                14.07 h
INSTRUM             spect
PROBHD   Z119470_0344 (
PULPROG              zg30
TD                  65536
SOLVENT             CDCl3
NS                      1
DS                      0
SWH              8012.820 Hz
FIDRES           0.244532 Hz
AQ              4.0894465 sec
RG                 116.65
DW                 62.400 usec
DE                   6.50 usec
TE                  298.0 K
D1             1.00000000 sec
TD0                     1
SFO1          500.1730010 MHz
NUC1                   1H
P0                   4.83 usec
P1                  14.50 usec
PLW1          10.80000019 W

F2 - Processing parameters
SI                  65536
SF            500.1700137 MHz
WDW                    EM
SSB                     0
LB                   0.30 Hz
GB                      0
PC                   1.00
```

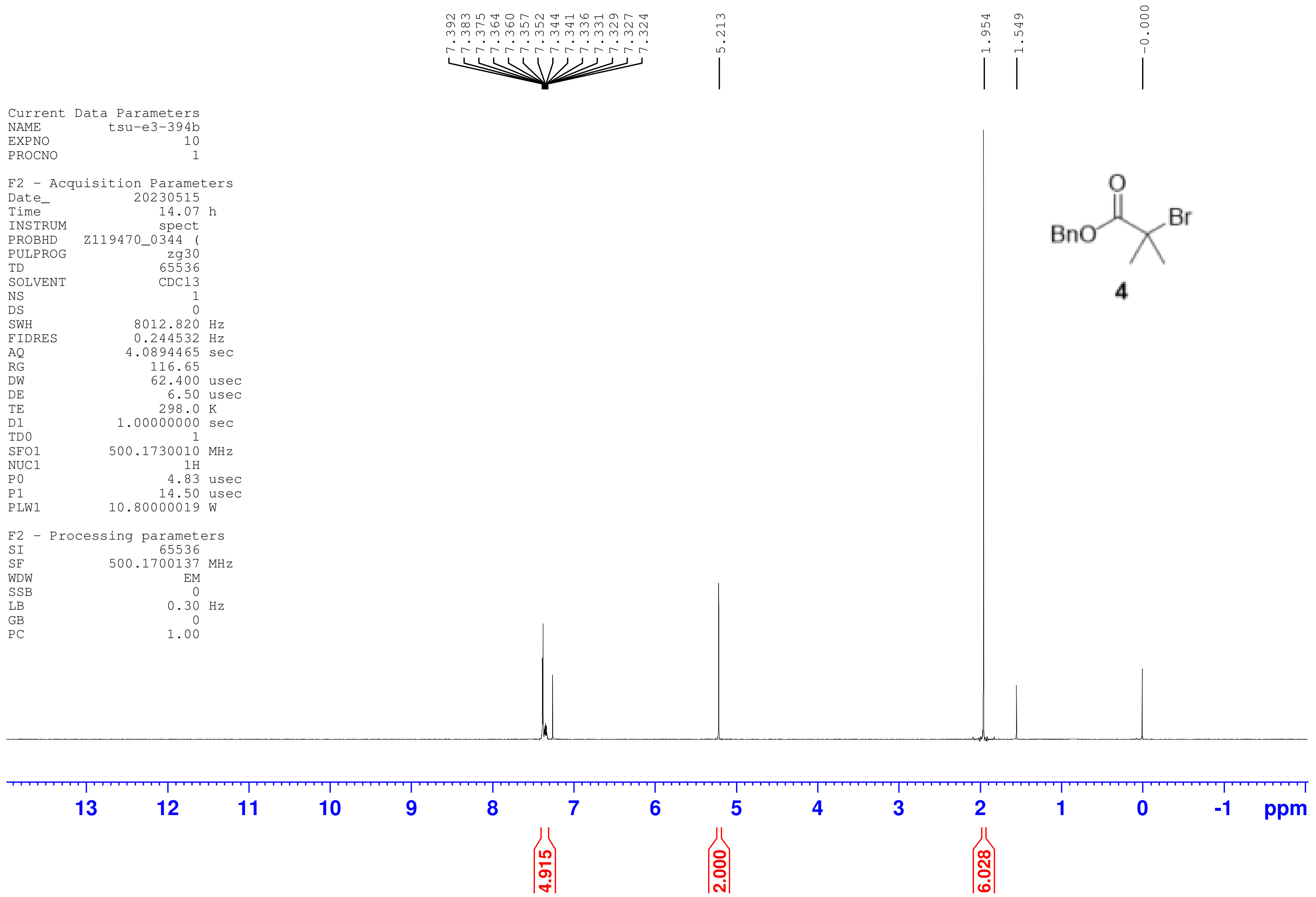

4

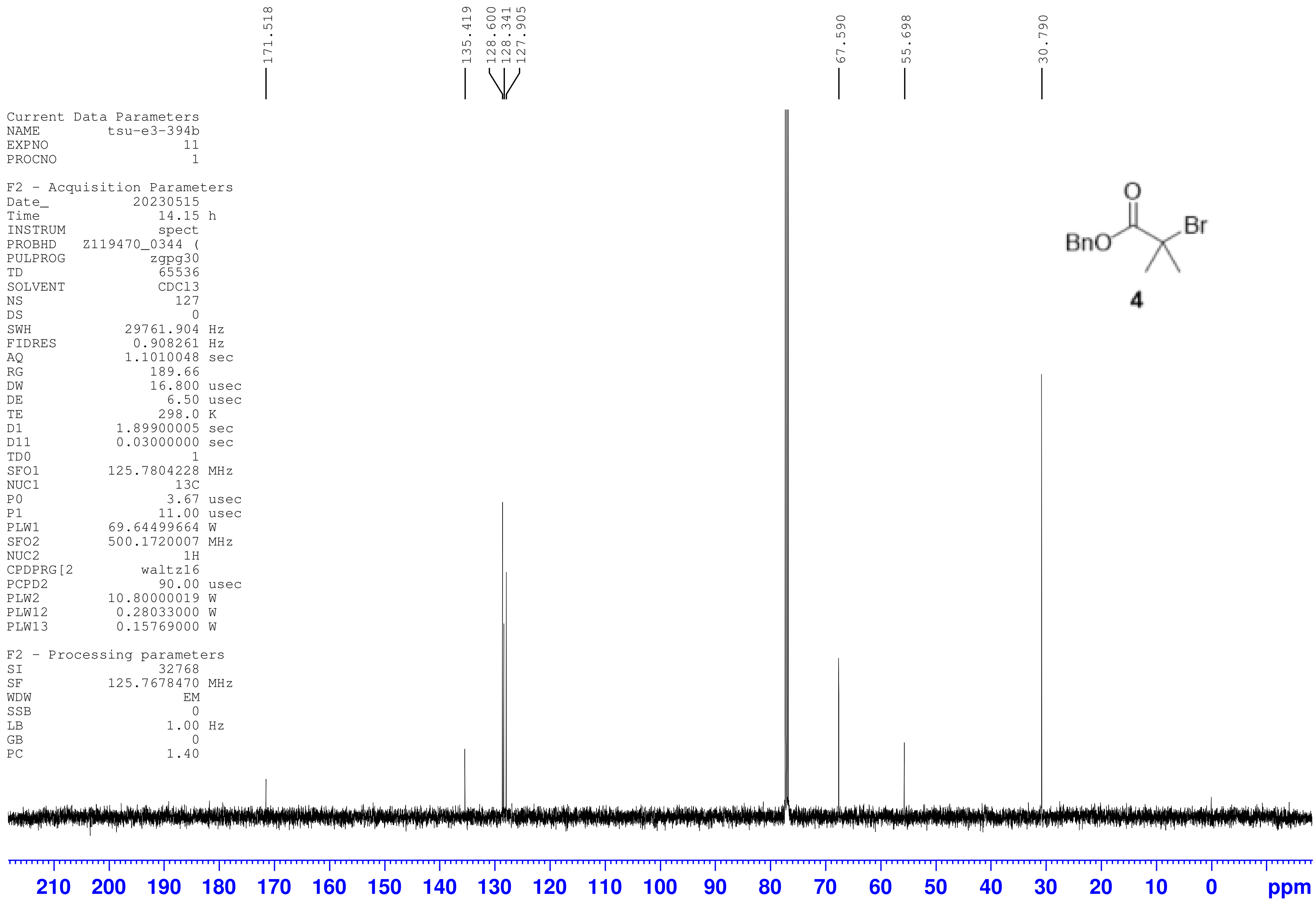
171.518
135.419
128.600
128.341
127.905
67.590
55.698
30.790
Current Data Parameters
NAME tsu-e3-394b
EXPNO 11
PROCNO 1
F2 - Acquisition Parameters
Date_ 20230515
Time 14.15 h
INSTRUM spect
PROBHD Z119470_0344 (
PULPROG zgpg30
TD 65536
SOLVENT CDCl3
NS 127
DS 0
SWH 29761.904 Hz
FIDRES 0.908261 Hz
AQ 1.1010048 sec
RG 189.66
DW 16.800 usec
DE 6.50 usec
TE 298.0 K
D1 1.89900005 sec
D11 0.03000000 sec
TD0 1
SFO1 125.7804228 MHz
NUC1 13C
P0 3.67 usec
P1 11.00 usec
PLW1 69.64499664 W
SFO2 500.1720007 MHz
NUC2 1H
CPDPRG[2 waltz16
PCPD2 90.00 usec
PLW2 10.80000019 W
PLW12 0.28033000 W
PLW13 0.15769000 W
F2 - Processing parameters
SI 32768
SF 125.7678470 MHz
WDW EM
SSB 0
LB 1.00 Hz
GB 0
PC 1.40
BnO
O
Br
4
210 200 190 180 170 160 150 140 130 120 110 100 90 80 70 60 50 40 30 20 10 0 ppm

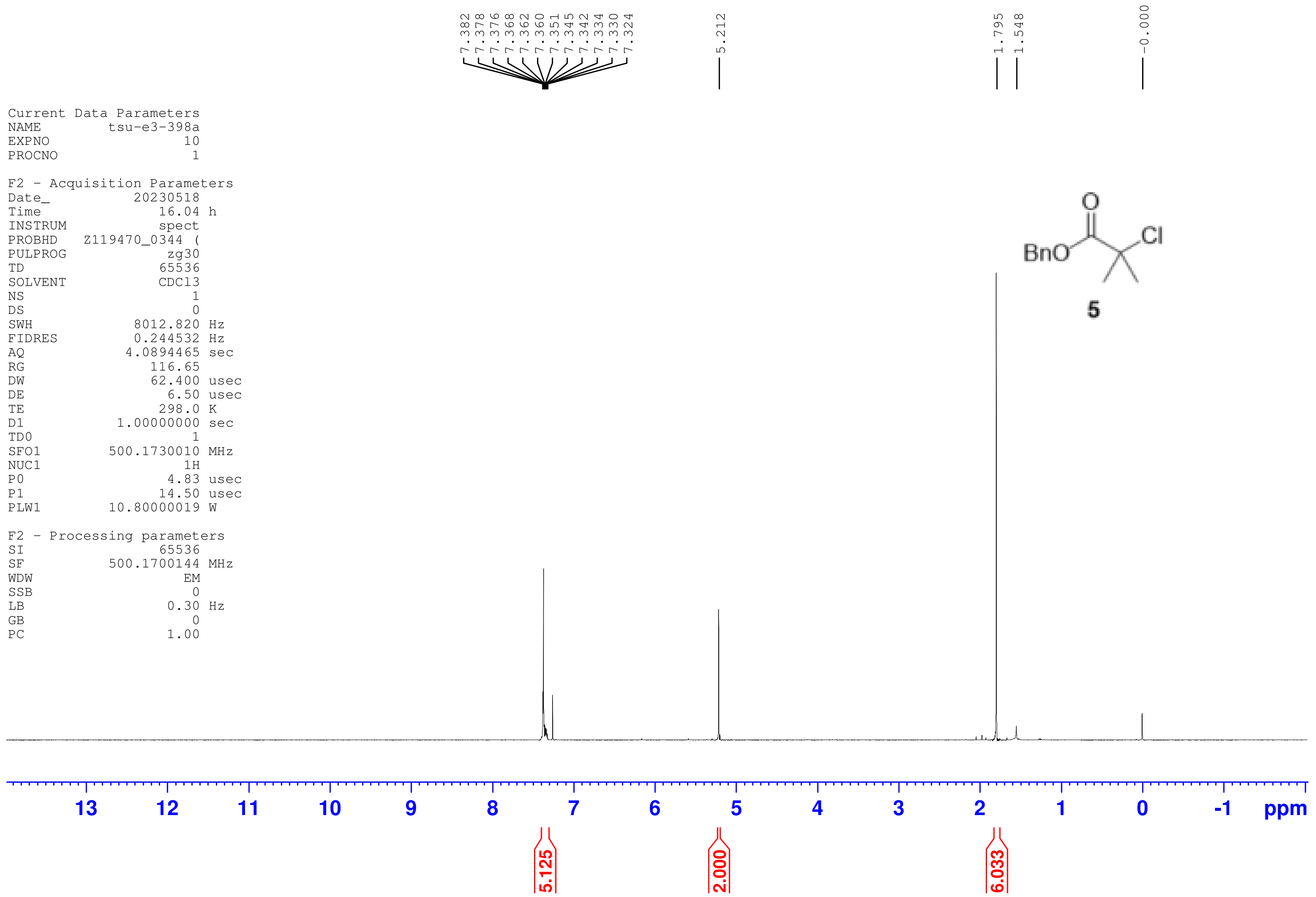
7.382
7.378
7.376
7.368
7.362
7.360
7.351
7.345
7.342
7.334
7.330
7.324
5.212
1.795
1.548
-0.000
Current Data Parameters
NAME tsu-e3-398a
EXPNO 10
PROCNO 1
F2 - Acquisition Parameters
Date_ 20230518
Time 16.04 h
INSTRUM spect
PROBHD Z119470_0344 (
PULPROG zg30
TD 65536
SOLVENT CDCl3
NS 1
DS 0
SWH 8012.820 Hz
FIDRES 0.244532 Hz
AQ 4.0894465 sec
RG 116.65
DW 62.400 usec
DE 6.50 usec
TE 298.0 K
D1 1.00000000 sec
TD0 1
SFO1 500.1730010 MHz
NUC1 1H
P0 4.83 usec
P1 14.50 usec
PLW1 10.80000019 W
F2 - Processing parameters
SI 65536
SF 500.1700144 MHz
WDW EM
SSB 0
LB 0.30 Hz
GB 0
PC 1.00
13
12
11
10
9
8
7
6
5
4
3
2
1
0
-1
ppm
5.125
2.000
6.033
O
BnO
Cl

5

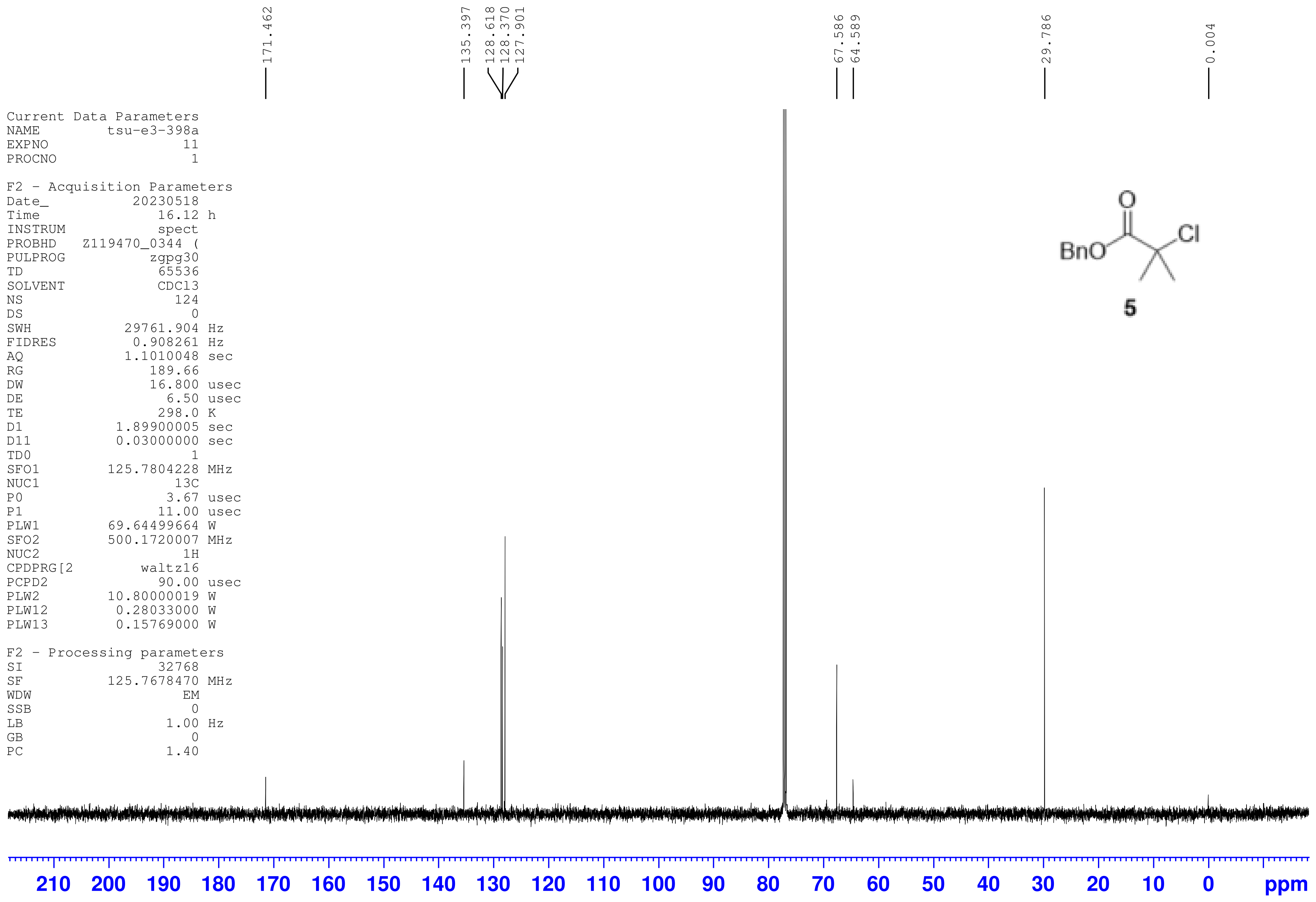
171.462
135.397
128.618
128.370
127.901
67.586
64.589
29.786
0.004
Current Data Parameters
NAME tsu-e3-398a
EXPNO 11
PROCNO 1
F2 - Acquisition Parameters
Date_ 20230518
Time 16.12 h
INSTRUM spect
PROBHD Z119470_0344 (
PULPROG zgpg30
TD 65536
SOLVENT CDCl3
NS 124
DS 0
SWH 29761.904 Hz
FIDRES 0.908261 Hz
AQ 1.1010048 sec
RG 189.66
DW 16.800 usec
DE 6.50 usec
TE 298.0 K
D1 1.89900005 sec
D11 0.03000000 sec
TD0 1
SFO1 125.7804228 MHz
NUC1 13C
P0 3.67 usec
P1 11.00 usec
PLW1 69.64499664 W
SFO2 500.1720007 MHz
NUC2 1H
CPDPRG[2 waltz16
PCPD2 90.00 usec
PLW2 10.80000019 W
PLW12 0.28033000 W
PLW13 0.15769000 W
F2 - Processing parameters
SI 32768
SF 125.7678470 MHz
WDW EM
SSB 0
LB 1.00 Hz
GB 0
PC 1.40
BnO
O
Cl
5
210 200 190 180 170 160 150 140 130 120 110 100 90 80 70 60 50 40 30 20 10 0 ppm

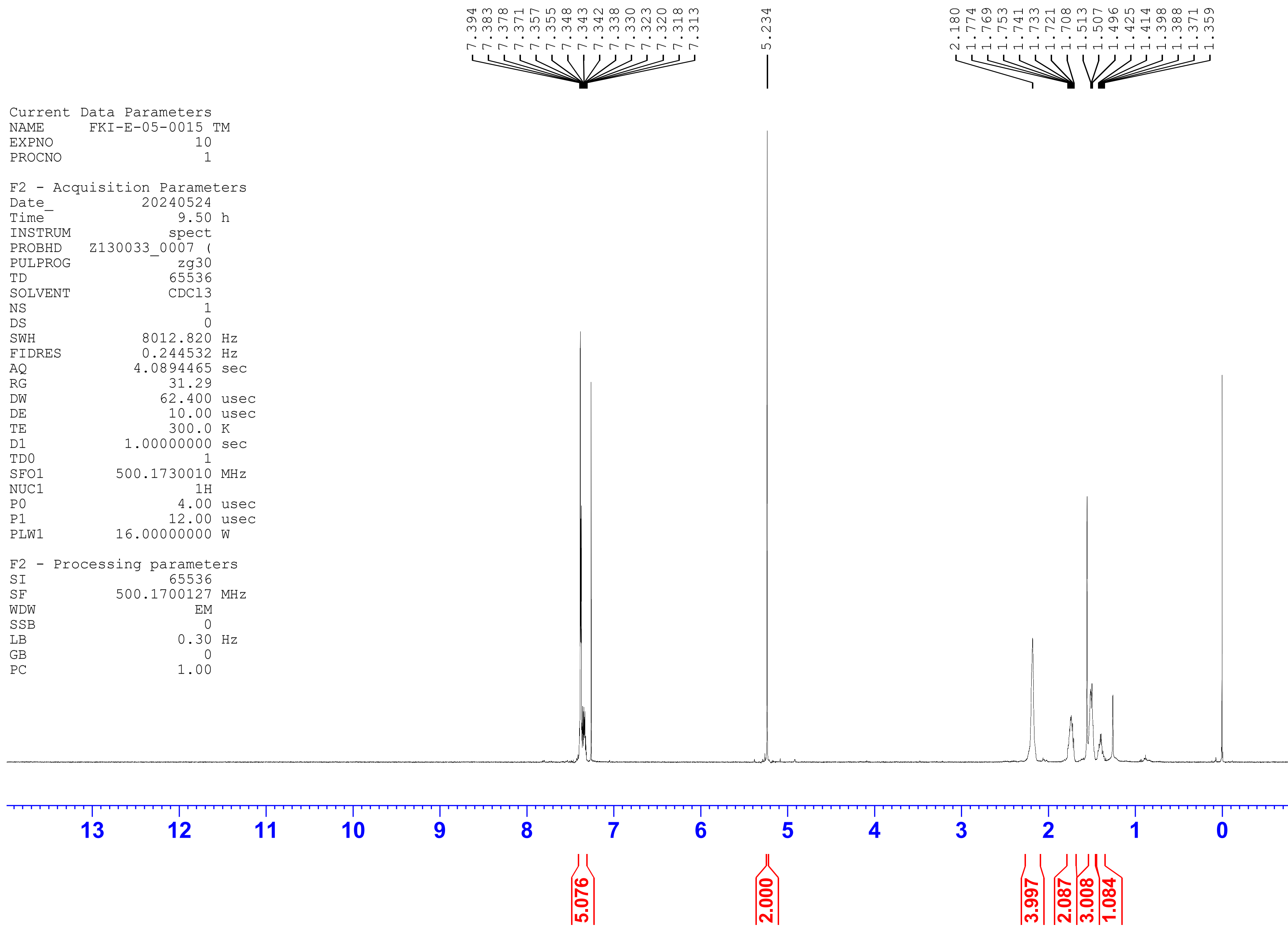
7.394
7.383
7.378
7.371
7.357
7.355
7.348
7.343
7.342
7.338
7.330
7.323
7.320
7.318
7.313
5.234
2.180
1.774
1.769
1.753
1.741
1.733
1.721
1.708
1.513
1.507
1.496
1.425
1.414
1.398
1.388
1.371
1.359
Current Data Parameters
NAME FKI-E-05-0015 TM
EXPNO 10
PROCNO 1
F2 - Acquisition Parameters
Date_ 20240524
Time 9.50 h
INSTRUM spect
PROBHD Z130033_0007 (
PULPROG zg30
TD 65536
SOLVENT CDCl3
NS 1
DS 0
SWH 8012.820 Hz
FIDRES 0.244532 Hz
AQ 4.0894465 sec
RG 31.29
DW 62.400 usec
DE 10.00 usec
TE 300.0 K
D1 1.00000000 sec
TD0 1
SFO1 500.1730010 MHz
NUC1 1H
P0 4.00 usec
P1 12.00 usec
PLW1 16.00000000 W
F2 - Processing parameters
SI 65536
SF 500.1700127 MHz
WDW EM
SSB 0
LB 0.30 Hz
GB 0
PC 1.00
13
12
11
10
9
8
7
6
5
4
3
2
1
0
-1
ppm
5.076
2.000
3.997
2.087
3.008
1.084

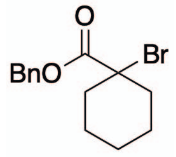
O
BnO
Br

6

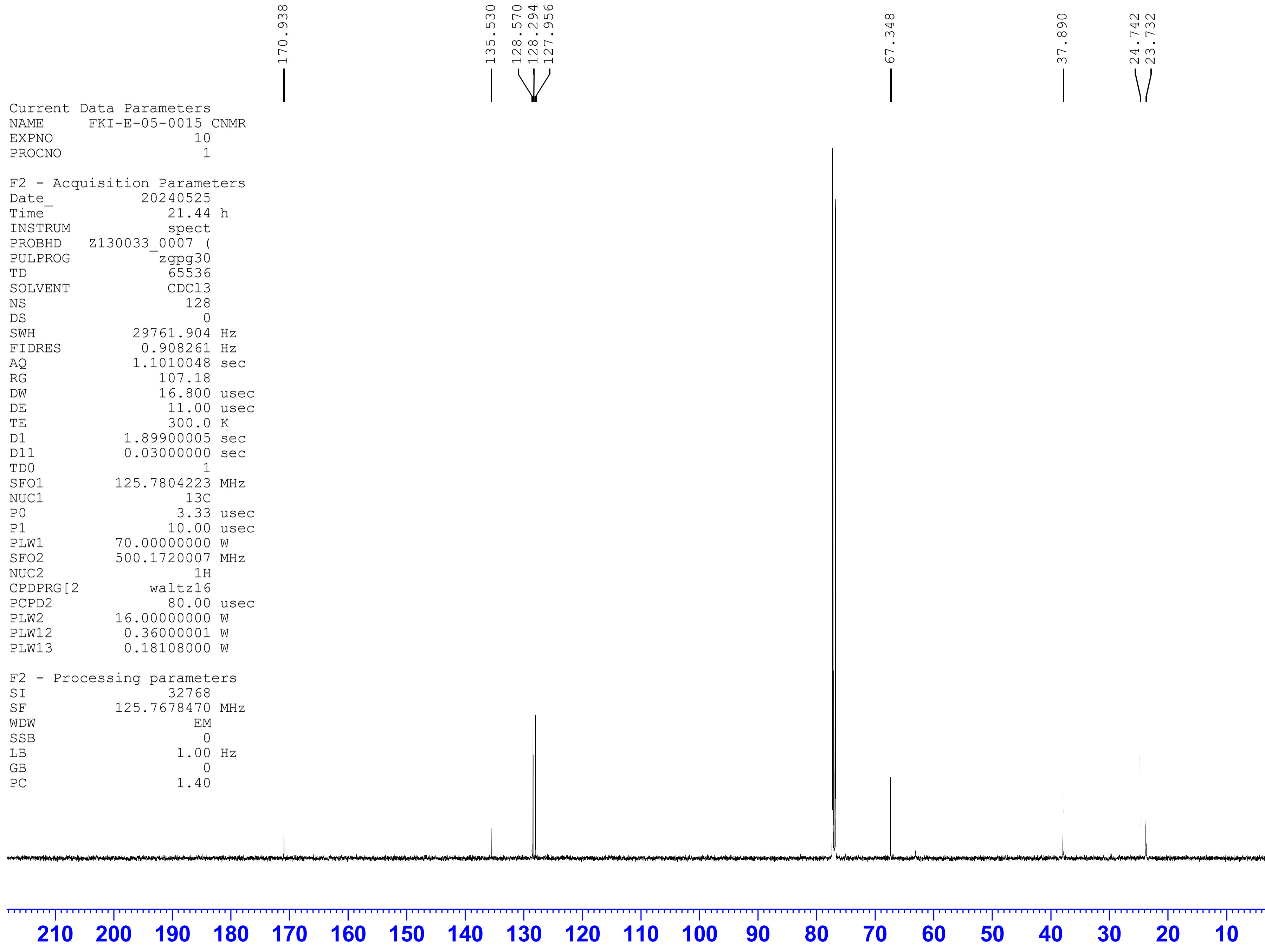
170.938
135.530
128.570
128.294
127.956
67.348
37.890
24.742
23.732
Current Data Parameters
NAME FKI-E-05-0015 CNMR
EXPNO 10
PROCNO 1
F2 - Acquisition Parameters
Date_ 20240525
Time 21.44 h
INSTRUM spect
PROBHD Z130033_0007 (
PULPROG zgpg30
TD 65536
SOLVENT CDCl3
NS 128
DS 0
SWH 29761.904 Hz
FIDRES 0.908261 Hz
AQ 1.1010048 sec
RG 107.18
DW 16.800 usec
DE 11.00 usec
TE 300.0 K
D1 1.89900005 sec
D11 0.03000000 sec
TD0 1
SFO1 125.7804223 MHz
NUC1 13C
P0 3.33 usec
P1 10.00 usec
PLW1 70.00000000 W
SFO2 500.1720007 MHz
NUC2 1H
CPDPRG[2 waltz16
PCPD2 80.00 usec
PLW2 16.00000000 W
PLW12 0.36000001 W
PLW13 0.18108000 W
F2 - Processing parameters
SI 32768
SF 125.7678470 MHz
WDW EM
SSB 0
LB 1.00 Hz
GB 0
PC 1.40
210 200 190 180 170 160 150 140 130 120 110 100 90 80 70 60 50 40 30 20 10 0 ppm

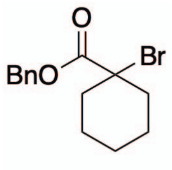
O
BnO
Br

6

```
Current Data Parameters
NAME      FKI-E-05-0036 TM
EXPNO                   10
PROCNO                   1

F2 - Acquisition Parameters
Date_             20240815
Time                 15.37 h
INSTRUM              spect
PROBHD    Z130033_0007 (
PULPROG               zg30
TD                   65536
SOLVENT              CDCl3
NS                       1
DS                       0
SWH               8012.820 Hz
FIDRES            0.244532 Hz
AQ               4.0894465 sec
RG                   31.29
DW                  62.400 usec
DE                   10.00 usec
TE                   300.1 K
D1              1.00000000 sec
TD0                      1
SFO1           500.1730010 MHz
NUC1                    1H
P0                    4.00 usec
P1                   12.00 usec
PLW1           16.00000000 W

F2 - Processing parameters
SI                   65536
SF             500.1700114 MHz
WDW                     EM
SSB                      0
LB                    0.30 Hz
GB                       0
PC                    1.00
```

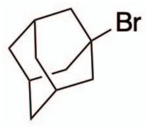

7

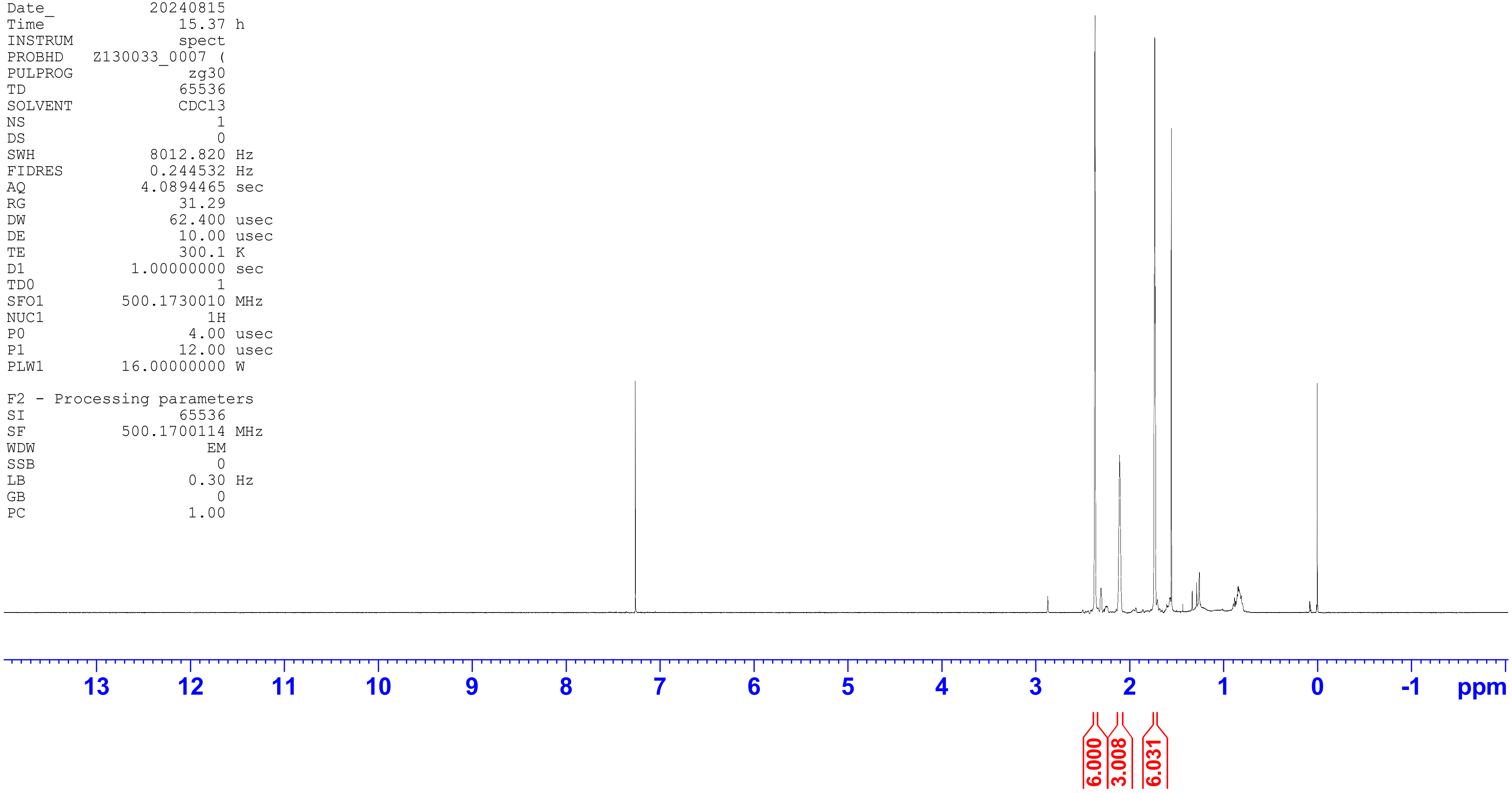

Current Data Parameters		
NAME	FKI-E-05-0036 TM	
EXPNO	11	
PROCNO	1	
F2 - Acquisition Parameters		
Date_	20240815	
Time_	15.45	h
INSTRUM	spect	
PROBHD	Z130033_0007 (	
PULPROG	zgpg30	
TD	65536	
SOLVENT	CDCl3	
NS	128	
DS	0	
SWH	29761.904	Hz
FIDRES	0.908261	Hz
AQ	1.1010048	sec
RG	189.66	
DW	16.800	usec
DE	11.00	usec
TE	300.0	K
D1	1.89900005	sec
D11	0.03000000	sec
TD0	1	
SFO1	125.7804223	MHz
NUC1	13C	
P0	3.33	usec
P1	10.00	usec
PLW1	70.00000000	W
SFO2	500.1720007	MHz
NUC2	1H	
CPDPRG[2	waltz16	
PCPD2	80.00	usec
PLW2	16.00000000	W
PLW12	0.36000001	W
PLW13	0.18108000	W
F2 - Processing parameters		
SI	32768	
SF	125.7678470	MHz
WDW	EM	
SSB	0	
LB	1.00	Hz
GB	0	
PC	1.40	

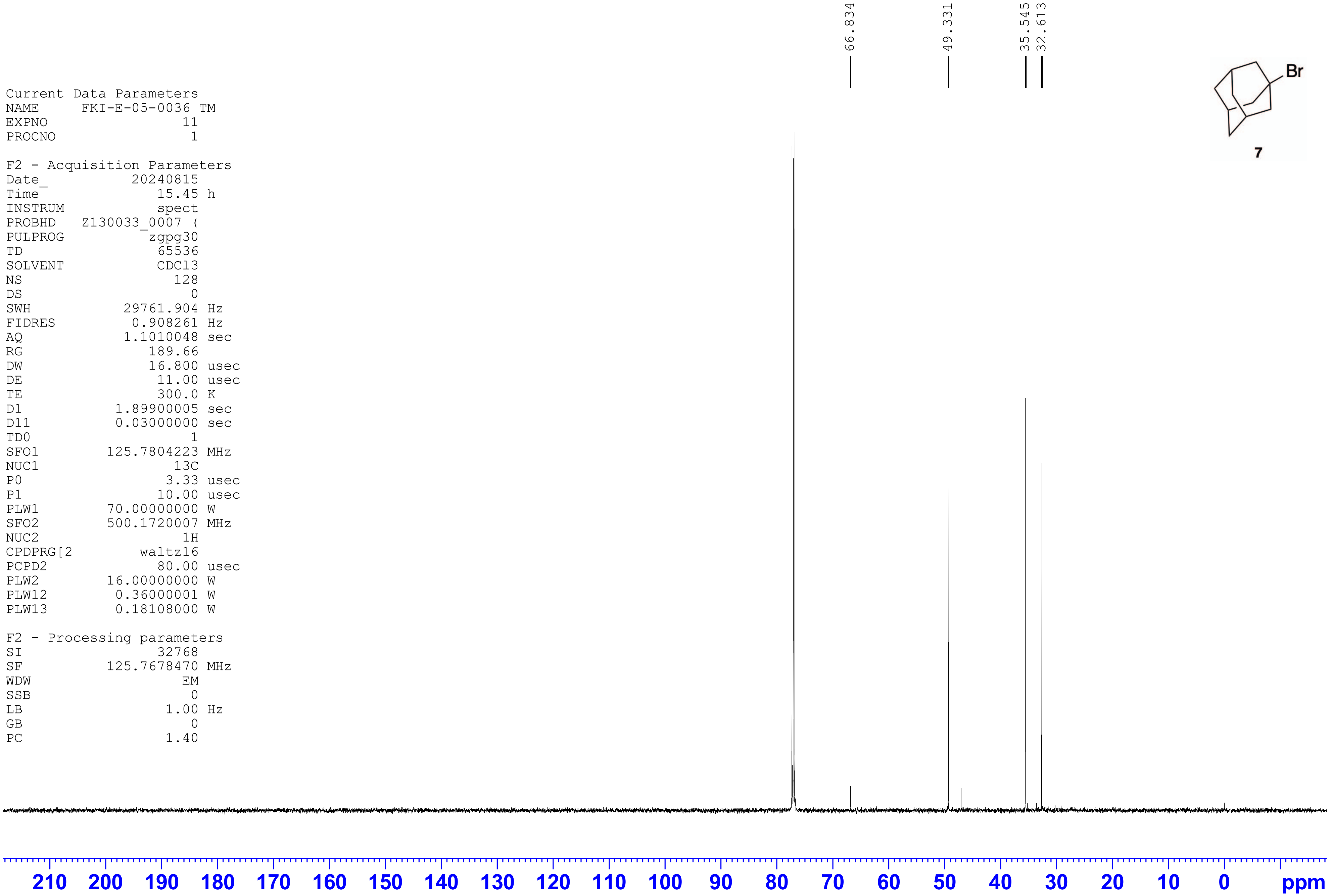

7

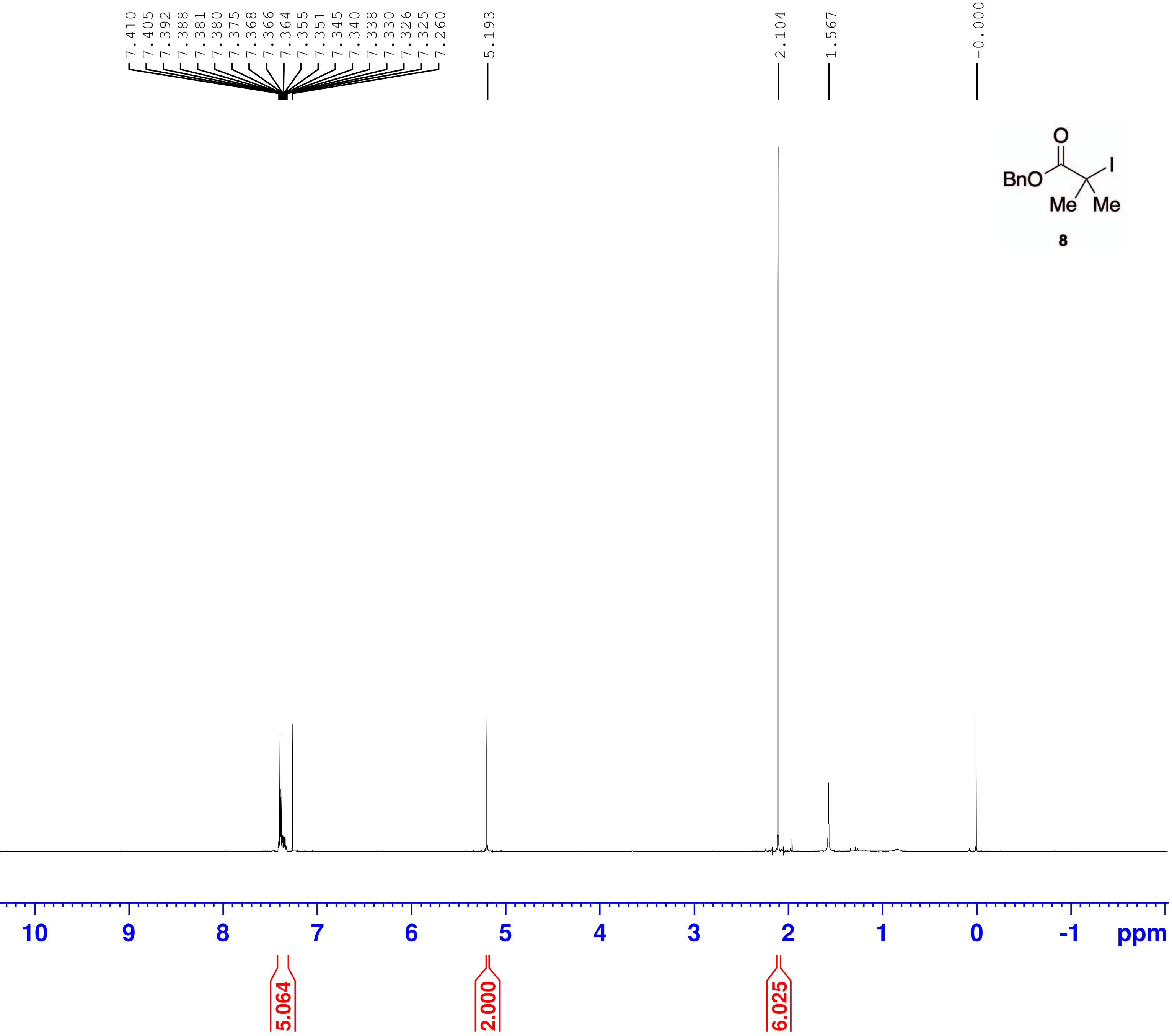

Current Data Parameters
NAME tsu-e3-453
EXPNO 10
PROCNO 1

F2 - Acquisition Parameters
Date_ 20230616
Time 17.04 h
INSTRUM spect
PROBHD Z119470_0344 (
PULPROG zg30
TD 65536
SOLVENT CDCl3
NS 1
DS 0
SWH 8012.820 Hz
FIDRES 0.244532 Hz
AQ 4.0894465 sec
RG 130.52
DW 62.400 usec
DE 6.50 usec
TE 298.0 K
D1 1.00000000 sec
TD0 1
SFO1 500.1730010 MHz
NUC1 1H
P0 4.83 usec
P1 14.50 usec
PLW1 10.80000019 W

F2 - Processing parameters
SI 65536
SF 500.1700128 MHz
WDW EM
SSB 0
LB 0.30 Hz
GB 0
PC 1.00

8

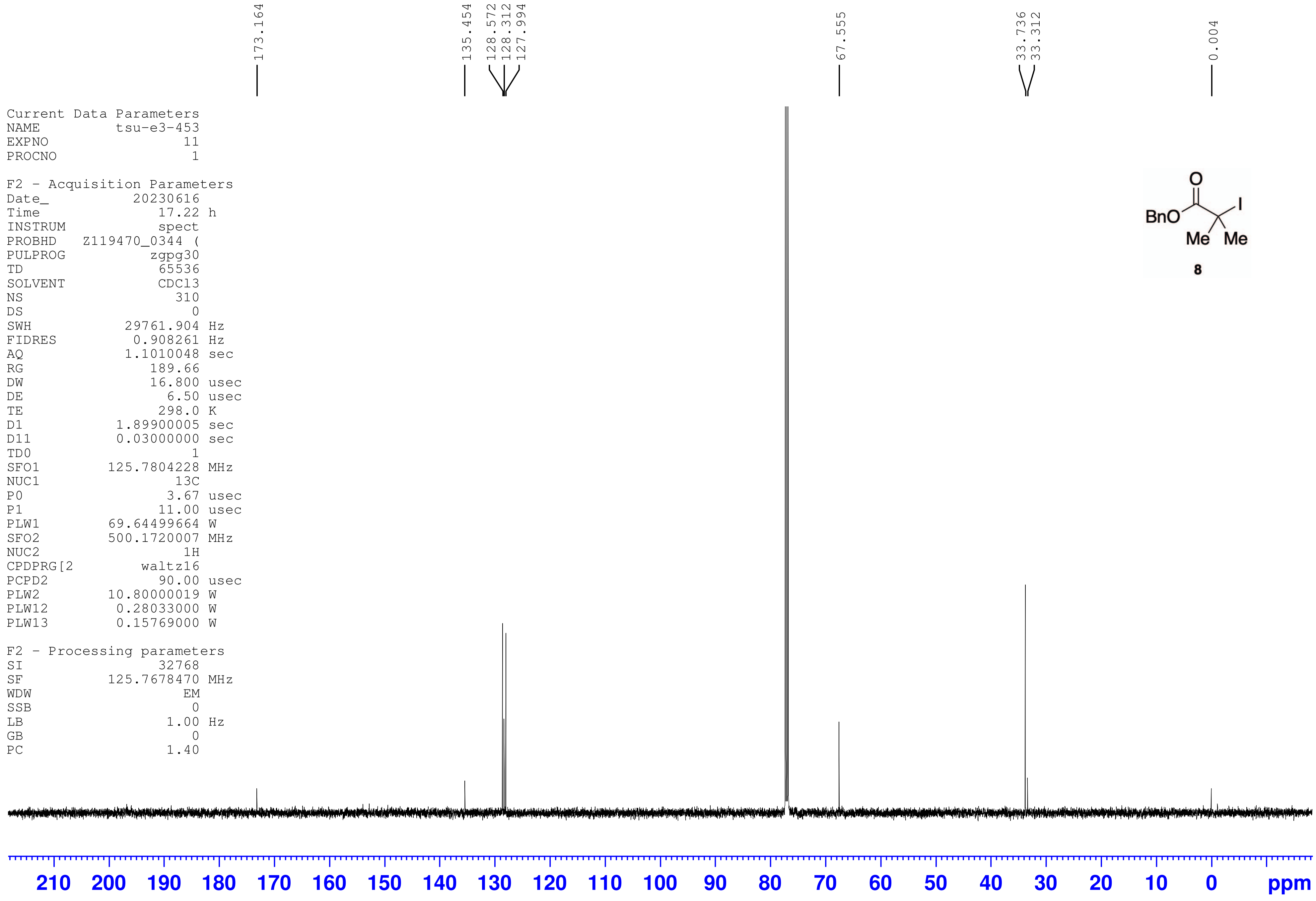
173.164
135.454
128.572
128.312
127.994
67.555
33.736
33.312
0.004
Current Data Parameters
NAME tsu-e3-453
EXPNO 11
PROCNO 1
F2 - Acquisition Parameters
Date_ 20230616
Time 17.22 h
INSTRUM spect
PROBHD Z119470_0344 (
PULPROG zgpg30
TD 65536
SOLVENT CDCl3
NS 310
DS 0
SWH 29761.904 Hz
FIDRES 0.908261 Hz
AQ 1.1010048 sec
RG 189.66
DW 16.800 usec
DE 6.50 usec
TE 298.0 K
D1 1.89900005 sec
D11 0.03000000 sec
TD0 1
SFO1 125.7804228 MHz
NUC1 13C
P0 3.67 usec
P1 11.00 usec
PLW1 69.64499664 W
SFO2 500.1720007 MHz
NUC2 1H
CPDPRG[2 waltz16
PCPD2 90.00 usec
PLW2 10.80000019 W
PLW12 0.28033000 W
PLW13 0.15769000 W
F2 - Processing parameters
SI 32768
SF 125.7678470 MHz
WDW EM
SSB 0
LB 1.00 Hz
GB 0
PC 1.40
210 200 190 180 170 160 150 140 130 120 110 100 90 80 70 60 50 40 30 20 10 0 ppm
O
BnO
I
Me
Me

8

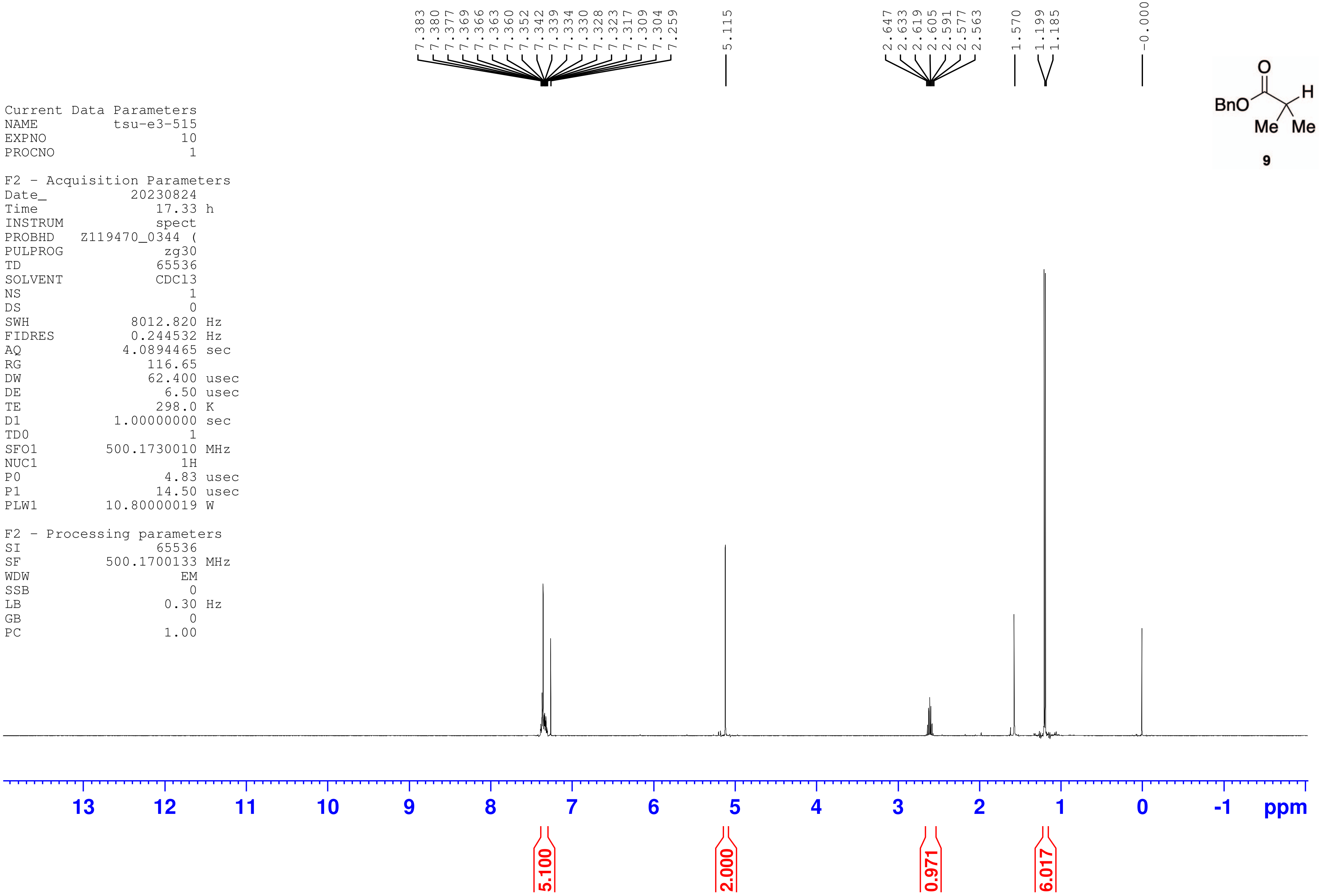
Current Data Parameters
NAME tsu-e3-515
EXPNO 10
PROCNO 1
F2 - Acquisition Parameters
Date_ 20230824
Time 17.33 h
INSTRUM spect
PROBHD Z119470_0344 (
PULPROG zg30
TD 65536
SOLVENT CDCl3
NS 1
DS 0
SWH 8012.820 Hz
FIDRES 0.244532 Hz
AQ 4.0894465 sec
RG 116.65
DW 62.400 usec
DE 6.50 usec
TE 298.0 K
D1 1.00000000 sec
TD0 1
SFO1 500.1730010 MHz
NUC1 1H
P0 4.83 usec
P1 14.50 usec
PLW1 10.80000019 W
F2 - Processing parameters
SI 65536
SF 500.1700133 MHz
WDW EM
SSB 0
LB 0.30 Hz
GB 0
PC 1.00
7.383 7.380 7.377 7.369 7.366 7.363 7.360 7.352 7.342 7.339 7.334 7.330 7.328 7.323 7.317 7.309 7.304 7.259
5.115
2.647 2.633 2.619 2.605 2.591 2.577 2.563
1.570
1.199 1.185
-0.000
13 12 11 10 9 8 7 6 5 4 3 2 1 0 -1 ppm
5.100
2.000
0.971
6.017
BnO
O
H
Me
Me

9

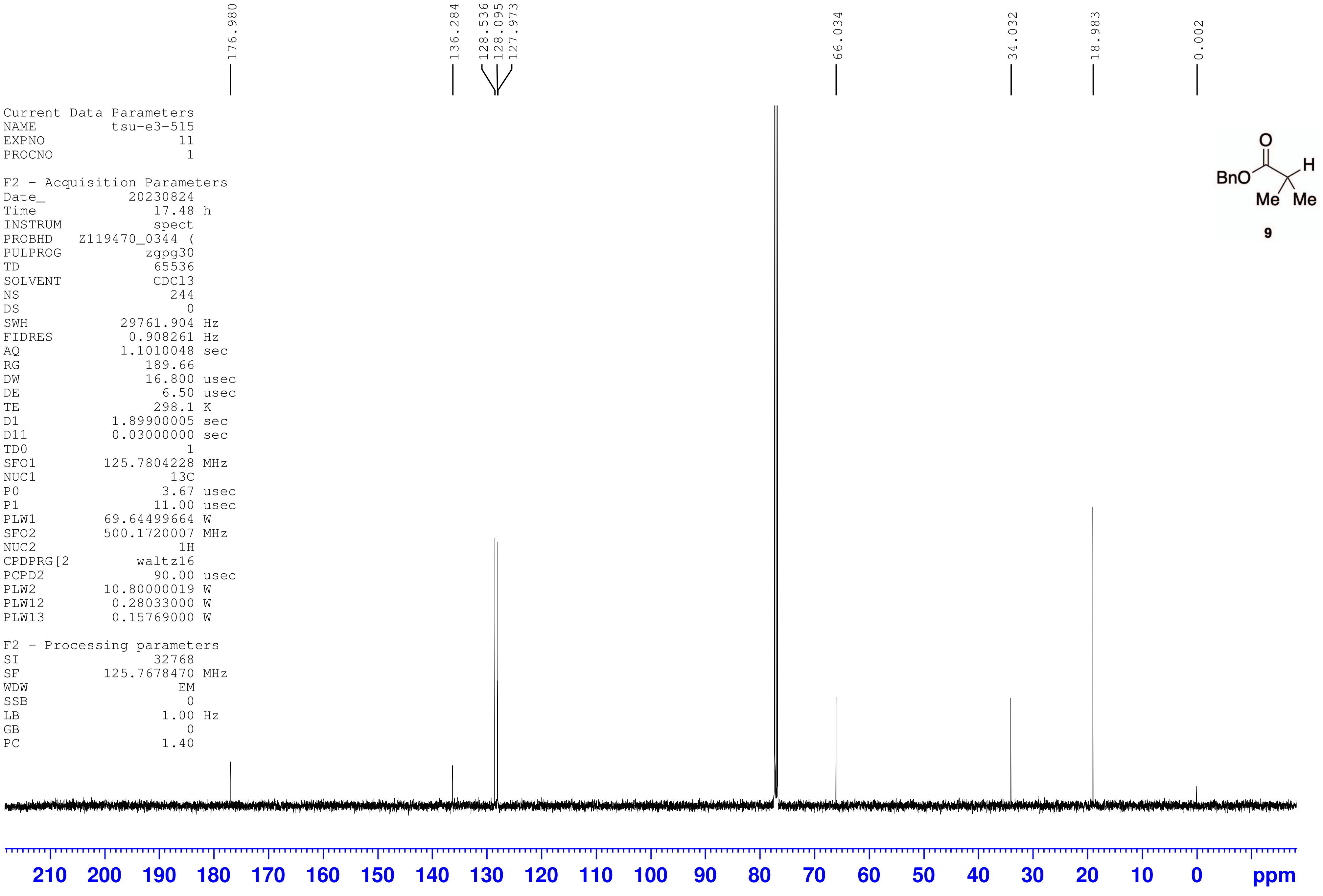

9

Current Data Parameters
NAME tsu-e3-515
EXPNO 11
PROCNO 1

F2 - Acquisition Parameters
Date_ 20230824
Time 17.48 h
INSTRUM spect
PROBHD Z119470_0344 (
PULPROG zgpg30
TD 65536
SOLVENT CDCl3
NS 244
DS 0
SWH 29761.904 Hz
FIDRES 0.908261 Hz
AQ 1.1010048 sec
RG 189.66
DW 16.800 usec
DE 6.50 usec
TE 298.1 K
D1 1.89900005 sec
D11 0.03000000 sec
TD0 1
SFO1 125.7804228 MHz
NUC1 13C
P0 3.67 usec
P1 11.00 usec
PLW1 69.64499664 W
SFO2 500.1720007 MHz
NUC2 1H
CPDPRG[2 waltz16
PCPD2 90.00 usec
PLW2 10.80000019 W
PLW12 0.28033000 W
PLW13 0.15769000 W

F2 - Processing parameters
SI 32768
SF 125.7678470 MHz
WDW EM
SSB 0
LB 1.00 Hz
GB 0
PC 1.40

10

Current Data Parameters
NAME tsu-e3-456a
EXPNO 10
PROCNO 1

F2 - Acquisition Parameters
Date_ 20230618
Time 17.32 h
INSTRUM spect
PROBHD Z119470_0344 (
PULPROG zg30
TD 65536
SOLVENT CDCl3
NS 1
DS 0
SWH 8012.820 Hz
FIDRES 0.244532 Hz
AQ 4.0894465 sec
RG 116.65
DW 62.400 usec
DE 6.50 usec
TE 298.0 K
D1 1.00000000 sec
TD0 1
SFO1 500.1730010 MHz
NUC1 1H
P0 4.83 usec
P1 14.50 usec
PLW1 10.80000019 W

F2 - Processing parameters
SI 65536
SF 500.1700124 MHz
WDW EM
SSB 0
LB 0.30 Hz
GB 0
PC 1.00

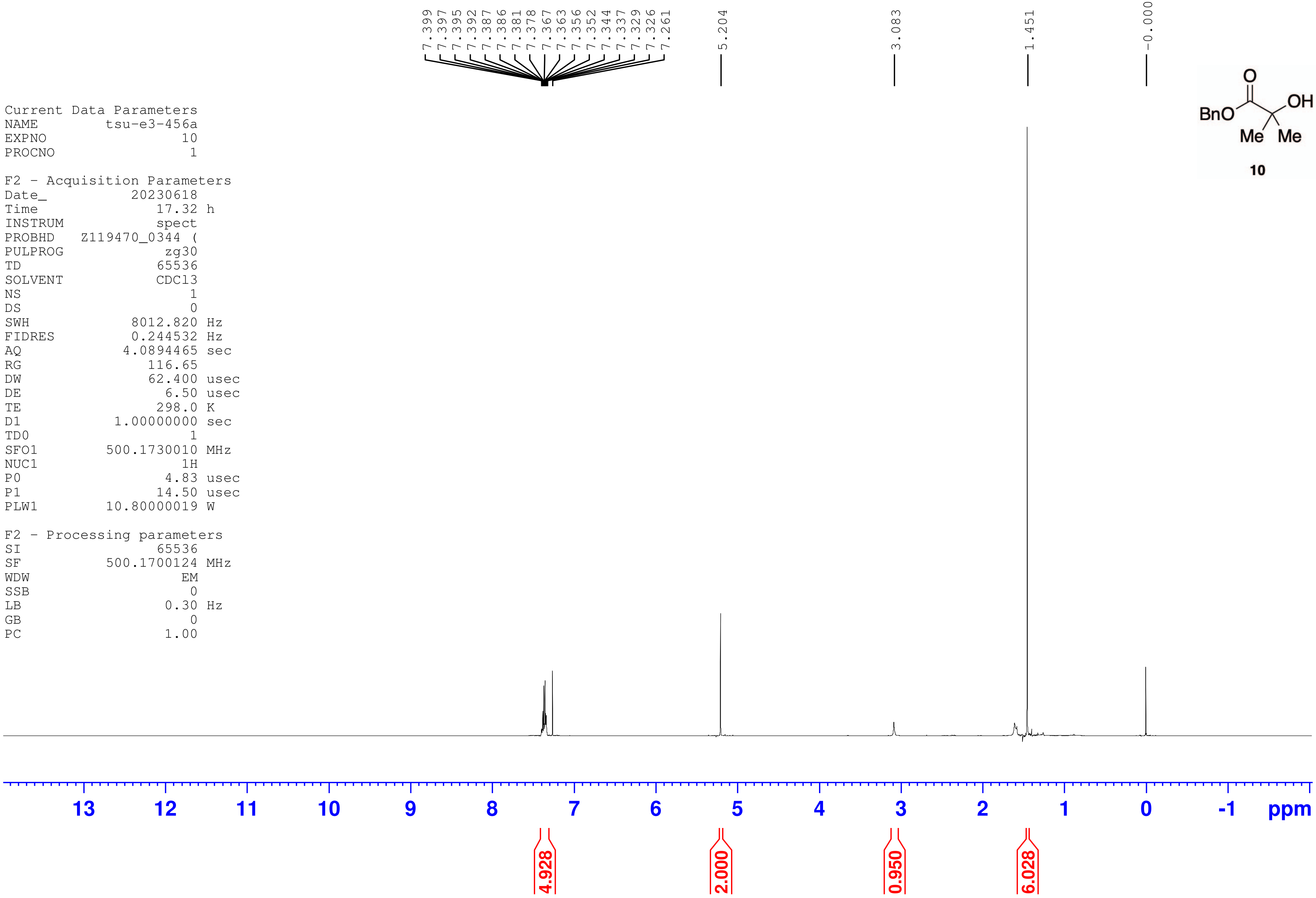

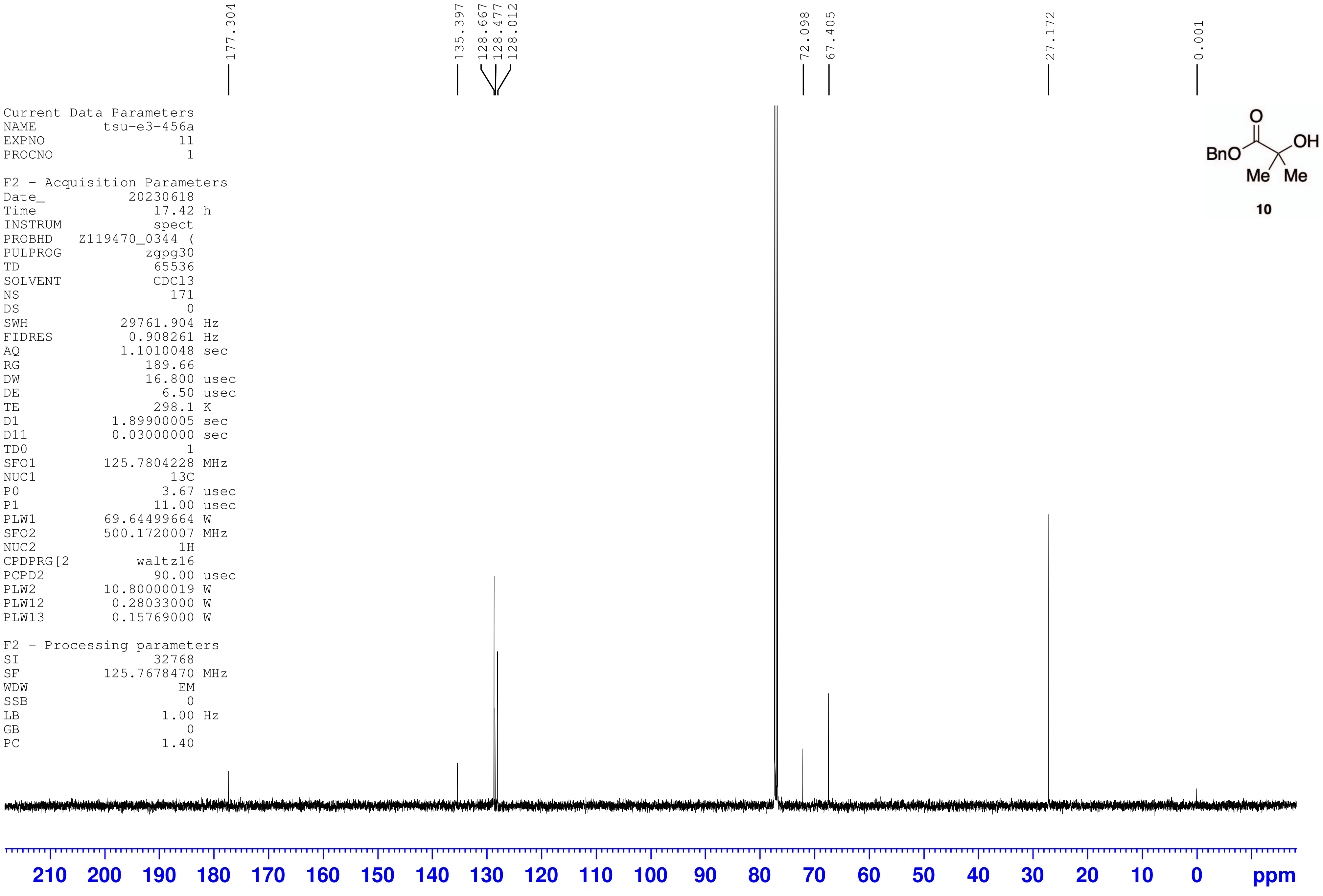

Current Data Parameters
NAME tsu-e3-456a
EXPNO 11
PROCNO 1

F2 - Acquisition Parameters
Date_ 20230618
Time 17.42 h
INSTRUM spect
PROBHD Z119470_0344 (
PULPROG zgpg30
TD 65536
SOLVENT CDCl3
NS 171
DS 0
SWH 29761.904 Hz
FIDRES 0.908261 Hz
AQ 1.1010048 sec
RG 189.66
DW 16.800 usec
DE 6.50 usec
TE 298.1 K
D1 1.89900005 sec
D11 0.03000000 sec
TD0 1
SFO1 125.7804228 MHz
NUC1 13C
P0 3.67 usec
P1 11.00 usec
PLW1 69.64499664 W
SFO2 500.1720007 MHz
NUC2 1H
CPDPRG[2 waltz16
PCPD2 90.00 usec
PLW2 10.80000019 W
PLW12 0.28033000 W
PLW13 0.15769000 W

F2 - Processing parameters
SI 32768
SF 125.7678470 MHz
WDW EM
SSB 0
LB 1.00 Hz
GB 0
PC 1.40

10

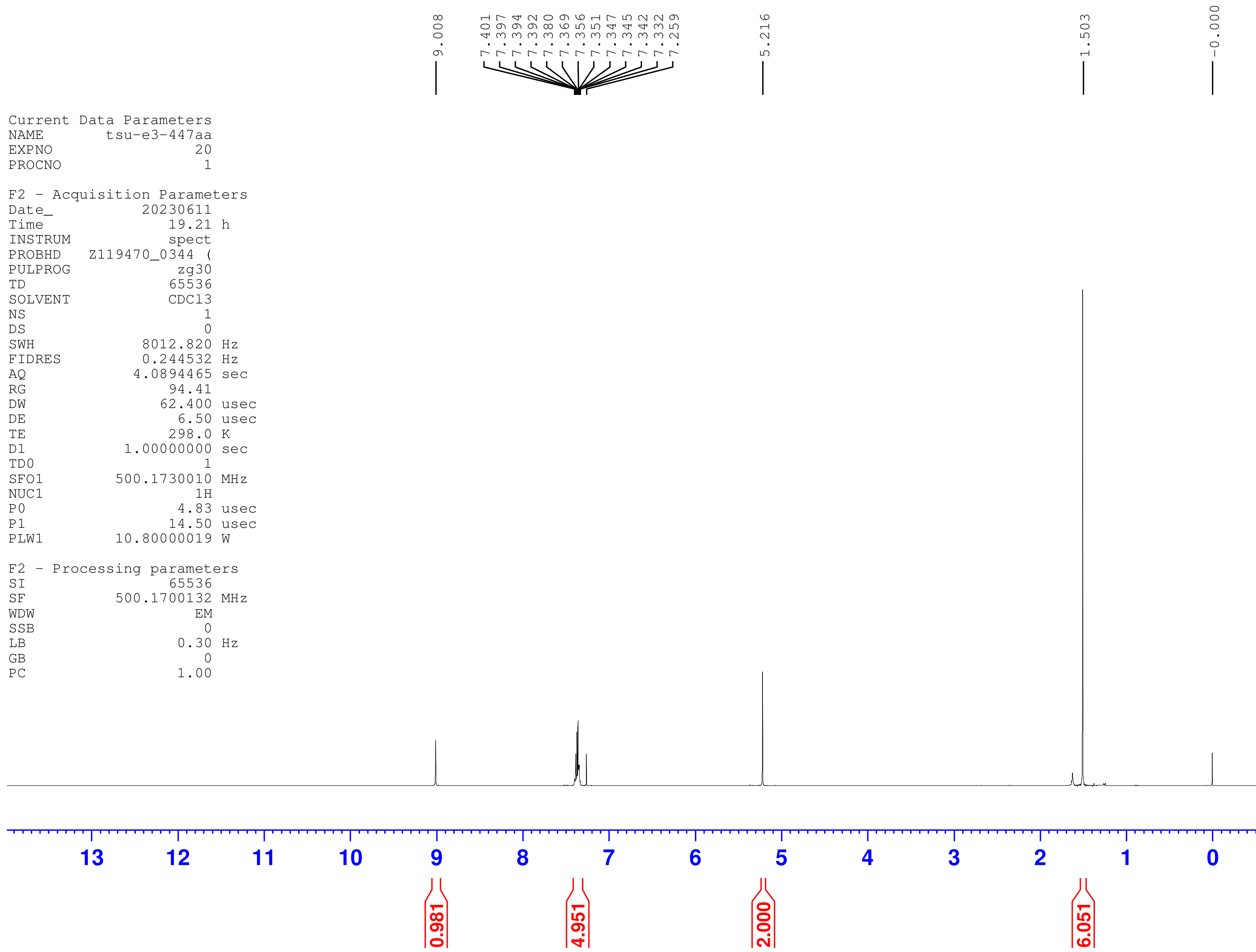

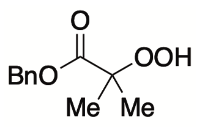

11

Current Data Parameters
NAME tsu-e3-447aa
EXPNO 20
PROCNO 1

F2 - Acquisition Parameters
Date_ 20230611
Time 19.21 h
INSTRUM spect
PROBHD Z119470_0344 (
PULPROG zg30
TD 65536
SOLVENT CDCl3
NS 1
DS 0
SWH 8012.820 Hz
FIDRES 0.244532 Hz
AQ 4.0894465 sec
RG 94.41
DW 62.400 usec
DE 6.50 usec
TE 298.0 K
D1 1.00000000 sec
TD0 1
SFO1 500.1730010 MHz
NUC1 1H
P0 4.83 usec
P1 14.50 usec
PLW1 10.80000019 W

F2 - Processing parameters
SI 65536
SF 500.1700132 MHz
WDW EM
SSB 0
LB 0.30 Hz
GB 0
PC 1.00

Current Data Parameters
NAME tsu-e3-447aa
EXPNO 21
PROCNO 1

F2 - Acquisition Parameters
Date_ 20230611
Time 19.25 h
INSTRUM spect
PROBHD Z119470_0344 (
PULPROG zgpg30
TD 65536
SOLVENT CDCl3
NS 61
DS 0
SWH 29761.904 Hz
FIDRES 0.908261 Hz
AQ 1.1010048 sec
RG 189.66
DW 16.800 usec
DE 6.50 usec
TE 298.1 K
D1 1.89900005 sec
D11 0.03000000 sec
TD0 1
SFO1 125.7804228 MHz
NUC1 13C
P0 3.67 usec
P1 11.00 usec
PLW1 69.64499664 W
SFO2 500.1720007 MHz
NUC2 1H
CPDPRG[2 waltz16
PCPD2 90.00 usec
PLW2 10.80000019 W
PLW12 0.28033000 W
PLW13 0.15769000 W

F2 - Processing parameters
SI 32768
SF 125.7678470 MHz
WDW EM
SSB 0
LB 1.00 Hz
GB 0
PC 1.40

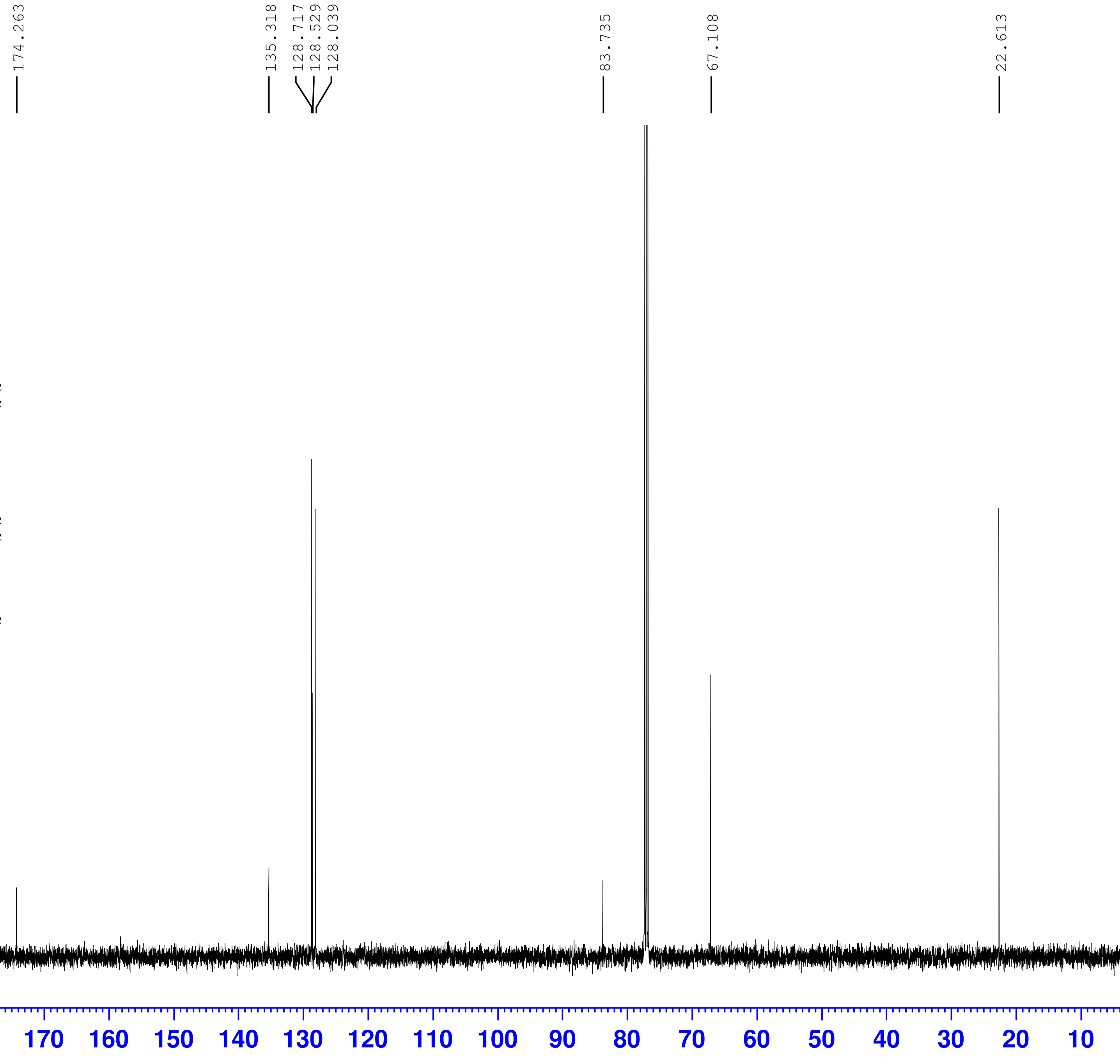

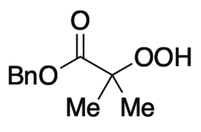

11

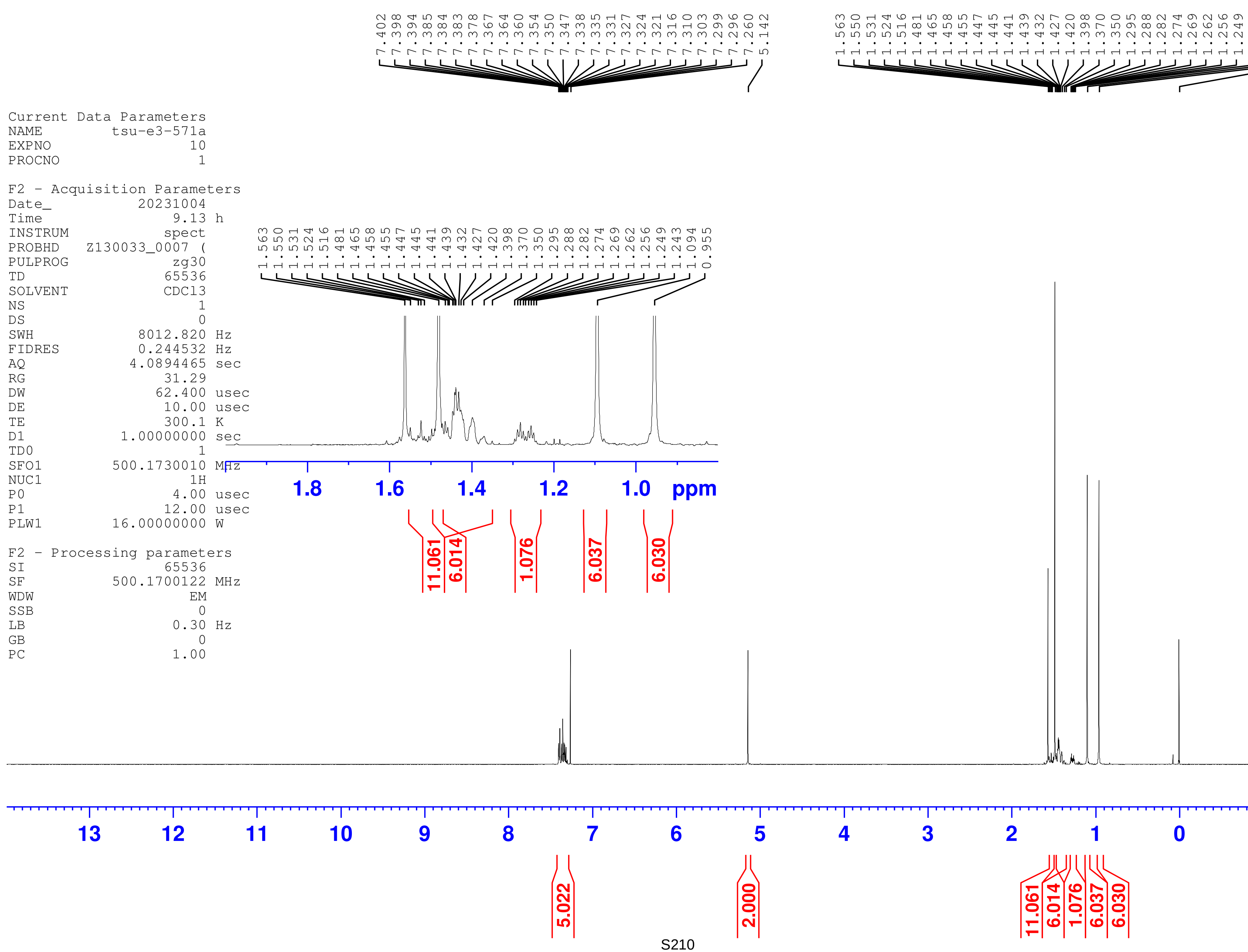
7.402 7.398 7.394 7.385 7.384 7.383 7.378 7.367 7.364 7.360 7.354 7.350 7.347 7.338 7.335 7.331 7.327 7.324 7.321 7.316 7.310 7.303 7.299 7.296 7.260 5.142
1.563 1.550 1.531 1.524 1.516 1.481 1.465 1.458 1.455 1.447 1.445 1.441 1.439 1.432 1.427 1.420 1.398 1.370 1.350 1.295 1.288 1.282 1.274 1.269 1.262 1.256 1.249 1.243 1.094 0.955 -0.000
Current Data Parameters
NAME tsu-e3-571a
EXPNO 10
PROCNO 1
F2 - Acquisition Parameters
Date_ 20231004
Time 9.13 h
INSTRUM spect
PROBHD Z130033_0007 (
PULPROG zg30
TD 65536
SOLVENT CDCl3
NS 1
DS 0
SWH 8012.820 Hz
FIDRES 0.244532 Hz
AQ 4.0894465 sec
RG 31.29
DW 62.400 usec
DE 10.00 usec
TE 300.1 K
D1 1.00000000 sec
TD0 1
SFO1 500.1730010 MHz
NUC1 1H
P0 4.00 usec
P1 12.00 usec
PLW1 16.00000000 W
F2 - Processing parameters
SI 65536
SF 500.1700122 MHz
WDW EM
SSB 0
LB 0.30 Hz
GB 0
PC 1.00
1.563 1.550 1.531 1.524 1.516 1.481 1.465 1.458 1.455 1.447 1.445 1.441 1.439 1.432 1.427 1.420 1.398 1.370 1.350 1.295 1.288 1.282 1.274 1.269 1.262 1.256 1.249 1.243 1.094 0.955
1.8 1.6 1.4 1.2 1.0 ppm
11.061 6.014 1.076 6.037 6.030
13 12 11 10 9 8 7 6 5 4 3 2 1 0 -1 ppm
5.022 2.000 11.061 6.014 1.076 6.037 6.030

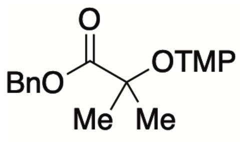
O
BnO
OTMP
Me
Me

12

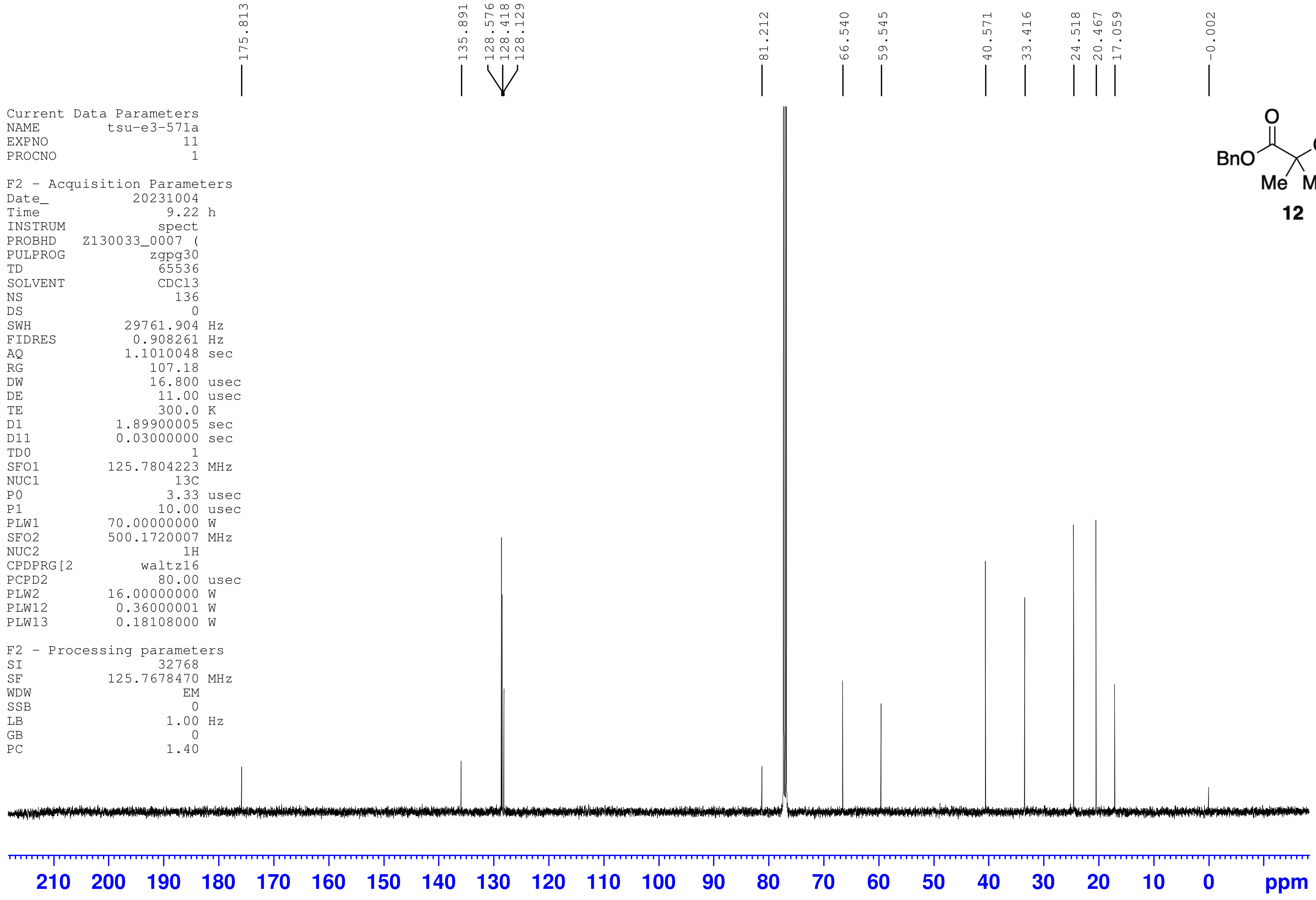
175.813
135.891
128.576
128.418
128.129
81.212
66.540
59.545
40.571
33.416
24.518
20.467
17.059
-0.002
Current Data Parameters
NAME tsu-e3-571a
EXPNO 11
PROCNO 1
F2 - Acquisition Parameters
Date_ 20231004
Time 9.22 h
INSTRUM spect
PROBHD Z130033_0007 (
PULPROG zgpg30
TD 65536
SOLVENT CDCl3
NS 136
DS 0
SWH 29761.904 Hz
FIDRES 0.908261 Hz
AQ 1.1010048 sec
RG 107.18
DW 16.800 usec
DE 11.00 usec
TE 300.0 K
D1 1.89900005 sec
D11 0.03000000 sec
TD0 1
SFO1 125.7804223 MHz
NUC1 13C
P0 3.33 usec
P1 10.00 usec
PLW1 70.00000000 W
SFO2 500.1720007 MHz
NUC2 1H
CPDPRG[2 waltz16
PCPD2 80.00 usec
PLW2 16.00000000 W
PLW12 0.36000001 W
PLW13 0.18108000 W
F2 - Processing parameters
SI 32768
SF 125.7678470 MHz
WDW EM
SSB 0
LB 1.00 Hz
GB 0
PC 1.40
210 200 190 180 170 160 150 140 130 120 110 100 90 80 70 60 50 40 30 20 10 0 ppm

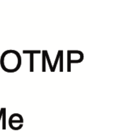
O
BnO
OTMP
Me
Me

12

Current Data Parameters
NAME tsu-e3-541
EXPNO 10
PROCNO 1

F2 - Acquisition Parameters
Date_ 20230911
Time 18.05 h
INSTRUM spect
PROBHD Z119470_0344 (
PULPROG zg30
TD 65536
SOLVENT CDCl3
NS 1
DS 0
SWH 8012.820 Hz
FIDRES 0.244532 Hz
AQ 4.0894465 sec
RG 130.52
DW 62.400 usec
DE 6.50 usec
TE 298.0 K
D1 1.00000000 sec
TD0 1
SFO1 500.1730010 MHz
NUC1 1H
P0 4.83 usec
P1 14.50 usec
PLW1 10.80000019 W

F2 - Processing parameters
SI 65536
SF 500.1700134 MHz
WDW EM
SSB 0
LB 0.30 Hz
GB 0
PC 1.00

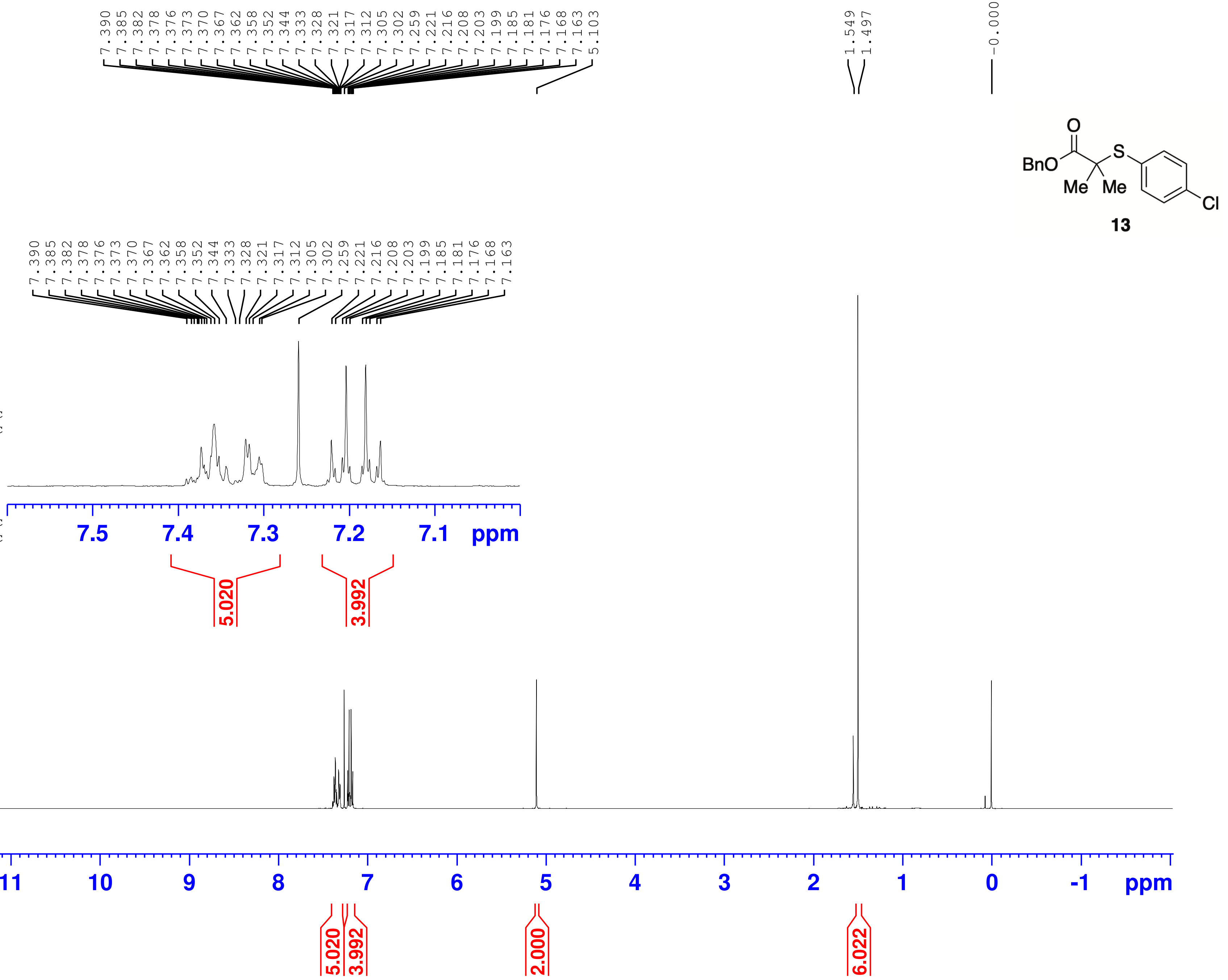

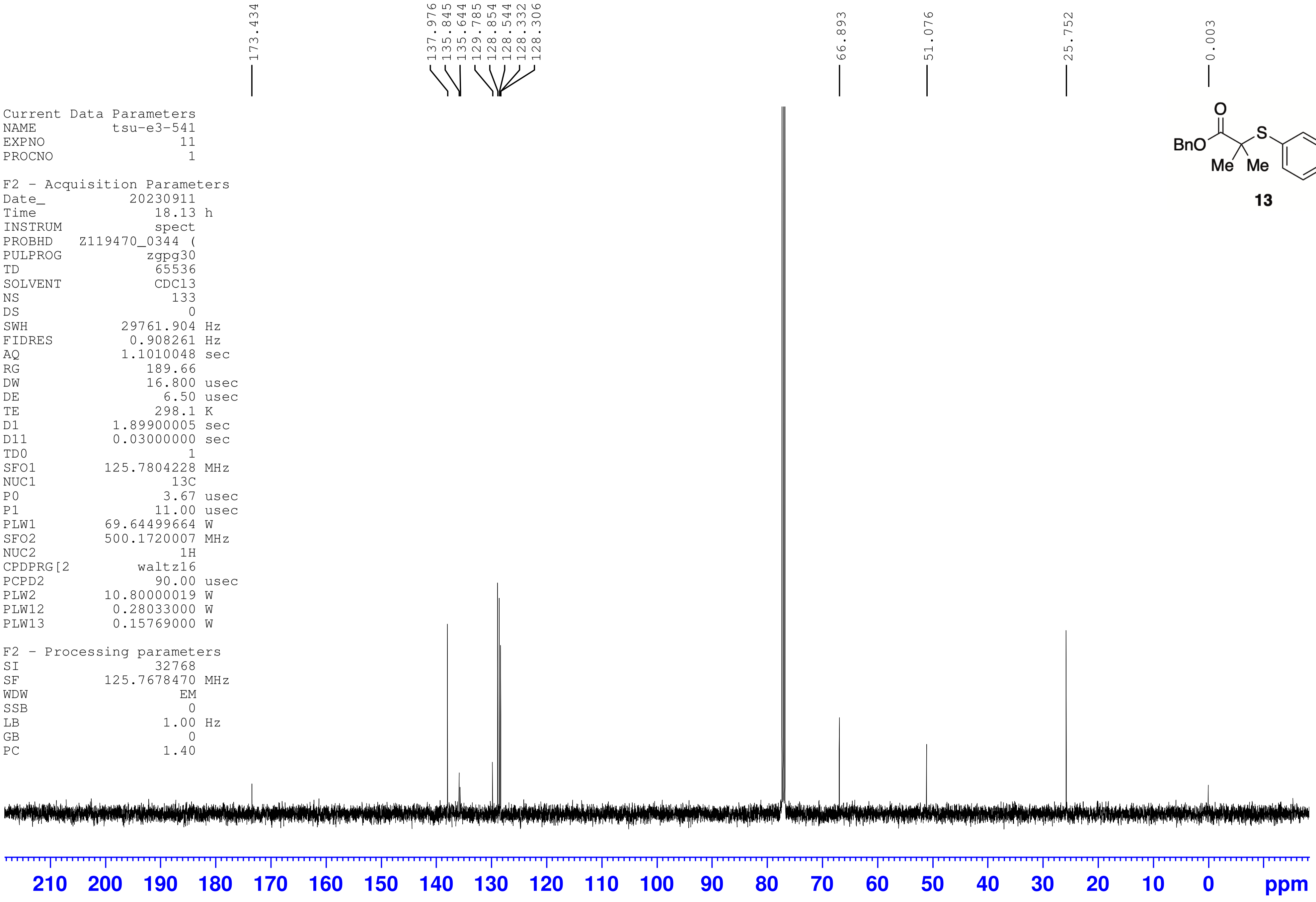


```
Current Data Parameters
NAME          tsu-e3-541
EXPNO                 11
PROCNO                 1

F2 - Acquisition Parameters
Date_           20230911
Time              18.13 h
INSTRUM            spect
PROBHD   Z119470_0344 (
PULPROG          zgpg30
TD                65536
SOLVENT           CDCl3
NS                  133
DS                    0
SWH           29761.904 Hz
FIDRES         0.908261 Hz
AQ            1.1010048 sec
RG               189.66
DW               16.800 usec
DE                 6.50 usec
TE                298.1 K
D1           1.89900005 sec
D11          0.03000000 sec
TD0                   1
SFO1        125.7804228 MHz
NUC1                13C
P0                 3.67 usec
P1                11.00 usec
PLW1        69.64499664 W
SFO2        500.1720007 MHz
NUC2                 1H
CPDPRG[2        waltz16
PCPD2             90.00 usec
PLW2        10.80000019 W
PLW12        0.28033000 W
PLW13        0.15769000 W

F2 - Processing parameters
SI                32768
SF          125.7678470 MHz
WDW                  EM
SSB                   0
LB                 1.00 Hz
GB                    0
PC                 1.40
```


13

S213

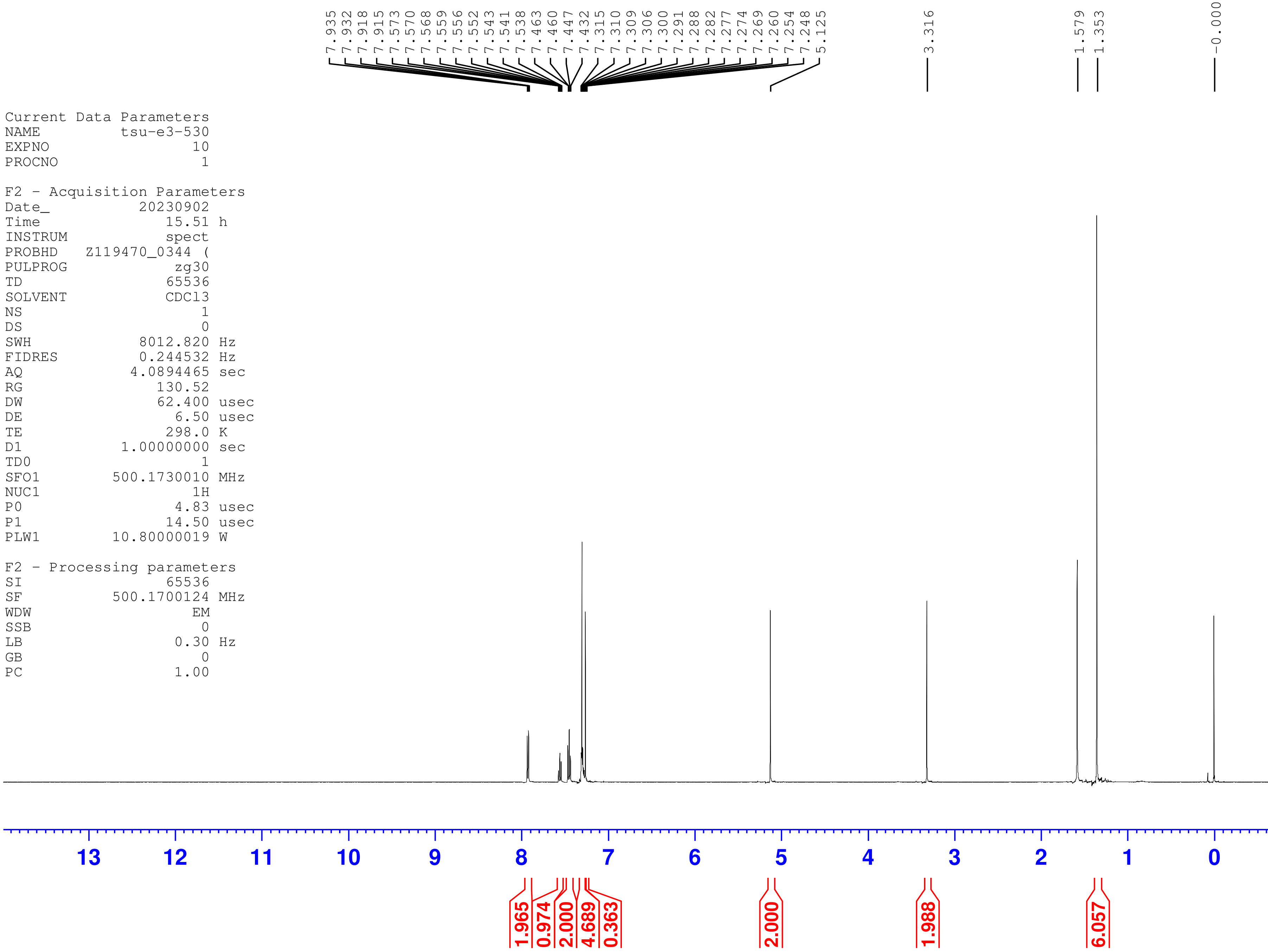
7.935
7.932
7.918
7.915
7.573
7.570
7.568
7.559
7.556
7.552
7.543
7.541
7.538
7.463
7.460
7.447
7.432
7.315
7.310
7.309
7.306
7.300
7.291
7.288
7.282
7.277
7.274
7.269
7.260
7.254
7.248
5.125
3.316
1.579
1.353
-0.000
Current Data Parameters
NAME tsu-e3-530
EXPNO 10
PROCNO 1
F2 - Acquisition Parameters
Date_ 20230902
Time 15.51 h
INSTRUM spect
PROBHD Z119470_0344 (
PULPROG zg30
TD 65536
SOLVENT CDCl3
NS 1
DS 0
SWH 8012.820 Hz
FIDRES 0.244532 Hz
AQ 4.0894465 sec
RG 130.52
DW 62.400 usec
DE 6.50 usec
TE 298.0 K
D1 1.00000000 sec
TD0 1
SFO1 500.1730010 MHz
NUC1 1H
P0 4.83 usec
P1 14.50 usec
PLW1 10.80000019 W
F2 - Processing parameters
SI 65536
SF 500.1700124 MHz
WDW EM
SSB 0
LB 0.30 Hz
GB 0
PC 1.00
13
12
11
10
9
8
7
6
5
4
3
2
1
0
-1
ppm
1.965
0.974
2.000
4.689
0.363
2.000
1.988
6.057

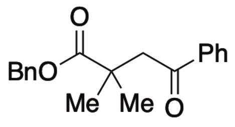
BnO
O
Me
Me
O
Ph

14

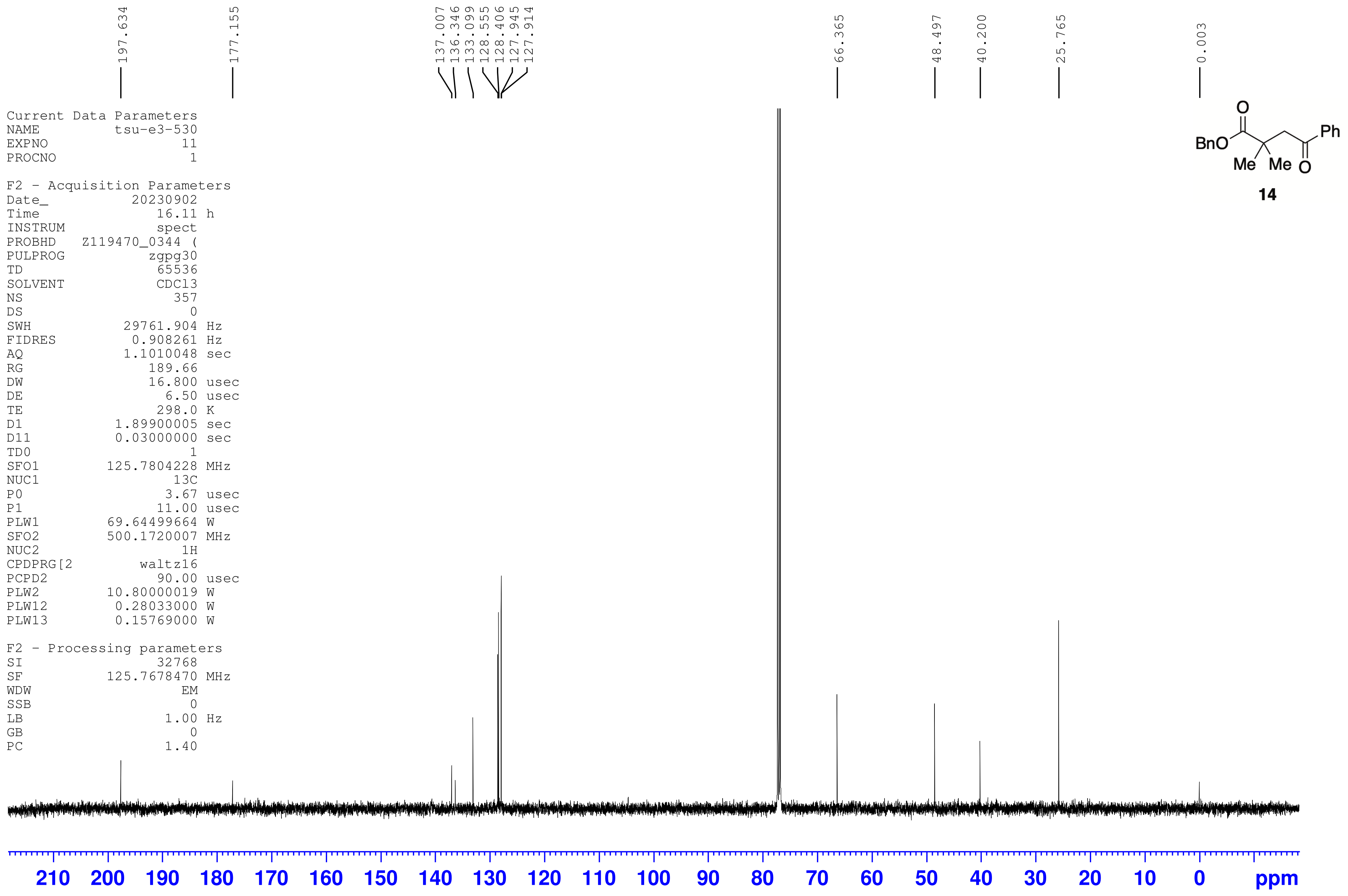
197.634
177.155
137.007
136.346
133.099
128.555
128.406
127.945
127.914
66.365
48.497
40.200
25.765
0.003
Current Data Parameters
NAME tsu-e3-530
EXPNO 11
PROCNO 1
F2 - Acquisition Parameters
Date_ 20230902
Time 16.11 h
INSTRUM spect
PROBHD Z119470_0344 (
PULPROG zgpg30
TD 65536
SOLVENT CDCl3
NS 357
DS 0
SWH 29761.904 Hz
FIDRES 0.908261 Hz
AQ 1.1010048 sec
RG 189.66
DW 16.800 usec
DE 6.50 usec
TE 298.0 K
D1 1.89900005 sec
D11 0.03000000 sec
TD0 1
SFO1 125.7804228 MHz
NUC1 13C
P0 3.67 usec
P1 11.00 usec
PLW1 69.64499664 W
SFO2 500.1720007 MHz
NUC2 1H
CPDPRG[2 waltz16
PCPD2 90.00 usec
PLW2 10.80000019 W
PLW12 0.28033000 W
PLW13 0.15769000 W
F2 - Processing parameters
SI 32768
SF 125.7678470 MHz
WDW EM
SSB 0
LB 1.00 Hz
GB 0
PC 1.40
210 200 190 180 170 160 150 140 130 120 110 100 90 80 70 60 50 40 30 20 10 0 ppm
O
BnO
Ph
Me
Me
O

14

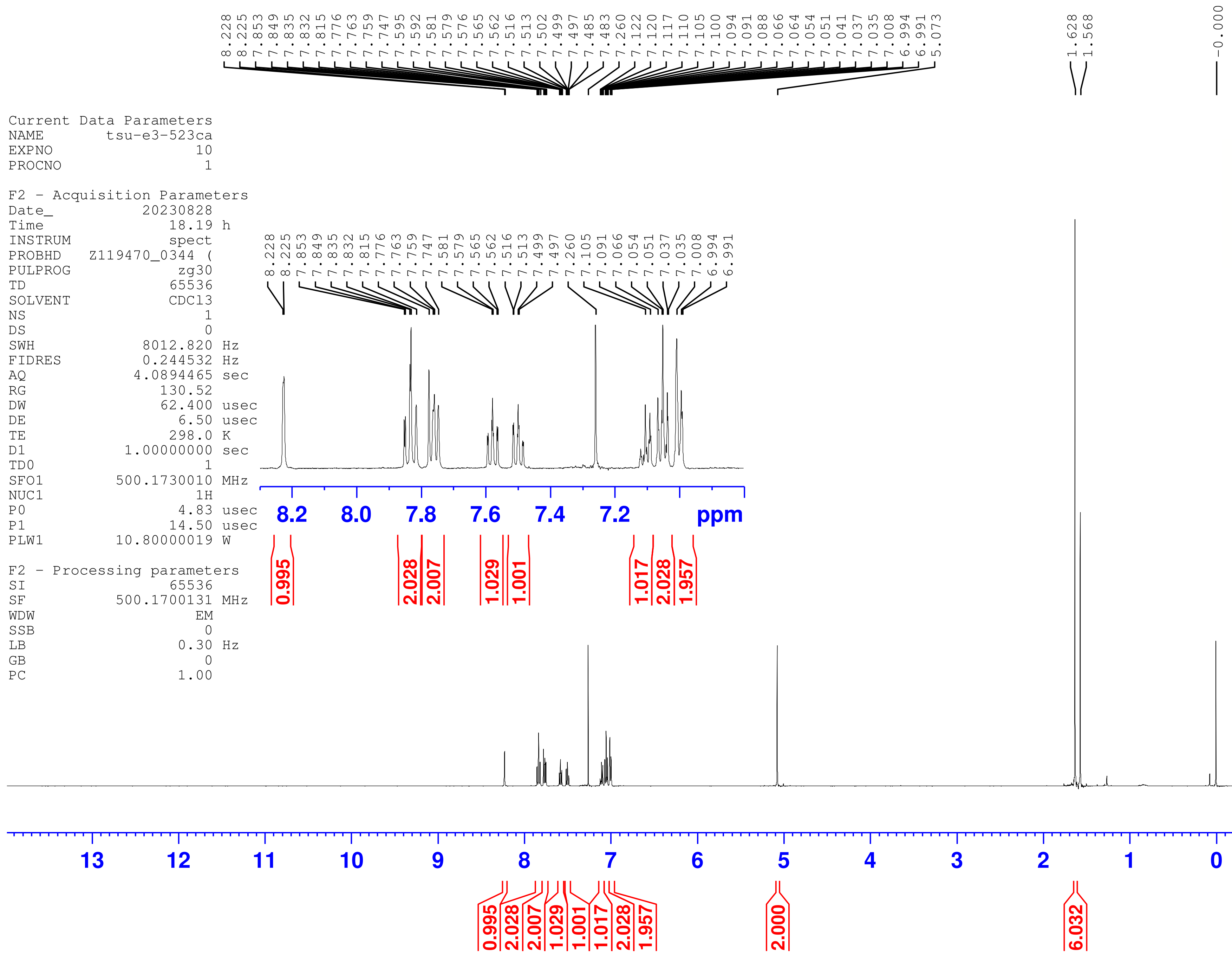
Current Data Parameters
NAME tsu-e3-523ca
EXPNO 10
PROCNO 1
F2 - Acquisition Parameters
Date_ 20230828
Time 18.19 h
INSTRUM spect
PROBHD Z119470_0344 (
PULPROG zg30
TD 65536
SOLVENT CDCl3
NS 1
DS 0
SWH 8012.820 Hz
FIDRES 0.244532 Hz
AQ 4.0894465 sec
RG 130.52
DW 62.400 usec
DE 6.50 usec
TE 298.0 K
D1 1.00000000 sec
TD0 1
SFO1 500.1730010 MHz
NUC1 1H
P0 4.83 usec
P1 14.50 usec
PLW1 10.80000019 W
F2 - Processing parameters
SI 65536
SF 500.1700131 MHz
WDW EM
SSB 0
LB 0.30 Hz
GB 0
PC 1.00
8.228 8.225 7.853 7.849 7.835 7.832 7.815 7.776 7.763 7.759 7.747 7.595 7.592 7.581 7.579 7.576 7.565 7.562 7.516 7.513 7.502 7.499 7.497 7.485 7.483 7.260 7.122 7.120 7.117 7.110 7.105 7.100 7.094 7.091 7.088 7.066 7.064 7.054 7.051 7.041 7.037 7.035 7.008 6.994 6.991 5.073
1.628 1.568
-0.000
0.995 2.028 2.007 1.029 1.001 1.017 2.028 1.957 2.000 6.032
ppm

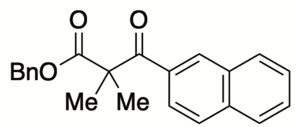
BnO
O
O
Me
Me

15

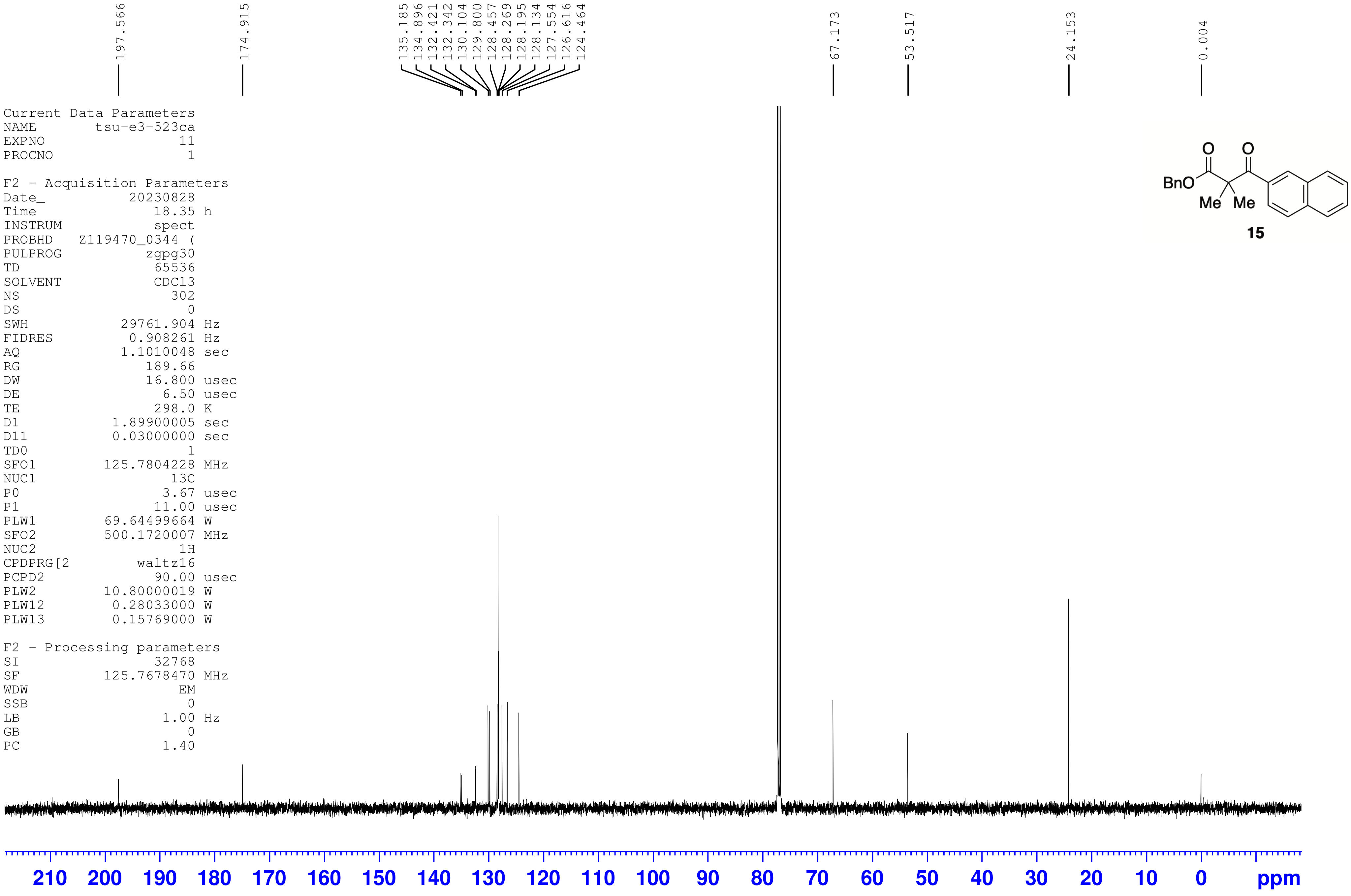
197.566
174.915
135.185
134.896
132.421
132.342
130.104
129.800
128.457
128.269
128.195
128.134
127.554
126.616
124.464
67.173
53.517
24.153
0.004
Current Data Parameters
NAME tsu-e3-523ca
EXPNO 11
PROCNO 1
F2 - Acquisition Parameters
Date_ 20230828
Time 18.35 h
INSTRUM spect
PROBHD Z119470_0344 (
PULPROG zgpg30
TD 65536
SOLVENT CDCl3
NS 302
DS 0
SWH 29761.904 Hz
FIDRES 0.908261 Hz
AQ 1.1010048 sec
RG 189.66
DW 16.800 usec
DE 6.50 usec
TE 298.0 K
D1 1.89900005 sec
D11 0.03000000 sec
TD0 1
SFO1 125.7804228 MHz
NUC1 13C
P0 3.67 usec
P1 11.00 usec
PLW1 69.64499664 W
SFO2 500.1720007 MHz
NUC2 1H
CPDPRG[2 waltz16
PCPD2 90.00 usec
PLW2 10.80000019 W
PLW12 0.28033000 W
PLW13 0.15769000 W
F2 - Processing parameters
SI 32768
SF 125.7678470 MHz
WDW EM
SSB 0
LB 1.00 Hz
GB 0
PC 1.40
210 200 190 180 170 160 150 140 130 120 110 100 90 80 70 60 50 40 30 20 10 0 ppm
BnO
O
O
Me
Me

15

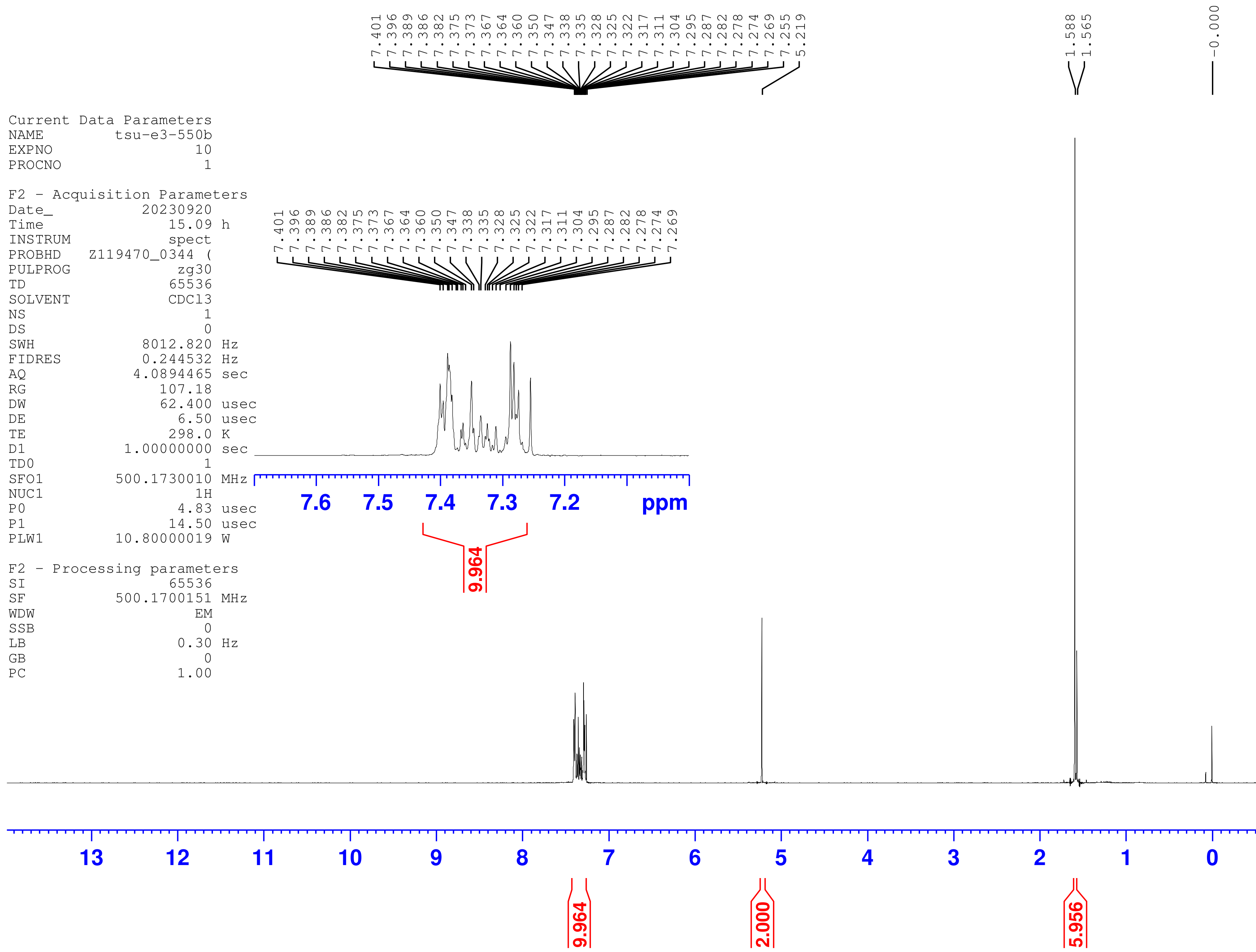

Current Data Parameters
NAME tsu-e3-550b
EXPNO 10
PROCNO 1
F2 - Acquisition Parameters
Date_ 20230920
Time 15.09 h
INSTRUM spect
PROBHD Z119470_0344 (
PULPROG zg30
TD 65536
SOLVENT CDCl3
NS 1
DS 0
SWH 8012.820 Hz
FIDRES 0.244532 Hz
AQ 4.0894465 sec
RG 107.18
DW 62.400 usec
DE 6.50 usec
TE 298.0 K
D1 1.00000000 sec
TD0 1
SFO1 500.1730010 MHz
NUC1 1H
P0 4.83 usec
P1 14.50 usec
PLW1 10.80000019 W
F2 - Processing parameters
SI 65536
SF 500.1700151 MHz
WDW EM
SSB 0
LB 0.30 Hz
GB 0
PC 1.00
7.401 7.396 7.389 7.386 7.382 7.375 7.373 7.367 7.364 7.360 7.350 7.347 7.338 7.335 7.328 7.325 7.322 7.317 7.311 7.304 7.295 7.287 7.282 7.278 7.274 7.269 7.255 5.219
1.588 1.565
-0.000
7.401 7.396 7.389 7.386 7.382 7.375 7.373 7.367 7.364 7.360 7.350 7.347 7.338 7.335 7.328 7.325 7.322 7.317 7.311 7.304 7.295 7.287 7.282 7.278 7.274 7.269
7.6 7.5 7.4 7.3 7.2 ppm
9.964
13 12 11 10 9 8 7 6 5 4 3 2 1 0 -1 ppm
9.964
2.000
5.956

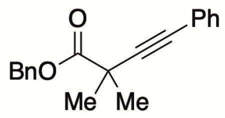

O
Ph
BnO
Me Me

16

```
Current Data Parameters
NAME          tsu-e3-550b
EXPNO                  11
PROCNO                  1

F2 - Acquisition Parameters
Date_            20230920
Time                15.27 h
INSTRUM             spect
PROBHD   Z119470_0344 (
PULPROG           zgpg30
TD                 65536
SOLVENT            CDCl3
NS                   124
DS                     0
SWH            29761.904 Hz
FIDRES          0.908261 Hz
AQ             1.1010048 sec
RG                189.66
DW                16.800 usec
DE                  6.50 usec
TE                 298.0 K
D1            1.89900005 sec
D11           0.03000000 sec
TD0                    1
SFO1         125.7804228 MHz
NUC1                 13C
P0                  3.67 usec
P1                 11.00 usec
PLW1         69.64499664 W
SFO2         500.1720007 MHz
NUC2                  1H
CPDPRG[2         waltz16
PCPD2              90.00 usec
PLW2         10.80000019 W
PLW12         0.28033000 W
PLW13         0.15769000 W

F2 - Processing parameters
SI                 32768
SF           125.7678470 MHz
WDW                   EM
SSB                    0
LB                  1.00 Hz
GB                     0
PC                  1.40
```

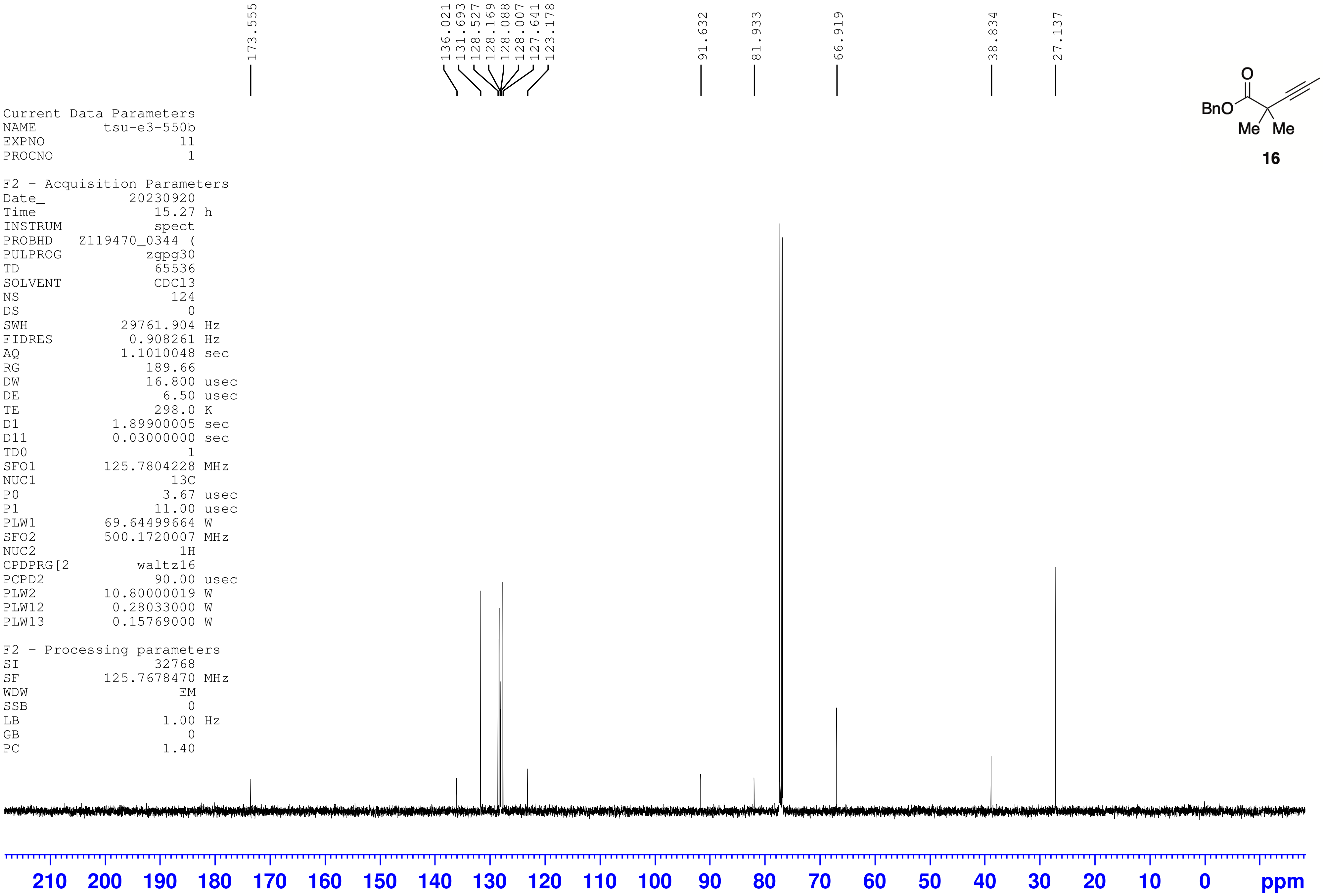

16

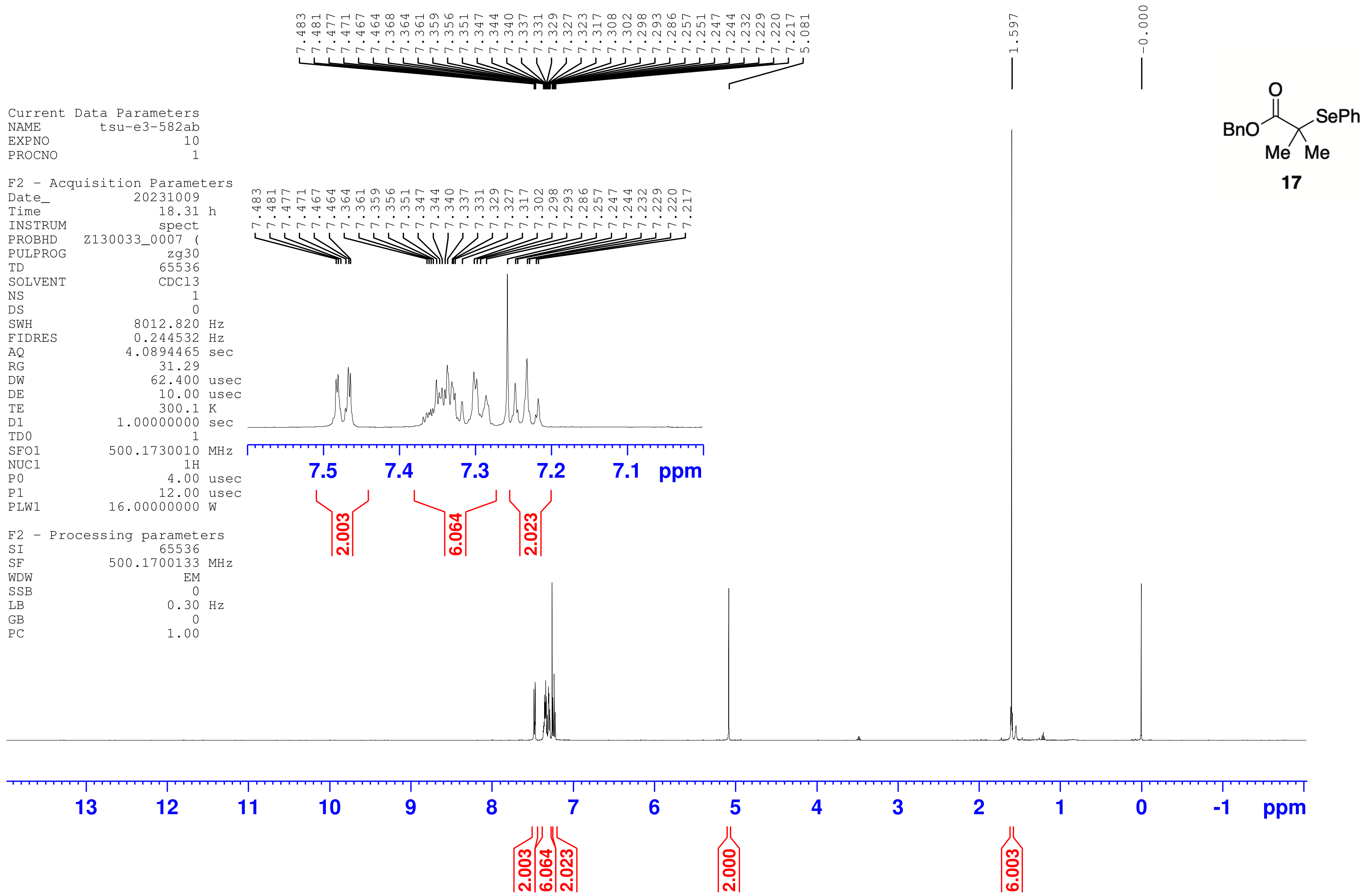
Current Data Parameters
NAME tsu-e3-582ab
EXPNO 10
PROCNO 1
F2 - Acquisition Parameters
Date_ 20231009
Time 18.31 h
INSTRUM spect
PROBHD Z130033_0007 (
PULPROG zg30
TD 65536
SOLVENT CDCl3
NS 1
DS 0
SWH 8012.820 Hz
FIDRES 0.244532 Hz
AQ 4.0894465 sec
RG 31.29
DW 62.400 usec
DE 10.00 usec
TE 300.1 K
D1 1.00000000 sec
TD0 1
SFO1 500.1730010 MHz
NUC1 1H
P0 4.00 usec
P1 12.00 usec
PLW1 16.00000000 W
F2 - Processing parameters
SI 65536
SF 500.1700133 MHz
WDW EM
SSB 0
LB 0.30 Hz
GB 0
PC 1.00
7.483
7.481
7.477
7.471
7.467
7.464
7.368
7.364
7.361
7.359
7.356
7.351
7.347
7.344
7.340
7.337
7.331
7.329
7.327
7.323
7.317
7.308
7.302
7.298
7.293
7.286
7.257
7.251
7.247
7.244
7.232
7.229
7.220
7.217
5.081
1.597
-0.000
7.483
7.481
7.477
7.471
7.467
7.464
7.364
7.361
7.359
7.356
7.351
7.347
7.344
7.340
7.337
7.331
7.329
7.327
7.317
7.302
7.298
7.293
7.286
7.257
7.247
7.244
7.232
7.229
7.220
7.217
7.5
7.4
7.3
7.2
7.1
ppm
2.003
6.064
2.023
13
12
11
10
9
8
7
6
5
4
3
2
1
0
-1
ppm
2.003
6.064
2.023
2.000
6.003
O
BnO
SePh
Me
Me

17

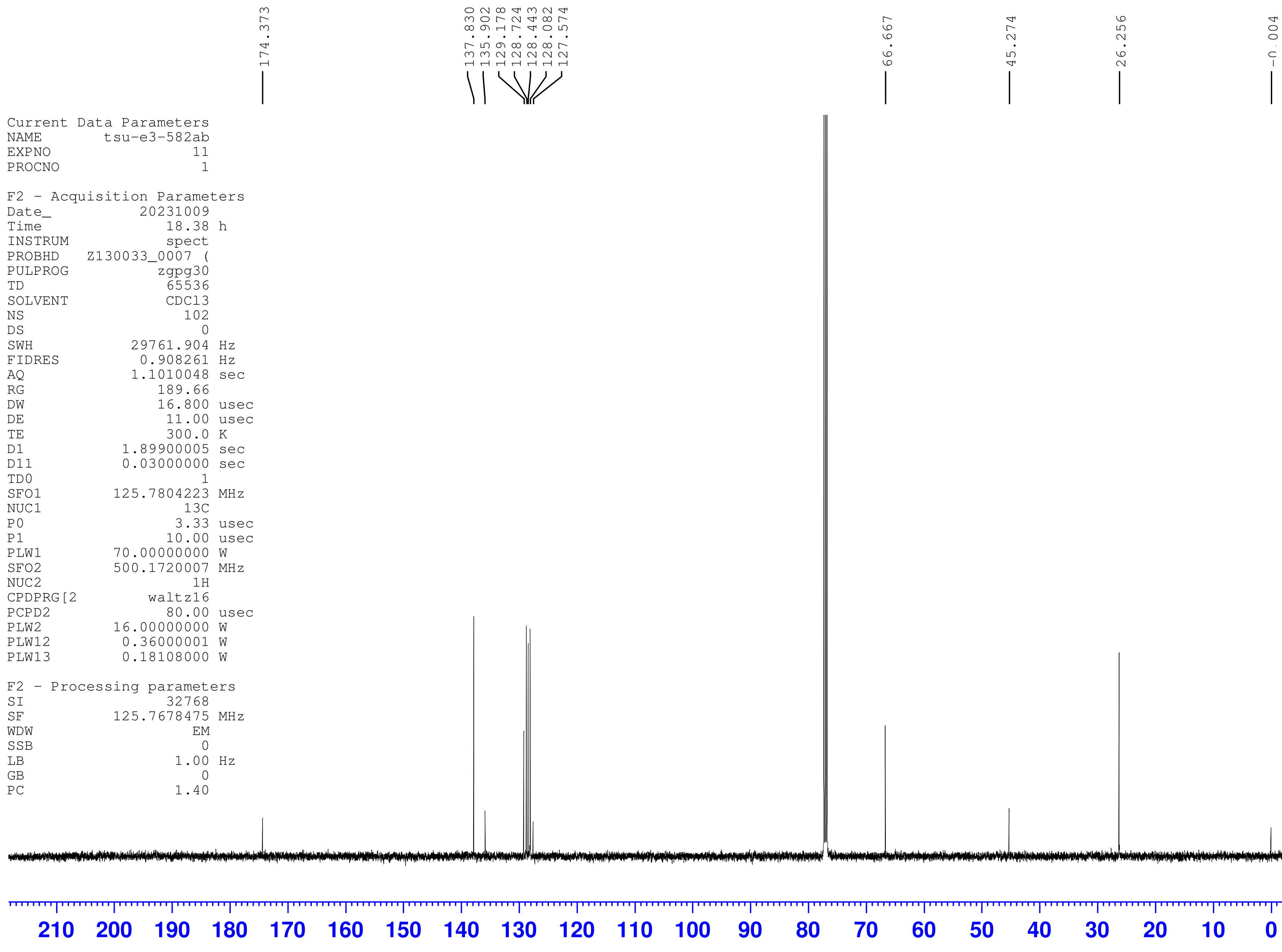
174.373
137.830
135.902
129.178
128.724
128.443
128.082
127.574
66.667
45.274
26.256
-0.004
Current Data Parameters
NAME tsu-e3-582ab
EXPNO 11
PROCNO 1
F2 - Acquisition Parameters
Date_ 20231009
Time 18.38 h
INSTRUM spect
PROBHD Z130033_0007 (
PULPROG zgpg30
TD 65536
SOLVENT CDCl3
NS 102
DS 0
SWH 29761.904 Hz
FIDRES 0.908261 Hz
AQ 1.1010048 sec
RG 189.66
DW 16.800 usec
DE 11.00 usec
TE 300.0 K
D1 1.89900005 sec
D11 0.03000000 sec
TD0 1
SFO1 125.7804223 MHz
NUC1 13C
P0 3.33 usec
P1 10.00 usec
PLW1 70.00000000 W
SFO2 500.1720007 MHz
NUC2 1H
CPDPRG[2 waltz16
PCPD2 80.00 usec
PLW2 16.00000000 W
PLW12 0.36000001 W
PLW13 0.18108000 W
F2 - Processing parameters
SI 32768
SF 125.7678475 MHz
WDW EM
SSB 0
LB 1.00 Hz
GB 0
PC 1.40
210 200 190 180 170 160 150 140 130 120 110 100 90 80 70 60 50 40 30 20 10 0 ppm

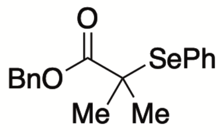
O
BnO
SePh
Me
Me

17

```
Current Data Parameters
NAME          tsu-e3-582ab
EXPNO                   13
PROCNO                   1

F2 - Acquisition Parameters
Date_            20231009
Time                18.49 h
INSTRUM             spect
PROBHD   Z130033_0007 (
PULPROG          deptsp90
TD                  65536
SOLVENT             CDCl3
NS                     17
DS                      0
SWH             29761.904 Hz
FIDRES           0.908261 Hz
AQ              1.1010048 sec
RG                 189.66
DW                 16.800 usec
DE                  11.00 usec
TE                  300.0 K
CNST2         145.0000000
D1             1.89900005 sec
D2             0.00344828 sec
D12            0.00002000 sec
TD0                     1
SFO1          125.7804223 MHz
NUC1                  13C
P1                  10.00 usec
P13               2000.00 usec
PLW0                    0 W
PLW1          70.00000000 W
SPNAM[5]      Crp60comp.4
SPOAL5              0.500
SPOFFS5                 0 Hz
SPW5          10.69499969 W
SFO2          500.1720007 MHz
NUC2                   1H
CPDPRG[2          waltz16
P3                  12.00 usec
P4                  24.00 usec
PCPD2               80.00 usec
PLW2          16.00000000 W
PLW12          0.36000001 W

F2 - Processing parameters
SI                  32768
SF            125.7678470 MHz
WDW                    EM
SSB                     0
LB                   1.00 Hz
GB                      0
PC                   1.40
```

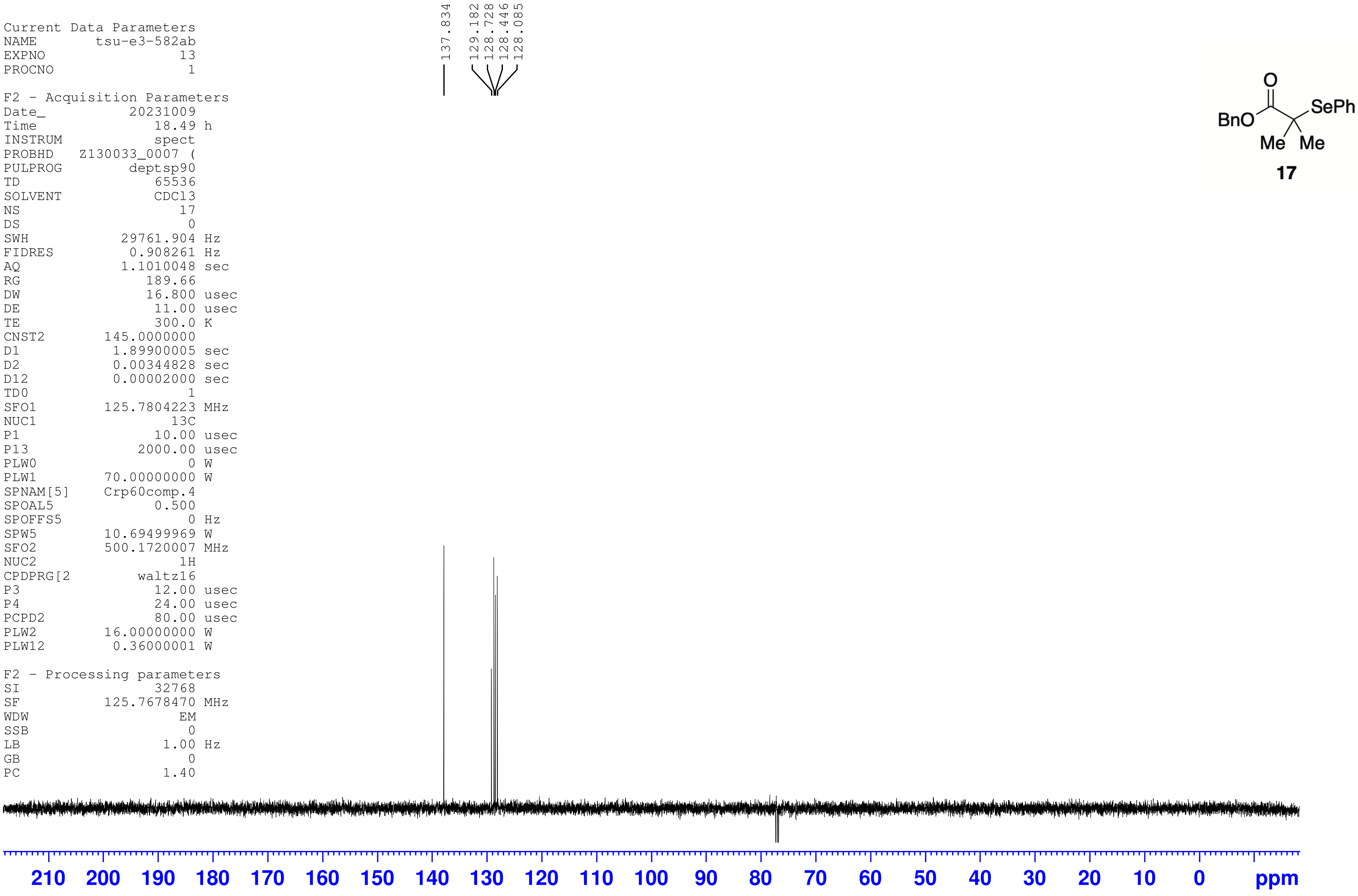

17

Current Data Parameters
NAME tsu-e3-579b
EXPNO 10
PROCNO 1

F2 - Acquisition Parameters
Date_ 20231007
Time 15.51 h
INSTRUM spect
PROBHD Z130033_0007 (
PULPROG zg30
TD 65536
SOLVENT CDCl3
NS 1
DS 0
SWH 8012.820 Hz
FIDRES 0.244532 Hz
AQ 4.0894465 sec
RG 31.29
DW 62.400 usec
DE 10.00 usec
TE 300.0 K
D1 1.00000000 sec
TD0 1
SFO1 500.1730010 MHz
NUC1 1H
P0 4.00 usec
P1 12.00 usec
PLW1 16.00000000 W

F2 - Processing parameters
SI 65536
SF 500.1700122 MHz
WDW EM
SSB 0
LB 0.30 Hz
GB 0
PC 1.00

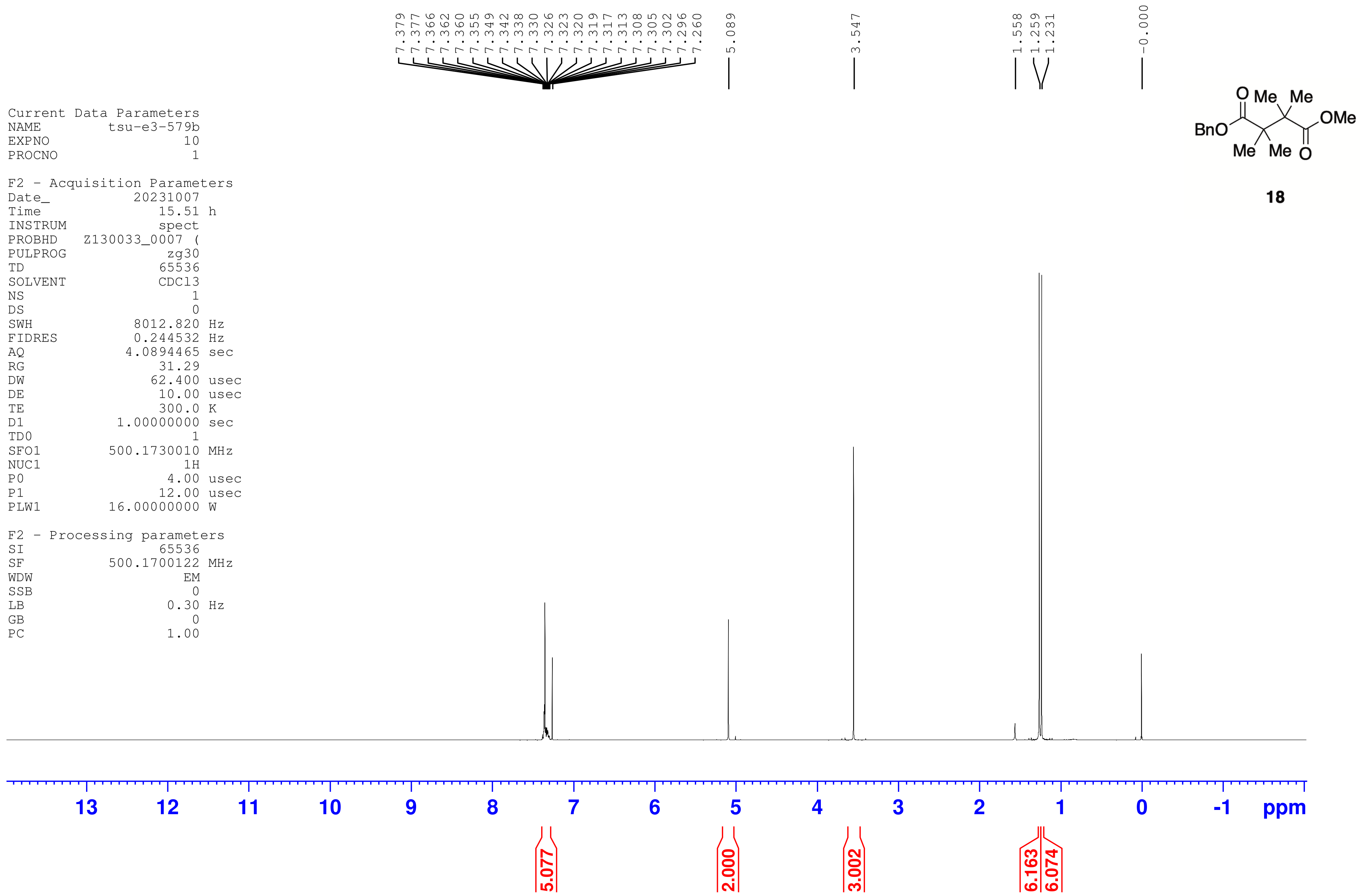

18

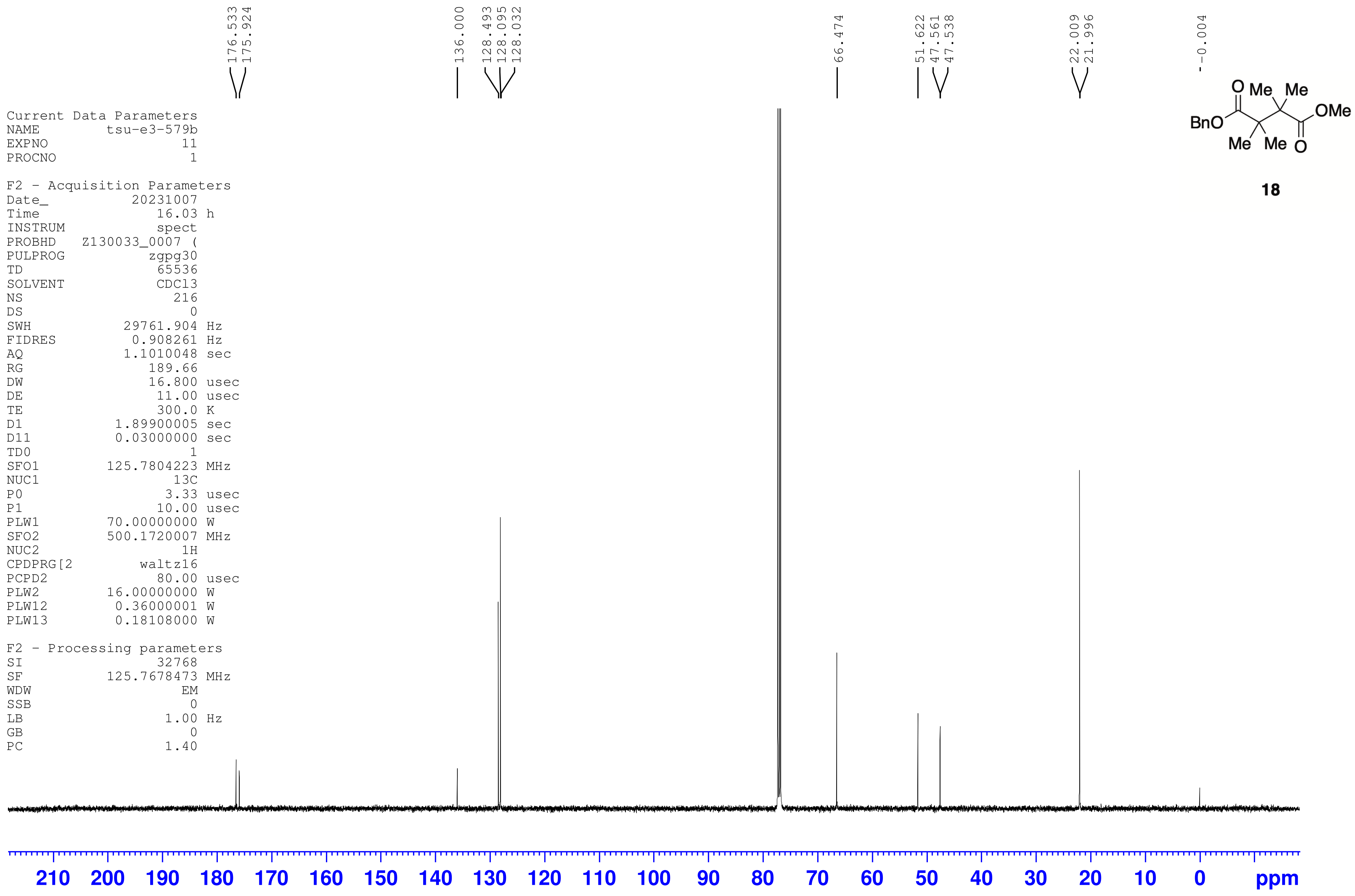
176.533
175.924
136.000
128.493
128.095
128.032
66.474
51.622
47.561
47.538
22.009
21.996
-0.004
Current Data Parameters
NAME tsu-e3-579b
EXPNO 11
PROCNO 1
F2 - Acquisition Parameters
Date_ 20231007
Time 16.03 h
INSTRUM spect
PROBHD Z130033_0007 (
PULPROG zgpg30
TD 65536
SOLVENT CDCl3
NS 216
DS 0
SWH 29761.904 Hz
FIDRES 0.908261 Hz
AQ 1.1010048 sec
RG 189.66
DW 16.800 usec
DE 11.00 usec
TE 300.0 K
D1 1.89900005 sec
D11 0.03000000 sec
TD0 1
SFO1 125.7804223 MHz
NUC1 13C
P0 3.33 usec
P1 10.00 usec
PLW1 70.00000000 W
SFO2 500.1720007 MHz
NUC2 1H
CPDPRG[2 waltz16
PCPD2 80.00 usec
PLW2 16.00000000 W
PLW12 0.36000001 W
PLW13 0.18108000 W
F2 - Processing parameters
SI 32768
SF 125.7678473 MHz
WDW EM
SSB 0
LB 1.00 Hz
GB 0
PC 1.40
210 200 190 180 170 160 150 140 130 120 110 100 90 80 70 60 50 40 30 20 10 0 ppm
BnO
O
Me
Me
OMe
Me
Me
O

18

Current Data Parameters
NAME FKI-E-05-0033 CD3OD
EXPNO 10
PROCNO 1

F2 - Acquisition Parameters
Date_ 20240811
Time 0.31 h
INSTRUM spect
PROBHD Z130033_0007 (
PULPROG zg30
TD 65536
SOLVENT MeOD
NS 1
DS 0
SWH 8012.820 Hz
FIDRES 0.244532 Hz
AQ 4.0894465 sec
RG 31.29
DW 62.400 usec
DE 10.00 usec
TE 300.0 K
D1 1.00000000 sec
TD0 1
SFO1 500.1730010 MHz
NUC1 1H
P0 4.00 usec
P1 12.00 usec
PLW1 16.00000000 W

F2 - Processing parameters
SI 65536
SF 500.1700599 MHz
WDW EM
SSB 0
LB 0.30 Hz
GB 0
PC 1.00

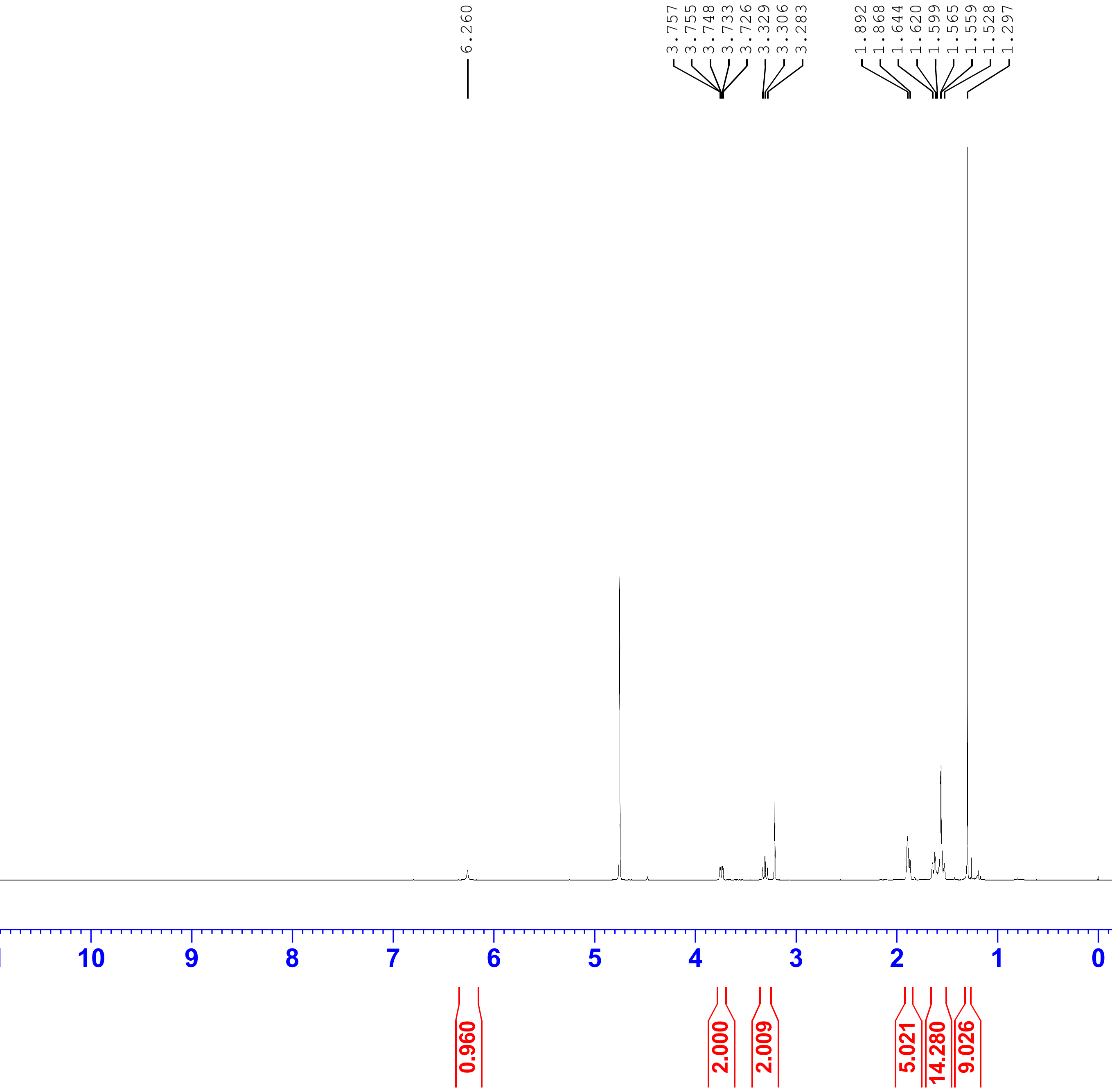

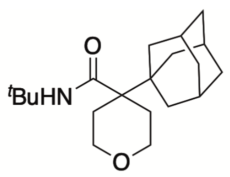

19

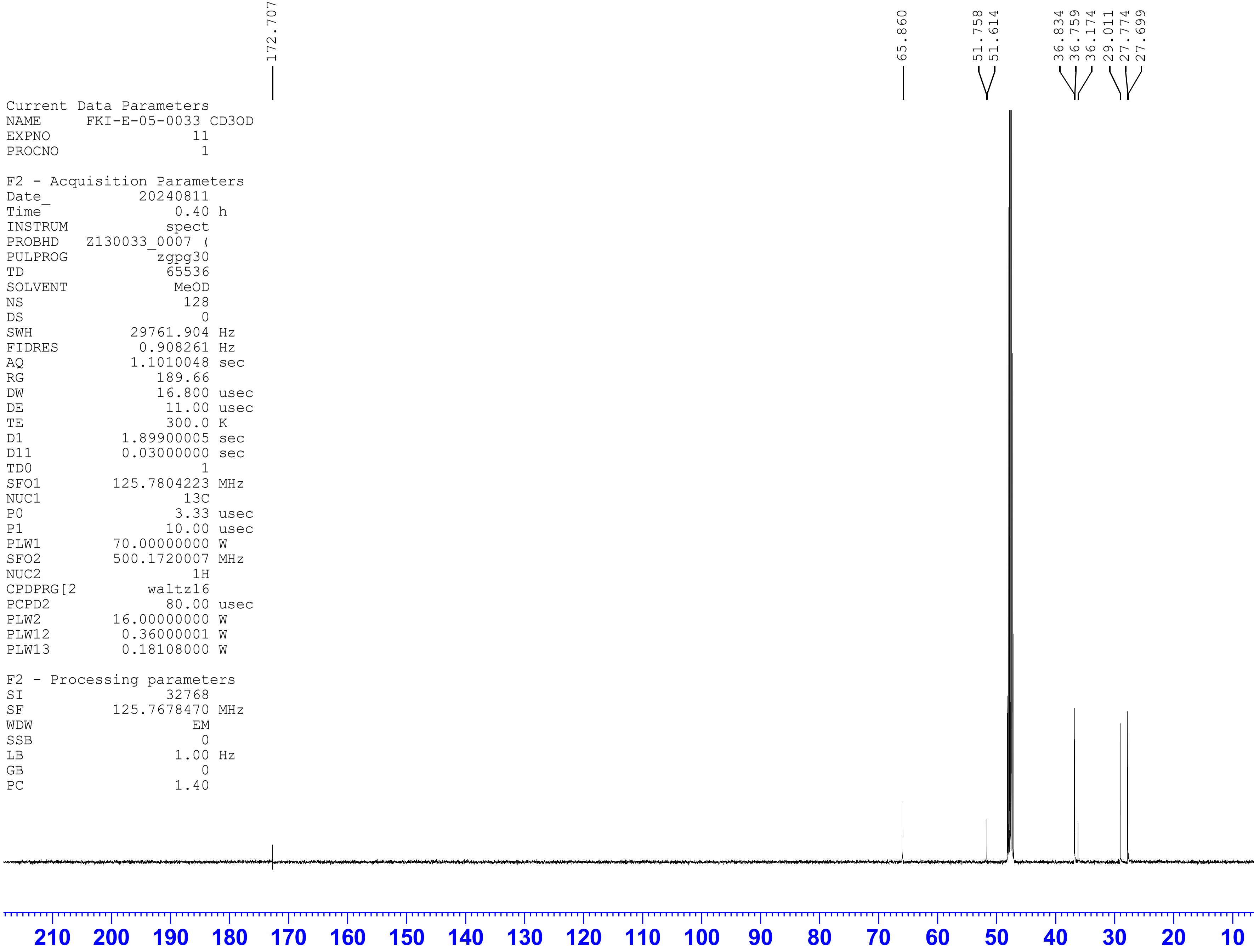

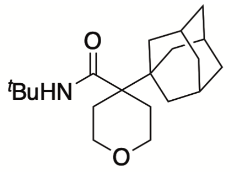

19

```
Current Data Parameters
NAME        FKI-E-05-0033 CD3OD
EXPNO                   11
PROCNO                   1

F2 - Acquisition Parameters
Date_            20240811
Time                 0.40 h
INSTRUM             spect
PROBHD   Z130033_0007 (
PULPROG           zgpg30
TD                 65536
SOLVENT             MeOD
NS                   128
DS                     0
SWH            29761.904 Hz
FIDRES          0.908261 Hz
AQ             1.1010048 sec
RG                189.66
DW                16.800 usec
DE                 11.00 usec
TE                 300.0 K
D1            1.89900005 sec
D11           0.03000000 sec
TD0                    1
SFO1         125.7804223 MHz
NUC1                 13C
P0                  3.33 usec
P1                 10.00 usec
PLW1         70.00000000 W
SFO2         500.1720007 MHz
NUC2                  1H
CPDPRG[2         waltz16
PCPD2              80.00 usec
PLW2         16.00000000 W
PLW12         0.36000001 W
PLW13         0.18108000 W

F2 - Processing parameters
SI                 32768
SF           125.7678470 MHz
WDW                   EM
SSB                    0
LB                  1.00 Hz
GB                     0
PC                  1.40
```

Current Data Parameters
NAME FKI-E-05-0042 acetone
EXPNO 10
PROCNO 1

F2 - Acquisition Parameters
Date_ 20250505
Time_ 13.34 h
INSTRUM spect
PROBHD Z130033_0007 (
PULPROG zg30
TD 65536
SOLVENT Acetone
NS 1
DS 0
SWH 8012.820 Hz
FIDRES 0.244532 Hz
AQ 4.0894465 sec
RG 31.29
DW 62.400 usec
DE 10.00 usec
TE 300.0 K
D1 1.00000000 sec
TD0 1
SFO1 500.1730010 MHz
NUC1 1H
P0 4.00 usec
P1 12.00 usec
PLW1 16.00000000 W

F2 - Processing parameters
SI 65536
SF 500.1700074 MHz
WDW EM
SSB 0
LB 0.30 Hz
GB 0
PC 1.00

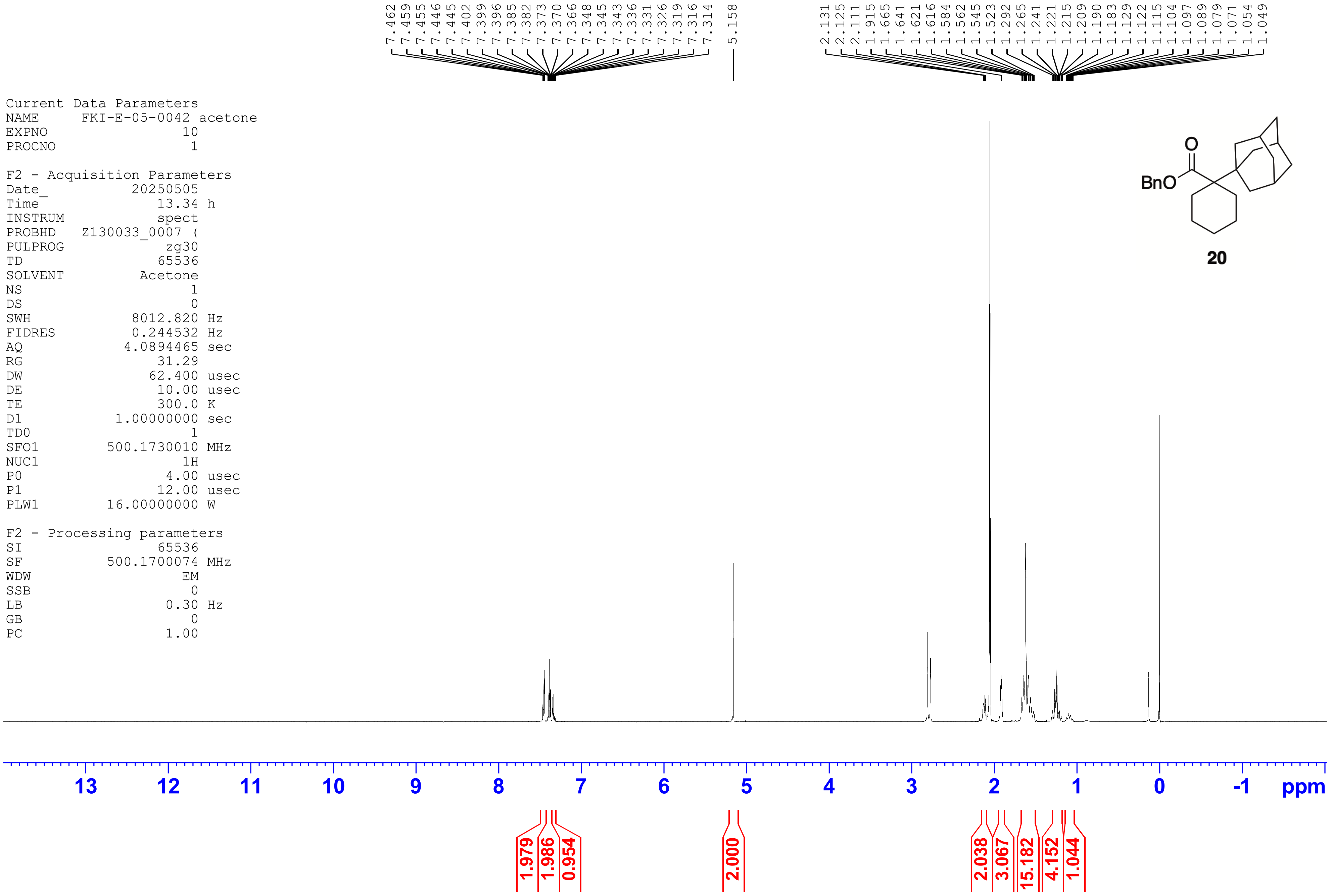

20

Current Data Parameters
NAME FKI-E-05-0042 acetone
EXPNO 10
PROCNO 1

F2 - Acquisition Parameters
Date_ 20250505
Time_ 13.34 h
INSTRUM spect
PROBHD Z130033_0007 (
PULPROG zg30
TD 65536
SOLVENT Acetone
NS 1
DS 0
SWH 8012.820 Hz
FIDRES 0.244532 Hz
AQ 4.0894465 sec
RG 31.29
DW 62.400 usec
DE 10.00 usec
TE 300.0 K
D1 1.00000000 sec
TD0 1
SFO1 500.1730010 MHz
NUC1 1H
P0 4.00 usec
P1 12.00 usec
PLW1 16.00000000 W

F2 - Processing parameters
SI 65536
SF 500.1700074 MHz
WDW EM
SSB 0
LB 0.30 Hz
GB 0
PC 1.00

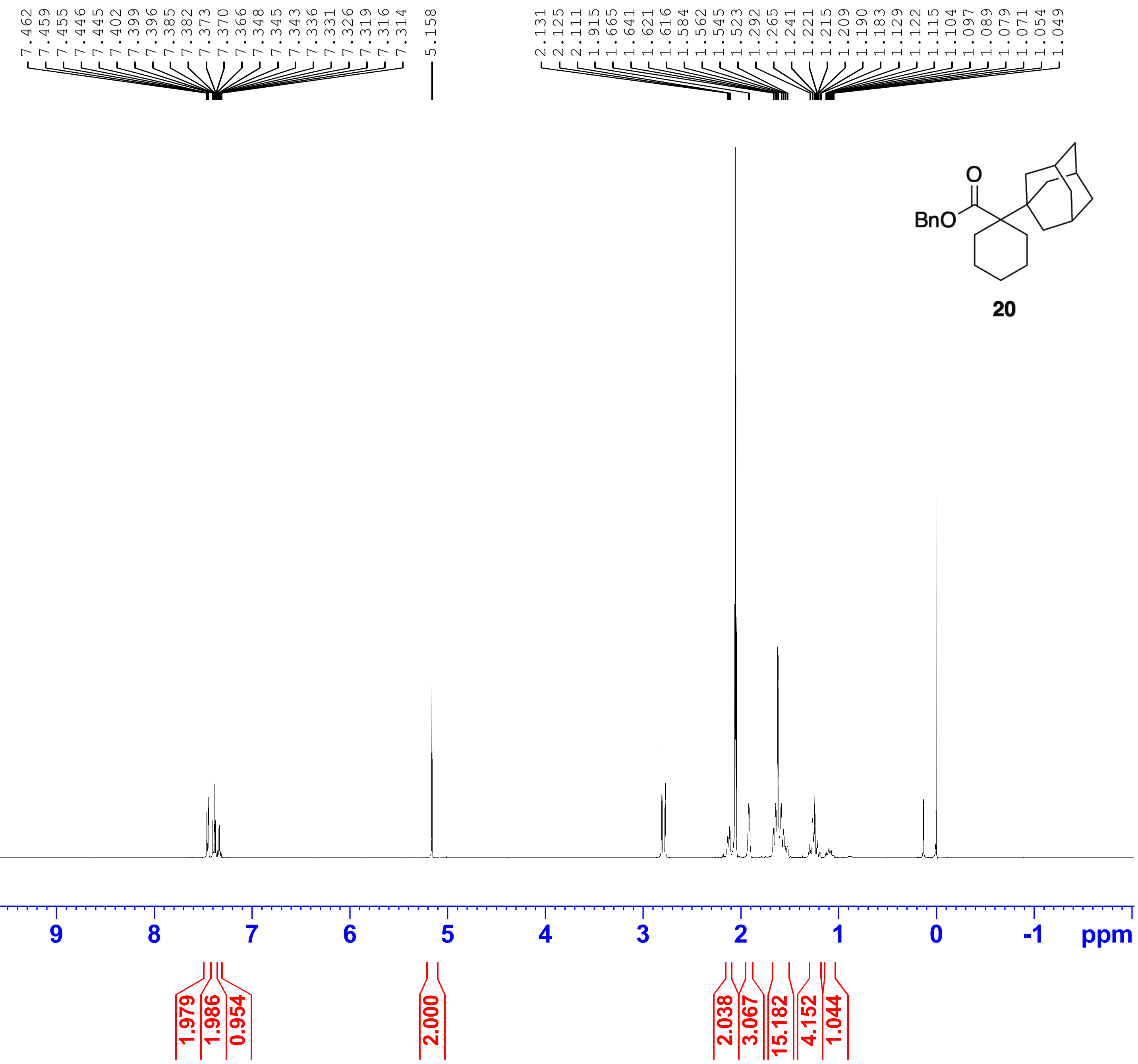

20

S228

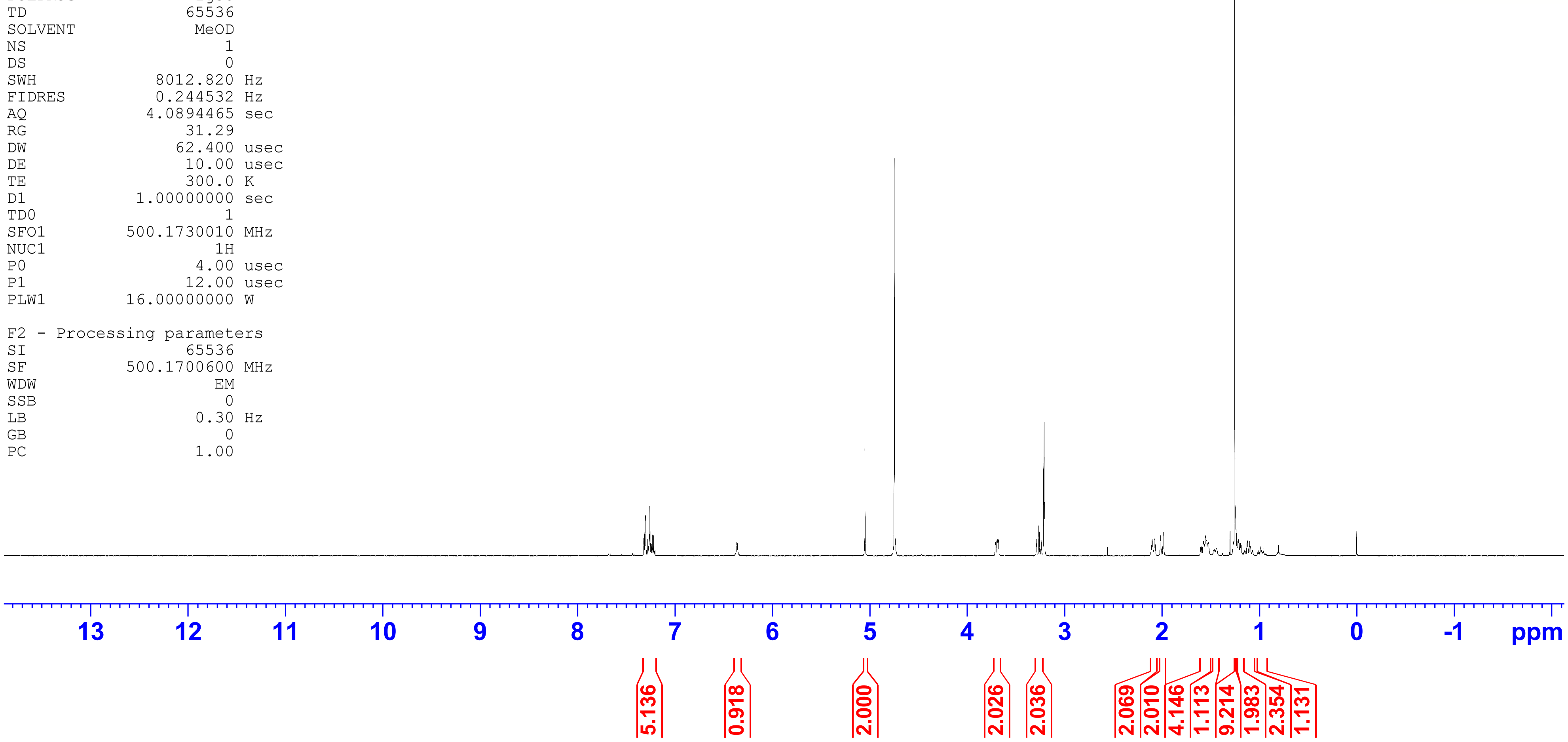
7.318
7.315
7.301
7.299
7.278
7.276
7.272
7.261
7.258
7.250
7.247
7.236
7.233
7.230
7.225
7.219
7.213
7.207
7.205
7.202
6.361
5.049
3.709
3.701
3.686
3.678
3.285
3.261
3.239
2.098
2.072
2.013
1.985
1.602
1.593
1.575
1.568
1.550
1.541
1.525
1.464
1.443
1.439
1.252
1.218
1.213
1.211
1.208
1.205
1.198
1.189
1.160
1.154
1.148
1.142
1.122
1.101
1.095
1.089
1.075
1.069
1.017
1.011
1.003
0.992
Current Data Parameters
NAME FKI-E-05-0041 TM
EXPNO 10
PROCNO 1
F2 - Acquisition Parameters
Date_ 20241003
Time 11.56 h
INSTRUM spect
PROBHD Z130033_0007 (
PULPROG zg30
TD 65536
SOLVENT MeOD
NS 1
DS 0
SWH 8012.820 Hz
FIDRES 0.244532 Hz
AQ 4.0894465 sec
RG 31.29
DW 62.400 usec
DE 10.00 usec
TE 300.0 K
D1 1.00000000 sec
TD0 1
SFO1 500.1730010 MHz
NUC1 1H
P0 4.00 usec
P1 12.00 usec
PLW1 16.00000000 W
F2 - Processing parameters
SI 65536
SF 500.1700600 MHz
WDW EM
SSB 0
LB 0.30 Hz
GB 0
PC 1.00
13
12
11
10
9
8
7
6
5
4
3
2
1
0
-1
ppm
5.136
0.918
2.000
2.026
2.036
2.069
2.010
4.146
1.113
9.214
1.983
2.354
1.131

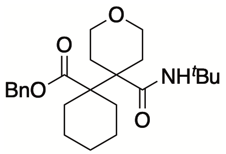
BnO
O
O
NH^{t}Bu
O

21

Current Data Parameters
NAME FKI-E-05-0041 CNMR
EXPNO 10
PROCNO 1

F2 - Acquisition Parameters
Date_ 20241003
Time 16.25 h
INSTRUM spect
PROBHD Z130033_0007 (
PULPROG zgpg30
TD 65536
SOLVENT MeOD
NS 128
DS 0
SWH 29761.904 Hz
FIDRES 0.908261 Hz
AQ 1.1010048 sec
RG 189.66
DW 16.800 usec
DE 11.00 usec
TE 300.0 K
D1 1.89900005 sec
D11 0.03000000 sec
TD0 1
SFO1 125.7804223 MHz
NUC1 13C
P0 3.33 usec
P1 10.00 usec
PLW1 70.00000000 W
SFO2 500.1720007 MHz
NUC2 1H
CPDPRG[2 waltz16
PCPD2 80.00 usec
PLW2 16.00000000 W
PLW12 0.36000001 W
PLW13 0.18108000 W

F2 - Processing parameters
SI 32768
SF 125.7678470 MHz
WDW EM
SSB 0
LB 1.00 Hz
GB 0
PC 1.40

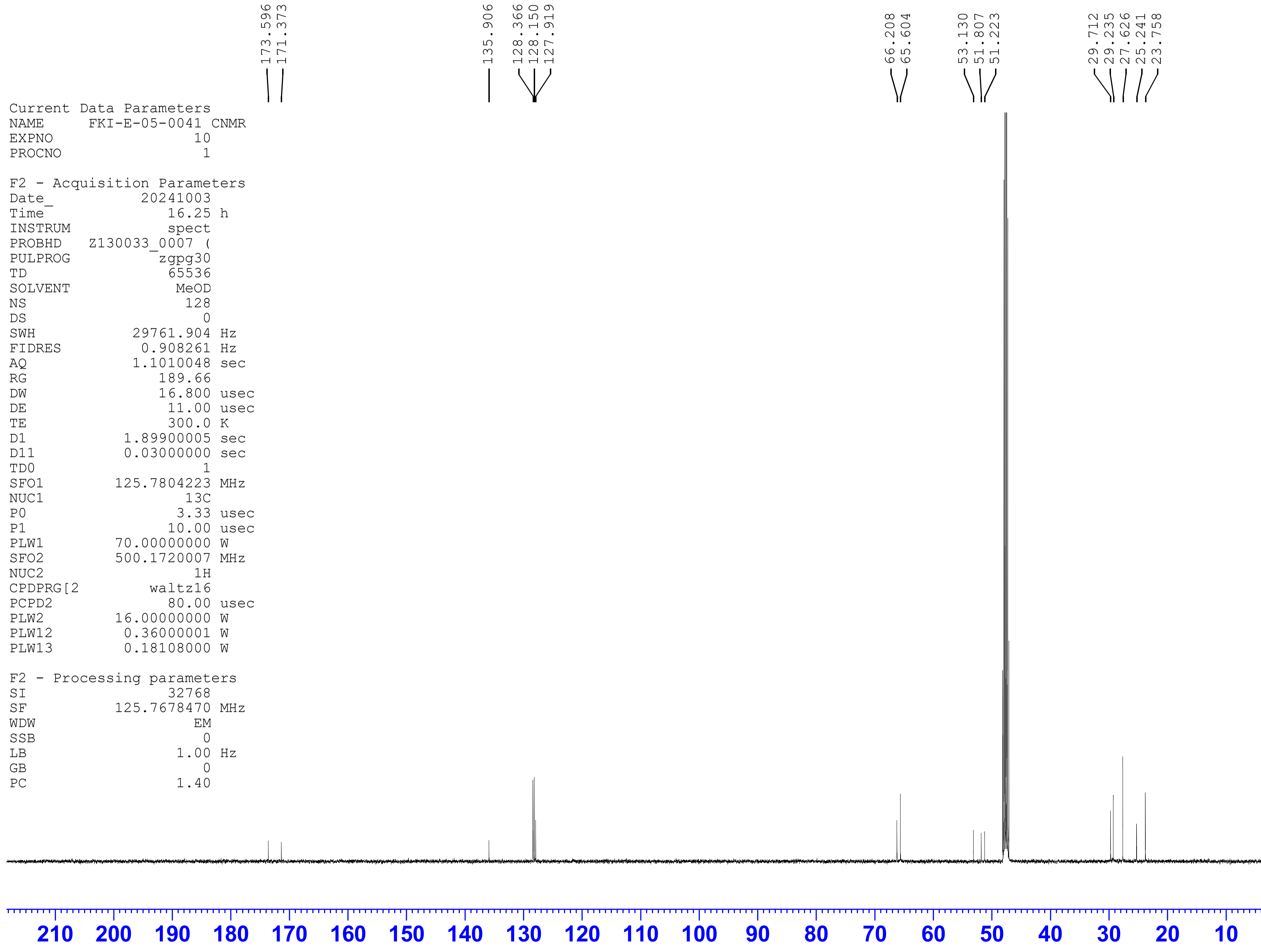

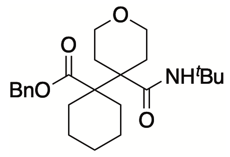

21

```
Current Data Parameters
NAME       tsu-e3-594 crude
EXPNO                    10
PROCNO                    1

F2 - Acquisition Parameters
Date_            20231024
Time                14.32 h
INSTRUM             spect
PROBHD    Z130033_0007 (
PULPROG              zg30
TD                  65536
SOLVENT             CDCl3
NS                      1
DS                      0
SWH              8012.820 Hz
FIDRES           0.244532 Hz
AQ              4.0894465 sec
RG                  31.29
DW                 62.400 usec
DE                  10.00 usec
TE                  300.1 K
D1             1.00000000 sec
TD0                     1
SFO1          500.1730010 MHz
NUC1                   1H
P0                   4.00 usec
P1                  12.00 usec
PLW1          16.00000000 W

F2 - Processing parameters
SI                  65536
SF            500.1700122 MHz
WDW                    EM
SSB                     0
LB                   0.30 Hz
GB                      0
PC                   1.00
```

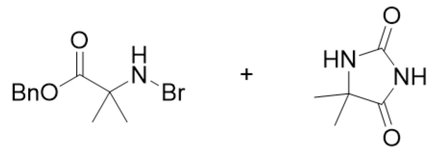

IM1

[1]H: 8.10 (br, 1H, N*H*)
5.78 (br, 1H, N*H*)
1.47 (s, 6H, C*H*$_3$)

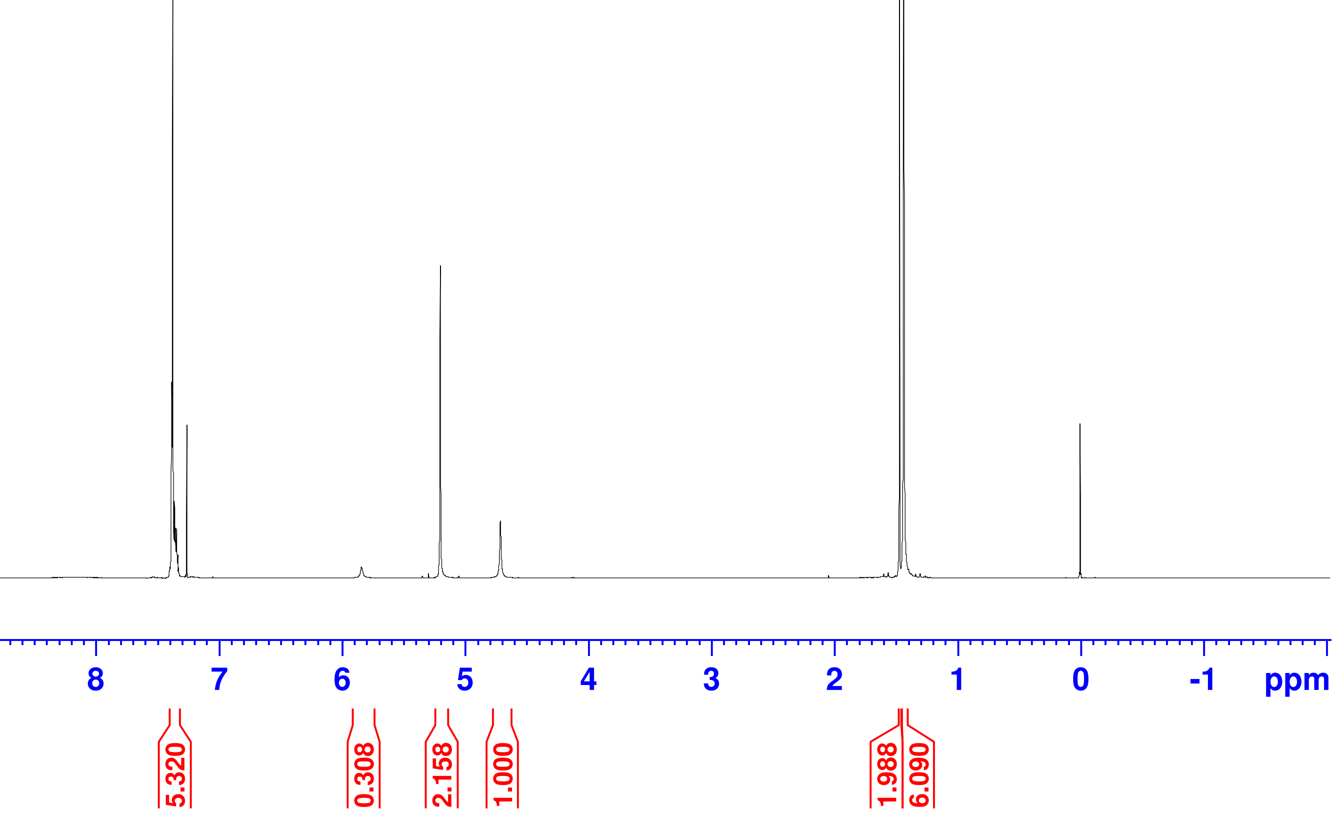

S231

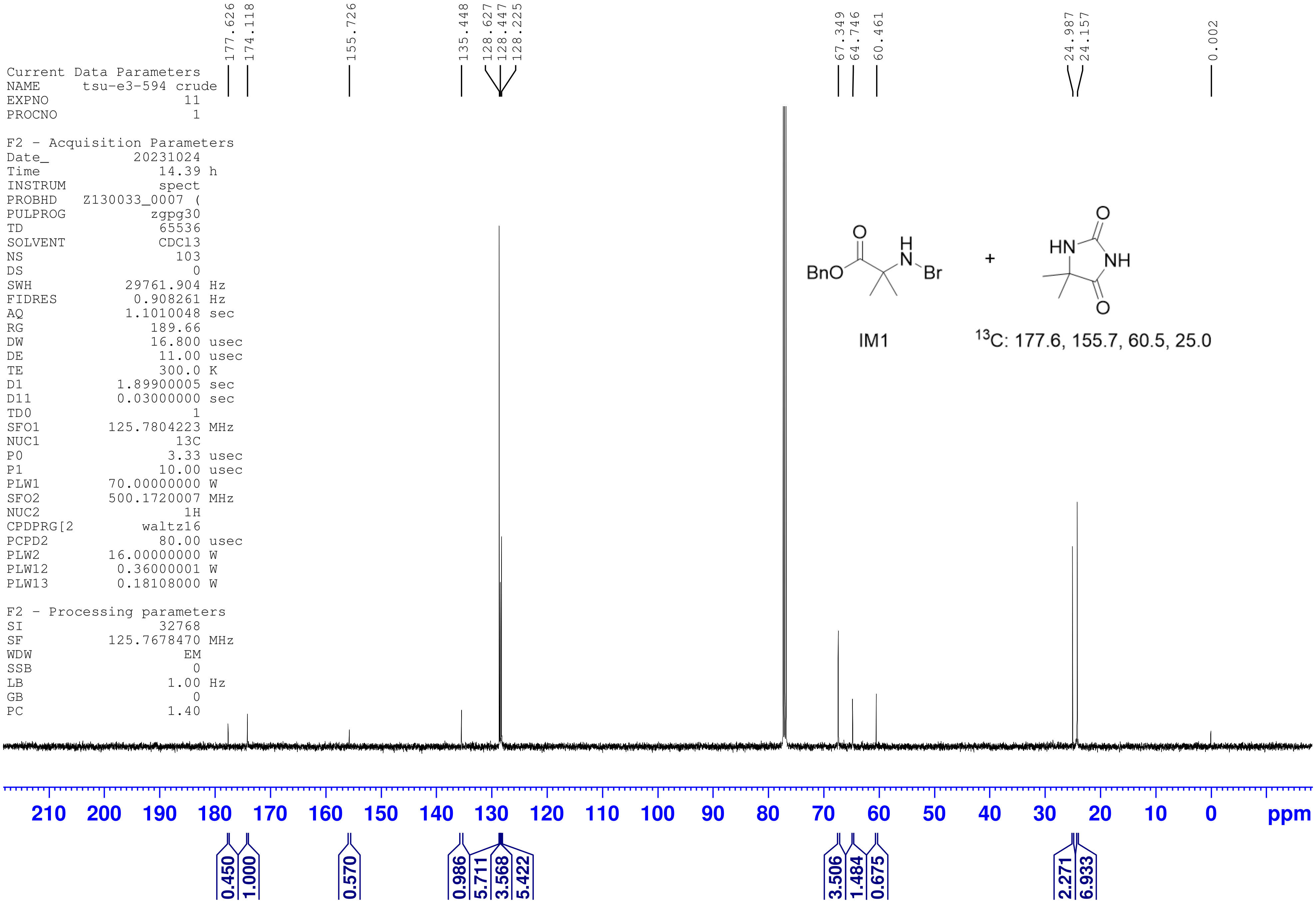


```
Current Data Parameters
NAME     tsu-e3-594 crude
EXPNO                  11
PROCNO                  1

F2 - Acquisition Parameters
Date_          20231024
Time              14.39 h
INSTRUM           spect
PROBHD   Z130033_0007 (
PULPROG          zgpg30
TD                65536
SOLVENT           CDCl3
NS                  103
DS                    0
SWH           29761.904 Hz
FIDRES         0.908261 Hz
AQ            1.1010048 sec
RG               189.66
DW               16.800 usec
DE                11.00 usec
TE                300.0 K
D1           1.89900005 sec
D11          0.03000000 sec
TD0                   1
SFO1        125.7804223 MHz
NUC1                13C
P0                 3.33 usec
P1                10.00 usec
PLW1        70.00000000 W
SFO2        500.1720007 MHz
NUC2                 1H
CPDPRG[2        waltz16
PCPD2             80.00 usec
PLW2        16.00000000 W
PLW12        0.36000001 W
PLW13        0.18108000 W

F2 - Processing parameters
SI                32768
SF          125.7678470 MHz
WDW                  EM
SSB                   0
LB                 1.00 Hz
GB                    0
PC                 1.40
```

^{13}C: 177.6, 155.7, 60.5, 25.0

Current Data Parameters	
NAME	hydantoin
EXPNO	10
PROCNO	1
F2 - Acquisition Parameters	
Date_	20231028
Time	16.31 h
INSTRUM	spect
PROBHD	Z130033_0007 (
PULPROG	zg30
TD	65536
SOLVENT	CDCl3
NS	1
DS	0
SWH	8012.820 Hz
FIDRES	0.244532 Hz
AQ	4.0894465 sec
RG	31.29
DW	62.400 usec
DE	10.00 usec
TE	300.1 K
D1	1.00000000 sec
TD0	1
SFO1	500.1730010 MHz
NUC1	1H
P0	4.00 usec
P1	12.00 usec
PLW1	16.00000000 W
F2 - Processing parameters	
SI	65536
SF	500.1700111 MHz
WDW	EM
SSB	0
LB	0.30 Hz
GB	0
PC	1.00

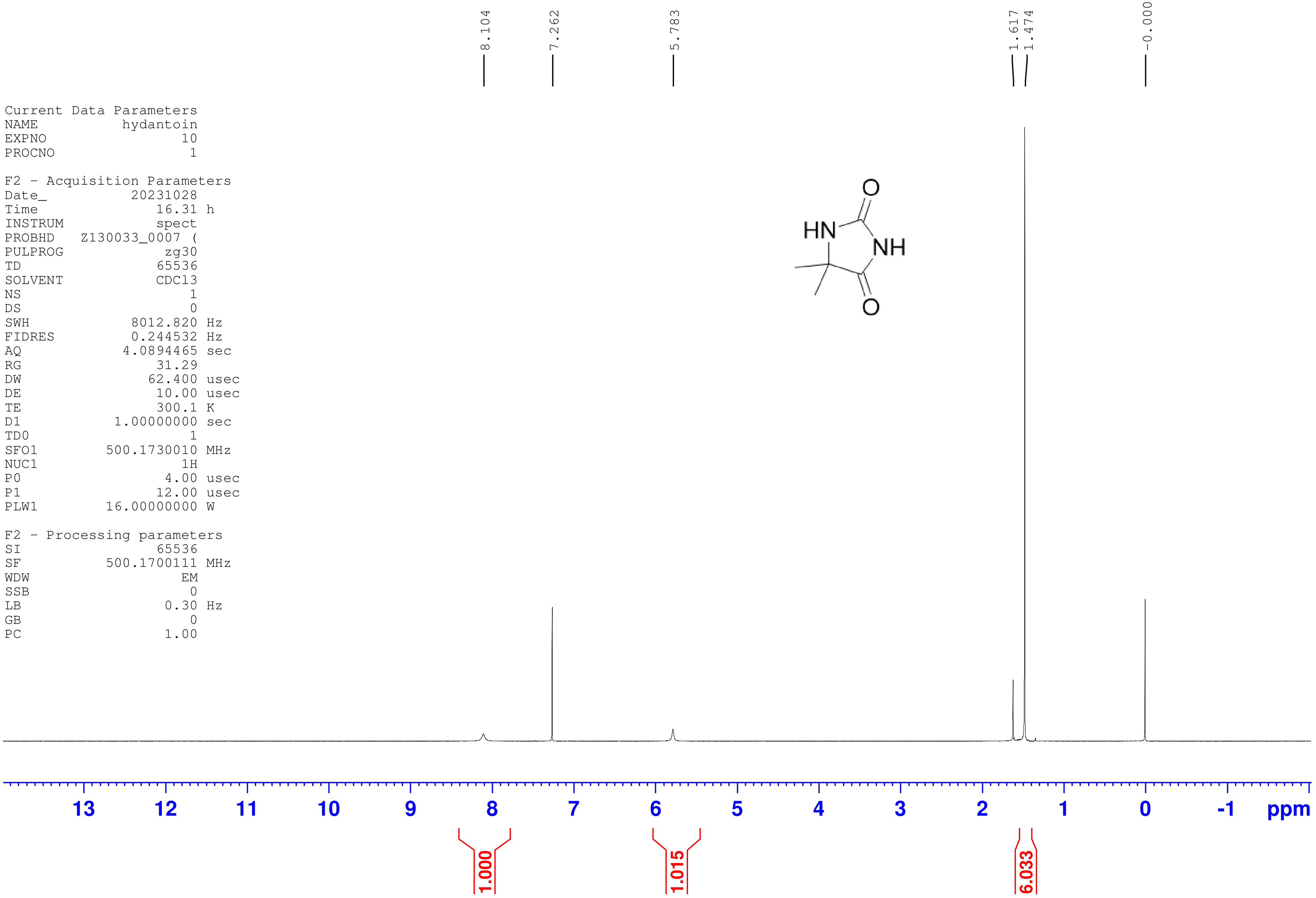

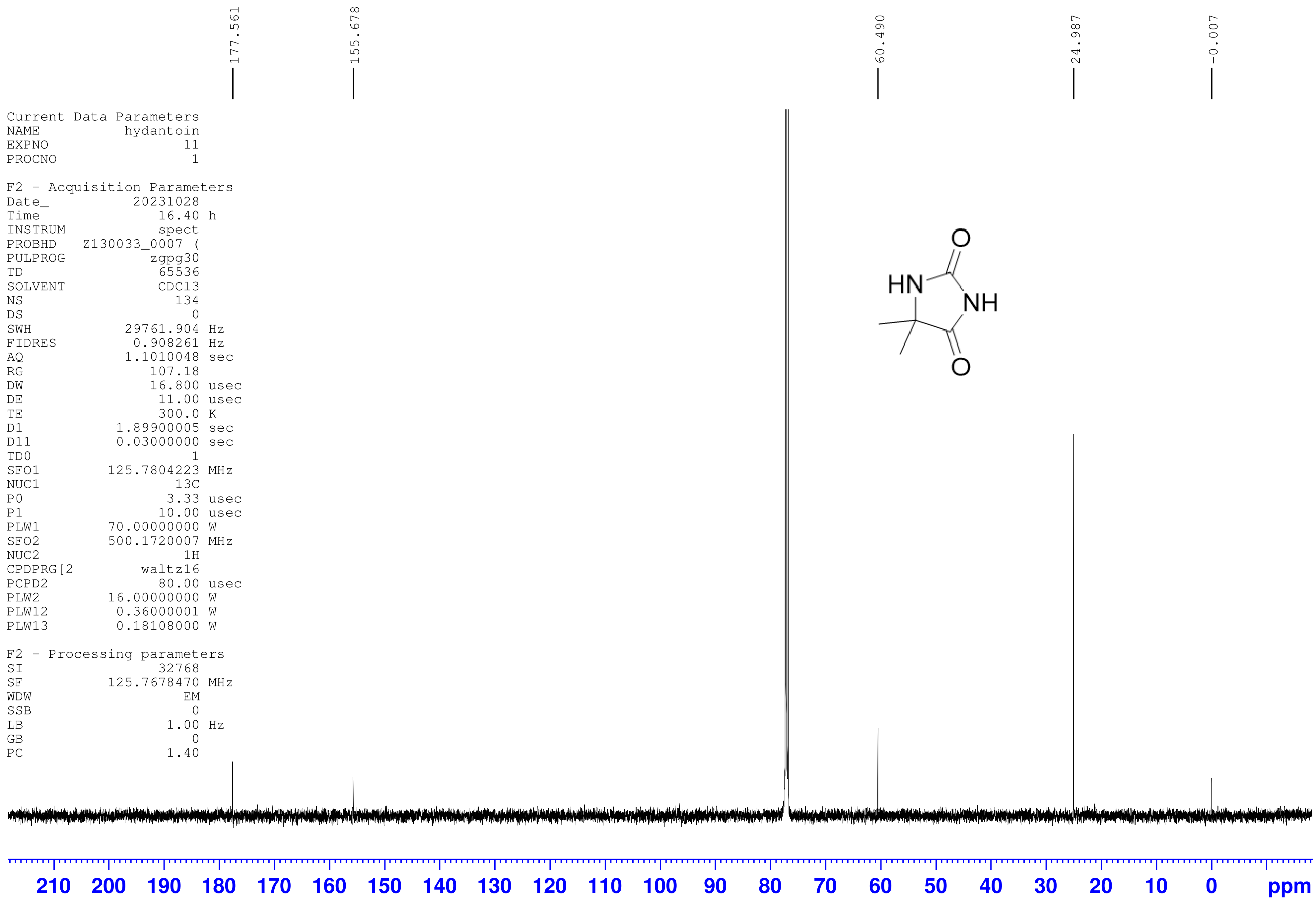
177.561
155.678
60.490
24.987
-0.007
Current Data Parameters
NAME hydantoin
EXPNO 11
PROCNO 1
F2 - Acquisition Parameters
Date_ 20231028
Time 16.40 h
INSTRUM spect
PROBHD Z130033_0007 (
PULPROG zgpg30
TD 65536
SOLVENT CDCl3
NS 134
DS 0
SWH 29761.904 Hz
FIDRES 0.908261 Hz
AQ 1.1010048 sec
RG 107.18
DW 16.800 usec
DE 11.00 usec
TE 300.0 K
D1 1.89900005 sec
D11 0.03000000 sec
TD0 1
SFO1 125.7804223 MHz
NUC1 13C
P0 3.33 usec
P1 10.00 usec
PLW1 70.00000000 W
SFO2 500.1720007 MHz
NUC2 1H
CPDPRG[2 waltz16
PCPD2 80.00 usec
PLW2 16.00000000 W
PLW12 0.36000001 W
PLW13 0.18108000 W
F2 - Processing parameters
SI 32768
SF 125.7678470 MHz
WDW EM
SSB 0
LB 1.00 Hz
GB 0
PC 1.40
HN
NH
O
O
210
200
190
180
170
160
150
140
130
120
110
100
90
80
70
60
50
40
30
20
10
0
ppm

Current Data Parameters
NAME tsu-e3-565
EXPNO 10
PROCNO 1

F2 - Acquisition Parameters
Date_ 20230929
Time 14.20 h
INSTRUM spect
PROBHD Z130033_0007 (
PULPROG zg30
TD 65536
SOLVENT CDCl3
NS 1
DS 0
SWH 8012.820 Hz
FIDRES 0.244532 Hz
AQ 4.0894465 sec
RG 31.29
DW 62.400 usec
DE 10.00 usec
TE 300.1 K
D1 1.00000000 sec
TD0 1
SFO1 500.1730010 MHz
NUC1 1H
P0 4.00 usec
P1 12.00 usec
PLW1 16.00000000 W

F2 - Processing parameters
SI 65536
SF 500.1700123 MHz
WDW EM
SSB 0
LB 0.30 Hz
GB 0
PC 1.00

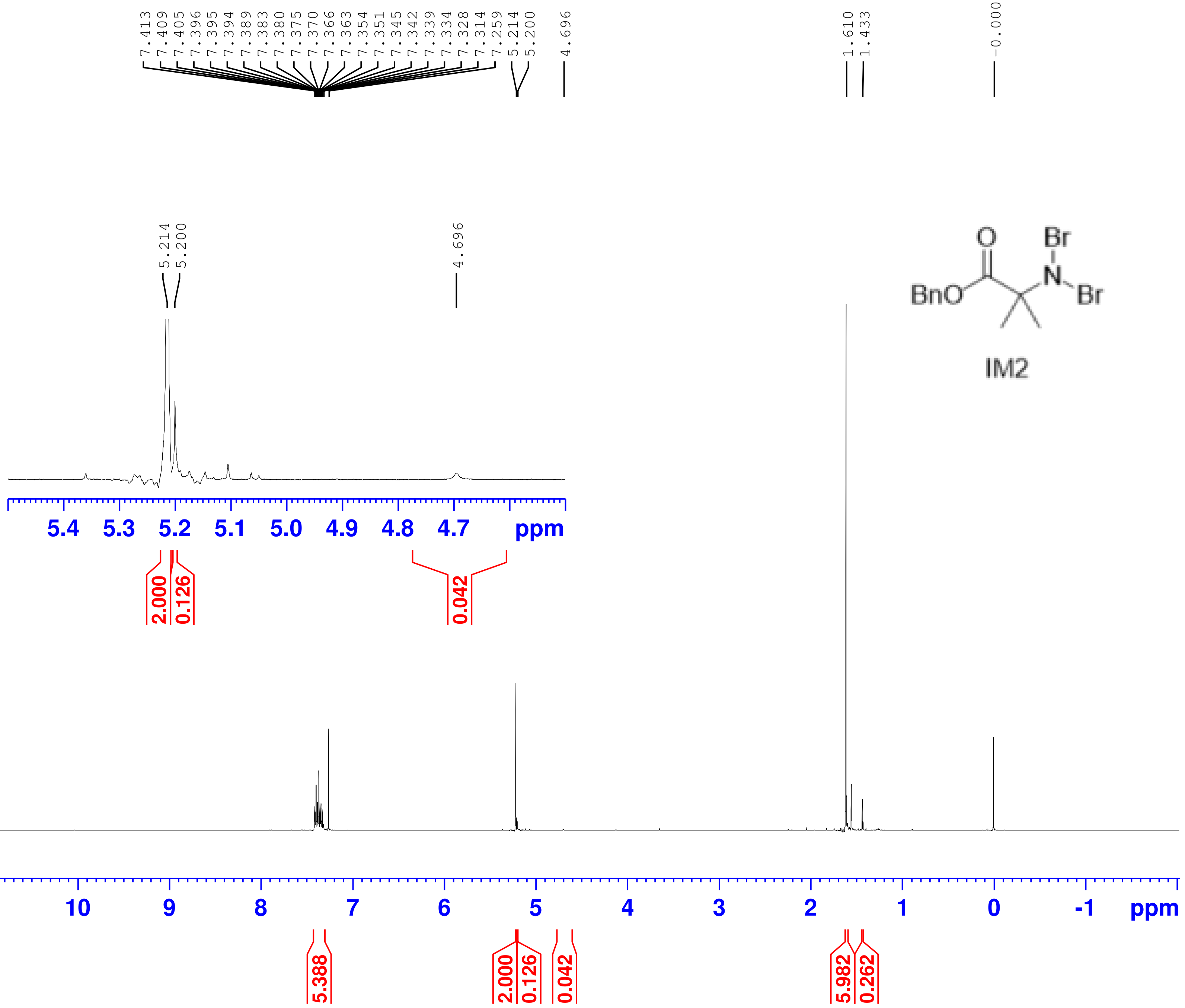

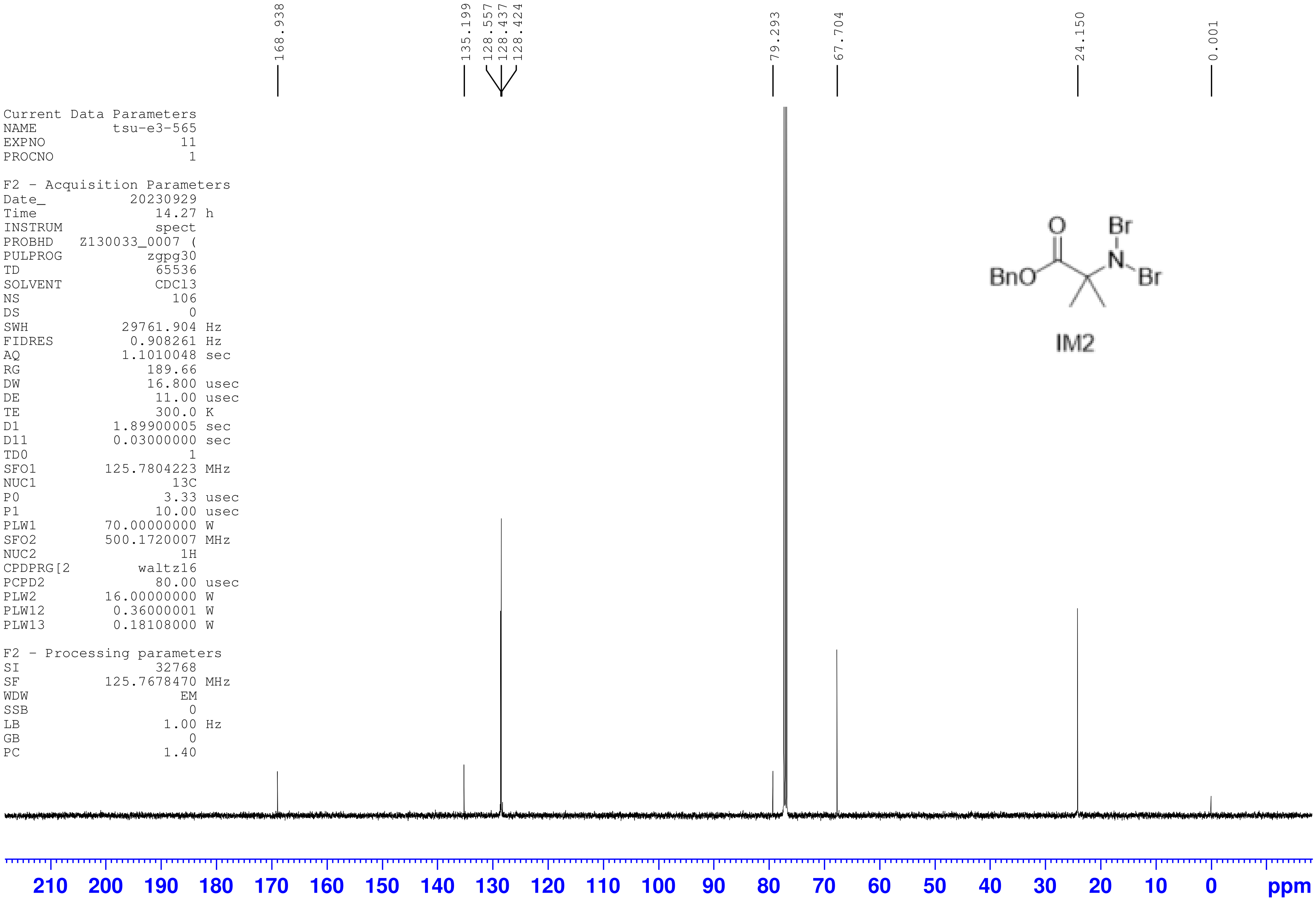

Current Data Parameters	
NAME	tsu-e3-565
EXPNO	11
PROCNO	1
F2 - Acquisition Parameters	
Date_	20230929
Time	14.27 h
INSTRUM	spect
PROBHD	Z130033_0007 (
PULPROG	zgpg30
TD	65536
SOLVENT	CDCl3
NS	106
DS	0
SWH	29761.904 Hz
FIDRES	0.908261 Hz
AQ	1.1010048 sec
RG	189.66
DW	16.800 usec
DE	11.00 usec
TE	300.0 K
D1	1.89900005 sec
D11	0.03000000 sec
TD0	1
SFO1	125.7804223 MHz
NUC1	13C
P0	3.33 usec
P1	10.00 usec
PLW1	70.00000000 W
SFO2	500.1720007 MHz
NUC2	1H
CPDPRG[2	waltz16
PCPD2	80.00 usec
PLW2	16.00000000 W
PLW12	0.36000001 W
PLW13	0.18108000 W
F2 - Processing parameters	
SI	32768
SF	125.7678470 MHz
WDW	EM
SSB	0
LB	1.00 Hz
GB	0
PC	1.40

IM2

14. Reference

1. Xiong, X. F. *et al.* Total synthesis and structure-activity relationship studies of a series of selective G protein inhibitors. *Nat. Chem.* **8**, 1035–1041 (2016).
2. Fujimoto, J. *et al.* Discovery of 3,5-Diphenyl-4-methyl-1,3-oxazolidin-2-ones as Novel, Potent, and Orally Available Δ-5 Desaturase (D5D) Inhibitors. *J. Med. Chem.* **60**, 8963–8981 (2017).
3. Frisch, H. *et al.* pH-Switchable Ampholytic Supramolecular Copolymers. *Angew. Chemie Int. Ed.* **52**, 10097–10101 (2013).
4. Hogg, K. F., Trowbridge, A., Alvarez-Pérez, A. & Gaunt, M. J. The α-tertiary amine motif drives remarkable selectivity for Pd-catalyzed carbonylation of β-methylene C–H bonds. *Chem. Sci.* **8**, 8198–8203 (2017).
5. Mir, F. M., Crisma, M., Toniolo, C. & Lubell, W. D. Influence of the C-terminal substituent on the crystal-state conformation of Adm peptides. *Pept. Sci.* **112**, (2020).
6. Yamamoto, H. *et al.* Orally active cephalosporins. Part 3: synthesis, structure–activity relationships and oral absorption of novel C-3 heteroarylmethylthio cephalosporins. *Bioorg. Med. Chem.* **9**, 465–475 (2001).
7. Hugelshofer, C. L. *et al.* Scalable Preparation of 4,4-Disubstituted Six-Membered Cyclic Sulfones. *Org. Lett.* **23**, 943–947 (2021).
8. Tsuji, T. *et al.* α-Amino acid and peptide synthesis using catalytic cross-dehydrogenative coupling. *Nat. Synth.* **1**, 304–312 (2022).
9. Summers, J. B. *et al.* Hydroxamic acid inhibitors of 5-lipoxygenase. *J. Med. Chem.* **30**, 574–580 (1987).
10. Raghuvanshi, D. S. & Verma, N. An iodine-mediated new avenue to sulfonylation employing N -hydroxy aryl sulfonamide as a sulfonylating agent. *Org. Biomol. Chem.* **19**, 4760–4767 (2021).
11. Fan, T. *et al.* Visible-Light-Induced Tandem Reaction of Allenes with Selenesulfonates Leading to (E)-2,3-Disulfonylpropene Derivatives. *J. Org. Chem.* **87**, 5846–5855 (2022).
12. Huang, H., Zhang, G. & Chen, Y. Dual Hypervalent Iodine(III) Reagents and Photoredox Catalysis Enable Decarboxylative Ynonylation under Mild Conditions. *Angew. Chemie Int. Ed.* **54**, 7872–7876 (2015).
13. Piel, I., Pawelczyk, M. D., Hirano, K., Fröhlich, R. & Glorius, F. A Family of Thiazolium Salt Derived N-Heterocyclic Carbenes (NHCs) for Organocatalysis: Synthesis, Investigation and Application in Cross-Benzoin Condensation. *European J. Org. Chem.* 5475–5484 (2011) doi:10.1002/ejoc.201100870.
14. Li, B., Shi, Y. & Fu, Z. Schiff base as a novel kind of catalyst for reversible complexation-mediated radical polymerization of methyl methacrylate. *J. Polym. Sci. Part A Polym. Chem.* **57**, 1653–1663 (2019).
15. Zhang, X. & Huang, H. Copper-Catalyzed Oxidative Coupling of AIBN and Ketone-Derived Enoxysilanes to γ-Ketonitriles. *Org. Lett.* **20**, 4998–5001 (2018).
16. Wang, J.-M., Chen, T., Yao, C.-S. & Zhang, K. Synthesis of β-Ketonitriles via N -Heterocyclic-Carbene-Catalyzed Radical Coupling of Aldehydes and Azobis(isobutyronitrile). *Org. Lett.* **25**, 3325–3329 (2023).
17. Movassaghi, M., Ahmad, O. K. & Lathrop, S. P. Directed Heterodimerization: Stereocontrolled Assembly via Solvent-Caged Unsymmetrical Diazene Fragmentation. *J. Am. Chem. Soc.* **133**, 13002–13005 (2011).
18. Frisch, M. J.; Trucks, G. W.; Schlegel, H. B.; Scuseria, G. E.; Robb, M. A.; Cheeseman, J. R.; Scalmani, G.; Barone, V.; Petersson, G. A.; Nakatsuji, H.; Li, X.; Caricato, M.; Marenich, A.; Bloino, J.; Janesko, B. G.;

Gomperts, R.; Mennucci, B.; Hratchian, H. P.; Ortiz, J. V.; Izmaylov, A. F.; Sonnenberg, J. L.; Williams-Young, D.; Ding, F.; Lipparini, F.; Egidi, F.; Goings, J.; Peng, B.; Petrone, A.; Henderson, T.; Ranasinghe, D.; Zakrzewski, V. G.; Gao, J.; Rega, N.; Zheng, G.; Liang, W.; Hada, M.; Ehara, M.; Toyota, K.; Fukuda, R.; Hasegawa, J.; Ishida, M.; Nakajima, T.; Honda, Y.; Kitao, O.; Nakai, H.; Vreven, T.; Throssell, K.; Montgomery, J. A., Jr.; Peralta, J. E.; Ogliaro, F.; Bearpark, M.; Heyd, J. J.; Brothers, E. N.; Kudin, K. N.; Staroverov, V. N.; Keith, T. A.; Kobayashi, R.; Normand, J.; Raghavachari, K.; Rendell, A. P.; Burant, J. C.; Iyengar, S. S.; Tomasi, J.; Cossi, M.; Millam, J. M.; Klene, M.; Adamo, C.; Cammi, R.; Ochterski, J. W.; Martin, R. L.; Morokuma, K.; Farkas, O.; Foresman, J. B.; Fox, D. J. Gaussian 16, revision A.03; Gaussian, Inc.: Wallingford, CT, 2016.

19. Becke, A. D. Density-functional thermochemistry. III. The role of exact exchange. *J. Chem. Phys.* **98**, 5648–5652 (1993).
20. Lee, C., Yang, W. & Parr, R. G. Development of the Colle-Salvetti correlation-energy formula into a functional of the electron density. *Phys. Rev. B* **37**, 785–789 (1988).
21. Vosko, S. H., Wilk, L. & Nusair, M. Accurate spin-dependent electron liquid correlation energies for local spin density calculations: a critical analysis. *Can. J. Phys.* **58**, 1200–1211 (1980).
22. Hay, P. J.; Wadt, W. R. Ab initio effective core potentials for molecular calculations. Potentials for the transition metal atoms Sc to Hg. *J. Chem. Phys.* **82**, 270–283 (1985).
23. Wadt, W. R.; Hay, P. J. Ab initio effective core potentials for molecular calculations. Potentials for main group elements Na to Bi. *J. Chem. Phys.* **82**, 284–298 (1985).
24. Zhao, Y. & Truhlar, D. G. The M06 suite of density functionals for main group thermochemistry, thermochemical kinetics, noncovalent interactions, excited states, and transition elements: two new functionals and systematic testing of four M06-class functionals and 12 other function. *Theor. Chem. Acc.* **120**, 215–241 (2008).
25. Marenich, A. V., Cramer, C. J. & Truhlar, D. G. Universal Solvation Model Based on Solute Electron Density and on a Continuum Model of the Solvent Defined by the Bulk Dielectric Constant and Atomic Surface Tensions. *J. Phys. Chem. B* **113**, 6378–6396 (2009).
26. Saito, N. *et al.* Functional Group Evaluation Kit for Digitalization of Information on the Functional Group Compatibility and Chemoselectivity of Organic Reactions. *Bull. Chem. Soc. Jpn.* **96**, 465–474 (2023).
27. Stoll, S., Schweiger, A. EasySpin, a comprehensive software package for spectral simulation and analysis in EPR. *J. Magn. Reson.* **178**, 42–55 (2006).
